# Characterization of limes (*Citrus aurantifolia*) grown in Bhutan and Indonesia using high-throughput sequencing

**DOI:** 10.1038/srep04853

**Published:** 2014-04-30

**Authors:** Tshering Penjor, Takashi Mimura, Ryoji Matsumoto, Masashi Yamamoto, Yukio Nagano

**Affiliations:** 1 Department of Applied Biological Sciences, Saga University, Honjo, Saga, Japan; 2 Renewable Natural Resources Research Centre Wengkhar, Mongar, Bhutan; 3 Faculty of Agriculture, Kagoshima University, Korimoto, Kagoshima, Japan; 4 Analytical Research Center for Experimental Sciences, Saga University, Honjo, Saga, Japan; 5 These authors contributed equally to this work.

## Abstract

Lime [*Citrus aurantifolia* (Cristm.) Swingle] is a *Citrus* species that is a popular ingredient in many cuisines. Some citrus plants are known to originate in the area ranging from northeastern India to southwestern China. In the current study, we characterized and compared limes grown in Bhutan (n = 5 accessions) and Indonesia (n = 3 accessions). The limes were separated into two groups based on their morphology. Restriction site-associated DNA sequencing (RAD-seq) separated the eight accessions into two clusters. One cluster contained four accessions from Bhutan, whereas the other cluster contained one accession from Bhutan and the three accessions from Indonesia. This genetic classification supported the morphological classification of limes. The analysis suggests that the properties associated with asexual reproduction, and somatic homologous recombination, have contributed to the genetic diversification of limes.

*Citrus* species, which include mandarin (*Citrus reticulata*), sweet orange (*C. sinensis*), lemon (*C. limon*), and grapefruit (*C. paradisi*), have high economic and nutritional value. Lime (synonym: West Indian lime, Mexican lime, and key lime), *C. aurantifolia* (Cristm.) Swingle, is a popular ingredient in the dishes and drinks of many countries because of its aroma and acidity. Assam, which is a northeastern state of India, is one of the candidate origins of some *Citrus* species, because numerous varieties of citrus are grown in this area^1^. Yunnan, which is a province located in southwestern China, is another candidate origin of some *Citrus* species^2^. Thus, the region from northeastern India to southwestern China is ideal for studying the diversity of *Citrus* species. Bhutan is located within this region. However, to date, the genetic resources for *Citrus* species have not been well characterized in this country^3^. Thus, it was chosen as one of our two study sites. Limes are also widely cultivated and consumed in Indonesia, with varieties from this region being introduced to Saga University, Japan, as budwoods in 1988. Thus, we chose Indonesia as the second of our two study sites.

Lime is often considered to be a chance seedling, with citron (*C. medica*) and papeda possibly being its parents^4^,^5^,^6^. Furthermore, some *Citrus* species, including lime, typically reproduce asexually through nucellar embryony. Vegetative propagation by humans (e.g., grafting, budding, and layering) is another method for the asexual reproduction of *Citrus* species. Consequently, the heterozygous state of limes has been maintained over the course of their diversification. Therefore, it is important to identify variation in the heterozygosity of limes when studying their genetic diversity.

Recent advances in DNA sequencing have allowed the extensive use of short DNA fragments (single or multiple genes) to study the phylogenetic relationships of *Citrus* species. For example, by using these techniques, we have previously reported the phylogenetic relationships of *Citrus* and its relatives based on *rbcL* and *matK* gene sequences^7^,^8^. However, it is difficult to study intraspecies relationships from one or several DNA fragments. Restriction site-associated DNA sequencing (RAD-seq)^9^ may solve this problem. RAD tags are the DNA sequences that immediately flank a particular restriction site throughout the genome^10^. Recent developments in high-throughput sequencing have enabled us to read all RAD tags in parallel. In particular, RAD-seq has facilitated the rapid discovery of thousands of single-nucleotide variants (SNVs). In fact, this technique has facilitated the determination of relationships within species^11^. However, this method has not been applied to the study of *Citrus* species or other asexually reproducing organisms.

In this study, we morphologically characterized limes grown in Bhutan. Furthermore, we analyzed five accessions among these limes by RAD-seq. For comparison, we analyzed three lime accessions that were previously introduced to Japan from Indonesia. Thus, in the current study, we studied the relationships of limes grown in Bhutan and Indonesia and the history of their genetic diversification.

## Results

**Figure 1:**
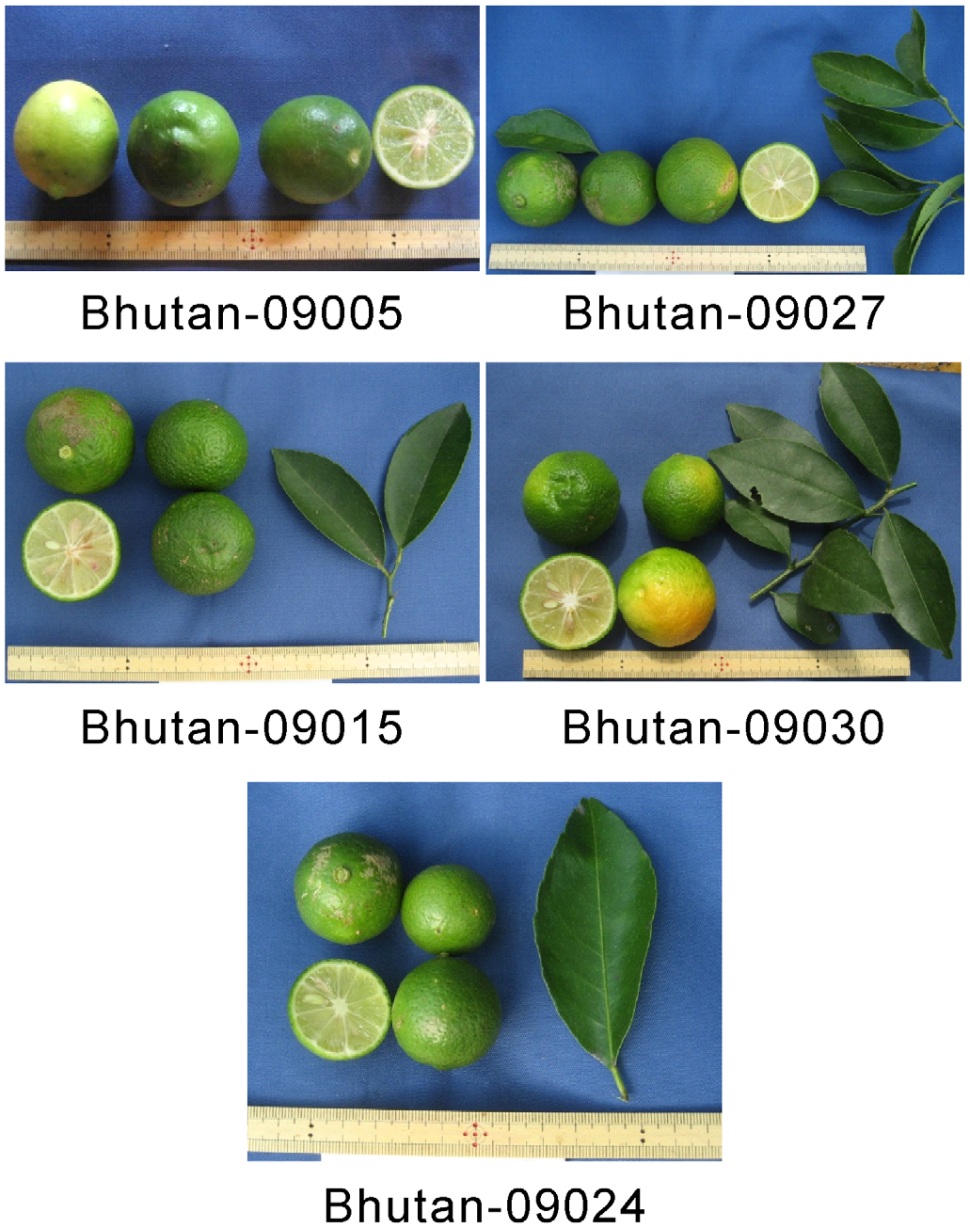
The fruits and leaves of the five Bhutanese lime accessions used in the RAD-seq analysis. Because we purchased the accession Bhutan-09005, a photograph of its leaves is not shown. The merchant confirmed the cultivation of this tree at Tsirang, southern Bhutan.

**Figure 2:**
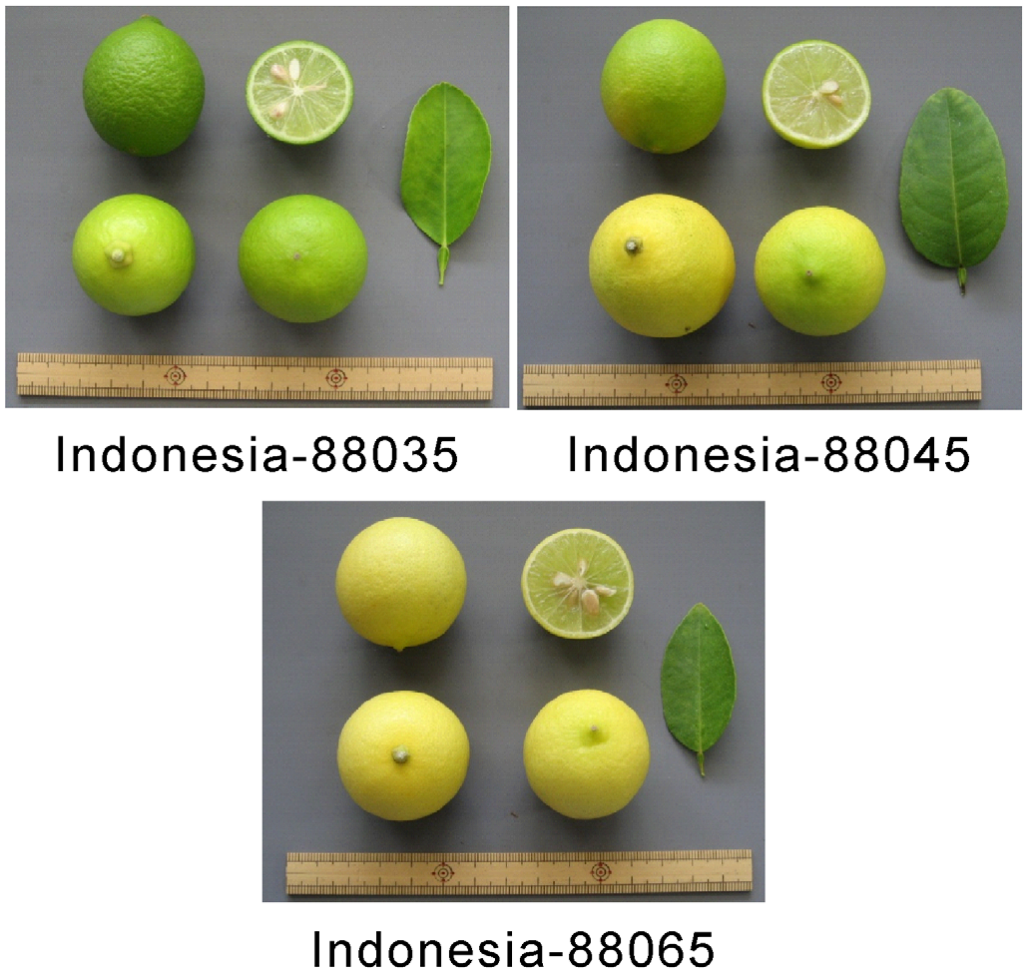
The fruits and leaves of the three Indonesian lime accessions used in the RAD-seq analysis.

Between 2009 and 2011, we identified several local lime accessions throughout Bhutan (Supplementary Table S1). Table 1 summarizes the leaf and fruit characteristics of these accessions. However, these data were influenced by environmental conditions because they were measured just after sampling in the field at each location or purchasing at the market. For instance, variation in skin and flesh color is influenced by the stage of maturation, as most limes turn yellow at maturation. However, these data were used to provide preliminary information about the characteristics of each accession. Associated photographs are presented in Figure 1. All accessions possessed the characteristic features of limes (e.g., a fruit rounder than a lemon, a highly acidic juice with a distinctive aroma, and a polyembryonic seed). Bhutanese accessions were separated into two classes based on their characteristics. The fruit surfaces of Bhutan-09003, Bhutan-09005, and Bhutan-11014 were smooth, whereas those of the other Bhutanese accessions were slightly smooth to coarse in texture. Furthermore, the wing of the leaf of Bhutan-11014 was wider than that of the other accessions. Bhutan-09005 and Bhutan-11014 are grown in the southern part of the country, whereas the other accessions from Bhutan are grown in almost all parts of the country. For comparative purposes, we morphologically characterized four lime accessions that were introduced to Saga University, Japan, in 1988 as budwoods from Indonesia (Table 1; Fig. 2). The four Indonesian accessions had relatively similar characteristics. Furthermore, the fruit surfaces of the Indonesian accessions were smooth, and the wing of the leaves was wider than that of other Bhutanese accessions, except for Bhutan-11014. Thus, these leaf characteristics are similar to those of Bhutan-11014, while the fruit characteristics are similar to those of Bhutan-09003, Bhutan-09005, and Bhutan-11014.

For further analysis, including RAD-seq analysis, we selected five Bhutanese limes (i.e., Bhutan-09005, Bhutan-09015, Bhutan-09024, Bhutan-09027, and Bhutan-09030) and three Indonesian limes (i.e., Indonesia-88035, Indonesia-88045, and Indonesia-88065) based on the preliminary fruit diversity characteristics. Both types of fruit surface were selected for the Bhutanese accessions, while three out of the four accessions were randomly selected for the Indonesian accessions. In addition, when we started the RAD-seq analysis, the only available Bhutanese accession carrying fruit with smooth surfaces was Bhutan-09005 (out of a possible Bhutan-09003, Bhutan-09005, and Bhutan-11014).

**Figure 3:**
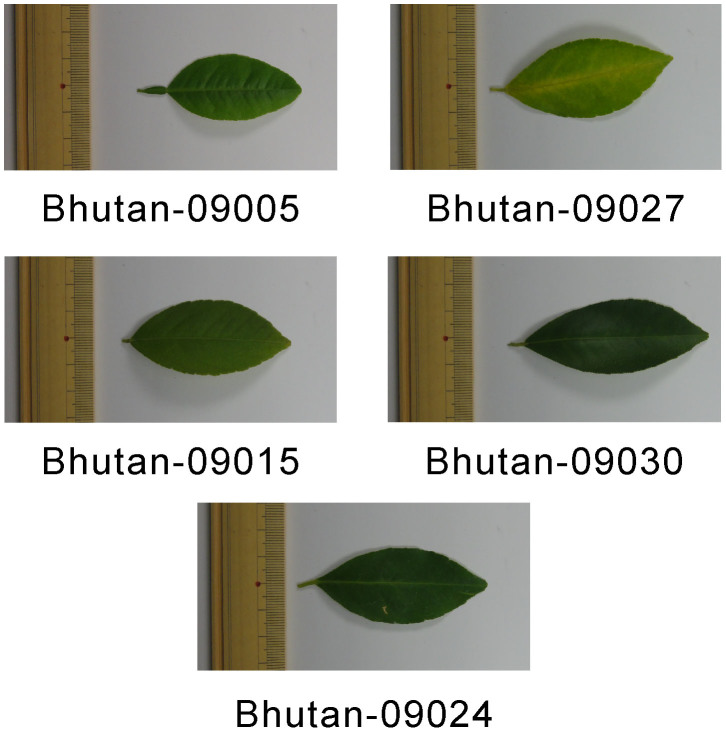
The leaves of the nucellar seedlings of the five Bhutanese lime accessions used in the RAD-seq analysis.

Next, to further elucidate the differences among the morphological traits of these eight accessions, we measured the leaf characteristics using trees grown under similar (greenhouse) conditions (Fig. 3, Table 2). Because the leaf characteristics of Bhutan-09005 were not available in the preliminary characterization, it is important to obtain a measurement. The area of the leaf blade ranged from 200.7π mm^2^ to 687.6π mm^2^ and from 313.3π mm^2^ to 651.8π mm^2^ in the Bhutanese and Indonesian accessions, respectively. The wing area ranged from 2.01π mm^2^ to 6.83π mm^2^ and from 8.48π mm^2^ to 17.70π mm^2^ in the Bhutanese and Indonesian accessions, respectively. The wing area of the Indonesian accessions was larger than that of the Bhutanese accessions. Bhutan-09005 had the largest wing of the Bhutanese accessions. The wing index of this accession was 3.33, which was the highest among eight accessions. The indices of the remaining Bhutanese accessions (Bhutan-09015, Bhutan-09024, Bhutan-09027, and Bhutan-09030) and three Indonesian accessions were 0.42–0.61 and 1.66–2.93, respectively. Based on the leaf traits, particularly wing size, Bhutan-09005 was clearly distinguished from the other Bhutanese accessions, and more closely resembled the Indonesian accessions. Thus, the quantitative characterization of leaf traits classified the eight accessions into two clusters as follows (p < 0.01): (1) the Bhutanese cluster containing Bhutan-09015, Bhutan-09024, Bhutan-09027, and Bhutan-09030 accessions with small wings and (2) the Indonesian cluster containing Bhutan-09005, Indonesia-88035, Indonesia-88045, and Indonesia-88065 accessions with large wings.

We sequenced the *Eco*RI RAD-tag from each of the eight accessions. After quality filtering the sequencing data, we obtained >24.5 million RAD-tag sequences (~1+ Gb sequences) from each sample (Table 3). We genotyped these eight accessions using the genome data of the sweet orange (327,944,670 bp)^12^ by analyzing the bowtie program (a program used to make short read alignments)^13^ and the pstacks program (a program used to build loci) of the Stacks pipeline^14^,^15^. Because the *de novo* RAD assembly of short reads overassembles some paralogous loci^16^, we only genotyped them in the presence of reference genome information. We showed the depth of RAD-seq coverage for each sample across the reference genome and the coverage histogram of each sample (Supplementary Fig. S1), and we calculated statistics from the alignment data created by the bowtie program (Supplementary Table S2). The RAD-seq results showed that more than sufficient amounts of sequencing data were obtained. Although the data from Indonesia-88045 contained a relatively higher amount of low-coverage reads (probably caused by the degradation of the genomic DNA), these low-coverage reads were removed by *in silico* analysis.

**Figure 4:**
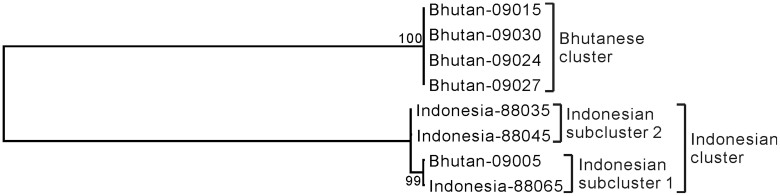
Maximum-likelihood tree based on the genotyped sites. Numbers at the nodes indicate bootstrap values (% over 1000 replicates).

To compare the genotypes of the eight accessions, we analyzed the aligned data using the Stacks pipeline as follows: cstacks (a program used to create a catalog of loci), sstacks (a program used to match samples with those in the catalog), and genotypes (a program used to export a Stacks data set as a set of observed haplotypes at each locus or with the haplotypes encoded into genotypes). These *in silico* steps integrated the data from the eight lime accession samples, corrected false-negative heterozygous genotypes and false-positive homozygous genotypes, extracted the sites useful for genotyping, and efficiently excluded certain errors, such as contamination caused by improper PCR amplification or degradation of the genomic DNA. However, it should be noted that it is difficult to completely remove all errors. The genotypes program compared 78,527 loci (3,219,607 bp, including the *Eco*RI sequence AATTC). The 42,613 sites that were potentially useful for genotyping (Supplementary Fig. S2) were extracted from the output of the genotypes program using the Perl script. Therefore, ~1.3% of sites from the analyzed regions were useful for genotyping. Among these 42,613 sites, 4846 were parsimony-informative. We constructed a phylogenetic tree (Fig. 4) using the maximum-likelihood method. This phylogenetic tree clearly showed that the eight accessions could be separated into two clusters, the Bhutanese and Indonesian clusters, as defined above. This classification was in accordance with the results of our morphological characterizations (Fig. 1, 2, and 3; Tables 1 and 2). Furthermore, phylogenetic analysis divided the Indonesian cluster into two subclusters, Indonesian subcluster 1, containing Bhutan-09005 and Indonesia-88065, and Indonesian subcluster 2, containing Indonesia-88035 and Indonesia-88045. Among the 42,613 sites, the Bhutanese cluster, Indonesian subcluster 1, and Indonesian subcluster 2 carried 41,691, 41,810, and 41,827 identical genotypes, respectively. In other words, the numbers of the genotypes that did not match within the Bhutanese cluster, Indonesian subcluster 1, and Indonesian subcluster 2 were 922, 803, and 786, respectively. Among the 42,613 sites, the Indonesian cluster carried 37,612 identical genotypes. In other words, the numbers of the genotypes that did not match within the Indonesian cluster was 5001.

Based on the results of the genotypes program, we estimated the minimum number of heterozygous genotypes for each accession (Table 3). The analysis showed that the frequencies of the heterozygous genotypes were at least 0.63–0.74%. Because of the hybrid origin of lime and the ability of limes to asexually reproduce, the heterozygosity of limes was maintained. The Bhutanese cluster contained more heterozygous genotypes than the Indonesian cluster. In the Indonesian cluster, Indonesian subcluster 1 contained more heterozygous genotypes than Indonesian subcluster 2.

We further classified the 42,613 sites into categories (i.e., Categories A–L) based on unique genotype combinations (Table 4; Supplementary Table S3). In Category A, we identified 7297/42,613 sites that contained unique heterozygous genotypes common to all eight accessions. In other words, M (A or C), R (A or G), W (A or T), S (C or G), Y (C or T), or K (G or T) were present in all eight accessions (Table 4; Supplementary Table S3).

In Category B, we identified 4026/42,613 sites that contained unique homozygous genotypes common to the Bhutanese cluster; other unique homozygous genotypes were common to the Indonesian cluster. For example, A and C were common to the Bhutanese and Indonesian clusters, respectively (Table 4; Supplementary Table S3). The classification of the eight accessions into two clusters may be primarily explained by the 4026 sites with unique homozygous genotypes.

In Category C, we identified 14,445/42,613 sites that contained unique heterozygous genotypes common to the Bhutanese cluster and unique homozygous genotypes that were common to the Indonesian cluster. For example, M and A were common to the Bhutanese and Indonesian clusters, respectively (Table 4; Supplementary Table S3). In comparison, we identified 10,912/42,613 sites in Category D that contained unique homozygous genotypes common to the Bhutanese cluster and unique heterozygous genotypes that were common to the Indonesian cluster. For example, A and M were common to the Bhutanese and Indonesian clusters, respectively (Table 4; Supplementary Table S3). The sum of the sites from these two categorical patterns was 25,357, which had the largest number of categorical patterns among the 42,613 sites. Several mechanisms may produce the differences between the two clusters. First, homologous recombination may explain the observed heterozygous genotypes changing into homozygous genotypes. Second, the observed heterozygous genotypes changing into homozygous genotypes may be explained by the loss of *Eco*RI sites, that is, the emergence of false-positive homozygous genotypes. Although we used the genotypes program to remove the false-positive genotypes, it may be difficult to remove them in all instances. Finally, the *de novo* mutations in the ancestor of each cluster may explain the observed changes of the homozygous genotypes into heterozygous genotypes.

In Category E, we identified 1432/42,613 sites that contained unique heterozygous genotypes common to the Bhutanese cluster; identical heterozygous genotypes and/or homozygous genotypes derived from the heterozygous genotypes were common to the Indonesian cluster. For example, M was common to the Bhutanese cluster, while M and A were present in the Indonesian cluster (Table 4; Supplementary Table S3; Supplementary Fig. S3). We counted genotype matches between each accession in the Indonesian cluster (Table 5). The analysis showed that, in many cases, the genotypes found in Indonesian subcluster 1 were identical to each other, with a similar result being obtained for Indonesian subcluster 2. This result may explain why the phylogenetic analysis classified the Indonesian cluster into two subclusters. As shown in Table 6, among the 1432 sites classified into Category E, 755 sites contained heterozygous genotypes that were common to Indonesian subcluster 1 and unique homozygous genotypes that were common to Indonesian subcluster 2. For example, M and A were common to the Indonesian subclusters 1 and 2, respectively. Moreover, among the 1432 sites, 271 were sites that contained unique homozygous genotypes that were common to Indonesian subcluster 1 and unique heterozygous genotypes that were common to the Indonesian subcluster 2. For example, A and M were common to Indonesian subclusters 1 and 2, respectively. Thus, the number of heterozygous genotypes in Indonesian subcluster 1 was approximately three-fold larger than that of Indonesian subcluster 2 in Category E. Because identical heterozygous genotypes are present in the Bhutanese cluster, these differences may be explained by the heterozygous genotypes changing into homozygous genotypes. Homologous recombination in the ancestors of the Indonesian cluster may, to some extent, explain the observed differences within the Indonesian cluster. Furthermore, these differences may, to some extent, be explained by the loss of the *Eco*RI sites (the emergence of false-positive homozygous genotypes).

In Category F, we identified 134/42,613 sites that contained unique heterozygous genotypes common to the Indonesian cluster; identical heterozygous genotypes and/or homozygous genotypes derived from the heterozygous genotypes were common to the Bhutanese cluster. For example, M and A were present in the Bhutanese cluster, while M was common to the Indonesian cluster (Table 4; Supplementary Table S3; Supplementary Fig. S4). Genotype matches between each accession in the Bhutanese cluster (Supplementary Table S4) had a narrow range (50–78 matches; 1.6-fold differences). This phenomenon may explain why the phylogenetic analysis did not classify the Bhutanese cluster into two subclusters. Because identical heterozygous genotypes are present in the Indonesian cluster, these differences may be explained by the heterozygous genotypes changing into homozygous genotypes. Like in Category E, both the homologous recombination in the ancestors of the Bhutanese cluster and the loss of *Eco*RI sites may explain the observed differences within the Bhutanese cluster.

In Category G, we identified 3087/42,613 sites that contained unique homozygous genotypes common to the Bhutanese cluster; unique heterozygous and homozygous genotypes derived from the heterozygous genotypes were present in the Indonesian cluster. For example, A was common to the Bhutanese cluster, while M and A were present in the Indonesian cluster (Table 4; Supplementary Table S3; Supplementary Fig. 5). We counted genotype matches between each accession belonging to the Indonesian cluster (Table 7). The analysis showed that, in many cases, genotypes found in Indonesian subcluster 1 were identical to each other, with the same result being obtained for Indonesian subcluster 2. This is the reason why the phylogenetic analysis classified the Indonesian cluster into two subclusters. As shown in Table 8, among the 3087 sites in Category G, 1573 sites contained heterozygous genotypes that were common to Indonesian subcluster 1 and unique homozygous genotypes that were common to Indonesian subcluster 2. Moreover, among the 3087 sites, 613 sites contained homozygous genotypes that were common to Indonesian subcluster 1 and unique heterozygous genotypes that were common to Indonesian subcluster 2. The number of heterozygous genotypes in Indonesian subcluster 1 was approximately three-fold larger than that of Indonesian subcluster 2 in Category G. When the Bhutanese cluster carried unique homozygous genotypes (e.g., A genotypes), changes from unique heterozygous genotypes (e.g., M genotypes) to homozygous genotypes identical to the Bhutanese cluster (e.g., A genotypes) occurred more frequently compared to those from heterozygous genotypes (e.g., M) to other homozygous genotypes (e.g., C genotypes) in the Indonesian cluster (2439 events vs. 609 events; estimated from Supplementary Table S3). Thus, the two types of changes did not arise equally (4-fold differences). Probably, the differences within the Indonesian cluster are not explained only by heterozygous genotypes changing into homozygous genotypes. Both heterozygous changes into homozygous genotypes and homozygous changes into heterozygous genotypes potentially contribute these differences. The former change may arise through homologous recombination and the loss of *Eco*RI sites, while the latter change may arise through *de novo* mutation.

In Category H, we identified 597/42,613 sites that contained unique homozygous genotypes common to the Indonesian cluster; unique heterozygous and homozygous genotypes derived from the heterozygous genotypes were present in the Bhutanese cluster. For example, M and A were present in the Bhutanese cluster, while A was common to the Indonesian cluster (Table 4; Supplementary Table S3; Supplementary Fig. S6). Genotype matches between each accession belonging to the Bhutanese cluster (Supplementary Table S5) had a narrow range (213–342 matches; 1.6-fold differences). This phenomenon is the reason why the phylogenetic analysis did not classify the Bhutanese cluster into two subclusters. When the Indonesian cluster carried the unique homozygous genotypes (e.g., A), the number of changes from the unique heterozygous genotypes (e.g., M) to the homozygous genotypes in the Bhutanese cluster that were identical to the Indonesian cluster (e.g., A) was 385. In contrast, the number of changes from the heterozygous genotypes (e.g., M) to the other homozygous genotypes (e.g., C) was 204. Thus, fewer differences in the occurrence of the two types of changes were observed (about 2-fold difference) when compared to Category G. However, like Category G, both changes from heterozygous to homozygous genotypes and changes from homozygous to heterozygous genotypes might contribute to these differences.

We were able to roughly estimate the contributions of the loss of *Eco*RI sites (the emergence of false-positive homozygous genotypes) to genetic diversification within the cluster. For example, we considered the differences within the Indonesian cluster, in which more frequent changes occurred (Category E and G). Some of the changes in Category G may be explained by *de novo* mutation. These *de novo* mutations occurred in the 36 bp sequences adjacent to the *Eco*RI site. If we assume that the 3087 events observed in Category G were caused by *de novo* mutation only, the possible number of *de novo* mutations of the *EcoRI* sites (6 bp sequence) is 515 ( = 3087/6). We observed 1432 events in Category E, which was about 3-fold larger than the hypothesized number. Thus, both homologous recombination and the loss of *Eco*RI sites may contribute the changes from heterozygous to homozygous genotypes.

In Category I, we identified 242/42,613 sites that contained heterozygous and homozygous genotypes that were distributed randomly among the eight accessions (Table 4; Supplementary Table S3: Supplementary Fig. 7). Some experimental or computational errors may have led to the identification of these sites. Genotype matches between each accession (Supplementary Table S6) had a relatively wide range (53–179 matches; 3.4-fold differences), but it was difficult to extract any clear trends from this analysis.

In Category J, we identified 242/42,613 sites that contained unique homozygous genotypes common to the Bhutanese cluster, and identical homozygous genotypes and other homozygous genotypes that were present in the Indonesian cluster (Table 4; Supplementary Table 3, Supplementary Fig. 8). We counted genotype matches between each accession belonging to the Indonesian cluster (Table 9). In most cases, genotypes found in Indonesian subcluster 1 were identical to each other, with the same result being obtained for Indonesian subcluster 2, which contributed to the classification of the two Indonesian subclusters.

In Category K, we identified 2/42,613 sites that contained two types of homozygous genotypes present in the Bhutanese cluster and unique homozygous genotypes that were common to the Indonesian cluster (Table 4; Supplementary Table S3; Supplementary Fig. S9). In Category L, we identified 197/42,613 sites that contained different types of heterozygous genotypes that were distributed among the eight accessions (Table 4; Supplementary Table S3; Supplementary Fig. S10). It was difficult to obtain useful information from these data.

## Discussion

Our analysis showed that none of the lime accessions assessed in the current study were fully homozygous, which may be explained by the hybrid origin of this species, as previously proposed^4^,^5^,^6^. As shown in Table 1, the seeds of these accessions are polyembryonic, which enables limes to maintain a heterozygous state by reproducing asexually. Thus, these observed heterozygosities support the proposed hybrid origin of limes and the ability of limes to reproduce asexually.

The genotypes shown in Category C and D mainly contributed the differences between the Bhutanese and Indonesian clusters (14,445 and 10,912 events, respectively). One cluster carried heterozygous genotypes, while the other cluster carried homozygous genotypes. These differences may arise through several mechanisms, including homologous recombination, the loss of *Eco*RI sites (the emergence of false-positive homozygous genotypes), and *de novo* mutations in the ancestor of each cluster. Sexual reproduction might contribute to homologous recombination in limes, which is one of the proposed mechanisms. Indeed, the lime has self-pollinating properties^17^. A previous study showed that both sexual and asexual reproduction in limes cultivated in Tunisia contributed to their genetic diversity^18^. However, further study is required to elucidate how sexual reproduction contributes to the genetic diversification of limes.

Our analyses separated the eight selected accessions into two clusters. In addition, the Indonesian cluster was further grouped into two subclusters. Thus, our analysis suggests that the Bhutanese cluster, and the Indonesian subclusters 1 and 2 may be independently selected. Because humans cultivate these eight accessions, humans may have played a role in these selection events. The ancestors of each cluster or subcluster may possess features that were beneficial for humans, which were subsequently selected for in subsequent generations. Alternatively, if humans did not select the ancestors of each cluster or subcluster, the ancestors may have acquired advantageous characteristics via natural selection.

Unexpectedly, the Indonesian cluster was distributed throughout both Indonesia and Bhutan. However, the origins of the Indonesian cluster are not clear. Bhutan-09005 is cultivated at Tsirang in southern Bhutan. This district is close to India, with the two countries having an active trading history. Therefore, the ancestor(s) of this tree may have had more opportunities to become distributed throughout various regions, unlike the four accessions of the Bhutanese cluster.

We showed that there were minimal differences among the genotypes within the Bhutanese cluster and within each Indonesian subcluster. Because 20,183 to 23,985 heterozygous genotypes were estimated from 42,613 sites (Table 3), crosses within the Bhutanese cluster or within each Indonesian subcluster are expected to result in the production of numerous genetic differences. Self-pollination would also result in the production of numerous genetic differences. It is possible that each cluster/subcluster was derived from a single tree by vegetative propagation. However, differences among the genotypes within the Bhutanese cluster or within each Indonesian subcluster remain, although some of these differences may have been generated from experimental or computational errors. During asexual reproduction, changes within a cluster or subcluster may have occurred. Some of the differences among the genotypes within the Bhutanese cluster or the two Indonesian subclusters (many events observed in Category E and F, and some events observed in Category G and H) may be explained by homologous recombination. Therefore, these observations strongly suggest that somatic homologous recombination, possibly by the mechanism of double strand break repair, may have contributed to the genetic diversification of limes. Furthermore, as observed in Category G and H, *de novo* mutation may have also contributed to this phenomenon.

Furthermore, small differences were obtained among the genotypes within the Indonesian cluster, namely those between each subcluster. Simple crosses within one subcluster are expected to result in the production of numerous genetic differences. The possibility of producing other types of subclusters by crossing is very low. Some differences among the genotypes between each subcluster (many events observed in Category E, and some events observed in Category G) may be explained by homologous recombination. Therefore, these observations suggest that somatic homologous recombination at larger regions may also contribute to genetic diversification within the Indonesian cluster. Furthermore, as observed in Category G, *de novo* mutation may have also contributed to this phenomenon.

Although we revealed the reproductive history of limes at the genomic level, the current study only analyzed a small number of accessions from just two countries. Therefore, larger sample numbers are required to elucidate the full evolutionary history of limes. Our analysis did not provide any information about the parental generation of the eight accessions. Analysis of other related *Citrus* species is required to address this question.

In conclusion, using high-throughput sequencing, we partially analyzed intraspecies relationships between limes. To the best of our knowledge, this is the first report about the characteristics of *Citrus* species using RAD-seq. Our analysis suggests part of the evolutionary history of limes and may link it to the past activities of humans.

## Methods

### Plant materials

Preliminary characterization was conducted by investigating 14 local lime accessions in Bhutan between 2009 and 2011 (as part of a collaborative study on exploration and identification of wild citrus relatives in Bhutan). Leaf and fruit characteristics were measured just after sampling in the field at each location or purchasing at the market. The characteristics of four lime accessions, which were introduced as budwoods to Saga University, Japan, from Indonesia, were also measured. We classified the accessions based on the characteristics of the fruit surfaces and the wing of the leaves, because these characteristics are minimally influenced by environmental or growth conditions.

Quantitative characterization of the leaf wings was conducted. Specifically, the seeds were extracted from the fruit of five Bhutanese accessions that were collected (i.e., Bhutan-09005, Bhutan-09015, Bhutan-09024, Bhutan-09027, and Bhutan-09030). The seedlings derived from these seeds were grown in a greenhouse. Nucellar seedlings were selected based on leaf morphology, because nucellar and zygotic seedlings are usually differentiated by leaf shape^19^. Because this selection method does not always work^20^, we used genomic data to confirm that we selected asexually reproducing plants. All nucellar seedlings were grown at the Renewable Natural Resources Research Centre Wengkhar, Mongar, Bhutan. The leaf shape of the five Bhutanese accessions and three Indonesian accessions (i.e., Indonesia-88035, Indonesia-88045, and Indonesia-88065) was investigated. Five leaves from one tree were used as the material for each accession. The length and width of the leaf blade and wing were measured. Leaf blade area and wing area were estimated using the formula for calculating the ellipse area: area = width/2 × length/2 × π. The wing index was expressed as: (area of the wing) × 100/(area of the leaf blade). For subsequent DNA analysis, the leaves collected from the five Bhutanese and three Indonesian accessions were used as the material.

### RAD-seq analysis

Genomic DNA was extracted from the leaves using the DNeasy Plant Mini Kit (Qiagen, Valencia, CA, USA), with some modifications (i.e., the addition of 8 μl β-mercaptoethanol and 4 mg polyvinylpolypyrrolidone to 400 μl Buffer AP1). The quality of the isolated genomic DNA was checked by 1% agarose gel electrophoresis. *EcoRI* RAD-seq analysis was commissioned to BGI (Shenzhen, China) where the data were generated using HiSeq 2000. At BGI, the raw data were modified using the following two steps: (1) the reads that were polluted by adapter sequences were deleted and (2) the reads that contained >50% low-quality bases (quality value ≤ 5) or >10% N bases were removed. Each read received from BGI was a 41-bp single-end sequence [AATTC (*Eco*RI site) + 36 bp sequence]. Sequences are available at the DDBJ Sequence Read Archive (http://trace.ddbj.nig.ac.jp/dra/index_e.shtml; Accession no. DRA000989).

### Sequence analysis

The data from BGI were analyzed using the Stacks software pipeline (version 1.09)^14^,^15^. The data were further quality-filtered by a process_shortreads program of Stacks (with the -c -q options). The reference genome data of the sweet orange (*Citrus sinensis*) were then downloaded from http://citrus.hzau.edu.cn/orange/download/csi.chromosome.fa.tar.gz12. The data were aligned with the reference genome by using the short read alignment program bowtie (version 1.0.0) with the -n 3 -k 10 --best --chunkmbs 1024 options^13^. The pstacks program of the Stacks pipeline extract stacks that have been aligned to a reference genome by the bowtie program and identify SNVs at each locus using a maximum likelihood framework. The pstacks program was used to analyze the aligned data with the -m 5 option (minimum depth of coverage required to report a stack is 5). The cstacks program of the Stacks pipeline builds a catalog from a set of samples processed by the pstacks program, and creates a set of consensus loci by merging the genotypes together. By the cstacks program, the data obtained from the pstacks program were analyzed with the -n 1 option (the number of mismatches allowed between sample tags when generating the catalog is 1). In the sstacks program of the Stacks pipeline, sets of stacks constructed by the pstacks program are searched against a catalog produced by the cstacks program. By the sstacks program, the data from pstacks and cstacks were analyzed with no options being selected. The genotypes program of the Stacks pipeline produces output tables of the genotypes. By the genotypes program, the data obtained from the pipeline were analyzed with -c options (with this option, the program corrected false-negative heterozygous genotypes and false-positive homozygous genotypes), the -r 8 option (the minimum number of progeny required to print a marker is 8), and the -m 5 option (the minimum stack depth required before exporting a locus in a particular individual is 5). A multiple alignment file was created by extracting the output of the genotypes program with the Perl script “MultiSNPs2.pl,” which is available from http://ecol.zool.kyoto-u.ac.jp/~tetsumi/image/script/MultiSNPs2.pl.zip.

Phylogenetic trees were constructed using the maximum-likelihood method of the MEGA program (version 5.2.2)^21^ with the following options: (1) test of Phylogeny: bootstrap method; (2) number of bootstrap replications: 1000; (3) model/method: Tamura-Nei model; (4) rate among sites: uniform rates; (5) gaps/missing data treatment: complete deletion; (6) ML heuristic method: nearest-neighbor-interchange; (7) initial tree for ML: make initial tree automatically (default – NJ/BioNJ); and (8) branch swap filter: very strong.

To analyze the sites that are useful for genotyping, a multiple alignment file was modified to construct a tab-delimited table file by combining the editor program and the “sed” command of Linux, which were then transposed by the “t” function of the programming language R. After removing the tab, this transposed file was analyzed by the “grep”, “egrep” and “wc” commands of Linux.

To calculate the depth of coverage, coverage histogram, and coverage statistics, the bed files showing the mapped regions were created using the bamToBed program of bedtools (version 2.17.0)^22^ by analyzing the bam file, which was obtained using the bowtie program. The bam and bed files were then analyzed using the Qualmap program (version 0.7.1)^23^.

## Supplementary Material

Supplementary Information Supplementary Information

## Figures and Tables

**Table 1:** Leaf and fruit characteristics of the Bhutanese and Indonesian lime accessions

			Leaf								Fruit								Seed	
Accession		Leaf blade		Wing	Skin color	Fruit surface	Flesh color	Puffing	Granulation	Peeling	Bitterness	Sweetness	Sourness	Diameter (mm)	Height (mm)	D/H index	pH of juice	Embryo color	Polyembryony	Number
	Length (mm)	Width (mm)	Length (mm)	Width (mm)																
Bhutan-09003	-	-	-	-	Yellowish green	Smooth	Green	None	None	Difficult	-	-	High	36.6	38.5	94.8	-	-	-	1.4
Bhutan-09005	-	-	-	-	Yellowish green	Smooth	Green	None	None	Slightly difficult	None	Low	High	34.3	38.5	89.1	-	Cream, Pale green	Poly	5.2
Bhutan-09015	65.1	30.0	5.6	1.5	Green	Slightly coarse	Cream	None	None	Difficult	None	Low	High	40.4	39.2	103.2	2.4	Pale green, Green	Poly	4.6
Bhutan-09018	84.7	38.3	8.5	1.7	Green	Slightly smooth	Green	None	None	Difficult	None	Low	Medium	34.7	35.9	96.7	2.5	Pale Green	Poly	0.3
Bhutan-09019	91.0	45.2	6.3	1.6	Yellowish green	Medium	Yellow	None	None	Difficult	None	Low	Medium	42.7	44.1	96.8	2.5	Pale Green	Poly	1.0
Bhutan-09024	85.4	37.6	5.0	1.6	Green	Slightly smooth	Green	None	None	Difficult	None	Low	Medium	30.4	29.8	102.0	2.4	Pale Green	Poly	4.8
Bhutan-09027	64.3	30.1	4.0	1.5	Green	Slightly coarse	Green	None	None	Difficult	None	Low	High	39.6	40.5	97.8	2.5	Cream, Pale green	Poly	6.4
Bhutan-09030	78.2	34.4	4.6	1.7	Yellowish green	Slightly coarse	Cream	None	None	Difficult	None	Low	High	45.2	46.6	97.0	2.5	Pale green, Green	Poly	15.3
Bhutan-11006	75.7	35.1	4.4	2.0	Yellow	Medium	Yellow	None	None	Difficult	None	Low	High	36.6	37.6	97.3	2.2	Cream, Pale green	Poly	12.0
Bhutan-11009	71.2	33.5	3.7	1.6	Yellow	Slightly smooth	Yellow	None	None	Difficult	None	Low	High	42.2	44.0	95.9	2.3	Cream	Poly	11.4
Bhutan-11014	64.1	39.6	8.8	5.5	Yellow	Smooth	Cream	None	None	Difficult	None	Low	High	39.7	44.9	88.4	2.1	Cream	Poly	6.8
Bhutan-11018	80.6	39.9	4.2	1.8	Green	Slightly coarse	Green	None	None	Difficult	-	-	-	23.7	24.3	97.5	-	-	-	-
Bhutan-11026	100.1	49.0	4.1	2.4	Green	Coarse	Cream	-	-	-	None	Low	High	54.6	52.6	103.8	2.5	Cream	Poly	11.5
Bhutan-11030	98.9	47.0	4.3	2.1	Green	Coarse	Cream	-	-	-	None	Low	High	39.1	39.3	99.5	2.3	Cream	Poly	4.4
Indonesia-88035	53.6	32.6	8.5	3.6	Yellowish green	Smooth	Yellowish green	None	None	Difficult	None	Low	High	33.7	38.0	88.7	2.2	Cream, Pale green	Poly	7.2
Indonesia-88045	65.2	36.3	6.6	3.1	Yellow	Smooth	Yellow	None	None	Difficult	None	Low	High	39.4	45.0	87.6	2.1	Cream	Poly	8.5
Indonesia-88065	49.1	26.0	5.1	2.6	Yellow	Smooth	Yellow	None	None	Difficult	None	Low	High	37.7	39.5	95.4	2.1	Cream	Poly	11.0
Indonesia-88076	52.0	34.6	5.3	3.1	Yellow	Smooth	Yellow	None	None	Difficult	None	Low	High	37.4	36.6	102.2	2.1	Cream	Poly	12.4

**Table 2:** Leaf characteristics of the nucellar seedlings of the five Bhutanese and three Indonesian lime accessions

		Leaf blade			Wing		
Accession	length (mm)	width (mm)	Area^z^ (mm^2^)	length (mm)	width (mm)	Area^z^ (mm^2^)	Wing index^y^
Bhutan-09015	51.1 ± 2.3 b^x^	26.2 ± 0.8 ab	335.7π ± 24.0π ab	5.7 ± 0.5 a	1.4 ± 0.1 a	2.01π ± 0.15π a	0.60 ± 0.03 a
Bhutan-09024	66.1 ± 1.7 cd	31.5 ± 1.1 bc	522.1π ± 26.6π bc	6.4 ± 1.0 a	1.9 ± 0.1 ab	3.09π ± 0.62π ab	0.59 ± 0.11 a
Bhutan-09027	62.1 ± 2.30 bc	32.2 ± 0.5 bc	501.5π ± 22.3π bc	6.7 ± 4.1 ab	1.8 ± 0.1 ab	3.11π ± 0.53π ab	0.61 ± 0.09 a
Bhutan-09030	76.2 ± 5.0 d	35.6 ± 1.9 bc	687.6π ± 81.8π c	5.8 ± 0.4 a	1.9 ± 0.1 ab	2.84π ± 0.33π ab	0.42 ± 0.05 a
Bhutan-09005	36.7 ± 1.3 a	22.4 ± 1.1 a	207.0π ± 15.3π a	9.2 ± 0.7 abc	2.9 ± 0.4 bc	6.83π ± 1.46π bc	3.33 ± 0.73 c
Indonesia-88035	64.6 ± 1.9 cd	37.4 ± 6.3 cd	605.3π ± 34.2π c	13.7 ± 0.5 d	5.1 ± 0.5 d	17.70π ± 1.47π d	2.93 ± 030 b
Indonesia-88045	64.5 ± 2.2 cd	40.1 ± 2.5 d	651.8π ± 34.2π c	11.8 ± 1.5 cd	3.7 ± 0.2 c	10.76π ± 1.22π c	1.66 ± 0.13 ab
Indonesia-88065	47.5 ± 2.0 ab	26.3 ± 0.9 ab	313.3π ± 20.7π ab	11.0 ± 1.5 bcd	3.1 ± 0.1 c	8.48π ± 1.33π c	2.72 ± 0.85 b

^z^Leaf blade and wing area were estimated using the formula for calculating the ellipse area.

^y^(Area of wing) × 100/Area of leaf blade.

^x^Different letters within each column indicate significant differences of 5% via Tukey's test.

**Table 3:** Summary of the RAD-seq analysis

Accession	Number of sequencing reads after quality filtering	Minimum number of heterozygous genotypes estimated from 42,613 sites	Minimum frequency of heterozygous genotypes estimated from 42,613 sites
Bhutan-09015	78,420,728	23,985	0.74%
Bhutan-09024	67,963,194	23,902	0.74%
Bhutan-09027	36,677,458	23,915	0.74%
Bhutan-09030	45,902,996	23,960	0.74%
Bhutan-09005	36,682,650	21,822	0.68%
Indonesia-88035	24,485,978	20,184	0.63%
Indonesia-88045	48,889,661	20,183	0.63%
Indonesia-88065	28,103,773	21,751	0.68%

**Table 4:** Summary of the analysis of the 42,613 sites genotyped in the current study

	Number of sites
Category A.Unique heterozygous genotypes are common to all eight accessions.	7,297
Category B.Unique homozygous genotypes are common to the Bhutanese cluster.Other unique homozygous genotypes are common to the Indonesian cluster.	4,026
Category C.Unique heterozygous genotypes are common to the Bhutanese cluster.Unique homozygous genotypes are common to the Indonesian cluster.	14,445
Category D.Unique homozygous genotypes are common to the Bhutanese cluster.Unique heterozygous genotypes are common to the Indonesian cluster.	10,912
Category E.Unique heterozygous genotypes are common to the Bhutanese cluster.Identical heterozygous genotypes and/or homozygous genotypes derived from the heterozygous genotypes are present in the Indonesian cluster.	1,432
Category F.Unique heterozygous genotypes are common to the Indonesian cluster.Identical heterozygous genotypes and/or homozygous genotypes derived from the heterozygous genotypes are present in the Bhutanese cluster.	134
Category G.Unique homozygous genotypes are common to the Bhutanese cluster.Unique heterozygous genotypes and homozygous genotypes derived from the heterozygous genotypes are present in the Indonesian cluster.	3,087
Category H.Unique homozygous genotypes are common to the Indonesian cluster.Unique heterozygous genotypes and homozygous genotypes derived from the heterozygous genotypes are present in the Bhutanese cluster.	597
Category I.Heterozygous and homozygous genotypes are distributed among eight accessions.	242
Category J.Unique homozygous genotypes are common to the Bhutanese cluster.Identical homozygous genotypes and other homozygous genotypes are present in the Indonesian cluster.	242
Category K.Two kinds of homozygous genotypes are present in the Bhutanese cluster.Unique homozygous genotypes are common to the Indonesian cluster.	2
Category L.Different kinds of heterozygous genotypes are distributed among eight accessions.	197
Total number of genotypes.	42,613

**Table 5:** Number of genotype matches between each accession of the Indonesian cluster in Category E

	Bhutan-09005	Indonesia-88035	Indonesia-88045
Indonesia-88035	163		
Indonesia-88045	144	1215	
Indonesia-88065	1270	180	194

**Table 6:** Comparison of genotypes among the Bhutanese cluster and the two Indonesian subclusters in Category E

Bhutanese cluster	Indonesian subcluster 1	Indonesian subcluster 2	Number of the groupings
M	M	A	26
M	M	C	34
R	R	A	120
R	R	G	110
W	W	A	45
W	W	T	57
S	S	C	37
S	S	G	29
Y	Y	C	100
Y	Y	T	105
K	K	G	44
K	K	T	48
		Total	755
M	A	M	16
M	C	M	13
R	A	R	36
R	G	R	33
W	A	W	15
W	T	W	18
S	C	S	20
S	G	S	11
Y	C	Y	34
Y	T	Y	46
K	G	K	17
K	T	K	12
		Total	271

**Table 7:** Number genotype matches between each accession of the Indonesian cluster in Category G

	Bhutan-09005	Indonesia-88035	Indonesia-88045
Indonesia-88035	437		
Indonesia-88045	313	2595	
Indonesia-88065	2575	508	464

**Table 8:** Comparison of the genotypes among the Bhutanese cluster and the two Indonesian subclusters in Category G

Bhutanese cluster	Indonesian subcluster 1	Indonesian subcluster 2	Number of the groupings
A	M	A	80
A	M	C	9
C	M	A	11
C	M	C	70
A	R	A	185
A	R	G	35
G	R	A	33
G	R	G	217
A	W	A	94
A	W	T	17
T	W	A	12
T	W	T	96
C	S	C	49
C	S	G	4
G	S	C	7
G	S	G	39
C	Y	C	195
C	Y	T	29
T	Y	C	35
T	Y	T	192
G	K	G	74
G	K	T	12
T	K	G	12
T	K	T	66
		Total	1573
A	A	M	20
A	C	M	5
C	A	M	5
C	C	M	24
A	A	R	62
A	G	R	13
G	A	R	15
G	G	R	92
A	A	W	27
A	T	W	7
T	A	W	7
T	T	W	37
C	C	S	17
C	G	S	4
G	C	S	7
G	G	S	19
C	C	Y	64
C	T	Y	19
T	C	Y	19
T	T	Y	79
G	G	K	33
G	T	K	0
T	G	K	4
T	T	K	34
		Total	613

**Table 9:** Number of genotype matches between each accession of the Indonesian cluster in Category J

	Bhutan-09005	Indonesia-88035	Indonesia-88045
Indonesia-88035	16		
Indonesia-88045	10	229	
Indonesia-88065	225	13	7
