# Supplementary material for: Characterization of limes (Citrus aurantifolia) grown in Bhutan and Indonesia using high-throughput sequencing
Source: Sci Rep. 2014 Apr 30;4:4853. doi: 10.1038/srep04853 (PMC5381282; doi:10.1038/srep04853)
Supplement: Supplementary Information [file srep04853-s1.pdf]

Supplementary Information

Characterization of limes (*Citrus aurantifolia*) grown in Bhutan and Indonesia using high-throughput sequencing

Tshering Penjor, Takashi Mimura, Ryoji Matsumoto, Masashi Yamamoto, and Yukio Nagano

Supplementary Figure Legends

Supplementary Figure S1. The depth of RAD-seq coverage for each sample across a reference genome and the coverage histogram for each sample.

Supplementary Figure S2. Sites useful for genotyping among the eight accessions (clustal format).

Supplementary Figure S3. Sites useful for genotyping among the eight accessions (clustal format) in Category E.

Supplementary Figure S4. Sites useful for genotyping among the eight accessions (clustal format) in Category F.

Supplementary Figure S5. Sites useful for genotyping among the eight accessions (clustal format) in Category G.

Supplementary Figure S6. Sites useful for genotyping among the eight accessions (clustal format) in Category H.

Supplementary Figure S7. Sites useful for genotyping among the eight accessions (clustal format) in Category I.

Supplementary Figure S8. Sites useful for genotyping among the eight accessions (clustal

format) in Category J.

Supplementary Figure S9. Sites useful for genotyping among the eight accessions (clustal format) in Category K.

Supplementary Figure S10. Sites useful for genotyping among the eight accessions (clustal format) in Category L.

Supplementary Table S1. List of investigated local lime genetic resources in Bhutan

Accession	Place		Altitude	Latitude	Longitude
	Administrative area (Zhonqkhaq)	Region			
Bhutan-09003	Tsirang	Southwest	-	-	-
Bhutan-09005	Tsirang	Southwest	-	-	-
Bhutan-09015	Mongar	East	1276 m	N 27.17°	E 91.13°
Bhutan-09018	Mongar	East	954 m	N 27.16°	E 91.12°
Bhutan-09019	Mongar	East	725 m	N 27.16°	E 91.08°
Bhutan-09024	Trashigang	East	1350 m	N 27.17°	E 91.32°
Bhutan-09027	Trashigang	East	1588 m	N 27.16°	E 91.32°
Bhutan-09030	Mongar	East	999 m	N 27.16°	E 91.22°
Bhutan-11006	Mongar	East	1366 m	N 27.15°	E 91.23°
Bhutan-11009	Samdrup Jongkhar	Southeast	516 m	N 26.52°	E 91.34°
Bhutan-11014	Samdrup Jongkhar	Southeast	260 m	N 26.51°	E 91.42°
Bhutan-11018	Pemagatsel	Southeast	968 m	N 27.00°	E 91.24°
Bhutan-11026	Trongsa	Midland	1068 m	N 27.21°	E 90.33°
Bhutan-11030	Tsirang	Southwest	1711 m	N 26.58°	E 90.79°

Bhutan-09003 and Bhutan-09005 were purchased in the market.

Supplementary Table S2. Statistics of the alignment data created by bowtie program.

	Mean coverage inside of mapped regions	Standard deviations	Size of mapped regions
Bhutan-09015	654.4	4189	12.78Mb
Bhutan-09024	560.2	3308	13.27Mb
Bhutan-09027	301.0	1573	12.66Mb
Bhutan-09030	375.2	2229	12.69Mb
Bhutan-09005	298.5	1563	12.23Mb
Indonesia-88035	211.8	1095	11.90Mb
Indonesia-88045	149.6	1418	35.34Mb
Indonesia-88065	255.7	1404	11.30Mb

Supplementary Table S3. Detailed summary of the analysis of the 42,613 sites genotyped in the current study

	Number of sites
Category A.<br>Unique heterozygous genotypes are common to all eight accessions.	7,297
M (A or C) are common to all 8 accessions.	691
R (A or G) are common to all 8 accessions.	2,118
W (A or T) are common to all 8 accessions.	932
S (C or G) are common to all 8 accessions.	544
Y (C or T) are common to all 8 accessions.	2,237
K (G or T) are common to all 8 accessions.	775
Category B.<br>Unique homozygous genotypes are common to the Bhutanese cluster.<br>Other unique homozygous genotypes are common to the Indonesian cluster.	4,026
A are common to Bhutanese cluster, and C are common to Indonesian cluster.	222
A are common to Bhutanese cluster, and G are common to Indonesian cluster.	622
A are common to Bhutanese cluster, and T are common to Indonesian cluster.	276
C are common to Bhutanese cluster, and A are common to Indonesian cluster.	202
C are common to Bhutanese cluster, and G are common to Indonesian cluster.	155
C are common to Bhutanese cluster, and T are common to Indonesian cluster.	585
G are common to Bhutanese cluster, and A are common to Indonesian cluster.	563
G are common to Bhutanese cluster, and C are common to Indonesian cluster.	137
G are common to Bhutanese cluster, and T are common to Indonesian cluster.	200
T are common to Bhutanese cluster, and A are common to Indonesian cluster.	255
T are common to Bhutanese cluster, and C are common to Indonesian cluster.	583
T are common to Bhutanese cluster, and G are common to Indonesian cluster.	226
Category C.<br>Unique heterozygous genotypes are common to the Bhutanese cluster.<br>Unique homozygous genotypes are common to the Indonesian cluster.	14,445
M are common to Bhutanese cluster, and A are common to Indonesian cluster.	668
M are common to Bhutanese cluster, and C are common to Indonesian cluster.	705
R are common to Bhutanese cluster, and A are common to Indonesian cluster.	2,166
R are common to Bhutanese cluster, and G are common to Indonesian cluster.	2,101
W are common to Bhutanese cluster, and A are common to Indonesian cluster.	903
W are common to Bhutanese cluster, and G are common to Indonesian cluster.	3
W are common to Bhutanese cluster, and T are common to Indonesian cluster.	869
S are common to Bhutanese cluster, and C are common to Indonesian cluster.	547
S are common to Bhutanese cluster, and G are common to Indonesian cluster.	539
S are common to Bhutanese cluster, and T are common to Indonesian cluster.	1
Y are common to Bhutanese cluster, and A are common to Indonesian cluster.	1
Y are common to Bhutanese cluster, and C are common to Indonesian cluster.	2,149
Y are common to Bhutanese cluster, and T are common to Indonesian cluster.	2,255
K are common to Bhutanese cluster, and A are common to Indonesian cluster.	3
K are common to Bhutanese cluster, and G are common to Indonesian cluster.	769
K are common to Bhutanese cluster, and T are common to Indonesian cluster.	766

Category D.<br>Unique homozygous genotypes are common to the Bhutanese cluster.<br>Unique heterozygous genotypes are common to the Indonesian cluster.	10,912
A are common to Bhutanese cluster, and M are common to Indonesian cluster.	536
C are common to Bhutanese cluster, and M are common to Indonesian cluster.	471
G are common to Bhutanese cluster, and M are common to Indonesian cluster.	1
A are common to Bhutanese cluster, and R are common to Indonesian cluster.	1,702
G are common to Bhutanese cluster, and R are common to Indonesian cluster.	1,529
T are common to Bhutanese cluster, and R are common to Indonesian cluster.	1
A are common to Bhutanese cluster, and W are common to Indonesian cluster.	702
G are common to Bhutanese cluster, and W are common to Indonesian cluster.	3
T are common to Bhutanese cluster, and W are common to Indonesian cluster.	682
C are common to Bhutanese cluster, and S are common to Indonesian cluster.	438
G are common to Bhutanese cluster, and S are common to Indonesian cluster.	427
C are common to Bhutanese cluster, and Y are common to Indonesian cluster.	1,524
G are common to Bhutanese cluster, and Y are common to Indonesian cluster.	1
T are common to Bhutanese cluster, and Y are common to Indonesian cluster.	1,715
A are common to Bhutanese cluster, and K are common to Indonesian cluster.	2
C are common to Bhutanese cluster, and K are common to Indonesian cluster.	1
G are common to Bhutanese cluster, and K are common to Indonesian cluster.	617
T are common to Bhutanese cluster, and K are common to Indonesian cluster.	560
Category E.<br>Unique heterozygous genotypes are common to the Bhutanese cluster.<br>Identical heterozygous genotypes and/or homozygous genotypes derived from the heterozygous genotypes are present in the Indonesian cluster.	1,432
M are common to Bhutanese cluster, and M and A are present in Indonesian cluster.	58
M are common to Bhutanese cluster, and M and C are present in Indonesian cluster.	63
M are common to Bhutanese cluster, and A and C are present in Indonesian cluster.	2
R are common to Bhutanese cluster, and R and A are present in Indonesian cluster.	212
R are common to Bhutanese cluster, and R and G are present in Indonesian cluster.	194
R are common to Bhutanese cluster, and A and G are present in Indonesian cluster.	15
W are common to Bhutanese cluster, and W and A are present in Indonesian cluster.	78
W are common to Bhutanese cluster, and W and T are present in Indonesian cluster.	93
W are common to Bhutanese cluster, and A and T are present in Indonesian cluster.	8
W are common to Bhutanese cluster, and W, A and T are present in Indonesian cluster.	2
S are common to Bhutanese cluster, and S and C are present in Indonesian cluster.	68
S are common to Bhutanese cluster, and S and G are present in Indonesian cluster.	46
S are common to Bhutanese cluster, and C and G are present in Indonesian cluster.	5
Y are common to Bhutanese cluster, and Y and C are present in Indonesian cluster.	192
Y are common to Bhutanese cluster, and Y and T are present in Indonesian cluster.	207
Y are common to Bhutanese cluster, and C and T are present in Indonesian cluster.	27
Y are common to Bhutanese cluster, and Y, C and T are present in Indonesian cluster.	4
K are common to Bhutanese cluster, and K and G are present in Indonesian cluster.	76
K are common to Bhutanese cluster, and K and T are present in Indonesian cluster.	79
K are common to Bhutanese cluster, and G and T are present in Indonesian cluster.	1
K are common to Bhutanese cluster, and K, G and T are present in Indonesian cluster.	2

Category F.<br>Unique heterozygous genotypes are common to the Indonesian cluster.<br>Identical heterozygous genotypes and/or homozygous genotypes derived from the heterozygous genotypes are present in the Bhutanese cluster.	134
M and A are present in Bhutanese cluster, and M are common to Indonesian cluster.	4
M and C are present in Bhutanese cluster, and M are common to Indonesian cluster.	4
R and A are present in Bhutanese cluster, and R are common to Indonesian cluster.	21
R and G are present in Bhutanese cluster, and R are common to Indonesian cluster.	24
W and A are present in Bhutanese cluster, and W are common to Indonesian cluster.	3
W and T are present in Bhutanese cluster, and W are common to Indonesian cluster.	6
S and C are present in Bhutanese cluster, and S are common to Indonesian cluster.	11
S and G are present in Bhutanese cluster, and S are common to Indonesian cluster.	3
Y and C are present in Bhutanese cluster, and Y are common to Indonesian cluster.	19
Y and T are present in Bhutanese cluster, and Y are common to Indonesian cluster.	27
K and G are present in Bhutanese cluster, and K are common to Indonesian cluster.	5
K and T are present in Bhutanese cluster, and K are common to Indonesian cluster.	7
Category G.<br>Unique homozygous genotypes are common to the Bhutanese cluster.<br>Unique heterozygous genotypes and homozygous genotypes derived from the heterozygous genotypes are present in the Indonesian cluster.	3,087
A are common to Bhutanese cluster, and M and A are present in Indonesian cluster.	129
A are common to Bhutanese cluster, and M and C are present in Indonesian cluster.	29
A are common to Bhutanese cluster, and M, A and C are present in Indonesian cluster.	2
C are common to Bhutanese cluster, and M and A are present in Indonesian cluster.	27
C are common to Bhutanese cluster, and M and C are present in Indonesian cluster.	126
A are common to Bhutanese cluster, and R and A are present in Indonesian cluster.	342
A are common to Bhutanese cluster, and R and G are present in Indonesian cluster.	108
A are common to Bhutanese cluster, and R, A and G are present in Indonesian cluster.	5
G are common to Bhutanese cluster, and R and A are present in Indonesian cluster.	100
G are common to Bhutanese cluster, and R and G are present in Indonesian cluster.	405
G are common to Bhutanese cluster, and R, A and G are present in Indonesian cluster.	7
A are common to Bhutanese cluster, and W and A are present in Indonesian cluster.	165
A are common to Bhutanese cluster, and W and T are present in Indonesian cluster.	38
A are common to Bhutanese cluster, and W, A and T are present in Indonesian cluster.	4
T are common to Bhutanese cluster, and W and A are present in Indonesian cluster.	33
T are common to Bhutanese cluster, and W and T are present in Indonesian cluster.	170
T are common to Bhutanese cluster, and W, A and T are present in Indonesian cluster.	4
C are common to Bhutanese cluster, and S and C are present in Indonesian cluster.	82
C are common to Bhutanese cluster, and S and G are present in Indonesian cluster.	13
C are common to Bhutanese cluster, and C, C and G are present in Indonesian cluster.	1
G are common to Bhutanese cluster, and S and C are present in Indonesian cluster.	19
G are common to Bhutanese cluster, and S and G are present in Indonesian cluster.	74
G are common to Bhutanese cluster, and S, C and G are present in Indonesian cluster.	1
C are common to Bhutanese cluster, and Y and C are present in Indonesian cluster.	333
C are common to Bhutanese cluster, and Y and T are present in Indonesian cluster.	97
C are common to Bhutanese cluster, and Y, C and T are present in Indonesian cluster.	7
T are common to Bhutanese cluster, and Y and C are present in Indonesian cluster.	89

T are common to Bhutanese cluster, and Y and T are present in Indonesian cluster.	337
T are common to Bhutanese cluster, and Y, C and T are present in Indonesian cluster.	9
G are common to Bhutanese cluster, and K and G are present in Indonesian cluster.	132
G are common to Bhutanese cluster, and K and T are present in Indonesian cluster.	30
G are common to Bhutanese cluster, and K, G and T are present in Indonesian cluster.	1
T are common to Bhutanese cluster, and K and G are present in Indonesian cluster.	26
T are common to Bhutanese cluster, and K and T are present in Indonesian cluster.	141
T are common to Bhutanese cluster, and K, G and T are present in Indonesian cluster.	1
Category H.<br>Unique homozygous genotypes are common to the Indonesian cluster.<br>Unique heterozygous genotypes and homozygous genotypes derived from the heterozygous genotypes are present in the Bhutanese cluster.	597
M and A are present in Bhutanese cluster, and A are common to Indonesian cluster.	14
M and C are present in Bhutanese cluster, and A are common to Indonesian cluster.	6
M and A are present in Bhutanese cluster, and C are common to Indonesian cluster.	15
M and C are present in Bhutanese cluster, and C are common to Indonesian cluster.	17
R and A are present in Bhutanese cluster, and A are common to Indonesian cluster.	68
R and G are present in Bhutanese cluster, and A are common to Indonesian cluster.	27
R and A are present in Bhutanese cluster, and G are common to Indonesian cluster.	49
R and G are present in Bhutanese cluster, and G are common to Indonesian cluster.	69
R, A and G are present in Bhutanese cluster, and G are common to Indonesian cluster.	4
W and A are present in Bhutanese cluster, and A are common to Indonesian cluster.	22
W and T are present in Bhutanese cluster, and A are common to Indonesian cluster.	12
W and A are present in Bhutanese cluster, and T are common to Indonesian cluster.	8
W and T are present in Bhutanese cluster, and T are common to Indonesian cluster.	21
W, A and T are present in Bhutanese cluster, and T are common to Indonesian cluster.	1
S and C are present in Bhutanese cluster, and C are common to Indonesian cluster.	15
S and G are present in Bhutanese cluster, and C are common to Indonesian cluster.	3
S, C and G are present in Bhutanese cluster, and C are common to Indonesian cluster.	2
S and C are present in Bhutanese cluster, and G are common to Indonesian cluster.	11
S and G are present in Bhutanese cluster, and G are common to Indonesian cluster.	8
Y and C are present in Bhutanese cluster, and C are common to Indonesian cluster.	62
Y and T are present in Bhutanese cluster, and C are common to Indonesian cluster.	27
Y and C are present in Bhutanese cluster, and T are common to Indonesian cluster.	25
Y and T are present in Bhutanese cluster, and T are common to Indonesian cluster.	42
K and G are present in Bhutanese cluster, and G are common to Indonesian cluster.	23
K and T are present in Bhutanese cluster, and G are common to Indonesian cluster.	10
K and G are present in Bhutanese cluster, and T are common to Indonesian cluster.	11
K and T are present in Bhutanese cluster, and T are common to Indonesian cluster.	24
K, G and T are present in Bhutanese cluster, and T are common to Indonesian cluster.	1
Category I.<br>Heterozygous and homozygous genotypes are distributed among eight accessions.	242
Category J.<br>Unique homozygous genotypes are common to the Bhutanese cluster.<br>Identical homozygous genotypes and other homozygous genotypes are present in the Indonesian cluster.	242
A are common to Bhutanese cluster, and A and C are present in Indonesian cluster.	8

A are common to Bhutanese cluster, and A and G are present in Indonesian cluster.	26
A are common to Bhutanese cluster, and A and T are present in Indonesian cluster.	21
C are common to Bhutanese cluster, and A and C are present in Indonesian cluster.	23
C are common to Bhutanese cluster, and C and G are present in Indonesian cluster.	6
C are common to Bhutanese cluster, and C and T are present in Indonesian cluster.	25
C are common to Bhutanese cluster, and A, C and T are present in Indonesian cluster.	1
G are common to Bhutanese cluster, and A and G are present in Indonesian cluster.	48
G are common to Bhutanese cluster, and C and G are present in Indonesian cluster.	6
G are common to Bhutanese cluster, and G and T are present in Indonesian cluster.	14
G are common to Bhutanese cluster, and A, C and G are present in Indonesian cluster.	1
G are common to Bhutanese cluster, and A, G and T are present in Indonesian cluster.	1
G are common to Bhutanese cluster, and C, G and T are present in Indonesian cluster.	1
T are common to Bhutanese cluster, and A and T are present in Indonesian cluster.	17
T are common to Bhutanese cluster, and C and T are present in Indonesian cluster.	32
T are common to Bhutanese cluster, and G and T are present in Indonesian cluster.	11
T are common to Bhutanese cluster, and A and C are present in Indonesian cluster.	1
Category K.<br>Two kinds of homozygous genotypes are present in the Bhutanese cluster.<br>Unique homozygous genotypes are common to the Indonesian cluster.	2
A and T are present in Bhutanese cluster, and A are common to Indonesian cluster.	1
A and G are present in Bhutanese cluster, and G are common to Indonesian cluster.	1
Category L.<br>Different kinds of heterozygous genotypes are distributed among eight accessions.	197
Total number of genotypes.	42,613

Supplementary Table S4. Number of genotype matches between each accession of the Bhutanese cluster in category F.

	Bhutan-09015	Bhutan-09024	Bhutan-09027
Bhutan-09024	78		
Bhutan-09027	52	50	
Bhutan-09030	65	131	69

Supplementary Table S5. Number of genotype matches between each accession of the Bhutanese cluster in category H.

	Bhutan-09015	Bhutan-09024	Bhutan-09027
Bhutan-09024	342		
Bhutan-09027	213	215	
Bhutan-09030	280	257	287

Supplementary Table S6. Number of genotype matches between each accession in category I.

	Bhutan-09015	Bhutan-09024	Bhutan-09027	Bhutan-09030	Bhutan-09005	Indonesia-88035	Indonesia-88045
Bhutan-09024	140						
Bhutan-09027	136	120					
Bhutan-09030	134	120	169				
Bhutan-09005	90	71	119	110			
Indonesia-88035	91	103	66	57	53		
Indonesia-88045	82	106	66	55	53	179	
Indonesia-88065	92	86	66	88	131	110	111

Supplementary Figure S1.

The depth of RAD-seq coverage for the sample Bhutan-09015 across a reference genome.

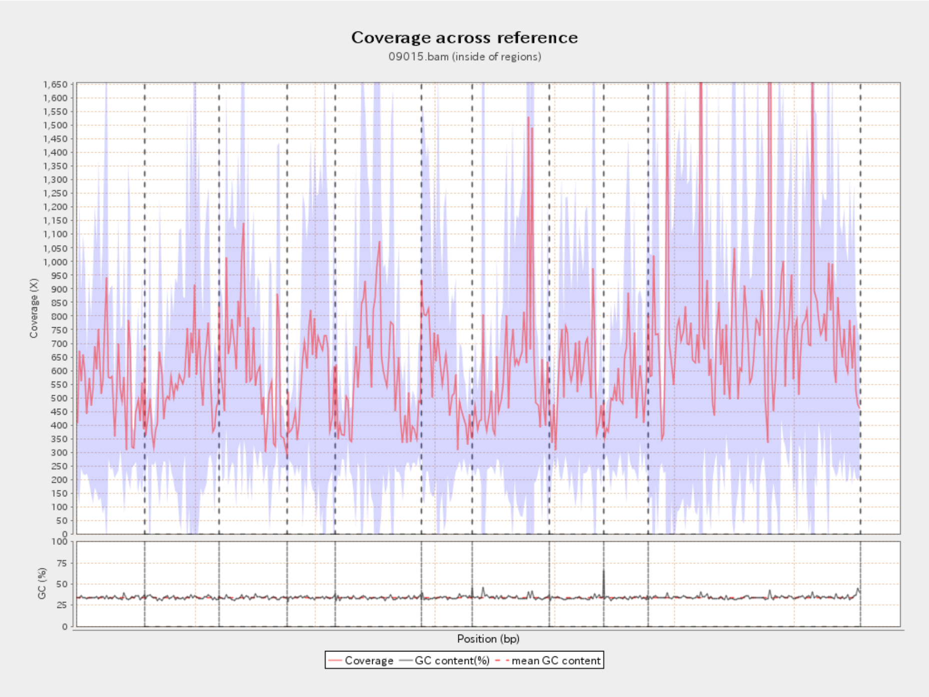

The coverage histogram for the sample Bhutan-09015.

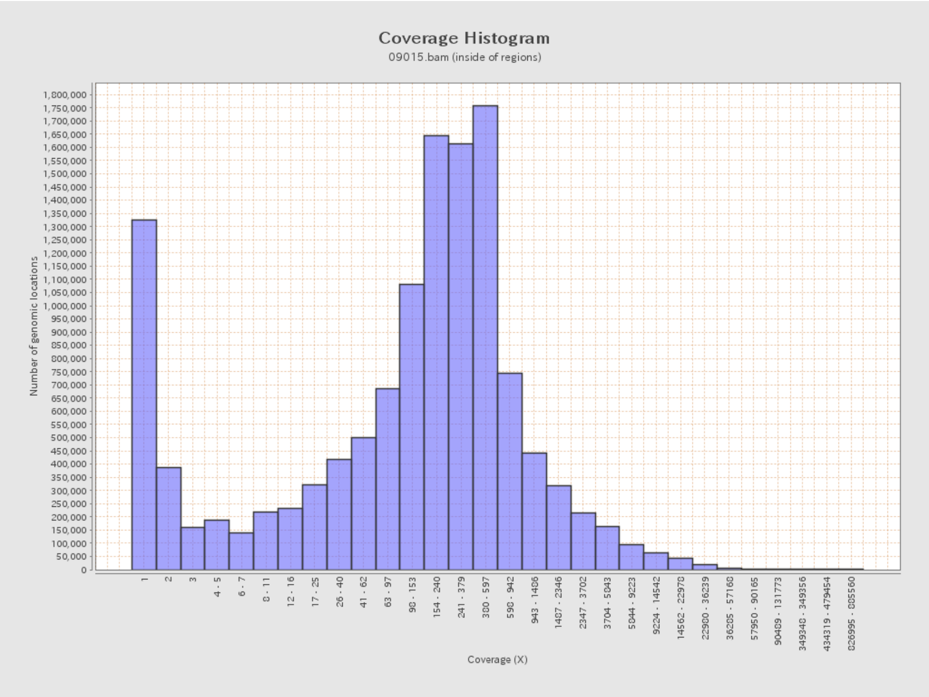

The depth of RAD-seq coverage for the sample Bhutan-09024 across a reference genome.

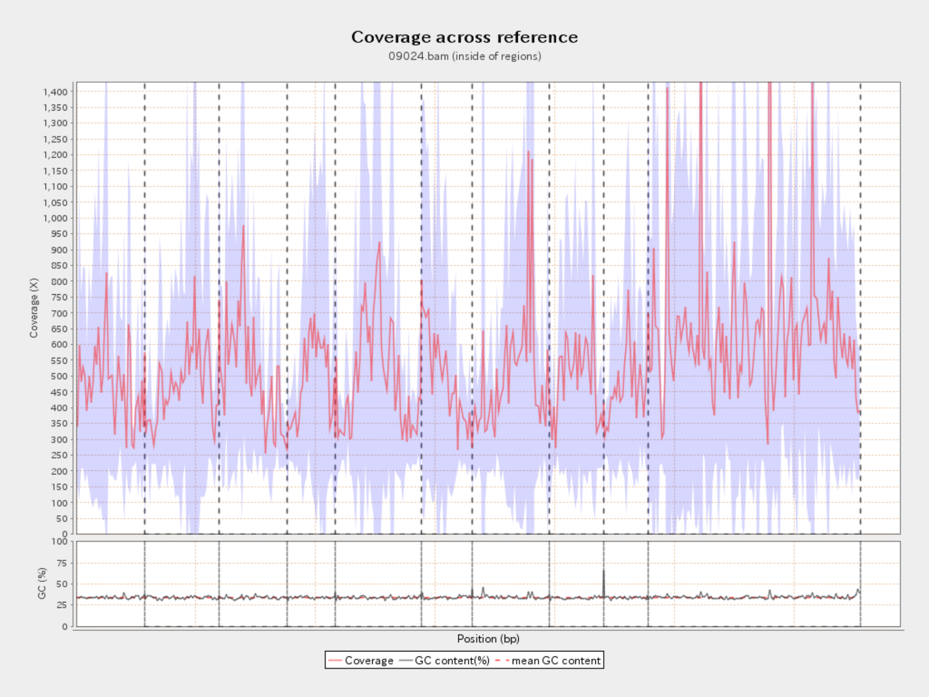

The coverage histogram for the sample Bhutan-09024.

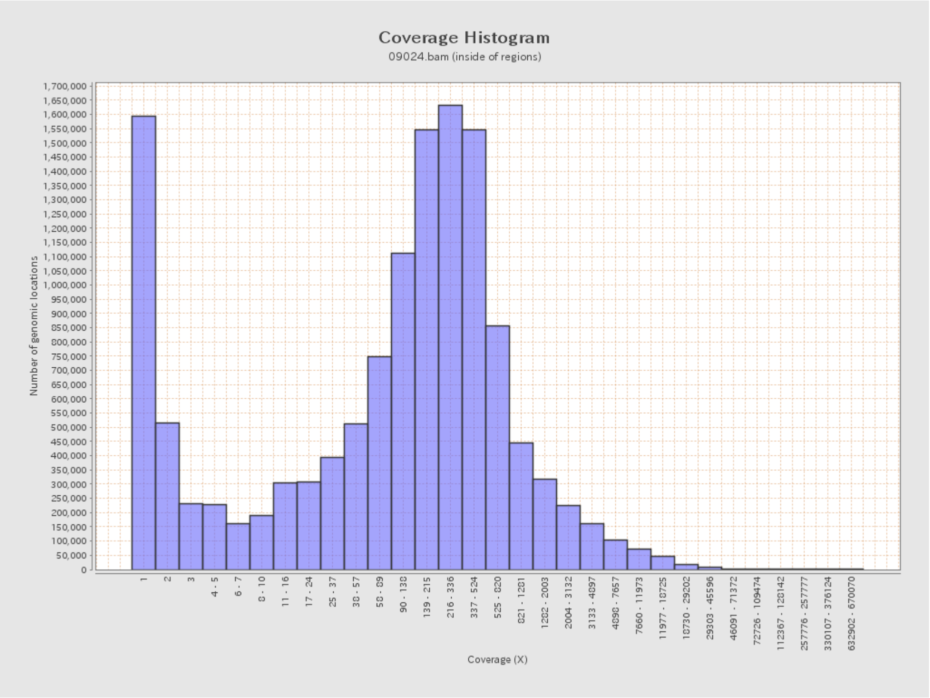

The depth of RAD-seq coverage for the sample Bhutan-09027 across a reference genome.

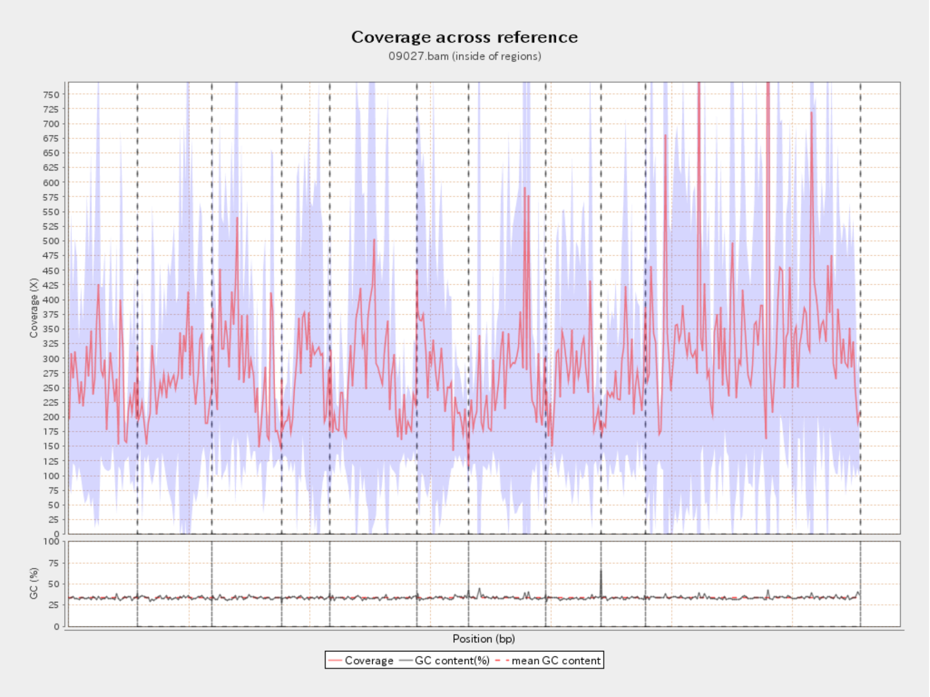

The coverage histogram for the sample Bhutan-09027.

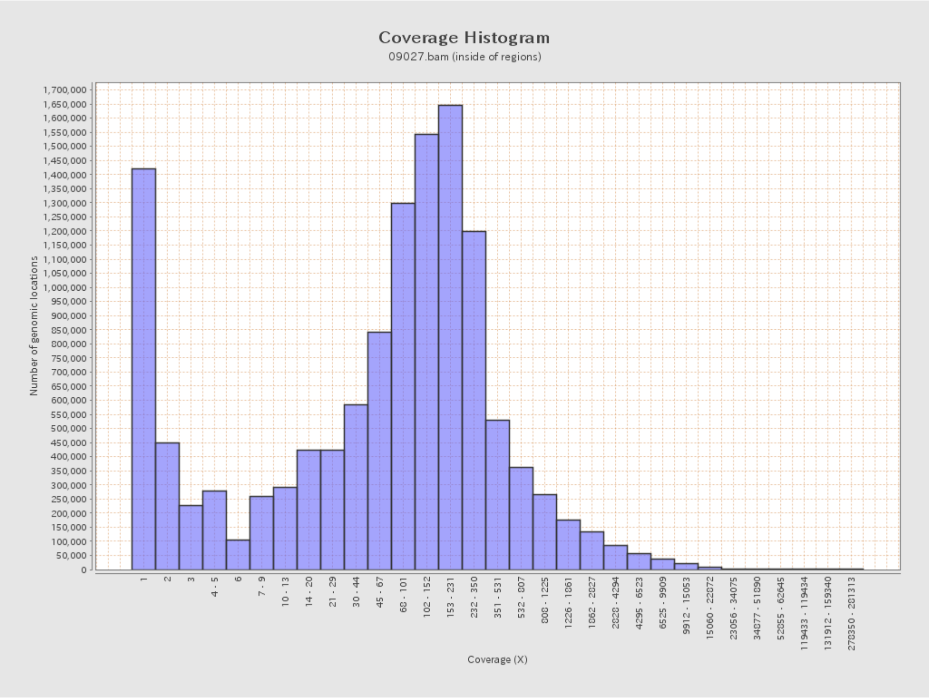

The depth of RAD-seq coverage for the sample Bhutan-09030 across a reference genome.

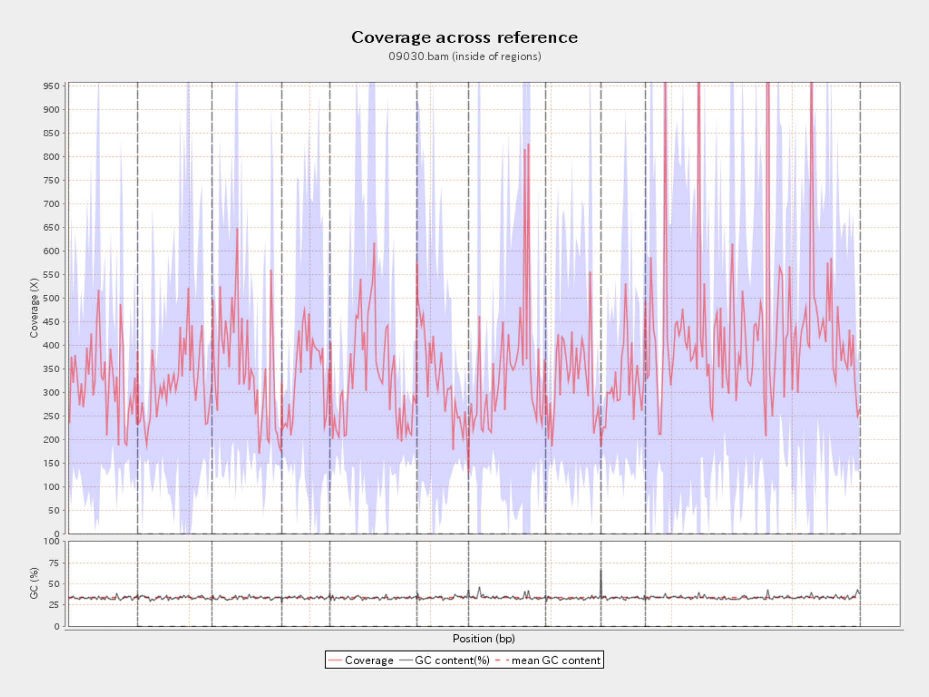

The coverage histogram for the sample Bhutan-09030.

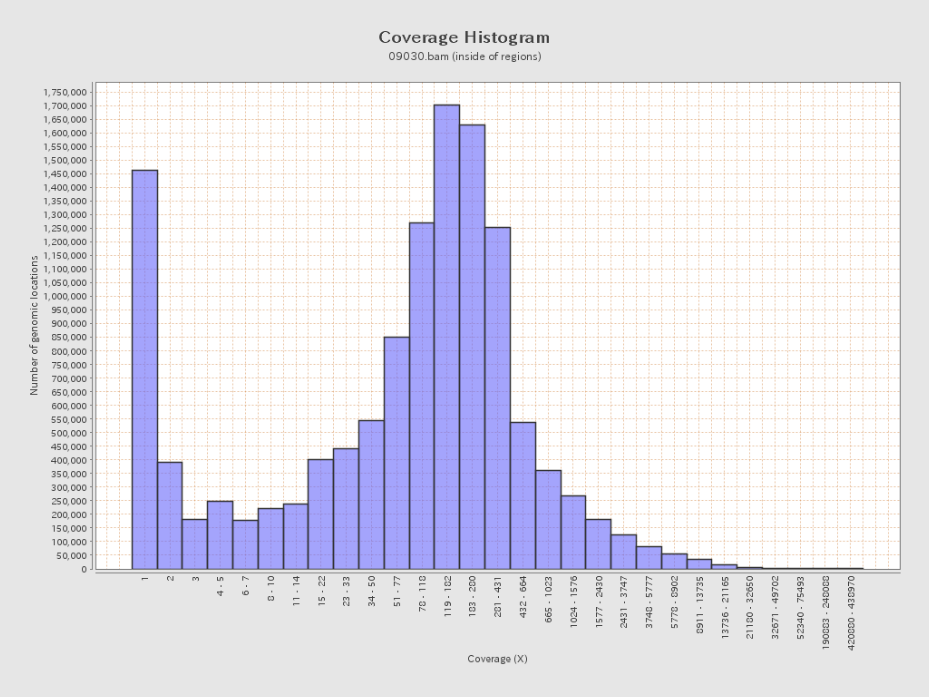

The depth of RAD-seq coverage for the sample Bhutan-09005 across a reference genome.

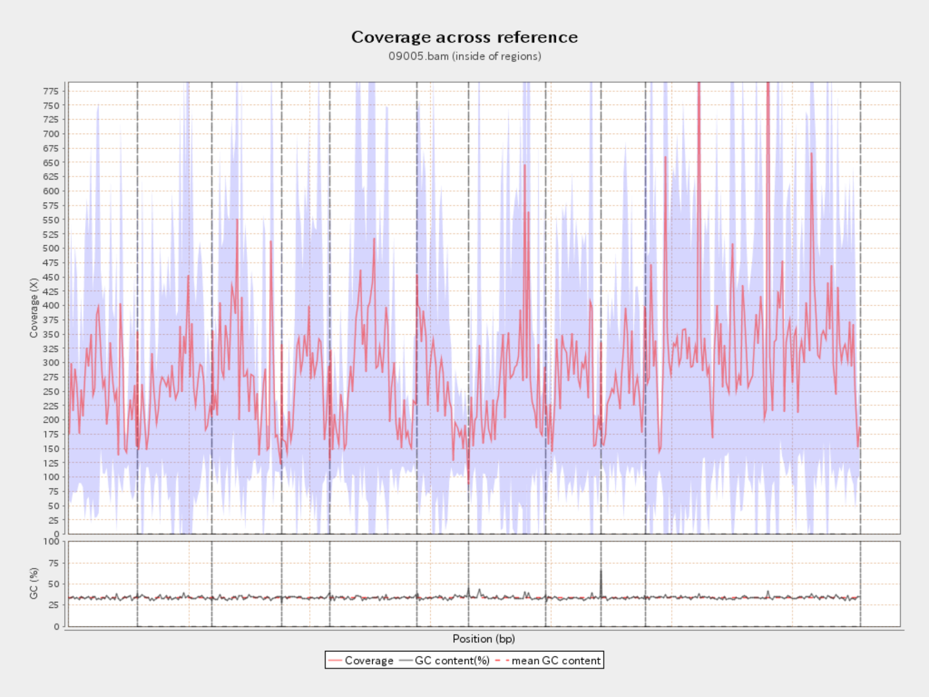

The coverage histogram for the sample Bhutan-09005.

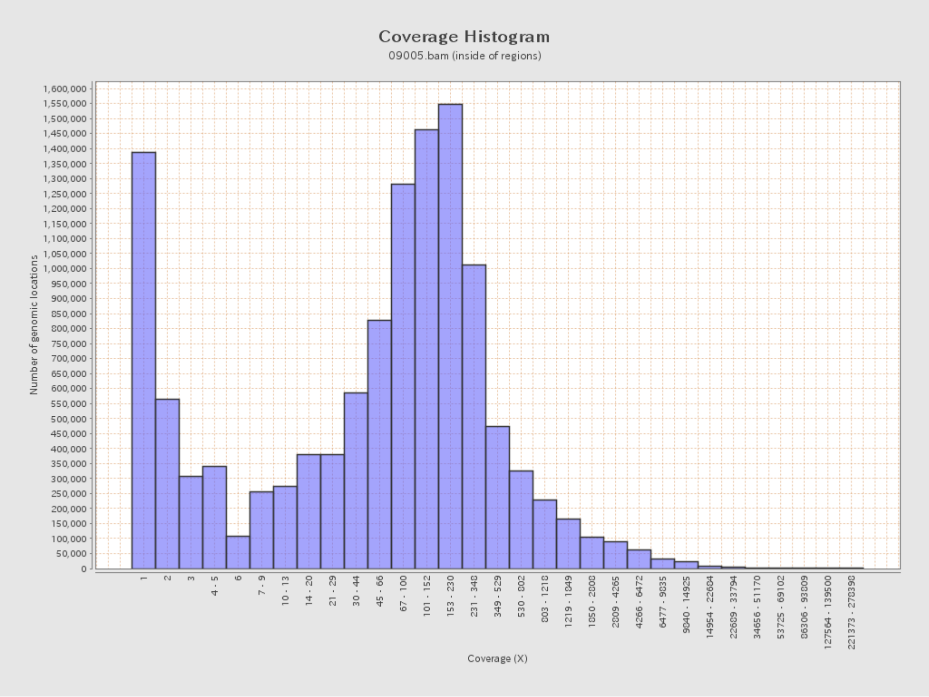

The depth of RAD-seq coverage for the sample Bhutan-88035 across a reference genome.

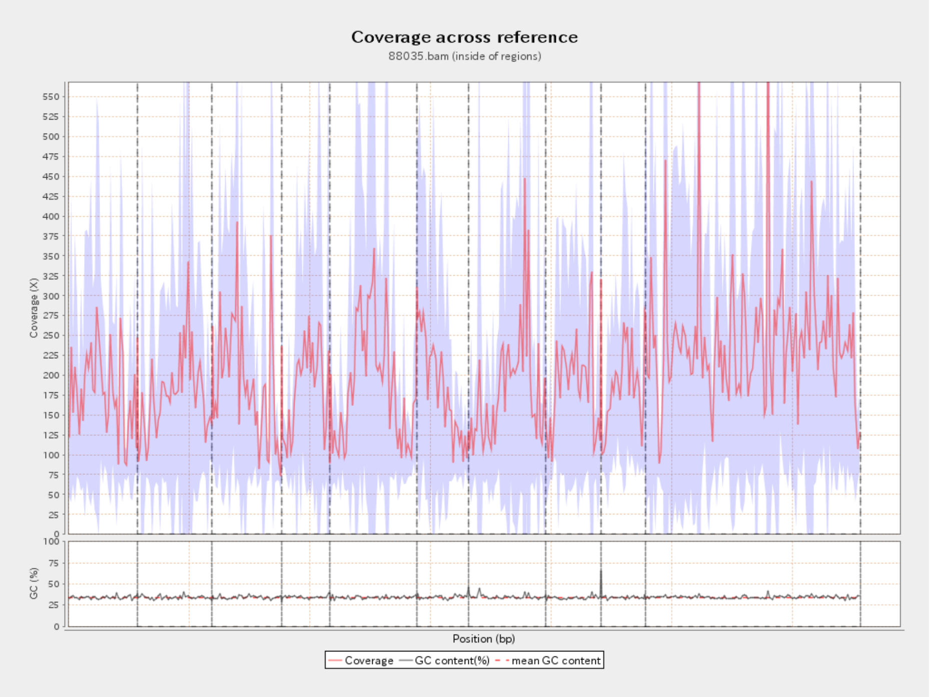

The coverage histogram for the sample Bhutan-88035.

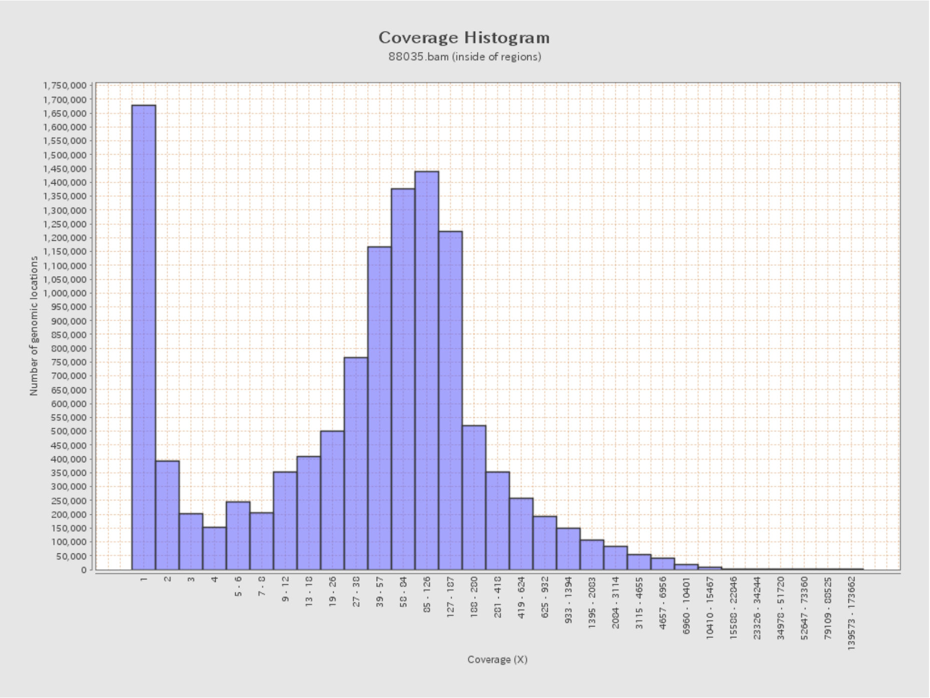

The depth of RAD-seq coverage for the sample Bhutan-88045 across a reference genome.

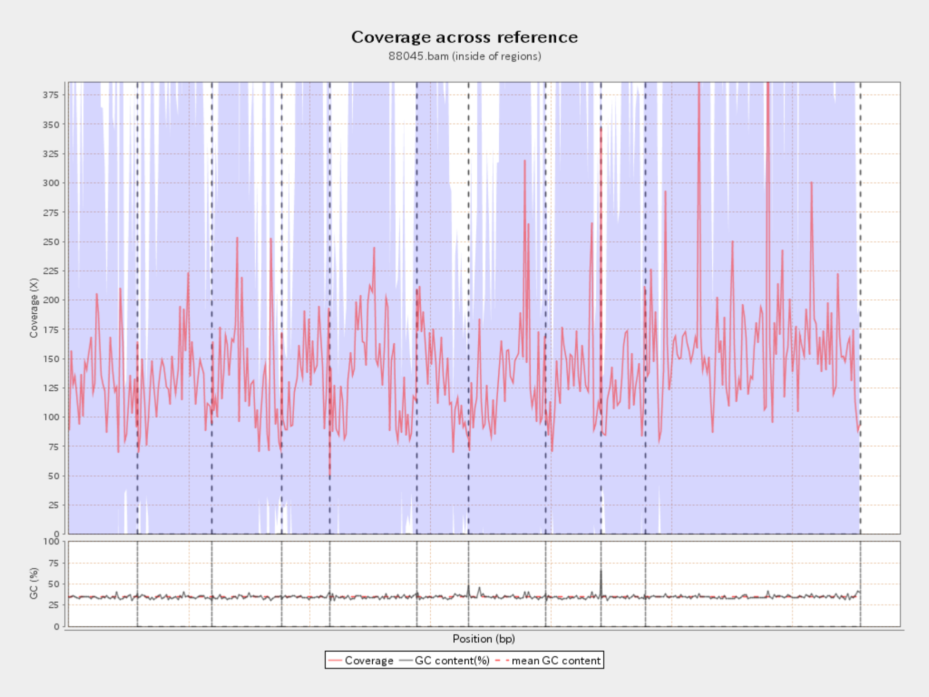

The coverage histogram for the sample Bhutan-88045.

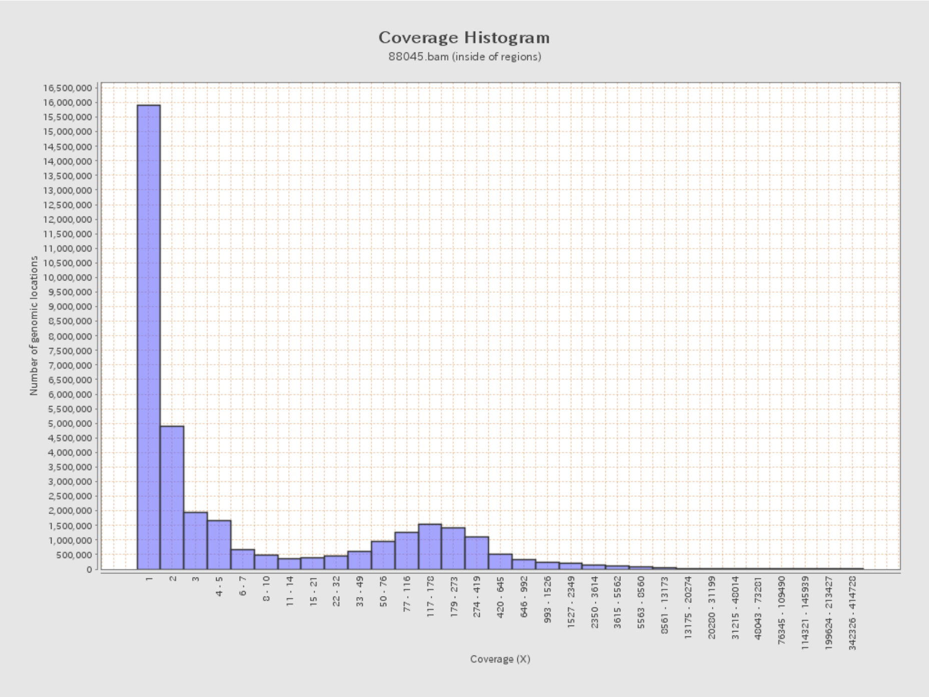

The depth of RAD-seq coverage for the sample Bhutan-88065 across a reference genome.

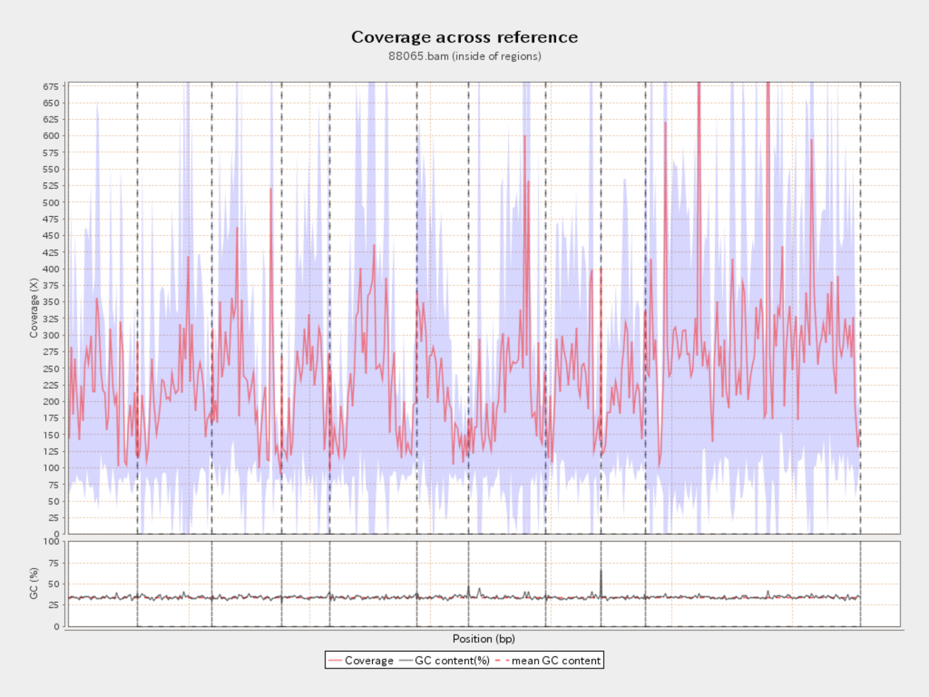

The coverage histogram for the sample Bhutan-88065.

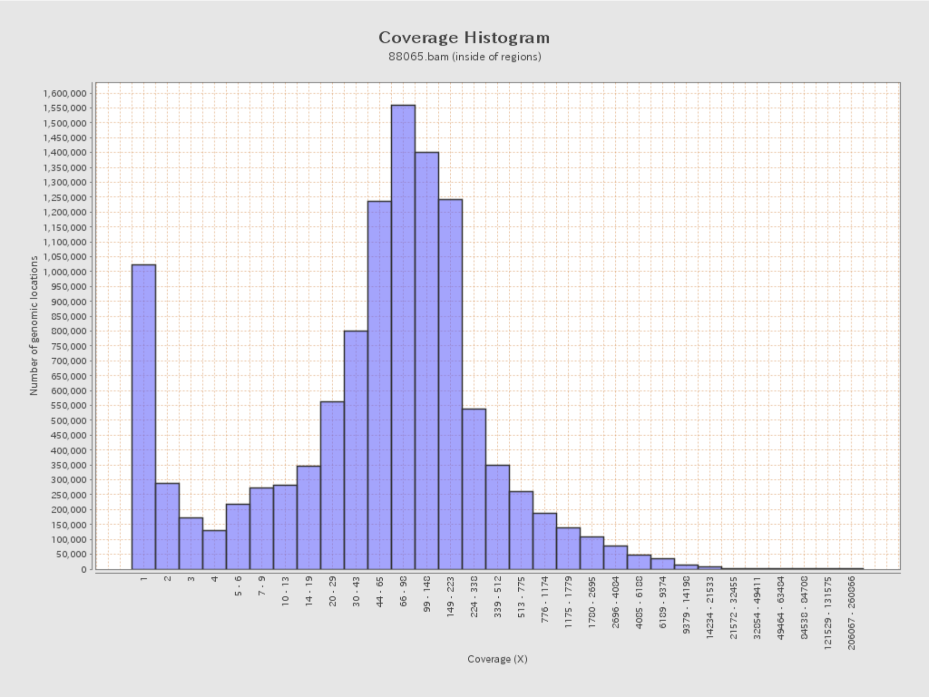

Supplementary Figure S2.

```
CLUSTAL 2.1 multiple sequence alignment


Bhutan-09015         TGGTYAAGTTCCSRYTCRTCATGACKTSWRARYTRRCRCMWTGTRAGSGRYATRSTAGYY 60
Bhutan-09024         TGGTYAAGTTCCSRYTCRTCATGACKTSWRARYTRRCRCMWTGTRAGSGRYATRSTAGYY 60
Bhutan-09027         TGGTYAAGTTCCSRYTCRTCATGACKTSWRARYTRRCRCMWTGTRAGSGRYATRSTAGYY 60
Bhutan-09030         TGGTYAAGTTCCSRYTCRTCATGACKTSWRARYTRRCRCMWTGTRAGSGRYATRSTAGYY 60
Bhutan-09005         YRRAYTTAYCTTCACKSRWTRYGGTKAYAAWGTYGATACAACRYARACKGAGYGGWRRCY 60
Indonesia-88035      YRRAYTTAYCTTCACKSRWTRYGGTKAYAAWGTYGAYATAACRYARACKGAGYGGWRRCY 60
Indonesia-88045      YRRATTTAYCTTCACKSRWTRYKGTKAYAAWGTYGAYATAACRYARACKGAGYGGWRRCY 60
Indonesia-88065      YRRAYTTAYCTTCACKSRWTRYGGTKAYAAWGTYGAYATAACRYARACKGAGYGGWRRCY 60
                        : ::.    .  ..*     . *:                   ..   .  .    *

Bhutan-09015         YWTWGYYSRACKGYMATYTYWWYKCSRCRAYKAKKAGGMASRTGYCGRRMKATRCARRAG 120
Bhutan-09024         YWTWGYYSRWSKGYMATYTYWWYKCSRCRAYKAKKAGGMASRTGYCGRRMKATRCARRAG 120
Bhutan-09027         YWTWGYYSRWSKGYMATYTYWWYKCSRCRACGAKKAGGMASRTGYCGRRMKATRCARRAG 120
Bhutan-09030         YWTWGYYSRWSKGYMATYTYWWYKCSRCRACGAKKAGGMASRTGYCGRRMKATRCARRAG 120
Bhutan-09005         CTGWCTCCGASGRYMMYYGCTTCGSGGMAWCGMTTTRTACGATKTARRGCGGYRSRARMR 120
Indonesia-88035      CTGWCTCCGASGRYMMYYGCTTCGSGGMAWCGMTTTRTACGAYGTARRGCGGYRSRARMR 120
Indonesia-88045      CTGWCTCCGASGRYMMYYGCTTCGSGGMAWCGMTTTRTACGATKTARRGCGGYRSRARMR 120
Indonesia-88065      CTGWCTCCGASGRYMMYYGCTTCGSGGMAWCGMTTTRTACGAYGTARRGCGGYRSRARMR 120
                        *   .  .  **  *      ..       ..:    ..   . *   .*.   *

Bhutan-09015         RYRRRGGCGGTTRTMTYYGTCWTYRGGRKWGTRRRYAGTTTCYYSKYSTCRGTSRRMYMY 180
Bhutan-09024         RYRRRGGCGGTTRTMTYYGTCWTYRGGRKWGTRRRYAGTTTCYYSKYSTCRGTSRRMYMY 180
Bhutan-09027         RYRRRGGCGGTTRTMTYYGTCWTYRGGRKWGTRRRYAGTTTCYYSKYSTCRGTSRRMYMY 180
Bhutan-09030         RYRRRGGCGGTTRTMTYYGTCWTYRGGRKWGTRRRYAGTTTCYYSKYSTCRGTSRRMYMY 180
Bhutan-09005         GCGRRTSAARWKGYAACTRCMWYYRRKATARTARRYRKCGYATCCGCSAMRRYSAGATCT 180
Indonesia-88035      GCGRRTSAARWKGYAACTRCMWYYRRKATARTARRYRKCGYATCCGCSAMRRYSAGATCT 180
Indonesia-88045      GCGRRTSAARWKGYAACTRCMWYYRRKATARTARRYRKCGYATCCGCSAMRRYSAGATCT 180
Indonesia-88065      GCGRRTSAARWKGYAACTRCMWYYRRKATARKARRYRKCGYATCCGCSAMRRYSAGATCT 180
                        ** ...  .   :     * **   .  . ***     .  .  *: *  *

Bhutan-09015         RAGYMYTGYYGGTARRGAGCYKKCWRYAYYCRWYGTMRATGRYWYGCKGAMTATSGARGR 240
Bhutan-09024         RAGYMYTGYYGGTARRGAGCYKKCWRYAYYCRWYGTMRATGRYWYGCKGAMTATSGARGR 240
Bhutan-09027         RAGYMYTGYYGGTARRGAGCYKKCWRYAYYCRWYGTMRATGRYWYGCKGAMTATSGARGR 240
Bhutan-09030         RAGYMYTGYYGGTARRGAGCYKKCWRYAYYCRWYGTMRATGRYWYGCKGAMTATSGARGR 240
Bhutan-09005         ARRCACWRYTARKRRGCGTAYKGTTGTCTTMAWYKGCGWWRGCWYRYKARCYRKCKWACR 240
Indonesia-88035      ARRCACWRYTARKRRGCGTAYKGTTGTCTTMAWYKGCGWWRGCWYRYKAACYRKCKWACR 240
Indonesia-88045      ARRCAYWRYTARKRRGCGTAYKGTTGTCTTMAWYKGCGWWRGCWYRYKARCYRKCKWACR 240
Indonesia-88065      ARRCACWRYTARKRRGCGTAYKGTTGTCTTMAWYKGCGWWRGCWYRYKARCYRKCKWACR 240
                             * . . *  . .**     .    **         **  *.    ..    *

Bhutan-09015         CMYGGYKWYCGAAARTCYWARTAAKCGAYRYTTRRAAYWYRAKSRCGYYTRCRKWRKKAY 300
Bhutan-09024         CCTGGYKWYCGAAARTCYWARTAAKCGAYRYTTRRAAYWYRRKSRCGYYTRCRKWRKKAT 300
Bhutan-09027         CMYGGYKWYCGAAARTCYWARTAAKCGAYRYTTRRAAYWYRRKSRCGYYTRCRGTRKKAY 300
Bhutan-09030         CMYGGYKWYCGAAARTCYWARTAAKCGAYRYTTRRAAYWYRRKSRCGYYTRCRGTRKKAY 300
Bhutan-09005         MCTRSYKATCGCGWAWATWMRCGMGTKRYRYYYGRRTTWCARKCAYRTTWGSRTAATTCT 300
Indonesia-88035      MCTRSYKATCRCGWAWATWMRCGMGTKRYRYYYGRRTTWCAAKCAYRTTWGSRTAATTCT 300
Indonesia-88045      MCTRSYKATMGCGWAWATWMRCGMGTKRYRYYYGRRTTWCAAKCAYRTTWGSRTAATTCT 300
Indonesia-88065      MCTRSYKATCGCGWAWATWMRCGMGTKRYRYYYGRRTTWCAAKCAYRTTWGSRTAATTCY 300
```

```
                         .**    ..   . * * .     ***   * : *   *.       .*   ...

Bhutan-09015         TSRWCTGWWTTTRTGCRTWRRTCATASWWTAYSRYMKRRGMRYCGYGRMAAWAGRSGRKY 360
Bhutan-09024         TSRWCTGWWTTTRTGCRTWRRTCATASWWTAYSRYMKRRGMRYCGYGRMAAWAGRSGRKY 360
Bhutan-09027         TSRWCTGWWTTTRTGCRTWRRTCATASWWTAYSRYMKRRGMRYCGYGRMAAWAGRSGRKY 360
Bhutan-09030         TSRWCTGWWTTTRTGCRTWRRTCATASWWTAYSRYMKRRGMRYCRYGRMAAWAGRSGRKY 360
Bhutan-09005         YMRWTKTWWWYKGCRYGYTARKGGYWSAWYMTCRYMTRRAAGCMRTRRAWRARRRSRAKY 360
Indonesia-88035      TMRWYKTWWWYKGCRYGYTARKGGYWSAWYMTCRYMTRRAAGCMRTRRATRARRRSRAKY 360
Indonesia-88045      YMRWYKTWWACKGCRYGYTARKGGYWSAWYMTCRYMTRRAAGCMRTRRATRARRRSRAKY 360
Indonesia-88065      TMRWYKTWWWYKGCRYGYTARKGGYWSAWYMTCRYMTRRAAGCMRTRRATRARRRSRAKY 360
                       ** . **  .        *. .  * *   .***.**.       *      **  **

Bhutan-09015         RMSMRYRRSGARYKYRYTCATYTGAWRCGAYARAYGAAGWGGTGGCTRRRYRYCGRWRTA 420
Bhutan-09024         RMSMRYRRSGARYKYRYTCATYTGAWRCGAYARAYGAAGWGGTRGCTRRRYRYCGRWRTA 420
Bhutan-09027         RMSMRYRRSGARYKYRYTCATYTGAWRCGAYARAYGAAGWGGTGGCTRRRYRYCGRWRTA 420
Bhutan-09030         RMSMRYRRSGARYKYRYTCATYTGAWRCGAYARAYGAAGWGGTGGCTRRRYRYCGRWRTA 420
Bhutan-09005         RASMGCARSRMGCTYRYGMWYYGCRWACRRCRARYKRMKWSRTGKSKRGAYATTAAAAKR 420
Indonesia-88035      RASMGCARSRMGCTYRYGMWYYGCRWRYRRCRARYKRMKWSRTTKSKRGAYATTAAAAKR 420
Indonesia-88045      RASMGCARSRMGCTYRYGMWYYGCRWRYRRCRARYKRMKWSRATKSKRGAYATTAAAAKR 420
Indonesia-88065      RASMGCARSRMGCTYRYGMWYTGCRWACRRCRARYKRMKWSRWAKSKRGAYATTAAAAKR 420
                     * **   **    .***        *          *    *.    ..*  *   .   .

Bhutan-09015         AGTCGYRYRGRKKYMRKTWSKRYRWWRWTYRMRYTAWRYRCTGGACTYKRRWKKCYYAAR 480
Bhutan-09024         AGTCGYRYRGRKKYMRKTWSKRYRWWRWTYRMRYTAWRYRCTGGACTYKRRWKKCYYAAR 480
Bhutan-09027         AGTCGYRYRGRKKYMRKTWSKRYRWWRWTYRMRYTAWRYRCTGGACTYKRRWKKCYYAAR 480
Bhutan-09030         AGTCGYRYRGRKKYMRKTWSKRYRWWRWTYRMRYTAWRYRCTGGACTYKRRWKKCYYAAR 480
Bhutan-09005         MAYYRTACGRAGGCMGGYTGTRYRAWRTKTACGCWRWGYRYWRRRSYYKRATGTATYGWG 480
Indonesia-88035      MAYTATACGRAGGCMGGYTGTRYRAWRTKTACGCWRWGYRYWRRRSYYKRATGTATYGWG 480
Indonesia-88045      MAYTATACGRAGGCMGGYTGTRYRAWRTKTACGCWRWGYRYWRRRSYYKRATGTATYGWG 480
Indonesia-88065      MAYYRTACGRAGGCMGGYTGTRYRAWRTKTACGCWRWGYRYWRRRSYYKRATGTATYGWG 480
                      .            *    ..*** ** .       * **     . ***   .. *.

Bhutan-09015         TGGGYYCTYWYTTWGRAWAGRKWYYCGTGWGMRSAGCYSCRGGGYYTGRWKAWYRGTCYS 540
Bhutan-09024         TGGGYYCTYWYTTWGRAWAGRKWYYCGTGWGMRSAGCYSCRGGGYYTGRWKAWYRGTCYS 540
Bhutan-09027         TGGGYYCTYWYTTWGRAWAGRKWYYCGTGWGMRSAGCYSCRGGGYYTGRWKAWYRGTCYS 540
Bhutan-09030         TGGGYYCTYWYTTWGRAWAGRKWYYCGTGWGMRSAGCYSCRGGGYYTGRWKAWYRGTCYS 540
Bhutan-09005         CTRKCTGATACAWWRRCTTRGGTTCYSYATAAASRRTYCYRRRKYTYRATGWWYRTYYCC 540
Indonesia-88035      CTRKCTGATACAWWRRCTTRGGTTCYSYATAAASRRTYCYRRRKYTYRATGWWYRTYYCC 540
Indonesia-88045      CTRKCTGATACAWWRRCTTRGGTTCYSYATAAASRRTYCYRRRKYTYRATGWWYRTYYCC 540
Indonesia-88065      CTRKCTGATACAWWRRCTTRGGTTCYSYATAAASRRTYCYRRRTYTYRATGWWYRTYYCC 540
                            :   : * *. :       . . .  *   *. *   *       ***    .

Bhutan-09015         MRARKCACCATCYAYWCGTTARTYAYAWGAYRAYARYYWRMYYRTYCACYTTTGAWCYYA 600
Bhutan-09024         MRARKCACCATCYAYWCGTTARTYAYAWGAYRAYARYYWRMYYRTYCACYTTTGAWCYYA 600
Bhutan-09027         MRARKCACCATCYAYWCGTTARTYAYAWGAYRAYARYYWRMYYRTYCRCYTTTGAWCYYA 600
Bhutan-09030         MRARKCACCATCYAYWCGTTARTYAYAWGAYRAYARYYWRMYYRTYCACYTTTGAWCYYA 600
Bhutan-09005         MRRGTTTTTGYYCCYWYRKYRRYTRCRTRRYRACCRYTTGATTRWYYRYYYCCAGWYCCR 600
Indonesia-88035      MRRGTTTTTGYYCCYWYRKYRRYTRCRTRRYRACCRYTTGATTRWYCAYYYCCAGWYCCR 600
Indonesia-88045      MRRGTTTTTGYYCCYWYRKYRRYTRCRTRRYRACCRYTTGATTRTYCAYYYCCAGWYCCR 600
Indonesia-88065      MRRGTTTTTGYYCCYWYRKYRRYTRCRTRRYRRCCRYTTGATTRWYCAYYYCCAGWYCCR 600
                     **  . :  .   .**  .  *        **  .**      * *   *   ..*

Bhutan-09015         YRRSYKTYRWTWAGWTYRSTGWKYTCGRWCTKAYATRATWRTTSTKYTYKYRTRSTRGYC 660
Bhutan-09024         YRRSYKTYRWTWAGWTYRSTGWKYTCGRWCTKAYATRATWRTTSTKYTYKYRTRSTRGYC 660
```

```
Bhutan-09027        YRRSYKTYRWTWAGWTYASTGWKYTCGRWCTKAYATRATWRTTSTKYTYKYRTRSTRGYC 660
Bhutan-09030        YRRSYKTYRWTWAGWTYRSTGWKYTCGRWYWKAYATRATWRTTSTKYTYKYRTRSTRGYC 660
Bhutan-09005        TAGGCAYYRWYTMATCCASKSTKYWYSRTYTKGYRKRTWWGYWGYKYTTTTRWACYMAYS 660
Indonesia-88035     TAGGCAYYRWYTMATCCASKSTKYWYSRTCTKGYRKRTWWGYWGYKYYTTTRWACYMAYS 660
Indonesia-88045     TAGGCAYYAAYTMATCCASKSTKYWYSRTCTKGYRKRTWWGYWGYKYYTTTRWACYMAYS 660
Indonesia-88065     TAGGCAYYRWYTMATCCASKSTKYWYSRTCTKGYRKRTWWGYWGYKYYTTTRWACYMAYS 660
                       .   *     .    *.. **  .*   *.* .*: *   . **  . *  .  .*.

Bhutan-09015        WYRSYRAGRYTYYWCMCGRRYWSCCKCYYRRCRWYYATKAMKRYTRRRRCMMGMSWTWWR 720
Bhutan-09024        WYRSYRAGRYTYYWCMCGRRYWSCCKCYYRRCRWYYATKAMKRYTRRRRCMMGMSWKWWR 720
Bhutan-09027        WYRSYRAGRYTYYWCMCGRRYWSCCKCYYRRCRWYYATKAMKRYTRRRRCMMGMSWKWWR 720
Bhutan-09030        WYRSYRAGRYTYYWCMCGRRYWSCCKCYYRRCRWYYATKAMTRYTRRRRCMMGMSWTWWR 720
Bhutan-09005        TYGCTGWSGTCTTASCYRAGTWGATTGYTGGYAWTYKYKMCKAYKGRGGMCARAGAKAWG 720
Indonesia-88035     TYGCTGWSGTCTTASCYRAGTWGATTGYTGGYAWTYKYKMCKAYKGRGGMCARAGATAWG 720
Indonesia-88045     TYGCTGWSGTCTTASCYRAGTWGATTGYTGGYAWTYKYKMCKAYKGRGGMCARAGATAWG 720
Indonesia-88065     TYGCTGWSGTCTTASCYRAGTWGATTGYTGGYAWTYKYKMCKAYKGRGGMCARAGATAWG 720
                     * .   .      .      *.. . *     * *  *  . *. *       . . *

Bhutan-09015        WSSTYYTTRCARRYRRMCYAGAWRCYRTRWWTMCKTTYYWYYAYGAAWRYWKRSCKCKTR 780
Bhutan-09024        WCSTYYTTRCARRYRRMCYAGAWRCYRTRWWTMCKTTYYWYYAYGAAWRYWKRSCKCKTR 780
Bhutan-09027        WSSTYYTTRCARRYRRMCYAGAWRCYRTRWWTMCKTTYYWYYAYGAAWRYWKRSCKCKTR 780
Bhutan-09030        WCSTYYTTRCARRYRRMCYAGAWRCYRTRWWTMCKTTYYWYYAYGAAWRYWKRSCKCKTR 780
Bhutan-09005        WCCWCCYWAYWRATGAAMYRRTTAMYRKATTYCYGYYCCACTGYGAATGTWTAGCGTKYR 780
Indonesia-88035     WCCWCCYWAYWRATGAAMYRRTTAMYRKATTYCYGYYCCACTGYGAATGTWTAGCGTKYR 780
Indonesia-88045     WCCWCCYWAYWRATGAAMYRRTTAMYRKATTYCYGYYCCACTGYGAATGTWTAGYKTKYR 780
Indonesia-88065     WCCWCCYWAYWRATGAAMYRRTTAMYRKATTYCYGYYCCACTGYRRWTGTWTAGCGTKYR 780
                    *..        *      *  :   **.              .*      *. .   * *

Bhutan-09015        ACYRSTTMRRRARAWTYKWWTYRKKSRYRTAAKYAWRGCRCKMKRAWGCTYSGRGWWGCC 840
Bhutan-09024        ACYRSTTMRRRARAWTYKWWTYRKKSRYRTAAKYAWRGCRCKMKRAWGCTYSGRGWWGCC 840
Bhutan-09027        ACYRSTTMRRRARAWTYKWWTYRKKSRYRTAAKYAWRGCRCKMKRAWGCTYSGRGWWGCC 840
Bhutan-09030        ACYRSTTMRRRARAWTYKWWTYRKKSRYRTAAKYAWRGCRCKMKRAWGCTYSGRGWWGCC 840
Bhutan-09005        RYCRGAAAAGAWRWAWCKAAYYAKGCRTRYWRTTGARRMGTTMTAGWATYCGRGRWTKYM 840
Indonesia-88035     RYCRGAAAAGAWRWAWCKAAYYAKGCRTRYWRTTGAGRMGTTMTAGWATYCGRGRWTKYM 840
Indonesia-88045     RYCRGAAAAGAWRWAWCKAAYYAKGCRTRYWRTTGARRMGTTMTAGWATYCGRGRWTKYM 840
Indonesia-88065     RYCRGAAAAGAWRWAWCKAAYYAKGCRTRYWRTTGARRMGTTMTAGWATYCGRGRWTKYM 840
                       *.::     *    *   * * .* *   . .      .*. .*.   .   *

Bhutan-09015        YGYCTMTRGGTRRSAGRYCCACCATYWMCTGRTTRAYYGTAMYTYYGCYRGGKAARCSRW 900
Bhutan-09024        YGYCTMTRGGTRRSAGRYCCACCATYWMCTGRTTRAYYGTAMYTYYGCYRGGKAARCSRW 900
Bhutan-09027        YGYCTMTRGGTRRSAGRYCYWCCATYWMCTGRTTRAYYGTAMYTYYGCYRGGKAARCSRW 900
Bhutan-09030        YGYCTMTRGGTRRSAGRYCCWCCATYWMCKGRTTRAYYGTAMYTYYGCYRGGKAARCSRW 900
Bhutan-09005        YRTMYCYARRCGASGRRYSCAAAWYTTCYTKRYYGRYYAYRAYYYYSYTARRKRWRYCAA 900
Indonesia-88035     YRTMYCYARRCGASGRRYSCAAAWYTTCTTKAYYGRYYAYRAYYYYSYTARRKRWRYCAA 900
Indonesia-88045     YRTMYCYARRCGASGRRYCCAAAWYTTCYTKRYYGRYYAYRAYYYYSYTARRKRWRYCAA 900
Indonesia-88065     YRTMYCYARRCGASGRRYSCAAATCTTCYTKRYYGRYYAYRAYYYYSYTARRKRWRYCAA 900
                    *            *. **.  ..      .      **.   * **.     *  * .

Bhutan-09015        MTRGAKRWWWAAARWWTKYAYACCACKCRATARMYWWGTTRRSKTYRRYCWRGAGYRYRC 960
Bhutan-09024        MTRGAKRWWWAAARWWTKYAYACCACKCRATARMYWWGTTRRSKTYRRYCWRGAGYRYRC 960
Bhutan-09027        MTRGAKRWWWAAARWWTKYAYACCACKCRATARMYWWGTTRRSKTYRRYCWRGAGYRYRC 960
Bhutan-09030        MTRGAKRWWWAAARWWTKYAYACCACKCRATARMYWWGTTRRSKTYRRYCWRGAGYRYRC 960
Bhutan-09005        CCRRRKAATAGRTATAYTYGTGTTWCKCACCMGCCWWKYYRRSTWYRGYSWRRWSCRYAY 960
Indonesia-88035     CCRRRKAATAGRTATAYTYGTGTTWCKYRCCMGCCWWKYYRRSTWYRGYSWRRWSCRYAY 960
```

```
Indonesia-88045      CCRRRKAATAGRTATAYTYGTGTTWYKYRCCMGCCWWKYYRRSTWYRGYSWRRWSCRYAY 960
Indonesia-88065      CCRRRKAATAGRTATAYTYGTGTTWYKYRCCMGCCWWKYYRRSTWYRGYSWRRWSCRYAY 960
                       *  *    . :    .*. .    *  .     **   ***. ** *.**  . **

Bhutan-09015         MYWMYMYRRTSGAGCYAGCGAAAWSYGARASGGKGARCYYASWTCRAMYTYWMKGCRGRC 1020
Bhutan-09024         MYWMYMYRRTSGAGCYAGCGAAAWSYGARASGGKGARCYYASWTCRAMYTYWMKGCRGRC 1020
Bhutan-09027         MYWMYMYRRTSGAGCYAGCGAAAWSYGARASGGKGARCYYASWTCRAMYTYWMKGCRGRC 1020
Bhutan-09030         MYWMYMYRRTSGAGCYAGCGAAAWSYGARASGGKGARCYYASWTCRAMYTYWMKGCRGRC 1020
Bhutan-09005         MYACCACAGYCRWRMTRRMSWWRTSCRRAMGRKTAWGMTYCSTKYARCCYCTATRMARRM 1020
Indonesia-88035      MYACCACAGYCRWRMTRRMSWWRTSCRRAMGRKTAWGMTYCSTKYARCCYCTATRMARRM 1020
Indonesia-88045      MYACCACAGYCRWRMTRRMSWWRTSCRRAMGRKTAWGMTYCSTKYARCCYCTATRMARRM 1020
Indonesia-88065      MYACCACAGYCRWRMTRRMSWWRTSCRRAMGRKTAWGMTYCSTKYARCCYCTATRMARRM 1020
                     **        .        .    *     .  ..    *.* .         .    *

Bhutan-09015         ATARAYKYAYYCTGRCYKYKTSWSRATYYATTSMRAYAACYRTTCYMTYCTATRMYKSAR 1080
Bhutan-09024         ATARAYKYAYYCTGRCYKYKTSWSRATYYATTSMRAYAACYRTTCYMTYCTATRMYKSAR 1080
Bhutan-09027         ATARAYKYAYYCTGRCYKYKTSWSRATYYATTSMRAYAACYRTTCYMTYCTATRMYKSAR 1080
Bhutan-09030         ATARAYKYAYYCTGRCYKYKTSWSRATYYATTSMRAYAACYRTTCYMTYCTATRMYKSAR 1080
Bhutan-09005         GYRGGCTTMCYMYRRTYKTKYCWCRGCYTMYWCCRWTTWYYRWYMTMYCSWCYAMCGSTR 1080
Indonesia-88035      GYRGGCTTMCYMYRRTYKTKYCWCRGCYTMYWCCRWTTWYYRWYMTMYCSWCYAMCGSTR 1080
Indonesia-88045      GYRGGCTTMCYMYRRTYKTKYCWCRGCYTMYWCCRWTTWYYRWYMTMYCSWCYAMCGSTR 1080
Indonesia-88065      GYRGGCTTMCYMYRRTYKTKYCWCRGCYTMYWCCRWTTWYYRWYMTMYCSWCYAMCGSTR 1080
                     .   . .   *   * ** * .*.*. *    . *  :  **    *  . .  *  *:*

Bhutan-09015         YCYARYKWKGCRKARWACRRCARWTTYAAYTAYAGYRTTGCTGYTTRARGACKGSGGWRA 1140
Bhutan-09024         YCYARYKWKGCRKARWACRRCARWTTYAAYTAYAGYRTTGCTGYTTRARGACKGSGGWRA 1140
Bhutan-09027         YCYARYKWKGCRKARWACRRCARWTTYAAYTAYAGYRTTGCTGYTTRARGACKGSGGWRA 1140
Bhutan-09030         YCYARYKWKGCRKARWACRRCARWTTYAAYTAYAGYRTTGCTGYTTRARGACKGSGGWRA 1140
Bhutan-09005         TYTWGCSTKSYRKWAWRTAGCGRTYKTRRYWMCRRTRYYRTWRTYYGGGARAGRCRRWRG 1140
Indonesia-88035      TYTWGCCTKSYRKWAWRTAGYGRTYKTRRYWMCRRTRYYRTWRTYYGGGARAGRCRRWAG 1140
Indonesia-88045      TYTWGCSTKSYRKWAWRTAGYGRTYKTRRYWMCRRTRYYRTWRTYYGGGARAGRCRRWAG 1140
Indonesia-88065      TYTWGCSTKSYRKWAWRTAGYGRTYKTRRYWMCRRTRYYRTWRTYYGGGARAGRCRRWRG 1140
                             *. **  *     .*  .   *      *          . . .  .  * .

Bhutan-09015         TCGMSYWRAGGAGGAYYWCYARTRTWRRAYMMWMKWWWARGYTYWARRMWGCKRRYCCTR 1200
Bhutan-09024         TCGMSYWRAGGRGGAYYWCYARTRTWRRAYMMWMKWWWARGYTYWARRMWGCKRRYCYTR 1200
Bhutan-09027         TCGMSYWRAGGRGGAYYWCYARTRTWRRAYMMWMKWWWARGYTYWARRMWGCKRRYCCTR 1200
Bhutan-09030         TCGMSYWRAGGRGGAYYWCYARTRTWRRAYMMWMKWWWARGYTYWARRMWGCKRRYCYTR 1200
Bhutan-09005         CYCASTWAGRRGRCRYCWYYMRYRKWGRWCMMACTWTARGGCWCARAGMAATTGRTTCAG 1200
Indonesia-88035      CYCASTWAGRRGRCRYCWYYMRYRKWGRWCMMACTWTARGACWCARRRMAAYTGRTTCAG 1200
Indonesia-88045      CYCASTWAGRRGRCRYCWYYMGYRKWGRWCMMACTWTARGACWCARRRMAAYTGRTTCAG 1200
Indonesia-88065      CYCASTWAGRRGRCRYCWYYMRYRKWGRWCMMACTWTARGGCWCARAGMAATTGRTTCAG 1200
                         * * .      * * *   *.* *  **  .*    .       * . . *   :

Bhutan-09015         TWYCRAGCWCMRCRYAGCWYATWRRRGRCYWCACAYCARGCYCARGMRRYAYAGMRWAAA 1260
Bhutan-09024         TWYCRAGCWCMRCRYAGCWYATWRRRGRCYWCACAYCARGCYCARGMRRYAYAGMRWAAA 1260
Bhutan-09027         TWYCRAGCWCMRCRYAGCWYATWRRRGRCYWCACAYCARGCYCARGMRRYAYAGMRWAAA 1260
Bhutan-09030         TWYCRAGCWCMRCRYAGCWYATWRRRGRCYWCACAYCARGCYCARGMRRYAYAGMRWAAA 1260
Bhutan-09005         CAYSARATWYARTGYGAYTCRYWGGRRRTCWMMTTYYRGKMYSRRRMRGCACARCGWAAR 1260
Indonesia-88035      CAYSARATWTAATGYGAYTCRYWGGARRTCWMMTTYYRGTAYSRRRMRGCRCRRCGWRRR 1260
Indonesia-88045      CAYSARATWTAATGYGAYTCRYWGGRRRTCWMMTTYYRGTAYSRRRMRGCRCRRCGWRRA 1260
Indonesia-88065      CAYSARATWYARTGYGAYTCRYWGGRRRTCWMMTTYYRGTAYSRRRMRGCACRRCGWAAR 1260
                       *.  . *     *..     *    *  *   :*     *. * **        *
```

```
Bhutan-09015         TTYRTCYCKAKTGTAAKTCRTAGRAMSRRGATKKMATCYSCWCGYCTSKMGRWRTMGRKM 1320
Bhutan-09024         TTYRTCYCKAKTGTAAKTCRTAGRAMSRRGATKKMATCYSCWCGYCTSKMGRWRTMGRKM 1320
Bhutan-09027         TTYRTCYCKAKTGTAAKTCRTAGRAMSRRGATKKMATCYSCWCGYCTSKMGRWRTMGRKM 1320
Bhutan-09030         TTYRTCYCKAKTGTAAKTCRTAGRAMSRRGATKKMATCYSCWCGYCTSKMGRWRTMGRKM 1320
Bhutan-09005         ACYRWYCSGCTWSYRRGYTAYWKATMGGAARGTKAWYYYSMAGSTYCCTCTGTAYCRAGA 1320
Indonesia-88035      ACYRWYCSGCTWSYRRGYTAYWKATMGGAARGTKAWYYYSMAGSTYCCTCTGTAYCRAGA 1320
Indonesia-88045      ACYRWYCSGCTWSYRRGYTAYWKATMGGAARGTKAWYYYSMAGSTYCCTCTGTAYCRAGA 1320
Indonesia-88065      ACYRWYCSGCTWSYRRGYTAYWKATMGGAARGTKAWYYYSMAGSTYCCTCTGTAYCRAGA 1320
                     : **   . .. .           :*.  .  .*    **   .   ..

Bhutan-09015         TTTCTGAAMTGAYWSRYCMYTSRRYYAAMRGCGYRTMTMWGRMMYAAWKAATRRKYYRKT 1380
Bhutan-09024         TTTCTGAAMTGAYWSRYCMYTSRRYYAAMRGCGYRTMTMWGRMMYAAWKAATRRKYYRKT 1380
Bhutan-09027         TTTCTGAAMTGAYWSRYCMYTSRRYYAAMRGCGYRTMTMWGRMMYAAWKAATRRKYYRKT 1380
Bhutan-09030         TTTCTGAAMTGAYWSRYCMYTSRRYYAAMRGCGYRTMTMWGRMMYAAWKAATRRKYYRKT 1380
Bhutan-09005         YKYYGRWTMYRTYWCRTYACYSGGYTRGMAATKTAWAWAWRRCMYRRTGRMWGATCCRTW 1380
Indonesia-88035      YKYYGRWTMYRTYWCRTYACYSGGYTRGMAATKTAWAWAWRRCMYRATGRMWGATCCRTW 1380
Indonesia-88045      YKYYGRWTMYRTYWCRTYACYSGGYTRGMAATKTAWAWAWRRCMYRATGRMWGATCCRTW 1380
Indonesia-88065      YKYYGRWTMYRTYWCRTYACYSGGYTRGMAATKTAWAWAWRRCMYRATGRMWGATCCRTW 1380
                      .     :*  :**.*     *  *  .* .        * * **         .  *.

Bhutan-09015         RWARTTTAKWRRYRWRGYYWYRRWCYRACYGTTCGAARRCKKKMRYRACGCATRRYAKYM 1440
Bhutan-09024         RWARTTTAKWRRYRWRRYYWYRRWCYRACYGTTCGAARRYKKKMRYRACGCATRRYAKYM 1440
Bhutan-09027         RWARTTTAKWRRYRWRRYYWYRRWCYRACYGTTCGAARRYKKKMRYRACGCATRRYAKYM 1440
Bhutan-09030         RWARTTTAKWRRYRWRRYYWYRRWCYRACYGTTCGAARRCKKKMRYRACGCATRRYAKYM 1440
Bhutan-09005         ATWRWGWRTWRRCRAARYYWYRATMYGMYCKYYMKRRGGTKKGMACGRYRGWKARYGKCC 1440
Indonesia-88035      ATWRWGWRTWRRCRAARYYWYRATMYGMTCKYYMKRRGGTKTGMACGRYRGWKARYGKCC 1440
Indonesia-88045      ATWRWGWRTWRRCRAARYYWYRATMYGMTCKYYMKRRGGTKKGMACGRYRGWKARYGKCC 1440
Indonesia-88065      ATWRWGWRTWRRCRAARYYWYRATMYGMTCKYYMKRRGGTKKGMACGRYRGWKARYGKCC 1440
                        *    .*** *   *****   *              *. *        . **.*

Bhutan-09015         ARTRAAMYYYRWGWMYGYYATYRRYRCRYSWRGTRMWCMCKAGRSYCKTRKYTGRWGGAC 1500
Bhutan-09024         ARTRAAMYYYRWGWMYGYYATYRRYRCRYSWRGTRMWCMCKAGRSYCKTRKYTGRWGGAC 1500
Bhutan-09027         ARTRAAMYYYRWGWMYGYYATYRRYRCRYSWRGTRMWCMCKAGRSYCKTRKYTGRWGGAC 1500
Bhutan-09030         ARTRAAMYYYRWGWMYGYYATYRRYRCRYSWRGTRMWCMCKAGRSYCKTRKYTGRWGGAC 1500
Bhutan-09005         RRKGCGMYCYRWSTACRCYMKYRGYGTMCGTGKCRCTYCYTMRGCTYTYRGCKKMARKRA 1500
Indonesia-88035      RRKGCGMYCYRWSTACRCYMKYRGYGTMCGTGKCRCTYCYTMRGCTYTYRGCKKMARKRA 1500
Indonesia-88045      RRKGCGMYCYRWSTACRCYMKYRGYGTMCGTGKCRCTYCYTMRGCTYTYRGCKKMARKRA 1500
Indonesia-88065      RRKGCGMYCYRWSTACRCYMKYRGYGTMCGTGKCRCTYCYTMRGCTYTYRGCKKMARKRA 1500
                      *. ..** ***.     * .** *    .    *     .   .  . *  .      .

Bhutan-09015         TKRRGTCAYSRWRKYCKMYARRWRGRKRAYTYYAMYWRYKYRYYRYTCYYTCTYTGRMMG 1560
Bhutan-09024         TKRRGTCAYSRWRKYCKMYARAWRGRKRAYTYYAMYWRYKYRYYRYTCYYTCTYTGRMMG 1560
Bhutan-09027         TKRRGTCAYSRWRKYCKMYARAWRGRKRAYTYYAMYWRYKYRYYRYTCYYTCTYTGRMMG 1560
Bhutan-09030         TKRRGTCAYSRWRKYCKMYARAWRGRKRAYTYYAMYWRYKYRYYRYTCYYTCTYTGRMMG 1560
Bhutan-09005         WKAARYYMYMAARGYYTMYMRAAGKATRWTATCCCYTRCKYAYTGTWYCTWYKYYKAMCK 1560
Indonesia-88035      WKAARYYMYMAARGYYTMYMRRAGKATRWTATCCCYTRCKYAYTGTWYCTWYKYYKAMCK 1560
Indonesia-88045      WKAARYYMYMAARGYYTMYMRRAGKATRWTATCCCYTRCKYAYTGTWYCTWYKYYKAMCK 1560
Indonesia-88065      WKAARYYMYMAARGYYTMYMRRAGKATRWTATCCCYTRCKYAYTGTWYCTWYKYYKAMCK 1560
                      *      *   * * .** *     .*   :  . * * **  *         .*   *

Bhutan-09015         MARGWWCTTGRCYGTTGATYTYGGWRSSGKACYTWYRRKYCCMGRYRTYGRTTTAATGTA 1620
Bhutan-09024         MARGWWCWTGRCYGTTGATYTYGGWRSSGKACYTWYRRKYCCMGRYRTYGRTTTAATGTA 1620
Bhutan-09027         MARGWWCWTGRCYGTTGATYTYGGWRSCGKACYTWYRRKYCCMGRYRTYGRTTTAATGTA 1620
Bhutan-09030         MARGWWCWTGRCYGTTGATYTYGGWRSSGKACYTWYRRKYCCMGRYRTYGRTTTAATGTA 1620
```

```
Bhutan-09005        CMATWWYTYCAYYRCATRYKYTSKTGSGRGMYTYWTAGKCYYWRGCRYTARCGCGRYAGW 1620
Indonesia-88035     CMATWWYTYCAYCRCATRYGYTSKTGSGRGMYTYWTAGKCYYWRGCRYTARCGCGRYAGW 1620
Indonesia-88045     CMATWWYTYCAYCRCATRTTYTSKTGSGRGMYTYWTAGKCYYWRGCRYTARCGCGRYAGW 1620
Indonesia-88065     CMATWWYTYCAYYRCATRYKYTSKTGSGRGMYTYWTAGKCYYWRGCRYTARCGCGRYAGW 1620
                        **         :      .   *       *   *       *  .*   .  .

Bhutan-09015        RWTCRYWTCYCSWRYYAACRASWYKWTRRYTYATTRAGGAYCRRCYCYRWGYGTGCYMAY 1680
Bhutan-09024        RWTCRYWTCYCSWRYYAACRASWYKWTRRYTYATTRAGGAYCAACYCYRWGYGTGCYMAY 1680
Bhutan-09027        RWTCRYWTCYCSWRYYAACRASWYKWTRRYTYATTRAGGAYCRRCYCYRTGYGTGCYMAY 1680
Bhutan-09030        RWTCRYWTCYCSWRYYAACRASWYKWTRRYTYATTRAGGAYCAACYCYRWGYGTGCYMAY 1680
Bhutan-09005        ATYYGTWWSCTSWACYRRMAWGWCKAYRGTKYRAGARKRWCYAAACTTGAKYAYKYYMMY 1680
Indonesia-88035     ATYYGTWWSCTSWACTGGMAWGWCKAYRGTKYRAGARKRWCYAAACTTGAKYAYKYYMMY 1680
Indonesia-88045     ATYYGTWWSCTSWACYRRMAWGWCKAYRGTKYRAGARKRWCYAAACTTGAKYATGYYMMY 1680
Indonesia-88065     ATYYGTWWSCTSWACYRRMAWGWCKAYRGTKYRAGARKRWCYAAACTTGAKYAYKYYMMY 1680
                          * .  **        .* *  *  .* :          .      *.   ** *

Bhutan-09015        MRRRWRAKRWYTYMCTTGAWWYSCYCATYARYARYRRYAYKRGRAWYGAMGYRKTCWYAG 1740
Bhutan-09024        MRRRWRAKRWYTYMCTTGAWWYSCYCATYARYARYRRYACTRGRAWYGAMGYRKTCWYAG 1740
Bhutan-09027        MRRRWRAKRWYTYMCTTGATATSCYCATYARYARYRRYAYKRGRAWYGAMGYRKTCWYAG 1740
Bhutan-09030        MRRRWRAKRWYTYMCTTGAWWYSCYCATYARYARYRRYACTRGRAWYGAMGYRKTCWYAG 1740
Bhutan-09005        CGAGTRRKRACYYAAWYRRATCCYTMWYCWRCWACGRYGYKAKARTCKGMRCGGCTATRS 1740
Indonesia-88035     CGAGTRRKRACYYAAWYRRATCCYTMWYCWRCWACGRYGCTAKARTCKGMRCGGCTATRS 1740
Indonesia-88045     CGAGTRRKRACYYAAWYRRATCCYTMWYCWRCWACGRYGCTAKARTCKGMRCGGCTATRS 1740
Indonesia-88065     CGAGTRRKRACYYAAWYRRATCCYTMWYCWRCWACGRYGCTAKARTCKGMRCGGCTATRS 1740
                         * **   * .       .       *     **. .       .*         .

Bhutan-09015        GYKTWCRWYAWGTWWRACYTATRSTCRKYASRKRYTRAACRGGMRYTWAMSRTGTTTTMA 1800
Bhutan-09024        GYKTWCRWYAWGTWWRACYTATRSTCRKYASRKRYTRAACRGGMRYTWAMSRTGTTTTMA 1800
Bhutan-09027        GYKTWCRWYAWGTWWRACYTATRSTCRKYASRKRYTRAACRGGMRYTWAMSRTGTTTTMA 1800
Bhutan-09030        GYKTWCRWYAWGTWWRACYTATRSTCRKYASRKRYTRAACRGGMRYTWAMSRTGTTTTMA 1800
Bhutan-09005        KCKWTTGWCRTRCTWRMYTKRKAGYYAGYWSATAYYRTCARRRMGYYWRCSAYRYKYYMC 1800
Indonesia-88035     KCKWTTGWCRTRCTWRMYTKRKAGYYAGYWSATAYYRTCARRRMGYYWRCSAYRYKYYMC 1800
Indonesia-88045     KCKWTTGWCRTRCTWRMYTKRKAGYYAGYWSATAYYRTCARRRMGYYWRCSAYRYKYYMC 1800
Indonesia-88065     KCKWTTGWCRTRCTWRMYTKRKAGYYAGYWSATAYYRTCARRRMGYYWRCSAYRYKYYMC 1800
                      *    *      **   . . .    * * . * *:..*  * * *  *    .  *.

Bhutan-09015        YSRATCATTTKCTAAATKKTRAYKYYTKCTRRMMWAASRRGCYTWKYAYGGGTRTTKKYK 1860
Bhutan-09024        YSRATCATTTKCTAAATKKTRAYKYYTKCTRRMMWAASRRGCYTWKYAYGGGTRTTKKYK 1860
Bhutan-09027        YSRATCATTTKCTAAATKKTRAYKYYTKCTRRMMWAASRRGCYYWKYAYGGGTRTTKKYK 1860
Bhutan-09030        YSRATCATTTKCTAAATKKTRAYKYYTKCTRRMMWAASRRGCYYWKYAYGGGTRTTKKYK 1860
Bhutan-09005        TGAMGYRYYWKMCRRGWKKYRRYGCSYGTWRRMATGRSRRAYYYWKTWCRRKYGKKGKTG 1860
Indonesia-88035     TGAMGYRYYWKMCRRGWKKYRRYGCSYGTWRRMATGASRRAYYTWKTWCRRKYGKKGKTG 1860
Indonesia-88045     TGAMGYRYYWKMCRRGWKKYRRYGCSYGTWRRMATGASGRAYYYWKTWCRRKYGKKGKTG 1860
Indonesia-88065     TGAMGYRYYWKMCRRGWKKYRRYGCSYGTWRRMATGRSRRAYYYWKTWCRRKYGKKGKTG 1860
                     .        *    . ** * *       ***  . * *. * **        .. *

Bhutan-09015        YRTRYWTGTAGTCYAGTWSWRTYKCYCTTWGKRYYSYAAYYGGACYYRRTYCAGCATCYK 1920
Bhutan-09024        YRTRYWTGTAGTCYAGTWSWRTYKCYCTTWGKRYYSYAAYYGGACYYRRTYCAGCATCYK 1920
Bhutan-09027        YRTRYWTGTAGTCYAGTWSWRTYKCYCTTWGKRYYSYAAYYGGACYYRRTYCAGCATCYK 1920
Bhutan-09030        YRTRYWTGTAGTCYAGTWSWRTYKCYCTTWGKRYYSYAAYYGGACYYRRTYCAGCATCYK 1920
Bhutan-09005        CAYRCWCTAGRYYYCTYTSWGGTGYYSKYTAKAYTCTRGYYKRRYYCGGYYYRRYRWYYK 1920
Indonesia-88035     CAYRCWCTAGRYYYCTYTSWGGTGYYCKYTAKAYTCTRGYYKRRYYCGGTYYRRYRWYYK 1920
Indonesia-88045     CAYRCWCTAGRYYYCTYTSWGGTGYYSKYTAKAYTCTRGYYKRRYYCGGTYYRRYRWYYK 1920
Indonesia-88065     CAYRCWCTAGRYYYCTYTSWGGTGYYSKYTAKAYTCTGGYYKRRYYCGGTYYRRYRWYYK 1920
```

```
                       * *  :.   *.   **     *..  .* * .  .**    *    *       **

Bhutan-09015           GGGRSWKAAAARGATTAYWYRTGRAKAMMRWTGCTRRSARYKCSAYWGGTRSCGWAYATY 1980
Bhutan-09024           GGGRSWKAAAARGATTAYWYRTGRAKAMMRWTGCTRRSARYKCSAYWGGTRSCGWAYATY 1980
Bhutan-09027           GGGRSWKAAAARGATTAYWYRTGRAKAMMRWTGCTRRSARYKCSAYWGGTRSCGWAYATY 1980
Bhutan-09030           GGGRSWKAAAARGATTAYWYRTGRAKAMMRWTGCTRRSARYKCSAYWGGTRSCGWAYATY 1980
Bhutan-09005           KSRGCAGRGCRARRYYRTTYAYAGMKMAMRWYRYKRRSRRCKYCMYWSKKRSYKWWTWYT 1980
Indonesia-88035        KSRGCAGRGCRARRYYRTTYAYAGMKMAMRWYRYKRRSRRCKYCMYWSKKRSYKWWTWYT 1980
Indonesia-88045        KSRGCAGRGCRARRYYGTTYAYAGMKMAMRWYRYKRRSRRCKYCMYWSKKRSYKWWTWYT 1980
Indonesia-88065        KSRGCAGRGCRARRYYRTTYAYAGMKMAMRWYRYKRRSRRCKYCMYWSKKRSYKWWTWYT 1980
                        .  .   ..         *  .  *  ***   .*** * * . **. .**  *

Bhutan-09015           ATYAYGSWGATRRCGSWMKMYGGYYSAYYKGRRGRCRYSRTMYCRKATARATCWTYRCAY 2040
Bhutan-09024           ATYAYGSWGATRRCGSWMKMYGGYYSAYYKGRRGRCRYSRTMYCRKATARATCWTYRCAY 2040
Bhutan-09027           ATYAYGSWGATRRCGSWMKMYGGYYSAYYKGRRGRCRYSRTMYCRKATARATCWTYRCAY 2040
Bhutan-09030           ATYAYGSWGATRRCGSWMKMYGGYYSAYYKGRRGRCRYSRTMYCRKATARATCWTYRCAY 2040
Bhutan-09005           WCCRCRCATRWRAYACAAKACRRCYSRYYTRRRGGSRCCGCMTYGTGWWGMWSWGYRSRT 2040
Indonesia-88035        WCCRCRCATRWRAYACAAKACRRCYSRYYTRRRRGSRCCGCMTYGTGWWGMWSWGYRSRT 2040
Indonesia-88045        WCCRCRCATRWRAYACAAKACRRCYSRYYTRRRRGSRCCGCMTYGTGWWGMWSWGYRSRT 2040
Indonesia-88065        WCCRCRCATRWRAYACAAKACRRCYSRYYTRRRRGSRCCGCMTYGTGWWGMWSWGYRSRT 2040
                             .    *  ..  *     ** **. **  .* .  *   ..     .* **.

Bhutan-09015           WYAKAYGYKMKASWKASAYRGCRGMWWWWYRTRGWSRRRARSYYTYGWRTAYKRMGRTAR 2100
Bhutan-09024           WYAKAYGYKMKASWKASAYRGCRGMWWWWYRTRGWSRRRARSYYTYGWRTAYKRMGRTAR 2100
Bhutan-09027           WYAKAYGYKMKASWKASAYRGCRGMWWWWYRTRGWCARRARSYYTYGWRTAYKRMGRTAR 2100
Bhutan-09030           WYAKAYGYKMKASWKASAYRGCRGMWWWWYRTRGWCARRARSYYTYGWRTAYKRMGRTAR 2100
Bhutan-09005           WCRGMTSTTAKWCTGMCRYRRSAKCAWWAYRYASACARAMRGYTWYSWAKWCTGMRACWA 2100
Indonesia-88035        WCRGMTSTTAKWCTGMCRYRRSAKCAWWAYRYASACARAMAGYTWYSWAKWCTGMRACWA 2100
Indonesia-88045        WCRGMTSTTAKWCTGMCRYRRSAKCAWWAYRYASACARAMRGYTWYSWAKWCTGMRACWA 2100
Indonesia-88065        WCRGMTSTTAKWCTGMCRYRRSAKCAWWAYRYASACARAMRGYTWYSWAKWCTGMRACWA 2100
                       *     . . * .   . ** .    ** **  . . *   .*  *.* .  . *

Bhutan-09015           SRRAATCRYCTTTARRYATCACMYYTYWRTRMRMRWKYRCRGKKGRYWAGTRYAYCTAGT 2160
Bhutan-09024           SRRAATCRYCTTTARRYATCACMYYTYWRTRMRMRWKYRCRGKKGRYWAGTRYAYCTAGT 2160
Bhutan-09027           SRRAATCRYCTTTARRYATCACMYYTYWRTRMRMRWKYRCRGKKGRYWAGTRYAYCTAGT 2160
Bhutan-09030           SRRAATCRYCTTTARRYATCACMYYTYWRTRMRMRWKYRCRGKKGRYWAGTRYAYCTAGT 2160
Bhutan-09005           SAGGTYYRYYYCCRGRTRYYRYATYKCWAYGMACRAKTAYRSTGAGYWARAATWTYYMRY 2160
Indonesia-88035        SAGGTYYRYYYCCRGRTRYYRYATYKCWAYGMACRAKTAYRSTGRGYWRRTATWTYYMGY 2160
Indonesia-88045        SAGGTYYRYYYCCRGRTRYYRYATYKCWAYGMACRAKTAYRSTGAGYWRRTATWTYYMGY 2160
Indonesia-88065        SAGGTYYRYYYCCRGRTRYYRYATYKCWAYGMACRAKTAYRSTGRGYWARAATWTYYMRY 2160
                       *  .:  **      *        *. *   *  * *   *..   **  :

Bhutan-09015           KATCAYTRACRCGGSYCTWWAGSGYWRAWYATATGGRCCCAYGGTYSYMMRSRAKSCACC 2220
Bhutan-09024           KATCAYTRACRCGGSYCTWWAGSGYWRAWYATATGGRCCCAYGGTYSYMMRSRAKSCACC 2220
Bhutan-09027           KATCAYTRACRCGGSYCTWWAGSGYWRAWYATATGGRCCCAYGGTYSYMMRSRAKSCACC 2220
Bhutan-09030           KATCAYTRACRCGGSYCTWWAGSGYWRAWYATATGGRCCCAYGGTYSYMMRSRAKSCACC 2220
Bhutan-09005           GAYGGCYRWMRTRKSYYYWWWASKTWRRATWTRTKRGSYYGTSGKCCCAAGGGTKGARSM 2220
Indonesia-88035        KMTGGCYRWMRTRKSYYYWWWASKTWRRATWKRYKGGSYYGTSKKCGCAAGGGTKGARSM 2220
Indonesia-88045        KMTGGCYRWMRTRKSYYYWWWASKTWRRATWKRYKGGSYYGTSKKCGCAAGGGTKGARSM 2220
Indonesia-88065        GAYGGCYRWMRTRKSYYYWWWASKTWRRATWTRTKRGSYYGTSGKCCCAAGGGTKGARSM 2220
                           .  *  *   **  ** .*  **    .     .  . . .      . :*.. .

Bhutan-09015           TYTYKTKTRTRTAKYAAYWYMGKMTAMCYGACTCWRYMGRAKCACYYGAGGTRYRRTAYY 2280
Bhutan-09024           TYTYKTKTRTRTAKYAAYWYMGKMTAMCYGACTCWRYMGRAKCACYYGAGGTRYRRTAYY 2280
```

```
Bhutan-09027         TYTYKTKTRTRTAKYAAYWYMGKMTACCYGACTCWRYMGRAKCACYYGAGGTRYRRTAYY 2280
Bhutan-09030         TYTYKTKTRTRTAKYAAYWYMGKMTAMCYGACTCWRYMGRAKCACYYGAGGYRYRRTAYY 2280
Bhutan-09005         YYKYGAGYRYAYRTTTMYWYCKTCARCMYRRYTYAGCASAMGMMTYYRRRRTACAAGGTC 2280
Indonesia-88035      YYKYGAGYRYRYRTTTMYWYCKTCARCMYRRYYYAGCASAMGMMTYYRRRRTACAAGGTC 2280
Indonesia-88045      YYKYGAGYRYRYRTTTMYWYCKTCARCMYRRYCTAGCASAMGMMTYYAGARTACAAGGTC 2280
Indonesia-88065      YYKYGAGYRYAYRTTTMYWYCKTCARCMYRRYYYAGCASAMGMMTYYRRRRYACAAGGTC 2280
                      *.* :  *    . : ***  . :   *         .      **          .

Bhutan-09015         YTTRWCTGYYKCCYYYYYCGAYWAGYAWRGATGYAYAGSTTTYTYRTRWKMTGSKCKGRR 2340
Bhutan-09024         YTTRWCTGYYKCCYYYYYCGAYWAGYAWRGATGYAYAGSTTTYTYRTRWKMTGSKCKGRR 2340
Bhutan-09027         YTTRWCTGYYKCCYYYYYCGAYWAGYAWRGATGYAYAGSTTTYTYRTRWKMTGSKCKGRR 2340
Bhutan-09030         YTTRWCTGYYKCCYYYYYCGAYWAGYAWRGATGYAYAGSTTTYTYRTRWKMTGSKCKGRR 2340
Bhutan-09005         TWYATSWRCTGMMYCTTYYRGYARKTWAARWCRCRYTCCYYYTWCGTGATMYGGGCGRAA 2340
Indonesia-88035      TWYATSWACTKMMYCTTYYAATARKTWARRTCRCRYTCSYYTTWCGWGATMYRGGYGRAA 2340
Indonesia-88045      TWYATSWACTKMMYCTTYYAATARKTWARRTCRCRYTCSYYTTWCGWGATMYRGGYGRAA 2340
Indonesia-88065      TWYATSWACTGMMYCTTYYRGYARKTWAARWCRCRYTCCYYYTWCGTGATMYGGGCGRAA 2340
                          .       *   *  .              *: .          .*  .

Bhutan-09015         GYATSRYYTYYYSYGYRRGYRRKRCGWATYYYRKSGRWGGTRMTYSWTATMMAYCCTRYYT 2400
Bhutan-09024         GYATSRYYTYYYSYGYRRGYRRKRCGWATYYYRKSGRWGGTRMTYSWTATMMAYCCTRYYT 2400
Bhutan-09027         GYATSRYYTYYYSYGYRRGYRRKRCGWATYYYRKSGRWGGTRMTYSWTATMMAYCCTRYYT 2400
Bhutan-09030         GYATSRYYTYYYSYGYRRGYRRKRCGWATYYYRKSGRWGGTRMTYSWTATMMAYCCTRYYT 2400
Bhutan-09005         KCRTGGTYYTCSCATRAKYAGTAYKARYTTCRRCKAWTRARACCGTYWYACGTMYYGCTY 2400
Indonesia-88035      KCRYGGTYYTCSCATRAKYAGTAYKARYTTCRRCKAWTRARACCGTYWYACGTATYGCTY 2400
Indonesia-88045      KCRYGGTYYTCSCATRAKYAGTAYKARYTTCAGCKAWTAARACCGTYWYACGTATYGCTY 2400
Indonesia-88065      KCRTGGTYYTCSCATRAKYAGTAYKARYTTCRRCKAWTAARACCGTYWYACGTATYGCTY 2400
                         .  *   * . *  *  .           .  *  :*   .      .

Bhutan-09015         AGTGCRKRARARMYCTTMAARAYYCSTTTRGMYAWATRGRYYYRWRYGMCYRWRYGWSCC 2460
Bhutan-09024         AGTGCRKRARARMYCTTMAARAYYCSTTTRGMYAWATRGRYYYRWRYGMCYRWRYGWSCC 2460
Bhutan-09027         AGTGCRKRARARMYCTTMAARAYYCSTTTRGMYAWATRGRYYYRWRYGMCYRWRYGWSCC 2460
Bhutan-09030         AGTGCRKRARARMYCTTMAARAYYCSTTTRGMYAWATRGRYYYRWRYGMCYRWRYGWSCC 2460
Bhutan-09005         WRYKYGKRRRMGCCTAYARMAGTTYCTYYAKCCMARWRRRYTCGAAYAAYTRAGTKAGYY 2460
Indonesia-88035      WRYGYGKRRRMGCCTATARMAGTTYCGYCAKCCMARWGRRYTCGAAYAAYCRAGTKAGYY 2460
Indonesia-88045      WRYGYGKRRRMGCCTATARMAGTTYCGYCAKCCMARWGRRYTCGAAYAAYCRAGTKAGYY 2460
Indonesia-88065      WRYKYGKRRRMGCCTAYARMAGTTYCTYYAKCCMARWRRRYTCGAAYAAYTRAGTKAGYY 2460
                           ** *     :     .   .             **     *.   *     .

Bhutan-09015         CAYRWMRRKYSKTRTSTSCCCTAYAYAKRATRYAGRRGKRATTYAARRYYTCWTAYYTGW 2520
Bhutan-09024         CAYRWMRRKYSKTRTSTSCCCTAYAYAKRATRYAGRRGKRATTYAARRYYTCWTAYYTGW 2520
Bhutan-09027         CAYRWMRRKYSKTRTSTSCCCTAYAYAKRATRYAGRRGKRATTYAARRYYTCWTAYYTGW 2520
Bhutan-09030         CAYRWMRRKYSKTRTSTSCCCTAYAYAKRATRYAGRRGKRATTYAARRYYTCWTAYYTGW 2520
Bhutan-09005         MMCGACGRKYCTYGCGCCYYYTAYRCRKGRYACRGAGATARACTGRAAYTCMTCRTYKSW 2520
Indonesia-88035      MCCGACGRKYCTYGYGTCYYYKMYRCRKGRTACRCAGATARACTGAAAYTCCTCRTYKSW 2520
Indonesia-88045      MMCGACGRKYCTYGYGTCYYYKMYRCRKGRTACRAAGATARACTGAAAYTCCTCRTYKSW 2520
Indonesia-88065      MMCGACGRKYCTYGCGCCYYYTAYRCRKGRYACAAAGATARACTGRAAYTCMTCRTYKSW 2520
                            ***..   . .   . *   *         ..  :  .   *       *..*

Bhutan-09015         YGRYGMYTMWRGRWMYATMTTYAAAYRRAMRRTKWGCRTYAYCGYGCRRTTKTRKYKAWK 2580
Bhutan-09024         YGRYGMYTMWRGRWMYATMTTYAAAYRRAMRRTKWGCRTYAYCGYGCRRTTKTRKYKAWK 2580
Bhutan-09027         YGRYGMYTMWRGRWMYATMTKYAAAYRRAMRRTKWGCRTYAYCGYGCRRTTKTRKYKAWK 2580
Bhutan-09030         YGRYGMYTMWRGRWMYATMTTYAAAYRRAMRRTKWGCRTYAYCGYGCRRTTKTRKYKAWK 2580
Bhutan-09005         YRGYSMYCAWAKGTCYRYCYTCMGGCAARARAWGWGCRYCACMAYTYAAYATYAGTGATT 2580
Indonesia-88035      YRGYSMYCAWAKGTCYRYCYTCAGGYAARARAWGWRSRYCGCMAYTYAAYATTAGTGWTT 2580
```

```
Indonesia-88045      YRGYSMYCAWAKGTCYRYCYTCAGGYAARARAWGWRSRYCGCMAYTYAAYATTAGTGWTT 2580
Indonesia-88065      YRGYSMYCAWAKGTCYRYCYTCMGGCAARARAWGWGCRYCACCAYTYAAYATYAGTGATT 2580
                     *  *.**  *     *    .  ..     *   *.*   .  .*     :.       .

Bhutan-09015         RRWTYRGCTAAMCCCMWYRTAYCGYRKTMTTGGSYCAMMGCCTWMSWCWRAYGGRTSRSR 2640
Bhutan-09024         RRWTYRGCTAAMCCCMWYRTAYCGYRKTMTTGGSYCAMMGCCTWMSWCWRAYGGRTSRSR 2640
Bhutan-09027         RRWTYRGCTAAMCCCMWYRTAYCGYRKTMTTGGSYCAMMGCCTWMSWCWRAYGGRTSRSR 2640
Bhutan-09030         RRWTYRGCTAAMCCCMWYRTAYCGYRKTMTTGGSYCAMMGCCTWMSWCWRAYGGRTSRSR 2640
Bhutan-09005         AGTWCGSSCRGCYSSMWYRYRCCSTAGCCYYKKCYTAACRSYYAACAMTRRTTRGGCRGR 2640
Indonesia-88035      AGTTCGSSYAGCYSSMWYRYRCYSTAGCCYYKKCYTRACRSYYAACAMTRRTTRGGCRGR 2640
Indonesia-88045      AGTTCGSSYAGCYSSMWYGYRCYSTAGCCYYKKCYTRACRSYYAACAMTRRTTGGGCRGR 2640
Indonesia-88065      AGTWCGSSCRGCYSSMWYRYRCCSTAGCCYYKKCYTAACRSYYAACAMTRRTTRGGCRGR 2640
                           ..  .  ..***     .         .*     .    .   *      .*.*

Bhutan-09015         ASWCYKTCATTYGWSRGCRARMSYYTMRYTRAYWTTYYTSYATYYGSYTTGAARYRTCCM 2700
Bhutan-09024         ASWCYKTCATTYGWSRGCRARMSYYTMRYTRAYWTTYYTSYATYYGSYTTGAARYRTCCM 2700
Bhutan-09027         ASWCYKTCATTYGWSRGCRARMSYYTMRYTRAYWTTYYTSYATYYGSYTTGAARYRTCCM 2700
Bhutan-09030         ASWCYKTCATTYGWSRGCRARMSYYTMRYTRAYWTTYYTSYATYYGSYTTGAARYRTCCM 2700
Bhutan-09005         RCTMTKGTGGGYGTCRRYRAAASTTCCRYYRRTTAGCTYCCMTCTKCCWWRRTRTAYMYA 2700
Indonesia-88035      ACTCTGGTGGTTKTCRRYRRAASTTCCRCYRRCTAGCTYCCMKCTKCCAARRTRTACAYA 2700
Indonesia-88045      ACTCTGGTGGTTKTCRRYRRAASTTCCRCYRRCTAGCTYCCMKCTKCCAARRTRTACAYA 2700
Indonesia-88065      RCTMTKGTGGGYGTCRRYRAAASTTCCRYYRRTTAGCTYCCMTCTKCCWWRRTRTAYMYA 2700
                      .      .     .*  *   *    *  *   :    .  .   .     :*

Bhutan-09015         RGYRTRCYTCWTRCRAGSGCTRRAAGYTAWYACWWYKYAGYTCYCYWYWYYACRCACRKT 2760
Bhutan-09024         RGYRTRCYTCWTRCRAGSGCTRRAAGYTAWYACWWYKYAGYTCYCYWYWYYACRCACRKT 2760
Bhutan-09027         RGYRTRCYTCWTRCRAGSGCTRRAAGYTAWYACWWYKYAGYTCYCYWYWYYACRCACRKT 2760
Bhutan-09030         RGYRTRCYTCWTRCRAGSGCTRRAAGYTAWYACWWYKYAGYTCYCYWYWYYACRCACRKT 2760
Bhutan-09005         ASYAGAYTAYAAAYRRCGKYYAARWRTKRWTCCAWTGTWRTYYCTYWTWYYRYRYRTGGA 2760
Indonesia-88035      ASYAKACTAYAAAYRRCGKYYAARARTKRWTCTAWTGTAGTTCCTYWYWYYRYRYRTGGA 2760
Indonesia-88045      ASYAKAYTAYAAAYRRCGKYYAARARTKRWTCTAWTGTAGTTCCTYWYWYYRYRYRTGGA 2760
Indonesia-88065      ASYAGACTAYAAAYRRCGKYYAARWRTKRWTCCAWTGTWRTYYCTYWTWYYRYRYRTGGA 2760
                      .*     :  :  *  .         . * .  *          ** ***  *     :

Bhutan-09015         WAAYTAYATKCTRMGSKASMTAAMCGYKTGTAAYYMKYYYRCTWWTCTYYAKYAACRTSY 2820
Bhutan-09024         WAAYTAYATKCTRMGSKASMTAAMCGYKTGTAAYYMKYYYRCTWWTCTYYAKYAACRTSY 2820
Bhutan-09027         WAAYTAYATKCTRMGSKASMTAAMCGYKTGTAAYYMKYYYRCTWWTCTYYAKYAACRTSY 2820
Bhutan-09030         WAAYTAYATKCTRMGSKASMTAAMCGYKTGTAAYYMKYYYRCTWWTCTYYAKYAACRTSY 2820
Bhutan-09005         ARGYYRCAYTYTRCRCKASACTGASACKCRYRRCTAGYYYRTTTTYMCCCRKTRRYGCGT 2820
Indonesia-88035      ARGYYRCRYTYYRCRCGMSACTGMSACKCRYRRCTAGYYYRTYTTYMCCCRKTRRYGCGT 2820
Indonesia-88045      ARGYYRCRYTYYRCRCGMSACTGMSACTCRYRRCTAGYYYRTYTTYMCCCRKTRRYGCGT 2820
Indonesia-88065      ARGYYRCAYTYTRCRCKASACTGASACKCRYRRCTAGYYYRTTTTYMCCCRKTRRYGCGT 2820
                       .*     .  *  .  *  :. .. .         ****          *      .

Bhutan-09015         RSYWTRYRKRMWWATAGTRRYRTCTKKYSTTYYYAYGYTARTMYCTSYCYYCCAARTCRK 2880
Bhutan-09024         RSYWTGYRKRMWWATAGTRRYRTCTKKYSTTYYYAYGYTARTMYCTSYCYYCCAARTCRK 2880
Bhutan-09027         RSYWTRYRKRMWWATAGTRRYRTCTKKYSTTYCYAYGYTARTMYCTSYCYYCCAARTCRK 2880
Bhutan-09030         RSYWTGYRKRMWWATAGTRRYRTCTKKYSTTYYYAYGYTARTMYCTSYCYYCCAARTCRK 2880
Bhutan-09005         GCTAAGYRGRMWTRYRRYAGTGWYYKGCSGKTCYWYRYYCGCCCMYSTACCMYGTRYYGK 2880
Indonesia-88035      GCTAAGYRGRMWTRYRRYAGTGWYYKGCSGKTCYWYRYYCGCCCMYSTACCMYGTRYYGK 2880
Indonesia-88045      GCTAAGYRGRMWTRYRRYAGTGWYYKGCSGKTCYWYRYYCGCCCMYSTACCMYGTRYYGK 2880
Indonesia-88065      GCTAAGYRGRMWTRYRRYAGTGWYYKGCSGKTCYWYRYYCGCCCMYSTACCMYGTRYYGK 2880
                      .  : ** ***             *  * .  * * * .      * .    .:*   *
```

```
Bhutan-09015        GAGTRTKYGTAYMGYTAYCWCGGGYYRKTKYGAARAMYRCCYRARGRWGGMYWRMWTRKR 2940
Bhutan-09024        GAGTRTKYGTAYMGYTAYCWCGGGYYRKTTYGAARAMYRCCTAARGRWGGMYWRMWTRKR 2940
Bhutan-09027        GAGTRTKYGTAYMGYTAYCWCGGGYYRKTKYGAARAMYRCCYRARGRWGGMYWRMWTRKR 2940
Bhutan-09030        GAGTRTKYGTAYMGYTAYCWCGGGYYRKTKYGAARAMYRCCYRARGRWGGMYWRMWTRKR 2940
Bhutan-09005        KWRTAKGTRWRCARYTWYMTMRSRTTGKKTTRMRACCCRYSCARGKGWRSMCARMTKGTA 2940
Indonesia-88035     KAGTATGTGTACAGCTATCTCGGGTTGTTTTGARACCCGCCCAAGGGTAGCCAGATKGTA 2940
Indonesia-88045     KAGKATGTGTACAGCTATCTCGGGTTGTTTTGARACCCGCCCAAGGGTAGCCAGATKGTA 2940
Indonesia-88065     KWRTAKGTRWRCARYYWYMTMRSRTTGKKTTRMRACMYRYSCARGKGWRSMCARMTKGTA 2940
                       . .                .    ...     .    .        .      . .

Bhutan-09015        TTWRMSAYMSYRASYRKYCCYCYRKWYMKAGWRWCTGGWYSWMWYRRSWRACYAYCWRAY 3000
Bhutan-09024        TTWRMSAYMSYRASYRKYCCYCYRKWYMKAGWRWCTGGWYSWMWYRRSWRACYAYCWRAY 3000
Bhutan-09027        TTWRMSAYMSYRASYRKYCCYCYRKWYMKAGWRWCTGGWYSWMWYRRSWGACYAYCWRAY 3000
Bhutan-09030        TTWRMSAYMSYRASYRKYCCYCYRKWYMKAGWRWCTGGWYSWMWYRRSWGACYAYCWRAY 3000
Bhutan-09005        YYARMMRYCCCAMSYRKCYYTMTGKATCKTRWAMYYRRAYCTCWYRRCAARSTRCYTGRC 3000
Indonesia-88035     TTAAACGTCCCAAGCGGCTTTCTGTATCTTATAACTGGATCTCTYAGCAAACTACTTGAC 3000
Indonesia-88045     TTAAACGTCCCAAGCGGCTTTCTGTATCTTATAACTGGATCTCTYAGCAAACTACTTGAC 3000
Indonesia-88065     YYARMAAYCCCAMSYRKCYYTMTGKATCKTRWAMYYRRAYCTCWYRRCAARSTRCYTGRC 3000
                             .   .          .   .:          .   *  .   .

Bhutan-09015        YMGYYTYTGRRRKCCTGAGSRKKYKTRWRMKWSAMYRRACSMYMRRSATCMTRWRKWGWY 3060
Bhutan-09024        YMGYYTYTGRRRKCCTGAGSRKKYKTRWRMKWSAMYAGACSMYMRRSATCMTRWRKWGWY 3060
Bhutan-09027        YMGYYTYTGRRRKCCTGAGSRKKYKTRWRMKWSAMYRRACSMYMRRSATCMTRWRKWGWY 3060
Bhutan-09030        YMGYYTYTGRRRKCCTGAGSRKKYKTRWRMKWSAMYRRACSMYMRRSATCMTRWRKWGWY 3060
Bhutan-09005        YAKCTYCCRARASMMWKWRSRKGYKWGWRMTWSAMYARMGCMTMRRSRGYAARTGGARWT 3060
Indonesia-88035     CAGCTTCCGAAAGCCTGAGGGTGCKTGWSCTTCWACAGAGCCTCGGGGGCAAATGGARTT 3060
Indonesia-88045     CAGCTTCCGAAAGCCTGAGGGTGCKTGWSCTTCWACAGAGCCTCGGGGGCAAATGGARTT 3060
Indonesia-88065     YAKCTYCCRARASMMWKWRSRKGYKWGWRMTWSAMYARMGCMTMRRSRGYAARTGGARWT 3060
                                       . .  *  *  . .       .     .    :

Bhutan-09015        TAYTYRWWYGRTAAKAGRRYTYRRKARAMCAGRRKKRRGSRYWKRCMYTCKAAAYCKCWM 3120
Bhutan-09024        TAYTYRWWYGRTAAKAGRRYTYRRKARAMCAGRRKKRRGSRYWKRCMYTCKAAAYCKCWM 3120
Bhutan-09027        TAYTYRWWYGRTAAKAGRRYTYRRKARAMCAGRRKKRRGSRYWKRCMYTCKAAAYCKCWM 3120
Bhutan-09030        TAYTYRWWYGRTAAKAGRRYTYRRKARAMCAGRRKKRRGSRYWKRCMYTCKAAAYCKCWM 3120
Bhutan-09005        CMYYTGWAYARYMAKMRRGCGTGATWRAMYASAATTARRCGTTTGMAYCCKWARCMTYTA 3120
Indonesia-88035     YATTYGAACAGTAWGARAGCTCGATWAWACRGAATTAAGCGTTTGCACTYKAMACATCTA 3120
Indonesia-88045     YATTYGAACAGTAWGARAGCTCGATWAWACRGAATTAAGCGTTTGCACTYKAMACATCTA 3120
Indonesia-88065     CMYYTGWAYARYMAKMRRGCGTGATWRAMYASAATTARRCGTTTGMAYYCKWARCMTYTA 3120
                             .              .      .  ..   .   .      *     .

Bhutan-09015        KGCYYYGYCGWRAGKCYTGGAKKCTRRRWRKCMRMGRTTASWYYGTYMARTWYYYWMWYY 3180
Bhutan-09024        KGCYYYGYCGWRAGKCYTGGAKKCTRRRWRKCMRMGRTTASWYYGTYMARTWYYYWMWYY 3180
Bhutan-09027        KGCYYYGYCGWRAGKCYTGGAKKCTRRRWRKCMRMGRTTASWYYGTYMARTWYYYWMWYY 3180
Bhutan-09030        KGCYYYGYCGWRAGKCYTGGAKKCTRRRWRKCMRMGRTTASWYYGTYMARTWYYYWMWYY 3180
Bhutan-09005        GRYYYYRCCRWARRKMTWKRRKGMWRRGAAGYMGARRKYRSACYSYYMMRYTYTYTWAYC 3180
Indonesia-88035     GGCCTTGCYRTAAGTCTTGGAGGCTAGGAAGTAGAGGTTACACTGTCCAATTCTTTAACC 3180
Indonesia-88045     GGCCTTGCYRTAAGTCTTGGAGGCTAGGAAGTAGAGGTTACACTGTCCAATTCTTTAACC 3180
Indonesia-88065     GRYYYYRCCRWARRKMTWKRRKGMWRRGAAGYMGARRKYRSACYSYYMMRYTYTYTWAYC 3180
                                  .                      .  .   .

Bhutan-09015        YMCWYTWWAWSKSRWMGWRAWYWYCRCCGRCRYYYYMMTATRYRGRYSRTMYGRKTRTRY 3240
Bhutan-09024        YMCWYTWWAWSKSRWMGWRAWYWYCRCCGRCRYYYYMMTATRYRGRYSRTMYGRKTRTRY 3240
Bhutan-09027        YMCWYTWWAWSKSRWMGWRAWYWYCGMCGRCRYYYYMMTATRYRGRYSRTMYGRKTRTRY 3240
Bhutan-09030        TMCWYTWWAWSKSRWMGWRAWYWYCRCCGRCRYYYYMMTATRYRGRYSRTMYGRKTRTRY 3240
```

```
Bhutan-09005        YAYTTYAARWGGSRAARTARWCAYYGCYRATGYYCYCCCMYRYGTATGRWMYRRTYRYRY 3240
Indonesia-88035     CATTTTAAATGGCGAAGTAATCACYGCCGATGYYCYCCCMYRYGTATGRWMYRRTYRYRY 3240
Indonesia-88045     CATTTTAAATGGCGAAGTAATCACYGCCGATGYYCYCCCMYRYGTATGRWMYRRTYRYRY 3240
Indonesia-88065     TAYTTYAARWGGSRAARTARWCAYYGCYRATGYYCYCCCMYRYGTATGRWMYRRTYRYRY 3240
                              . .                   ** *     **    .* ** *. * **

Bhutan-09015        KYYRYCGKRRTSAAWYRGRARRCCAGTCGAYRYMKYYKRMACATCMRARRTATTRTTGRT 3300
Bhutan-09024        KYYRYCGKRRTSAAWYRGRARRCCAGTCGAYRYMKYCGRMACATCMRARRTATTRTTGRT 3300
Bhutan-09027        KYYRYCGKRRTSMAWYRGRARRCCAGTCGAYRYMKYYKRMACATCMRARRTATTRTTGRT 3300
Bhutan-09030        KYYRYCGKRRTSAAWYRGRARRCCAGTCGAYRYMKYCGRMACATCMRARRTATTRTTGRT 3300
Bhutan-09005        TTCACYATARKSAGTCGKRCRGSTGSYTSWCAYMKKTKRCRYRAMCGWRACMYWRYYKRY 3300
Indonesia-88035     TTCACYATARKSMRTCGKRCRGSTGSYTSWCATMKKTKRCRYRAMCGWRACMYWRYYKRY 3300
Indonesia-88045     TTCACYATARKSMRTCGKRCRGSTGSYTSWCATMKKTKRCRYRAMCGWRACMYWRYYKRY 3300
Indonesia-88065     TTCACYATARKSAGTCGKRCRGSTGSYTSWCATMKKTKRCRYRAMCGWRACMYWRYYKRY 3300
                    .     .. *.*      *.* . ..  .    **   *    :    *     *   *

Bhutan-09015        CSGKYSGRKYARACGMYWTCASRRMCTMTTTYYAAWGGYTASKCTCTTTAACMRYYACCR 3360
Bhutan-09024        CSGKYSGRKYARACGMYWTCASRRMCTMTTTYYAAWGGYTASKCTCTTTAACMRYYACCR 3360
Bhutan-09027        CSGKYSGRKYARACGMCWTCASRRMCTMTTTYYAAWGGYTASKCTCTTTAACMRYYACCR 3360
Bhutan-09030        CSRKYSGRKYARACGMCWTCASRRMCTMTTTYYAAWGGYTASKCTCTTTAACMRYYACCR 3360
Bhutan-09005        TGGKYSRAWCRAMSKMCAYMWGAGATCCWWGCTWRTCRYGRGKAKTACCRGGAATTMYYS 3360
Indonesia-88035     TGGKYSRAWCRAMSKMCAYMWGAGATCCWWGCTWRTCRYGRGKAKTACCRGGAATTMYYS 3360
Indonesia-88045     TGGKYSRAWCRAMSKMCAYMWGAGATCCWWGCTWRTCRYGRGKAKTACCRGGAATTMYYS 3360
Indonesia-88065     TGGKYSRAWCRAMSKMCAYMWGAGATCCWWGCTWRTCRYGRGKAKTACCRGGAATTMYYS 3360
                     . ***       . *     .                *  .*.. :   .

Bhutan-09015        TRRSKYRYYSRTGAAYRRMGCKCGYTATKRAKYWRAGTCTTYAAMKGTMCKYTMGCRKTC 3420
Bhutan-09024        TRRSKYRYYSRTGAAYRRMGCKCGYTATKRAKYWRAGTCTTYAAMKGTMCKYTMGCRKTC 3420
Bhutan-09027        TRRSKYRYYSRTGAAYRRMGCKCGYTATKRAKYWRAGTCTTYAAMKGTMCKYTMGCRKTC 3420
Bhutan-09030        TRRSKYRYYSRTGAAYRRMGCKCGYTATKRAKYWRAGTCTTYAAMKGTMCKYTMGCRKTC 3420
Bhutan-09005        YGGGGCGCTGAYTRWTGRMAYGYRCYWYTGTKTARMKYAYWYRWMTTKCAKYYMAYATYY 3420
Indonesia-88035     YGGGGCGCTGAYTRWTGRMAYGYRCYWYTGTKTARMKYAYWYRWMTTKCAKYYMAYATYY 3420
Indonesia-88045     YGGGGCGCTGAYTRWTGRMAYGYRCYWYTGTKTARMKYAYWYRWMTTKCAKYYAAYATYY 3420
Indonesia-88065     YGGGGCGCTGAYTRWTGRMAYGYRCYATTGTKTARMKYAYWYRWMTTKCAKYYMAYATYY 3420
                       .     .       **.        . :*  *   .  *  *. . .**  .  .

Bhutan-09015        CRCAKWTRCMRTYRWMRWCRMWGYTTGGTTTYCGWAACCCRRWRSKAGRRCCAYYRAGRY 3480
Bhutan-09024        CRCAKWTRCMRTYRWMRWCRMWGYTTGGTTTYCGWAACCCRRWRSKAGRRCCAYYRAGRY 3480
Bhutan-09027        CRCAKWTRCMRTYRWMRWCRMWGYTTGGTTTYMRWAACCCRRWRSKAGRRCCAYYRAGRY 3480
Bhutan-09030        CRCAKWTRCMRTYRWMRWCRMWGYTTGGTTTYMRWAACCCRRWRSKAGRRCCAYYRAGRY 3480
Bhutan-09005        YRTGKTYRYCAYTGWMAAMRATCTYYACWKTTCGAGRMYMGGWACGWKRAMYRCTAGRRT 3480
Indonesia-88035     YRTGKTYRYCAYTGTCAAMRATCTTYACRKWTCGAGRMYMGGWACGWKRAMYRCTAGRRT 3480
Indonesia-88045     YRTGKTYRYCAYTGTCAAMRATCTTYACRKWTCGAGRMYMGGWACGWKRAMYRCTAGRRT 3480
Indonesia-88065     YRTGKTYRYCAYTGWMAAMRATCTTYACWKTTCGAGRMYMGGWACGWKRAMYRCTAGRRT 3480
                     * .*  *           *      .  .     .      * .   *       . *

Bhutan-09015        RTYYRTTGRTTYAYASKCRTCTTCAAMYGGGGWARAAYSCRAMAYMGACRKCTRWRGARR 3540
Bhutan-09024        RTYYRTTGRTTYAYASKCRTCTTCAAMYGGGGWARAAYSCRAMAYMGACRKCTRWRGARR 3540
Bhutan-09027        RTYYRTTGRTTYAYASKCRTCTTCAAMYGGGGWARAAYSCRAMAYMGACRKCTRWRGARR 3540
Bhutan-09030        RTYYRTTGRTTYAYASKCRTCTTCAAMYGGGGWARAAYSCRAMAYMGACRKCTRWRGARR 3540
Bhutan-09005        GYTYAYWRRYKCTCRSGSGWYGCYRRCTAARRTMRAACGYARAGYMRCSRKGCATGRMAA 3540
Indonesia-88035     GYTYAYWRRYKCTCRSGSGWYGCYRRCTAARRTMRAACGYARAGYMRCSRKGCATGRMAA 3540
Indonesia-88045     GYTYACWRRYKCTCRGGSGWYGCYRRCTAARRTMRATCGYAAAGYMRCGRKGCATGRMAA 3540
Indonesia-88065     GYTYACWRRYKCTCRGGSGWYGCCRRCTAARRTMRTACGYAAAGYMRCSRKGCATGRMAA 3540
```

```
                      *    * . :  . .          ..    *:: .    .** . **

Bhutan-09015          YAMTKTWARGCSMGAARRRYCRWRRAATRCWGYYCRGRSWAYYYYGCWWACRSYGWGSRK 3600
Bhutan-09024          YAMTKTWARGCSMGAARRRYCRWRRAATRCWGYYCRGRSWAYYYYGCWWACRSYGWGSRK 3600
Bhutan-09027          YAMTKTWARGCSMGAARRRYCRWRRAATRCWGYYCRGRSWAYYYYGCWWACRSYGWGSRK 3600
Bhutan-09030          YAMTKTWARGCSMGAARRRYCRWRRAATRCWGYYCRGRSWAYYYYGCWWACRSYGWGSRK 3600
Bhutan-09005          TTMKGATGRKAGCRTRAGATYGMRGMGCAYAKTYTAKGCTTTCYTAYTAGTACTRARGAK 3600
Indonesia-88035       TTMKGATGRKAGCRTRAGATYGMRGMGCAYAKTYTAKGCTTTCYTAYTAGTACTRARGAK 3600
Indonesia-88045       TTMKGATGRKAGCRTRAGATYGMRGMGCAYAKTYTAKGCTTTCYTAYTAGTACTRARGAK 3600
Indonesia-88065       TTMKGATGRKAGCRTRAGATYGMRGMGCAYAKTYTAKGCTTTCYTAYTAGTACTRARGAK 3600
                       :*. : .* ..  :        *  .      *    . :  * .   .  .    . *

Bhutan-09015          YYSGYRRARCACKYTMGRCARTYYWATWAWYRYKYSYTCAYRGYKYCKAWMKTKAATWKC 3660
Bhutan-09024          YYSGYRRARCACKYTMGRCARTYYWATWAWYRYKYSYTCAYRGYKYCKAWMKTKAATWKC 3660
Bhutan-09027          YYSGYRRARCACKYTMGRCARTYYWATWAWYRYKYSYTCAYRGYKYCKAWMKTKAATWKC 3660
Bhutan-09030          YYSGYRRARCACKYTMGRCARTYYWATWAWYRYKYSYTCAYRGYKYCKAWMKTKAATWKC 3660
Bhutan-09005          YTSRTARGGYTTKYWMARAWAWTTTMKAGWCGCGCGCWTWTGSCKYTKMWMKYKCRWWGG 3660
Indonesia-88035       YTSRTARGGYTTKYWMARAWAWTTTMKAGWCGCGCGCWTWTGSCKYTKMWMKYKCRWWGG 3660
Indonesia-88045       YTSRTARGGYTTKYWMARAWAWTTTMKAGWCGCGCGCWTWTGSCKYTKMWMKYKCRWWGG 3660
Indonesia-88065       YTSRTARGGYTTKYWMARAWAWTTTMKAGWCGCGCGCWTWTGSCKYTKMWMKYKCRWWGG 3660
                      * *   *.  : ** *.*.       . .*     .      . ** * *** *.  *

Bhutan-09015          GAGRACSSWACKYRARTRYAGGYAACTYGYYGWYGCYYKRTAAGGTKAKCYRRYTSKCGY 3720
Bhutan-09024          GAGRACSSWACKYRARTRYAGGYAACTYGYYGWTGCYYKRTAAGGTKAKCYRRYTSKCGY 3720
Bhutan-09027          GAGRACSSWACKYRARTRYAGGYAACTYGYYGWYGCTCTRTAAGGTKAKCYRRYTSKCGY 3720
Bhutan-09030          GAGRACSSWACKYRARTRYAGGYAACTYGYYGWYGCYYKRTAAGGTKAKCYRRYTSKCGY 3720
Bhutan-09005          RWSGMMSSWWMKYGMAGAYCAAYWRTCTACCTWTKYTCGRYWGRKYTRKTCAGCKCTYSY 3720
Indonesia-88035       RWSGMMSSWWMKYGMAGAYCAAYWRTCTACCTWTKYTCGRYWGRKYTRKYCAGCKCTYSY 3720
Indonesia-88045       RWSGMMSSWWMKYGMAGAYCAAYWRTCTACCTWTKYTCGRYWGRKYTRKYCAGCKCTYSY 3720
Indonesia-88065       RWSGMMSSWWMKYGMAGAYCAAYWRTCTACCTWTKYTCGRYWGRKYTRKYCAGCKCTYSY 3720
                        .   ***  **     *...*     .   *      *  .   . *     ... .*

Bhutan-09015          YCTYMAGTRCATAYRMSTYRMYYGCYYGKCGATCSGSYYGWWYKAARWCRYCCRCWTRGG 3780
Bhutan-09024          YCTYMAGTRCATAYRMSTYRMYYGCYYGKCGATCSGSYYGWWYKAARWCRYCCRCWTRGG 3780
Bhutan-09027          YCTYMAGTRCATAYRMSTYRMYYGCYYGKCGATYSGSYYGWWYKAARWCRYCCRCWTRGG 3780
Bhutan-09030          YCTYMAGTRCATAYRMSTYRMYYGCYYGKCGATYSGSYYGWWYKAARWCRYCCRCWTRGG 3780
Bhutan-09005          YYATCRSYASAAWTAAGYYAMTYTTTTRKTSRKCGRSTCRWTYKRRAATGCMYGTTYRAR 3780
Indonesia-88035       YYATCRSYASRAWTAAGYYAMTCTTTTRKTSRKCGRSTCRWTYKRRAATGCMYGTTYRAR 3780
Indonesia-88045       YYATCRSYASAAWTAAGYYAMTYTTTTRKTSRKCGRSTCRWTYKRRAATGCMYGTTYRAR 3780
Indonesia-88065       YYATCRSYASAAWTAAGYYAMTYTTTTRKTSRKCGRSTCRWTYKAAAATGCMYGTTYRAR 3780
                      * :   .  . :    . * *       * . . . *   * **             *.

Bhutan-09015          RAWYAYAAKCCCRYRGTATRSGYGYYYRTTCYRYCSCATGCAACSAYRRKWKCYYTASWM 3840
Bhutan-09024          RAWYAYAAKCCCRYRGTATRSGYGYYYRTTCYRYCSCATGCAACSAYRRKWKCYYTASWM 3840
Bhutan-09027          RAWYAYAAKCCCRYRGTATRSGYGYYYRTTCYRYCSCATGCAACSAYRRKWKCYYTASWM 3840
Bhutan-09030          RAWYAYAAKCCCRYRGTATRSGYGYYYRTTCYRYCSCATGCAACSAYRRKWKCYYTASWM 3840
Bhutan-09005          GRWYRTRRGTYYRCGRYMWGGRCRTYYGYYSCRYYGYWYKYMRYCRCRGGAKSYTYRSAM 3840
Indonesia-88035       GRWYRTRRGTYYRCGRYMWGGRCRTYYGYYSCRYYGTWYKYMRYCRCRGGAKSYTYRSAM 3840
Indonesia-88045       GRWYRTRRGTYYRCGRYMWGGRCRTYYGYYSCRYYGTWYKYMRYCRCRGGAKSYTYRSAM 3840
Indonesia-88065       GRWYRTRRGTYYRCGRYMWGGRCRTYYGYYSCRYYGTWYKYMRYCRCRGGAKSYTYRSAM 3840
                        **        *       .    **   . ** .        .  *   *.*   * *

Bhutan-09015          AGKGACGAMGGYTMRASRYKRAYTAWRCGRTYYYYYRMRRAMTCTTRCWMMYMTMMWMTK 3900
Bhutan-09024          AGKGACGAMGGYTMRASRYKRAYTAWRCGRTYYYYYRMRRAMTCTTRCWMMYMTMMWMTK 3900
```

```
Bhutan-09027        AGKGACGAMGGYTMRASRYKRAYTAWRYRRTYYYYYRMRRAMTCTTRCWMMYMTMMWMTK 3900
Bhutan-09030        AGKGACGAMGGYTMRASRYKRAYTAWRYRRTYYYYYRMRRAMTCTTRCWMMYMTMMWMTK 3900
Bhutan-09005        GAGKWSKTCAAYKARMCGYTAMCYWAATRAWYTCTCAMGGMCYYWYGGACMYCCSAWMYT 3900
Indonesia-88035     GAGKWSKTCAAYKARMCGYTAMCYWAATRAWYTCTCAMGGMCCTTTGGACMYCCSAWMYT 3900
Indonesia-88045     GAGKWSKTCAAYKAAMCGYTAMCYWAATRAWYTCTCAMGGMCYYTTGGACMYCCSAAMYT 3900
Indonesia-88065     GAGKWSKTCAAYKAAMCGYTAMCYWAATRAWYTCTCAMGGMCYYTTGGACMYCCSAWMYT 3900
                    ..   . : ..*.   . *.           *     *            **     * .

Bhutan-09015        AGMKRYRRRGRYRGYGGGSRGYYYTTATYTYYYYTWKRYCGRYYKYCWRARWCKCAYSYY 3960
Bhutan-09024        AGMKRYRRRGRYRGYRGGSRGYYYTTATYTYYYYTWKRYCGRYYKYCWRARWCKCAYSYY 3960
Bhutan-09027        AGMKRYRRRGRYRGYRGGSRGYYYTTATYTYYYYTWKRYCGRYYKYCWRARWCKCAYSYY 3960
Bhutan-09030        AGMKRYRRRGRYRGYRGGSRGYYYTTATYTYYYYTWKRYCGRYYKYCWRAGTCKCAYSYY 3960
Bhutan-09005        RSAKACGAATGTGRYATTGARCYTYYRYYCCCTCAWKRYYKGTTGTAAAGAAYTYGTCCY 3960
Indonesia-88035     RSAKACGAATGTGRYATTGARCYTYYRYYCCCTCAWKRYYKGTTGTAAARAAYTYGTCCY 3960
Indonesia-88045     RSAKACGAATGTGRYATTGARCYTYYRYYCCCTCAWKRYYKGTTGTAAARAAYTYGTCCY 3960
Indonesia-88065     RSAKACGAATGTGRYATTGARCYTYYRYYCCCTCAWKRYYKGTTGTAAARAAYTYGTCCY 3960
                     . *          *   .   *     *     :****       .      . . . *

Bhutan-09015        YTMGRGYGGRTWAAYCAYKAWYGRGKYAAYAGRSYAKRYRWWCYAGRWYSKCGKAAARRW 4020
Bhutan-09024        YTMGRGYGGRTWAAYCAYKAWYGRGKYAAYAGRSYAKRYRWWCYAGRWYSKCGKAAARRW 4020
Bhutan-09027        YTMGRGYGGRTWAAYCAYKAWYGRGKYAAYAGRSYAKRYRWWCYAGRWYSKCGKAAARRW 4020
Bhutan-09030        YTMGRGYGGRTWAAYCAYKAWYGRGKYAAYAGRSYAKRYRWWCYAGRWYSKCGKAAARRW 4020
Bhutan-09005        YYCSGSCGRRAAGWCTRTTMTTRRRKCWWTMTGSCRWATATWMYWRGWCGKYKTMGRGAW 4020
Indonesia-88035     YYCSGSCGRRAAGWCTRTTMTTRRRKCWWTMTGSCRWATATWMYWRGWCGKYKTMGRGAW 4020
Indonesia-88045     YYCSGSCRRRAAGWCTRTTMTTRRRKCWWTMTGSCRWATATWMYWRGWCGKYKTMGRGAW 4020
Indonesia-88065     YYCSGSCRRRAAGWCTRTTMTTRRRKCWWTMTGSCRWATATWMYWRGWCGKYKTMGRGAW 4020
                    *  . .   *: .     .    * *       *       * *   * .*  . .   *

Bhutan-09015        YYAATCAGRWWWRSKSMYKACTAYCGAAYCYWYTTYYKMACKTAKTCGWRRMMRRRTYGC 4080
Bhutan-09024        YYAATCAGRWWWRSKSMYKACTAYCGAAYCYWYTTYYKMACKKAKTCGWRRMMRRRTYGC 4080
Bhutan-09027        YYAATCAGRWWWRSKSMYKACTAYCGAAYCYWYTTYYKMACKTAKTCGWRRMARRRTYGC 4080
Bhutan-09030        YYAATCAGRWWWRSKSMYKACTAYCGAAYCYWYTTYYKMACKKAKTCGWRRMMRRRTYGC 4080
Bhutan-09005        YYCCCTGAGATAGGKCMYKRMYRYSKRRCYYTCYYCCGMGSTGRKCGRWGGAAAGGYCTG 4080
Indonesia-88035     YYCCCTGAGATAGGKCMYKRMYRYSKGRCYYTCYYCCGMGSTGRKCGRWGGAAAGGYCTG 4080
Indonesia-88045     YYCCCTGAGATAGGKCMYKRMYRYSKRRCYYTCYYCCGMGSTGGTCGRWGGAAAGGYCTG 4080
Indonesia-88065     YYCCCTGAGATAGGKCMYKRMYRYSKRRCYYTCYYCCGMGSTGRKCGRWGGAAAGGYCTG 4080
                    **..  ..     .*.***    *.     *       *...  .   *

Bhutan-09015        TAGTCCRGRSCGTRTYRMRMCCGCGAACAWCGGYRWYRYAMKMAWWWMMRRARYTKGMRM 4140
Bhutan-09024        TAGTCCRGRSCGTRTYRMRMCCGCGAACAWCGGYRWYRYAMKMAWWWMARRARYTKGMRM 4140
Bhutan-09027        TAGTCCRGRSCGTRTYRMRMCCGCGAACAWCGGYRWYRYAMKMAWWWMMRRARYTKGMRM 4140
Bhutan-09030        TAGTCCRGRSCGTRTYRMRMCCGCGAACAWCGGYRWYRYAMKMAWWWMMRRARYTKGMRM 4140
Bhutan-09005        YRSASYRGGCYRWAYTRMRAYYRYKRRYMAMKKCRATACRAKMGWWWAMARWRCWKTMGC 4140
Indonesia-88035     YRSASYRSGCYRWAYTRMRAYYGCKRRYMAMKKCRATACRAKMGWWWAMARWRCWKTMGC 4140
Indonesia-88045     YRSASYRGGCYRWAYTRMRAYYRYKRRYMAMKKCRATACRAKMGWWWAMARWRCWKTMGC 4140
Indonesia-88065     YRSASYRGGCYRWAYTRMRAYYRYKRRYMAMKKCRATACRAKMGWWWAMARWRCWKTMGC 4140
                      .:. *. .      ***               *      **.***   * *  * *

Bhutan-09015        WYCRGMRARYAMKATCRKMYGYTATASASWRCWYWGRKTYYRWWCGRGYMKTYYRWAAWY 4200
Bhutan-09024        WYCRGMRARYAMKATCRKMYGYTATASASWRCWYWGRKTYYRWWCGRGYMKTYYRWAWWY 4200
Bhutan-09027        WYCRGMRARYAMKATCRKMYGYTATASASWRCWYWGRKTYYRWWCGRGYMKTYYRWAWWY 4200
Bhutan-09030        WYCRGMRARYAMKATCRKMYGYTATASASWRCWYWGRKTYYRWWCGRGYMKTYYRWAAWY 4200
Bhutan-09005        AYAGRAGRRCRAGRYTGTCCKYCGKRSRCWRYTYAKRTCYTRAWTRGRCMKYCYRWWAWT 4200
Indonesia-88035     AYAGRAGRRCRAGRYTGTCCKYCGKRSRCWRYTYAKRTCYTRAWTRGRCMKYCYRWWAWT 4200
```

```
Indonesia-88045      AYAGRAGRRCRAGRYTGTCCKYCGKRSRCWRYTYAKRTCYTRAWTRGRCMKYCYRWWAWT 4200
Indonesia-88065      AYAGRAGRRCRAGRYTGTCCKYCGKRSRCWRYTYAKRTCYTRAWTRGRCMKYCYRWWAWT 4200
                      *.     *        .   * .. * .**  *  *. * * *     **  ***  *

Bhutan-09015         GCCGGCCRTYKGTRCGWRCYRGKRGYWTRTWSARSYTKWYARKWAKCMKKRSAYMYRYKM 4260
Bhutan-09024         GCCGGCCRTYTGTRCGWRCYRGKRGYWTRTWSARSYTKWYARKWAKCMKKRSAYMYRYKM 4260
Bhutan-09027         GCCGGCCRTYKGTRCGWRCYRGKRGYWTRTWSARSYTKWYARKWAKCMKKRSAYMYRYKM 4260
Bhutan-09030         GCCGGCCRTYKGTRCGWRCYRGKRGYWTRTWSAGSYTKWYARKWAKCMKKRSAYMYRYKM 4260
Bhutan-09005         AACKKYYRCCKRCRYSAGSTRRTRSCTWAKWCMACTYGWTRGTTMGGAGTGCRTCTACGM 4260
Indonesia-88035      AACKKYYRYYTRCRYSAGSTRRTRSCTWAKWCMACTYGWTRGTTCGGAGTGCRTCTACGM 4260
Indonesia-88045      AACKKYYRYYKRCRYSAGSTRRTRSCTWAKWCMACTYGWTRGTTMGGAGTGCRTCTACGM 4260
Indonesia-88065      AAYKKYYRYYKRCRYSAGSTRRTRSCTWAKWCMACTYGWTRGTTMGGAGTGCRTCTACGM 4260
                     ..     *  .  * .  . * .*.    .*.  .   *   .      . .       *

Bhutan-09015         RSMMARCYRTKKRYTSTMTSYCYWRWKGYMCYAWGCTATTRTGMRRYKKRRSMYRSRYAK 4320
Bhutan-09024         RSMMARSYRTKKRYTSTMTSYCYWRWKGYMCYAWGCTATTRTGMRRYKKRRSMYRSRYAK 4320
Bhutan-09027         RSMMARSYRTKKRYTSTMTSYCYWRWKGYMCYAWGCTATTRTGMRRYKKRRSMYRSRYAK 4320
Bhutan-09030         RSMMARCTRTKKRYTSTMTSYCYWRWKGYMCYAWGCTATTRTGMRRYKKRRSMYRSRYAK 4320
Bhutan-09005         GSCACAGCGYKTAYYSYCYSTYTAATGRTCYYCWRTYWAAACKMRACTGRAGCCASGYCK 4320
Indonesia-88035      GSCACAGCGYKTAYYSYCYSTYTAATKRTCYYCWRTYWAAACKMRACTGRAGCCASGYCK 4320
Indonesia-88045      GSCACAGCGYKTAYYSYCYSTYTAATKRTCYYCWRTYWAAACKMRACTGRAGCCASGYCK 4320
Indonesia-88065      GSCACAGCGYKTAYYSYCYSTYTAATKRTCYYCWRTYWAAACKMRACTGRAGCCASGYCK 4320
                      *  .     *. * *   *           *.*    ::   **  . * .   * *.*

Bhutan-09015         SARCTAWSTRGRYKCRYWMCWWWKTGYRWRRYACYMMAYGYRYRSSYWMYWTTRGRRKCY 4380
Bhutan-09024         SARCTAWSTRGRYKCRYWMCWWWKTGYRWRRYACYMMAYGYRYRSSYWMYWTTRGRRKCY 4380
Bhutan-09027         SARCTAWSTRGRYKCRYAMCWWWKTGYRWRRYACYMMAYGYRYRSSYWMYWTTRGRRKCY 4380
Bhutan-09030         SARCTAWSTRGRYKCRYAMCWWWKTGYRWRRYACYACAYGYRYRSSYWMYWTTRGRRKCY 4380
Bhutan-09005         SCATYRWSWRRRYTTRYACATTAKYKTGWGRYRYCACATGTGTRSGTTATAKKRRAATYY 4380
Indonesia-88035      SCATYRWSWRRRYTTACACATTAKYKTGWGRYRYCACAYRYGTRSGTTATAKKRRAATYY 4380
Indonesia-88045      SCATYRWSWRRRYTTRYACATTAKYKTGWGRYRYCACRYRYGTRSGTTATAKKRRAATYY 4380
Indonesia-88065      SCATYRWSWRRRYTTRYACATTAKYKTGWGRYRYCACRYRYGTRSGTTATAKKRRAATYY 4380
                     *.    ** * **.     .   *    * **           **.     ..*   . *

Bhutan-09015         RRGRWTKATRRCAYACWAYYCCMRRYMYCYRAYCAYTYTYSRTACYYARTGWCWRAKAYA 4440
Bhutan-09024         RRGRWTKATRRCAYACWAYYCCMRRYMYCYRAYCAYTYTYSRTACYYARTGWCWRAKAYA 4440
Bhutan-09027         RRGRWTKATRRCAYACWAYYCCMRRYMYCYRAYCAYTYTYSRTACYYARTGWCWRAKAYA 4440
Bhutan-09030         RRGRWTKATRRCAYACWAYYCCMRRYMYCYRAYCAYTYTYSRTACYYARTGWCWRAKAYA 4440
Bhutan-09005         AAARTYTRYRGSRCRYWRCCYMMGRYMYYCAGCYRYYTGYGAGGYYYWRWRWYTRGKRCR 4440
Indonesia-88035      AAARTYTRYRGSRCRYWRCCYMMGRYMYYCAGCYRYYTGYGAGGYYYWRWRWYTRGKRCR 4440
Indonesia-88045      AAARTYTRYRGSRCRYWRCCYMMGRYMYYCAGCYRYYTGYGAGGYYYWRWRWYTRGKRCR 4440
Indonesia-88065      AAARTYTRYRGSRCRYWRCCYMMGRYMYYCAGCYRYYTGYGAGGYYYWRWRWYTRGKRCR 4440
                       .*  .  * .    *     * ****   .   *   *.  . ** *  *  *.*

Bhutan-09015         MMYAGCYKSCWTTACAAYYGATCRTCCAYYRRGRTCTTTCWCYRYMWTAYTMRWWCGYKA 4500
Bhutan-09024         MMYAGCYKSCWTTACAAYYGATCRTCCAYYRRGRTCTTTCWCYRYMWTAYTMRWWCGYKA 4500
Bhutan-09027         MMYAGCYKSCWTTACAAYYGATCRTCCAYYRRGRTCTTTCWCYRYMWTAYTMRWWCGYKA 4500
Bhutan-09030         MMYAGCYKSCWTTACAAYYGATCRTCCAYYRRGRTCTTTCWCYRYMWTAYTMRWWCGYKA 4500
Bhutan-09005         MMYRSYCTGSAYYGMWGTTSRYYRWTTRCYGGKGCTYKWSWYCGCWTYRYCAAWATRCTT 4500
Indonesia-88035      MMYRSYCTGSAYYAMWGTTSRYYRWTTRCYGGKGCTYKWSWYCGCWTYRYCAAWATRCTT 4500
Indonesia-88045      MMYRSYCTGSAYYAMWGTTSRYYRWTTRCYGGKGCTYKWSWYCGCWTYRYCAAWATRCTT 4500
Indonesia-88065      MMYRSYCTGSAYYGMWGTTSRYYRWTTRCYGGKGCTYKWSWYCGCWTYRYCAAWATRCTT 4500
                     *** .  ...   .  .  .   *     *       . .*        *   *    .:
```

```
Bhutan-09015          RYTMYGWGRAGAWCSAAWYGRMWGRRRYYYKMGMYYYTRGYYGTCARRTGYKYGYGSCAC 4560
Bhutan-09024          RYTMYGWGRAGAWCSAAWYGRMWGRRRYYYKMGMYYYTRGYYGTCARRTGYKYGYGSCAC 4560
Bhutan-09027          RYTMYGWGRAGAWCSAAWYGRMWGRRRYYYKMGMYYYTRGYYGTCARRTGYKYGYGSCAC 4560
Bhutan-09030          RYTMYGWGRAGAWCSAAWYGRMWGRRRYYYKMGMYYYTRGYYGTCARRTGYKYGYGSCAC 4560
Bhutan-09005          ATCCTKWRAGAGRYCTRTYAAATSRGACTTKMRACTCWGSYYRCYRGRAKYTCSCRCYRC 4560
Indonesia-88035       ATCCTKWRAGAGRYCTATYAAATSRGACTTKMRACTCWGSYYRCYRGRAKYTCSCRCCAM 4560
Indonesia-88045       ATCCTKWRAGAGRYCTATYAAATSRGACTTKMRACTCWGSYYRCYRGRAKYTCSCRCCAM 4560
Indonesia-88065       ATCCTKWRAGAGRYCTRTYAAATSRGACTTKMRACTCWGSYYRCYRGRAKYTCSCRCYRM 4560
                            *  ...  .:  *.   .*     **       .**     *: *. .  .

Bhutan-09015          AYAYYTKYYGCKGGTAGTTMTSYGGRKKTAGCGYYRRTGRTGTYRARWTCAGYKACTTRR 4620
Bhutan-09024          AYAYYTKYYGCKGGTAGTTMTSYGGRKKTAGCGYYRRTGRTGTYRARWTCAGYKACTTRR 4620
Bhutan-09027          AYAYYTKYYGCKGGTAGTTMTSYGGRKKTAGCGYYRRTGRTGTYRARWTCAGYKACTTRR 4620
Bhutan-09030          AYAYYTKYYGCKGGTAGTTMTSYGGRKKTAGCGYYRRTGRTGTYRARWTCAGYKACTTRR 4620
Bhutan-09005          AYRYCKKCCRTGCAYRAYKAKGCRRATTYCRYATCGGTGRKRYTARRTCTGATGRTWGRG 4620
Indonesia-88035       RYRYCKKCCRTGCAYRAYKAKGCRRATTYCRYATCGGTGRKRYTARRTCTGATGRTWGRG 4620
Indonesia-88045       RYRYCKKCCRTGCAYRAYKAKGCRRATTYCRYATCGGKRRKRYTARRTCTGATGRTWGRG 4620
Indonesia-88065       RYRYCKKCCRTGCAYRAYKAKGCRRATTYCRYATCGGTGRKRYTARRTCTGATGRTWGRG 4620
                       * * .*      .  . .  ..    .. .  .    . *.     *   ..      *

Bhutan-09015          YYRYGTGTTASTTCRARYWYCGGRYRCYGTYCMMYCRTTCAMRWASYRRKYYRYGRRCCR 4680
Bhutan-09024          YYRYGTGTTASTTCRARYWYCGGRYRCYGTYCMMYCRTTCAMRWASYRRKYYRYGRRCCR 4680
Bhutan-09027          YYRYGTGTTASTTCRARYWYCGRRYRCYGTYCMMYCRTTCAMRWASYRRKYYRYGRRCCR 4680
Bhutan-09030          YYRYGTGTTASTTCRARYWYCGRRYRCYGTYCMMYCRTTCAMRWASYRRKYYRYGRRCCR 4680
Bhutan-09005          TYGTRKAYWCCGYMARRTWCYRGGKAYCRYCYCCCYRYYTMARTWCYAAKTCACRAGMSG 4680
Indonesia-88035       TYGTRKAYWCCGYMARRTWCYRGGKAYCRYCYCCCYRYYTMARTWCYAAKTCACRAGMSG 4680
Indonesia-88045       TYGTRKAYWCCGYMARRTWCYRGGKAYCRYCYCCCYRYYTMARTWCYAAKTCACRAGMSG 4680
Indonesia-88065       TYGTRKAYWCCGYMARRTWCYRGGKAYCRYCYCCCYRYYTMARTWCYAAKTCACRAGMSG 4680
                       *   ..  ..     * *                 *     *  .*  *        .

Bhutan-09015          YCRCYAGCTTCKAWGARTTCYACTTYTRRYAASTCTCWYYCARYKARSRRACCTMCTYKR 4740
Bhutan-09024          YCRCYAGCTTCKAWGARTTCYACTTYTRRYAASTCTCWYYCARYKARSRRACCTMCTYKR 4740
Bhutan-09027          YCRCYAGCTTCKAWGARTTCYACTTYTRRYAASTCTCWYYCARYKARSRRACCTMCTYKR 4740
Bhutan-09030          YCRCYAGCTTCKAWGARTTCYACTTYTRRYAASTCTCWYYCARYKARSRRACCTMCTYKR 4740
Bhutan-09005          TYAYTWKYACTKWASMRGWSCWYWYYWGGCWGCWYKYTCYYRGYKRGSRRWMMYCMKTKA 4740
Indonesia-88035       TYAYTWKYACTKWASMRGWSCWYWYYWGGCARCWYKYKCYCRRYKRGSRRWMMYCMKTKA 4740
Indonesia-88045       TYAYTWKYACTKWASMRGWSCWYWYYWGGCARCWYKYKCYCRRYKRGSRRWMMYCMKTKA 4740
Indonesia-88065       TYAYTWKYACTKWASMRGWSCWYWYYWGGCWGCWYKYTCYYRGYKRGSRRWMMYCMKTKA 4740
                              :  *  . *  .     *      .  .   *   **  ***      . *

Bhutan-09015          RCGACASKGMMGSYSRTTGGRRTTYCGWWARKKSMYTRCYRGGRMWKSYCTMCRGAWWYR 4800
Bhutan-09024          RCGACASKGMMGSYSRTTGGRRTTYCGWWARKKSMYTRCYRGGRMWKSYCTMCRGAWWYR 4800
Bhutan-09027          RCGACASKGMMGSYSRTTGGRRTTYCGWWARKKSMYTRCYRGGRMWKSYCTMCRGAWWYR 4800
Bhutan-09030          RCGACASKGMMGSYSRTTGGRRTTYCGWWARKKSMYTRCYRGGRMWKSYCTMCRGAWWYR 4800
Bhutan-09005          GMRMSRGKTMMRGCCRKYRRGRYKCGKAWGGGTCMCKGYTGKGGAWWGTMWAYGRMTWYA 4800
Indonesia-88035       GMRMCRGKTMMRGCCRKYRRGRYKCGKATGGKKCMCKGYTGKRGAWWGTMWAYGRMTWYA 4800
Indonesia-88045       GMRMSRGKTMMRGCCRTYRRGRYKCGKATGGKKCMCKGYTGKRGAWWGTMWAYGRMTWYA 4800
Indonesia-88065       GMRMSRGKTMMRGCCRKYRRGRYKCGKAWGGGTCMCKGYTGKGGAWWGTMWAYGRMTWYA 4800
                          . .* ** . .*.    * .     .  ..* .        * .         **

Bhutan-09015          YGTAARTSGSGRMCTTAMARTWKRWYTTGRWWCCAYAGGARTRTTARYCMAYKYAGYYTA 4860
Bhutan-09024          YGTAARTSGSGRMCTTAMARTWKRWYTTGRWWCCAYAGGARTRTTARYCMAYKYAGCYTA 4860
Bhutan-09027          YGTAARTSGSGRMCTTAMARTWKRWYTTGRWWCCAYWSSARTRTTARYCMAYKYAGYYTA 4860
Bhutan-09030          YGTAARTSGSGRMCTTAMARTWKRWYTTGRWWCCAYAGGARTRTTARYCMAYKYAGYYTA 4860
```

```
Bhutan-09005        YRKGTAKCSGRGMYGKTCCGYAGRACCCSRWTMYRTAGGGRYGYGAGYACRCGTMGCYWM 4860
Indonesia-88035     YRKGTAKCSGRGMYGKTCCGYAGRACCYSRWTMYRTWSSRRYGYGRGYCCRCGTMKCYWM 4860
Indonesia-88045     YRKGTAKCSGRGMYGKTCCGYAGRACCYSRWTMYRTWSSRRYGYGRGYCCRCGTMGCYWM 4860
Indonesia-88065     YRKGTAKCSGGGMYGKTCCGYAGRACCCSRWTMYRTAGGGRYGYGAGYACRCGTMGCYWM 4860
                    * ..: ....  *  .: .    *    .**      .. *      *.        *

Bhutan-09015        CYRAKMRYYRWRRARAACTTWGYKYCGYGAMRAKAAAMYRKATATCKWTCKWRTGTGTGR 4920
Bhutan-09024        CYRAKMRYYRTGAARAACTTWGYKYCGYGAMRAKAAAMYRKATATCKWTCKWRTGTGTGR 4920
Bhutan-09027        CYRAKMRYYRWRRARAACTTWGYKYCGYGAMRAKAAAMYRKATATCKWTCKWRTGTGTGR 4920
Bhutan-09030        CYRAKMRYYRTGAAGAACTTWGYKYCGYGAMRAKAAAMYRKATATCKWTCKWRTGTGTGR 4920
Bhutan-09005        YYRRTAACTAAARRAWWYCCARTTCYRCRRCARKRRRAYATGYCYMTTKYKWRKRYRYGG 4920
Indonesia-88035     YYRRTAACTAAARRATTTCCARTTCYRCRRCARGRRRAYATGYCYMTTTYKWRKRYRYRG 4920
Indonesia-88045     YYRRTAACTAAARRATTTCCAATTCYRCRRCARGRRRAYATGCCCMTTTYKWRKRYRTRG 4920
Indonesia-88065     YYRRTAACTAAARRATTYCCARTTCYRCRRCARKRRRAYATGCCCMTTTYKWRKRYRYRG 4920
                     ** .                  .              * .. .  . . ***.

Bhutan-09015        TRCYKWRWTRKYYYRRTARWYYYKKRTCWYYCCTAWWRWASARMAWMWCGCCSWSRGTYC 4980
Bhutan-09024        TRCYKWRWTRKYYYRRTARWYYYKKRTCWYYCCTAWWRWASARMAWMWCGCCSWSRGTYC 4980
Bhutan-09027        TRCYKWRWTRKYYYRRTARWYYYKKRTCWYYCCTAWWRWASARMAWMWCGCCSWSRGTYC 4980
Bhutan-09030        TRCYKWRWTRKYYYRRTARWYYYKKRTCWYYCCTAWWRWASARMAWMWCGCCSWSRGTYC 4980
Bhutan-09005        YGYCTWRWYAKYYYARKRGWYTTKTRYTWYTGGYTWWRWRCRGMRTAASRYSGWSGWWCY 4980
Indonesia-88035     YGYCTWRWYAKYYYARKRGWYTTKTRYTWYTGGYTWWRWRCRGMRTAASRYSGWSGWWCT 4980
Indonesia-88045     YGYCTWRWYAKYYYARKRGWYTTKTRYTWYTGGYTWWRWRCRGMRTAASRYSGWSGWWCY 4980
Indonesia-88065     YGYCTWRWYAKYYYARKRGWYTTKTRYTWYTGGYTWWRWRCRGMRTAASRYSGWSGWWCT 4980
                        .***  **** *.  **  *.*  **    :**** .  *    .  ..**

Bhutan-09015        KYGMTAWWTARRMCKCMYGRWATGSWYCMAATYYYGGTYGYRRYKARRWYCWYYKARGGG 5040
Bhutan-09024        KYGMTAWWTARRMCKCMYGRWATGSWYCMAATYYYGGTYGYRRYKARRWYCWYYKARGGG 5040
Bhutan-09027        KYGMTAWWYARRMCKCMYGRWATGSWYCMAATYYYGGTYGYRRYKARRWYCWYYKARGGG 5040
Bhutan-09030        KYGMTAWWYARRMCKCMYGRWATGSWYCMAATYYYGGTYGYRRYKARRWCCWYYKARGGG 5040
Bhutan-09005        KTRAYRAWCWAGAGTTCTRRAGYAGWTSMRRKCCTAAYCGYGGYKTAAACTTCTGWASTR 5040
Indonesia-88035     GTRAYRAWCWAGAGTTCTRRAGYAGWTSMRRKCCTAAYCRYGGYKTAAACTTCTGWASTR 5040
Indonesia-88045     KTRAYRAWCWAGAGTTCTRRAGYAGWTSMRRKCCTAAYCRYGGYKTAAACTTCTGWASTR 5040
Indonesia-88065     GTRAYRAWCAAGAGTTCTRRAGYAGWTSMRRKCCTAAYCGYGGYKTAAACTTCTGWASTR 5040
                           *      .    * . ..* .*  .   ..   *  **:           .

Bhutan-09015        WRGGGYGKMKRGKWAMAWRWKYAGRWGWAMSGRMTRTSRGWAWCGYRATARAGTWCGYYG 5100
Bhutan-09024        WRGGGYGKMKRGKWAMAWRWKYAGRWGWAMSGRMTRTSRGWAWCGYRATARAGTWCGYYG 5100
Bhutan-09027        WRGGGYGKMKRGKWAMAWRWKYAGRWGWAMSGRMTRTSRGWAWCGYRATARAGTWCGYYG 5100
Bhutan-09030        WRGGGYGKMKRGKWAMAWRWKYAGRWGWAMSGRMTRTSRGWAWCGYRATARAGTWCGYYG 5100
Bhutan-09005        WGRRKYKKCGAAGATMGWRTTYRSAAGAMMYRAMARYGGATRTYRTGRGTRGACWCKYTR 5100
Indonesia-88035     WGRRKYKKCGAAGATMGWRTTYRSAARAMMYRAMARYGGATRTYRTGRGTRGACWYKYTR 5100
Indonesia-88045     WGRRKYKKCGAAGATCGWRTTYRSAARAMMYRAMARYGGATRTYRTGRGTRGACWYKYTR 5100
Indonesia-88065     WGRRKYKKCGAAGATMGWRTTYRSAAGAMMYRAMARYGGATRTYRTGRGTRGACWYKYTR 5100
                    *    * *   .  : .** .* .     *   *:* . .         :*.. *  *

Bhutan-09015        CYKGTGAGCTCCGTAYYWRGCRSCGCACRYKYAWAYCGRRWAGCYRAASWTWTGSARYYY 5160
Bhutan-09024        CYKGTGAGCTCCGTAYYWRGCRSCGCACRYKYAWAYCGRRWAGCYRAASWTWTGSARYYY 5160
Bhutan-09027        CYKGTGAGCTCCGTAYYWRGCRSCGCACRYKYAWAYCGRRWAGCYRAASWTWTGSARYYY 5160
Bhutan-09030        CYKGTGAGCTCCGTAYYWRGCRSCGCACRYKYAWAYCGRRWAGCYRAASWTWTGSARYYY 5160
Bhutan-09005        YCTACRASYYTYKYWCYTGGYRCYAYRMATTTWAACGASGWMCYYATASAKTYSCRGTTT 5160
Indonesia-88035     YCTRCRAGCTTYKYACYTGAYRCYAYRMATTTWAWCGRSGWMCYYATASAKTYSCRGTTT 5160
Indonesia-88045     YCTRCRWGCTTYKYACYTGAYRCYAYRMATTTWAWCGRSGWMCYYATASAKTYSCRGTTT 5160
Indonesia-88065     YCTRCRASYYTYKYWCYTGGYRCYAYRMATTTWAACGASGWMCYYATWSAKTYSCRGTTT 5160
```

```
                                  .     .          *  . *. .       .          *    * : * .  ..

Bhutan-09015           WRMYGGGKMTYTAGYGYRSRAGYRCCWCARYWGTMYRGYWYATGTRCCWYYGRCCYKYKG 5220
Bhutan-09024           TGMYGGGKMTYTAGYGYRSRAGYRCCWCARYWGTMYRGYWYATGTRCCWYYGRCCYKYKG 5220
Bhutan-09027           WRMYGGGKMTYTAGYGYRSRAGYRCCWCARYWGTMYRGYWYATGTRCCWYYGRCCYKYKG 5220
Bhutan-09030           WRMYGGGKMTYTAGYGYRSRAGYRCCWCARYWGTMYRGYWYATGTRCCWYYGRCCYKYKG 5220
Bhutan-09005           WGCCAARKMWYWRRYRTRSGRRYAYYTTRRYASKCYAATTYWTGYAYYTTCKGYGCGTTS 5220
Indonesia-88035        WGCCAARKMAYWRRYRTRSGRRYAYYTTRRYASKCYAATTYWKSTAYYTTCKGYCCGTTS 5220
Indonesia-88045        WGCCAARKMWYWRRYRTRSGRRYAYYTTRRYASKCYAATTYWKSYAYYTTCKGYCCGTTS 5220
Indonesia-88065        WGCCAARKMWYWRRYRTRSGRRYAYYTTRRYASKCYAATTYWTGYAYYTTCKGYGCGTTS 5220
                          .. ** *    *  **    *       ** .. * .  * ..               ..

Bhutan-09015           AAWMSTSAWYGMRCRTTTYYGATRWRASATARYSCTGAWRRASACKCGATRRKTWRTGGR 5280
Bhutan-09024           AAWMSTSAWYGMRCRTTTYYGATRWRASATARYSCTGAWRAASACKCGATRRKTWRTGGR 5280
Bhutan-09027           AAWMSTSAWYGMRCRTTTYYGATRWRASATARYSCTGAAAAASACKCGATRRKTWRTGGR 5280
Bhutan-09030           AAWMSTSAWYGMRCRTTTYYGATRWRASATARYSCTGAAAAASACKCGATRRKTWRTGGR 5280
Bhutan-09005           WTWMGYGWATKAGTRYYYTCRGAGTGRGWKRACGYCRRAAGTGWMTGGRYGAGTWGCKAR 5280
Indonesia-88035        WTWMGYGWATKAGTRYYYTCRGAGTGRGWKRACGYCGRAAGTGACTGRRYGAGYWGCKAR 5280
Indonesia-88045        WTWMGYGWATKAGTRYYYTCRGAGTGGGWKRACGYCGRAAGTGACTGRRYGAGYWGCKAR 5280
Indonesia-88065        WTWMGYGWATKAGTRYYYTCRGAGTGRGWKRACGYCGRAAGTGACTGGRYGAGTWGCKAR 5280
                        :**. .       *       .:     . .   .        :.  .         *   .*

Bhutan-09015           AKRKTSCTYCASMRRRYCMRYYKTMRRAMRGYYYKCYRWCTTRARSGMRWRGMMTTWYRM 5340
Bhutan-09024           AKRKTSCTYCASMRRRYCMRYYKTMRRAMRGYYYKCYRWCTTRARSGMRWRGMMTTWYRM 5340
Bhutan-09027           AKRKTSCTYCASMRRRYCMRYYKTMRRAMRGYYYKCYRWCTTRARSGMRWRGMMTTWYRM 5340
Bhutan-09030           AKRKTSCTYCASMRRRYCMRYYKTMRRAMRGYYYKCYRWCTTRARSGMRWRGMMTTWYRM 5340
Bhutan-09005           MGRTYGCYYMACMGAGCYCGTTGWMRRWCRSTCTKSTRTYYTRWAGRAATGRMCCGTCRC 5340
Indonesia-88035        MGATYGSYYARCMGAGCYCGTTGWMRRWMRSTTTCTRTYYWRWAGRAATGRMCCGWCRC 5340
Indonesia-88045        MGATYGSYYARCMGAGCTCGTTGWMRRWMRSTTTKCTRTYYWRWAGRAATGRMCCGWCRC 5340
Indonesia-88065        MGRTYGCYYMACMGAGCYCGTTGWMRRWCRSTCTKSTRTYYTRWAGRAATGRMCCGTCRC 5340
                          . .. *  .*           ***  *.   .. *     *  .       *      *

Bhutan-09015           YAMCCAYWMWWRMWCYYRASRCYKYKTYMTGSTGAGACYKGAWRYAGGRGARRCTARCSCTA 5400
Bhutan-09024           YAMCCAYWMWWRMWCYYRASRCYKYKTYMTGSTGAGACYKGAWRYAGGRGARRCTARCSCTA 5400
Bhutan-09027           YAMCCAYWMWWRMWCYTAASRCYKYKTYMTGSTGAGACYKGAWRYAGGRGARRCTARCSCTA 5400
Bhutan-09030           YAMCCAYWMWWRMWCYYRASRCYTTYMTGSTGAGACYKGAWRYAGGRGARRCTARCSCTA 5400
Bhutan-09005           TAMYYATWCAAAMTCYYRRSRYYKYYCYKSCRRARSYTSRARCRRRRRRRTCGGMCYYA 5400
Indonesia-88035        TMMYYRTWCAAAAAAYYRRSRYYKYYCYKSCRRARSYTSRARCRRGRRRRTCGRCGCCR 5400
Indonesia-88045        TMMYYRTWCAAAAAAYYRRSRYYTYYCYKSCRRARSYTSRARCRRGRRRRTCGRCGCCR 5400
Indonesia-88065        TAMYYATWCAAAMTCYYRRSRYYKYYCYKSCRRARSYTSRARCRRRRRRRTCGGMCYYA 5400
                         *     *       .*   ** *. *    *    . .*..  *     *  **   .

Bhutan-09015           TGAWSTARAGYTYYGCYCRKMATATYSRTCTTACRRYCRYKYYAYTMWATTMATWCMRRG 5460
Bhutan-09024           TGAWSTARAGYTYYGCYCRKMATATYSRTCTTACRRTCRYKYYAYTMWATTMATWCMRRG 5460
Bhutan-09027           TGAWSTARAGYTYYGCYCRKMATATYSRTCTTACRRTCRYKYYAYTMWATTMATWCMRRG 5460
Bhutan-09030           TGAWSTARAGYTYYGCYCRKMATATYSRTCTTACRRTCRYKYYAYTMWATTMATWCMRRG 5460
Bhutan-09005           WRWWSWMGMSYAYYRYCYAGMRYCKYCGWYYYRYGAYSGTGTYTCYCTRCKARWWTAGRR 5460
Indonesia-88035        WRWWSWMGASYAYYRTCTAGMRYCKYCGWYYYRYGAYSGTGTYTCYCTRCKARWWTAGRR 5460
Indonesia-88045        WRWWGWMGASTAYYRTCTAGMRYCKYCGWYYYRYGAYSGTGTYTCYCTRCKARWWTAGRR 5460
Indonesia-88065        WRWWSWMGMSYAYYRTCTAGMRYCKYCGWYYYRYGAYSGTGTYTCYCTRCKARWWTAGRR 5460
                          *.     . :**       *  ..*.           .     *:       .    *   *

Bhutan-09015           CARAYCTWKKRTCRRKWYTGTTYGMYYRYWSRAARRRYYRYGARTCYGMCRCTSTWGRYT 5520
Bhutan-09024           CARAYCTWKKRTCRRKWYTGTTYRMYYRYWSRAARRRYYRYGARTCYGMCRCTSTWGRYT 5520
```

```
Bhutan-09027        CARAYCTWKKRTCRRKWYTGTTYRMYYRYWSRAARRRYYRYGARTCYGMCRCTSTWGRYT 5520
Bhutan-09030        CARAYCTWKKRTCRRKWYTGTTYRMYYRYWSRAARRRYYRYGARTCYGMCRCTSTWGRYT 5520
Bhutan-09005        CARRCYWTTKRCTRAKTYYSYKTRCYTAYTGAGRGAATYATRMGKYYAMYGSKSYTRACW 5520
Indonesia-88035     YRRRCYWTTKRTTRAKTYYSYKTRCYTAYTGAGRGAATYATRMGKYYAMYGSKSYTRACW 5520
Indonesia-88045     YRRRCYWTTKRTTRAKTYYSYKTRCYTAYTGAGRGAATYATRMGKYYAMYGSKSYTRACW 5520
Indonesia-88065     YRRRCYWTTKRCTRAKTYYSYKTRCYTAYTGAGRGAATYATRMGKYYAMYGSKSYTRACW 5520
                      *     .**  * * * . .   *  * . .     *     . *.*  ..*

Bhutan-09015        TWYGWRTSAKYRWCRSWKTAACAGKRYYRRGRCTTTMGCTYYRAYYRAGCTATCCRGMCT 5580
Bhutan-09024        TWYGWRTSAKYRWCRSWKTAACAGKRYYRRGRCTTTMGCTYYRAYTRAGCTATCCRGMCT 5580
Bhutan-09027        TWYGWRTSAKYRWCRSWKTAACAGKRYYRRGRCTTTMGCTYYRAYYRAGCTATCCRGMCK 5580
Bhutan-09030        TWYGWRTSAKYRWCRSWKTAACAGKRYYRAGRCTTKMSSTYYRAYYRAGCTATCCRGMCK 5580
Bhutan-09005        KWYCARYCRTCGTCACTGYGWYMGTRYTRRRATYWTCGCCTTRWCCRRRMYRYGARACYK 5580
Indonesia-88035     KWYCARYCRTCGTMACTGYGWTMCTGTTARRATYWTCGCCTTRWCCRRRMYRYGARACCT 5580
Indonesia-88045     KWYCARYCRTCGTMACTGYGWTMCTGTTRRGATYWTCGCCTTRWCCRRRMYRYGARACCT 5580
Indonesia-88065     KWYCARYCRTCGTCACTGYGWCMGTRYTRRRATYWTCGCCTTRWCCRRRMYRYGARACYK 5580
                    .**  * . .     .   .    .          . ..   *   *       .*.  .

Bhutan-09015        AYARTRYRWWAMYRWKAAYYYMACWRYRRWGSYYRMGRRYYMYTRAWRSRRTGYYGTYSC 5640
Bhutan-09024        AYARTRYRWWAMYRWKAAYYYMACWRYRRWGSYYRMGRRYYMYTRAWRSRRTGYYGTYSC 5640
Bhutan-09027        AYARTRYRWWAMYRWKAAYYYMACWRYRRWGSYYRMGRRYYMYTRAWRSRRTGYYGTYSC 5640
Bhutan-09030        AYARTRYRWWAMYRWKAAYYYMACWRYRRWGSYYRMGRRYYMYTRAWRSRRTGYYGTYSC 5640
Bhutan-09005        RCMRGGTGATMATGATRCYYCCTGWACGGWTCTCRCAGAYCMCCGRWRGAAYATTAKYSY 5640
Indonesia-88035     RCMRGGTGATMATGATRCYYCCTGWACGGWGCTCRCAGAYCMCCGRWRGAAYRTTAKYSY 5640
Indonesia-88045     RCMRGGTGATMATGATRCYYCCTGWACGGWGCTCRCAGAYCMCCGRWRGAAYATTAKYSY 5640
Indonesia-88065     RCMRGGTGATMATGATRCYYCCTGWACGGWTCTCRCAGAYCMCCGRWRGAAYATTAKYSY 5640
                       *           . .**  : *    * .  * .  * *    **.      ..**

Bhutan-09015        YCCCTAMCYYRACYRYRATCRKRGYGSMWKACCCAYRAKMCAMTTCCYAMMYCCTAYYRY 5700
Bhutan-09024        YCCCTAMCYYRACYRYRATCRKRGYGSMWKACCCAYRAKMCAMTTCCYAMMYCCTAYYRY 5700
Bhutan-09027        YCCCTAMCYYRACYRYRATCRKRGYGSMWKACCCAYRAKMCAMTTCCYAMMYCCTAYYRY 5700
Bhutan-09030        YCCCTAMCYYRACYRYRATCRKRGYGSMWKACCCAYRAKMCAMTTCCYAMMYCCTAYYRY 5700
Bhutan-09005        YTCYCTMSTCRWYYRCRMTYGGRRTASAATTYCCGYGATMCACYCTTCWMMCYYYWYYGT 5700
Indonesia-88035     YTSYCTMSTCGWYYRCRMTCRKRGTASAATTYYYGYGMTMYGCYCTTYWMMCYYYWYYGT 5700
Indonesia-88045     YTSYCTMSTCGWYYRCRMTCRKRGTASAATTYYYGYGMTMYGCYCTTYWMMCYYYWYYGT 5700
Indonesia-88065     YTCYCTMSTCRWYYRCRMKYGGRRTASAATTYCCGYGATMCACYCTTCWCCCYYYWYYGT 5700
                    * .  :*.     ** * .   *  .*  .:   .*  .* .              **

Bhutan-09015        CYTRGYYRRTSYGCMYKRTGRGYAYTRTAYTTASGYRCGYYAAGWSCSGRWCRCMYCAAS 5760
Bhutan-09024        CYTRGYYRRTSYGCMYKRTGRGYAYTRTAYTTASGYRCGYYAARWSCSGRWCRCMYCAAS 5760
Bhutan-09027        CYTRGYYRRTSYGCMYKRTGRGYAYTRTAYTTASGYRCGYYRAGWSCSGRWCRCMYCAAS 5760
Bhutan-09030        CYTRGYYRRTSYGCMYKRTGRGYAYTRTAYTTAKGYRCGYYAAGWSCSGRWCRCMYCAAS 5760
Bhutan-09005        STYRRYYAGYSTSAAYKRKSRSTRCYAWWYYYRSRTRYRCYAAGWSYGSGTYRGMTYRWS 5760
Indonesia-88035     SYYRRYYAGYSTSAAYKRKSRSTRCYAWWYYYRSRTRYRCYAAGWSYGSGTYRGMTYRWS 5760
Indonesia-88045     SYYRRYYAGYSTSAAYKRKSRSTRCYAWWYYYRKRTRYRCYARGWSYGSGTYRGMTYRWS 5760
Indonesia-88065     STYRRYYAGYSTSAAYKRKSRSTRCYAWWYYYRSRTRYRCYAAGWSYGSGTYRGMTYRWS 5760
                    .  * **   * .. ***..*.       *   .  *   *   ** ..   * *    *

Bhutan-09015        SKWCARYGSRWAAARWGYTATATGYYGKTGRTAMRMAYWCSTYWRYKKYMRRYTCRRACA 5820
Bhutan-09024        SKWCARYGSRWAAARWGYTATATGYYGKTGRTAMRMAYWCSTYWRYKKYMRRYTCRRACA 5820
Bhutan-09027        SKWCARYGSRWAAARWGYTATATGYYGKTGRTAMRMAYWCSTYWRYKKYMRRYTCRRACA 5820
Bhutan-09030        SKWCARYGSRWAAARWGYTATATGYYGKTGRTAMRMAYWCSTYWRYKKYMRRYTCRRACA 5820
Bhutan-09005        CTTYCACAGGWGMRGATYYMYRGKYCRTCKRCRCRMTCASSYYWRTGGCCARYWMRGRYR 5820
Indonesia-88035     CTTYCACAGGWGMRGATYYMYRGKYCRTCKRCRCRMTCASSYYWRTGGCCARYWMRGRTG 5820
```

```
Indonesia-88045      CTTYCACAGGWGMRGATYYMYRGKYCRTCKRCRCRMTCASSYYWRTGGCCARYWMRGRTG 5820
Indonesia-88065      CTTYCACAGGWGMRGATYYMYRGKYCRTCKRCRCRMTCASSYYWRTGGCCARYWMRGRTG 5820
                     ..  .  .. *.     *      *  .  *   **:  .* ***      **  *

Bhutan-09015         TRMWTYTTRWWYYYRKGKGYRGMGAYTCYKAWMYCMMSAKGRYMTGGGWTGGAYRGSKRA 5880
Bhutan-09024         TRMWTYTTRWWYYYRKGKGYRGMGAYTCYKAWMYCMMSAKGRYMTGGGWTGGAYRGSKRA 5880
Bhutan-09027         TRMWTYTTRWWYYYRKGKGYRGMGAYTCYKAWMYCMMSAKGRYMTGGGWTGGAYRGSKRA 5880
Bhutan-09030         TRMWTYTTRWWYYYRKGKGYRGMGAYTCYKAWMYCMMSAKGRYMTGGGWTGGAYRGSKRA 5880
Bhutan-09005         CGCAACYWRAWTYCRKSTTCRKMRMYCYCGWTCTSACCWKTRYMYACRTYKRMTAGSKGR 5880
Indonesia-88035      CGCAACYWRAWTYCRKSTTCRKMRMYCYCGWTCTSACCWKTRYMYACRTYKRMTAGSKGR 5880
Indonesia-88045      CGCAACYWRAWTYCRKSTTCRKMRMYCYCGWTCTSACCWKTRYMYACRTYKRATATSKGR 5880
Indonesia-88065      CGCAACYWRAWTYCRKSTTCRKMRMYCYCGWTCTSACCWKTRYMYACRTYKRMTACSKGR 5880
                         :   * * * **..  * *  *        .  . * *** .          **

Bhutan-09015         YWTMKRTMWCGRRKRCYRGKGMCCWSMTGRAWTWGCKWTTAWGRGYRYAWRAAMWCRARC 5940
Bhutan-09024         YWTMKRTMWCGRRKRCYRGKGMCCWSMTGRAWTWGCKWTTAWGRGYRYAWRAAMWCRARC 5940
Bhutan-09027         YWTMKRTMWCGRRKRCYRGKGMCCWSMTGRAWTWGCKWTTAWGRGYRYAWRAAMWCRARC 5940
Bhutan-09030         YWTMKRTMWCGRRKRCYRGKGMCCWSMTGRAWTWGCKWTTAWGRGYRYAWRAAMWCRARC 5940
Bhutan-09005         CWYCGGWCWYSRRKRYYRRGSMMYTGAYARRWCWRTKWAATARGRYAYRTRWRCAMGCGS 5940
Indonesia-88035      CWYCGGWCWYSRRKRYYRRKSMMYTGAYARRWCWRTKWAATARGRYAYRTRWRCAMGCGS 5940
Indonesia-88045      CWYCGGWCWYSRRKRYYRRKSMMYTGAYARRWCWRTKWAATARGRYAYRTRWRCAMGCGS 5940
Indonesia-88065      CWYCGGWCWYSRRKRYYRRGSMMYTGAYARRWCWRTKWAATARGRYAYRTRWRCAMGCGS 5940
                      *      *.****  **  .*   .  .* * *  **:::    * *  *      . .

Bhutan-09015         TAGTRGTKRCWRTYGATRTYAGRGTRRYKYWRTAYTRRRYTRMSYCTRTCYRGAAMCGYG 6000
Bhutan-09024         TAGTRGTKRCWRTYGATRTYAGRGTRRYGTARTAYTRRRYTRMSYCTRTCYRGAAMCGYG 6000
Bhutan-09027         TAGTRGTKRCARTYGATRTYAGRGTRRYKYWRTAYTRRRYTRMSYCTRTCYRGAAMCGYG 6000
Bhutan-09030         TAGTRGTKRCWRTYGATRTYAGRGTRRYKYWRTAYTRRRYTRMSYCTRTCYRGAAMCGYG 6000
Bhutan-09005         CRRYRRYKASTRWTARYRYYMRGAYARYKTWRTATGAARTWGAGYSWGYYCGRRRCMRTR 6000
Indonesia-88035      CRRYRRYKASTRWTARYRYYMRGAYARYGTARTATGAARTWGAGYSWGYYCGRRRCMRTR 6000
Indonesia-88045      CRRYRRYKASTRWTARYRYYMRGAYARYGTARTTTGAARTWGAGYSWGYYCGAGRCMRTR 6000
Indonesia-88065      CRRYRRYKASTRWTARYRYYMRGAYARYGTARCATGAARTWGAGYSWGYYCGAGRCMRTR 6000
                         *  * . *  .  * *   .  **   * :    *    .*.

Bhutan-09015         WYSWMKTWAKYMWSTKMTWYARYWARYTCCWCTRCRMWGMTTTTMRGCAAAAASWACMYR 6060
Bhutan-09024         WYSWMKTWAKYMWSTKMTWYAAYWARYTCCWCTRCRMWGMTTTTMRGCAAAAASWACMYR 6060
Bhutan-09027         WYSWMKTWAKYMWSTGATWYAAYWARYTCCWCTRCRMWGMTTTTMRGCAAAAASWACMYR 6060
Bhutan-09030         WYSWMKTWAKYMWSTGATWYAAYWARYTCCWCTRCRMWGMTTTTMRGCAAAAASWACMYR 6060
Bhutan-09005         WCGTCKYTTGYAWSYTCYAYGACTTRYWYYTCYRMGCWRCCYKYAASYMWRCRSWWMATS 6060
Indonesia-88035      WCGTCKYTTGYAWSYTCYAYGRCTTRYWYYTMYRMGCWRCCYKYAASYMWRCRSWWMATS 6060
Indonesia-88045      WCGTCKYTTGYAWSYTCYAYGRCTTRYWYYTMYRMGCWRCCYKYAASYMWRCRSWWMATS 6060
Indonesia-88065      WCGTCKYTTGYAWSYTCYAYGRCTTRYWYYTMYRMGCWRCCYKYAASYMWRCRSWWMATS 6060
                     * .  *  : * **     *.   :**      *   *    .   .    . **

Bhutan-09015         YRMYWAYRCRSYYYMWYAAMMYCGAYYSCRMYCTRTWGGTRYYGYYACGGTGGYASRCYW 6120
Bhutan-09024         YRMYWAYRCGSYTCMWYAAMMYCGAYYSCRMYCTRTWGGTRYYGYYACGGTGGYASRCYW 6120
Bhutan-09027         YRMYWAYRCGSYYYMWYAAMMYCGAYYSCRMYCTRTWGGTRYYGYYACGGTGGYASRCYW 6120
Bhutan-09030         YRMYWAYRCGSYTCMWYAAMMYCGAYYSCRMYCTRTWGGTRYYGYYACGGTGGYASRCYW 6120
Bhutan-09005         CRMYWWTRYGSTTCMWYWRMCYSRWTTGMRMCYCRCAARCACTRTYGTRGTAKTRSGYTW 6120
Indonesia-88035      CRMYWWTRYGSYTCMWYWAMCYSRWTTGCRMCYCRCAARCACTRTYGTRSYAKTRSGYTW 6120
Indonesia-88045      CRMYWWTRYGSYTCMWYWRMCYSRWTTGCRMCYCRCAARCACTRTYGTRSYAKTRSGYTW 6120
Indonesia-88065      CRMYWWTRYGSTTCMWYWRMCYCRTTTGMRMCYCRCAARCACTRYYGTRSYAKTRSGYTW 6120
                      ****  *  *   ***  * *.    . **   *  .       *.  . .   *   *
```

```
Bhutan-09015         YATAGGSGYTGACRRGCGTKWKKTWYYWCGMGKYYTRMRTMRTACGAAGRYGRYGTGYCR 6180
Bhutan-09024         YATAGGSGYTGACRRGCGTKWKKTWYYWCGMGKYYTRMRTMRTACGAAGRYGRYGTGYCR 6180
Bhutan-09027         YATAGGSGYTRACRRGCGTKWKKTWYYWCGMGKYYTRMRTMRTACGAAGRYGRYGTGYCR 6180
Bhutan-09030         YATAGGSGYTGACRRGCGTKWKKTWYYWCGMGKYYTRMRTMRTACGAAGRYGRYGTGYCR 6180
Bhutan-09005         YGYRRGSRCWAWTAGKTRYTTGKYACTAYRARTYCYGAAYCAWGGRRGRGCRRYRYRYTA 6180
Indonesia-88035      YGYRAKSRCWAWTAGKTGYTTGKYACTAYRARTYCCGAAYCAWGGRRGRGCGRYRYRYTA 6180
Indonesia-88045      YGYRAKSRCWAWTAGKTRYTTGKYACTACGARTYCYGAAYCAWGGRRGRGCGRYGTRYTA 6180
Indonesia-88065      YGYRRGSRCWAWTAGKTRYTTGKYACTAYRARTYCYGAAYCAWGGRRGRGCGRYRYRYTA 6180
                     *.    *            .  *         .*         .   .    **   *

Bhutan-09015         KYRWGRAAGKMRYWYGWGYKRWCSRYCAWYWTYRWCCTMCRYYCRTKYRKMCAMTRAWTM 6240
Bhutan-09024         KYRWGRAAGKMRYWYGWGYKRWCSRYCAWYWTYRWCCTMCRYYCRTKYRKMCAMTRAWTM 6240
Bhutan-09027         KYRWGRAAGKMRYWYGWGYKRWCSRYCAWYWTYRWCCTMCRYYCRTKYRKMCAMTRAWTM 6240
Bhutan-09030         KYRWGRAAGKMRYWYGWGYKRWCSRYCAWYWTYRWCCTMCRYYCRTKYRKMCAMTRAWTM 6240
Bhutan-09005         TCATYRGWKKCGCTCAARYKRTYCGYMRTYWKCGAYSTCTGCTSRYGCRGCTGCAGRTTC 6240
Indonesia-88035      TCATYRGWKKCGCTCAARYKRTYCGYMAWYWKCGAYSKCTGCTCAYGCRGCTGCAGRTYC 6240
Indonesia-88045      TCATYRGWKKCGCTCAARYKRTYCGYMAWYWKCGAYSKCTGCTCAYGCRGCTGCAGRTYC 6240
Indonesia-88065      TCATYRGWKKCGCTCAARYKRTYCGYMRTYWKCGAYSKCTGCTCAYGCRGCTGCAGRWYC 6240
                     .    *.  *     .  ***  . *   **.    ..     .    *   . :

Bhutan-09015         AYARGGGWWGRTAWAYYWYYYYWWYAGYWSTRYYMCARCSWTCRAMYAMTTCCAWKAACA 6300
Bhutan-09024         AYARGGGWWGRTAWAYYWYYYYWWYAGYWSTRYYMCARCSWTCRAMYAMTTCCAWKAACA 6300
Bhutan-09027         AYAAGGGWWGRTAWAYYWYYYYWWYAGYWSTRYYMCARCSWTCRAMYAMTTCSAWKAACA 6300
Bhutan-09030         AYARGGGWWGRTAWAYYWYYYYWWYAGYWSTRYYMCARCSWTCRAMYAMTTCCAWKAACA 6300
Bhutan-09005         AYGGRSKAWRACRAWCYWTCYCAWYAGCTGYGTYMYRRYCAGTAMCCRMCATCTWGMRTG 6300
Indonesia-88035      AYGGGSKAWRACRAWCYWTCYCAWYRRCTGYGTCMYRRYSWGTAMCCRMCATCTWKARTG 6300
Indonesia-88045      RYGGGSKAWRACRAWCYWTCYCAWYRRCTGYGTCMYRRCSWGTAMCCRMCATCTWKARTG 6300
Indonesia-88065      RYGGGSKAWRACRAWCYWTCYCAWYRRCTGYGTYMYRRYCAGTAMCCRMCATCTWGMRTG 6300
                      *.  .  *       **  *  **    .    *  * .        * : .:*    .

Bhutan-09015         WKAATRRAWGCMGYYYYWKSRAYGYWAMYTTGGYRRGCRKWCKCKRYRYTWGASGGMRGR 6360
Bhutan-09024         WKAATRRAWGCMGYYYYWKSRAYGYWAMYTTGGYRRGCRKWCKCKRYRYTWGASGGMRGR 6360
Bhutan-09027         WKAATRRAWGCMGYYYYWKSRAYGYWAMYTTGGYRRGCRKWCKCKRYRYTWGASGGMRGR 6360
Bhutan-09030         WKAATRRAWGCMGYYYYWKSRAYGYWAMYTTGGYRRGCRKWCKCKRYRYTWGASGGMRGR 6360
Bhutan-09005         WGWRYGAAWSYMRTCTYAGCRGYRYWRCYYYKRTARRCRKWYGMKGTGSYAARGRAARSA 6360
Indonesia-88035      WGWRYGARWSYMRTCTYAGSRGYRYWRCYYYKRTARRYRKWYGMKGTGSYARRGRRARSA 6360
Indonesia-88045      WGWRYGAAWSYMRTCTYAGSRGYRTTACYYYKRTARRYRKWYGMKGTGSYARRGRRARSA 6360
Indonesia-88065      WGWRYGAAWSYMRTCTYAGCRGYRTTACYYYKRTARRCRKWYGMKGTGSYAARGRAARSA 6360
                     *       *. *    *  .*.*     *      *  ***   *        .   *.

Bhutan-09015         YATMAKCRGCWGATRRCSYAYGAKTCRCGYGRRRCAATRGAGTTRRRRKKGATYCRYTSR 6420
Bhutan-09024         YATMAKCRGCWGATRRCSYAYGAKTCRCGYGRRRCAATRGAGTTRRRRKKGATYCRYTSR 6420
Bhutan-09027         YATMAKCRGCWGATRRCSYAYGAKTCRCGYGRRRCAATRGAGTTRRRRKKGATYCRYTSR 6420
Bhutan-09030         YATMAKCRGCWGATRRCSYAYGAKTCRCGYGRRRCAATRGAGTTRRRRKKGATYCRYTSR 6420
Bhutan-09005         TGGCWGSARYAKMARRTGCGYTRTYYATRYAGGGTRRYGATTKGGAGGGGKRWYGGTAGG 6420
Indonesia-88035      TGGCWGSARYAKMARRTGCGYTRTYYATRYAGGGTRRYGATTKGGAGGGGKRWYGGTAGG 6420
Indonesia-88045      TGGCTGSARYAKMARRTGCGYTRTYYATRYAGGGTRRYGATTKGGAGGGGKRWYGGTAGG 6420
Indonesia-88065      TGGCTGSARYAKMARRTGCGYTRTYYATRYAGGGTRRCGATTKGGAGGGGKRWYGGTAGG 6420
                      .    .      :** . .*  .     *.        .: .          *   :.

Bhutan-09015         AKTCYRACCKTACWMTYTYTRGRRWRAGTMKTACCRGMTYCWTAGAYAWTSGRYRKARKY 6480
Bhutan-09024         AKTCYRACCKTACWMTYTYTRGRRWRAGTMKTACCRGMTYCWTAGAYAWTSGRYRKARKY 6480
Bhutan-09027         AKTCYRACCKTACWMTYTYTRGRRWRAGTMKTACCRGMTYCWTAGAYAWTSGRYRKARKY 6480
Bhutan-09030         AKTCYRACCKTACWMTYTYTRGRRWRAGTMKTACCRGMTYCWTAGAYAWTSGRYRKARKY 6480
```

```
Bhutan-09005          MTKYYRGYYKYRSAACTCTYASRRAGGRWAGYWSTARMKYTAYGRRYWAYGRRYAGRAGY 6480
Indonesia-88035       MTKCYRGYYKYRSAACTCTYASRGAGGRWAGYWSTARMKYTAYGRRYWAYGRRYAGRAGY 6480
Indonesia-88045       MTKCYRGYYKYRSAACTCTYASRGAGGRWAGYWSTARMKYTAYGRGTWAYGRRYAGRAGY 6480
Indonesia-88065       MTKYYRGYYKYRSAACTCTYASRRAGGRWAGYWSTARMKYTAYGRGTWAYGRRYAGRAGY 6480
                       .. **.  *  .        .*   .      .   *.*   .      . **     *

Bhutan-09015          SYYCSTRWRMRKGGMAGGGCARWTTRMRRCYYRTYTYYKAGAGWRWRAGCGCRAAYKARG 6540
Bhutan-09024          SYYCSTRWRMRKGGMAGGGCARWTTRMRRCYYRTYTYYKAGAGWRWRAGCGCRAAYKARG 6540
Bhutan-09027          SYYCSTRWRMAGGGMAGGGCARWTTRMRRCYYRTYTYYKAGAGWRWRAGCGCRAAYKARG 6540
Bhutan-09030          SYYCSTRWRMRKGGMAGGGCARWTTRMRRCYYRTYTYYKAGAGWRWRAGCGCRAAYKARG 6540
Bhutan-09005          CTTYSKGAGMRGRTMMRTKYRAAYYAAAATCCAYCKYTKMSRRAAARRATASGWMYGRAA 6540
Indonesia-88035       CTTYSKGAGMGGRTCCATKYRACYYGAAATCCAYCKYTKMSRRAAARRATASGWMYGRAA 6540
Indonesia-88045       CTTYSKGAGMGGRTCCATKYRACYYGAAATCCAYCKYTKMSRRAAARRATASGWMYGRAA 6540
Indonesia-88065       CTTYSKGAGMRGRTMMRTKYRAAYYAAAATCCAYCKYTKMSRRAAARRATASGWMYGRAA 6540
                      .   *.   *                         .* * .     * . ..   *   .

Bhutan-09015          CAWTACRGRTCAACCYRRYRWYMKGCTKCTCTTTTKWKYYYCSGWCYTYGATRRMCRRWM 6600
Bhutan-09024          CAWTACRGRTCAACCYRRYRWYMKGCTKCTCTTTTKWKYYYCSGWCYTYGATRRMCRRWM 6600
Bhutan-09027          CAWTACRGRTCAACCYRRYRWYMKGCTKCTCTTTTKWKYYYCSGWCYTYGATRRMCRRWM 6600
Bhutan-09030          CAWTACRGRTCAACCYRRYRWYMKGCTKCTCTTTTKWKYYYCSGWCYTYGATRRMCRRWM 6600
Bhutan-09005          AWACGAARRYYRTAMCRATGTCCKRMWTYYYAWYWKWKTYTYSSTMCYYSRAGGAYRRWA 6600
Indonesia-88035       AWACGAARRTCATAMCRATGTCCKRMWTYYYAWYWKWKTYTYSSTMCYYSRAGGAYAGWA 6600
Indonesia-88045       AWACGAARRTCATAMCRATGTCCKRMWTTYYAWYTTWKTYTYSSTMCYYSRAGGAYRRWA 6600
Indonesia-88065       AWACGAARRYYRTAMCRATGTCCKRMWTYYYAWYWKWKTYTYSSTMCYYSRAGGAYRRWA 6600
                      .   ..  *   :.  *      *   .   :   .** *  *.    *. :      *

Bhutan-09015          CAYTSYRYCGRYRGKMARSYATKSSRCKSGRTCYMKYMGTYGRYSRYATAYKRYRGRGYS 6660
Bhutan-09024          CAYTSYRYCGRYRGKMARSYATKSSRCKSGRTCYMKYMGTYGRYSRYATAYKRYRGRGYS 6660
Bhutan-09027          CAYTSYRYCGRYRGKMARSYATKSSRCKSGRTCYMKYMGTYGRYSRYATAYKRYRGRGYS 6660
Bhutan-09030          CAYTSYRYCGRYRGKMARSYATKSSRCKSGRTCYMKYMGTYGRYSRYATAYKRYRGRGYS 6660
Bhutan-09005          YRYWSCRTMRAYARKMWRGCWKGGSRYTSAGCYTCGYMSCTKRTGGCRYRYKGCATARTS 6660
Indonesia-88035       YGYWSCRTMRAYARKMWRGCWKGGSRYTSAGCYTCGYMSCTKRTGGCRYRYKGCATARTS 6660
Indonesia-88045       YRYWSCRTMRAYARKMWRGCWKGGSRYTSAGCYTCGYMSCTKRTGGCRYRYKGCATARTS 6660
Indonesia-88065       YRYWSCRTMRAYARKMWRGCWKGGSRYTSAGCYTCGYMSCTKRTGGCRYRYKGCATARTS 6660
                        * * *    *  ** *.  . .** .*.      **.   * .     **       *

Bhutan-09015          ARGKARRGWGAARACRSRGGTRSCAYWGRYYRYYYKYYYGMYMKRCYWYYTRWRRRKKCA 6720
Bhutan-09024          ARGKARRGWGRMRACRSRGGTRSCAYWGRYYRYYYKYYYGMYMKRCYWYYTRWRARKKCA 6720
Bhutan-09027          ARGKARRGWGRMRACRSRGGTRSCAYWGRYYRYYYKYYYGMYMKRCYWYYTRWRRRKKCA 6720
Bhutan-09030          ARGKARRGWGRMRACRSRGGTRSCAYWGRYYRYYYKYYYGMYMKRCYWYYTRWRRRKKCA 6720
Bhutan-09005          CAKGRGAKTRAARATRCRTAYRSAGTARRYYGYTTTCCYRATMTATYAYYKRAARRTKYC 6720
Indonesia-88035       CAKGRGAKTRAARMTRCRTAYRSAGTARRYYGYTTTCCYRATMTATYAYYKRAAARTKYC 6720
Indonesia-88045       CAKGRGAKTGAARMTRCRTAYRSAGTARRYYGYTTTCCYRATMTATYAYYKRAARRTKYC 6720
Indonesia-88065       CAKGRGAKTRAARMTRCRTAYRSAGTARRYYGYTTTCCYRATMTATYAYYKRAARRTKYC 6720
                      .           *  *.* . **..   *** *  .  *   *.  * **.*   *.* .

Bhutan-09015          CYRKMTAKAMARYKYGKKCMTTSMYATYTACCKYAWAAGTRCMMGYARMKGCYSMYAGKR 6780
Bhutan-09024          CYRKMTAKAMARYKYGKKCMTTSMYATYTACCKYAWAAGTRCMMGYARMKGCYSMYAGKR 6780
Bhutan-09027          CYRKMTAKAMARYKYGKKCMTTSMYATTTACCKYAWAAGTRCMMGYARMKGCYSMYAGKR 6780
Bhutan-09030          CYRKMTAKAMARYKYGKKCMTTSMYATYTACCKYAWAAGTRCMMGYARMKGCYSMYAGKR 6780
Bhutan-09005          ATGGMYWKRCAAYKCATTAMCWCATMCTYWSTKYRARRRYAMCCRCMRATRSYGACMCTA 6780
Indonesia-88035       ATGKMYWKRCRAYTCATTAMCWCATMCTYWSTKYRARRRYAMCCRCMRATRSYSMCMSTA 6780
Indonesia-88045       ATGKMYWKRCRAYTCATTAMCWCATMCTYWSTKYRARRRYAMCCRCMRATRSYSMCMSTA 6780
Indonesia-88065       ATGKMYWKRCAAYKCATTAMCWCATMCTYWSTKYRARRRYAWCCRCMRATRSYGACMCTA 6780
```

```
                     .   *  *    *. ....*  .       . **             * . .*.    .

Bhutan-09015         TMRKGATGTWCSWAGKKWYRMSYGGGTGTTYWYWYRRAAGGRRSSTTTRMWCATYAYSYG 6840
Bhutan-09024         TMRKGATRTWCSWAGKKWYRMSYKGGTGTTYWYWYRRAAGGRRSSTTTRMWCATYAYSYG 6840
Bhutan-09027         TARKGATGTWCSWAGKKWYRMSYKGGTGTTYWYWYRRAAGGRRSSTTTRMWCATYACSYG 6840
Bhutan-09030         TMRKGAWGYWCSWAGKKWYRMSYKGGTGTTYWYWYRRAAGGRRSSTTTRMWCATYACSYG 6840
Bhutan-09005         YCRTGAWGTTYCARKKTATAACTGRKTSYYYWYWYRRRRKRGRSSWWWRCTYRYCTYGTR 6840
Indonesia-88035      YCRTGATGTTCCARKTKATGACYGRKCSYYYWYWYRRRRKRGRSSWWWRCTYRYCTYGTR 6840
Indonesia-88045      YCRTKWWGTTCCARKTKATGACYGRKCSYYYWYWYRRRRGRGRSSWWWRCTYATCTYGTR 6840
Indonesia-88065      YCRTKWTGTTYCARKKTATAACTGRKTSYYYWYWYRRRRKRGRSSWWWRCTYRYCTYGTR 6840
                       *.       .   ..    .     .  *******     ***   *      : .

Bhutan-09015         ARRATATAKYRWGMKKGRTGTYYGMYYRAGTCCYRTACKYRTSKRRTCTARRYGWTKRGT 6900
Bhutan-09024         ARRATATAKYRWGMKKGRTGWYYGMYYRAGTCCYRTACKYRTSKRRTCTARRYGWTKRGT 6900
Bhutan-09027         ARRATATAKYRWGMKKGRTGWYYGMYYRAGTCCYRTACKYRTSKRRTCTARRTGWTKRGT 6900
Bhutan-09030         ARRATATAKYRWGMKKGRTGWYYGMYYRAGTCCYRTACKYRTSKRRTCTARRTGWTKRGT 6900
Bhutan-09005         RRRMYRWRTTGWRMGTTGCATCYKCCYGGTKMYCAYRMTYAYSKAAKYCAAGCRAYTAGK 6900
Indonesia-88035      RRRMYRWRTTGWRMGTTGCATCYKCCYGATKMYCAYRMTTAYSKAAKYTTAGYRAYKRRK 6900
Indonesia-88045      RRRMYRWRTTGWRMGTTGCATCYKCCYGATKMYCAYRMTTAYSKAAKYTTAGYRAYKRRK 6900
Indonesia-88065      RRRMYRWRTTGWRMGTTGCATCYKCCYGGTKMYCAYRMTYAYSKAAKYCAAGCRAYTAGK 6900
                      **     .  * * .   .  *   * . .       .   **  .  :      .  .

Bhutan-09015         YRRAYTMSWYKKYKMYCCGYTWYKCTCCYAKTGTRARRWGTRCRYRKGWTTAKYTTWYYW 6960
Bhutan-09024         YRRAYTMSWYKKYKMYCCGYTWYKCTCCYAKTGTRARRWGTRCRYRKGWTTAKYTTWYYW 6960
Bhutan-09027         YRRAYTMSWYKKYKMYCCGYTWYKCTCCYAKTGTRARRWGTRCRYRKGWTTAKYTTWYYW 6960
Bhutan-09030         YRRAYTMSWYKKYKMYCCGYTWYKCTCCYAKTGTRARRWGTRCRYRKGWTTAKYTTWYYW 6960
Bhutan-09005         TAARCYACTTAGCGCCYGATAACKTYMTCWKCAYGRGGARGGARYWTKTYKRGTYYWYYT 6960
Indonesia-88035      TAARCYACTTAGCGCCYGATAACKTYCTCAKCATGRGRARGGARYWTGTYGGGTYYWYYT 6960
Indonesia-88045      TAARCYACTTAGCGCCYGATAACKTYCTCAKCATGRGRARGGARYWTGTYGGGTYYWYYT 6960
Indonesia-88065      TAARCYACTTAGCGCCYGATAACKTYMTCWKCAYGRGGARGGARYWTKTYKRGTYYWYYT 6960
                            .          . :  *      * .         .** .         ***

Bhutan-09015         AKRTTTGRMTTTKATGRYYRTGCSRYKTKCYYTAMRYRACTWTAAGTGTSAYYTAWTCAY 7020
Bhutan-09024         AKRTTTGRMTTTKATGRYYRTGCSRYKTKCYYTAMRYRACTWTAAGTGTSAYYTAWTCAY 7020
Bhutan-09027         AKRTTTGRMTTTKATGRYYRTGCSRYKTKCYYTAMRCGACTWTAAGTGTSAYYTAWTCAY 7020
Bhutan-09030         AKRTTTGRMTTTKATGRYYRTGCSRYKTKCYYTAMRYRACTWTAAGTGTSAYYTAWTCAY 7020
Bhutan-09005         RGGYCYAGCAYWTMCTGTYRWRYGACGYTYCYYGAGCGMSCWYMWRYTYSRCYKWWYAGC 7020
Indonesia-88035      RGGYCYAGCAYWTMCTGTYRWRYGACGYTYCYYGAGCGMSCWYMWRYTYSRCYKWWYAGC 7020
Indonesia-88045      RGGYCYAGCAYWTMCTGTYRWRYGACGYTYCYYGAGCGMSCWYMWRYTYSRCYKWWYAGC 7020
Indonesia-88065      RGGYCYAGCAYWTMCTGTYRWRYGACGYTYCYYGAGCGMSCWYMWRYTYSRCYKWWYAGC 7020
                           .  :  .     **   .    .  * .     . *       *  *. * ..

Bhutan-09015         YAGRKRTCGMTWYGWCYYMRKTAMRYKMYRCTTWKCKCMMARYKMSYRMYYGRRATYGGG 7080
Bhutan-09024         YAGRKRTCGMTWYGWCYYMRKTAMRYKMYRCTTWKCKCMMARYKMSYRMYYGRRATYGGG 7080
Bhutan-09027         YAGRKRTCGMTWYGWCYYMRKTAMRYKMYRCTTWKCKCMMARYKMSYRMYYGRRATYGGG 7080
Bhutan-09030         YAGRKRTCGMTWYGTCYYMRKTAMRYKMYRCTTWKCKCMMARYKMSYRMYYGRRATYGGG 7080
Bhutan-09005         CRRGTGGYKCYACRWYTCMRKWRAGCGACGGAYTKYTYMMRAYKASCACYYARRRYTAKR 7080
Indonesia-88035      CRRGTGGYKCYACRWYTCMRKWRAGCGACGGAYTKYTYMMRAYKASCACYYARRRYTAKR 7080
Indonesia-88045      CRRGTGGYKCYACRWYTCMRKWRAGCGACGGAYTKYTYMMRAYKASCACYYARRRYTAKR 7080
Indonesia-88065      CRRGTGGYKCYACRWYTCMRKWRAGCGACGGAYTKYTYMMRAYKASCACYYARRRYTAKR 7080
                         .             ***          :  * . **  ** *   **.**   .

Bhutan-09015         WYWGMTRTRTAAGYAYACTKACRCRGTTCMWRRARCYGRRKGYMGYYTRRTAAMATYAAM 7140
Bhutan-09024         WYWGMTRTRTAAGYAYACTKACRCRGTTMMWRRARCYGRRKGYMGYYTRRTAAMATYAAM 7140
```

```
Bhutan-09027         WYWGMTRTRTAAGYAYACTKACRCRGTTMMWRRARCYGRRKGYMGYYTRRTAAMATYAAM 7140
Bhutan-09030         WYWGMTRTRTAAGYAYACTKACRCRGTTMMWRRARCYGRRKGYMGYYTRRTAAMATYAAM 7140
Bhutan-09005         ACTRCCGYAYRRRTRCMYYKWSGYGRGYACAGAMAACAAAGRTCRCTYGGCRGARWCGWC 7140
Indonesia-88035      ACTRCCGYAYRRRYRYMYYKWSGYGRGYACAGAMAACAAAGRTCRCTYGGCRGARWCGAC 7140
Indonesia-88045      ACTRCCGTAYRRRYRYMYYKWSGYGRGYACAGAMAACAAAGRTCRCTYGGCRGARWCGWC 7140
Indonesia-88065      ACTRCCGYATRRRTRCMYYKWSGYGRGYACAGAMAACAAAGRTCRCTYGGCRGARWCGWC 7140
                                        * .             . .              .    .

Bhutan-09015         AAGTGRRYMKRGAAYRRGGTTATWCYGYAKCAWGYCRMRYCAYRTTTTYGACCYCYRMTA 7200
Bhutan-09024         AARWGRRYMKRGAAYRRGGTTATWCYGYAKCAWGYCGMRYCAYRTTTTYGACCYYYRMTA 7200
Bhutan-09027         AARWGRRYMKRGAAYRRGGTTATWCYGYAKCAWGYCRMRYCAYRTTTTYGACCYYYRMTA 7200
Bhutan-09030         AARWGRRYMKRGAAYRRGGTTATWCYGYAKCAWGYCRMRYCAYRTTTTYGACCYCYRMTA 7200
Bhutan-09005         GRRTKAGYMKRSMRCAATTYWMYWYTAYWTYRARYTRCRYYWTAYYWYTSRYTTCCRMKW 7200
Indonesia-88035      GRRTKAGYMKRSMRCAATTYWMYWYTAYWTYRARYTRCRYYWTAYYWYTSRYTTCCRMKW 7200
Indonesia-88045      GRRTKAGYMKRSMRCAATTYWMYWYTAYWTYRARYTRCRYYWTAYYWYTSRYTTCCRMKW 7200
Indonesia-88065      GRRTKAGYMKRSARCAATTYWMYWYTAYWTYRARYTRCRYYWTAYYWYTSRYTTCCRMKW 7200
                     .      ****.           *  .* .    *   **         .      **.

Bhutan-09015         GKRYRTASRKRKTMRYKGYYTKCCYYWYYRTGYATACSWGTWGYAAGGAAMMKGRAYCRM 7260
Bhutan-09024         GKRYMWASRKRKTMRYKGYYTKCCYYWYYRTGYRTACSWGTWGYAAGGAAMMKGRAYCRM 7260
Bhutan-09027         GKRYAWASRKRKTMRYKGYYTKCCYYWYYRTGYRTACSWGTWGYAAGGAAMMKGRAYCRM 7260
Bhutan-09030         GKRYRTASRKRKTMRYKGYYTKCCYYWYYRTGYRTACSWGTWGYAAGGAAMMKGRAYCRM 7260
Bhutan-09005         RKRYATMCGTAYKAAYKRTYCTMYCYATTAYKCAKRTSARYTKTWRRARRCMKSRWTYRC 7260
Indonesia-88035      RKRYRTACGTAYKAAYKRTYCTMYCYATTAYKCAKRTSARYTKTWRRARRCMKSRWTYRC 7260
Indonesia-88045      RKRYRTACGTAYKAAYKRTYCTMYCYATTAYTCAKRTSAGTTKTWRRARRCMKSRWTYRC 7260
Indonesia-88065      RKRYATACGTAYKAAYKRTYCTMYCYATTAYKCAKRTSARYTKTWRRARRCMKSRWTYRC 7260
                      ***   . .  .  **  * .   *        .  *         .   **.*   *

Bhutan-09015         RRCCSWRRYWSKYGTSSATKMTYRYWKWWYWRYCGRMRRTTYKRRRYMRMCKRRYARKCY 7320
Bhutan-09024         RRCCSWRRYWSKYGTSSATKMWYRYWKWWYWRYCGRMRRTTYKRRRYMRMCKRRYARKCY 7320
Bhutan-09027         RRCCSWRRYWSKYGTSSATKMWYRYWKWWYWRYCGRMRRTTYKRRRYMRMCKRRYARKCY 7320
Bhutan-09030         RRCCSWRRYWSKYGTSSATKMWYRYWKWWYWRYCGRMRRTTYKRRRYMRMCKRRYARKCY 7320
Bhutan-09005         RRYYGARGCASTYRCSSTCGMTCGCAKATCTGCARRCRRYYTKRAACARMYKRRYRAKCY 7320
Indonesia-88035      RRYYGARGCASTYRCSGTCGMTCGCAKATCTGCARRCRRYYTKRAACARMYKRRYRAKYY 7320
Indonesia-88045      RRYYGARGCASTYRCSGTCGMTCGCAKATCTGCARRCRRYYTKRAACARMYKRRYRAKCY 7320
Indonesia-88065      RRYYGARGCASTYRCSSTCGMTCGCAKATCTGCARRCRRYYTKRAACARMYKRRYRAKCY 7320
                     **  . *   *.*  *.:  *     *      . * **   **    ** ****  * *

Bhutan-09015         STCGRTGYSKYGRYRAATCTMRGSATKATAARYCRCTYRGTAWTYGCRRWGYYMCYRTSA 7380
Bhutan-09024         STCGRTGYSKYGRYRAATCTMRGSATKATAARYCRCTYRGTAWTYGCRRWGYYMCYRTSA 7380
Bhutan-09027         STCGRTGYSKYGRYRAATCTMRGSATKATAARYCRCTYRGTAWTYGCRRWGYYMCYRTSA 7380
Bhutan-09030         STCGRTGYSKYGRYRAATCTMRGSATKATAARYCRCTYRGTAWTYGCRRWGYYMCYRTSA 7380
Bhutan-09005         SKSKAYRYCGCRRYAGWYYWCGASCYKGGRRRTYRSKCGKYMWYTCYGGTKTYMTTGKSM 7380
Indonesia-88035      SKSKAYRYCGCRRYAGWYYWCGASCYKGGRRRTYRSKCGKYMWYTCYGGTKTYMTTGKSM 7380
Indonesia-88045      SKSKAYRYCGCRRYAGWYYWCGASCYKGGRRRTYRSKCGKYMWYTCYGGTKTYMTTGKSM 7380
Indonesia-88065      SKSKAYRYCGCRRYAGWYYWCGASCYKGGRRRTYRSKCGKYMWYTCYGGTKTYMTTGKSM 7380
                     *..    *.   ** .      .*. *.   *  *..     *         **   .*

Bhutan-09015         GAGRCRAYCMWYKACAATKGGCACYWRSYSGCCRAYGTSAMMTRTCTTATATMGYGTGAY 7440
Bhutan-09024         GAGRCRAYCMWYKACAATKGGCACYWRSYSGCCRAYGTSAMMTRTCTTATATMGYGTGAY 7440
Bhutan-09027         GAGRCRAYCMWYKACAATKGGCACYWRSYSGCCRAYGTSAMMTRTCTTATATMGYGTGAY 7440
Bhutan-09030         GAGRCRAYCMWYKACAATKGGCACYWRSYSGCCRAYGTSAMMTRTCTTATATMGYGTGAY 7440
Bhutan-09005         AWKRSRTYMMACKRSMCTKSRYGTTWRCYCRYMAGTKKGWCMCGYMYTRTRTAGTRTRWC 7440
Indonesia-88035      AWKRSRTTACACGRSMCCKSRYGTTWRCYCRYCAGTKKGWCMCGYMYWRWRYASTAYRWC 7440
```

```
Indonesia-88045      AWKRSRTTACACGRSMCCKSRYGTTWRCYCRYMAGTKKGWCMCGYMYWRWRYASTRYRWC 7440
Indonesia-88065      AWKRSRTYMMACKRSMCTKSRYGTTWRCYCRYMAGTKKGWCMCGYMYTRTRTAGTRTRWC 7440
                     .  *.*:       . . *.  .  **.*.    .  ..  *           .

Bhutan-09015         KRYMMWAYGRATCMTTAYRCTCYAGYCGGTRRARRMTSYMRTKAGARGWTTKGRACKRWS 7500
Bhutan-09024         KRYMMWAYGRATCMTTAYRCTCYAGYCGGTRRARRMTSYMRTKAGARGWTTKGRACKRWS 7500
Bhutan-09027         KRYMMWAYGRATCMTTAYRCTCYAGYCGGTRRARRMTSYMRTKAGARGWTTKGRACKRWS 7500
Bhutan-09030         KRYMMWAYGRATCMTTAYRCTCYAGYCGGTRRARRMTSYMRTKAGARGWTTKGRACKRWS 7500
Bhutan-09005         TGYACATYRARCSYCCGYACWSYRRCARSYGGMRRACCYCAWTGKCGRTAATSGRYGGTC 7500
Indonesia-88035      TGYACATYRARCCYCCGYASWSYRRCARSYGGMRRACCYCATTGKCGRTAATSGRYGGTC 7500
Indonesia-88045      TGYACATYRARCCCCCGYASWSYRRCARSYGGMRRACCYCATTGKCGRTAATSGRYGGTC 7500
Indonesia-88065      TGYACATYRARCCCCCGYACWSYRRCARSYGGMRRACCYCATTGKCGRTAATSGRYGGTC 7500
                     . *   :*    .   .* . .*   . .    **  .*   .. .   ::..      .

Bhutan-09015         ARWCYRRTWWYRYRTTRYYGGKTYAYCRWWCWRYTKMARWSRGWGRRMASMCTCKGRCCR 7560
Bhutan-09024         ARWCYRRTWWYRYRTTRYYGGKTYAYCRWWCWRYTKMARWSRGWGRRMASMCTCKGRCCR 7560
Bhutan-09027         ARWCYRRTWWYRYRTTRYYGGKTYAYCRWWCWRYTKMARWSRGWGRRMASMCTCKGRCCR 7560
Bhutan-09030         ARWCYRRTWWYRYRTTRYYGGKTYAYCRWWCWRYTKMARWSRGWSRRMASMCTCKGRCCR 7560
Bhutan-09005         WGAYCGAYAATGCAYKATTKRTYCWYYGTWYWRYYGCRAACAKAGRAMWCMTYMGRGYYA 7560
Indonesia-88035      WGAYCGAYAATGCAYKATTKRTYCWYYGTWYWRYYGCRAACAKAGRAMWCMTYMGRGYYA 7560
Indonesia-88045      WGAYCGAYAATGCAYKATTKRTYCWYYGTWYWRYYGCRAACAKAGRAMWCMTYMGRGYYA 7560
Indonesia-88065      WGAYCGAYAATGCAYKATTKRTYCWYYGTWYWRYYGCRAACAKAGRAMWCMTYMGRGYYA 7560
                                    .     .   *   * ***      .   .* * .*

Bhutan-09015         RTGKRSYMYRYAMYTMSSTGYRYRKCTGYGGKAAKRMRKYRYYAYTRKTGYSRTRRKYYY 7620
Bhutan-09024         RTGKRSYMYRYAMYTMSSTGYRYRKCTGYGGKAAKRMRKYRYYAYTRKTGYSRTRRKYYY 7620
Bhutan-09027         RTGKRSYMYRYAMYTMSSTGYRYRKCTGYGGKAAKRMRKYRYYAYTRKTGYSRTRRKYYY 7620
Bhutan-09030         RTGKRSYMYRYAMYTMSSTGYRYRKCTGYGGKAAKRMRKYRYYAYTRKTGYSRTRRKYYY 7620
Bhutan-09005         AAAKRCYCTACMAYWASSWCYACATCYSYSKGMCKGMRKTGYYRTYGGARYSAWARKTTT 7620
Indonesia-88035      AAAKRCYCTACMAYWASSWCYACAKSYSCSKGMCKGMRKTGYYRTYGGARYSAWARKTTT 7620
Indonesia-88045      AAAKRCYCTACMAYWASSWCYACATCYSCSKGMCKGMRKTGYYRTYGGARYSAWARKTTT 7620
Indonesia-88065      AAAKRCYCTACMAYWASSWCYACATCYSYSKGMCKGMRKTGYYRTYGGARYSAWARKTTT 7620
                      :.**.*      *  **  *   .. . .   .* ***  **     : **   **

Bhutan-09015         ATWGSGKASRWRAYRMYRRYARCRGRTWGGAYYRKCCTRMYACGGKGTGAAYYYTSYTAS 7680
Bhutan-09024         ATWGSGKASRWRAYRMYRRYARCRGRTWGGAYYRKCCTRMYACGGKGTGAAYYYTSYTAS 7680
Bhutan-09027         ATWGSGKASRWRAYRMYRRYARCRGRTWGGAYYRKCCTRMYACGGKGTGAAYYYTSYTAS 7680
Bhutan-09030         ATWGSGKASRWRAYRMYRRYARCRGRTWGGAYYRKCCTRMYACGGKGTGAAYYCTSYTAS 7680
Bhutan-09005         GWWRGSKRSATRCYAAYAATMGYAKGYAGKGCTATMYYACTMSRRKRYSWRYYYYGCYRS 7680
Indonesia-88035      GWWRGSKRSATRCYAAYAATMGYAKGYASGACTATCYYACTMSRRKRYCWRYYYYGCTRS 7680
Indonesia-88045      GWWRGSKRSATRCYAAYAATMGYAKGYASGACTATCYYACTMSRRKRYCWRYYTYGCTRS 7680
Indonesia-88065      GWWRGSKRSATRCYAAYAATMGYAKGYAGKGCTATMYYACTMSRRKRYSWRYYYYGCYRS 7680
                     . * ..* *  *.*  *           . .   .       .  *     **  .   *

Bhutan-09015         RYKMYACYCCTYKYWGAWAMCACCACYMTYTTCCYGWCSYYKTWAACRYRCRTAKWSCTG 7740
Bhutan-09024         RYKMYACYCCTYKYWGAWAMCACCACYMTYTTCCYGWCSYYKTWAACRYRCRTAKWSCTG 7740
Bhutan-09027         RYKMYACYCCTYKYWGAWAMCACCACYMTYTTCCYGWCSYYKTWAACRYRCRTAKWSCTG 7740
Bhutan-09030         RYKMYACYCCTYKYWGAWAMCACCACYMTYTTCCYGWCSYYKTWAACRYRCRTAKWSCTG 7740
Bhutan-09005         RCGACCYTGYYYGTWTAATCYCYYRSTAWYYGTMTSTYSTTTTTGMYAYGYRYRKTGSYA 7740
Indonesia-88035      RCGACAYTSYYYKTWKAWTCCCYYRSYAWYYTTMTGTYSTTTCTGMYAYGYRYRKTGSYA 7740
Indonesia-88045      RCGACAYTSTCYKTWTAATCCCYYRSYAWYYTTMTGTYSTTTCTGMYAYGYRYRKTGSYA 7740
Indonesia-88065      RCGACCYTGYYYGTWKWATCYCYYRSTAWYYGTMTSTYSTTTTTGMYAYGYRYRKTGSYA 7740
                     *    .     *  *   :  .   .   *     .  *  .  .   *  *  * .. .
```

```
Bhutan-09015        GCRAKGTSCRSKKWWYTCRWWAACCTGRGTWRYGGYCSKYYYTRRYYKTTYYCGTSRSGR 7800
Bhutan-09024        GCRAKGTSCRSKKATTTCRWWAACCTGRGTWRYGGYCSKYYYTRRYYKTTYYCGTSRSGR 7800
Bhutan-09027        GCRAKGTSCRSKKWWYTCRWWAACCTGRGTWRYGGYCSKYYYTRRYYKTTYYCGTSRSGR 7800
Bhutan-09030        GCRAKGTSCRSKKWWYTCRWWAACCTGRGTWRYGGYCSKYYYTRRYYKTTYYCGTSRSGR 7800
Bhutan-09005        RMGRKSCGSASKGTTTASGTAWRSYKRGKYTRCGCCAGTYTCYRRYYTKWCTMRWSRCRR 7800
Indonesia-88035     RMGRKSCGSASKGTWYASGTAWRSYKRGKTTRCRCCAGKYTCTRRYYTTWCTMRWSRCRA 7800
Indonesia-88045     RMGRKSCGSASKGTTTASGTAWRSYKRGKTTRCRCCAGKYTCTRRYYTTWCTMRWSRCRA 7800
Indonesia-88065     RMGRKSCGSASKGTTTASGTAWRSYKRGKTTRCGCCAGTYTCYRRYYTKWCTMRWSRCRR 7800
                        *. .. **    :.     . .     *    ...*   ****..      **.

Bhutan-09015        YYTRAMAAGTYRAMKYGTRTGRWCAYCMRATAYYAAACGYTAARRYYTSCAYTRRCWARC 7860
Bhutan-09024        YYTRAMAAGTYRAMKYGTRTGRWCAYCMRATAYYAAACGYTAARRYYTSCAYTRRCWARC 7860
Bhutan-09027        YYTRAMAAGTYRAMKYGTRTGRWCAYCMRATAYYAAACGYTAARRYYTSCAYTRRCWARC 7860
Bhutan-09030        YYTRAMAAGTYRAMKYGTRTGRWCAYCMRATAYYAAACGYTAARRYYTSCAYTRRCWARC 7860
Bhutan-09005        CCKRRMGRSCYRGMTYACRWSRRYWCCAAWCRTCRRWTAYWWRRRCYTGCAYAAGTARAA 7860
Indonesia-88035     CCKRRMGRSCYRGMTYACRWSRRYACYAAWCRTCRRWTGYWWGRRCTYGYRYAAGTARAA 7860
Indonesia-88045     CCKRRMGRSCYRGMTYACRWSRRYACYAAWCRTCRRWTGYWWGRRCTYGYRYAAGTARAA 7860
Indonesia-88065     CCKRRMGRSCYRGMTYACRWSRRYWCCAAWCRTCRRWTAYWWRRRCYTGCAYAAGTARAA 7860
                      .* *. . **.*.*. * .*                .*   **   .  *:      .

Bhutan-09015        RYRAGSRTYATGGATTYWRGMRWRYWYYKRKRCGAAGAARRTRTTYRYYWAGCSRTCTYK 7920
Bhutan-09024        RYRAGSRTYATGGATTYWRGMRWRYWYYKRKRCGARGAARRTRTTYRYYWAGCSRTCTYK 7920
Bhutan-09027        RYRAGSRTYATGGATTYWRGMRWRYWYYKRKRCGARGAARRTRTTYRYYWAGCSRTCTYK 7920
Bhutan-09030        RYRAGSRTYATGGATTYWRGMRWRYWYYKRKRCGARGAARRTRTTYRYYWAGCSRTCTYK 7920
Bhutan-09005        ATAWRGAYCRYARWYYYAAKARAGTTTCGRGRYKCGTWWRRYGYKTAYYWAKSSRCMKCG 7920
Indonesia-88035     ATRWRGAYCRYARWYYYAAKARAGTTTCGRGRYTCATAARRYGYKTAYYWGKSSRCAGCG 7920
Indonesia-88045     ATRWRGAYCRYARWYYYAAKARAGTTTCGRGRYTCATAARRYGYKTAYYWGKSSRCAGCG 7920
Indonesia-88065     ATAWRGAYCRYARWYYYAAKARAGTTTCGRGRYKCGTWWRRYGYKTAYYWAKSSRCMKCG 7920
                         .     .    *    *       * *  .    **   .  ***. .**

Bhutan-09015        GAWYATWMYGKTTMYYRARMWRRTYCYWYCGTCYATWRMTMATGCTTMRKCWRATSCWRA 7980
Bhutan-09024        GAWYATWMYGKTTMYYRARMWRRTYCYWYCGTCYATWRMTMATGCTTMRKCWRATSCWRA 7980
Bhutan-09027        GAWYATWMYGKTTMYYRARMWRRTYCYWYCGTCYATWRMTMATGCTTMRKCWRATSCWRA 7980
Bhutan-09030        GAWYATWMYGKTTMYYRARMWRRTYCYWYCGTCYATWRMTMATGCTTMRKCWRATSCWRA 7980
Bhutan-09005        RCTYCYACTSKYYMCCAMGATAGYTSCTCYRYYTTWAACTCMYRYWYMAKSTRMKSYARR 7980
Indonesia-88035     GCTYCYACTSKYYMCCAMGATAGYTSCWCYRYYYWWAACACMYRYWYMAKSTRMKSYARR 7980
Indonesia-88045     GCTYCYACTSKYYMCCAMGATAGYTSCWCYRYYYWWAACACMYRYWYMAKSTRMKSYARR 7980
Indonesia-88065     RCTYCYACTSKYYMCCAMGATAGYTSCTCYRYYTTWAACTCMYRYWYMAKSTRMKSYARR 7980
                     . *.    .*  *           .             :       * *. * .*  *

Bhutan-09015        TTKGAYSTGTGCMRYYYRCRAKMMYRAGTAARTRWGRMCKARYAKYYWATRSAYRWGRGC 8040
Bhutan-09024        TTKGAYSTGTGCMRYYYRCRAKMMYRAGTAARTRWGRMCKARYAKYYWATRSAYRWGRGC 8040
Bhutan-09027        TTKGAYSTGTGCMRYYYRCRAKMMYRAGTAARTRWGRMCKARYAKYYWATRSAYRWGRGC 8040
Bhutan-09030        TTKGAYSTGTGCMRYYYRCRAKMMYRAGTAARTRWGRMCKARYAKYYWATRSAYRWGRGC 8040
Bhutan-09005        YTKGWCCYRYRTCATTCASRRTMATAAKKMGRYAWARMYKRACWTYYWGKGCRTRASGRY 8040
Indonesia-88035     YYGRACSYRYRCCGTCCRSRRTMATARKKMGRYAWAAMYKRACWTYYWGKGCRTAASGRY 8040
Indonesia-88045     YYGRACSYRYRCCGTCCRSRRTMATARKKMGRYAWARMYKRACWTYYWGKGCRTAASGRY 8040
Indonesia-88065     YTKGWCCYRYRTCATTCASRRTMATAAKKMGRYAWARMYKRACWTYYWGKGCRTRASGRY 8040
                          .           .* .*     . .*  *. * *    .***.. .    .

Bhutan-09015        TRTMGRGTSAKTYYAKRRYASRAGRGWKKGWCWRCYMRTRTCTCTMASYYCRKAGRTRKY 8100
Bhutan-09024        TRTMGRGTSAKTYYAKRRYASRAGRGWKKGWCWRCYMRTRTCTCTMASYYCRKAGRTRKY 8100
Bhutan-09027        TRTMGRGTSAKTYYAKRRYASRAGRGWKKGWCWRCYMRTRTCTCTMASYYCRKAGRTRKY 8100
Bhutan-09030        TRTMGRGTSAKTYYAKRRYASRAGRGWKKGWCWRCYMRTRTCTCTMASYYCRKAGRTRKY 8100
```

```
Bhutan-09005         YRYAKRACGWGWTYWKRRYWSRRRARWKKSAYTRYTMRYGYMCSWAWGCTSAKRKAYAGT 8100
Indonesia-88035      YRYAKGAYGWGWTYWKRRYWCRRAARWKKSAYTRYTMRYGYMCSWAWGCTSAKRKAYAGT 8100
Indonesia-88045      YRYAKGAYGWGWTYWKRRYWCRRAARWKKSAYTRYTMRYGYMCSWAWGCTSAKRKAYAGT 8100
Indonesia-88065      YRYAKRACGWGWTYWKRRYWSRRRARWKKSAYTRYTMRYGYMCSWAWGCTSAKRKAYAGT 8100
                      *    . .    * **** .*    ***.   *  **     .   .  . *

Bhutan-09015         CTACRAAYTGGGYYYGTRMYTRGAKYRWSWWYWAWRTTTATTGTGTRARRCGTRRRRAGR 8160
Bhutan-09024         CTACRAAYTGGGYYYGTRMYTRGAKYRWSWWYWAWRTTTATTGTGTRARRCGTRRRRAGR 8160
Bhutan-09027         CTACRAAYTGGGYYYGTRMYTRGAKYRWSWWYWAWRTTTATTGTGTRARRCGTRRRRAGR 8160
Bhutan-09030         CTACRAAYTGGGYYYGTRMYTRGAKYRWSWWYWAWRTTTATTGTGTRARRCGTRRRRAGR 8160
Bhutan-09005         SWRSRWWYYGGGYYTSYRAYYRSCTCGTCAWTWRTATYKWGWRTRTGGARMRYGGGRRKG 8160
Indonesia-88035      SWGSGWTCYKRKYYTSYRAYYGSCTCGTCAWTWRTAKYKWGARYGAGGARMRCGGGGRKG 8160
Indonesia-88045      SWGSGWTCYKRKYYTSYRAYYGSCTCGTCAWTWRTAKYKWGARYGAGGARMRCGGGGRKG 8160
Indonesia-88065      SWRSRWWYYGGGYYTSYRAYYRSCTCGTCAWTWRTATYKWGWRTRTGGARMRYGGGRRKG 8160
                     .  .        ** . * *  ...   . * *   . .      : . *

Bhutan-09015         AYGCTMRRCYKAKARSSSKRRAKSGTKYGYRCTAARGATAGRTYKRRCRTCRYGAATYAW 8220
Bhutan-09024         AYGCTMRRCYKAKARSSSKRRAKSGTKYGYRCTAARGATAGRTYKRRCRTCRYGAATYAW 8220
Bhutan-09027         AYGCTMRRCYKAKARSSSKRRAKSGTKYGYRCTAARGATAGRTYKRRCRTCRYGAATYAW 8220
Bhutan-09030         AYGCTMRRCYKAKARSSSKRRAKSGTKYGYRCTAARGATAGRTYKRRCRTCRYGAATYAW 8220
Bhutan-09005         CTGTKMARCYGRTRGCGGGRGMTGRYTTRYRYYWWGAWYWKRWCGGACACYGTRRRYCMW 8220
Indonesia-88035      CTRTKMARTYGRTRGCGGGRGMTGRYTTRYRYYWWGAWYWKRWCGGACACCGTRRRYCMW 8220
Indonesia-88045      CTRTKMARTYGRTRGCGGGRGMTGRYTTRYRYYWWGAWYWKRTCGGAYACCGTRRRYCMW 8220
Indonesia-88065      CTGTKMARCYGRTRGCGGGRGMTGRYTTRYRYYWWGAWYWKRWCGGAYACCGTRRRYCMW 8220
                     .   .* * *  .  ... *  ..  .  **     .    *                 *

Bhutan-09015         YYYATRSMWAMWMSAASKWAYRYTTTCGCYATRRRAACRCGTAAMWKRCYGYGAWYYWTY 8280
Bhutan-09024         YYYATRSCAAMWMSAASKWAYRYTTTCKCYATAAAAACRCGTAAMWKRCYGYGATYYWTY 8280
Bhutan-09027         YYYATRSMWAMWMSAASKWAYRYTTTCKCYATAAAAACRCGTARMWKRCYGYGAWYYWTY 8280
Bhutan-09030         YYYATRSMWAMWMSAASKWAYRYTTTCKCYATAAAAACRCGTARMWKRCYGYGATYYWTY 8280
Bhutan-09005         CCTTCGCCAWAACSMMGTTRYGCYWWSTYCWYAAAWRYRYRTARCATAYTGTATTCCTYT 8280
Indonesia-88035      CCTWCGCCAWAACSMMGTTRYGCYWWSTYCATAAAWRYRYRTTACATAYTRTATTCCTYT 8280
Indonesia-88045      CCTWCGCCAWAACSMMGTTRYGCYWWSTYCWYAAAWRYRYRTTACATAYTRTATTCCTYT 8280
Indonesia-88065      CCTTCGCCATAACSMMGTTRYGCYWWSTYCWYAAAWRYRYRWWACATAYTRTATTCCTYT 8280
                           .      *  ..  *     .           *       .     .:

Bhutan-09015         GACCCAAACTTGAAKYGACRTTMYSKYAYYWYKCGGTCAGKWGGGRGTMTTKCSKASGYR 8340
Bhutan-09024         GACCCAAACTTGAAKYGACRTTMYSKYAYYWYKCGGTCAGKWGGGRGTMTTKCSKASGYR 8340
Bhutan-09027         GACCCAAACTTGAAKYGACRTTMYSKYAYYWYKCGGTCAGKWGGGRGTMTTKCSKASGYR 8340
Bhutan-09030         GACCCAAACTTGAAKYGACRTTMYSKYAYYWYKCGGTCAGKWGGGRGTMTTKCSKASGYR 8340
Bhutan-09005         AWYYYMRRYWYRRWTMTGYRYACYCTTTTCTYKMRTYSMRKWGKRARYAYWGYGTRGRTA 8340
Indonesia-88035      AWYYYMRRYWYRRWTMTGYRYACYCTTTTCTYKMRTYSMRKWGKRARYAYWGYGTRGRTA 8340
Indonesia-88045      AWYYYMRRYWYRRWTMTGYRYACYCTTTTCTYKMRTYSMRKWKKRARYAYWGYGTRGRTA 8340
Indonesia-88065      AWYYYMRRYWYRRWTMTGYRYACYCTTTTCTYKMRTYSMRKWGKRARYAYWGYGTRGRTA 8340
                     .             .  . * : *.. :   **    .  **           .. .

Bhutan-09015         CYRWKMRCTGCWYCCRYAMYYCCWYTTACTSTCTKWGTTYWCAARTACAATYRKAGRYYW 8400
Bhutan-09024         CYRWKMRCTGCWYCCRYAMYYCCWYTTACTSTCTKWGTTYWCAARTACAATYRKAGRYYW 8400
Bhutan-09027         CYRWKMRCTGCWYCCRYAMYYCCWYTTACTSTCTKWGTTYWCAARTACAATYRKAGRYYW 8400
Bhutan-09030         CYRWKMRCTGCWYCCRYAMYYCCWYTTACTSTCTKWGTTYWCAARTACAATYRKAGRYYW 8400
Bhutan-09005         YTGAGMGMWRYAYTTRYWMCYYYATKARYCSYYKKTTGCYAYTMGKMYRRYTATTRAYCA 8400
Indonesia-88035      YTGAGMGMWRYAYTTRYWMCYYYATKARYCSYYKKTTGCYAYTMGKMYRRYTATTRAYCA 8400
Indonesia-88045      YTGAGMGMWRYAYTTRYWMCYYYATKARYCSYYKKTTGCYAYTMGKMYRRYTATTRAYCA 8400
Indonesia-88065      YTGAGMGMWRYAYTTRYWMCYYYATKARYCSYYKKTTGCYAYTMGKMYRRYTATTRAYCA 8400
```

```
                                   *      *  ** * *    .:   *  .*    *  :  .       .:  *

Bhutan-09015         WMRYRYWYYCGKCAWRYTWRWCGYTCYYRYYAYKWWWYGSSYRYAYCCCRGKRRRTRCCG 8460
Bhutan-09024         WMRYRYWYYCGKCAWRYTWRWCGYTCYYRYYAYKWWWYGSSYRYAYCCCRGKRRRTRCCG 8460
Bhutan-09027         WMRYRYWYYCGKCAWRYTWRWCGYTCYYRYYAYKWWWYGSSYRYAYCCCRGKRRRTRCCG 8460
Bhutan-09030         WMRYRYWYYCGKCAWRYTWRWCGYTCYYRYYAYKWWWYGSSYRYAYCCCRGKRRRTRCCG 8460
Bhutan-09005         TMGCGTWTTYRTTAARCKTAWYRCTSCCRCYWYGWWWYRSGYRYWYKSYASKRGAYGTGK 8460
Indonesia-88035      TMGCGTWTTYRTTRWRCKTAWYRCYSCCRCCWYGWWWYRSGYRYWYKSYASKRGAYGTGK 8460
Indonesia-88045      TMGCGTWTTYRTTRWRCKTAWYRCYSCCRCYWYGWWWYRSGYRYWYKSYASKRGAYGTGK 8460
Indonesia-88065      TMGCGTWTTYRTTAARCKTAWYRCTSCCRCCWYGWWWYRSGYRYWYKSYASKRGAYGTGK 8460
                      *    *    .   * .  *    .  *   * **** *.*** * .  .**

Bhutan-09015         YRAMRTTYMGRTTYRRTSWTCCRYRRRRGMTRWGRYSYATMCRYWMAAAGAGYKYKWSRA 8520
Bhutan-09024         YRAMRTTYMGRTTYRRTSWTCCRYRRRRGMTRWGRYSYATMCRYWMAAAGAGYKYKWSRA 8520
Bhutan-09027         YRAMRTTYMGRTTYRRTSWTCCRYRRRRGMTRWGRYSYATMCRYWMAAAGAGYKYKWSRA 8520
Bhutan-09030         YRAMRTTYMGRTTYRRTSWTCCRYRRRRGMTRWGRYSYATMCRYWMAAAGAGYKYKWSRA 8520
Bhutan-09005         CRGCACKYMAAKYYRATCTWMMACAAAGSCCGARGYGTWYAYGYWMTRWRACTTCKWSRM 8520
Indonesia-88035      CRGCACKYMAAKYYRAYCTWMMACAAAGSCCGARGYGTWTAYGYWMTRWRGCYTCKWSRM 8520
Indonesia-88045      CRGCACKYMAAKYYRAYCTWMMACAAAGSCCGARGYGTWTAYGYWMTRWRGCYTCKWSRM 8520
Indonesia-88065      CRGCACKYMAAKYYRATCTWMMACAAAGSCCGARGYGTWYAYGYWMTRWRACTTCKWSRM 8520
                      *.   .**. . **  .          .      *.      ***:   .  . ****

Bhutan-09015         RAGTAKCAGTAYYACCYYWACYYWAWACRMCRYCMWRRCYSRWRMGRTSRCCTYMRTCYA 8580
Bhutan-09024         RAGTAKCAGTAYYACCYYWACYYWAWACRMCRYCMWRRCYSRWRMGRTSRCCTYMRTCYA 8580
Bhutan-09027         RAGTAKCAGTAYYACCYYWACYYWAWACRMCRYCMWRRCYSRWRMGRTSRCCTYMRTCYA 8580
Bhutan-09030         RAGTAKCAGTAYYACCYYWACYYWAWACRMCRYCMWRRCYSRWRMGRTSRCCTYMRTCYA 8580
Bhutan-09005         RMKYMKYWKKRTTRYYTCATYTCWATRTACSGCCCAAGYYCRAAAKAGGRYTKYMRCYCR 8580
Indonesia-88035      RCKYCTYWKKRTTRYYTCATYTCWRTRTACSGCSCAAGYYCRAAAGAKGRYYKYMGCYCR 8580
Indonesia-88045      RMKYMKYWKKRTTRYYTCATYTYWATRTACSGCSCAAGYYCRAAAGAKGRYYKYMGCYCR 8580
Indonesia-88065      RMKYMKYWKKRTTRYYTCATYTCWATRTACSGCCCAAGYYCRAAAKAGGRYTKYMRCYCR 8580
                     *    .   .         :    *      .   .     *.*      .*   .**

Bhutan-09015         CYTSGTWWMGYSRCTMCKRCWYTWYYYAWTRCGWRGYCAAMAARRTYRWYGAGYTGTART 8640
Bhutan-09024         CYTSGTWWMGYSRCTMCKRCWYTWYYYAWTRCGWRGYCAAMAARRTYRWYGAGYTGTART 8640
Bhutan-09027         CYTSGTWWMGYSRCTMCKRCWYTWYYYAWTRCGWRGYCAAMAARRTYRWYGAGYTGTART 8640
Bhutan-09030         CYTSGTWWMGYSRCTMCKRCWYTWYYYAWTRCGWRGYCAAMAARRTYRWYGAGYTGTART 8640
Bhutan-09005         TTYCRYATCRCCAYKMYTGTWTAAYTCRAGAMTTAKYYRWMRAAGYCAATAGRTYKWGGK 8640
Indonesia-88035      TTYCRYATCACCAYKMYTGCWTAAYTCRAGAMTTAKYYRWMRTAGYCAATRGRTYKWGGK 8640
Indonesia-88045      TTYCRYATCRCCAYKMYTGCWTAAYTCRAGAMTTAKYYRWMRTAGYCAATRGRTYKWGGK 8640
Indonesia-88065      TTYCRYATCRCCAYKMYTGTWTAAYTCRAGAMTTAKYYRWMRAAGYCAATAGRTYKWGGK 8640
                        .       .  .* .  * : *           *   * :         .      . .

Bhutan-09015         YWYACARRRWCSGMTARGRWCRWRWAMRTTYCSACRTRYRTMGWGRCKKTTRCCAGMRTM 8700
Bhutan-09024         YWYACARRRWCSGMTARGRWCRWRWAMRTTYCSACRTRYRTMGWGRCKKTTRCCAGMRTM 8700
Bhutan-09027         YWYACARRRWCSGMTARGRWCRWRWAMRTTYCSACRTRYRTMGWGRCKKTTRCCAGMRTM 8700
Bhutan-09030         YWYACARRRWCSGMTARGRWCRWRWAMRTTYCSACRTRYRTMGWGRCKKTTRCCAGMRTM 8700
Bhutan-09005         CAYRMWGRRTYCRCYWRGGWCRWRWWMAYYTYCMYRKRTRKCRWGRTKGAYAYYRAMAKA 8700
Indonesia-88035      CACRMWGRRTYCRCCWRAGAMRWRWWMAYYTYCMYRKRTRKCRWSRTKGTYGCCAAMAKA 8700
Indonesia-88045      CACRMWGRRTYCRCCWRAGAMRWRWWMAYYTYCMYRKRTRKCRWSRTKGTYGCCAAMAKA 8700
Indonesia-88065      CAYRMWGGGTYCRCYWRGGACRWRWWMAYYTYCMYRKRTRKCRWGRTKGAYAYYRAMAKA 8700
                                .    *.   **** *      .  *.* *.   *.* * :      .* .

Bhutan-09015         MAYMRWKGRYCYRCATGRGGAGTSTYYTCRYATMGAWGYAYYYSYGRYWGRYYRRYGCYK 8760
Bhutan-09024         MAYMRWKGRYCYRCATGRGGAGTSTYYTCRYATMGAWGYAYYYSYGRYWGRYYRRYGCYK 8760
```

```
Bhutan-09027          MAYMRWKGRYCYRCATGRGGAGTSTYYTCRYATMGAWGYAYYYSYGRYWGRYYRRYGCYK 8760
Bhutan-09030          MAYMRWKGRYCYRCATGRGGAGTSTYYTCRYATMGAWGYAYYYSYGRYWGRYYRRYGCYK 8760
Bhutan-09005          AWTAGWKKRYMTRTGCARRKWSYSWYYGCRTWWMSCWRTWTTYCYAACWKRTCAATCSYK 8760
Indonesia-88035       AWTAGWKKRYMTRTGCARRKWSYSWYYGYRTWWMSCWRTWTTYCYAACWGRCCAGTGSYK 8760
Indonesia-88045       AWTAGWKKRYMTRTGCARRKWSYSWYYGYRTWWMSCWRTWTTYCYAACWGRCCAGTGSYK 8760
Indonesia-88065       AWTAGWKKRYMTRTGCARRKWSYSWYYGCRTWWMSCWRTWTTYCYAACWKRTCAATCSYK 8760
                           ** **  * . .*   . * **  *   *..*     *.*.  * *      .**

Bhutan-09015          MKTTYTMCWGCCYTYCCACCTSCAGTMYYGMARYYRRGWSWRRYGYKWAAGYCATGASCT 8820
Bhutan-09024          MKTTYTMCWGCCYTYCCACCTSCAGTMYYGMARYYRRGWSWRRYGYKWAAGYCATGASCT 8820
Bhutan-09027          MKTTYTMCWGCCYTYCCACCTSCAGTMYYGMARYYRRGWSWRRYGYKWAAGYCATGASCT 8820
Bhutan-09030          MKTTYTMCWGCCYTYCCACCTSCAGTMYYGMARYYRRGWSWRRYGYKWAAGYCATGASCT 8820
Bhutan-09005          MTCCTWAYTRTSYYTYYATCWGMMCKMYCKARGTTGAKWGWARCSYKWRWATSRYRWSYY 8820
Indonesia-88035       MTCCYWAYTRTSYYTYYWTSWGMMCKMYCKARGTTGAKWGWARCSYKWRWATSRYRWSYY 8820
Indonesia-88045       MTCCYWAYTRTSYYTYYWTSWGMMCKMYCKARGTTGAKWGWARCSYKWRWATSRYRWSYY 8820
Indonesia-88065       MTCCTWAYTRTSYYTYYWTSWGCACKMYCKARGTTGAKWGWARCSYKWRWATSRYRWSYY 8820
                      *.         .*      . .   .**          *.* * .***  . .    *

Bhutan-09015          CRYRYRMRKAMYTGCYASAYRAAGGWRTCAYMAWKGKAGGTYACGTAARKGARSTSGYCY 8880
Bhutan-09024          CRYRYRMRKAMYTGCYASAYRAAGGWRTCAYMAWKGKAGGTYACGTAARKGARSTSGYCY 8880
Bhutan-09027          CRYRYRMRKAMYTGCYASAYRAAGGWRTCAYMAWKGKAGGTYACGTAARKGARSTSGYCY 8880
Bhutan-09030          CRYRYRMRKAMYTGCYASAYRAAGGWRTCAYMAWKGKAGGTYACGTAARKGARSTSGYCY 8880
Bhutan-09005          SAYGYGCGTWMTYRTTRCCTRGWSKWGYSRYAMWGKTMARYYTYRYRWGKCRRGYCRYST 8880
Indonesia-88035       SAYGYGCGTWMTYRTTRCATRGWSKWGYSRYAMWGKTMARYYTYRYRWGKCRRGYCRYST 8880
Indonesia-88045       SAYGYGCGTWMTYRTTRCATRGASKWGYSRYAMWGKTMAACYTYRYRWGKCRRGYCRYST 8880
Indonesia-88065       SAYGYGCGTWMTYRTTRCCTRGWSKWGYSRYAMWGKTMARYYTYRYRWGKCRRGYCRYST 8880
                      . * *   . *      .. *. . *  . *  * . .  *:      *  *. . *.

Bhutan-09015          RRRGYATYAKRKRYWKRRRGYACRMARGWCACGTGGYTCMCWRTYRCRYTYRMAYCRAAC 8940
Bhutan-09024          RRRGYATYAKRKRYWKRRRGYACRMARGWCACGTGGYTCMCWRTYRCRYTYRMAYCRAAC 8940
Bhutan-09027          RRRGYATYAKRKRYWKRRRGYACRMARGWCACGTGGYTCMCWRTYRCRYTYRMAYCRAAC 8940
Bhutan-09030          RRRGYATYAKRKRYWKRRRGYACRMARGWCACGTGGYTCMCWRTYRCRYTYRMAYCRAAC 8940
Bhutan-09005          RGGKTWYCRGRGACTKAAARYMSGCGGRAYRSSYRRCYYCMTAGTAMGTCYAMMTYAMGT 8940
Indonesia-88035       RGGKTWYCAGRGACTKAAARYMSGCGGRAYRSSYRRCYYCMTAGTAMGTCYAMMTYAMGT 8940
Indonesia-88045       RGGKTWYCAGRGACTKAAARYMSGCGGRAYRSSYRRCYYCMTAGTAMGTCYAMMTYAMGT 8940
Indonesia-88065       RGGKTWYCRGRGACTKAAARYMSGCGGRAYRSSYRRCYYCMTAGTAMGTCYAMMTYAMGT 8940
                      *         *    *    * .  .     ..                 * *     .

Bhutan-09015          AAGARYCCTAAYKGACTRAWKTCCTRRGYGMYGRGAKRWRWWAGGGSCATYRCRRYRGYS 9000
Bhutan-09024          AAGARYCCTAAYKGACTRAWKTCCTRRGYGMYGRGAKRWRWWAGRGSCATYRCRRYRGYS 9000
Bhutan-09027          AAGARYCCTAAYKGACTRAWKTCCTRRGYGMYGRGAKRWRWWAGRGSCATYRCRRYRGYS 9000
Bhutan-09030          AAGARYCCTAAYKGACTRAWKTCCTRRGYGMYGRGAKRWRWWAGRGSCATYRCRRYRGYS 9000
Bhutan-09005          GCRRACMSCRWYTRTYGGRATCTAGAKKYSCCRAKWTAAGAWRSAKSGWKCAGRRCARTS 9000
Indonesia-88035       GCRRACMSCRWYTRTYGGRATCTAGAKKYSCCRATTTAAGAWGSATSGWKCAGRRCARTS 9000
Indonesia-88045       GCRRACMSCRWYTRTYGGRATCTAGAKKYSCCRATTTAAGAWGSATSGWKCAGRRCARTS 9000
Indonesia-88065       GCRRACMSCRWYTRTYGGRATCTAGAKKYSCCRAKWTAAGAWRSAKSGWKCAGRRCARTS 9000
                      ..     .   *. :     .  .  : *.      .    * .  *  .   **    *

Bhutan-09015          CRRAYRYTASTRSKYARRMRTRATRRRCTAGMKYMTKRWRTYACRCSGYRTTGTYYMRRC 9060
Bhutan-09024          CRRAYRYTASTRSKYARRMRTRATRRRCTAGMKYMTKRWRTYACRCSGYRTTGTYYMRRC 9060
Bhutan-09027          CRRAYRYTASTRSKYARRMRTRATRRRCTAGMKYMTKRWRTYACRCSGYRTTGTYYMRRC 9060
Bhutan-09030          CRRAYRYTASTRSKYARRMRTRATRRRCTAGMKYMTKRWRTYACRCSGYRTTGTYYMRRC 9060
Bhutan-09005          SRARYATKWSKGGTCRRGCAYARCRRRYYRKMKYCWTAWGWYRYAMSAYRKYSYYCCRAM 9060
Indonesia-88035       SRARTATKWSKGGTCRRGCAYARCRRRYYRKMKYCWTAWGWYRYAMSAYGKYSYYCCRAM 9060
```

```
Indonesia-88045        SRAAYATKWSKGGTCRRGCAYARCRRRYYRKMKYCWTAWGWYRYAMSAYGKYSYYCCRAM 9060
Indonesia-88065        SRARYATKWSKGGTCRRGCAYARCRRRYYRKMKYCWTAWGWYRYAMSAYGKYSYYCCRAM 9060
                       .*     . *. ..  *       ***    ***  . *  *    *.* . . *  *

Bhutan-09015           GGTWYWKWTGGCSWWRRMTTATTTGGMKTATCAAAKYRAAWACTCWTRYKSRMYAYYGRY 9120
Bhutan-09024           GGTWYWKWTGGCSWWRRMTTATTTGGMKTATCAAAKYRAAWACTCWTRYKSRMYAYYGRY 9120
Bhutan-09027           GGTWYWKWTGGCSWWRRMTTATTTGGMKTATCAAAKYRAAWACTCWTRYKSRMYAYYGRY 9120
Bhutan-09030           GGTWYWKWTGGCSWWRRMTTATTTGGMKTATCAAAKYRAAWACTCWTRYKSRMYAYYGRY 9120
Bhutan-09005           RSYWCWGAYRRTCAWARMWWMWWKRRMTGRYYWGRTTAMTTRYYYAYGYKGGMCTYTRGT 9120
Indonesia-88035        RSYWCWGAYRRTCAAARMWWMWWKRRMTGRYYWGRTTAMTTRYYYAYGYKCGMCTYTRGT 9120
Indonesia-88045        RSYWCWGAYRRTCAAARMWWMWWKRRMTGRYYWGRTTAMTTRYYYAYGYKCGMCTYTRGT 9120
Indonesia-88065        RSYWCWGAYRRTCAWARMWWMWWKRRMTGRYYWGRTTAMTTRYYYAYGYKGGMCTYTRGT 9120
                        . * *      .   **     .  *.     . .   :        **  * :*

Bhutan-09015           AYTAGKAAAWYGYTTMSCYTGYWTCRTRGWTSYTRGMCMAWYYWYKWWAAKRAATKAAYC 9180
Bhutan-09024           AYTAGKAAAWYGYTTMSCYTGYWTCRKRGWTSYTRGMCMAWYYWYKWWAAKRAATKAAYC 9180
Bhutan-09027           AYTAGKAAAWYGYTTMSCYTGYWTCRKRGWTSYTRGMCMAWYYWYKWWAAKRAATKAAYC 9180
Bhutan-09030           AYTAGKAAAWYGYTTMSCYTGYWTCRKRGWTSYTRGMCMAWYYWYKWWAAKRAATKAAYC 9180
Bhutan-09005           RTWMRGRGRWCACAGMCACKRCAKTGKRCWWSYYRKCTCRWYTATKWTGGTAMRYGRMYG 9180
Indonesia-88035        RTWMRGRGRWCACAGMCACKRCAKTGTRCWWGYYRKCTCRWYTATKWTGGTAMRYGRMYG 9180
Indonesia-88045        RTWMRGRGRWCACAGMCACKRCAKTGKRCWWGYYRKCTCRWYTATKWTGGTAMRYGRMYG 9180
Indonesia-88065        RTWMRGRGRWCACAGMCACKRCAKTGKRCWWSYYRKCTCRWYTATKWTGGTAMRYGRMYG 9180
                              . * . : *.. .   .  .* * .* *     **   ** ...       *

Bhutan-09015           YWKYAWRKKTKTTYAYTWGMRKMKYKACMGGAYGYACGYAYAGMYRGRRRAAWKMGRACW 9240
Bhutan-09024           YWKYAWRKKTKTTYAYTWGMRKMKYKACMGGAYGYACGYAYAGMYRGRRRAAWKMGRACW 9240
Bhutan-09027           YWKYAWRKKTKTTYAYTWGMRKMKYKACMGGAYGYACGYAYAGMYRGRRRAAWKMGRACW 9240
Bhutan-09030           YWKYAWRKKTKTTYAYTWGMRKMKYKACMGGAYGYACGYAYAGMYRGRRRAAWKMGRACW 9240
Bhutan-09005           YWTYMTATTKKYYCRCYAKMGGCGTGWCASRMYRYMYRYWYRRATGRRRRGRWTCTGRMW 9240
Indonesia-88035        YATYMTATTKKYYCRCYAKMGGCGTGWCASRMYRYMYRYWYRRATGRRAGGRWTCTGRMW 9240
Indonesia-88045        YWTYMTATTKKYYCRCYAKCGGCGTGWMASRMYRYMYRYWYRRATGRRRRGRWTCTGRMW 9240
Indonesia-88065        YWTYMTATTKKYYCRCYAKMGGCGTGWCASRMYRYMYRYWYRRATGRRRRGRWTCTGRMW 9240
                       * .*   ...*                  .  * *   * *      *  . *.     *

Bhutan-09015           YGKGGTKSCSCTYGGCYGTAAGYSAYCGKTRGRRWRYTWTCATSCRTRTYRYRYTYAYWK 9300
Bhutan-09024           YGKGGTKSCSCTYGGCYGTAAGYSAYCGKTRGRRWRYTWTCATSCRTRTYRYRYTYAYWK 9300
Bhutan-09027           YGKGGTKSCSCTYGGCYGTAAGYSAYCGKTRGRRWRYTWTCATSCRTRTYRYRYTYAYWK 9300
Bhutan-09030           YGKGGTKSCSCTYGGCYGTAAGYSAYCGKTRGRRWRYTWTCATSCRTRTYRYRYTYAYWK 9300
Bhutan-09005           TKGGRCKCMCYYYRRYCRYMRRYSTYMGGYAKRRTATGTYMRYSYGYRYTRYRTYTRTAT 9300
Indonesia-88035        TKGRGYKCMCYYYRRCCRTARRYSTYMRGYAGGRTATGTYMRYSYGYRYTRYRTYTRTAT 9300
Indonesia-88045        TKGRGYKCMCYYYRRCCRTARRYSTYMRGYAGGRTATGTYMRYSYGYRYTRYGTYTRTAT 9300
Indonesia-88065        TKGGRCKCMCYYYRRYCRTARRYSTYMGGYAKRRTATGTYMRYSYGYRYTRYRTYTRTAT 9300
                             *. .  *         **:*       *         *   *  **       .

Bhutan-09015           TTRMTTRMYKTATTYGWYKARYGACYYWTYMAYYAKAGYRAMYRRWRTKRCMYCYWGRSY 9360
Bhutan-09024           TTRMTTRMYKTATTYGWYKARYGACYYWTYMAYYAKAGYRAMYRRWRTKRCMYCYWGRSY 9360
Bhutan-09027           TTRMTTRMYKTATTYGWYKARYGACYYWTYMAYYAKAGYRAMYRRWRTKRCMYCYWGRSY 9360
Bhutan-09030           TTRMTTRMYKTATTYGWYKARYGACYYWTYMAYYAKARYRAMYRRWRTKRCMYCYWGRSY 9360
Bhutan-09005           CGGAAGRYCTARTKCKTCKGRTRWTCCWCTAMYYGGMACAWCYGGTATKGYATYYWRRCT 9360
Indonesia-88035        CGGAAGRYCTARWKCKTCKGRTATTCYWCTAMYYGGMACAWCYAGTAAKGYATYYWRRCT 9360
Indonesia-88045        CGGAAGRYCTARWKCKTCKGRTATTCYWCTAMYYGGMACAWCYAGTAAKGYATCYWRRCT 9360
Indonesia-88065        CGGAAGRYCTARTKCKTCKGRTRWTCCWCTAMYYGGMACAWCYGGTATKGYATYYWRRCT 9360
                           : *  .:  .    *.*      *    **.       *    :*     ** *.
```

```
Bhutan-09015         AARATYRRGCCCATCTSGTRAAYRRRRCGRRKCSRWASTYKKAGRRCTGGRTMGTTMYSR 9420
Bhutan-09024         AARATYRRGCCCATCTSGTRAAYRRRRCGRRKCSRWASTYKKAGRRCTGGRTMGTTMYSR 9420
Bhutan-09027         AARATYRRGCCCATCTSGTRAAYRRRRCGRRKCSRWASTYKKAGRRCTGGRTMGTTMYSR 9420
Bhutan-09030         AARATYRRGCCCATCTSGTRAAYRRRRCGRRKCSRWASTYKKAGRRCTGGRTMGTTMYSR 9420
Bhutan-09005         WRAMTCRARATTTCTASACRRCCGRRGMRRATGGGWRSYYKKMRAGSYKKAGCKCYMCGG 9420
Indonesia-88035      WRAACCRARCTTTCTASACRRCCGRRGMRRATGGGWRSYYKKMRAGSTKKAGCGYYMCGG 9420
Indonesia-88045      WRAACCRARCTTTCTACACRRCCGRRGMRRATGGGWRSYYKKMRAGSTGKAGCGYYMCGG 9420
Indonesia-88065      WRAMTCRARATTTCTASACRRCCGRRGMAGATGGGWRSYYKKMRAGSYKKAGCKCYMCGG 9420
                           *  .  :  :.. * .  **     . . * * ***    .         * .

Bhutan-09015         CYGTMRAKWYYSRGYYCTRGCCTTTATTTAYARCCCCGRYTTSYTSRRTYTMAYKCRCYS 9480
Bhutan-09024         CYGTMRAKWYYSRGYYCTRGCCTTTATTTAYARCCCCGRYTTSYTSRRTYTMAYKCRCYS 9480
Bhutan-09027         CYGTMRAKWYYSRGYYCTRGCCTTTATTTAYARCCCCGRYTTSYTSRRTYTMAYKCRCYS 9480
Bhutan-09030         CYGTMRAKWYYSRGYYCTRGCCTTTATTTAYARCCCCGRYTTSYTSRRTYTMAYKCRCYS 9480
Bhutan-09005         YSAAMARGWTTGGRCCYWAAGTCWWGWWCRYWRSTYYRRCCYGCYGRRYYYARYGAAYCM 9480
Indonesia-88035      YSAAMARGWTTGGGCCYWARSTYWWGTWYAYARSYCTRRCTYGCTGRRYYYARYGCAYCM 9480
Indonesia-88045      YSAAMARGWTTGGGCCYWARSTYWWGTWYAYAASYCTRRCTYGCTGRRYYYARYGCAYCM 9480
Indonesia-88065      YSAAMARGWTTGGRCCYWAAGTCWWGWWCRYWRSTYYRRCCYGCYGRRYYYAGYGAAYCM 9480
                       .:*   *  .             .    *  .    *   .  .** *   * .

Bhutan-09015         TSMYGGGSWRCGWTAGYKAYMGGTMYRKYCSMYSTRTACYACAWAGRWAAWTTYSWCSGT 9540
Bhutan-09024         TSMYGGGSWRCGWTAGYKAYMGGTMYRKYCSMYSTRTACYACAWAGRWAAWTTYSWCSGT 9540
Bhutan-09027         TSMYGGGSWRCGWTAGYKAYMGGTMYRKYCSMYSTRTACYACAWAGRWAAWTTYSWCSGT 9540
Bhutan-09030         TSMYGGGSWRCGWTAGYKAYMGGTMYRKYCSMYSTRTACYACAWAGRWAAWTTYSWCSGT 9540
Bhutan-09005         ACCYRKSGWRYAAKRRCTGYMGKYMCRGCYSCCCYGKMTYWMRWMRRRRRAKCCSTYSAW 9540
Indonesia-88035      ACCYRKSGWRYRAKRRCKAYARKYMCRGCYSCCCYGKMTYWMRWMRRRRRAKCCSTYSAW 9540
Indonesia-88045      ACCYRKGGWRYRAKRRCGAYARKYMCRGCYSCCCYGKMTYWMRWARRRRRAKCCSTYSAT 9540
Indonesia-88065      ACCYRKSGWRYRAKRRCTGYARKYMCRGCYSCCCYGKMTYWMRWMRRRRRAKCCSTYSAT 9540
                     :. *  ..**   .    .*    * *   *  .  .  *   *  *    .  *  *.

Bhutan-09015         TAAYGTTAARAMTYYWSYYRAYRWYATAWSWRCAWWYAACAYGGRCGGCYWYGCYRRYWW 9600
Bhutan-09024         TAAYGTTAARAMTYYWSYYRAYRWYATAWSWRCAWWYAACAYGGRCGGCYWYGCYRRYWW 9600
Bhutan-09027         TAAYGTTAARAMTYYWSYYRAYRWYATAWSWRCAWWYAACAYGGRCGGCYWYGCYRRYWW 9600
Bhutan-09030         TAAYGTTAARAMYYYWSYYRAYRWYATAWSWRCAMWCAACAYGGRCGGCYWYGCYRRYWW 9600
Bhutan-09005         YRMMAKWWRRWYTYTTCTYGRCGAYGAWTCAGAGYTCCGAMCRARYKRYTWYRYYRACAT 9600
Indonesia-88035      YRMMAGATRRWYTYTTSYYRRCGAYGAWTCAGAGYTCCGAMCRARCKRYTWCRYYGACAT 9600
Indonesia-88045      TRMMAKWWRRWYTYTTSYYRRCGAYGAWTCAGAGYTCCGAMCRARCKRYTWCRYYGACAT 9600
Indonesia-88065      TRMMAKWWRRWYTYTTCTYGRCGAYGAWTCAGAGYTCCGAMCRARYKRYTWYRYYRACAT 9600
                         .    *   *  . *     *.:  .  ..   ...   .*     *   *

Bhutan-09015         WWKTKWWRYKAGCCYRARCTTRRKRKKYGYGSRYRKTYRYYAWYGYWMGWCTTATTKTMM 9660
Bhutan-09024         WWKTKWWRYKAGCCYRARCTTRRKRKKYGYGSRYRKTYRYYAWYGYWMGWCTTATTKTMM 9660
Bhutan-09027         WWKTKWWRYKAGCCYRAGCTTRRKRKKYGYGSRYRKTYRYYAWYGYWMGWCTTATTKTMM 9660
Bhutan-09030         WWKTKWWRYKAGCCYRAGCTTRRKRKKYGYGSRYRKTYRYYAWYGYWMGWCTTATTKTMM 9660
Bhutan-09005         ATSWKWAACTRRYSTACGYYAGGTRGTCGCRCRTGTYCMTCRWCGTWMRTTAYRYKSYAA 9660
Indonesia-88035      ATYWKWAACTRRTGTACGYYAGGTRGTCRCGCRTGTYCMTCRWCRYWMRTTAYRYKSYAA 9660
Indonesia-88045      ATYWKWAACTRRYSTACGYYAGGTRGTCRCGCRTGTYCMTCRWCRYWMRTTAYRYKSYAA 9660
Indonesia-88065      ATSWKWAACTRRYSTACGYYAGGTRGTCGCRCRTGTYCMTCRWCGTWMRTTAYRYKSYAA 9660
                         **   .      .   :  .* .    .*  .      *  **   :   ..

Bhutan-09015         YYGYMMRTAWAYGGGRKKCAYRTYWKYTWCTKATATYMRYTATYRAWACGCCARTTCWCM 9720
Bhutan-09024         YYGYMMRTAWAYGGGRKKCAYRTYWKYTWCTKATATYMRYTATYRAWACGCCARTTCWCM 9720
Bhutan-09027         YYGYMMRTAWAYGGGAGKCAYRTYWKYTWCTKATATYMRYTATYRAWACGCCARTTCWCM 9720
Bhutan-09030         YYGYMMRTAWATGGGAGKCAYRTYWKYTWCTKATATYMRYTATYRAWACGCCARTTCWCM 9720
```

```
Bhutan-09005         YCGCCCGYRTWYGRGAGKCATGKTTTMYWMCTCYGYTAATWGATGRAAYRYYGAWCTTMM 9720
Indonesia-88035      YCGCCCGYRTWCRRGRKGYRTGKTTTMYAACTCYGYTAATWGATGAAGYRYYGAWCTTMM 9720
Indonesia-88045      YCGCCCGYRTWCRRARKGYRTGKTTTMYAACTCYGYTAATWGATGRAGYRTTGAWCTTMM 9720
Indonesia-88065      YCRCCCGYRTWYGRGAGKCATGKTTTMYWMCTCYGYTAATWGATGRAAYRYYGAWCTTMM 9720
                     *             .       .  .     .. .      .:    .    .      *

Bhutan-09015         WTGAYYMYYCRTTCRTKGACMCCYATSCRCYRGWMMTSRRKCAMCRTYRTKMYRMCYTSY 9780
Bhutan-09024         WTGAYYMYYSRTTCRTKGACMCCYATSCRCYRGWMMTSRRKCAMCRTYRTKMYRMCYTSY 9780
Bhutan-09027         WTGAYYMYYSRTTCRTKGACMCCYATSCRCYRGWMMTSRRKCAMCRTYRTKMYRMCYTSY 9780
Bhutan-09030         WTGAYYMYYSRTTCRTKGACMCCYATSCRCYRGWMMTSRRKCAMCRTYRTKMYRMCYTSY 9780
Bhutan-09005         WTGRCCATCSAWWSRKTARYASYTRWGCGTTGRAAMCMRAKYWMSRKTRYGCYRCCTYSY 9780
Indonesia-88035      WKRRCCATCSAWWSAGTARYASYTRWSYGYTGRAAMCMRAKYWMSGKTRYGCYRCYTYSY 9780
Indonesia-88045      WKRRCCATCSAWWSAGTARYASYTRWSYGYTGRAAMCMRAKYWMSGKTRYGCYRCYTYSY 9780
Indonesia-88065      WTGRCCATCSAWWSRKTARYASYTRWGCGTTGRAAMCMRAKYWMSRKTRYGCYRCYTYSY 9780
                     *.       .   .  ..   .    .        *  * *  *. . *   **    **

Bhutan-09015         GAAMWYKRMRMTCMGYKYACMYRRTCGMSYRTTAAGTSYATGAKRAWTGTARYRGCWTSG 9840
Bhutan-09024         GAAMWYKRMRMTCMGYKYACMYRRTCGMSYRTTAAGTSYATGAKRAWTGTARYRGCWTSG 9840
Bhutan-09027         GAAMWYKRMRMTCMGYKYACMCRRYCGMSYRTTAAGTSYATGAKRAWTGTARYRGCWTSG 9840
Bhutan-09030         GAAMWYKRMRMTCMGYKYACMCRRYCGMSYRTTAAGTSYATGAKRAWTGTARYRGCWTSG 9840
Bhutan-09005         RGRCTTTACGMKSMATKTRYMYGATYRRGTGYYWWSKCYWWRWTGTAYRYWRYGKYMYSR 9840
Indonesia-88035      RRRCTTTACGAKSMATKTRYMYGATYRRGTGYYWWSKCYWWRWTGTAYRYWRYGKYCYSR 9840
Indonesia-88045      RRRCTTTACGAKSMATKTRYMYGATYRRGTGYYWWSKCYWWRWTGTAYRYWRYGKYMYSR 9840
Indonesia-88065      RGRCTTTACGMKSMATKTRYMYGATYRRGTGYYWWSKCYWWRWTGTAYRYWRYGKYMYSR 9840
                           .    ..*. *   *       .      ...*    . :     **     *

Bhutan-09015         TYRRAGKMCRMMYRTRARAYASCAYGYTRCRGSCRTCGYKAYAACTTYWYRRGYTGYKYW 9900
Bhutan-09024         TYRRAGKMCRMMYRTGARAYASCAYGYTRCRGSCRTCGYKAYAACTTYWYRRGYTGYKYW 9900
Bhutan-09027         TYRRAGKMCRMMYRTRARAYASCAYGYTRCRKSCRTCGYKAYAACTTYWYRRGYTGYKYW 9900
Bhutan-09030         TYRRAGKMCRMMYRTGARAYASCAYGYTRCRKSCRTCGYKAYAACTTYWYRRGYTGYKYW 9900
Bhutan-09005         YYARRKGMSRMCCRKGRGGTCSYRCKTYRSRGSMRYARYKRCMRYKKYTTGASCWKTKYA 9900
Indonesia-88035      YYARRKGMSRMCCRKGRGGTCSYRCKTYRSRGSMRYARYKRCMRYKKYTTGASCWKTKYA 9900
Indonesia-88045      YYARRKGMSRMCCRKGRGGTCSYRCKTYRSRGSMRYAGYKRCMRYKKYTTGASCWKTKTA 9900
Indonesia-88065      YYARRKGMSRMCCRKGRGGTCSYRCKTYRSRGSMRYARYKRCMRYKKYTTGASCWKTKYA 9900
                      * *   *.** *.    . .*      *.* * * . **     ..*    .    *

Bhutan-09015         YGGTGTRTTWTWCMYYGRGACTYMYTTYATRTAWATGATACCGAYRGRTKCTYMCKYRRY 9960
Bhutan-09024         YGGTGTRTTWTWCMYYGRGACTYMYTTYATRTAWATGATACCGAYRGRTKCTYMCKYRRY 9960
Bhutan-09027         YGGTGTRTTWTWCMYYGRGACTYMYTTYATRTAWATGATACCGAYRGRTKCTYMCKYRRY 9960
Bhutan-09030         YGGTGTRTTWTWCMYYGRGACTYMYTTYATRTAWATGATACCGAYRGRTKCTYMCKYRRY 9960
Bhutan-09005         TAKYSWGYYTCTGCCYRASWYYCCYKWCRKAYMWRWKTCRYYKMTACGYKYKTAYKYGAC 9960
Indonesia-88035      TAKYSWGYYTCTGCCYRASWYYCCYKWCRKAYMWRWKTCRTYKMTACGYKYKTAYKYGAC 9960
Indonesia-88045      TAKYSWGYYTCTGCCYRASWYYCCYKWCRKAYMWRWKTCRYYKMTACGYKCTTAYKYGAC 9960
Indonesia-88065      TAKYSWGYYTCTGCCYRASWYYCCYKWCRKAYMWRWKTCRYYKMTACGYKYKTAYKYGAC 9960
                      .  .          *  .     *.   .   *   :           * .   **

Bhutan-09015         YKCGGTRYYAKRCKKCGGASGGGMRGACACGYGSCCRATMYCKMRACTTYYRTGGYAKMC 10020
Bhutan-09024         YKCGGTRYYAKRCKKCGGASGGGMRGACACGYGSCCRATMYCKMRACTTYYRTGGYAKMC 10020
Bhutan-09027         YKCGGTRYYAKRCKKCGGASGGGMRGACACGYGSCCRATMYCKMRACTTYYRTGGYAKMC 10020
Bhutan-09030         YKCGGTRYYAKRCKKCRGASGGGMRGACACGYGSCCRATMYCKMRACTTYYRTGGYAKMC 10020
Bhutan-09005         CGTKRWRTCRTGYGTSGKWGKRRAATWSCYRYTCMARRGMYMTCRRYCCYTGWKKYMTMS 10020
Indonesia-88035      CGTKRWRTCRTGYGTGGKWGKRRAATTGCYGYTCMARRGMYMTCRRYCCYTGWKKYMTMS 10020
Indonesia-88045      CGTKRWRTCRTGYGTGGKWGKRRAATTGCYGYTCMARRGMYMTCRRYCCYTGWKKYMTMS 10020
Indonesia-88065      CGTKRWRTCRTGYGTGGKWGKRRAATWSCYRYTCMARRGMYMTCRRYCCYTGWKKYMTMS 10020
```

```
                            *   .   .    .        .  * . .*  ** . *    *     * .*.

Bhutan-09015          GWGWCCCAYTKGMRRRGACGGRTGWMMRACYGYRYRRTRRWYRYYCGCGRKGTCGWYYRM 10080
Bhutan-09024          GWGWCCCAYTKGMRRRGACGGRTGWMMRACYGYRYRRTRRWYRYYCGCGRKGTCGWYYRM 10080
Bhutan-09027          GWGWCCCAYTKGMRRRGACGGRTGWMMRACYGYRYRRTRRWYRYYCGCGRKGTCGWYYRM 10080
Bhutan-09030          GWGWCCCAYTKGMRRRGACGGRTGWMMRACYGYRYRRTRRWYRYYCGCGRKGTCGWYYRM 10080
Bhutan-09005          RARASTTTYCTRARRRRMTSRAWTWAAGWYYTYGCGAWGATYRTTSSAARGRWYATTCRM 10080
Indonesia-88035       RARASTTTYCTRARRRRMTSRAWTWAAGWYYTYGCGAWGATYRTTSSAARGRWYATTCRM 10080
Indonesia-88045       RARASTTTYCTRARRRRMTSRAWTWAAGWYYTYGCGAWGATYRTTSSAARGRWYATTCRM 10080
Indonesia-88065       RARASTTTYCTRARRRRMTSRAWTWAAGWYYTYGCGAWGATYRTTSSAARGRWYATTCRM 10080
                          .  :* .  ***   .    *     * *        **  ....*    .   **

Bhutan-09015          RYSRAKYRGRYCGTRTKRRARSRTKYTRMRMTAATRMMYYGMRRYTRCASTWYTAYRSMC 10140
Bhutan-09024          RYSRAKYRGRYCGTRTKRRARSRTKYTRMRMTAATRMMYYGMRRYTRCASTWYTAYRSMC 10140
Bhutan-09027          RYSRAKYRGRYCGTRTKRRARSRTKYTRMRMTAATRMMYYGMRRYTRCASTWYTAYRSMC 10140
Bhutan-09030          RYSRAKYRGRYCGTRTKRRARSRTKYTRMRMTAATRMMYYGMRRYTRCASTWYTAYRSMC 10140
Bhutan-09005          RYGRWKYRARCYRYAYGRARRSAYKYYACAAKRRYACMCTKCRGTKAYWCCACYRYRCMA 10140
Indonesia-88035       RYGRWKYRARCYRYAYGRARRSAYTCYACAAKRRYACMCTKCRGTKAYWCCACYRYRCMA 10140
Indonesia-88045       RYGRWKYRARCYRYAYGRARRSAYKYYACAAKRRYACMCTKCRGTKAYWCCACYRYRCMA 10140
Indonesia-88065       RYGRWKYRARCYRYAYGRARRSAYKYYACAAKRRYACMCTKCRGTKAYWCCACYRYRCAA 10140
                      **.* ***.*       *  **  .      .     *    *  .   .     **. .

Bhutan-09015          YGYKGGSKSTCGATRWSCRYRSTMKRGAGGTTYCWTAMTRGCCTGRYRCMSRAYCYRRCY 10200
Bhutan-09024          YGYKGGSKSTCGATRWSCRYRSTMKRGAGGTTYCWTAMTRGCCTGRYRCMSRAYCYRRCY 10200
Bhutan-09027          YGYKGGSKSTCGATRWSCRYRSTMKRGAGGTTYCWTAMTRGCCTGRYRCMSRAYCYRRCY 10200
Bhutan-09030          YGYKGGSKSTCGATRWSCAYRSTMKRGAGGTTYCWTAMTRGCCTGRYRCMSRAYCYRRCY 10200
Bhutan-09005          TKYTRASGSKSSGWAASMGCACYMKAARRAYYCYWCGAKARYSWKRCRYMSGRYMCARAT 10200
Indonesia-88035       TKYTRASGSKSSGWAASMGCACYMKAARRAYYCYWCGAKARYSWKRCRYMSGRYMCARAT 10200
Indonesia-88045       TKYTRASGSGSSGWAASMGCACYMKAARRAYYCCWCGAKARYSWKRCRYMSGRYMCARAT 10200
Indonesia-88065       TKYTRASGSKSSGWAASMGCACYMKAARRAYYCYWCGAKARYSWKRCRYMSGRYMCARAT 10200
                        *. .* * ...   *    . ** .  .    * . .   .  * * **  *   *.

Bhutan-09015          GTTSTAYKYYSSKYYMYARKTCMAYGARCRGSRMYRWTRYTMGAMRWYWRRTYCYYAWGR 10260
Bhutan-09024          GTTSTAYKYYSSKYYMYARKTCMAYGARCRGSRMYRWTRYTMGAMRWYWRRTYCYYAWGR 10260
Bhutan-09027          GTTSTAYKYYSSKYYMYARKTCMAYGARCRGSRMYRWTRYTMGAMRWYWRRTYCYYAWGR 10260
Bhutan-09030          GTTSTAYKYYSSKYYMYARKTCMAYGARCRGSRMYRWTRYTMGAMRWYWRRTYCYYAWGR 10260
Bhutan-09005          RWYSKGTKYTGSKYYATRRKYMAMYKRASGRGAACRWYGTWMRRCAAYWGRWTYCYRWRG 10260
Indonesia-88035       RWYSKGTKYTGSKYYATRRKYMAMYKRASGRGAACRWYGTWMRRCAAYWGRWTYCYRWRG 10260
Indonesia-88045       RWYSKGTKYTGSKYYATRRKYMAMYKRASGRGAACRWYGTWMRRCAAYWGRWTYCYRWRG 10260
Indonesia-88065       RWYSKGTKYTGSKYYATRRKYMAMYKRASGRGAACRWYGTWMRRCAAYWGRWTYCYRWRG 10260
                         *.. ** .****   **    *   .  .   **    *     ** *    * *

Bhutan-09015          AKMYTSRYYKRYYTRYSMRGCCTTCTTWKAWRATRAKMSRWRKRKGRTTYCGTKRKRRGR 10320
Bhutan-09024          AKMYTSRYYKRYYTRYSMRGCCTTCTTWKAWRATRAKMSRWRKRKGRTTYCGTKRKRRGR 10320
Bhutan-09027          AKMYTSRYYKRYYTRYSMRGCCTTCTTWKAWRATRAKMSRWRKRKGRTTYCGTKRKRRGR 10320
Bhutan-09030          AKMYTSRYYKRYYTRYSMRGCCTTCTTWKAWRATRAKMSRWRKRKGRTTYCGTKRKRRGR 10320
Bhutan-09005          MKMYTCGCCKGCCWATGAAKMYYYYWYTTWWRWYGRKACGWGGRGGAYACYRGKRTGRRG 10320
Indonesia-88035       MKMYKCGCCKGCCWATGAAKMYYYYWYTTWWRWYGRKACGWGGRGRAYACYRGKRTGRRG 10320
Indonesia-88045       MKMYKCGCCKGCCWATGAAKMYYYYWYTTWWRWYGRKACGWGGRGRAYACYRGKRTGRRG 10320
Indonesia-88065       MKMYKCGCCKGCCWATGAAKMYYYYWYTTWWRWYGRKACGWGGRGGAYACYRGKRTGRRG 10320
                       ***..   *      .           . **    * . *  *    :    **. *

Bhutan-09015          AATYKRTTTKGAWTASARGRKTGATWKYMATWYRTTRYMATRYYGRWKGYWYRYYWWGCR 10380
Bhutan-09024          AATYKRTTTKGATTASARGRKTGATWKYMATWYRTTRYMATRYYGRWKGYWYRYYWWGCR 10380
```

```
Bhutan-09027         AATTKRTTTKGAWTASAGGRKTGATWKYMATWYRTTRYMATRYYGRWKGYWYRYYWWGCR 10380
Bhutan-09030         AATYKRTTTKGAWTASARGRKTGATWKYMATWYRTTRYMATRYYGRWKGYWYRYYWWGCR 10380
Bhutan-09005         WWYYTRYYWKKRWYRGAGTRTWSWYWGCARWWCGWYATMRWAYCKGTGRCWYRTTWWRSG 10380
Indonesia-88035      WWYYTRYYWKKRWYRGRGTRTWSWYWGCARWWCGWYATMRWAYCKGTGRCWYRTTWWRSG 10380
Indonesia-88045      WWYYTRYYWKKRWYRGRGTRTWSWYWGCARWWCGWYATMRWAYCKGTGRCWYRTTWWRSG 10380
Indonesia-88065      WWYYTRYYWKKRWYRGRGTRTWSWYWGCARWWCGWYATMRWAYCKGTGRCWYRTTWWRSG 10380
                         .*   *     .   *. .  *     *      *   *       ***  ** .

Bhutan-09015         GCGTAGWWYYRCSCRYTARYKYKGRTGARTATTACGYYYYMTRTMWSKCKYSACGYYTKA 10440
Bhutan-09024         GCGTAGWWYYRCSCRYTARYKYKGRTGARTATTWCGYYYYMTRTMWSKCKYSACGYYTKA 10440
Bhutan-09027         GCGTAGWWYYRCSCRYTARYKYKGRTGARTATTWCGYYYYMTRTMWSKCKYSACGYYTKA 10440
Bhutan-09030         GCGTAGWWYYRCSCRYTARYKYKGRTGARTATTWCGYYYYMTRTMWSKCKYSACGYYTKA 10440
Bhutan-09005         KSTYMRWTTWRYCAGYCGGCTYTKGCRTGWRYYAYSCYYTCKRYMACRYTTGRYCTTYGR 10440
Indonesia-88035      KSTYMRTTTWRYCAGYCGGCTYTKGCRTGWRYYAYSCYYTCKRYMACRYTTGRYCTTYGR 10440
Indonesia-88045      KSTYMRWTTWGYCAGYCGGCTYTKGCRTGWRYYAYSCYYTCKRYMACRYTTGGYCTTYGR 10440
Indonesia-88065      KSTYMRWTTWRYCAGYCGGCTYTKGCRTGWRYYAYSCYYTCKRYMACRYTTGRYCTTYGR 10440
                      .       :  .. * .  .*.    :       . **  .* * .: . .

Bhutan-09015         CKGSGRGTGMMYGYRRCKYASRRGSWTSGKYRSKGATGYSCCCARYCYYKSYTMRYRRYR 10500
Bhutan-09024         CKGSGRGTGMMYGYRRCKYASRRGSWTSGKYRSKGATGYSCCCARYCYYKSYTMRYRRYR 10500
Bhutan-09027         CKGSGRGTGMMYGYRRCKYASRRGSWTSGKYRSKGATGYSCCCARYCYYKSYTMRYRRYR 10500
Bhutan-09030         CKGSGRGTGMMYGYRRCKYASRRGSWTSGKYRSKGATGYSCCCARYCYYKSYTMRYRRYR 10500
Bhutan-09005         YKRCKGRYKCMYCYRRYGCWGGGCCTACTKYAGGCRYSYSMYGWACYTCKCTYAACAATG 10500
Indonesia-88035      YKRCKGRYKCMYCYRRYGCWGGGCCTACTKYAGGCRYSYSMTGWACYTCKCTYAACAATG 10500
Indonesia-88045      YKRCKGRYKCMYCCRRYGCWGGGCCTACTKYAGGCRYSYSMTGWACYTCKCTYAACAATG 10500
Indonesia-88065      YKRCKGRYKCMYCYRRYGCWGGGCCTACTKYAGGCRYSYSMYGWACYTCKCTYAACAATG 10500
                      * .      **  **    .   . :. ** .    .**         *.

Bhutan-09015         ACYWRTTRGRWCTRAASRKRTYAGAYRWMRGAYRGYAYYYAGYGRTMKYMYYKKTGMCCA 10560
Bhutan-09024         ACYWRTTRGRWCTRAASRKRTYAGAYRWMRGAYRGYAYYYAGYGRTMKYMYYKKTGMCCA 10560
Bhutan-09027         ACYWRTTRGRWCTRAASRKRTYAGAYRWMRGAYRGYAYYYAGYGRTMKYMYYKKTGMCCA 10560
Bhutan-09030         ACYWRTTRGRWCTRAASRKRTYAGAYRWMRGAYRGYAYYYAGYGRTMKYMYYKKTRMCCA 10560
Bhutan-09005         WGYAGYYARAWYWGRCGAKAGTCRRYRTMACRYARCWTCTRAYRRCCKYMYCKKTAMAYR 10560
Indonesia-88035      WGYAGYYARRAYWGRCGAKAGTCRRYATMAGRYARCWTCTRAYGRCCKYMYCKKYAMCYA 10560
Indonesia-88045      WGYAGYYARRAYWGRCGAKAGTCRRYATMAGRYARCWTCTRAYGRCCKYMYCKKYAMCYA 10560
Indonesia-88065      WGYAGYYARAWYWGRCGAKAGTCRRYRTMACRYARCWTCTRAYRRCCKYMYCKKTAMAYR 10560
                       *            .. *   .  *  *   *        .* *  **** **  *.

Bhutan-09015         TKRTYTYKYCARYTMRCTTRYTTYKCTRGAMWYCCYYRWRYCWATSRYSACTSAAYGCTA 10620
Bhutan-09024         TKRTYTYKYCARYTMRCTTRYTTYKCTRGAMWYCCYYRWRYCWATSRYSACTSAAYGCTA 10620
Bhutan-09027         TKRTYTYKYCARYTMRCTTRYTTYKCTRGAMWYCCYYRWRYCWATSRYSACTSAAYGCTA 10620
Bhutan-09030         TKRTYTYKYCARYTMRCTTRYTTYKCTRGAMWYCCYYRWRYCWATSRYSACTSAAYGCTA 10620
Bhutan-09005         YKRYYYYKCCWRTYAATKYGCYWCKYKRRMMTYTACTATGCTTACSRYSTCYGRATSCYT 10620
Indonesia-88035      TKRYYYYGCSARTYAATKYGCYWCKYKRRMMTYTACTATGCTTMCSRYSTMYGGRTSSYT 10620
Indonesia-88045      TKRYYYYGCSARTYAATKYGCYWCKYKRRMMTYTACTATGCTTMCSRYSTMYGGRTSSYT 10620
Indonesia-88065      YKRYYYYKCCWRTYAATKYGCYWCKYKRRMMTYTACTATGCTTACSRYSTCYGRATSCYT 10620
                      ** * *  . *     .      * .*  * * .          ****:  .   .. :

Bhutan-09015         RCRCWWAAYWTCACGAAYWRRGYWCYAGRYKACRYTACTKAAYMMGTRYGCRSAYCTGGA 10680
Bhutan-09024         RCRCWWAAYWTCACGAAYWRRGYWCYAGRYKACRYTACTKAAYMAGTRYGCRSAYCTGGA 10680
Bhutan-09027         ACRCWWAAYWTCACGAAYWRRGYWCYAGRYKACRYTACTKAAYMAGTRYGCRSAYCTGGA 10680
Bhutan-09030         RCRCWWAAYWTCACGAAYWRRGYWCYAGRYKACRYTACTKAAYMMGTRYGCRSAYCTGGA 10680
Bhutan-09005         GYAYWRWWYAWYRYKRRTARARYTSTRKATTMMATWCTYGRRTAAATGYRYGGRTSYKRR 10680
Indonesia-88035      GYAYWRWWYAWYRYKRRTARARYTSTRKATTMMATWCTYGRRYMARWRYGYGGRTSYKRR 10680
```

```
Indonesia-88045      GYAYWRWWYAWYRYKRRTARARYTSTRKATTMMATWCTYGRRYMARWRYGYGGRTSYKRR 10680
Indonesia-88065      GYAYWRWWYAWYRYKRRTARARYTSTRKATTMMATWCTYGRRTAARWGYRYGGRTSYKRR 10680
                         *   *          *  * .     .     .           *   .  .

Bhutan-09015         GRYAGKYCKYYRTMTYSWMYGGARRYRRRMRAGWTYSAMWWWYYYASCWTMASRRRGARG 10740
Bhutan-09024         GRYAGKYCKYYRTMTYSWMYGGARRYRRRMRAGWTYSAMWWWYYYASCWTMASRRRGARG 10740
Bhutan-09027         GRYAGKYCKYYRTMTYSWMYGGARRYRRRMRAGWTYSAMWWWYYYASCWTMASRRRGARG 10740
Bhutan-09030         GRYAGKYCKYYRTMTYSWMYGGARRYRRRMRAGWTYSAMWWWYYYASCWTMASRRRGARG 10740
Bhutan-09005         SGCGRTTTGYCGYCKYSWATKCMRATRRACRTKTKCSMMWTACTCRSSAYCWSRAGSRGS 10740
Indonesia-88035      SGCGGKTTGYCGYCTYSWATKSMRATRRACRAKWTYSMMWTACTCRSSAYCWSRAGSRGS 10740
Indonesia-88045      SGCGGKTTGYCGYCTYSWATKSMRATRRACRAKWTYSMMWTACTCRSSAYCWSRAGSRGS 10740
Indonesia-88065      SGCGRTTTGYCGYCKYSWATKCMRATRRACRTKTKCSMMWTACTCRSSAYCWSRAGSRGS 10740
                     .  . .   *    .***     *  **  *:  . * **      *.    **  .  .

Bhutan-09015         TWKYYRTKKYAGKSRYYMAMYYAMAKRRRTYYTAWTKTCYWATRSTYTRASTMTATATYT 10800
Bhutan-09024         TWKYYRTKKYAGKSRYYMAMYYAMAKRRRTYYTAWTKTCYWATRSTYTRASTMTATATYT 10800
Bhutan-09027         TWKYYRTTKYAGKSRYYAAMYYAMAKRRRTYYTAWTKTCYWATRSTYTRASTMTATATYT 10800
Bhutan-09030         TWKYYRTKKYAGKSRYYMAMYYAMAKRRRTYYTAWTKTCYWATRSTYTRASTMTATATYT 10800
Bhutan-09005         WWSTCGWKGYGATSACTCRATCRCGTRAGGTTYAWCGTMCARYAGWYAARGYAYMYRKTK 10800
Indonesia-88035      WWSTCGWKGYGATSACTCRATCRCGTRAGKTTYMWCGYMCARYAGWYAARGYAYMYRKTK 10800
Indonesia-88045      WWSTCGWKGCGATSACTCRATCRCGTRAGKTTYMWCGYMCARYAGWYAARGYAYMYRKTK 10800
Indonesia-88065      WWSTCGWKGYGATSACTCRATCRCGTRAGGTTYAWCGTMCARYAGWYAARGYAYMYRKTK 10800
                      *.    .  ...*          ..*       *         . *:  .      . .

Bhutan-09015         AYAYKAYGCRAKCAGAKYYYCRYYYGGTTGCYAKYAATRRATYKCGMWMCRMRTMYYSMT 10860
Bhutan-09024         AYAYKAYGCRAKCAGAKYYYCRYYYGGTAGCYAKYAATRRATYKCGMWMCRMRTMYYSMT 10860
Bhutan-09027         AYAYKAYGCRAKCAGAKYYYCRYYYGGTAGCYAKYAATRRATYKCGMWMCRMRTMYYSMT 10860
Bhutan-09030         AYAYKAYGCRAKCAGAKYYYCRYYYGGTTGCYAKYAATRRATYKCGMWMCRMRTMYYSMT 10860
Bhutan-09005         RCWCKRCAYAAGYWRRGTCTMRYTTGGKARCCWGCGGKARRTYKYRAWAYGYGWACCCMY 10860
Indonesia-88035      RCWCKRCAYARGYWRRGTCTMRYYYGGKARYCWGCGGTARRYYKYRAWAYAYGAACCCMY 10860
Indonesia-88045      RCWCKRCAYARGYWRRGTCTMRYYYGGKARYCWGCGGTARRYYKYRAWAYAYGAACCCMY 10860
Indonesia-88065      RCWCKRCAYAAGYWRRGTCTMRYTTCRKARCCWGCGGKARRTYKYRAWAYGYGWACCCMY 10860
                         *  .             **    .:      ... *  **   *         .*

Bhutan-09015         WTTGRYTGGSAYYRCTRMKYGKGCYAKWRRCRGCRTCTGCAMTTRARRKRYMAGYYGCSY 10920
Bhutan-09024         WTTGRYTGGSAYYRCTRMKYGKGCYAKWRRCRGCRTCTGCAMTTRARRKRYMAGYYGCSY 10920
Bhutan-09027         WTTGRYTGGSAYYRCTRMKYGKGCYAKWRRCRGCRTCTGCAMTTRARRKRYMAGYYGCSY 10920
Bhutan-09030         WTTGRYTGGSAYYRCTRMKYGKGCYATTRRCRGCRTCTGCAMTTRARRKRYMAGYYGCSY 10920
Bhutan-09005         WYTRATCKGGRTTAMTRCKYAKGYYRGAGGYRASKYYYKMWACYGRARWATCAACCRSCT 10920
Indonesia-88035      WYWGACTKKGRTTAMYRCKYAKRYYRGAGGYRASKYYYKMWMCYGRARKATCAACCRSCT 10920
Indonesia-88045      WYWGACTKKGRTTAMYRCKYAKRYYRGAGGYRASKYYYKMWMCYGRARKATCAACCRSCT 10920
Indonesia-88065      WYTRATCKGGRTTAMTRCKYAKGYYRGAGGYRASKYYYKMWACYGRARWATCTACCRSCT 10920
                     *        .      * **.*  *      *..:            *    :.   ..

Bhutan-09015         TTCATYWMCSAMTWYWRARGTMCTCSGARSYYRSRRYGYMRYKAKATRRYYRYYATCGAA 10980
Bhutan-09024         TTCATYWMCSAMTWYWRARGTMCTCSGARSYYRSRRYGYMRYKAKATRRYYRYYATCGAA 10980
Bhutan-09027         TTCATYWMCSAMTWYWRARGTMCTCSGARSYYRSRRYGYMRYKAKATRRYYRYYATCGAA 10980
Bhutan-09030         TTCATYWMCSAMTWYWRARGTMCTCSGARSYYRSRRYGYMRYKAKATRRYYRYYATCGAA 10980
Bhutan-09005         YYMAYTTAYGRCCACARRGKCCYTTGRGAGCTACGGCKTMRTGRTRWARCTRCYRWYKWR 10980
Indonesia-88035      YYMRYTTAYGACCACARRGKCCYYCGRGAGCTACGGCKTMRTGRTRWARCTRCYRWYKWR 10980
Indonesia-88045      YYMRYTTAYGRCCACARRGKCCYYCGRGAGCTACGGCKTMRTGATRWARCTRCYRWYKWR 10980
Indonesia-88065      YYMAYTTAYGRCCACARRGKCCYTTGRGAGCTACGGCKTMRTGRTRWARCTRCYRWYKWR 10980
                              .      *        . . .   .     **   .   *  * *
```

```
Bhutan-09015        RMYRARRSKCTWRRCCWMWSRCGYGGWYWCWGRSWTRGAWCTSMTRYRAGATARMRYRAG 11040
Bhutan-09024        RMYRARRSKCTWRRCCWMWSRCGYGGWYWCWGRSWTRGAWCTSMTRYRAGATARMRYRAG 11040
Bhutan-09027        RMYRARRSKCTWRRCCWMWSRCGYGGWYWCWGRSWTRGAWCTSMTRYRAGATARMRYRAG 11040
Bhutan-09030        RMYRARRSKCTWRRCCWMWSRCGYGGWYWCWGRSWTRGAWCTSMTRYRAGATARMRYRAG 11040
Bhutan-09005        RCYGRRGGGYYTGRSSTAASGYACTRATYYASRSTKGCGWYYCCYRYRRKWYGGAGYAWR 11040
Indonesia-88035     RCYGRRGGKYYTGRSSTAASGYACTRATYYASRSTKGCGWYYCCYRYRRKWYGGAGYAWR 11040
Indonesia-88045     RCYGRRGGGYYTGRSSTAASGYACTRATYYASRSTGGCGWYYCCYRYRRKWYGGAGYAWR 11040
Indonesia-88065     RCYGRRGGKYYTGRSSTAASGYACTRATYYASRSTKGCGWYYCCYRYRRKWYGGAGYAWR 11040
                    * *  * .     *..   *  .     :  .**    .*  .  ***    .   *

Bhutan-09015        RCSKKYRSSRGRARATRAMMARTMAAYGGWCRAKRWTAGRMAYCGTYYAYYYRCTMYRGG 11100
Bhutan-09024        RCSKKYRSSRGRARATRAMMARTMAAYGGWCRAKRWTAGRMAYCGTYYAYYYRCTMYRGG 11100
Bhutan-09027        RCSKKYRSSRGRRRATRAMMARTMAAYGGWCRAKRWTAGRMAYCGTYYAYYYRCTMYRGG 11100
Bhutan-09030        RCSKKYRSSRGRRRATRAMMARTMAAYGGWCRAKRWTAGRMAYCGTYYAYYYRCTMYRGG 11100
Bhutan-09005        ATCGTCRCSRCGGGWYRWMMRGWCMMCASAYARKAAAWKGMWTMRWYYWTTCGYYCCGKR 11100
Indonesia-88035     ATCGTCRCSRCGGGWYRWMMRGWCMMCASAYARKAAAWKGMWTMRWYYWTTCGYYCCGKR 11100
Indonesia-88045     ATCGTCRCSRCGGGWYRWMMRGWCMMCASAYARKAAAWKGMWTMRWYYWTTCGYYCCGKR 11100
Indonesia-88065     ATCGTCRCSRCGGGWYRWMMRGWCMMCASAYARKAAAWKGMWTMRWYYWTTCGYYCCGKR 11100
                      . . *.**      * **       ..    *  :   *     **

Bhutan-09015        RTRRKRKYYATCTTGGTAMSRRWKMAYTRATKYSSKTWKYRYRARAYSYGYKAYSGRYYA 11160
Bhutan-09024        RTRRKRKYYATCTTGGTAMSRRWKMRYTRATKYSSKTWKYRYRARAYSYGYKAYSGRYYA 11160
Bhutan-09027        RTRRKRKYYATCTTGGTAMSRRWKMRYTRATKYSSKTWKYRYRARAYSYGYKAYSGRYYA 11160
Bhutan-09030        RTRRKRKYYATCTTGGTAMSRRWKMRYTRATKYSSKTWKYRYRARAYSYGYKAYSGRYYA 11160
Bhutan-09005        AYRGTRKYYRYAKTRAGRMCRGWKMATCGGWGTCSGCTTYGYWWAWCCTKCKRCCSGTTR 11160
Indonesia-88035     AYRGTRKYYRYAKTRAGAMCRGWKMATCGGWGTCSGCTTYGYWWAWCCTKCKRCCSGTTR 11160
Indonesia-88045     AYRGTRKYYRYAKKRAGAMCAGWKMATCGGWGTCSGCTTYGYWWAWCCTKCKRCCSGTTR 11160
Indonesia-88065     AYRGTRKYYRYAKKRAGRMCRGWKMATCGGWGTCSGCTTYGYWWAWCCTKCKRCCSGTTR 11160
                      * .****  ... .  *.  ***    .   .*   .* *     .   *  ..

Bhutan-09015        KRCTTGTRGARTYTAKTTWYWRYRYRMGYARTSTKYYCGGYCYYGYYRRYYRGWTWYCRK 11220
Bhutan-09024        KRCTTGTRGARTYTAKTTWYWRYRYRMGYARTSTKYYCGGYCYYGYYRRYYRGWTWYCRK 11220
Bhutan-09027        KRCTTGTRGARTYTAKTTWYWRYRYRMGYARTSTKYYCGGYCYYGYYRRYYRGWTWYCRK 11220
Bhutan-09030        KRCTTGTRGARTYTAKTTWYWRYRYRMGYARTSTKYYCGGYCYYGYYRRYYRGWTWYCRK 11220
Bhutan-09005        KGSYYRYARRRWCKWKKYWYWACGYRARTWAKCYKCYYAKYTCCAYYRGCYRRWYAYYRK 11220
Indonesia-88035     KGSYYRYARRRWCKWKKYWYWACGYRARTWAKCYKCYYAKYTCCGYYRGCYRRWYAYYRK 11220
Indonesia-88045     KGSYYRYARRRWCKWKKYWYWACGYRARTWAKCYKCYYAKYTCCGYYRGCYRRWYAYYRK 11220
Indonesia-88065     KGSYYRYARRRWCKWKKYWYWACGYRARTWAKCYKCYYAKYTCCAYYRGCYRRWYAYYRK 11220
                    * .       *  . *. ***   **     .. * * . *   .***  ** *  * **

Bhutan-09015        CYRGCWTGYYYYTCYTRAGTGTGRCRCRTTMRASMRYGYRCTYYTATCAAYSKRYTTACG 11280
Bhutan-09024        CYRGCWTGYYYYTCYTRAGTGTGRCRCRTTMRASMRYGYRCTYYTATCAAYSKRYTTACG 11280
Bhutan-09027        CYRGCWTGYYYYTCYTRAGTGTGRCRCRTTMRASMRYGYRCTYYTATCAAYSKRYTTACG 11280
Bhutan-09030        CYRGCWTGYYYYTCYTRAGTGTGRCRCRTTMRASMRYGYRCTYYTATCAAYSKRYTTACG 11280
Bhutan-09005        CTARYAYRYTYYKYTWGRRYRKRGMGYRYACGMCMGYRYRYCYTYMYYTGCSTGTYTASR 11280
Indonesia-88035     ATARYAYRYTYYKYTWGRRYRKRGMGYRYACGMCMGYRYRYCYTYMYYTGCSTGTYYRSR 11280
Indonesia-88045     ATARYAYRYTYYKYTWGRRYRKRGMGYRYACGMCMGYRYRYCYTYMYYTGCSTGTYYRSR 11280
Indonesia-88065     CTARYAYRYTYYKYTWGRRYRKRGMGYRYACGMCMGYRYRYCYTYMYYTGCSTGTYYRSR 11280
                    .       * **.        .     * :   .* * **  *     :. *.     .

Bhutan-09015        RYSSRTMWGKCKTATYRRRGKRYGYWKCYTKTYWYYGWCCRAATRMGTTRARTCWASCKA 11340
Bhutan-09024        RYSSRTMWGKCKTATYRRRRKRYGYWKCYTKTYWYYGWCCRAATRMGTTRARTCWASCKA 11340
Bhutan-09027        RYSSRTMWGKCKTATYRRRRKRYGYWKCYTKTYWYYGWCCRAATRMGTTRARTCWASCKA 11340
Bhutan-09030        RYSSRTMWGKCKTATYRRRRKRYGYWKCYTKTYWYYGWCCRAATRMGTTRARTCWASCKA 11340
```

```
Bhutan-09005        RYKSRWMTSKYKYRTTGRRAGRYAYWKSCWKYYWYYAWYYRRRYRMKYYGMRYYWGGYGR 11340
Indonesia-88035     RYKSRWMTSKYKYRWTGRRAGRYAYWKSCWKTYWYYAWYYRRRYRMKYYGMRYYWGSYGA 11340
Indonesia-88045     RYKSRWMTSKYKYRWTGGRAGRYAYWKSCWKTYWYYAWYYRRRYRMKYYGMRYYWGSYGA 11340
Indonesia-88065     RYKSRWMTSKYKYRTTGRRAGRYAYWKSCWKYYWYYAWYYRRRYRMKYYGMRYYWGGYGR 11340
                    **.** * .* *       *  **.***.  * ****.*  *    **      *  *..

Bhutan-09015        CYTCRGSGCAGKWSTCGTGRYRTRTYGACTAWKGYGCCTTWCGYTKYYWAASAKYMYRTG 11400
Bhutan-09024        CYTYRGSGCAGKWSTCGTGRYRTRTYGACTAWKGYGCCTTWCGYTKYYWAASAKYMYRTG 11400
Bhutan-09027        CYTYRGSGCAGKWSTCGTGRYRTRTYGACTAWKGYGCCTTWCGYTKYYWAASAKYMYRTG 11400
Bhutan-09030        CYTCRGSGCAGKWSTCGTGRYRTRTYGACTAWKGYGCCTTWCGYTKYYWAASAKYMYRTG 11400
Bhutan-09005        YCYCRASRTAGGTSWYRWRAYRYACYRMYARTKRTRMSYKAYCYYGYCARRGRGYAYRYK 11400
Indonesia-88035     CCYCRASRTCRKASWYRTRRYRYACYRMYARTKRTRCSYKAYCYYGYCARRGRGYAYRYK 11400
Indonesia-88045     CCYCRASRTCRKASWYRTRRYRYACYRMYARTKRTRCSYKAYCYYGYCARRGRGYAYRYK 11400
Indonesia-88065     YCYCRASRTAGGTSWYRWRAYRYACYRMYARTKRTRMSYKAYCYYGYCARRGRGCAYRYK 11400
                        *.*  .   *      **   *   :  *    . .   *  *     .    **

Bhutan-09015        CWWGRRRMKAWCWTCGCMTAASSWGRMTGAAYGYGSAGYCWRMRRRGYTMGWCRTRTRTC 11460
Bhutan-09024        CWWGRRRMKAWCWTCGCMTAASSWGRMTGAAYGYGSAGYCWRMRRRGYTMGWCRTRTRTC 11460
Bhutan-09027        CWWGRRRMKAWCWTCGCMTAASSWGRMTGAAYGYGSAGYCWRMRRRGYTMGWCRTRTRTC 11460
Bhutan-09030        CWWGRRRMKAWCWTSRCMTAASSWGRMTGAAYGYGSAGYCWRMRRRGYTMGWCRTRTRTC 11460
Bhutan-09005        MTTKARRMKGTYWYCGAMARTSCARRCCRWRCKCKSMRYATAAGRRKCYCSWTRCRYRCT 11460
Indonesia-88035     MTTKARRMKGTYWYCGAMARTSCAGRCCGARCKCKSMRYATAAGRRKCYCSWTGCRYRCT 11460
Indonesia-88045     MTTKARRMKGTYWYCGAMARTSCAGRCCGARCKCKSMRYATAAGRRKCYCSWTRCRYRCT 11460
Indonesia-88065     MTTKARRMKGTYWYCGAMARTSCARRCCRWRCKCKSMRYATAAGRRKCYCSWTRCRYRCT 11460
                         ****.  * . .*: :*.  *          *  *.    **     .*   * *

Bhutan-09015        KTKAMYMMMGGMYTASRRYYMMAYWRTTYAACMKCATKYYRACYMYTAYMWTGKAYTSGW 11520
Bhutan-09024        KTKAMYMMMGGMYTASRRYYMMAYWRTTYAACMKCATKYYRACYMYTAYMWTGKAYTSGW 11520
Bhutan-09027        KTKAMYMMMGGMYTASRRYYMMAYWRTTYAACMKCATKYYRACYAYTAYMWTGKAYTSGW 11520
Bhutan-09030        KTKAMYMMMGGMYTASRRYYMMAYWRTTYAACMKCATKYYRACYMYTAYMWTGKAYTSGW 11520
Bhutan-09005        KTTGCYCMCARCCCCSAGCCAAMCARWKTRGYATSMGGTCAWATCYYMYMTKKTGTKSGA 11520
Indonesia-88035     KYTGCYCMCARCCCCSAGCCAAMCARWKTRRYMKSMGGTCAACTCYYMYMTKKTGTKSRA 11520
Indonesia-88045     KYTGCYCMCARCCCCSAGCCAAMCARWKTRRYMKSMGGTCAACTCYYMYMTKKTGTKSRA 11520
Indonesia-88065     KTTGCYCMCARCCCCSAGCCAAMCARWKTRGYATSMGGTCAWATCYYMYMTKKTGTKSGA 11520
                    * .. * * .    .*         * .      ..        .  *  ** . .. .*

Bhutan-09015        KARGYWGWGRYYWRYRTYMRYYWARRYGACARTWARGYYKYGRCMYWYRCTGYACCMCTM 11580
Bhutan-09024        KARGYWGWGRYYWRYRTYMRYYWARRYGACMRTWARGYYKYGRCMYWYRCTGYACCMCTM 11580
Bhutan-09027        KARGYWGWGRYYWRYRTYMRYYWARRYGACMRTWARGYYKYGRCMYWYRCTGYACCMCTM 11580
Bhutan-09030        KARGYWGWGRYYWRYRTYMRYYWARRYGACMRTWARGYYKYGRCMYWYRCTGYACCMCTM 11580
Bhutan-09005        KGGRCARWSRYYWRTGYTARYTWGRGTARCARWWMRGTYKTSGYMYTCGYYRYWYSMSYM 11580
Indonesia-88035     KGGRCARWSRYYWRTGYTARCTWAGRYAGTCRWWMRATYKTSGYMYTCGCYRYWYSMSYM 11580
Indonesia-88045     KGGRCARWSRYYWRTGYTARYTWAGRYAGTCRWWMRATYKTSGYMYTCGCYRYWYSMSYM 11580
Indonesia-88065     KGGRCARWSRYYWRTGYTARYTWGRGTARYMRWWMRGTYKTSGYMYTCGYYRYWYSASYM 11580
                    *.      *.*****      *  *.    .   * * *. ** .  **       *  . . *

Bhutan-09015        MWCYTYTYWWTGGYKSGWYRTRRMYYRKYTGKGRKCARATYGRARAGCYGYTRYYYRYYA 11640
Bhutan-09024        MWCYTYTYWWTGGYKSGWYRTRRMYYRKYTGKGRKCARATYGRARAGCYGYTRYYYRYYA 11640
Bhutan-09027        MWCYTYTYWWTGGYKSGWYRTRRMYYRKYTGKGRKCARATYGRARAGCYGYTRYYYRYYA 11640
Bhutan-09030        MWCYTYTYWWTGGYKSGWYRTRRMYYRKYTGKGRKCARATYGRARAGCYGYTRYYYRYYA 11640
Bhutan-09005        CTSYWTKTTTYKSCTSRTYRCRAAYCATTYSTRRKMAAWYTRGAGMSYYRCKAYCTATCM 11640
Indonesia-88035     CTSYTTKTTTYKSCTSRWYRCRAAYCATTYSTRRKMRATCTAGGGMSYYRCKAYCTATCM 11640
Indonesia-88045     CTSYTTKTTTYKSCTSRWYRCRAAYCATTYSTRRKMRATCTAGGGMSYYRCKAYCTATCM 11640
Indonesia-88065     CTSYWTKTTTYKSCTSRTYRCRAAYCATTYSTRRKMAAWYTRGAGMSYYRCKAYCTATCM 11640
```

```
                       .*  .     . .*  ** *  *  .  .. **         .  . *  . *

Bhutan-09015          GKYYTRAKAAYMYGTGRCGRCCTGWGGCKGRRYWAMRTCCTTRGGCTCGGGRCGYWYSTY 11700
Bhutan-09024          GKYYTRAKAAYMYGTGRCGRCCTGWGGCKGRRYWAMRTCCTTRGGCTCGGGRCGYWYSYY 11700
Bhutan-09027          GKYYTRATAAYMYGTGRCGRCCTGWGGCKGRRYWAMRTCCTTRGGCTCGGGRCGYWYSYY 11700
Bhutan-09030          GKYYTRATAAYMYGTGRCGRCCTGWGGCKGRRYWAMRTCCTTRGGCTCGGGRCGYWYSYY 11700
Bhutan-09005          KGYCYRWSMWCATRYRGYCAMMYRWKKYKRRRTWWMAKYYYWRRRYGCKSTAAATWTGTC 11700
Indonesia-88035       KGYCYRASMWCMTRYRGYSAMMYRWKKYKRRRTWWMAKYYYWRRRYGAKSTAMATWTGTC 11700
Indonesia-88045       KGYCYRASMWCMTRYRGYSAMMYRWKKYKRRRTWWMAKYYYWRRRYGAKSTAMATWTGTC 11700
Indonesia-88065       KGYCYRWSMWCMTRYRGYCAMMYRWKKYKRRRTWWMAKYYYWRRRYGCKSTAAATWTGTC 11700
                        *  * .                  *   * ** * * .    *     . .    . * .

Bhutan-09015          YYRACSTCRTSWRGKYTYARYGGGRWACRAYTTKTTGRGAYRRKYAGSGTGKRRGRATRY 11760
Bhutan-09024          YYRACSTCRTSWRGKYTYARYGGGRWACRAYTTKTTGRGAYRRKYAGSGTGKRRGRATRY 11760
Bhutan-09027          YYRACSTCRTSWRGKYTYARYGGGRWACRAYTTKTTGRGAYRRKYAGSGTGKRRGRATRY 11760
Bhutan-09030          YYRACSTCRTSWRGKYTYARYGGGRWACRAYTTKTTGRGAYRRKYAGSGTGKRRGRATRY 11760
Bhutan-09005          YYAGASYTRYCWAGTYKYRRCGGKRYTMRWTGCTYYRARWTGAKYRKCRKRTARSRGWAY 11760
Indonesia-88035       YYAGASYTRYCWARTYKCRACRRKRYTMRTTGCTYYRARWTGAKYRKCRKRTARSRGWAY 11760
Indonesia-88045       YYAGASYTRYCWARTYKYRACRRKRYTMRTTGCTYYRARWTGAKYRKCRKRTARSRGWAY 11760
Indonesia-88065       YYAGASYTRYCWAGTYKYRRCGGKRYTMRWTGCTYYRARWTGAKYRKCRKRTARSRGWAY 11760
                      ** ..*  * .*  .*.        *:: *     .         **  . . . *.*.  *

Bhutan-09015          RRYSKYSACATYRYYYRAKMCSTRMWYYTCRTWATAAWRARCRYKKMYAAWGCYGTYKSR 11820
Bhutan-09024          RRYSKYSACATYGTYYRATMCSTRMWYYTCRTWATAAWRARCRYKKMYAAWGCYGTYKSR 11820
Bhutan-09027          RRYSKYSACATYGTYYRAKMCSTRMWYYTCRTWATAAWRARCRYKKMYAAWGCYGTYKSR 11820
Bhutan-09030          RRYSKYSACATYGTYYRAKMCSTRMWYYTCRTWATAAWRARCRYKKMYAAWGCYGTYKSR 11820
Bhutan-09005          AGTSGCCGTRKYRYTTGTTCMSYGATYCCTAYAGTAWTRRGSGYKKCTRMARYCSYCKCR 11820
Indonesia-88035       AGTGGCCGTRKYRYTTGTTCCSYGATYCCTRYAGWWWTRRGSGYKKCTRMARYCSYCKCR 11820
Indonesia-88045       AGTGGCCGTRKYRYTTGWTCCSYGATYCCTRYAGWWWTRRGSGYKKCTRMARYCSYCKCR 11820
Indonesia-88065       AGTSGCCGTRKYRYTTGTTCMSYGATYCCTAYAGTAWTRRGSGYKKCTRMARYCSYCKCR 11820
                         .  ..  .*       .  *    *       .     *  . ***         .  *.*

Bhutan-09015          WCYSYMYYTCTGAYYAYARRGTARTSTGGCWMTTRSRWMYTRCSSGTGKKRAAYYYGMCA 11880
Bhutan-09024          WCYSYMYYTCTGAYYAYAAGGTARTSTGGCWMTTRSRWMYTRCSSGTGKKRAAYYYGMCA 11880
Bhutan-09027          WCYSYMYYTCTGAYYAYARRGTARTSTGGCWMTTRSRWMYTRCSSGTGKKRAAYYYGMCA 11880
Bhutan-09030          WCYSYMYYTCTGAYYAYARRGTARTSTGGCWMTTRSRWMYTRCSSGTGKKRAAYYYGMCA 11880
Bhutan-09005          AYYGYACTYTWRMYTATMARRYRAKGYRSAAMGKGCAAMCYAYGCRWSKTRRAYYCSASW 11880
Indonesia-88035       AYYGYACTYTWRMYTWTMAGRYRAKGYRSAAMGKGCAAMCYAYGCRWSKTRRRYYCSASW 11880
Indonesia-88045       AYYGYACTYTWRMYTWTMAGRYRAKGYRSAAMGKGCAAMCYAYGCRWSKTRRRYYCSASW 11880
Indonesia-88065       AYYGYACTYTWRMYTATMAGRYRAKGYRSAAMGKGCAAMCYAYGCRWSKTRRAYYCSASW 11880
                        *.*        *           ..  .. * . .  *     ..  .*.*  ** . .

Bhutan-09015          KWTRGTRYKWGKTRYRWARTWCRYAYMGYCSSRSAGAMWYYARTTAWAYCTWGCWAMRRA 11940
Bhutan-09024          KWTRGTRYKWGKTRYRWARTWCRYAYMGYCSSRSAGAMWYYARTTAWAYCTWGCWAMRRA 11940
Bhutan-09027          KWTRGTRYKWGKTRYRWARTWCRYAYMGYCSSRSAGAMWYYARTTAWAYCTWGCWAMRRA 11940
Bhutan-09030          KWTRGTRYKWGKTRYRWARTWCRYAYMGYCSSRSAGAMWYYARTTAWAYCTWGCWAMRRA 11940
Bhutan-09005          GWWGKKAYKARGCRTATRRYTTRCGCARCYGCAGCRRCWYTWRACWTMCMYARYTWMAAG 11940
Indonesia-88035       GWWGKTRYKARKCRTATRRYTTRCGCARCYGSRGCRRCWYTWRACWTMCMYWGYTWMAAG 11940
Indonesia-88045       GWWGKTRYKARKCRTATRRYTTRCGCARCYGSRGCRRCWYTWRACWTMCMYWGYTWMAAG 11940
Indonesia-88065       GWWGKKAYKARGCRTATRRYTTRCGCARCYGCAGCRRCWYTWRACWTMCMYARYTWMAAG 11940
                       *   . **    *    *   * .      .. ..   **  *:             *  .

Bhutan-09015          RAMYRGMWTCGAATGCTTCCRYRGATTKYTMKAGTMKYGTACGAARSWCGYRRYRRWYMG 12000
Bhutan-09024          RAMYRGMWTCGAATGCTTCCRYRGATTKYTMKAGTMKYGTACGAARSWCGYRRYRRWYMG 12000
```

```
Bhutan-09027        RAMYRGMWTCGAATGCTTCCRYRRMTTKYTMKAGTMKYGTACGAARSWCGYRRYRRWYMG 12000
Bhutan-09030        RAMYRGMWTCGAATGCTTCCRYRRMTTKYTMKAGTMKYGTACGAARSWCGYRRYRRWYMG 12000
Bhutan-09005        GRMYRRCAWYRRGYKMGYYYGYARMCTGTWCTWRTAGYKYGTSARRCAMKYGGCRRTYMA 12000
Indonesia-88035     GGMYRRCAWTRRGYKMGYYCGYAGACWGTWCTWRKAGCKYGYSTRRCAMKYGGCRRTYMA 12000
Indonesia-88045     GGMYRRCAWTRRGYKMGYYCGYAGACWGTWCTWRKAGCKYGYSTRRCAMKYGGCRRTYMA 12000
Indonesia-88065     GRMYRRCAWYRRGYKMGYYYGYAGACTGTWCTWRTAGYKYGTSARRCAMKYGGCRRTYMA 12000
                      ***       .        *         .  .     . .: *.   *   ** **.

Bhutan-09015        AWYYSGYYYMGKGGAYGCGRCCKATTMARMKYGGYRRAKKWGCARYYSYRGYCTMASRTW 12060
Bhutan-09024        AWYYSGYYYMGKGGAYGCGRCCKATTMARMKYGGYRRAKKWGCARYYSYRGYCTMASRTW 12060
Bhutan-09027        AWYYSGYYYMGKGGAYGCGRCCKATTMARMKYGGYRRAKKWGCARYYSYRGYCTMASRTW 12060
Bhutan-09030        AWYYSGYYYMGKGGACGCGRCCKATTMARMKYGGYRRAKKWGCARYYSYRGYCTMASRTW 12060
Bhutan-09005        CWYYSTYCTCKGKRWTGYKAGYGRCAARGAGCGAYRRMKKTATRGYYGCRSYMYMWSGYA 12060
Indonesia-88035     CWYYSTYCTCKGKRWTAYKAGCGRTAAAGAGCRAYRRMKTTATRGYYGCRSYMYMWSGYA 12060
Indonesia-88045     CWYYSTYCTCKGKRWTAYKAGCGRTAAAGAGCRAYRRMKTTATRGYYGCRSYMYMWSGYA 12060
Indonesia-88065     CWYYSTYCTCKGKRWTGYKAGYGRCAARGAGCGAYRRMKKTATRGYYGCRSYMYMWSGYA 12060
                    .**** *         .        :       .*** *. .   **. *.*  * *

Bhutan-09015        GYRACCRMYKGRKYCAGCYGMGGKYAGTSATTWYMKYYGTTGYSSTYYYSMYGGKMYGSA 12120
Bhutan-09024        GYRACCRMYKGRKYCAGCYGMGGKYAGTSATTWYMKYYGTTGYSSTYYYSMYGGKMYGSA 12120
Bhutan-09027        GYRACCRMYKGRKYCAGCYGMGGKYAGTSATTWYMKYYGTTGYSSTYYYSMYGGKMYGSA 12120
Bhutan-09030        GYRACCRMYKGRKYCAGCYGMGGKYAGTSATTWYMKYYGTTGYSSTYYYSMYGGKMYGSA 12120
Bhutan-09005        GYRTCTAMYTSRKYYGKYYRMRATCRRYGRYTACMTTYCWYKTGGCYTYSCTGKTMYKGR 12120
Indonesia-88035     RYRWYTRMYTSRKYTGKYYRMRATCRGTGRYCTCMKTYSWYKTGGCYTYSCTKKTMYKGG 12120
Indonesia-88045     RYRWYTRMYTSRKYTGKYYRMRATCRGTGRYCTCMKTYSWYKTGGCYTYSCTKKTMYKGG 12120
Indonesia-88065     GYRTCTAMYTSRKYYGKYYRMRATCRRYGRYTACMTTYCWYKTGGCYTYSCTGKTMYKGR 12120
                     **    **..*** .  * * ..    .     *. *     .. * **    .** .

Bhutan-09015        RYWTMGTKSTAYYGCKAYRTYRTCKWRGYGCYRYTRTRKRCWAGTCYKWGRTWATRTMYG 12180
Bhutan-09024        RYWTMGTKSTAYYGCKAYRTYRTCKWRGYGCYRYTRTRKRCWAGTCYKWGRTWATRTMYG 12180
Bhutan-09027        RYWTMGTKSTAYYGCKAYRTYRTCKWRGYGCYRYTRTRKRCWAGTCYKWGRTWATRTMYG 12180
Bhutan-09030        RYWTMGTKSTAYYGCKAYRTYRTCKWRGYGCYRYTRTRKRCWAGTCYKWGRTWATRTMYG 12180
Bhutan-09005        RYWYCGTKSCMYCTYKRYRYYAKYKTGACKMTRCYAKAKGYWRAYGYTWSAKWRWGYMYA 12180
Indonesia-88035     RYAYCKYKSCMYCTYKRYRYYAKYKTGACKMTRCYAKAKGYWRAYGYTWSAKWRWGYMYA 12180
Indonesia-88045     RYAYCKYKSCMYCTYKRTRYYAKYKTGACKMTRCYAKAKGYWRAYGYTWSAKWRWGYMCA 12180
Indonesia-88065     RYWYCGTKSCMYCTYKRYRYYAKYKTGACKMTRCYAKAKGYWRAYGYTWSAKWRWGYMCA 12180
                    **     **  *   *  * * . *  .    *   . *  * .  *.*. .*    * .

Bhutan-09015        WMCCKWYCRMRGMTGWTRYKRAARGTGYWYYGYYMKRSMWTGGTYAYTWSRCMRYMACGA 12240
Bhutan-09024        WMCCKWYCRMRGMTGWTRYKRAARGTGYWYYGYYMKRSMWTGGTYAYTWSRCMRYMACGA 12240
Bhutan-09027        WMCCKWYCRMRGMTGWTRYKRAARGTGYWYYGYYMKRSMWTGGTYAYTWSRCMRYMACGA 12240
Bhutan-09030        WMCCKWYCRMRGMTGWTRYKRAARGTGYWYYGYYMKRSMWTGGTYAYTWSRCMRYMACGA 12240
Bhutan-09005        TCYSGWYYGARKCWRWYRYTARARACRCAYYSTYCTRCATYKAATRYGWSGMARCAGYRT 12240
Indonesia-88035     TCYSGWYYGMRKCWRWYRTTAGRAACRCAYYSTYCTRCATYKATTRYGWSGMAACARYRT 12240
Indonesia-88045     TCYSGWYYGMRKCWRWYRTTAGRAACRCAYYSTYCTRCATYKATTRYGWSGMARCARYRT 12240
Indonesia-88065     TCYSGWYYGARKCWRWYRYTAGARACRCAYYSTYCTRCATYKAATRYGWSGMARCAGYRT 12240
                       . **   *    * * .    .    **. * .*.    .:  * **         :

Bhutan-09015        GAASCYYGACGGYYGGYRSWCWCCKYWTYYWMTYTASKRTAYMCGCGKKYYKARRKWRTG 12300
Bhutan-09024        GAASCYYGACGGYYGGYRSWCWCCKYWTYYWMTYTASKRTAYMCGCGKKYYKARRKWRTG 12300
Bhutan-09027        GAASCYYGACGGYYGGYRSWCWCCKYWTYYWMTYTASKRTAYMCGCGKKYYKARRKWRTG 12300
Bhutan-09030        GAASCYYGACGGYYGGYRSWCWCCKYWTYYWMTYTASKRTAYMCGCGKKYYKARRKWRTG 12300
Bhutan-09005        KRRYYTYKRAASTCRKTASWYATYTCAACCTCYYKRGKAARTAARSRGKYTTRRAGWGWC 12300
Indonesia-88035     KRRYYTYKRAASYCGKTASWYATCTCATCTTCTYKRGKRWRTMAGCRGKTTTARAGWGWC 12300
```

```
Indonesia-88045      KRRYYTYKRAASYCGKTASWYATCTCATCTTCTYKRGKRWRTMAGCRGKTTTARAGWGWC 12300
Indonesia-88065      KRRYYTYKRAASTCRKTASWYATYTCAACCTCYYKRGKAARTAARSRGKYTTRRAGWGWC 12300
                           *  ...      **    .  :     *. .*     . .  *  . *  *

Bhutan-09015         MRRRYCGTCTAGACCGGYYRYCRGMRAGKATCMKARMSAWRWSTYYMGSRYCMMYYARWC 12360
Bhutan-09024         MRRRYCGTCTAGACCGGYYRYCRGMRAGKATCMKARMSAWRWSTYYMGSRYCMMYYARWC 12360
Bhutan-09027         MRRRYCGTCTAGACCGGYYRYCRGMRAGKATCMKARMSAWRWSTYYMGSRYCMMYYARWC 12360
Bhutan-09030         MRRRYCGTCTAGACCGGYYRYCRGMRAGKATCMKARMSAWRWSTYYMGSRYCMMYYARWC 12360
Bhutan-09005         AGGGCYTYAYGRMYYATYCATYATCRWKKWWSAKRACSCWATCYYYARCGTYMMTYTAWY 12360
Indonesia-88035      AGGGCYTYAYARMYYATYCATYATCRWKKWWSAKRACSCWATCYYYARCGTTMCTYTAWY 12360
Indonesia-88045      AGGGCYTYAYARMYYATYCATYATCRWKKWWSAKRACSCWATCYYYARCGTTMCTYTAWY 12360
Indonesia-88065      AGGGCYTYAYGRMYYATYCATYATCRWKKWWSAKRACSCWATCYYYARCGTYMMTYTAWY 12360
                             . .    . *       *  *  . *   *.*  . **  .   *  *: *

Bhutan-09015         RMRGYSSTARYCYTAAWCKGTYWGTYRMRKAWAMYGRRSYMTGAMRGMRKYYWTRKMCTR 12420
Bhutan-09024         RMRGYSSTARYCYTAAWCKGTYWGTYRMRKAWAMYGRRSYMTGAMRGMRKYYWTRKMCTR 12420
Bhutan-09027         RMRGYSSTARYCYTAAWCKGTYWGTYRMRKAWAMYGRRSYMTGAMRGMRKYYWTRKMCTR 12420
Bhutan-09030         RMRGYSSTARYCYTAAWCKGTYWGTYRMRKAWAMYGRRSYMTGAMRGMRKYYWTRKMCTR 12420
Bhutan-09005         ACARTCGYGATGYCMATSKTACWRYYACATGATCYRRRCYATRTMRAARGCYWTATMYWG 12420
Indonesia-88035      ACRRTCSYGATGYCMRTSKTACWRYYACATGATCYGRRCYAYRTMAAARGCTTWATMYWG 12420
Indonesia-88045      ACRRTCSYGATGYCMRTSKTACWRYYACATGATCYGRRCYAYRTMAAARGCYTWATMYWG 12420
Indonesia-88065      ACARTCGYGATGYCMATSKTACWRYYACATGATCYRRRCYATRTMRAARGCYWTATMYWG 12420
                          .. .   *    .* : *  *   .. : * **.*   :* . *      .*

Bhutan-09015         YARSTTGATCAAWAKRGAYYGSTAYARRTTSYRTAKWWKMTWGCAAGKTTKRRCKYAKRG 12480
Bhutan-09024         YARSTTGATCAATAKRGAYTGSTAYARRTTSYRTAKWWKMTWGCAAGKTTKRRCKYAKRG 12480
Bhutan-09027         YARSTTGATCAAWAKRGAYYGSTAYARRTTSYRTAKWWKMTWGCAAGKTTKRRCKYAKRG 12480
Bhutan-09030         YARSTTGATCAAWAKRGAYYGSTAYARRTTSYRTAKWWKMTWGCAAGKTTKRRCKYAKRG 12480
Bhutan-09005         YTRCCKRTAARRTGTGAGYTACWAYWRRTCSTRWRTTTGMYWRYRRGKYTTAATKCAKGG 12480
Indonesia-88035      YTGCCKRTACRRTGTGAGCTACTRYWRRWCSTRWRTTTGMYWRYGGTKYATAATKCRKGK 12480
Indonesia-88045      YTGCCKRTACRRTGTGAGCTACTRYWRRWCSTRWRTTTGMYWRYGGTKYATAATKCRKGG 12480
Indonesia-88065      YTRCCKRTAARRTGTGAGYTACWAYWRRTCSTRWRTTTGMYWRYRRGKYTTAATKCAKGG 12480
                     *: . . ::.   .. ..  ..  * **  * *  .   * *     * :.   *  *

Bhutan-09015         KGTGMSTRWTWTRTRGSTTWYWWYRTYSYRKRRKCSAYGYGGACTWAWWTYGRGTYYWRT 12540
Bhutan-09024         KGTGMSTRWTWTRTRGSTTWYWWYRTYSYRKRRKCSAYGYGGACTWAWWTYGRGTYYWRT 12540
Bhutan-09027         KGTGMSTRWTWTRTRGSTTWYWWYRTYSYRKRRKCSAYGYGGACTWAWWTYGRGTYYWRT 12540
Bhutan-09030         KGTGMSTRWTWTRTRGSTTWYWWYRTYSYRKRRKCSAYGYGGACTWAWWTYGRGTYYWRT 12540
Bhutan-09005         TKKRMCYGTYAYGYGRGCWWTWTTACYGCGKRRKSCGCRCARRMYWRTTKCKARYCTAAW 12540
Indonesia-88035      TKKRMCYGTYWYGYGRGCWWTWWTATYGCGKRRKSCACGTARRMYWRTTKCKARYCTAAT 12540
Indonesia-88045      TKKRMCYGTYWYGYGRGCWWTWWTATYGCGKRRKSCACGTARRMYWRTTKCKARYCTAAT 12540
Indonesia-88065      TKKRMCYGTYAYGYGRGCWWTWTTACYGCGKRRKSCGCRCARRMYWRTTKCKARYCTAAW 12540
                     . . *.          .  * *    *.  ****...   .    *   .

Bhutan-09015         AWMYMASWTRMTTWCTKTKSMCRYYTTTYYKRAWYGRGGGCTARWYYYRCAMRGKYTYMR 12600
Bhutan-09024         AWMYMASWTRMTTWCTKTKSMCRYYTTTYYKRAWYGRGGGCTARWYYYRCAMRGKYTYMR 12600
Bhutan-09027         AWMYMASWTRMTTWCTKTKSMCRYYTTTYYKRAWYGRGGGCTARWYYYRCAMRGKYTYMR 12600
Bhutan-09030         AWMYMASWTRMTTWCTKTKSMCRYYTTTYYKRAWYGRGGGCTARWYYYRCAMRGKYTYMR 12600
Bhutan-09005         RACYMMSWYRMKCTYATWGCAYGYYYWWCYKRRWCAAGGAYWRRWTYYRYWCARKCGAAA 12600
Indonesia-88035      AACYMMSWYRMKYTYWTAGCAYGYYYWWCYKRRWCAARRAYWRRWTYYRYWCARKCKWMA 12600
Indonesia-88045      AACYMMSWYRMKYTYWTAGCAYGYYYWWCYKRRWCAAGRAYWRRWTYYRYWCARKCKWMA 12600
Indonesia-88065      RACYMMSWYRMKYTYATWGCAYGYYYWWCYKRRWCAAGGAYWRRWTYYRYWCARKCGAAA 12600
                        ** ** **.    .  .   **    *** * .   .   ** ***     *
```

```
Bhutan-09015         CCRCYCGRMYSCTRARYCYYTRARATWKWRCTCATRYAAAGRGRTTKYACRYMCAYSMCY 12660
Bhutan-09024         CCRCYCGRMYSCTRARYCYCTRARATWKWRCTCATRYAAAGARRTTKYACRYMCAYSMCY 12660
Bhutan-09027         CCRCYCGRMYSCTRARYCYYTRARATWKWRCTCATRYAAAGARRTTKYACRYMCAYSMCY 12660
Bhutan-09030         CCRCYCGRMYSCTRARYCYYTRARATWKWRCTCATRYAAAGRGRTTKYACAYMCAYSMCY 12660
Bhutan-09005         TGATTSSGATGYWAAGCYCTKGRRRYATAGYWMRYGTWMTRAGGYKGTWYAYCYRTSAMC 12660
Indonesia-88035      YGAYTSSGATGYWAAGCYCTKGRRRTATAGYWMRYGTWMTRAGGYKGTWYAYCYRTSAMC 12660
Indonesia-88045      YGAYTSSGATGYWARRCYCTKGRRRTATAGYWMRTGTWMTGARGYKGTWYAYCYRTSAMC 12660
Indonesia-88065      TGATTSSGATGYWAAGCYCTKGRRRYATAGYWMRYGTWMTRAGGYKGTWYAYCYRTSAMC 12660
                          ..   .         .  *   .           :     .     *    *

Bhutan-09015         ASYMWTCCRRGSYCMRWYCTRGWYRRSAGTTSTTACYRTTRKRRYCYSYWYYGTRSGYAM 12720
Bhutan-09024         ASYMWTCCRRGSYCMRWYCTRGWYRRSAGTTSTTACYRTTRKRRYCYSYWYYGTRSGYAM 12720
Bhutan-09027         ASYMWTCCRRGSYCMRWYCTRGWYRRSAGTTSTTACYRTTRKRRYCYSYWYYGTRSGYAM 12720
Bhutan-09030         ASYMWTCCRRGSYCMRWYCTRGWYRRSAGTTSTTACYRTTRKRRYCYSYWYYGTRSGYAM 12720
Bhutan-09005         WGYMTWYCGRSCCSCGTYYYRGTTARSAAGCCCGTGCAYCRGGGYYYSYWYTRKRSRCRC 12720
Indonesia-88035      WGYMTWYAGRSCCSCGTYYYRSTCGRSGGGCCCGTGCAYCRGGGYYYSYWYTRKRSRCRC 12720
Indonesia-88045      WGYMTWYAGRSCCSCGTYYYRSTCGRSGGGCCCGTGCAYCRGGGYYYSYWYTGTRSRCRC 12720
Indonesia-88065      WGYMTWYCGRSCCSCGTYYYRGTTARSAAGCCCGTGCAYCRGGGYYYSYWYTGTRSRCRC 12720
                      .**   . *.. .   *  *.   **..  .  :     *   * *****  .**

Bhutan-09015         TGCKTYGYCYGTCMTACGRGYCTYTAMRYRRSSYTACYTCCRAYYATGRYRMRAWWTYCG 12780
Bhutan-09024         TGCKTYGYCYGTCMTACGRGYCTYTAMRYRRSSYTACYTCCRAYYATGRYRMRAWWTYCG 12780
Bhutan-09027         TGCKTYGYCYGTCMTACGRGYCTYTAMRYRRSSYTACYTCCRAYYATGAYRAAAWWTYCG 12780
Bhutan-09030         TGCKTYGYCYGTCCTACGRGYCTYTAMRYRRSSYTACYTCCRAYYATGRYRMAAWWTYCG 12780
Bhutan-09005         YKMGYYGCYCRYMMWGYSGSTYYCYRARYRGCSTAMYTWTSRGCTRYSGCAMARATYCSR 12780
Indonesia-88035      YKMGYYGCYCGYMCWGCSGSTYYCYRARYRGCSTAMYTWTSRGCTRYSGCAMRRATYCSR 12780
Indonesia-88045      YKMGYYRCTCRYMMWGCSGSTYYCYRARYRGCSTAMYTWTSRGCTRYSGCAMRRATYCSR 12780
Indonesia-88065      YKMGYYGCYCRYMCWGYSGSTYYCYRARYRGCSTAMYTWTSRGCTRYSGCAMRRATYCSR 12780
                          *         . . .       *** .* :     .*.    .          .

Bhutan-09015         TAGGCATACAAGWARSARGKAWTRYYGWRKYMYRRACCRRYGAGATYYKSYRSGCGMRKA 12840
Bhutan-09024         TAGGCATACAAGWARSARGKAWTRYYGWRKYMYRRACCRRYGAGATYYKSYRSGCGMRKA 12840
Bhutan-09027         TAGGCATACAAGWARSARGKAWTRYYGWRKYMYRRACCRRYGAGATYYKSYRSGCGMRKA 12840
Bhutan-09030         TAGGCATACAAGWARSARGKAWTRYYGWRKYMYRWACYRRYGAGATYYKSYRSGCGMRKA 12840
Bhutan-09005         KWKKYRWCYRWGWGRGRGATMAKRTCKAAGTCTRGRYCRRYARRRYTYTCTACATRWRKG 12840
Indonesia-88035      KWKKYRWCYRWTWRRGRGATMAKRTCKAAGTCTRGRYCRRYARRRYTYTCTACATRWRKG 12840
Indonesia-88045      KWKKYRWCYRWTWRRGRGATMAKRTCKAAGTCTRGRYCRRYARRRYTYTCTACATRWRKG 12840
Indonesia-88065      KWKKYRWCYRWGWGRGRGATMAKRTCKAAGTCTRGRYCRRYARRRYTYTCTACATRWRKG 12840
                     .      .    * *.  . . .*         *    ***.     *..  ..   **.

Bhutan-09015         WYYTGGCYYKGTRWGKMKRYCRMTKKMYMRRCGYCGCRTYTCTWATRKYGWYTTTCCMRR 12900
Bhutan-09024         WYYTGGCYYKGTRWGKMKRYCRMTKKMYMRRCGYCGCRTYTYTWATRKYGWYTTTCCMRR 12900
Bhutan-09027         WYYTGGCYYKGTRWGKMKRYCRMTKKMYMAACGYCGCRTYTCTAATRKYGWYTTTCCMRR 12900
Bhutan-09030         WYYTGGCYYKGTRWGKMKRYCRMTKKMYMAACGYCGCRTYTCTWATRKTGWYTTTCCMRR 12900
Bhutan-09005         TCTYRRYCYKRYRARKMGACYGMWTGMTMGATACTRYRTTYCYAGARKTKTCACKSMMGR 12900
Indonesia-88035      TCTYRRYCYKRYRARKMGACYGMWTGMTMGRTACTRYRATYCYAGARKTKTCATKSMMRR 12900
Indonesia-88045      TCTYRRYCYKRYRARKMGACYGMWTGMTMGRTACTRYRATYCYAGARKTKTCATKSMMRR 12900
Indonesia-88065      TCTYRRYCYKRYRARKMGACYGMWTGMTMGRTACTRYRTTYCYAGARKTKTCACKSCMGR 12900
                             **  *  **     * . * *   .    *:     .:**    : .. * *

Bhutan-09015         CAKYGCRRACRGTWCMGTWGYTCYMRYCKGKGMYYGCKSWATGYRRMMTWAATRWCGGRS 12960
Bhutan-09024         CAKYGCRRACRGTWCMGTWSCTCYMRYCKGKGMYYGCKSWATGYRRMMTWAATRWCGGRS 12960
Bhutan-09027         CAKYGCRRACRGTWCMGTWGYTCYMRYYKGKGMYYGCKSWATGYRRMMTWAATAWCGGRS 12960
Bhutan-09030         CAKYGCRRACRGTWCMGTWSYTCYMRYCKGKGMYYGCKSWATGYRRMMTWAATRWCGGGC 12960
```

```
Bhutan-09005         SMGTKMARRMRKYTYCTYAGCGTTMRTCTRTRCYYRYTGATYRCGACAYTRMYRWSKARS 12960
Indonesia-88035      SMGTKMARRMRGTTYCTYAGCGTTMRTCTRTRCYYRYTGATYRCGACAYTRMYRWSKARS 12960
Indonesia-88045      SMGTKMARRMRKYTYCTYAGCGTTMRTCTRTRCYYRYTGATYRCGACAYTRMYRWSKARS 12960
Indonesia-88065      SMGTKMARRMRKYTYCTYAGCGTTMRTCTRTRCYYRYTGATYRCGACAYTRMYRWSKARS 12960
                     .      *  *        .    **  . .  **  .. :             *. . .

Bhutan-09015         ARWRRYAYYRCKTMKKGTYGTTAACGTYYYATWYAMTGACKTRCYGAYCWWYMRCTRWYC 13020
Bhutan-09024         ARWRRYAYYRCKTMKKGTYGTTAACGTYYYATWYAMTGACKTRCYGAYCWWYMRCTRWYC 13020
Bhutan-09027         ARWRRYAYYRCKTMKKGTYGTTAACGTYYYATWYAMTGACKTRCYGAYCWAYMRCTRWYC 13020
Bhutan-09030         ARWRRYAYYRCKTMKKGTYGTTAACGTYYYATWYAMTGACKTRCYGAYCWWYMRCTRWYC 13020
Bhutan-09005         RGAGRTMCCATGYCKGRWTRYCWTYRCTCYGCTCMCYRRYGYGMTAGYYAWCAGYYAWTY 13020
Indonesia-88035      RGAGRTMCCATGYCKGRTYRTTWTYRCTCYGCTCMCYRRYGYGMTAGYYAWCAGYYAWTY 13020
Indonesia-88045      RGAGRTMCCATGYCKGRTYRTTWTYRCTCYGCTCMCYRRYGYGMTAGYYAWCAGYYAWTY 13020
Indonesia-88065      RGAGRTMCCATGYCKGRWTRYCWTYRCTCYGCTCMCYRRYGYGMTAGYYAWCAGYYAWTY 13020
                         *         *        :     *.              ..*         *

Bhutan-09015         GCKWTACGTCKCRTCRSYWCSTYKCGAMKTCGRCGGTRAWRYYKTYGTYYRRKGGYRYTY 13080
Bhutan-09024         GCKWTACGTCKCRTCRSYWCSTYKCGAMKTCGRCGGTRAWRYYKTYGTYYRRKGGYGYTY 13080
Bhutan-09027         GCKWTACGTCKCRTCGSYWCSTYKCGAMKTCGRCGGTRAWRYYKTYGTYYRRKGGYRYTY 13080
Bhutan-09030         GCKWTACGTCKCRTCRSYWCSTYKCGAMKTCGRCGGTRAWRYYKTYGTYYRRKGGYRYTY 13080
Bhutan-09005         SYKWCASKWCGYRYTGSYAYGYYTSTGMKCMSGYASWGRTRYYGWCRYTTARGRAYGYCT 13080
Indonesia-88035      SYGATCSKWCGYRYYGSYAYGYYTSTGMGCASGYRSWGRTRYYGACRYTTARGRAYGYCT 13080
Indonesia-88045      SYGATCSKWCGYRYYGSYAYGYYTSTGMGCASGYRSWGRTRYYGACRYTTARGRAYGYCT 13080
Indonesia-88065      SYKWCASKWSGYRYTGSYAYGYYTSTGMKCMSGYASWGRTRYYGWCRYTTARGRAYGYCT 13080
                     .    ..  .  *   **  . *.. .*   .   .    ***        *  .* *

Bhutan-09015         RGTKTGRARRYTAYWYCAYACRYTCRCCACWRRRCGTAAAAMRRWRCYKTGWMYATCAGM 13140
Bhutan-09024         AGTKTGRARRYTAYWYCRYACRYTCACCACWRRRCGTAAAAMRRWRCYKTGWMYATCAGM 13140
Bhutan-09027         GGTKTGRARRYTAYWYCAYACRYTCRCCACWRRRCGTAAAAMRRWRCYKTGWMYATCAGM 13140
Bhutan-09030         RGTKTGRARRYTAYWYCAYACRYTCRCCACWRRRCGTAAAAMRRWRCYKTGWMYATCAGM 13140
Bhutan-09005         GAYTWSARRRYWWYWCSAYGSACYYASAWMMAGGSGYGCTRAARTRMTTWGTCTRYTGAC 13140
Indonesia-88035      GAYTWSARRRYWWYWCSAYGSACYYASAWAMAGGSSYGCTRAARTRMTTWTTCTRYTGAC 13140
Indonesia-88045      GAYTWSARRRYWWYWCSAYGSACYYASAWAMAGGSSTGCTRAARTRMTTWTTCTRYTGAC 13140
Indonesia-88065      GAYTWSARRRYWWYWCSAYGSACYYASAWMMAGGSGTGCTRAARTRMTTWGTCTRYTGAC 13140
                      . . .  ***  ** . *..     ..      .. ..:   * *  .        ..

Bhutan-09015         RCYAGARRCRCTGGRMKCTCCACAASGACYWRYTWMRSRGKWYYRYRCYTKRTYRYMCTG 13200
Bhutan-09024         RCYAGARRCRCTGGRMKCTCCACAASGACYWRYTWMRSRGKWYYRYRCYTKRTYRYMCTG 13200
Bhutan-09027         RCYAGARRCRCTGGRMKCTCCACAASGACYWRYTWMRSRGKWYYRYRCYTKRTYRYMCTG 13200
Bhutan-09030         RCYAGARRCRCTGGRMKCTCCACAASGACYWRYTWMRSRGKWYYRYRCYTKRTYRYMCTG 13200
Bhutan-09005         AYCMRTGRYASWKKGCKCWMYRYMRCRRYYWRCCRMRSGSYWYTRTASYKKAYTRTMMTS 13200
Indonesia-88035      RYYAGAGRYASWKKGCKAWMYRYMRCRRYYWRCCRMRSGSYWYTRYRSYKKAYTRTMMYS 13200
Indonesia-88045      RYYAGAGRYASWKKGCKAWMYRYMRCGRYYWRCCRMRSGSYWYTRYRSYKKAYTRTMMYS 13200
Indonesia-88065      AYCMRTGRYASWKKGCKCWMYRYMRCRRYYWRCCRMRSGSYWYTRYRSYKKAYTRTMMTS 13200
                          : *  .     *.       .   ***   *** . ** *  .*.*   * *  .

Bhutan-09015         ATYKMRCCMTATCAWTARTYRYYRMGCTGYWWYMAARTWRMACGMKMAASTTCMRGWCGY 13260
Bhutan-09024         ATYKMRCCMTATCAWTARTYRYYRMGCTGYWWYMAARTWRMACGMKMAASTTCMRGWCGY 13260
Bhutan-09027         ATYKMRCCMTATCAWTARTTRYYRMGCTGYWWYMAARTWRMACGMKMAASTTCMRGWCGY 13260
Bhutan-09030         ATYKMRCCMTATCAWTARTYRYYRMGCTGYWWYMAARTWRMACGMKMAASTTCMRGWCGY 13260
Bhutan-09005         RKCGMRYYCYRTTMWTRRKTGYCAARSCATWTCAWMRCWRMMYRCRARWSCWMMACTYRY 13260
Indonesia-88035      RKCGMRYYCCRYTMWTRRKTGCCAARSCATWTCAWMRCWRMMYRCGARWCCAMMACTCGY 13260
Indonesia-88045      RKCGMRYYCCRYTMWKRRKTGCCAARSCATWTCAWMRCWRMMYRCGARWCCAMMACTCGY 13260
Indonesia-88065      RKCGMRYYCYRYTMWKRRKTGYCAARSCATWTCAWMRCWRMMYRCRARWSCWMMACTYRY 13260
```

```
                         .  **        *. *.       . . *     * ***        .   *     *

Bhutan-09015            RAYMYYAASYCTYRRRRAYYYCGCYRTACRTCYWCGGGGGGYTMMRSGYRYKRRYWCWGA 13320
Bhutan-09024            RAYMYYAASYCTYRRRRAYYYCGCYRTACRTCYWCGGGGGGYTMMRSSYRYKRRYWCWGA 13320
Bhutan-09027            RAYMYYAASYCTYRRRRAYYYCGCYRTACRTCYWCGGGGGGYTMMRSSYRYKRRYWCWGA 13320
Bhutan-09030            RAYMYYAASYCTYRRRRAYYYCGCYRTACRTCYWCGGGGGGYTMMRSGYRYKRRYWCWGA 13320
Bhutan-09005            ATTMYTWWGTMWYRAGARCCCTAGCACMYGCTCTMRRRRRKYKAMRSSYAYTGGTWYTRG 13320
Indonesia-88035         ATTMYTWWGTMWYRAGARCCCTAGCACMYGCTCTMRRRRRKYKAMRSGYAYTGGTWYTRG 13320
Indonesia-88045         ATTMYTWWGTMWYRAGARCCCTAGCACMYGCTCTMRRRRRKYKAMRSSYAYTGGTWYTRG 13320
Indonesia-88065         ATTMYTWWGTMWYRAGARCCCTAGCACMYGCTCTMRRRRRKYKAMRSSYAYTGGTWYTRG 13320
                         : **   .   **        .                  *. ***.* *.   *   .

Bhutan-09015            CTGKWRGRYYYGRCRYWRAGTCSYKSGSKGSWRGMKTTASCCMAWTWKGYGATWGCAAYR 13380
Bhutan-09024            CTGKWRGRYYYGRCRYWRAGTCSYKSGSKGSWRGMKTTASCCMAWTWKGYGATWGCAAYR 13380
Bhutan-09027            CTGKWRGRYYYGRCRYWRAGTCSYKSGSKGSWRGMKTTASCCMAWTWKGYGATWGCAAYR 13380
Bhutan-09030            CTGKWRGRYYYGRCRYWRAGTCSYKSGSKGSWRGMKTTASCCMAWTWKGYGATWGCAAYR 13380
Bhutan-09005            MCATARKRCYYAAYGYWRRRYYCCTCASGRGAATCKCWGGTYAMWGATRTRWWWRTMRYG 13380
Indonesia-88035         MCATAAKRCYYAACGYWRGAYYCCTCASGRGAATCKCWGGTYAMWGATRTRATTRTMAYG 13380
Indonesia-88045         MCATARKRCYYAACGYWRRRYYCCTCASGRGAATCKCWGGTYAMWGATRTRATTRTMAYG 13380
Indonesia-88065         MCATARKRCYYAAYGYWRRRYYCCTCASGRGAATCKCWGGTYAMWGATRTRWWWRTMRYG 13380
                          ..   * **.   ***    . ...*  .    *  ..    *  .          *

Bhutan-09015            AMRMWYGKRRSYMSSTTRYASKKTCYYYATGKYYWAASMYGYMSRGYRMKTAMRYATMYG 13440
Bhutan-09024            AMRMWYGKRRSYMSSTTRYASKKTCYYYATGKYYWAASMYGYMSRGYRMKTAMRYATMYG 13440
Bhutan-09027            AMRMWYGKRRSYMSSTTRYASKKTCYYYATGKYYWAASMYGYMSRGYRMKTAMRYATMYG 13440
Bhutan-09030            AMRMWYGKRRSYMSSTTRYASKKTCYYYATGKYYWAASMYGYMSRGYRMKTAMRYATMYG 13440
Bhutan-09005            RMGAWCRGAASTMGGAYRTGGTGGMCTYGYRGTTTMMGMYSCCCGRTRMTGGCATWYCYC 13440
Indonesia-88035         RMGAWCRGAASTMGGACRTGGTGGMCTTGTGGTTTAMGATCCCCGRTRMTGGCATTCCYC 13440
Indonesia-88045         RMGAWCRGAASTMGGACRTGGTGGMCTTGTGGTTTAMGATCCCCGRTRMTGGCATTCCYC 13440
Indonesia-88065         RMGAWCRGAASTMGGAYRTGGTGGMCTTGYRGTTTAAGMYSCCCGRTRMTGGCATWYCYC 13440
                         *  *     * *..: * ...      .        .     .   **. .      *

Bhutan-09015            RTGCKWRWTRCWRMKSRYRKTTGRRAGYKAAYTWYKYWGMMTRRWGACSRTKCTTSCWYW 13500
Bhutan-09024            RTGCKWRWTRCWRMKSRYRKTTGRRAGYKAAYTWYKYWGMMTRRWGAYSRWKCTTSCWYW 13500
Bhutan-09027            RTGCKWRWTRCWRMKSRYRKTTGRRAGYKAAYTWYKYWGMMTRRWGACSRTKCTTSCWYW 13500
Bhutan-09030            RTGCKWRWTRCWRMKSRYRKTTGRRAGYKAAYTWYKYWGMMTRRWGACSRWKCTTSCWYA 13500
Bhutan-09005            RCSYTWGTCRYWAATCGCRKKWCGGRRYGRGYWWCGCAAMMKAAWRMYSGWKYKKGYTTW 13500
Indonesia-88035         RCSYTWGTCRYWAATCGCRKTTCGGRRYGRGYWWCGCAAMMKAAWGACCGWGCTTGYTTW 13500
Indonesia-88045         RCSYTWGTCRYWAATCGCRKKWCGGRRCGGGYWWCGCAAMMKAAWGACCGWKYKTGYTTW 13500
Indonesia-88065         RCSYTWGTCRYWAATCGCRKKWCGGRRYGRGYWWCGCAAMMKAAWRMCSGWKYKKGYTTA 13500
                        * . .*   * *  ..  **.         .* *    .**.  *   .    ...

Bhutan-09015            CRYYRMRWRRRKKTGYTRTCYWTGYYYRCAKATGCGRCAASRMCAYWRSKRATARAWYWY 13560
Bhutan-09024            CRYYRMRWRRRKKTGYTRTCYWTGYYYRCAKATGCGRCAASRMCAYWRSKRATARAWYWY 13560
Bhutan-09027            CRYYRMRWRRRKKTGYTRTCYWTGYYYRCAKATGCGRCAASRMCAYWRSKRATARAWYWY 13560
Bhutan-09030            CRYYRMRWRRRKKTGYTRTCYWTGYYYRCAKATGCGRYAASRMCAYWRSKRATARAWYWY 13560
Bhutan-09005            YAYYAAGTGRAGKYACWRWYYWYRYYTGYWGRYRYRACWRCRMYRCTASKRGCGRMAYWM 13560
Indonesia-88035         YAYYAAGTGGAGKYAYWRWCCWTRYYTGYWGRYRYGACAACAMYRCTASKRGCGRMAYWM 13560
Indonesia-88045         YAYYAAGTGGAGKYAYARWCCWTRYYTGCWGRYRYGACWACAMYRCTASKRGCGRMAYWM 13560
Indonesia-88065         YAYYAAGTGRAGKYACWRWYYWYGYYTGYWGRYRYRACWRCRMYRCTASKRGCGRMAYWM 13560
                          **        * .  *   *  **              . *     ***. .*  **

Bhutan-09015            AWCRWWCTTATTTYTKYTTYRRSCYYYCWMYGYYRRRRRKGYMCWWWACRRWRRRRTGCM 13620
Bhutan-09024            AWCRWWCTTATTTYTKYTTYRRSCYYYCWMYGYYRRRRRKGYMCWWWACRRWRRRATGCM 13620
```

```
Bhutan-09027        AWCRWWCTTATTTYTKYTTYRRSCYYYCWMYGYYRRRRRKGYMCWWWACRRWRRRATGCM 13620
Bhutan-09030        AWCRWWCTTATTTYTKYTTYRRSCYYYCWMYGYYRRRRRKGYMCWWWACRRWRRRRTGCM 13620
Bhutan-09005        MWSGTAYYTAGWWTYTYTAYAASYYYYYWMTRYCMRGAAGKYCYTTWTTRRWAGRAYSTC 13620
Indonesia-88035     MWSGTAYTYGGTTTYTYYAYAASYYYYYWMTRYCMRGAAGKYCYTTWTTRRWAGRATGTC 13620
Indonesia-88045     MWSGTAYTYGGTTTYTYYACAASYYYYYWMTRYCMRGAAGKYCYTTWTTRRWAGRATGTC 13620
Indonesia-88065     MWSGTAYYTAGWWTYTYTAYAACCTYYYWMTRYCMRGAAGKYCYTTWTTRRWAGRAYSTC 13620
                     *.      .     .* :   .  ** **  *  *     *    *: ***  *  .

Bhutan-09015        RRACMYAWWRAWMRYRWKGCRYATARKCKMKYGYRKGGSWRAYAWASTTTTWTCWGCATS 13680
Bhutan-09024        RRACMYAWWRAWMRYRWKGCRYATARKCKMKYGYRKGGSWRAYAWASTTTTWTCWGCATS 13680
Bhutan-09027        GRACMYAWWRAWMRYRWKGCRYATARKCKMKYGYRKGGSWRAYAWASTTTTWTCWGCATS 13680
Bhutan-09030        RRACMYAWWRAWMRYRWKGCRYATARKCKMKYGYRKGGSWRAYAWASTTTTWTCWGCATS 13680
Bhutan-09005        RRMYATRTTGRACACRWKATRTWYMGTTGAKCRCAGRRGAATYWWRGYWWWWCCWAMCCG 13680
Indonesia-88035     RAACATATTGRWCAYRWKATRTWYMGTTGAKCRYRGRRGAATCAAASTTTWACYWAACCG 13680
Indonesia-88045     RAACATATTGRWCAYRWKATRTWYMGTTGAKCRYRGRRGAATCAAASTTTWACYAAACCG 13680
Indonesia-88065     RRMYATRTTGRACACRWKATRTWYMGTTGAKCRYRGRRGAATYWWRGYWWWWCYWAMCCG 13680
                                   ***. *     .   *       .  :    .        . . .

Bhutan-09015        YWYCYRRRARGYKGASRWGRYRYGRCRAYCYMWACRGTYAYRSRYRAYYTKRGKYCRAYR 13740
Bhutan-09024        YWYCYRRRARGYKGASRWGRYRYGRCRAYCYMWACRGTYAYRSRYRAYYTKRGKYCRAYR 13740
Bhutan-09027        YWYCYRRRARGYKGASRWGRYRYGRCRAYCYMWACRGTYAYRSRYRAYYTKRGKYCRAYR 13740
Bhutan-09030        YWYCYRRRARGYKGASRWGRYRYGRCRAYCYMWACRGTYAYRSRYRAYYTKRGKYCRAYR 13740
Bhutan-09005        CWYSYAAAGGRYKRWGGTKRTGCRRYAGCMCATGYRKYYGCACRCRRYYKTASGTYGGCA 13740
Indonesia-88035     CWYSYAAAGGGTKRWGGTKRTGCRRYAGCMCATGYRKYYGCACRCRRYYKTASGTYGGCA 13740
Indonesia-88045     CWYSYAAAGGGTKATGGTKRTGCRRYAGCMCATGYRKYYGCACRCRRYYKTASGTYGGCA 13740
Indonesia-88065     CWYSYAAAGGRYKRWGGTKRTGCRRYAGCMCATGYRKYYGCACRCRRYYKTASGTYGGCA 13740
                     **.*   .   *  .   *    *  .     . *  *.  .* * **.. .    .

Bhutan-09015        KWWWAYSRACYCARAYRTRGYRAYYRMARYRYKCYAAYWSRYRCGSAWKYGMRYRGRGYS 13800
Bhutan-09024        KWWWAYSRACYCARAYRTRGYRAYYRMARYRYKCYAAYWSRYRCGSAWKYGMRYRGRGYS 13800
Bhutan-09027        KWWWAYSRACYCARAYRTRGYRAYYRMARYRYKCYAAYWSRYRCGSAWKYGMRYRGRGYS 13800
Bhutan-09030        KWWWAYSRACYCARAYRTRGYRAYYRMARYRYKCYAAYWSRYRCGSAWKYGMRYRGRGYS 13800
Bhutan-09005        KAAWWYGRRSCYRGMYAYRKCRRCTGWMRCACGCCMGCASGTAYTGWATCRCRYGRGAYC 13800
Indonesia-88035     KAAWWYGRAGCYRGMYAYRKCGACTGWMRCACGYYMGCASGTATTGWATCGCRYGRGAYC 13800
Indonesia-88045     KAAWWYGRAGCYRGMYAYRKCGACTGWMRCACGYYMGCASGTAYTGWATCGCRYGRGAYC 13800
Indonesia-88065     KAAWWYGRRSCYRGMYAYRKCRRCTGWMRCACGYYMGCASGTAYTGWATCRCRYGRGAYC 13800
                    *  * *.*       *  *         *       .  *     .  .   **   .*.

Bhutan-09015        KYTYYYRYRWSYSYCTRRKCRCTCRYKAGARRWKRYAGCWATSAYRMAATGWGRRRACYG 13860
Bhutan-09024        KYTYYYRYRWSCSYCTRRKCRCTCRYKAGARRWKRYAGCWATSAYRMAATGWGRRRACYG 13860
Bhutan-09027        KYTYYYRYRWSYSYCTRRKCRCTCRYKAGARAATRYAGCWAYSAYRMAATGWGRRRACYG 13860
Bhutan-09030        KYTYYYRYRWSYSYCTRRKCRCTCRYKAGARRWTRYAGCWAYSAYRMAATGWGRRRACYG 13860
Bhutan-09005        TCTCYYRCAACTGCYCRATYRYGTRCGAKWAGTGRYWRSWRCCWCRMRMYTASARGWTYR 13860
Indonesia-88035     TYYCYYRCAACTGCYCRATYRYGTRCKRKWAGTGRYWRSWRCCWCRMRMYGASARGATYR 13860
Indonesia-88045     TYYCYYRCAACTGCYCRATYRYGTRCKRKWAGTGRYWRSWRCCWCRMRMYGASARGATYR 13860
Indonesia-88065     TYYCYYRCAACTGCYCGATYRYGTRCKRKWAGTGRYWRSWRCCWCRMRMYKASARGWTYR 13860
                    .   ***   . .     . *   *         **  .*  .  **     . *   *

Bhutan-09015        CKCTAKYTCSYKMTCRCSTKAYGRWGAWYYYCAAYGRCAAGATSRWYKYTKTMKARRRCY 13920
Bhutan-09024        CKCTAKYTCSYKMTCRCSTKAYGRWGAWYYYCAAYGRCAAGATSRWYKYTKTMKARRRCY 13920
Bhutan-09027        CKCTAKYTCSYKMTCRCSTKAYGRWGAWYYYCAAYGRMAAGATSRWYKYTKTMKARRRCY 13920
Bhutan-09030        CKCTAKYTCSYKMTCRCSTKAYGRWGAWYYYCAAYGRMAAGATSRWYKYTKTMKARRRCY 13920
Bhutan-09005        YKACGGCAYSYGAYARSCCKWYRGASRWYYCAWMYRACMCARYGAWYKYYGWATWAAAYT 13920
Indonesia-88035     YKCCGKCWCCCGATARSCCKWYRGASRWYYCAWMYRACMCAATGAWYKYYGWATWAAAYT 13920
```

```
Indonesia-88045     YKCCGKCWCCCGATARSCCKWYRGASRWYYCAWMYRACMCAATGAWYKYYGWATWAAAYT 13920
Indonesia-88065     YKACGKCWYSYGAYARSCCKWYRGASRWYYCAWMYRACMCARYGAWYKYYGWATWAAAYT 13920
                     *. .    .    .*.. * *   . *** .  *    ..  . ****    .

Bhutan-09015        MWACGTCRRRASRGCGTGGTATCGYRTKYTARYTARSRGSGTMWCTTACARRRCGGTAYK 13980
Bhutan-09024        MWACGTCRRRASRGCGTGGTATCGYRTKYTARYTARSGGSGTMWCTTACARRRCGGTAYK 13980
Bhutan-09027        MWACGTCRRRASRGCGTGGTATCGYRTKYTARYTARSRGSGTMWCTTACARRRCGGTAYK 13980
Bhutan-09030        MWACGTCRRRASRGCGTGGTATCGYRTKYTARYTARSRGSGTMWCTTACARRRCGGTAYK 13980
Bhutan-09005        MAMTTWYAGRRSRRYRYRAYRYYRCAYKCWGGYARACRSCAKAAYGYCTTGGAYRRKRTG 13980
Indonesia-88035     CAATTWYAGRRSRRYRCAACATCGCAYKCWGGYAAACGSCAKAAYGYCTTGGAYRRKRTG 13980
Indonesia-88045     CAATTWYAGRRSRRYRCAACATCGCAYKCWGGYAAACGSCATAAYGYCTTGGAYRRKRTG 13980
Indonesia-88065     MAMTTWYAGRRSRRYRYRAYRYYRCAYKCWGGYARACRSCAKAAYGYCTTGGAYRRKRTG 13980
                             * **     .        *  . *:  . ....     . :      .

Bhutan-09015        TRYTTTAKSMCYRCYGAWSTTYSGRSYCAAYYYCRMTCAYRMGKACCYMSRGGWSMRSRK 14040
Bhutan-09024        TRYTTTAKSMCYRCYGAWSTTYSGRSYCAAYYYCRMTCAYRMGKACCYMSRGGWSMRSRK 14040
Bhutan-09027        TRYTTTAKSMCYRCYGAWSTTYSGRSYCAAYYYCRMTCAYRMGKACCYMSRGGWSMRSRK 14040
Bhutan-09030        TRYTTTAKSMCYRCYGAWSTTYSGRSYCAAYYYCRMTCAYRMGKACCYMSRGGWSMRSRK 14040
Bhutan-09005        CATKWCTKCMATAYCRRGCYYTGSRCTMGTYYYSACYMRTGMATGYMCCSRRRWCCAGRT 14040
Indonesia-88035     CATTWCTKCMATAYCRRGCYYTGSRCTMGTYYYSACYMRTGMATGCCCCSRRRWCCAGRT 14040
Indonesia-88045     CATTWCTKCMATAYCRRGCYYTGSRCTMGTYYYSACYMRTGMATGCCCCSRRRWCCAGRT 14040
Indonesia-88065     CATTWCTKCMATAYCRRGCYYTGSRCTMGTYYYSACYMRTGMATGYMCCSRRRWCCAGRT 14040
                       .  :*.*.       .   ..*.  .:***.       *...    **  *.  .*.

Bhutan-09015        YKCYCCAARGCRCRYWYAWSAYRYRMYRYRKYGTWARTTTYYRMMYATYATRTKTTTYYK 14100
Bhutan-09024        YKCYCCAARGCRCRYWYAWSAYRYRMYRYRKYGTWARTTTYYRMMYATYATRTKTTTYYK 14100
Bhutan-09027        YKCYCCAARGCRCRYWYAWSAYRYRMYRYRKYGTWARTTTYYRMMYATYATRTKTTTYYK 14100
Bhutan-09030        YKCYCCAARGCRCRYWYAWSAYRYRMYRYRKYGTWARTTTYYRMMYATYATRTKTTTYYK 14100
Bhutan-09005        YKACMYWWRAMGYRYWTMWSGYAYRMYASRGCAKTWGACATYRMMYRWYGARYGGGKYTW 14100
Indonesia-88035     CKACMYWWRAMGYGYWTMWSGYAYRMYASRGCAKTWGACATCGMMYRWYGARYGGGKYTW 14100
Indonesia-88045     CGACMYWWRAMGYGYWTMWSGYAYRMTASRGCAKTWGACATYRMMYRWYGARYGGGKYTW 14100
Indonesia-88065     YKACMYWWRAMGYRYWTMWSGYAYRMYASRGCAKTWGACATYRMMYRWYGARYGGGKYTW 14100
                      .     *.    **  **.* ***   *  ..   : :   ***  *.:*    .*

Bhutan-09015        KYAGRGCRRCYTTCASYYYYACRYAAGGTRWGRGRRRACRTAYRMCCGSSMGCCGGTGSY 14160
Bhutan-09024        KYAGRGCRRCYTTCASYYYYACRYAAGGTRWGRGRRRACRTAYRMCCGSSMGCCGGTGSY 14160
Bhutan-09027        KYAGRGCRRCYTTCASYYYYACRYAAGGTRWGRGRRRACRTAYRMCCGSSMGCCGGYGSY 14160
Bhutan-09030        KYAGRGCRRCYTTCASYYYYACRYAAGGTRWGRGARRACRTAYRMCCGSSMGCCGGYGSY 14160
Bhutan-09005        KYGTAAGRRYYYWYTSTTTYGSACTRRRKRWTARGGRRTRYMYRCTYGGSASTTARTRST 14160
Indonesia-88035     KYGTAAGRRYYYTCTSTTTTGSACTRRGTRWTAGGRRRTRYMYRCTYGGCASTTARTGCT 14160
Indonesia-88045     KYGTAAGRRYYYTCTSTTTTGSACTRRGTRWTAGGRRRTRYMYRCTYRGSASTTARTRST 14160
Indonesia-88065     KYGTAAGRRYYYWYTSTTTYGSACTRRRKRWTARGRRRTRYMYRCTYRGSASTTARTRST 14160
                    **.  . ** *   :*    ..  :   .**     *  *  **    .. .  .   .

Bhutan-09015        GTCYRGTYYCTWRRCMKWYWYKRYRGTACSYWRMRRYTRRWSAMWYSAKMRTCTTYRMWK 14220
Bhutan-09024        GTCYRGTYYCTWRRCMKWYWYKRYRGTACSYWRMRRYTRRWSAMWYSAKMRTCTTYRMWK 14220
Bhutan-09027        GTCYRGTYYCTWRRCMKWYWYKRYRGTRYSYWRMRRYTRRWSAMWYSAKMRTCTTYRMWK 14220
Bhutan-09030        GTCYRGTYYCTWRRCMKWYWYKRYRGTRYSYWRMRRYTRRWSAMWYSAKMRTCTTYRMWK 14220
Bhutan-09005        ACMCGRKTTYYTGRTAGTCAYGGTRGCACSYARAGRCWRRWSTCTYGRGAAKYWWCGCTK 14220
Indonesia-88035     ACMCGRKTTYYTGRTAGTCAYGGTRGCRYSYARAGRCWRRWSTCTYGRGAAKYWWCGCTK 14220
Indonesia-88045     ACMCGRKTTYYTGRTAGTCAYGGTRRCACSYARAGRCWRRWSTCTYGRGAAKYWWCGCTK 14220
Indonesia-88065     ACMCGRKTTYYTGRTAGTCAYGGTRGCACSYARAGRCWRRWSTCTYGRGAAKYWWCGCTK 14220
                    .     .      *      *   *    ** *  *  ****:  *.    .       *
```

```
Bhutan-09015          RAYATAGRCYKRATYYCYKWTCWRCCYTYCRWGYRKSYWCCRKYWWCCGGACWTTCRKYT 14280
Bhutan-09024          RAYATAGRCYKRATYYCYKWTCWRCCYTYCRWGYRKSYWCCAGYWWCCGGACWTTCRKYT 14280
Bhutan-09027          RAYATAGRCYKRATYYCYKWTCWRCCYTYCRWGYRKSYWCSRKYWWCCGGACWTTCRKYT 14280
Bhutan-09030          RAYATAGRCYKRATYYCYKWTCWRCCYTYCRWGYRKSYWCCAGYWWCCGGACWTTCRKYT 14280
Bhutan-09005          RRTMWGTGACKGRYCCYCTTYGAGAMYKTSRASYAKCCTTGGTTAAYYKKRTAYYYAGYY 14280
Indonesia-88035       GRTMWGTGACKGRYCCYCTTYGAGAMYKTSRASYAKCCTTGGTTAAYYKKATAYYYAGYY 14280
Indonesia-88045       GRTMWGTGACKGRYCCYCTTYGAGAMYKTSRASYAKCCTYGGTTAAYYKKATAYYYAGYY 14280
Indonesia-88065       RRTMWGTGACKGRYCCYCTTYGAGAMYKTSRASYAKCCTTGGTTAAYYKKRTAYYYAGYY 14280
                           .  . *       .     . *. .* .* *.                     *

Bhutan-09015          WGGMMATRKYATRYCRAYCKWAMMYKRRWYCMYCTTRSAYMKCATARGGYTSAMRWSTRY 14340
Bhutan-09024          WGGMMATRKYATRYCRAYCKWAMMYKRRWYCMYCTTRSAYMKCATARGGYTSAMRWSTRY 14340
Bhutan-09027          WGGMMATRKYATRYCRAYCKWAMMYKRRWYCMYCTTRSAYMKCATARGGYTSAMRWSTRY 14340
Bhutan-09030          WGGMMATRKYATRYCRAYCKWAMMYKRRWYCMYCTTRSAYMKCATARGGYTSAMRWSTRY 14340
Bhutan-09005          WRSCMMARTYTAACYGRTTGTRCATKRAATYCTTTWGGGCCKTGAGGARTWGMCGWGYAY 14340
Indonesia-88035       WASCMMARTYTAACYGRTTGTRCATKRAATYCTTTWGGGCCKTGWGGARTWGMCGWGYAY 14340
Indonesia-88045       WRSCMMARTYTAACYGRTTGTRCATKRAATYCTTKWGGGCCKTGWGGARTWGMCGWGYAY 14340
Indonesia-88065       WRSCMMARTYTAACYGRTTGTRCATKRAATYCTTTWGGGCCKTGWGGARTWGMCGWGYAY 14340
                      * . * :*.*::             **       .  ..  * . . .   .   *.  *

Bhutan-09015          CRCTAGYTRYAWRMWYCCGAGGAAKYTKWGWTSRRRATYTRRYRCYYGARCAAAMYACTY 14400
Bhutan-09024          CRCTAGYTRYAWRMWYCCGAGGAAKYTKWGWTSRRRATYTRRYRCYYGARCAAAMYACTY 14400
Bhutan-09027          CRCTAGYTRYAWRMWYCCGRRGAAKYTKWGWTSRRRATYTRRYRCYYGARCAAAMYACTY 14400
Bhutan-09030          CRCTAGYTRYAWRMWYCCGAGGAAKYTKWGWTSRRRATYTRRYRCYYGARCAAAMYACTY 14400
Bhutan-09005          SAYYRSTWRTRARMWTTSRRGRMMGTAGAAAYGGAACCTWRAYATCTSGGYTRTCTTYYC 14400
Indonesia-88035       SAYYRSTWRTRARMWTTSRRGRMMGTAGAGAYGGAACCTWRAYATCTSGGYTRTCTTCTC 14400
Indonesia-88045       SAYYRSTWRTRARMWTTSRRGRMMGTAGAGAYGGAACCTWRAYATCTSGGYTRTCTTCTC 14400
Indonesia-88065       SAYYRSTWRTRARMWTTSRRGRMMGTAGAGAYGGAACCTWRAYATCTSGGYTRTCTTYYC 14400
                      .    .  *   ***  .        :  .  .   .   * *    ..  : :  :

Bhutan-09015          RTARKRRKCYYGKGWGRAYWKSGGAGRWYWKKRATTAAGWGCYCCAWWMGGCTYMCRRYG 14460
Bhutan-09024          RTARKRRKCYYGKGWGRAYWKSGGAGRWYWKKRATTAAGWGCYCCAWWMGGCTYMCRRYG 14460
Bhutan-09027          RTARKRRKCYYGKGTGRAYWKSGGAGRWYWKKRATTAAGWGCYCCAWWMGGCTYMCRRYG 14460
Bhutan-09030          RTARKRRKCYYGKGWGRAYWKSGGAGRWYWKKRATTAAGWGCYCCAWWMGGCTYMCRRYG 14460
Bhutan-09005          AWRATGRKYCTSTKARAMCAKSKRMKGACWKKGMYYRRRAKYTYSRTKATATWCAYAAYR 14460
Indonesia-88035       AWRATGRGCCTSTKARAMCAKSKRAKGACWKKGMYYRRRAKYTYSRTKATATWCAYAAYG 14460
Indonesia-88045       AWRATGRGCCTSTKAGAMCAKSKRAKGACWKKGMYYRRRAKYTYSRTKATATWCAYAAYG 14460
Indonesia-88065       AWRATGRKYCTSTKARAMCAKSKRMKGACWKKGMYYRRRAKYTYSRTKATATWCAYAAYR 14460
                          . *    ..       **       ***            .     .       *

Bhutan-09015          CCCCATRRWRKSRGGYCAYYRRMCWKWGWGKGCARRRAGYRARYYTYAMYTCTGWYTMTR 14520
Bhutan-09024          CCCCATRRWRKSRGGYCAYYRRMCWKWGWGKGCARRRAGYRARYYTYAMYTCTGWYTMTR 14520
Bhutan-09027          CCCCATRAWRKSRGGYCAYYRRMCWKWGWGKGCARRRAGYRARYYTYAMYTCTGWYTMTR 14520
Bhutan-09030          CCCCATRRWRKSRGGYCAYYRRMCWKWGWGKGCARRRAGYRARYYTYAMYTCTGWYTMTR 14520
Bhutan-09005          ATMSWKGAARGSGTRCYATTGRCYAKARWRGCGRGRGRRCGRRCCWTWATYYYRWCTMYR 14520
Indonesia-88035       ATCCAKGAAGKSGTRCYWTTGGCYATARWRGCGRGRGRRCGRRCCWTWATYYCAACYCTA 14520
Indonesia-88045       ATCCAKGAAGKSGTRCYWTTRGCYATARWRGCGRGRGRRCGRRCCWTWATCTCAACYCTA 14520
Indonesia-88065       ATMSWKGAARGSGTRCYATTRRCYAKARWRGCGRGRGRRCGRRCCWTWATCTYRWCTMYR 14520
                      .  . .     *             .  *      *      *

Bhutan-09015          GAGYARTTCTSRRKTYMWAKYTYYRWMYYRSCAGWACACATWTYGGSWWRSRTTCKACRR 14580
Bhutan-09024          GAGYARTTCTSRRKTYMWAKYTYCGWMYYRSCAGWACACATWTYGGSWWRSRTTCKACRR 14580
Bhutan-09027          GAGYARTTCTSRRKTYMWAKYTYYRWMYYRSCAGWACACATWTYGGSWWRSRTTCKACRR 14580
Bhutan-09030          GAGYARTTCTSRRKTYMWAKYTYYRWMYYRSCAGWACACATWTYGGSWWRSRTTCKACRR 14580
```

```
Bhutan-09005        KAGCRRYKCYGRRTYYATCKTYTTRACCYRSYGRWRSCYMWAYCRRCTAGSGCWMGRAAA 14580
Indonesia-88035     KMSTARTKYYGGGTYYATCTTYTTAACCYRSYGRWRSCYMWAYCRRCTAGSGCTMGRAAA 14580
Indonesia-88045     KMSTARTKYYGGGTYYATCTTYTTAACCYRSYGRWRSCYMWAYCRRCTAGSGCTMGRAAA 14580
Indonesia-88065     KAGCRRYKCYGRRTYYATCKTCTTRACCYRSYGRTRSCYMWAYCRRCTAGSGCWMGRAAA 14580
                      .  * .  .  . *  ..        *** .   ..        .   *      .

Bhutan-09015        TCRWRMSAYGRRYKCKRMYSSACSGSYCGRYYYAYRRCYGRYRRRCSRYGGRASYTAWRM 14640
Bhutan-09024        TCRWRMSAYGRRYKCKRMYSSACSGSYCGRYYYAYRRCYGRYRRRCSRYGGRASYTAWRM 14640
Bhutan-09027        TCRWRMSAYGRRYKCKRMYSSACSGSYCGRYYYAYRRCYGRYRRRCSRYGGRASYTAWRM 14640
Bhutan-09030        TCRWRMSAYGRRYKCKRMYSSACSGSYCGRYYYAYRRCYGRYRRRCSRYGGRASYTAWRM 14640
Bhutan-09005        YSRTACGAYRGACGMKRCCCSRMCRCYSKATCTRTRAYTKACGGASCACKRRMSTGGTGA 14640
Indonesia-88035     TCGWACGCYRGACGMKRMYSSGMCRCYSKATCTRTRAYCKACAGASCACKRRACTGGTGA 14640
Indonesia-88045     TCGWACGCYRGACGMKRMYSSGMCRCYSKATCTRTRAYCKACAGASCACKRRACTGGTGA 14640
Indonesia-88065     YSRTACGAYRGACGMKRCCCSRMCRCYSKATCTRTRAYTKACGGASCACKRRMSTGGTGA 14640
                     .    ..*      **  .*  . .*.       *         ..    *  .  .

Bhutan-09015        ARRWYCRYKKSRGKYMAKRCTTYRRRYGKCCASCYAKRYWATYAYRKASKWRKMKTCGYR 14700
Bhutan-09024        ARRWYCRYKKSRGKYMAKRCTTYRRRYGKCCASCYAKRYWATYAYRKASKWRKMKTCGYR 14700
Bhutan-09027        ARRWYCRYKKSRGKYMAKRCTTYRRRYGKCCASCYAKRYWATYAYRKASKWRKMKTCGYR 14700
Bhutan-09030        ARRWYCRYKKSRGKYMAKRCTTYRRRYGKCCASCYAKRYWATYAYRKASKWRKMKTCGYR 14700
Bhutan-09005        RRGTYYAYGTCGSKYCTTRYYYTRAATRKYYRGYTMKGYAACCRYRGGSKTGGCTGYKYR 14700
Indonesia-88035     RRGTYYAYGTCGSKYCTTRYYYTRAATGGTYRGYTMKGYARYCRTRGGSKTGGCTGYKYR 14700
Indonesia-88045     RRGTYYAYGTCGSKYCTTRYYYTRAATGGTYRGYTMKGYARYCRTRGGSKTGGCTGYKYR 14700
Indonesia-88065     RRGTYYAYGTCGSKYCTTRYYYTRAATRKYYRGYTMKGYAACCRYRGGSKTGGCTGYKYR 14700
                     *  *  * .. .** :.*    *        .   * *      * .**    .   **

Bhutan-09015        WYTAWMKCWMMYCMGCCMSSKATYGGSRRTRCKRYKYMWYTTWGYRYAAGWAMMWAGRYG 14760
Bhutan-09024        WYTAWMKCWMMYCMGCCMSSKATYGGSRRTRCKRYKYMWYTTWGYRYAAGWAMMWAGRYG 14760
Bhutan-09027        WYTAWMKCWMMYCMGCCMSSKATYGGSRRTRCKRYKYMWYTTWGYRYAAGWAMMWAGRYG 14760
Bhutan-09030        WYTAWMKCWMMYCMGCCMSSKATYGGSRRTRCKRYKYMWYTTWGYRYAAGWAMMWAGRYG 14760
Bhutan-09005        WTYRWCGYAAACYCRYYASSGRKTKTCARYGYTRYKYAWTKKWKTACRTKTWMMARRACG 14760
Indonesia-88035     WTYRWCGCAAACYMRYYASSGRKTKTCARYGYTRYKYATTKKWKTACRTTTWMMARRACR 14760
Indonesia-88045     WTYRWCGCAAACYMRYYASSGRKTKTCARYGYTRYKYATTKKWKTACRTTTWMMARRACR 14760
Indonesia-88065     WTYRWCGYAAACYCRYYASSGRKTKTCARYGYTRYKYAWTKKWKTACRTKTWMMARRACG 14760
                    *   *             **   .   . *   .****  ..*     :   **

Bhutan-09015        WYARGYWYSWRAAARWRRRYACARAYAGAYKYMYKRKACRKRWKRARYWAKMKCMTRGCA 14820
Bhutan-09024        WYARRYWYSWRAAARWRRRYACARAYAGAYKYMYKRKACRKRWKRARYWAKMKCMTRGCA 14820
Bhutan-09027        WYARRYWYSWRAAARWRRRYACARAYAGAYKYMYKRKACRKRWKRARYWAKMKCMTRGCA 14820
Bhutan-09030        WYARRYWYSWRAAARWRRRYACARAYAGAYKYMYKRKACRKRWKRARYWAKMKCMTRGCA 14820
Bhutan-09005        TCWARYWTCWACRWRWGGATCMRAMTRRRYKCMYTGGRSRGAWGARACWRTAGGAYARSG 14820
Indonesia-88035     TCWARYWTCWACRWRWGGATCMRAMTRRRCKCMYTGGRSRGAWGARACWRTAGGAYARSG 14820
Indonesia-88045     TCWARYWTCWACRWRWGGATCMRAMTRRRCKCMYTGGRSRGAWGARACWRTAGGAYARSG 14820
Indonesia-88065     TCWARYWTCWACRWRWGGATCMRAMTRRRYKCMYTGGRSRGAWGARACWRTAGGAYARSG 14820
                         ** .* .  **    .         * **.   .*  *     * .       ..

Bhutan-09015        RRCGTRRACYRSTRYCTCYCTMYYTTCRMAAWRYMRRRTYSGYTYCKTWYYWSTAKYRAC 14880
Bhutan-09024        RRCGTRRACYRSTRYCTCYCTMYYTTCRMAAWRYMRRRTYSGYTYCKTWYYWSTAKYRAC 14880
Bhutan-09027        RRCGTRRACYRSTRYCTCYCTMYYTTCRMAAWRYMRRRTYSGYTYCKTWYYWSTAKYRAC 14880
Bhutan-09030        RRCGTRRACYRSTRYCTCYCTMYYTTCRMAAWRYMRRRTYSGYTYCKTWYYWSTAKYRAC 14880
Bhutan-09005        ARTRYRARSTRGYACYACYGYAYCYCTAARGAATCARRYYGRYKTTGYTCYWGKAKYGWT 14880
Indonesia-88035     ARTRYRARSTRGTACYAYYGYAYCTCTAARGAATAGGGTYGGYKTTGYTCYWGKTKYGWT 14880
Indonesia-88045     ARTRYRARSTRGTACYAYYGYAYCTCTAAAGAATAGGGTYGGYKTTGYTCYWGKTKYGWT 14880
Indonesia-88065     ARTRYRARSTRGYACYACYGYAYCYCTAARGAATCARRYYGRYKTTGYTCYWGKAKYGWT 14880
```

```
                      *   *  . *.    : *   *       .        *. *.      **..:**

Bhutan-09015         RYGYYYKCRSRWTMKYYATWGTRYGTGTYGGMAAYRYTKARYSYCAAYWYYCYCYYYYYT 14940
Bhutan-09024         RYGYYYKCRSRWTMKYYATAGTRYGTGTYGGMAAYRYTKARYSYCAAYWYYCYCYYYYYT 14940
Bhutan-09027         RYGYYYKCRSRWKMKYYATWGTRYGTGTYRRMAAYRYTKARCSYCAAYWYYCYCYYYYYT 14940
Bhutan-09030         RYGYYYKCRSRWKMKYYATWGTRYGTGTYRRMAAYRYTKARYSYCAAYWYYCYCYYYYYT 14940
Bhutan-09005         RTGYTTGTACRTKMTCCRYAAYACRWRKYRRCRRYRTWTRAYSYTRWYAYTTYMYCTCTG 14940
Indonesia-88035      RTRYTTGTACRTKMTCCRYAAYACRWRKYGGCRRYRTWTRAYSYTAAYAYTTYMYCTCTG 14940
Indonesia-88045      RTRYTTGTACRTKMTCCRYAAYACRWRKYGGCRRYRTWTRAYSYTAAYAYTTYMYCTCTG 14940
Indonesia-88065      RTGYTTGTACRTKMTCCRYAAYACRWRKYGGCRRYRTWTRAYSYTRWYAYTTYMYCTCTG 14940
                     *  *     .* .*.      .      .*      **  .   **   * *  * *

Bhutan-09015         YYAGTRGGGGWRYGMYGSGARRWKMRRYCKGGYRCCTGCGCYYWCYYRSCRYSYCKATWG 15000
Bhutan-09024         YYAGTRGGGGWRYGMYGSGARRWKMRRYCKGGYRCCTGCGCYYWCYYRSCRYSYCKATWG 15000
Bhutan-09027         YYAGTRGGGGWRYGMYGSGARRWKMRRYCKGGYRCCTGCGCYYWCYYRSCRYSYCKATWG 15000
Bhutan-09030         YYAGTRGGGGWRYGMYGSGARRWKMRRYCKGGYRCCTGCGCYYWCYYRSCRYSYCKATWG 15000
Bhutan-09005         CTWRAGRRSRAGTSCYRSRGRAWGMAATYGRRYRATYKYASYYTYCTGGMGTCYCTRKWK 15000
Indonesia-88035      CTWRAGRRGRAGTSCYRSRGRAWGMAATYGRRYRATYKYASYYWCCTGGMGTCYMTGKWK 15000
Indonesia-88045      CTWRAGRAGRAGTSCYRSRGRAWGMAATYGRRYRATYKYASYYWCCTGGMGTCYMTGKWK 15000
Indonesia-88065      CTWRAGRRSRAGTSCYRSRGRAWGMAATYGRRYRATYKYASYYTYCTGGMGTCYCTRKWK 15000
                         :   .    . * * .* * *       **.     ..**      .   .* . .*

Bhutan-09015         CKGGYRSRCWTAAGSYTMKGYWGSSRTGWGRWTRCRWYRRCKKYSCWWCTASARRYSGMR 15060
Bhutan-09024         CKGGYRSRCWTAAGSYTMKGYWGSSRTGWGRWTRCRWYGRCKKYSCWWCTASARRYSGMR 15060
Bhutan-09027         CKGGYRSRCWTAAGSYTMKGYWGSSRTGWGRWTRCRWYGRCKKYSCWWCTASARRYSGMR 15060
Bhutan-09030         CKGGYRSRCWTAAGSYTMKGYWGSSRTGWGRWTRCRWYRRCKKYSCWWCTASARRYSGMR 15060
Bhutan-09005         YKGRYACAYAYRWSGCACGSCTAGSRYRWARWYRGGWYGAYTGCSTWYYKRGMRRYCRMR 15060
Indonesia-88035      YTRRYACAYATRTSGCACGGCTAGSRYRWGRWYRGGWYGAYTGCCTWYCTAGARRYCRMR 15060
Indonesia-88045      YTRRYACAYATRTSGCACGGCTAGSRYRWGRWYRGGWYGAYTGCCTWYCTAGARRYCRMR 15060
Indonesia-88065      YKGRYACAYAYRWSGCACGSCTAGSRYRWARWYRGGWYGAYTGCSTWYYKRGMRRYCRMR 15060
                      .  * .      .. :  .  ..**  *.** *  **   .  .  *: . . ***. **

Bhutan-09015         YAGGYCYTRRRMYWRRYASAYAYRWYAYWGRTWRYKYTYYCRWCCAKACTGTYYARGKRK 15120
Bhutan-09024         YAGGYCYTRRRMYWRRYASAYAYRWYAYWGRTWRYKYTYYCRWCCAKACTGTYYARGKRK 15120
Bhutan-09027         YAGGYCYTRRRMYWRRYASAYAYRWYAYWGRTWRYKYTYYCRWCCAKACTGTYYARGKRK 15120
Bhutan-09030         YAGGYCYTRRRMYWRRYASAYAYRWYAYWGRTWRYKYTYYCRWCCAKACTGTYYARGKRK 15120
Bhutan-09005         TRSRYYTYARRMCWRRTWSRCCYRWYRYAARTYACGTTYYYATCGCGAMYRWCYRGTTRT 15120
Indonesia-88035      TAGRYTTYARRACWRRTWSRCCYRWYRYAARYYACGTKYYYATTGCGWMYGTCYAGTTRT 15120
Indonesia-88045      TAGRYTTYARRACWGGTWSRCCYRWYRYAARYYACGTKYYYATTGCGWMYGTCYAGTTRT 15120
Indonesia-88065      TRSRYYTYARRMCWRRTWSRCCYRWYRYAARTYACGTTYYYATCGCGAMYRWCYRGTTRT 15120
                       . *    **  *    *  .**** * .* :    .**       .       *   .*.

Bhutan-09015         YYRAGWKMCTGCYKRAGTRRKACYAYGCYARCRRAYYARGTTGTTRRKGMAYCYSWCCYR 15180
Bhutan-09024         YYRAGWKMCTGCYKRAGTRRKACYAYGCYARCRRAYYARGTTGTTRRKGMAYCYSWCCYR 15180
Bhutan-09027         YYRAGWKMCTGCYKRAGTRRKACYAYGCYARCRRAYYARGTTGTTRRKGMAYCYSWCCYR 15180
Bhutan-09030         YYRAGWKMCTGCYKRAGTRRKACYAYGCYARCRRAYYARGTTGTTRRKGMAYCYSWCCYR 15180
Bhutan-09005         CTGWKTTAYYRYYKRGAWGRKATYRTRSTWRYRATTTRRACCAYYRRGRARYMTSWYYCA 15180
Indonesia-88035      CTGWKTTACTRYYKRGAWGRKRYYRTRSTWRYRATTTRRACCAYYRRGGARYMTSWYYCA 15180
Indonesia-88045      CTGWKTTACTRYYKRGAWGRKRYYRTRSTWRYRATTTRRACCAYYRRGGARYMTSWYYCA 15180
Indonesia-88065      CTGWKTTAYYRYYKRGAWGRKRTYRTRSTWRYRATTTRRACCAYYRRGGARYMTSWYYCA 15180
                           .     ***..  **  *    .  * * :   *.   .  **     *  **

Bhutan-09015         SRRGGRYKATWAKYAMRTMATRTSRYYYYRYRRKWRSKGTRMAYRYWYCCGAGYGYAYRK 15240
Bhutan-09024         SRRGGRYKATWAKYAMRTMATRTSRYYYYRYRRKWRSKGTRMAYRYWYCCGAGYGYAYRK 15240
```

```
Bhutan-09027        SRRRGRYKATWAKYAMRTMATRTSRYYYYRYRRKWRSKGTRMAYRYWYCCGAGYGYAYRK 15240
Bhutan-09030        SRRRGRYKATWAKYAMRTMATRTSRYYYYRYRRKWRSKGTRMAYRYWYCCGAGYGYAYRK 15240
Bhutan-09005        GAGARRYKWYWRKCWAAWCGYGKCGYCYYACGATAAGTRARARCRTTTYYRRSTRTRYAG 15240
Indonesia-88035     GAGRRRYKWYTAKCWAAWCGYGKCGYCYYACGATAACTRARARCRTTTYYRRSTRTRYAG 15240
Indonesia-88045     GAGRRRYKWYTAKCWAAWCGYGKCGYCYYACGATAACTAARARCRTTTYYRRSTRTRYAG 15240
Indonesia-88065     GAGARRYKWYWRKCWAAWCGYGKCGYCYYACGATAAGTRARARCRTTTYYRRSTRTRYAG 15240
                    .    ***    *      .  .. * **    .   . :*   *       .    *

Bhutan-09015        KGCACRYRWWRKYWWKSRAGYWYSRGTKYWCGACGSYKRRRGCSRWGCRARCYKCYKRYT 15300
Bhutan-09024        KGCACRYRWWRKYWWKSRAGYWYSRGTKYWCGACGSYKRRRGCSRWGCRARCYKCYKRYT 15300
Bhutan-09027        KGCACRYRWWRKYWWKSRAGYWYSRGTKYWCGACGSYKRRRGCSRWGCRARCYKCYKRYT 15300
Bhutan-09030        KGCACRYRWWRKYWWKSRAGYWYSRGTKYWCGACGSYKRRRGCSRWGCRARCYKCYKRYT 15300
Bhutan-09005        KRYRYRYRWTATCATGCAGAYWCCRRWKYASRRMSCTKAGGRMGATGMRRGYYTYCKGYC 15300
Indonesia-88035     KRYRYRYRWTATCATGCAGRYWCCRRWKYASRRMSCTKAGGAMGATRMRRGYYTYCKGYC 15300
Indonesia-88045     KRYRYRYRWTATCATGCAGRYWCCRRWKYASRRMSCTKAGGAMGATRMRRGYYTYCKGYC 15300
Indonesia-88065     KRYRYRYRWTATCATGCAGRYWCCRRWKYASRRMSCTKAGGRMGATGMRRGYYTYCKGYC 15300
                    *    ****  .    . . ** .*  ** .   .. *     .    *   *.  * *

Bhutan-09015        GKYTGRYYWYYYKAWGSCCKCGRYYKAGCMYKKATTCGRCSSYCMTCYYACAARRRKYKR 15360
Bhutan-09024        GKYTGRYYWYYYKAWGSCCKCGRYYKAGCMYKKATTCGRCSSYCMTCYYACAARRRKYKR 15360
Bhutan-09027        GKYTGRYYWYYYKAWGSCCKCGRYYKAGCMYKKATTCGRCSSYCMTCYYACAARRRKYKR 15360
Bhutan-09030        GKYTGRYYWYYYKAWGSCCKCGRYYKAGCMYKKATTCGRCSSYCMTCYYACAARRRKYKR 15360
Bhutan-09005        AGYYKAYTACCYTRTRGMYTYSATYTGATMTKTMYCMRAYCCTMAYMTCWSRWARAKYKA 15360
Indonesia-88035     AGYYKAYTACCYTRTRGMYTYSATTTGATMTKTMYCMRAYCCTMAYMTCWSRWARATTKA 15360
Indonesia-88045     AGYYKAYTACCYTRTRGMYTYSATTTGATMTKTMCCMRAYCCTMAYATCWSRWARATTKA 15360
Indonesia-88065     AGYYKAYTACCYTRTRGMYTYSATYTGATMTKTAYCMRAYCCTMAYMTCWSRWARAKYKA 15360
                    . *   *    *.   .  . .   ... * *.       ..        .   * . *

Bhutan-09015        YTRWMRYRMRCGSRSAYARYCSYMRTRYMAYYKRGTGCKYYGCRWRMCARYMMWAGKCCW 15420
Bhutan-09024        YTRWMRYRMRCGSRSAYARYCSYMRTRYMAYYKRGTGCKYYGCRWRMCARYMMWAGKCCW 15420
Bhutan-09027        YTRWMRYRMRCGSRSAYARYCSYMRTRYMAYYKRGTGCKYYGCRWRMCARYMMWAGKCYW 15420
Bhutan-09030        YTRWMRYRMRCGSRSAYARYCSYMRTRYMAYYKRGTGCKYYGCRWRMCARYMMWAGKCCW 15420
Bhutan-09005        TYRWMGYRMRYAGRGMCRRCSCTAAYRCMGTTKARAATGCTSYGTGATWRYMMATRKMCW 15420
Indonesia-88035     TYRWMGYRMRYAGRGMCRRCSCTAAYRCMGYTKARAATGCTSYGTGATWRYMMATRKCCW 15420
Indonesia-88045     TYRWMGYRMRYAGRGMCRRCSCTAAYRCMGYTKARAATGCTSYGTGATWRYMMATRKCCW 15420
Indonesia-88065     TYRWMGYRMRYAGRGMCRRCSCTAAYRCMGYTKARAATGCTSYGTGATWRYMMATRKMCW 15420
                      *** **** ..*.   * ..    * *.  *  :.    .       **** : *  *

Bhutan-09015        KGTRAAMAYRRYRGYYGYWRRYWRWACCTAYMAKYGACRYGCYYGTRGGYSAYMTKARWY 15480
Bhutan-09024        KGTRAAMAYRRYRGYYGYWRRYWRWACCTAYMAKYGACRYGCYYGTRGGYSAYMTKARWY 15480
Bhutan-09027        KGTRAAMAYRRYRGYYGYWRRYWRWAMYYRYMAKYGACRYGCYYGTRGGYSAYMTKARWY 15480
Bhutan-09030        KGTRAAMAYRRYRGYYGYWRRYWRWAMYYRYMAKYGACRYGCYYGTRGGYSAYMTKARWY 15480
Bhutan-09005        KRWGRWCWTAATRRCTRTAGGYTGAGACYRTMMKYTTMRYATYYRARAKYCGYMYTRAAT 15480
Indonesia-88035     TGWGRWCWTAATRRCTRTAGAYTGAGACYATMMKYTTMRYATYYRARAKYCGYMYTRAAT 15480
Indonesia-88045     TGWGRWCWTAATRRCTRTAGAYTGAGACYRTMMKYTTMRYATYYRARAKYCGYMYTRAAT 15480
Indonesia-88065     KRWGRWCWTAATRRCTRTAGGYTGAGACYATMMKYTTMRYATYYRARAKYCGYMYTRAAT 15480
                    .           *        *   .     * ** : **. ** :*. *..** .

Bhutan-09015        CARACAKYRACYRWSGGWWCYARWGCATTARRYAWCTTRGKWYWTRRYRARYKCCRKRGT 15540
Bhutan-09024        CARACAKYRACYRWSGGWWCYARWGCATTARRYAWCTTRGKWYWTRRYRARYKCCRKRGT 15540
Bhutan-09027        CARACAKYRACYRWSGGWWCYARWGCATTARRYAWCTTRGKWYWTRRYRARYKCCRKRGT 15540
Bhutan-09030        CARACAKYRACYRWSGGWWCYARWGCATTARRYAWCTTRGKWYWTRRYRARYKCCRKRGT 15540
Bhutan-09005        YRAGYWGYARTYRWRRRTATCMAWKYRYWRARTRTYWKARTTTTYARCARATKSYGKRSA 15540
Indonesia-88035     YRAGYWGYARTCRWRRRTATCMAWKYRYWRARTRTYTTARTTTTYARCARATKSYGKRSA 15540
```

```
Indonesia-88045      YRAGYWGYARTCRWRRRTATCMAWKYRYWRARTRTYTTARTTTTYARCARATKSYGKRSA 15540
Indonesia-88065      YRAGYWGYARTYRWRRRTATCMAWKYRYWRARTRTYWKARTTTTYARCARATKSYGKRSA 15540
                        .   *    **         *       *     .  .     *     *.  **.:

Bhutan-09015         TYYYKGMMSGRCSAYYYRWTKGATRKYRCKACYTYARSSMSAYGKRAATGTKMAYRYCYY 15600
Bhutan-09024         TYYYKGMMSGRCSAYYYRWTKGRWRKYRCKACYTYARSSMSAYGKRAATGTKMAYRYCYY 15600
Bhutan-09027         TYYYKGMMSGRCSAYYYRWTKGATRKYRCKACYTYARSSMSAYGKRAMTGTKMAYRYCYY 15600
Bhutan-09030         TYYYKGMMSGRCSAYYYRWTKGRWRKYRCKACYTYARSSMSAYGKRAMTGTKMAYRYCYY 15600
Bhutan-09005         CCTYGRMASRATCRYYTGTCKGATAKYRYKCTYYCRGSGASMTRGRRCWKWTMRCRCTTY 15600
Indonesia-88035      CCTYGRMASRATCRYYTGTCKGATAKYGYKCTYYCRGSGACMTRGRRCWKWTMRCRCTTC 15600
Indonesia-88045      CCTYGRMASRATCRYYTGTCKRATAKYGYKCTYYCRGSGACMTRGRRCWKWTMRCRCTTC 15600
Indonesia-88065      CCTYGRMASRATCRYYTGTCKGATAKYRYKCTYYCRGSGASMTRGRRCWKWTMRCRCTTY 15600
                        *  * *   . **    *    **  *. *    *. .    *     .*  *

Bhutan-09015         AKRYTMCMKYRTTGTTGAGATTGTRYACRWYRATKRATAMTWRYYWYYCKTKRKRTGTKW 15660
Bhutan-09024         AKRYTMCMKYRKTGTTGAGATTGTRYACRWYRATKRATAMTWRYYWYYCKTKRKRTGTKW 15660
Bhutan-09027         AKRYTMCMKYRTTGTTGAGATTGTRYACRWYRATKRATAMTWRYYWYYCKTKRKRTGTKW 15660
Bhutan-09030         AKRYTMCMKYRTTGTTGAGATTGTRYACRWYRATKRATAMTWRYYWYYCKTKRKRTGTKW 15660
Bhutan-09005         RGAYTAYMKYRTKRKARRRRWKKKGYMGRTYGRYKGRCGMYTATYWYYYTWGGTGKTWTT 15660
Indonesia-88035      RGAYYAYMKYRTKRTARRRRWKKTGCMGRTYGRYKGRCGMYTATYWYYYTWGGTGKTWTT 15660
Indonesia-88045      RGAYYAYMKYRKKRTARRRRWKKTGCMGRTYGRYKGRCGMYTATYWYYYTAGGTGKTWTT 15660
Indonesia-88065      RGAYYAYMKYRTKRKARRRRWKKTGYMGRTYGRYKGRCGMYTATYWYYYTWGGTGKTWTT 15660
                        *   ****.. .:     . .    * *   *   .*    **** .   . .  .

Bhutan-09015         AMYAAGCARYMARWWGARRGRMYMRGWGTAGSTRCYKAAYTRASGTWTRRRRYKTRYTRC 15720
Bhutan-09024         AMYAAGCARYMARWWGARRGRMYMRGWGTAGSTRCYKAAYTRASGTWTRRRRYKTRYTRC 15720
Bhutan-09027         AMYAAGCARYMAAWWGARAGRMYMRGWGTAGSTRCYKAAYTRASGTWTRRRRYKTRYTRC 15720
Bhutan-09030         AMYAAGCARYMAAWWGARRGRMYMAGWGTAGSTRCYKAAYTRASGTWTRRRRYKTRYTRC 15720
Bhutan-09005         RATMWCYRACCGAMWARAGCRATCRKAAYMKGKASCGGTYKGWGKWWYGGGGYGWGCKAM 15720
Indonesia-88035      AATAWCYRACCGRMWARAGCRATCRKAAYMKGTASCGGTYKGWGKWWYGGGGYGWGCKAM 15720
Indonesia-88045      AATAWCYRACCGRMWARAGCRATCRKAAYMKGTASCGGTYKGWGKWWYGGGGYGWGCKAM 15720
Indonesia-88065      RATMWCYRACCGRMWARAGCRATCRKAAYMKGKASCGGTYKGWGKWWYGGGGYGWGCKAM 15720
                                .  *.    *      .   .. .  .:*.  .  *     *    .

Bhutan-09015         KYMYSYACTGRMTKYYGKAATSMKSRYWWSRACRRWWYCYYRRYRYMRMMCTYKAYWKWR 15780
Bhutan-09024         KYMYSMACTGRMTKYYGKAATSMKSRYWWSRACRRWWYCYYRRYRYMRMMCTYKAYWKWR 15780
Bhutan-09027         KYMYSCACTGRMTKYYGKAATSMKSRYWWSRACRRWWYCYYRRYRYMRMMCTYKAYWKWR 15780
Bhutan-09030         KYMYSCACTGRMTKYYGKAATSMKSRYWWSRACRRWWYCYYRRYRYMRMMCTYKAYWKWR 15780
Bhutan-09005         GYMYGCACTKRACTTCSKMTYCMKSACTASAWGAATWYMTTAGTRYMRAATKYKRCTYWA 15780
Indonesia-88035      GYMYGATTTKRACTTCSKMTYCMKSACTASAWGAATWYMTTAGTRYMRAATKYKRCTYWA 15780
Indonesia-88045      GYMYGAWAWKRACTTCSKMTYCMKSACTASAWGAATWYMTTAGTRYMRAATKYKRCTYWA 15780
Indonesia-88065      GYMYGAAAAKRACTTCSKMTYCMKSACTASAWGAATWYMTTAGTRYMRAATKYKRCTYWA 15780
                      ***.     *  .  .* : .***    *      **      ****   .**    *

Bhutan-09015         AWCAGKATAYTRMRCTGYWAGWSYMWGMTTYTYCTMRAKTTYYCTCYRMCMTRYTWWWMY 15840
Bhutan-09024         AWCAGKATAYTRMRCTGYWAGWSYMWGMTTYTYCTMRAKTTYYCTCYRMCMTRYTWWWMY 15840
Bhutan-09027         AWCAGKATAYTRCACTGYWAGWSYMWGMTTYTYCTMRAKTTYYCTCYRMCMTRYTWWWMY 15840
Bhutan-09030         AWCAGKATAYTRCACTGYWAGWSYMWGMTTYTYCTMRAKTTYYCTCYRMCMTRYTWWWMY 15840
Bhutan-09005         RWSRGTCWWCKRCAYWRCTRRTSYCTACCYTKCYYARWTYWTCSKYCGAMCWGCYAAACT 15840
Indonesia-88035      RWSRRTCWWCKRCATWRCTRGTGTMTACCYTKCYTARWTTWTCSKYCGAMCWGCYAAACT 15840
Indonesia-88045      RWSRRTCWWCKRCATWRCTAGTGTMTACCYTKCYTARWTTWTCSKYCGAMCTGCYAAACT 15840
Indonesia-88065      RWSRGTCWWCKRCAYWRCTRRTSYCTACCYTKCYYARWTYWTCSKYCGAMCWGCYAAACT 15840
                      *.  ..   .*          .   .    .    * .    ..
```

```
Bhutan-09015        TGRAWRWMKYRSTWRMYGMAGWRKYYRYSTRACSGCTYRWRYAGWKCMGRAYAATRAATY 15900
Bhutan-09024        TGRAWRWMKYRSTWRMYGMAGWRKYYRYSTRACSKYTYRWRYAGWKCMGRAYAATRAATY 15900
Bhutan-09027        TGRAWRWMKYRSTWRMYGMAGWRKYYRYSTRACSKYTYRWRYAGWKCMGRAYAATRAATY 15900
Bhutan-09030        TGRAWRWMKYRSTWRMYGMAGWRKYYRYSTRACSKYTYRWRYAGWKCMGRAYAATRAATY 15900
Bhutan-09005        YRARWGACKTGGYTGACRARTAAGTYRCCCARYGGTYTRWGYAGWKTCSARTWRKARMWT 15900
Indonesia-88035     YRARWGACKTGGYTGACRARGAAGTYRCCCARYGGTYTRWGYAGWKTCSARTWRKARMWT 15900
Indonesia-88045     YRARWGACKTGGYTGACRARGAAGTYRCCCARYGGTYTRWGYAGWKTCSARTWRKAAMWT 15900
Indonesia-88065     YRARWGACKTGGYTGACRARTAAGTYRCCCARYGGTYTRWGYRKWKTCSARTWRKARMWT 15900
                        *   *  .             ** .    .    ** *  **  .     .

Bhutan-09015        YTRGCTKWWWRYRYWAAGYYRTKACSGRYWYASRYGRTGTCKCRTYSCAGYCGCWMCKTR 15960
Bhutan-09024        YTRGCTKWTWRYRYWAAGYYRTKACSGRYWYASRYGRTGTCKCRTYSCAGYCGCWMCKTR 15960
Bhutan-09027        YTRGCTKWWWRYRYWAAGYYRTKACSGRYWYASRYGRTGTCKCRTYSCAGYCGCWMCKTR 15960
Bhutan-09030        YTRGCTKWWWRYRYWAAGYYRTKACSGRYWYASRYGRTGTCKCRTYSCAGYCGCWMCKTR 15960
Bhutan-09005        CYRAYKKWWWRCAYWGRKTTRWTWTGAGTATWGRYKAYSCYTTGKTGYRKTYSTWMAGYA 15960
Indonesia-88035     CYRAYKTAWWRCAYWGRGTTRWTWTGAGTATWGRYKAYSCYTTGKTGYAKTYSTAAAGTA 15960
Indonesia-88045     CYRAYKTAWWRCACWGRGTTRWTWTGAGTATWGRYKAYSCCTTGKTGYAKTYSTAAAGTA 15960
Indonesia-88065     CYRAYKKWWWRCAYWGRKTTRWTWTGAGTATWGRYKAYSCYTTGKTGYRKTYSTWMAGYA 15960
                      *. ..  **   *.    * .  ..     .**   .  .  . .     .   .

Bhutan-09015        GYGRGGAYGWRTKCYRATTTSKYCTAWRGWMRATYCRMCRGRKWCTRTCRRSRTARRGTW 16020
Bhutan-09024        GYGRGGAYGWRTKCYRATTTSKYCTAWRGWMRATYCRMCRGRKWCTRTCRRSRTARRGTW 16020
Bhutan-09027        GYGRGGAYGWRTKCYRATTTSKYCTAWRGWMRATYCRMCRGRKWCTRTCRRSRTARRGTW 16020
Bhutan-09030        GYGRGGAYGWRTKCYRATTTSKYYTMWRGWMRATYCRMCRGRKWCTRTCRRSRTARRGTW 16020
Bhutan-09005        RTKGARGCCTAKTYYRWYGYGKYCCATGRACARYYTRMWGRRKATCRCTRGCAKGRRAKT 16020
Indonesia-88035     RTKGARGYCTAKTYYRTCGCGKYCCATGRACARYYTRMAGRRKATYACTRGCAKGRRAKT 16020
Indonesia-88045     RTKGARGYCTAKTYYRTCGCGKYCCATGRACARYYTRMAGRRKATYACTRGCAKGRRAKT 16020
Indonesia-88065     RTKGARGCCTAKTYYRWYGYGKYCCATGRACARYYTRMWGRRKATCRCTRGCAKGRRAKT 16020
                        . .    .. **    .**           * **   **      * . ..**..

Bhutan-09015        KYMKSATSWCGYKCGRWYYKCRRRYGGYTSRCRMSACGYRCKCSRTYRCYGTCAAARSYM 16080
Bhutan-09024        KYMKSATSWMRCGCGRWYYKCRRRYGGYTSRCRMSACGYRCKCSRTYRCYGTCAMARSYM 16080
Bhutan-09027        KYMKSATSWMRYKCGRWYYKCRRRYGGYTSRCRMSACGYRCKCSRTYRCYGTCAMARSYM 16080
Bhutan-09030        KYMKSATSWMRYKCGRWYYKCRRRYGGYTSRCRMSACGYRCKCSRTYRCYGTCAMARSYM 16080
Bhutan-09005        TTMTCRYSTMRYKSRRATYKMRRGCRRCWGATGMGTYACRYTYSRYYRYCRGYGAWACTA 16080
Indonesia-88035     TTMTCRYSTCGCGSRRATYKMRRGCRGCTGATGMGTTACRYTYCGTCGCCRGYGAWACTA 16080
Indonesia-88045     TTMTCATSTCGCGSRRATYKMRRGCRGCTGATGMGTTACRYTYCGTCGCCRGYGAWACTA 16080
Indonesia-88065     TTMTCRYSTCGCGSRRATYKMRRGCRRCWGATGMGTYACRYTYSGYYRYCRGYGAWACTA 16080
                    . *..  *     . *  ** **      .   *.: . * . .         .   .

Bhutan-09015        TWWYMKYARARYWCRKAKGGCCTCTRTRCASRWKGYKYYYKYRRAYMGWRYRYKWKMGAS 16140
Bhutan-09024        TWWYMKYARARYWCRKAKGGCCTCWRTRCASRWKGYKYYYKYRRAYMGWRYRYKWKMGAS 16140
Bhutan-09027        TWWYMKYARARYWCRKAKGGCCTCWRTRCASRWKGYKYYYKYRRAYMGWRYRYKWKMGAS 16140
Bhutan-09030        TWWYMKYARARYWCRKAKGGCCTCWRTRCACGWKGYKYYYKYRRAYMGWRYRYKWKMGAS 16140
Bhutan-09005        YTRTATYRRGATTSAGWTRRGAKTAGYRYRCGWTCCGCCTGCAARCCAAGTGCGWGMRRS 16140
Indonesia-88035     YTATATYGRGATTSAGWTRRGAKTAGYRYRCGWTCCGCYTGCAARCCAAGTGCGWGMRRG 16140
Indonesia-88045     YTATATYRRGATTSAGWTRRGAKTAGYRYRCGWTCCGCYTGCAARCCAAGTGCGWGMRRG 16140
Indonesia-88065     YTATATYGRGATTSAGWTRRGAKTAGYRYRCGWTCCGCYTGCAARCCAAGTGCGWGMRRS 16140
                         .* *.   .   .   ..    *  . *.             .      * *  .

Bhutan-09015        RYRCGTKCCRTYTGAGGYTMMGAMCGYMYAYRARRKWKWRTMYSAYSWYWMMYTTWARTA 16200
Bhutan-09024        RYRCGTKCCRTYTGAGGYTMMGACCGYMYAYRARRKWKWRTMYSAYSWYWMMYTTWARTA 16200
Bhutan-09027        RYRCGTKCCRTYTGAGGYTMMRACYGYMYAYRARRKWKWRTMYSAYSWYWMMYTTWARTA 16200
Bhutan-09030        RYRCGTKCCRTYTGAGGYTMMGACYGYMYAYRARRKWKWRTMYSAYSWYWMMYTTWARTA 16200
```

```
Bhutan-09005        GCGYSAGSYRWYCSRKAYTCAGACCRTACRTGWAGKTGWGKCTCRYSACWMCYKATWGCG 16200
Indonesia-88035     GCGYSAGSYRWYCSRKAYKCAGWCCGTACRTGWAGKTGWGKCTCRYSACWMCYKATWGCG 16200
Indonesia-88045     GCGYSAGSYRWYCSRKAYKCAGWCCGTACRTGWAGKTGWGKCTCRYSACWMCYKATWGCG 16200
Indonesia-88065     GCGYSAGSYRWYCSRKAYTCAGWCCRTACRTGWAGKTGWGKCTCRYSACWMCYKATWGCG 16200
                        .: . * * .  .*.                *  * .  . **  ** *.:    .

Bhutan-09015        TGGTTYGTKWTRMWRYARYKRKTWTGYCCKGARTRYTAWRYARRAYRCMACTAAAYRRYK 16260
Bhutan-09024        TGGTTYGTKWTRMWRYARYKRKTWTGYCCKGARTRYTAWRYARRAYRCMACTAAAYRRYK 16260
Bhutan-09027        TGGTTYGTKWTRMWRYARYTGGTWTGYCCKGARTRYTAWRYARRAYRCMACTAAAYRRYK 16260
Bhutan-09030        TGGTTYGTKWTRMWRYARYTRKTWTGYCCKGARTRYTAWRYARRAYRCMACTAAAYRATK 16260
Bhutan-09005        GRSCCTAYGTARCTRYWRYTGGAWKRTYYGRWRYRCWMARYGGRRYGCCRMCTMRCGRYK 16260
Indonesia-88035     GGGCCTATGWAAMTRYWRYTGGAWKRTYYGRWRYRCWMARYGGRRYGCCRMCTMRCGRYG 16260
Indonesia-88045     GRSCCTATGWAAMTRYWRYTGGAWKRTYYGRWRYRCWMARYGGRRYGCCRMCTMRCGRYG 16260
Indonesia-88065     GRSCCTAYGTARCTRYWRYTGGAWKRTYYGRWRYRCWMARYGGRRYGMMRMCTMRCGRYK 16260
                      .   .   :   ** **.  :*.        * *    **. * *       :

Bhutan-09015        YCYTYWKKSYRYYATRYGATCRCRSTRRSCWRGKWRYKRATWMMMRCSYRTAARMWWKGC 16320
Bhutan-09024        YCYTYWKKSYRYYATRYGATCRCRSTRRSCWRGKWRYKRATWMMMRCSYRTAARMWWKGC 16320
Bhutan-09027        YCYTYWKKSYRYYATRYGATCRCRSTRRSCWRGKWRYKRATWMMMRCSYRTAARMWWKGC 16320
Bhutan-09030        YCYTYWKKSYRYYATRYGATCRCRSTRRSCWRGKWRYKRATWMMMRCSYRTAARMWWKGC 16320
Bhutan-09005        TYTYYAKKCTGCCTYAYRRYTAMRSYRRSYWASKTRTGRAYAMAMRYSYRWMWRMWTTRM 16320
Indonesia-88035     TCTYYAGGCTGCCTYAYRRYTAMRSYRRSYWASKTRTGRRYAMAMRYSYRWMWRMWTTRM 16320
Indonesia-88045     TCTYYAGGCTGCCTYAYRRYTAMRSYRRSYWASKTRTGRRYAMACRYSYRWMWRMWTTRM 16320
Indonesia-88065     TYTYYAKKCTGCCTYAYRRYTAMRSYRRSYWASKTRTGRAYAMACRYSYRWMWRMWTTRM 16320
                        *    .    :  *       ** *** * .* *  *    *  * ***    *** .

Bhutan-09015        AGSRMRCCATAKTTGCRTKWMKCATACWWATAKSKACKCTGYMRSYGTAWSYMSTMYKSW 16380
Bhutan-09024        AGSRMRCCATAKTTGCRTTWMKCATACWWATAKSKACKCTGYMRSYGTAWSYMSTMYKSW 16380
Bhutan-09027        AGSRMRCCATAKTTGCRTTWMKCATACWWATAKSKACKCTGYMRSYGTAWSYMSTMYKSW 16380
Bhutan-09030        AGSRMRCCATAKTTGCRTTWMKCATACWWATAKSKACKCTGYMRSYGTAWSYMSTMYKSW 16380
Bhutan-09005        AAGGCRTYRKWTKYKYRATTCGATWMYATMKGKGTRTGACRTAAGYRARACCMCYATKGW 16380
Indonesia-88035     TGGGCRTCRKWTKYKYRATTCGATWMYATMKGKGTRTGACRTAAGYRARACCMCYATKGW 16380
Indonesia-88045     TGGGCRTCRKWTKYKYRATTCGATWMYATMKGKGTRTGACRTAAGYRARACCMCYATKGW 16380
Indonesia-88065     AAGGCRTYRKWTKYKYRATTCGATWMYATMKGKGTRTGACRTAAGYRARACCMCYATKGW 16380
                    :..  *   . ..   *:.   .:       ..*..   .     .* :  . *.   *.*

Bhutan-09015        YKCWMYRYAGSKRMGCAYCWATGWYGCCRAWGYTRKRRSRYRGRRYGGYGCATRCRRYGY 16440
Bhutan-09024        YKCWMYRYAGSKRMGCAYCWATGWYGCCRAWGYTRKRRSRYRGRAYRRYGCATRCRRYGY 16440
Bhutan-09027        YKCWMYRYAGSKRMGCAYCWATGWYGCCRAWGYTRGARSRYRGRAYGGYGCATRCRRCGC 16440
Bhutan-09030        YKCWMYRYAGSKRMGCAYCWATGWYGCCRAWGYTRKRRSRYRGRAYGGYGCATRCRRYGY 16440
Bhutan-09005        YTYTCTRTRKCKGCATMCYWRKSWYCTYRTAAYWRGRGCRTRRGGTRRCGTGWGYGGYRY 16440
Indonesia-88035     YTTTCTRTRKCKGCATMCYWRKSWYCTYRTAACARGRGCRTRRGGTRRCKTGWGYGGYRY 16440
Indonesia-88045     YTYTCTATRKCKGCATMCYWRKSWYCTYRTAACARGRGCRTRRGGTGGCKTGWGYGGYRY 16440
Indonesia-88065     YTYTCTATRKCKGCATMCYWRKSWYCTYRTAAYWRGRGCRTRRGGTRRCGTGWGYGGYRY 16440
                    *.         .*  .     * ..**    *: .  *    .* *            .

Bhutan-09015        RCCKYRWARTTMSMSKYWCWYRYYYKRMRMTRCYKYAKYYTCATTTYTKCMYCKRAYRWT 16500
Bhutan-09024        RCCKYRWARTTMSMSKYWCWYRYYYKRMRMTRCYKYAKYYTCATTTYTKCMYCKRAYRWT 16500
Bhutan-09027        ACCKYRWARTTMSMSKYWCWYRYYYKRMRMTRCYKYAKYYTCATTTYTKCMYCKRAYRWT 16500
Bhutan-09030        RCCKYRWARTTMSMSKYWCWYRYYYKRMRMTRCYKYAKYYTCATTTYTKCMYCKRAYRWT 16500
Bhutan-09005        GTGGYAWRGGWMGCCTYWSATAYYTGRCRMYGMYKCRKYCYMWTYWYCTYWTCGACCRWY 16500
Indonesia-88035     GTGGYAWRGGWMGCCTYWSATAYYTGRCRMYGMYKCRKYCYMWKYWYCTYWTCGACCRWY 16500
Indonesia-88045     GTGGYAWRGGWMGCCTYWSATAYYTGRCRMYGMYKCRKYCYMWKYWYCTYWYYKACCRWY 16500
Indonesia-88065     GTGGYAWRGGWMGCCTYWSATAYYTGRCRMYGMYKCRKYCYMWYTWYCTYWTCGACCRWY 16500
```

```
                         * *    *. ..**.   **   * **  **   **      * .      . **

Bhutan-09015         MCRARYRRCTRWRCCCCCGTCRTMWKTGCTTRARTCARWYAYYYRTGWWGYRWRRGAAYY 16560
Bhutan-09024         MCRARYRRCTRWRCCCCCGTCRTMWKTGCTTRARTCARWYAYYYRTGWWGYRWRRGAAYY 16560
Bhutan-09027         MCRARYRRCTRWRCCCCCGTCRTMWKTGCTTRARTCARWYAYYYRTGWWGYRWRRGAAYY 16560
Bhutan-09030         MCRARYRRCTRWRCCCCCGTCRTMWKTGCTTRARTCARWYAYYYRTGWWGYRWRRGAAYY 16560
Bhutan-09005         CYRGATGGTWRWRYYYTYRYTGYCWGCAMKYGRGYMRGWTMCTCGKRWWRCGARRRRGCC 16560
Indonesia-88035      CYRGATGGTWRWRYYYTYRYTGYCWGCAMGTGRGYMRGWTMCTCGKRWWRCGARRRRGCC 16560
Indonesia-88045      CYRGATGGTWRWRYYYTYRYTGYCWGCAMGYGRGYMRGWTMCTCGKRWWRCGARRRRGCC 16560
Indonesia-88065      CYRGATGGTWRWRYYYTYRYTGYCWGCAMGYGRGYMRGWTMCTCGKRWWRCGARRRRGCC 16560
                       *.      ***           *  .          *      . **    **  .

Bhutan-09015         YAYAYYAKMYTYWYKSTYATTATTRKWRYKGYTWYRWTGRSKGYYMYWCRSRSACARRAA 16620
Bhutan-09024         YAYAYYAKMYTYWYKSTYATTATTRGAGTKGYTWYRWTGRSKGYYMYWCRSRSACARRAA 16620
Bhutan-09027         YAYAYYAKMYTYWYKSTYATTATTRGAGTKGYTWYRWTGRSKGYYMYWCRSRSACARRAA 16620
Bhutan-09030         YAYAYYAKMYTYWYKSTYATTATTRKWRYKGYTWYRWTGRSKGYYMYWCRSRSACAGRAA 16620
Bhutan-09005         YTYMTYRKAYGYTCGSYTATCMYYGTTACKRYKAYAAYRGCGRTYATTYGCACRYGGAWM 16620
Indonesia-88035      YTYMTYRKAYGYTCGSYTATCMYYGTTACKRYKAYAAYRGCGRTYATTYGCACRYGGAWM 16620
Indonesia-88045      YTYMTYRKAYGYTCGSYTRWYAYYGTTACKRYKAYAAYRGCGRTYATTYGCACRYGGAWM 16620
Indonesia-88065      YTYMTYRKAYGYTCGSYTATCMYYGTTACKRYKAYAAYRGCGRTYATTYGCACRYGGAWM 16620
                     *:*  * * * *   *             * *. *     .   *     .  .  .

Bhutan-09015         WTTGWWWKTWGGTYWGKTTYRTWGGCYYTYKYCATKRRKRGKWAGYTTTRAAKSYAYCTG 16680
Bhutan-09024         WTTGWWWKTWGGTYWGKTTYRTWGGCYYTYKYCATKRRKRGKWAGYTWTRAATGYAYCTG 16680
Bhutan-09027         WTTKWWWKTWGGTYWGKTTYRTWGGCYYTYKYCATKRRKRGKWAGYTWTRAAKSYAYCTG 16680
Bhutan-09030         WTTGWWWKTWGGTYWGKTTYRTWGGCYYTYKYCATKRRKRGKWAGYTWTRAAKSYAYCTG 16680
Bhutan-09005         AYYTTWWTCARRKCWKGYYCGYTCKYCYWYGYSRYGAGKRSTTRKYCWWRGRKSYGTYKR 16680
Indonesia-88035      AYYTTWWTCARRKCWKGYYCGYTCKYCYWYGYSRYGAGKRSTTRKYCTTRGRKSYGYYKR 16680
Indonesia-88045      AYYTTWWTCARRKCWKGYYCGYTCKYCYWYGYSRYGAGKRSTTRKYCTTRGRKGYGYYKR 16680
Indonesia-88065      AYYTTWWTCARRKCWKGYYCGYTCKYCYWYGYSRYGAGKRSTTRKYCWWRGRKSYGYYKR 16680
                          **.    . *            * * *.     **..   *   *. ..*.  .

Bhutan-09015         SCCCWGCGTWRRTYACSRAGARWKRRKATSRAAYKCTYYTTYYYCAYRKCKRYCRYGASS 16740
Bhutan-09024         SCCCWGCGTWRRTYACSRAGARWKRRKATGGAAYKCTYYTTYYYCAYRKCKRYCRYGASS 16740
Bhutan-09027         SCCCWGCGTWRRTYACSRAGARWKRRKATGGAAYKCTYYKTYYYCAYRKCKRYCRYGASS 16740
Bhutan-09030         SCCCWGCGTWRRTYACSRAGARWKRRKATSRAAYKCTYYKTYYYCAYRKCKRYCRYGASS 16740
Bhutan-09005         SYYYARSRWAWAYCGYGRWKTRWGARTRYGGAGYTSACTTYCYTYGCGGGTWCMACSRGS 16740
Indonesia-88035      SYYYARSRWAWAYCGYGRWGTRWGARTRYGGAGYTCACTKYCYTYGCGGGTWCMACSRGS 16740
Indonesia-88045      SYYYARSRWAWAYCGYGRWGTRWGARTRYSRAGYTSACTKTCYTYGCGGGTWCMACSRGS 16740
Indonesia-88065      SYYYARSRWAWAYCGYGRWKTRWGARTRYSRWGYTSACTKTCYTYGCGGGTWCMACSRGS 16740
                     *     .       . .*  :**  *.  .  .*..:  .  *  .    .     . .*

Bhutan-09015         ATTKGCYYAWYWMRMKYYYMRCCMYSYRRTYRMAWMTYKAGRGCRCMRTATYAYTRSTCR 16800
Bhutan-09024         ATTKGCYYAWYWMRMKYYYMRCCMYSYRRTYRMAWMTYKAGRGCRCMRTATYAYTRSTCR 16800
Bhutan-09027         ATTKGCYYAWYWMRMKYYYMRCCMYSYRRTYRMAWMTYKAGRGCRCMRTATYAYTRSTCR 16800
Bhutan-09030         ATTKGCYYAWYWMRMKYYYMRCCMYSYRRTYRMAWMTYKAGRGCRCMRTATYAYTRSTCR 16800
Bhutan-09005         WYWKRYCCRWYAMAAGCYCAGMYMTGYRRTCACCAMYYGRRRKCATCGYMGYRCYACWYA 16800
Indonesia-88035      WYWKRYCCRWYAMAAGCYCAGMYMTGYRGKCACCACCCGRRAKCATCGYMGYRCYACWYA 16800
Indonesia-88045      WYWKRYCCRWYAMAAGCYCAGMYMTGYRGKCACCACCCGRRAKCATCGYMGYRCYACWYA 16800
Indonesia-88065      WYWKRYCCRWYAMAAGCYCAGMYMTGYRRTCACCAMYYGRRRKYATCGYMGYRCYACWYA 16800
                        *     ** *    *     * .**  .   .                 *    .

Bhutan-09015         CYRYMMYGCTYYCRTAWGGCTTYRCTKRAKYRMRWCYYCCCKGCGTWMSTTWAMAYAGWG 16860
Bhutan-09024         CYRYMMYGCTYYCRTAWRGCTTYRCTKRAKYRMRWCYYCCCKGCGTWMSTTWAMAYAGWG 16860
```

```
Bhutan-09027           CYRYMMYGCTYYCRTAWRGCTTYRCTKRAKYRMRWCYYCCCKGCGTWMSTTWAMAYAGWG 16860
Bhutan-09030           CYRYMMYGCTYYCRTAWRGCTTYRCTKRAKYRMRWCYYCCCKGCGTWMSTTWAMAYAKWG 16860
Bhutan-09005           MCGYCACATYYCTRKRWRAMYYYAMWTRGGCAMRTMCCYYYGAYRYWMSKYARMRYGGWA 16860
Indonesia-88035        MCGYCACATCTCTRKRWRAMYYYAMWTRGGCAMRTMCCYYYGAYGYWMSKYARMRYGGWA 16860
Indonesia-88045        MCGYCACATCTCTRKRWRAMYYYAMWTRGGCAMRTACCYYYGAYRYWMSKYARMRYGGWA 16860
Indonesia-88065        MCGYCACATCTCTRKRWRAMYYYAMWTRGGCAMRTMCCYYYGAYRYWMSKYARMRYGGWA 16860
                          *   .     *. * .   *   .*.   **        .   ***.   * *. *.

Bhutan-09015           TYARSMRMWATTCGACTTAGTGARRCAARRRYWKTYRSMWKSGACTATYSMWYRTRYWMA 16920
Bhutan-09024           TYARSMRMWATTCGACTTAGTGARRCAARRRTWKTYRSMWKSGACTATYSMWYRTRYWMA 16920
Bhutan-09027           TYARSMRMWATTCGACTTAGTGARRCAARRRYWKTYRSMWKSGACTATYSMWYRTRYWMA 16920
Bhutan-09030           TYARSMRMWATTCGACTTAGTGARRCAARRRYWKTYRSMWKSGACTATYSMWYRTRYWMA 16920
Bhutan-09005           TTMRCWSMWTKKYRRYYYWKYRRRGTRWASGYTKYYGSMWKCRMYYRYCGMWYGWRTWMT 16920
Indonesia-88035        TTMRCWSMWTKKYRRYYYWKYRRRGTRWASGYTKYYGSMWKCRMYYRYCGMWYGWRTWMT 16920
Indonesia-88045        KTMRCWSMWTKKYRRYYYWKYRRRGTRWASGYTKYYGSMWKCRMYYRYCGMWYGWRTWMT 16920
Indonesia-88065        TTMRCWSMWTKKYRRYYYWKYRRRGTRWASGYTKYYGSMWKCRMYYRYCGMWYGWRTWMT 16920
                       .  *.  **:..           *         * * ****.       .***  * **:

Bhutan-09015           RYKWKYYWRAYCGGCYCTTRRRKACGRYACWGAMRAAYTGCTRYSYSMMKKTYAWGYAGK 16980
Bhutan-09024           RYKWKYYWRAYCGGCYCTTRRRKACGRYACWGAMRAAYTGCTRYSYSMMKKTYAWGYAGK 16980
Bhutan-09027           RYKWKYYWRAYCGGCYCTTRRRKACGRYACWGAMRAAYTGCTRYSYSMMKKTYAWGYAGG 16980
Bhutan-09030           RYKWKYYWRAYCGGCYCTTRRRKACGRYACWGAMRAAYTGCTRYSYSMMKKTYAWGYAGG 16980
Bhutan-09005           ACTTGCYWGMTYRRMTYYYAARTMYRRYRYWSRARTCYWKMKRYGTCCMGTKTWARCAGG 16980
Indonesia-88035        ACTTGCYWRMTCAAMTYYYAARTMYRRYRYWSRARTCYWKMKRYGTCCMGTKTWARCWRG 16980
Indonesia-88045        ACTTGCYWRMTCAAMTYYYAARTMYRRYRYWSRARTCYWKMKRYGTCCMGTKTWARCARG 16980
Indonesia-88065        ACTTGCYWGMTYRRMTYYYAARTMYRRYRYWSRARTCYWKMKRYGTCCMGTKTWARCARG 16980
                         .   **             *.   **  *.  *:.*   .**. . * ..

Bhutan-09015           GWGGWYYYSYWCCYCRYARGYYYRRTYYKCGGSRWMRACYGTRYRYRAWWSGRCYWRCYY 17040
Bhutan-09024           GWGGWYYYSYWCCYCRYARGYYYRRTYYKCGGSRWMRACYGTRYRYRAWWSGRCYWRCYY 17040
Bhutan-09027           GWGGWYYYSYWCCYCRYARGYYYRRTYYKCGGSRWMRACYGTRYRYRAWWSGRCYWRCYY 17040
Bhutan-09030           GWGGWYYYSYWCCYCRYARGYYYRRTYYKCGGSRWMRACYGTRYRYRAWWSRRCYWRCYY 17040
Bhutan-09005           RWARWYCTCCWYYTSGTWGCYTCGRGYYKYRKCRWARRYCRYGYRTATWWSRGYYAGYYC 17040
Indonesia-88035        RTARWYCTCCWYYTSGTWGCYTCGRGYYKYRKCRWARRYCRYGYRTATWWSRGYYAGYYC 17040
Indonesia-88045        RTARWYCTCCWYYTSGTWGCYTCGRGYYKYRKCRWARRYCRYGYRTATWWSRGYYAGYYC 17040
Indonesia-88065        RWARWYCTCCWYYTSGTWGCYTCGRGYYKYRKCRWARRYCRYGYRTATWWSRGYYAGYYC 17040
                         . **  . *   .     *   * ***   .** *      **  :***   *   *

Bhutan-09015           CAYACGAMGTTGYACWWRCAYKYRRGCYTYRTRKRGWGGWYRRGSCCMYSYTCATWWAYT 17100
Bhutan-09024           CAYACGAMGTTGYACWWRCAYKYRRGCYTYRTRKRGWGGWYRRGSCCMYSYTCATWWAYT 17100
Bhutan-09027           CAYACGAMGTTGYACWWRCAYKYRRGCYTYGTRKRGWGGWYRRGSCCMYSYTCATWWAYT 17100
Bhutan-09030           CAYACGAMGTTGYACWWRCAYKYRRGCYTYRTRKRGWGGWYRRGSCCMYSYTCATWWAYT 17100
Bhutan-09005           YRCGSWWMRCYRCMSTTRYRCGTRRRYYYTAYATGRARKTTGGASTYAYCCYYACATCCW 17100
Indonesia-88035        YRCRSWWMRCYRCMSTTRKRCGTRRRYYYTAYATGRARKTTGGASTYAYCCYYACATCCW 17100
Indonesia-88045        YRCRSWWMRCTGCMSTTRKRCGTRRRYYYTAYATGRARKTTGGASTYAYCCYYRCATCCW 17100
Indonesia-88065        YRCGSWWMRCYRCMSTTRYRCGTRRRYYYTAYATGRARKTTGGASTYAYCCYYACATCCW 17100
                           .  *      .  *     **  *     .         .*   *.       .

Bhutan-09015           ARWSRKRRATYCYYTMYYMAKRYYTCCTYAGTWAGGRRTTTGRYCYRCCCCTTRYGMRTY 17160
Bhutan-09024           ARWSRKRRATYCYYTMYYMAKRYYTCCTYAGTWAGGRRTTTGRYCYRCCCCTTRYGMRTY 17160
Bhutan-09027           ARWSRKRRATYCYYTMYYMAKRYYTCCTYAGTWAGGRRTTTGRYCYRCCCCTTRYGMRTY 17160
Bhutan-09030           ARWSRKRRATYCYYTMYYMAKRYYTCCTYAGTWAGGRRTTTGRYCYRCCCCTTRYGMRTY 17160
Bhutan-09005           RGWSATAGACTACYYMCCCRTGYYKYMKCRSKATAARRCYYRGYTCRYYTMYWRYRMRYT 17160
Indonesia-88035        RGWSATARRCTACYYMCCCRTGYYTCMKCRSKATAARRCYYRGYTCRYYTMYWRYRMRYT 17160
```

```
Indonesia-88045     RGWSATARRCTACYYMCCCRTGYYKYMKCRSKATAARACYYRGYTCRYYTMYWRYRMRYT 17160
Indonesia-88065     RGWSATARRCTACYYMCCCRTGYYKYMKCRSKATAARRCYYRGYTCRYYTMYWRYRMRYT 17160
                      ** .     . * *    . **.  .  .. :..*      *  *      ** **

Bhutan-09015        YCWCTGRACGRAACSKGKWRKRCRYWRYYSMWGMGYCSYRCRTRSCWASACGTGCRRRYG 17220
Bhutan-09024        YCWCTGRACGRRACSKGKWRKRCRYWRYYSMWGAGYCSYRCRTRSCWASACGTGCRRRYG 17220
Bhutan-09027        YCWCTGRACGRRACSKGKWRKRCRYWRYYSMWGAGYCSYRCRTGCCWASACGTGCRRRYG 17220
Bhutan-09030        YCWCTGRACGRRACSKGKWRKRCRYWRYYSMWGAGYCSYRCRTRSCWASACGTGCRRRYG 17220
Bhutan-09005        TTWYGRGRSRRRRYCKRKWRKGYRYWACTSAYAMGTMCYATRYGCCTAGRTRYRYGRGCR 17220
Indonesia-88035     TTWYGRGRSRRRRYCKRKWRKGYRYWACTSAYAAGTMCYATRYGCSWRGRTRYRYGRGCR 17220
Indonesia-88045     TTWYGRGRSRRRRYCKRKWRKGYRYWACTSAYAARTMCYATRYGCSWRGRTRYRYGRGCR 17220
Indonesia-88065     TTWYGRGRSRRRRYCKRKWRKGYRYWACTSAYAMGTMCYATRYGCSWRGRTRYRYGRGCR 17220
                      *     . *   .* ****  ***   * :.    .*  *  ..  .       *

Bhutan-09015        RRYARMCGACSRRCGMRMRRYARKGTSTGTCGAAYMMRCGTTGTATCCMRTGRTGRTGRY 17280
Bhutan-09024        RRYARMCGACSRRCGMRMRRYAGKGTSTGTCGAAYMMRCGTTGTATCCMRTGRTGRTGRY 17280
Bhutan-09027        RRYARMCGACSRRCGMRMRRYAGKGTSTGTCGAAYMMRCGTTGTATCCMRTGRTGRTGRY 17280
Bhutan-09030        RRYARMCGACSRRCGMRMAAYAGKGTSTGTCGAAYMMRCGTTGTATCCMRTGRTGRTGRY 17280
Bhutan-09005        RGCRRMYRRTSARSRAGCGGCTGKRYGKRYSRRAYMMRTKGYRYRYCTCRCRRKRAKKGT 17280
Indonesia-88035     RGCRRMYRRTSARSRAGCGGCTGKRYGKACGRRGYMMRTKGYRYRYCYCRCRRKGAKKGT 17280
Indonesia-88045     RGCRRMYRRTSARSRAGCGGCTGKRYGKACGRRGYMMRTKGYRYRYYYCRCRRKGAKKGT 17280
Indonesia-88065     RGCRRMYRRTSARSRAGCGGCTGKRYGKRYSRRAYMMRTKGYRYRYCYCRCRRKRAKGGT 17280
                    *   **    * *.       : *  ..     .****           *  *.  .

Bhutan-09015        GTWSRCRYAAYAKCYTGGYYGRMAGTSCYRYARAGRKATGGRTAWRRCTMACRWRAAYGA 17340
Bhutan-09024        GTWSRCRYAAYAKCYTGGYYGRMAGTSCYRYARAGRKATGGRTAWRRCTMACRWRAAYGA 17340
Bhutan-09027        GTWSRCRYAAYAKCYTGGYYGRMAGTSCYRYARAGRKATGGRTAWRRCTMACRWRAAYGA 17340
Bhutan-09030        GTWSRCRYAAYAKCYTGGYYGRMAGTSCYRYARAGRKATGGRTAWRRCTMAYRWRAAYGA 17340
Bhutan-09005        GTACRAAYRRCWTACWRKTYSRMRSKSYTACRGWKRKMCRRAYRAARMKCRCRWRWMTRR 17340
Indonesia-88035     RYACRAAYRRCWTACWRKTYSRMRSKSYTACRGWKRKMCRRAYRAARMKCRCRWRWMTRR 17340
Indonesia-88045     RYACRAAYRRCWTACWATTYSRMRSKSYTACRGWKRKMCRRAYRAARMKCRCRWRWMTRR 17340
Indonesia-88065     GTACRAAYRRCWTACWRKTYSRMRSKSYTACRGWKRKMCRRAYRAARMKCRCRWRWMTRR 17340
                       .*. *    ..     *.** ..*        **         * .   ***

Bhutan-09015        YMCRCKYCYGKYTAYMCYKTYRYCRGCCGMRWSCYRRGWTGRYTMMMRGTWTTGRRCCRR 17400
Bhutan-09024        YMCRCKYCYGKYTAYMCYKTYRYCRGCCGMRWSCYRRGWTGRYTMMMRGTWTTGRRCCRR 17400
Bhutan-09027        YMCRCKYCYGKYTAYMCYKTYRYCRGCCGMRWSCYRRGWTGRYTMACRGTWTTGRRCCRR 17400
Bhutan-09030        YMCRCKYCYGKYTAYMCYKTYRYCRGCCGMRWSCYRRGWTGRYTMMMRGTWTTGRRCCRR 17400
Bhutan-09005        YCARYKYTYRKYYRTCATGYCGCYRSSMRAGTSYTAGSWWRATWCCAGRAWAWKGGYSAA 17400
Indonesia-88035     YCARYKYTYRKYYRTCATGYCGCYRSSMRAGTSYTAGSATRATWCCAGRAWAWKGGYSAA 17400
Indonesia-88045     YCARYKYTYRKYYRTCATGYCGCYRSSMRAGTSYTAGSATRATWCCAGRAWAWKGGYSAA 17400
Indonesia-88065     YCARYKYTYRKYYRTCATGYCGCYRSSMRAGTSYTAGSWWRATWCCAGRAWAWKGGYSAA 17400
                    * .* ** * **    .       *..     *    .           :*:     .

Bhutan-09015        YYRTYRRRCYWRTYYRCTTGTYAYCYTRCRCAKCRTAATYRAYYRGMYRRRCARWTTAYT 17460
Bhutan-09024        YYRTYRRRCYWRTYYRCTWGKYAYCYTRCRCAKCRTAATYRAYYRGMYRRRCARWTTAYT 17460
Bhutan-09027        YYRTYRRRCYWRTYYRCTTGTYAYCYTRCRCAKCRTAATYRAYYRGMYRRRCARWTTAYT 17460
Bhutan-09030        YYRTYRRRCYWRTYYRCTWGTYAYCYTRCRCAKCRTAATYRAYYRGMYRRRCARWTTAYT 17460
Bhutan-09005        CYAWCSRGTTWRWTCRYCWGKCRYYYWRTRSWKYGYRRWYRGTYASMCRKRYGGTYYRCG 17460
Indonesia-88035     CYAWCSRGTTWRWTCRYCTGTYATTTARTRCWKYGYRRAYRGTYASMCRKRYAGTYYACG 17460
Indonesia-88045     CYAWCSRGTTWRWTCRYCTRTYAYYYWRTRCWKYGYRRAYRGTYASMCRKRYAGTYYACG 17460
Indonesia-88065     CYAWCSRGTTWRWTCRYCTGTCRYYYWRTRSWKYGYRRWYRGTYASMCRKRYGGTYYACG 17460
                     *    *   **   *    .      * *. *      **. * .* *:* .
```

```
Bhutan-09015         ARMKYYSCGKYRWMYWAAMKYRTRRYTSTTRCWCAMRSTACAGTRAYTARTCTATAMRKY 17520
Bhutan-09024         ARMKYYSCGKYRWMYWAAMKYRTRRYTSTKRCWCAMRSTACAGTRAYTARTCTATAMRKY 17520
Bhutan-09027         RRMKYYSCGKYRWMYWAAMKYRTRRYTSTKRCWCAMRSTACAGTRAYTARTCTATAMRKY 17520
Bhutan-09030         RRMKYYSCGKYRWMYWAAMKYRTRRYTSTTRCWCAMRSTACAGTRAYTARTCTATAMRKY 17520
Bhutan-09005         RGAKYTGTRGCRTCTTWRMTYATRRYKCYTRYWYRMAGAGGTKCRMYCWGKYWTGGCAKY 17520
Indonesia-88035      RGAKYTGTRGCRTCTTWRMTYATRRYKCYTAYWYRMAGAGGTTCRMYCWGKYWTGGCAKY 17520
Indonesia-88045      RGAKYTGTRGCRTCTTAGMTYAKRRYKCYKRYWYRMAGAGGTKCRMYCWGKYWTGGCAKY 17520
Indonesia-88065      RGAKYTGTRGCRTCTTAGMTYATRRYKCYTRYWYRMAGAGGTKCRMYCWGKYWTGGCAKY 17520
                        ** .    *      *.* .***.. .  *  * .:. :  * *   .  : .  **

Bhutan-09015         RTGGRAYATSWSSWYRYRKRKCTYKGRARRTSYCARRRYAASRGSATTRGRYMRMYYRCT 17580
Bhutan-09024         RTGGRAYATSWSSWYRYRKRKCTYKGRARRTSYCARRRYAASRGSATTRGRYMRMYYRCT 17580
Bhutan-09027         RTGGRAYATSWSSWYRYRKRKCTYKGRARRTSYCARRRYAASGGSATTRGRYMRMYYRCT 17580
Bhutan-09030         RTGGRAYATSWSSWYRYRKRKCTYKGRARRTSYCARRRYAASGGSATTRGRYMRMYYRCT 17580
Bhutan-09005         GCSRARYRWCTGSWYAYRKRKSYCTRARAAKSTMRGARYTTSGRCCACGGATAKACTAGC 17580
Indonesia-88035      GCSRARYRWCTGSWYAYRKRKSYCTRARAAKSTMRGARYTTSGRCCACGRATAKACTAGC 17580
Indonesia-88045      GYSRARYRWCTGSWYAYRKRKSYCTRARAAKSTMRGARYTTSGRCCACGRATAKACTAGC 17580
Indonesia-88065      GYSRARYRWCTGSWYAYRKRKSYCTRARAAKSTMRGARYTTSGRCCACGGATAKACTAGC 17580
                       .   *  . .*** *****.  .     .*     **::*  ..:      :

Bhutan-09015         RRRSAAARYSMCGMYWTTWYYYGACWCRTRYRAYTSWGMTWKARWGARTRGMWCTRATCC 17640
Bhutan-09024         RRRSAAARYSMCGMYWTTWYYYGACWCRTRYRAYTSWGMTWKARWGARTRGMWCTRATCC 17640
Bhutan-09027         RRRSAAARYSMCGMYWTTWYYYGACWCRTRYRAYTSWGMTWKARWGARTRGMWCTRATCC 17640
Bhutan-09030         RRRSAAARYSMCGMYWTTWYYYGACWCRTRYRAYTSWGMTWKARWGARTRGMWCTRATCC 17640
Bhutan-09005         ARAGRRMACGCTRMYTWGAYCTCGAAYRKGCACYCGWRATAGTGWKGRCGTMWYKGRYYT 17640
Indonesia-88035      ARAGRRMACGCTRMYTWGAYCTGGAAYRKGCACYCGWRAKAGTGWKGRCGTMWYKGRYYT 17640
Indonesia-88045      ARAGRRMACGCTRMTTWGAYCTGGAAYRKGCACYCGWRAKAGTGWKGRCGTMWYKGRYYT 17640
Indonesia-88065      ARAGRRMACGCTRMYTWGAYCTCGAAYRKGCACYCGWRAKAGTGWKGRCGTMWYKGRYYT 17640
                      * .     .   *     *   ..  *.   .* .*  .   : * .*   ** .

Bhutan-09015         YKRGTRKARAYYWAGYYYSAGCYKCAGARKRMKRAGTMYGCYAMKSKGGYYYYGGWWRYT 17700
Bhutan-09024         YKRGTRKARAYYWAGYYYSAGCYKCAGARKRMKRAGTMYGCYAMKSKGGYYYYGGWWRYT 17700
Bhutan-09027         YKRGTRKARAYYWAGYYYSAGCYKCAGAAKRMKRAGTMYGCYAMKSKGGYYYYGGWWRYT 17700
Bhutan-09030         YKRGTRKARAYYWAGYYYSAGCYKCAGARKRMKRAGTMYGCYAMKSKGGYYYYGGWWRYT 17700
Bhutan-09005         CKGRGRGWRTTYWRSCYYGRSYTGSRKWGKAMKRAAKACKMCGATSTTSTYCYRRWWRTY 17700
Indonesia-88035      CKGRGRGWATTYWRSCYYGRSYTGSRKWGKAMKRAAKACKMCGATSTTSTYCYRRWWRTY 17700
Indonesia-88045      CKGRGRGWRTTYWRSCYYGRSYTGSRKWGKACKRARKACKMCGATSTTSTYCYRRWWRTY 17700
Indonesia-88065      CKGRGRGWRTTYWRSCYYGRSYTGSRKWGKAMKRWAKACKMCGATSTTSTYCYRRWWRTY 17700
                      *   *   : ** . **. .    .    *  **   .      . .*. . * *  ***

Bhutan-09015         KCARRYKSCCMTTARTWAWWCYYTRMMYCGRCYAYWAWCARRCGMYRARCRTGGAATTAC 17760
Bhutan-09024         KCARRYKSCCMTTARYWAWWCYYTRMMYCGRCYAYWAWCARRCGMYRARCRTGGAATTAC 17760
Bhutan-09027         KCARRYKSCCMTTARTWAWWCYYTRMMYCGRCYAYWAWCARRCGMYRARCRTGGAATTAC 17760
Bhutan-09030         KCARRYKSCCMTTARTWAWWCYYTRMMYCGRCYAYWAWCARRCGMYRARCRTGGAATTAC 17760
Bhutan-09005         TYRRGYTGYMCYYWAYTWTWYYTWGACYYKAYTRCTWWYRRGTRCYRRRYRWRRWWKKRY 17760
Indonesia-88035      TYRRGYTGYMCYYWAYTWTWYYTWGACCTKAYTRCTWWYRRGTRCYRRRTRWRRWWKKGT 17760
Indonesia-88045      TYRRGYTGYMCYYWAYTWTTYYTWGACCTKAYTRCTWWYRRGTRCYRRRTRWRRWWKGRY 17760
Indonesia-88065      TYRRGYTGYMCYYWAYTWTWYYTWGACYYKAYTRCTWWYRRGTRCYRRRYRWRRWWKKRY 17760
                     .  * *..              *                *  *    ** * *      .

Bhutan-09015         ATTACTCATRSYMRKATRRWAYYGCTRWRCCKTTRCYAMSYGWWGKWATAWWRAGMGATW 17820
Bhutan-09024         ATTACTCATRSYMRKATRRWAYYGCTRWRCCKTTRCYAMSYGWWGKWATAWWRAGMGATW 17820
Bhutan-09027         ATTACTCATRSYMRKATRRWAYYGCTRWRCCKTTRCYAMSYGWWGKWATAWWRAGMGATW 17820
Bhutan-09030         ATTACTCATRSYMRKATRRWAYYGCTRWRCCKTTRCYAMSYGWWGKWATAWWRAGMGATW 17820
```

```
Bhutan-09005       MKWMMYYMYACTAGTAKAAWMCTAYTGGASYTGYAMYRCCCRATRGTWKRTAGCKCRRYT 17820
Indonesia-88035    MKWMMYYMYACTAGTAKAAWMCTAYKGGACCTTYAMYRCCCRATRGTWKRTAGCKCRRYT 17820
Indonesia-88045    MKWMMYYMYACTAGTWKAAWMCTAYKGGACCTTYAMYRCCCRATRGTWKRTAGCKCRRYT 17820
Indonesia-88065    MKWMMYYMYACTAGTWKAAWMCTAYTGGASYTGYAMYRCCCRATRGTWKRTAGCKCRRYT 17820
                    .        .   . .  *   . .   . .    *  .        .    .

Bhutan-09015       TRMCATRGAKAYRTYKTTYMCTMCTATYCMARAYKACYYCRKRRTCYYCTTGTTAKMYWY 17880
Bhutan-09024       TRMCATRGAKAYRTYKTTYMCTMCTATYCMARAYKACCYCRKRRTCYYCTTGTTAKMYWY 17880
Bhutan-09027       TRMCATRGAKAYRTYKTTYMCTMCTATYCAARAYKACYYCRKRRTCYYCTTGTTAKMYWY 17880
Bhutan-09030       TRMCATRGAKAYRTYKTTYMCTMCTATYCAARAYKACYYCRKRRTCYTCTTGTTAKMYWY 17880
Bhutan-09005       WAAYRTRKRGRYRYTKKYYAYWMTWTTYYCRGRCGCYCTYGTRGCSYTGGYTWGMGCCAC 17880
Indonesia-88035    WAAYRWGKRGRYRYTTKYYAYWMTAWKYYCRGRCGCYCTYGTRGCSYTGGYTWGMGCCAC 17880
Indonesia-88045    WAAYRWGKRGRYRYTTKYYAYWMYWWKYYCRGRCGCYCTYGTRGCSYTGGYTWGMGCCAC 17880
Indonesia-88065    WAAYRTRKRGRYRYTKKYYAYWMTWTTYYCRGRCGCYCTYGTRGCSYTGGYTWGMGCCAC 17880
                              **  .. *   *   .*       .     .*  .*

Bhutan-09015       GGCRRRWRMYYTARWWRKCKMCAGACYRCGSTKCARCRGSMKGCRRRRGGACGKATCGTC 17940
Bhutan-09024       GGCRRRWRMYYTARWWRKCKMCARACYRCGSTKCARCRGSMKGCRRRRGGACGKATCGTC 17940
Bhutan-09027       GGCRRRWRMYYTARWWRKCKMCAGACYRCGSTKCARCRGSMKGCRRRRGGACGKATCGTC 17940
Bhutan-09030       GGCRRRWRMYYTARWWRKCKMCRGACYRCGSTKCARCRGSMKGCRRRRGGACGKATCGTC 17940
Bhutan-09005       KRSAAGTAMCCKRRWAGGYKMCAATMCRYRSYTSGAGRRSAGSSGGGARRRYRKWKTAYT 17940
Indonesia-88035    KRSAAGTAMCCKRRWAGGYKMYARTMCRYRSYTSGAGRRSAGSSGGGARRRYRKWKTAYT 17940
Indonesia-88045    KRSAAGTAMCCKRRWAGGYKMYARTMCRYRSYTSGAGRRSAGSSGGGARRRYRKWKTAYT 17940
Indonesia-88065    KRSAAGTAMCCKRRWAGGYKMYARTMCRYRSYTSGAGRRSAGSSGGGARRRYRKWKTAYT 17940
                     .     *  . **    **   :  *  * ...  * *  ..         * . .

Bhutan-09015       CARRRAAAKYCRKTTRRGRSYYTAYYYRARWYMRYRMTASACAARAGMGYYGGYKCAYWT 18000
Bhutan-09024       CARRRAAAKYCRKTTRRGRSYYTAYYYRARWYMRYRMTASACAARAGMGYYGGYKCAYWT 18000
Bhutan-09027       CARRRAAAKYCRKTTRRGRSYYTAYYYRARWYMRYRMTASACAARAGMGYYGGYKCAYWT 18000
Bhutan-09030       CARRRAAAKYCRKTTRRGRSYYTAYYYRARWYMRYRMTASACAARAGMGYYGGYKCAYWT 18000
Bhutan-09005       YGARRGRRKCYATYYRRRGGYYARCYTRWAWYMRYAMAMGWSRRARSCTYTRSTTSWTAW 18000
Indonesia-88035    YGARRGRRKCYATYYRRRGGYYARCYTRWAWYMRYAAAMGWSRRARSCTYTRSTTSWTAW 18000
Indonesia-88045    YGARRGRRKCYATYYRRRGGYYARCYTRWAWYMRYAMAMGWSRRAAGCTYTRSTTSWTAW 18000
Indonesia-88065    YGARRGRRKCYATYYRRRGGYYARCYTRWAWYMRYAMAMGWSRRARSCTYTRSTTSWTAW 18000
                    . **.  *   .  **  .**:  * *  *****  : . .    .  *  . ..

Bhutan-09015       AYYRGWMGSAYYAGTYCWMCGWGWRYRYAGCGCARCRMTTYTWWTAYTATMSRAGGRAYT 18060
Bhutan-09024       AYYRGWMGSAYYAGTYCWMCGWGWRYGYAGCGCARCRMTTYTWWTAYTATMSRAGARAYT 18060
Bhutan-09027       AYYRGWMGSAYYAGTYCWMCGWGWRYGCAGCGCARSAMTTYTWWTAYTATMSRAGARAYT 18060
Bhutan-09030       AYYRGWMGSAYYAGTYCWMCGWGWRYRYAGCGCARSRMTTYTWWTAYTATMSRAGGRAYT 18060
Bhutan-09005       RYYASACARMTTWRKTYWAYRTRTRYGCMKYAMTGGACYGYAATYWYCRYCCARAGRRYT 18060
Indonesia-88035    RYYASACAGMTTWRKTYWAYRTRTRYGCMKYAMTGGACYGYAATYWYCGCCCARAGRRYY 18060
Indonesia-88045    RYYASACAGMTTWRKTYWAYRTRTRYGCMKYAMTGGACYGYAATYWYCRYCCARAGRRYY 18060
Indonesia-88065    RYYASACAGMTTWRKTYWAYRTRTRYGCMKYAMTGGACYGYAATYWYCRYCCARAGRRYT 18060
                    ** .  .      .  *      **     . :      *:    *    .  ..* *

Bhutan-09015       YTKSRSAYYTTKWYARCTTYRCCGGRRGRRCYRTCRRKYRRGAYRMKMYWGRTRGGMRCR 18120
Bhutan-09024       CTKSRSAYYTTKWYARCTTYRCCGGAAGRRCYRTCRRKYRRGAYRMKMYWGRTRGGMRCR 18120
Bhutan-09027       CTKSRSAYYTTKWYARCTTYRCCGGRRGRRCYRTCRRKYRRGAYRMKMYWGRTRGGMRCR 18120
Bhutan-09030       CTKSRSAYYTTKWYARCTTYRCCGGGGGRRCYRTCRRKYRRGAYRMKMYWGRTRGGMRCR 18120
Bhutan-09005       MCGCAGWCYWTGWCAAMYCYAYMRRGGRRRSKRYCGATTARSRCGAGCCTAGAGRCAGCG 18120
Indonesia-88035    MYKCAGWCYWYGWCRAMYCYAYMRGGGRRRSKRYCGATYARSRCRMKCYWRGWGRCAGCG 18120
Indonesia-88045    MYKCAGWCYWYGWCRAMYCYAYMRGGGRRRSKRYYGATYARSRCRMKCYWRGWGRCAGYG 18120
Indonesia-88065    MCGCAGWCYWTGWCAAMYCYAYMRGGGRRRSKRYCGATYARSRCRMKCYWRGWGRCAGCG 18120
```

```
                        . .  *   *      *        **. *    .  *.

Bhutan-09015         YMRWAAGGYRCGCTAAAATSACMCTAYYYSYAKGRRRYWYGRRCRYRRRYYTGAYYAYGM 18180
Bhutan-09024         YMRWAAGGYRCGCTAAAATSACMCTAYCTSYAKGRRRYWYGRRCRYRRRYYTGAYYAYGM 18180
Bhutan-09027         YMRWAAGGYRCGCTAAAATSACMCTAYCTSYAKGRRRYTTGRRCRYRRRYYTGAYYAYGM 18180
Bhutan-09030         YMRWAAGGYRCGCTAAAATSACMCTAYCTSYAKGRRRYTTGRRCRYRRRYYTGAYYAYGM 18180
Bhutan-09005         YCRAWRKRYRSSGYRRGGYGGTMYYRCTCCTAKGAGRCWYRAGGACGGACYYKAYTWTAM 18180
Indonesia-88035      YCRAWRKRYRSSGYRRGGYGGTMYYRCTCCTWKRAGRCWYRAGCACGGACYYKWYTWTAM 18180
Indonesia-88045      YCRAWRKRYRSSGYRRGGYGGTMYYRCTCCTAKRAGRCWYRAGCACGGACYYKWYTWTAM 18180
Indonesia-88065      YCRAWRKRYRSSGYRRGGYGGTMYYRCTCCTAKGAGRCWYRAGGACGGACYYKAYTWTAM 18180
                     * *     **..    .. .. *      .  *   *             *   *   .*

Bhutan-09015         TYGARGGARCCGWYRAGACTKACAAGTYTTGTYYTGMRRKRGYYRCYAARACYMYGMWYK 18240
Bhutan-09024         TYGARGGARCCGWYRAGACTKACAAGTYTTGTYYTGMRRKRGYYRCYAARACYMYGMWYK 18240
Bhutan-09027         TYGARGGARCCGWYRAGACTKACAAGTYTTGTYYTGMRRKRGYYRCYAARACYMYGMWYK 18240
Bhutan-09030         TYGARGGARCCGWYRAGACTGACAAGTYTTGTYYTGMRRKRGYYRCYAARACYMYGMWYK 18240
Bhutan-09005         CTSCARRRGAYRAYRRRYACGRTMTCYCCYRCTCYRAAGGGGYYGYTCMRRYTCYRAWYK 18240
Indonesia-88035      CTSCARRRGAYRAYRRRAMCKATAWCYCCTRYYCYRAAGGGSYYGYTCMRRYTCYRAWYK 18240
Indonesia-88045      CTSCARRRGAYRAYRRRAMCKATAWCYCCTRYYCYRAAGGGSYYGYTCMRRYTCYRAWYK 18240
Indonesia-88065      CTSCARRRGAYRAYRRRYACGRTMTCYCCYRCTCYRAAGGGGYYGYTCMRRYTCYRAWYK 18240
                       ..     .   **                          .**   . *    *  ***

Bhutan-09015         KGCAAKCAARTCRKWKMARATRRRWRWAWYTYACRCTTYYYCACRMYWWAYCRKGTSAAG 18300
Bhutan-09024         KGCAAKCAARTCRKWKMARATRRRWRWAWYTYACRCTTYYYCACRMYWWAYCRKGTSAAG 18300
Bhutan-09027         KGCAAKCAARTCRKWKMARATRRRWRWAWYTYACRCTTYYYCACRMYWWAYCRKGTSAAG 18300
Bhutan-09030         KGCAAKCAARTCRKWKMARATRRRWRWAWYTYACRCTTYYYCACRMYWWAYCRKGTSAAG 18300
Bhutan-09005         TKYMGTATTRYYGGRKMARRYAAATRTMACYYCYRSWWYYTYWGGCYATRCTGGRWCRWR 18300
Indonesia-88035      TKYMGTATTRYYGGRKMRRRYAAATRTMACYYMCRSWWTCTYWGGCYATRTCGGRWCRWR 18300
Indonesia-88045      TKYMGTATTRYYGGRKMRRRYAAATRTMACYYMCRSWWTCTYWGGCYATRTCGGRWCRWR 18300
Indonesia-88065      TKYMGTATTRYYGGRKMARRYAAATRTMACYYCYRSWWYYTYWGGCYATRCTGGRWCRWR 18300
                     .   ...::*     ** *      *     *  *.          *         .

Bhutan-09015         KGAGRRARATKYGCMYARYYMSTTYTATTSYGSTAYTTGAAGYRTYYAMGARWSYWWCKR 18360
Bhutan-09024         TGAGRRARATKYGCMYARYYMSTTYTATTSYGSTAYTTGAAGYRTYYAMGARWSYWWCKR 18360
Bhutan-09027         KGAGRRARATKYGCMYARYYMSTTYTATTSYGSTAYTTGAAGYRTYYAMGARWSYWWCKA 18360
Bhutan-09030         KGAGRRARATKYGCMYARYYMSTTYTATTSYGSTAYTTGAAGYRTYYAMGARWSYWWCKR 18360
Bhutan-09005         GRRRGAGARKTYRTMTTRTTASKYYYRCWTCKCYRYYYKWRRYAYYTRCTRGACYWWYKR 18360
Indonesia-88035      GRRRGAGARKTYRTMCTRTTMSKYYYRCWTCKCTRYYYKWRRYRTYTRCTRGACYWWCKR 18360
Indonesia-88045      GRRRGAGARKTYRTMCTRTTMSKYYYRCWTCTCTRYYYKWRRYRTYTRCTRGACYWWCKR 18360
Indonesia-88065      GRRRGAGARKTYRTMTTRTTASKYYYRCWTCKCYRYYYKWRRYAYYTRCTRGACYWWYKR 18360
                           .  ..*  *  :*  *. *    :  .  *      *  *       .*** *

Bhutan-09015         YTKGCTWCRMRKTTYCSKRMMYYGYKYKATWTCTYRCYSKRRTCTAYRTGMRMWYGKGCW 18420
Bhutan-09024         YTKGCTWCRMRKTTYCSKRMMYYGYKYKATWTCTYRCYSKRRTCTAYRTGMRMWYGKGCW 18420
Bhutan-09027         CTKGCTWCRMRKTTYCSKRMMYYGYKYKATWTCTYRCYSKRRTCTAYRTGMRMWYGKGCW 18420
Bhutan-09030         YTKGCTWCRMRKTTYCSKRMMYYGYKYKATWTCTYRCYSKRRTCTAYRTGMRMWYGKGCW 18420
Bhutan-09005         YATRSYTYGMAGWCTSGTAAMYCTYKYKMYTWGYTATTGTGAYYYRCGYSAAMWTRKATT 18420
Indonesia-88035      YTTRSTTYGMAGWCTSGTAAMYCTYKYKMYTWGYTACTGTGATYYRCGYSAAMWTRKATT 18420
Indonesia-88045      YTTRSTTYGMAGWCTSGTAAMYCTYKYKMYTWGYTACTGTGATYYRCGYSAAMWTRKATT 18420
Indonesia-88065      YATRSYTYGMAGWCTSGTAAMYCTYKYKMYTWGYTATTGTGAYYYRCGYSAAMWTRKATT 18420
                      :. .    *     ...  **  ****          ..         .  **  *.

Bhutan-09015         ACYRCKYYCCATGCTGARAGRSYWWGRRTKCRAWYWYYGYTYKRGCTWSAGMARCTGRWC 18480
Bhutan-09024         ACYRCKYYCCATGCTGARAGRSYWWGRRTKCRAWYWYYGYTYKRGCTWSAGMARCTGRWC 18480
```

```
Bhutan-09027            ACYRCKYYCCATGCTGARAGRSYWWGRRTKCRAWYWYYGYTYKRGMTWSAGMARCTGRWC 18480
Bhutan-09030            ACYRCKYYCCATGCTGARAGRSYWWGRRTKCRAWYWYYGYTYKRGMTWSAGMARCTGRWC 18480
Bhutan-09005            RYCGMGCTYGTWRYCARRRAGTATRRGWKSAMWYTTSRCYYKGKMYAGMSMRRAYTGAT 18480
Indonesia-88035         AYCGMGCTCGTWRYYRAARRAGTATRRGWKSAMWYTTSRCTYKGKMYAGMSMRRAYTGAT 18480
Indonesia-88045         AYCGMGCTCGTWRYYRAARRAGTATRRGWKSAMWYTTSRCTYKGKMYAGMSMRRAYTGAT 18480
Indonesia-88065         RCCGMGCTYGTWRYCARRRAGTATRRGWKSAMWYTTSRCTYKGKMYAGMSMRRAYTGAT 18480
                                  :          .    *  *.   **     **     . .* *.

Bhutan-09015            RCYGGYRRWRWYGYCYKAKWSYCGGYATTTGKTCKTGGWYAASRYAWKCYRRWYTAAMRC 18540
Bhutan-09024            RCYGGYRRWRWYGYCYKAKWSYCGGYATTTGKTCKTGGWYAASRYAWKCYRRWYTAAMRC 18540
Bhutan-09027            RCYGGYRRWRWYGYCYKAKWSYCGGYATTTGKTCKTGGWYAASRYAWKCYRRWYTAAMRC 18540
Bhutan-09030            RCYGGYRRWRWYGYCYKAKWSYCGGYATTTGKTCKTGGWYAASRYAWKCYRRWYTAAMRC 18540
Bhutan-09005            AGTACCAAWGATKTYTGCGAGYTARTRWTTCTKYKAARWCRRCRTRWGGCAGTTKRRCRY 18540
Indonesia-88035         AGTGCYAAWGATKTYTGCGAGTTARTAWWYCTKYKAGRWCRRCRTRWGGCAGTTKRRCRY 18540
Indonesia-88045         AGTGCYAAWGATKTYTGCGAGTTARTAWWYCTKYKAGRWCRRCRTRWGGCAGTTKRRCRY 18540
Indonesia-88065         AGTACYAAWGATKTYTGCGAGTTARTATTTCTKYKAARWCRRCRTRWGGCAGTTKRRCRY 18540
                           .    *        .  .  .       .. *:. *   .*  *       .   *

Bhutan-09015            ATRRAARWARKKYSSAWTRTSTGTGYGWGGAGGTYTKARAYAKMATGCYGRWCCGGTACR 18600
Bhutan-09024            ATRRAARWMRKKYSSAWTRTSTGTGYGWGGAGGTYTKARAYAKMATGCYGRWCMGGTACR 18600
Bhutan-09027            ATRRAARWMRKKYSSAWTRTSTGTGYGWGGAGGTYTKARAYAKMATGCYGRWCMGGTACR 18600
Bhutan-09030            ATRRAARWARKKYSSAWTRTSTGTGYGWGGAGGTYTKARAYAKMATGCYGRWCMGGTACR 18600
Bhutan-09005            MYAAWRATCGTGYCCTTTGYCKRYSYGWGATSKYYTGAGRYRKMRYKMYCGWTCAAWMMG 18600
Indonesia-88035         MYAAWAATCGTGYCCTTKGTCKRYSYKWAATSKYYKGMGRYRKMRYGMYCGWTCAAWMMG 18600
Indonesia-88045         MYAAWAATCGTGYCCTTKGTCKRYSYKWAATSKYYKGMGRYRKMRYGMYCGWTCAAWMMG 18600
Indonesia-88065         MYAAWRATCGTGYCCTTTGYCKRYSYGWGATSKYYTGAGRYRKMRYKMYCGWTCAAWMMG 18600
                                  . *..: .  ..  .* *..:.  *.    * **    *  *  ..

Bhutan-09015            RCGGMMCRTAYACTGKMRTGTKYKMWMKKMYSAKARCGTWWGGYYGYRATTYYGKYRTCG 18660
Bhutan-09024            RCGGMMCRTAYACTGKARTGTKYKMWMKKMYSAKARCGTWWGGYYGYRATTYYGKYRTCG 18660
Bhutan-09027            RCGGMMCRTAYAYTGKMRTGTKYKMWMKKMYSAKARCGTWWGGYYGYRATTYYGKYRTCG 18660
Bhutan-09030            RCGGMMCRTAYAYTGKMGTGTKYKMWMKKMYSAKARCGTWWGGYYGYRATTYYGKYRTCG 18660
Bhutan-09005            ACSRAASGCRCWCYRTARTRYKYGCAATYAYCRKWRYAWAAAAWCKTRWYKCTAGCAYCK 18660
Indonesia-88035         AMSRAASGYRYWCYRTARWRYKYGCAATYAYCRKWACATWAAAWCKTRWYKCTAGCAYYK 18660
Indonesia-88045         AMSRAASGYRYWCYRTARWRYKYGCAATYAYCRKWRCATWAAAWCKTRWYKCTAGCAYYK 18660
Indonesia-88065         ACSRAASGCRCWCYRTARTRYKYGCAATYAYCRKWRYAWAAAAWCKTRWYKCTAGCAYCK 18660
                          .   .        .     **    .  *. *   .   ..:   *  .  .

Bhutan-09015            AYRCMMTRMAGTGTAYCACRRAAYRRRYYGATGGTGCSGWYAYRYTYGYTYSAAYKMGWA 18720
Bhutan-09024            AYRCMMTRMAGTGTAYCACRRAAYRRRYYGATGGTGCSGWYAYRYTYGYTYSAAYKMGWA 18720
Bhutan-09027            AYRCMMTRMAGTGTAYCACRRAAYRRRYYGATGGTGCSGWYAYRYTYGYTYSAAYKMGWA 18720
Bhutan-09030            AYRCMMTRMAGTGTAYCACRRAAYRRRYYGATGGTGCSGWYAYRYTYGYTYSAAYKMGWA 18720
Bhutan-09005            KYRGMAYAMRRYCYMYMMSAATRTAAGYTSMYSRARYSRTTRCAYCTRYWTCRWTGASWW 18720
Indonesia-88035         KCRGMAYAMRRYCYMYMMSAATRTAAGYTCCYSRARYSRTTRCAYCTRYWTCRWTGASWW 18720
Indonesia-88045         KCRGMAYAMRRYCYMYMMSAATRTAAGYTCCYSRARYSRTTRCAYCTRYWTCRWTGASWW 18720
Indonesia-88065         KYRGMAYAMRRYCYMYMMSAATRTAAGYTSMYSRARYSRTTRCAYCTRYWTCRWTGASWW 18720
                          * *   *      *  .  :     *    . :  *      *   *  .     .*

Bhutan-09015            AGRAKTGGGCYGCKAMRAMTAYTTGGTCCRAGAWCGYRCGSYCRSKCYTAGYYGCWRRYT 18780
Bhutan-09024            AGRAKTGGGCYGSKAMRAMTAYTTGGTCCRAGAWCGYRCGSYCRSKCYTAGYYGCWRRYT 18780
Bhutan-09027            AGRAKTGGGCYGSKAMRAMTAYTYGGTCCRAGAWCGYRCGSYCRSKCYTAGYYGCWRRYT 18780
Bhutan-09030            AGRAKTGGGCYGCKAMRWMTAYTYGGTCCRAGAWCGYRCGSYCRSKCYTAGYYGCWRRYT 18780
Bhutan-09005            RRRWTKSSKSYRSTMCAAATRYKCRRYYYRGARWMACASRCYTRSGSYCCRYCRMARGTW 18780
Indonesia-88035         RRRWTKSSKSYRSTMCATATRYKCRRYYYRRAGAMACASRCYTRSGSYCCRYCRMARGTW 18780
```

```
Indonesia-88045     RRRWTKSSKSYRSTMCATACRYKCRRYYYRRAGAMACASRCYTRSGSYCCRYCRMARGTW 18780
Indonesia-88065     RRRWTKSSKSYRSTMCAWATRYKCRRYYYRGARWMACASRCYTRSGSYCCRYCRMARGTW 18780
                      * .... .* ..       *.      * .   .  . .* ** .* . *    *

Bhutan-09015        RGKTRCRCYCGWCACWGGSWYAWCGYYTYYAGCCRYAGYTCWRYYYARYARAGACGATMR 18840
Bhutan-09024        RGKTRCRCYCGWCACWGGSWYAWCGYYTYYAGCCRYAGYTCWRYYYARYARAGACGATMR 18840
Bhutan-09027        RGKTRCRCYCGWCACWGGSWYAWCGYYTYYAGCCRYAGYTCWRYYYARYARAGACGATMR 18840
Bhutan-09030        RGKTRCRCYCGWCACWGGSWYAWCGYYTYYAGCCRYAGYTCWRYYYARYARAGACGATMR 18840
Bhutan-09005        AAKYAMRMTYAWYMMWRSGACRTYKYYCYYMATYGYWRTCTWGYYTWATGGGKRSRMWCR 18840
Indonesia-88035     AAKYRCRMTYAWYMMWRSGACRWCKYYCYYMATYGYWRTCTWGYYTWATGGRKRSRMWCR 18840
Indonesia-88045     AAKYRCRMTYAWYMMWRSGACRWCKYYCYYMATYGYWRTCTWGYYTWATGGRKRSRMWCR 18840
Indonesia-88065     AAKYAMRMTYAWYMMWRSGACRTYKYYCYYMATYGYWRTCTWGYYTWATGGAKRSRMWCR 18840
                     .*   *   .*   * ..      ** ** .   *     * **    .    .    *

Bhutan-09015        SKRYTCSYTGGAGAWRMGAYRSTGACGRRYGARRTYWGTKKAYGYYCKRGACARTMWYCA 18900
Bhutan-09024        SKRYTCSYTGGAGAWRMGAYRSTGACGRRYGARRTYWGTKKAYGYYCKRGACARTMWYCA 18900
Bhutan-09027        SKRYTCSYTGGAGAWRMGAYRSTGACGRRYGARRTYWGTKKAYGYYCKRGACARTMWYCA 18900
Bhutan-09030        SKRYTCSYTGGAGAWRMGAYRSTGACGRRYGARRTYWGTKKAYGYYCKRGACARTMWYCA 18900
Bhutan-09005        SGACKCSTTKRRRTAACGWCACYRAYGGACGRAAWTWKKKGWCCYYYKGSRYRAWCWWMT 18900
Indonesia-88035     SGACKMCTCTRRRTAACKWCRCYRMYRGACKRAAWTWKKKGWCCYYYKGSACRAWCWWMT 18900
Indonesia-88045     SGACKMCTCTRRRTAACKWCRCYRMYRGACKRAAWTWKKKGWCCYYYKGSACRAWCWWMT 18900
Indonesia-88065     SGACKCSTTKRRRTAACGWCACYRAYGGACGRAAWTWKKKGWCCYYYKGSRYRAWCWWMT 18900
                    *   . .      :       .              * .*    ** * .      *: :

Bhutan-09015        GTYCGRWWGAGTTGRYYGGYTTTYMSATARKYYRTCGTRKYGYTCACACCCGYRYRAYRT 18960
Bhutan-09024        GTYCGRWWGAGTTGRYYGGTTTTTMSATARKYYRTCGTRKYGYTCACACCCGYRYRAYRT 18960
Bhutan-09027        GTYCGRWWGAGTTGRYYGGYTTTYMSATARKYYRTCGTRKYGYTCACACCCGYRYRAYRT 18960
Bhutan-09030        GTYCGRWWGAGTTGRYYGGYTTTYMSATARKYYRTCGTRKYGYTCACACCCGYRYRAYRT 18960
Bhutan-09005        KCTYGRWTRWTYTRATYGRYYYKYCSRYRATYCRYYRGATCRTKYWSGYYYRTATARYGY 18960
Indonesia-88035     GCTYSRTTRWTYGRATYSRTYYKYCCRCRATYCRYYRTATCRTKYASGYYYRTATARYGY 18960
Indonesia-88045     GCTYSRTTRWTYGRATYSRTYYKYCCRCRATYCRYYRTATCRTKYASGYYYRTATARYGY 18960
Indonesia-88065     KCTYGRWTRWTYTRATYGRYYYKYCSRYRATYCRYYRGATCRTKYWSGYYYRTATARYGY 18960
                        .*          *.    .  .    .* *     .   .  ..         *

Bhutan-09015        YWYKWRKSRCTYMYMYKATTTTACRAYCYKARYCYMWKMWGYYMWTWATRMYGWYWGSYW 19020
Bhutan-09024        YWYKWRKSRCTYMYMYKATTTTACRAYCYKARYCYMWKMWGYYMWTWATRMYGWYWGSYW 19020
Bhutan-09027        YWYKWRKSRCTYMYMYKATTTTACRAYCYKARYCYMWKMWGYYMWTWATRMYGWYWGSYW 19020
Bhutan-09030        YWYKWRKSRCTYMYMYKATTTTACRAYCYKARYCYMWKMWGYYMWTWATRMYGWYWGSYW 19020
Bhutan-09005        TWCTWRGGRYYYAYMCKRWYKKRMAWCTTTMGYYTMTGATKTCCTWWWKAMYRAYWASYA 19020
Indonesia-88035     TWCTWRGGRYYYAYMCKRWYKKRMAWCTTTMGYYTMTGATKTCCTWWWKAMYAAYWASYA 19020
Indonesia-88045     TWCTWRGGRYYYAYMCKRWYKKRMAWCTTTMGYYTMTGATKTCCTWWWKAMYRAYWASYA 19020
Indonesia-88065     TWCTWRGGRYYYAYMCKRWYKKRMAWCTTTMGYYTMTGATKTCCTWWWKAMYRAYWASYA 19020
                     * .** .*  * ** *   ..       .  *  *          * . **  **.**

Bhutan-09015        SRSCWYSTKRARTCGRTTYRATSRWYRACTYSCAKMSSTGATGWTRWATTYWTARKARRA 19080
Bhutan-09024        SRSCWYSYKRARTCGRTTYRATSRWYRACTYSCAKMSSTGATGWTRWATTYWTARKARRA 19080
Bhutan-09027        SRSCWYSYKRARTCGRTTYRATSRWYRACTYSCAKMSSTGATGWTRWATTYWTARKARRA 19080
Bhutan-09030        SRSCWYSTTRARTCGRTTYRATSRWYRACTYSCAKMSSTGATGWTRWATTYWTARKARRA 19080
Bhutan-09005        CAGSTTCYGARACYRAGYYRMKMGWYGWYKCGYRTMSKYSRWRWWAARGWTTYWRTRAAR 19080
Indonesia-88035     CAGSTTCYGARACYRAGYYRMKMGAYGWYKCGYRTMSKYSRWRWWAARGWTTYWRTRAAR 19080
Indonesia-88045     CAGSTTCYGARACYRAGYYRMKMGAYGWYKCGYRTMSKYSRWRWWAARGWTTYWRTRAAR 19080
Indonesia-88065     CAGSTTCYGARACYRAGYYRMKMGWYGWYKCGYRTMSKYGATGWWAARGWTTYWRTRAAR 19080
                    . ..  .           ** .   *   . .  .**. .   *          *.
```

```
Bhutan-09015         YRRMYCRYYRTCGGMWRAKWGRWRKKCYGCCRRGYYRKWRKKCWAMGCTGRGRAAATRRA 19140
Bhutan-09024         YRRMYCRYYRTCGGMWRAKWGRWRKKCYGCCRRGYYRKWRKKCWAMGCTGRGRAAATRRA 19140
Bhutan-09027         YRRMYCRYYRTCGGMWRAKWGRWRKKCYGCCRRGYYRKWRKKCWAMGCTGRGRAAATRRA 19140
Bhutan-09030         YRRMYCRYYRTCGGMWRAKWGRWRKKCYGCCRRGYYRKWRKKCWAMGCTGRGRAAATRRA 19140
Bhutan-09005         TAACTYAYCRKYRRAWRRKACRWGKKTCRTTRGATTGTAATKAWWMRTYRGRAGRATGRR 19140
Indonesia-88035      TAACTYAYCRKYRRAWRRKACRWGKKTCRTTRGATTGTAATKAWWMRTYRGRAGRATGRR 19140
Indonesia-88045      TAACTYAYCRKYRRAWRRKACRWGKKTCRTTRGATTGTAATKAWWMRTYRGRAGRRKGRR 19140
Indonesia-88065      TAACTYAYCRKYRRAWRRKACRWGKKTCRTTRGATTGTAATKAWWMRTYRGRAGRRKGRR 19140
                            * *.    ** *  ** **     * .   . .*.* *       .  . *

Bhutan-09015         CWYYGSSAYRGRKGKAYGWTTKTCCRAYYKTYYCRGTRTRAYTRRRCWACGGRACRCRYY 19200
Bhutan-09024         CWYYGSSAYRGRKGKAYGWTTKTCCRAYYKTYYCRGTRTRAYTRRRCWACGGRACRCRYY 19200
Bhutan-09027         CWYYGSSAYRGRKGKAYGWTTKTCCRAYYKTYYCRGTRTRAYTRRRCWACGGRACRCRYY 19200
Bhutan-09030         CWYYGSSAYRGRKGKAYGWTTKTCCRAYYKTYYCRGTRTRAYTRRRCWACGGRACRCRYY 19200
Bhutan-09005         TACTTCCRCAGAGAGGTRAYYTCGYAWTTTAYTYASYGCGRTYGGACATSRKAGCRTGCT 19200
Indonesia-88035      TAYYTSSGCAKAGAGGTAATTTCGYRATTTTYTYASCGCGRTYGGAYATSRKAGSRTGCT 19200
Indonesia-88045      TAYYTSSGCAKAGAGGTAATTTCGYRATTTTCTYASCGCGRTYGGAYATSRKAGSRTGCT 19200
Indonesia-88065      TACTTCCGCAGAGAGGTRAYYTCGYAWTTTAYTYASYGCGRTYGGACATSRKAGCRTGCT 19200
                          ..      . .     .       .:    .            :.   ..*

Bhutan-09015         WRRKARAYYCGTTSCCYGATMGAASRYAYARRCAMYMCTKTSTCRAGAAWMTSMSARYSA 19260
Bhutan-09024         WGRKARAYYCGTTSCCYGATMGAASRYAYARRCAMYMCTKTSTCRAGAAWMTSMSARYSA 19260
Bhutan-09027         WGRKARAYYCGTTSCCYGATMGAASRYAYARRCAMYMCTKTSTCRAGAAWMTSMSARYSA 19260
Bhutan-09030         WARKARAYYCGTTSCCYGATMGAASRYAYARRCAMYMCTKTSTCRAGAAWMTSMSARYSA 19260
Bhutan-09005         WGAKWAWCCYSWYSTSYARKATRWGACATRRRSMCCAMYKWCYAGRRWATCYCACRGTCW 19260
Indonesia-88035      WGAKWAWCCYSWYSTSYARKATRWGRYWTRRRSMCCAMYKWCTMGARTWTCYCACRGTCW 19260
Indonesia-88045      WGAKWAWCCYSWYSTSYARKATRWGRYWTRRRSMCCAMYKWCTMGARTWTCYCACRGTCW 19260
Indonesia-88065      WGAKWAWCCYSWYSTSYARKATRWGACATRRRSMCCAMYKWCYAGRRWATCYCACRGTCW 19260
                     *  *      .  * .*. .    .     **.      * .          . .   .

Bhutan-09015         KSGAWRCGGTRCGRRAYTCAGRGGCMRRGAARRYAATWCWCSKWRWRYKYYRYYYKRKGC 19320
Bhutan-09024         KSGAWRCGGTRCGRRAYTCAGRGGCMAAGAARRYAATWCWCSKWRWRYKYYRYYYKRKGC 19320
Bhutan-09027         KSGAWRCGGTRCGRRAYTCAGRGGCMRRGAARRYAATWCWCSKWRWRYKYYRYYYKRKGC 19320
Bhutan-09030         KSGAWRCGGTRCGRRAYTCAGRGGCMRRGAARRYAATWCWCSKWRWRYKYYRYYYKRKGC 19320
Bhutan-09005         KSRGAGYKRKRTRAARTYYRARSSYCAAKWWARCRMYTMWSCTTATGYKTTGCYYTAGRM 19320
Indonesia-88035      KSRGAGYKRKRTRAARTYYRARSSYCAAKWWARCRMYTMWSCTTATGYKTTGCYYTAGRM 19320
Indonesia-88045      KSRGAGYKRKRTRAARTYYRARSSYCAAKWWARCRMYTMWSCTTATGYKTTGCYYTAGRM 19320
Indonesia-88065      KSRGAGYKRKRTRAARTYYRARSSYCAAKWWARCRMYTMWSCTTATGYKTTGCYYTAGRM 19320
                     ** .     .*         .*..        *      *...    **    **.

Bhutan-09015         YYCSYRSTTCMTCRCSCYYGSSYRCWTAYKAYWCSTGTYCGGCTTASYTSAMSGGATACA 19380
Bhutan-09024         YYCSYRSTTCMTCRCSCYYGSSYRCWTAYKAYWCSTGTYCGGCTTASYTSAMSGGATACA 19380
Bhutan-09027         YYCSYRSTTCMTCRCSCYYGSSYRCWTAYKAYWCSTGTYCGGCTTASYTSAMSGGATACA 19380
Bhutan-09030         YYCSYRSTTCMTCRCSCYYGSSYRCWTAYKAYWCSTGTYCGGCTTASYTSAMSGGATACA 19380
Bhutan-09005         CYASYRGYTSCYYGMSSYCRSCYRTWARCTWCWYSWKKYSRRSKAMSYCSWMGCATGWGG 19380
Indonesia-88035      CYASYRGYKSCYYGMSSYCRSCYRTWARCTWCWYSWKKYSRRSKACSYCGWMGCATGWGG 19380
Indonesia-88045      CYASYRGYKSCYYGMSSYCRSCYRTTARCTWCWYSWKKYSRRSKACSYCGWMGCATGWGG 19380
Indonesia-88065      CYASYRGYTSCYYGMSSYCRSCYRTWARCTWCWYSWKKYSRRSKWMSYCSWMGCATGWGG 19380
                      *.***. ..     *.*  *.**  :  .  * *  .*.  ..  ** . *. .:   .

Bhutan-09015         RGGTYWYTATCATAATSRYRYRYARSGAMYKAWRAWKTKWCKWAYARCWSKMTGKTYRRR 19440
Bhutan-09024         RGGTYWYTATCATAATGATRYRYARSGAMYKAWRAWKTKWCKWAYARCWSKMTGKTYRRR 19440
Bhutan-09027         RGGTYWYTATCATAATSRYRYRYARSGAMYKAWRAWKTKWCKWAYARCWSKMTGKTYRRR 19440
Bhutan-09030         RGGTYWYTATCATAATSRYRYRYARSGAMYKAWRAWKTKWCKWAYARCWSKMTGKTYRRR 19440
```

```
Bhutan-09005        GRAGTAYYRAMGAWMCGATGCACGRSKRMCTRWRWTKYKWCGWMYRGYTRKACAKYYRRG 19440
Indonesia-88035     GRAGTAYYGAAGATMCGATGCACGRSKRMCTRWRWTKYKWCGWMYRGYTRKACAKYYRRG 19440
Indonesia-88045     GRAGTAYYRAMGATMCGATGCACGRSKRMCTRWRWTKYKWYGWMYRGYTRKACAKYYRRG 19440
Indonesia-88065     GRAGTAYYRAMGAWMCGATGCACGRSKRMCTRWRWTKYKWCGWMYRGYTRKACAKYYRRG 19440
                      .   *  : .:   .      .**  * . **  * **  * *     *  .* ***

Bhutan-09015        GAGRYYRWGYAYCKGAYTACYKRSACAYCCGATYCCCKYACRCTTGSCGKMWCYTYYYCA 19500
Bhutan-09024        GAGRYYRWGYAYCKGAYTACYKRSACAYCCGATYCCCKYACRCTTGSCGKMWCYTYYYCA 19500
Bhutan-09027        GAGRYYRWGYAYCKGAYTACYKRSACAYCCGATYCCCKYACRCTTGSCGKMWCYTYYYCA 19500
Bhutan-09030        GAGRYYRWGYAYCKGAYTACYKRSACAYCCGATYCCCKYACRCTTGSCGKMWCYTYYYCA 19500
Bhutan-09005        SWRRCKAAATRYSTRCTGWYTTAGCYRYAMGTYTSGGKYRSAYWYSGYRTAASTWCTTAR 19500
Indonesia-88035     SWRRCKAAATRYSTACTGWYTTAGCYRYCMKTYTSGGKYRSAYTYSGYRTAASTWCTTAR 19500
Indonesia-88045     SWRRCKAAATRYSTRCTGWYTTAGCYRYCMKTYTSGGKYRSAYTYSGYRTAASTWCTTAR 19500
Indonesia-88065     SWRRCKAAATRYSTRCTGWYTTAGCYRYAMGTYTSGGKYRSAYWYSGYRTAASTWCTTAR 19500
                    .  *    .  *.. .     . ..  *.  :  .  ** .    ..  .  .     .

Bhutan-09015        CGGRRTGCGTGRCSSRYTWCCGMRTYTWRYAYATARYKKCCARTTSACSRAGWGGYTCYM 19560
Bhutan-09024        CGGRRTGCGTGRCSSRYTWCCGMRTYTWRYAYATARYKKCCARTTSACSRAGWGGYTCYM 19560
Bhutan-09027        CGGRRTGCGTGRCSSRYTWCCGMRTYTWRYAYATARYKKCCARTTSACSRAGWGGYTCYM 19560
Bhutan-09030        CGGRRTGCGTGRCSSRYTWCCGMRTYTWRYAYATARYKKCCARTTSACSRAGWGGYTCYM 19560
Bhutan-09005        YRTAGKRARYRGYGGRYCAMYGCGYCTWGCMCWARAYKKAYRGTWSRYGARRTTAYYYTA 19560
Indonesia-88035     CGTAGKRARYRGYGGRYCACYRCGTCWTGCMCWAAAYKKATGGYWSRYGARRTTAYYYTA 19560
Indonesia-88045     CGTAGKRARYRGYGGRYCACYRCGTCWTGCMCAAAAYKKATGGYWSRTGARRTTAYYYTA 19560
Indonesia-88065     YRTAGKRARYRGYGGRYCAMYGCGYCTWGCMCAARAYKKAYRGTWSRYGARRTTAYYYTA 19560
                         . .     ..**                :  ***.     *  .     .*

Bhutan-09015        MGGATTTCKARRRYTYYRGSYAAGYAGKGATYYWCCTGTKCYAARYCRRCYYMYCYWGGY 19620
Bhutan-09024        MGGATTTCKARAAYTYYRGSYAAGYAGKGATYYWCCTGTKCYAARYCRRCYYMTCYWRGY 19620
Bhutan-09027        MGGATTTCKARRRYTYYRGSYAAGYAGKGATYYWCCTGTKCYAARYCRRCYYMYCYWRGY 19620
Bhutan-09030        MGGATTTCKARRRYTYYRGSYAAGYAGKGATYYWCCTGTKCYAARYCRRCYYMYCYWRGY 19620
Bhutan-09005        CKKGTGWTKRATGCYTTRASYWRRTMAGGGWTTTMMYRYKMCWRATYGGYCYAYGCTGGT 19620
Indonesia-88035     MKGGTKTTKRAGGCYTTRASYARRTAAKGGTTTTMMYRYKMCAAATYGGYCYAYGCTGAT 19620
Indonesia-88045     MKGGTKTTKRAGGCYTTRASYARRTAAKSGTTTTMMYRYKMCAAATYGGYCYAYGCTGRT 19620
Indonesia-88065     CKKGWKWTKRATGCYTTRASYARRTMAGGGWTTTMAYRYTACWRATYGGYCYAYGCTGGT 19620
                       .    *        *.**     . ..         .           *

Bhutan-09015        AYRGTRMRTGSGGGCWCGTRRYYYRGGTGGAGAMARYRAGRYAKTYYYRTMMSMGYGWYY 19680
Bhutan-09024        AYRGTRMRTGSGGGCWCGTRRYYYRGGTGGAGAMARYRAGRYAKTYYYRTMMSMGYGWYY 19680
Bhutan-09027        AYRGTRMRTGSGGGCWCGTRRYYYRGGTGGAGAMARYRAGRYAKTYYYRTMMSMGYGWYY 19680
Bhutan-09030        AYRGTRMRTGSGGGCWCGTRRYYYRGGTGGAGAMARYRAGRYAKTYYYRTMMSMGYGWYY 19680
Bhutan-09005        TCRTYAMGYRCKSAMTYRAAATCYRARWCSRRRMRATARRGYAGKTTCAYCMSMRCATCC 19680
Indonesia-88035     ACRTYAMGYRCKSAMTYRAAATCYRARWCCRRRMRATARRGYGGGTTCAYMMGMRCATCC 19680
Indonesia-88045     ACRTYAMGYRCKSAMTYRAAATCYRARWCSRRRMRATARRGYGGGTTCAYMMGMRCATCC 19680
Indonesia-88065     TCRTYAMGYRCKSAMTYAAAATCYRARWCSRRRMRATARRGYAGKTTCAYCMSMRCATCC 19680
                    : *   *   . ..    :    **.       *       *.        *.*  .

Bhutan-09015        GRGYYGCAKMTYGRRRASMGTYKMYYKWAGGSCGCYYATAGTAWSSTMYRWAWYWACGWW 19740
Bhutan-09024        GRGYYGCAKMTYGRRRASMGTYKMYYKWAGGSCGCYYATAGTAWSSTMYRWAWYWACGWW 19740
Bhutan-09027        GRGYYGCAKMTYGRRRASMGTYKMYYKWAGGSCGCYYATAGTAWSSTMYRWAWYWACGWW 19740
Bhutan-09030        GRGYYGCAKMTYGRRRASMGTYKMYYKWAGGSCGCYYATAGTAWSSTMYRWAWYWACGWW 19740
Bhutan-09005        KASCTCARGCCYTGGGGSCRWCKCTYKWGTCCATTCTGYWCYMASSYCYGWRTYWRMKTW 19740
Indonesia-88035     KASCTCARGCCYTGGGGSCRWCKCTYKWGTCCATTCTGYWCTAWSSTMYGWRTYWRMKTT 19740
Indonesia-88045     KASCTCARGCCYTGGGGSCRWCKCTYKWGTCCATTCTGYWCTAWSSTMYGWRTYWRMKTT 19740
Indonesia-88065     KASCTCARGCCYTGGGGSCRWCKCTYKWGTCCATTCTGYWCYMASSYCYGWRTYWRMKTW 19740
```

```
                          .   .     *     .*     *  ***.  ..     .       **  * *  **

Bhutan-09015         RSSRAYTRCYGRSAWYAYCATYMYARRKWGYYRACTYMRACYYTYRTWAWWRRYWTAYCG 19800
Bhutan-09024         RSSRAYTRCYGRSAWYAYCATYMYARRKWGYYRACTYMRACYYTYRTWAWWRRYWTAYCG 19800
Bhutan-09027         RSSRAYTRCYGRSAWYAYCATYMYARRKWGYYRACTYMRACYYTYRTWAWWRRYWTAYCG 19800
Bhutan-09030         RSSRAYTRCYGRSAWYAYCATYMYARRKWGYYRACTYMRACYYTYRTWAWWRRYWTAYCG 19800
Bhutan-09005         ASGGGTCAMTRRCWTCRYTGYTMYRRAGWATYRRMYCSRGGTTWCGWTMTTAACACWTYS 19800
Indonesia-88035      ASGGGTCAMTRACWTCRYTGYTMYRRAGWATYRRMYCSRGGTTWCGWTMTTAACACWTYS 19800
Indonesia-88045      ASGGGTCAMTRRCWTCRYTGYTMYRRAGWATYRRMYCSRGGTTWCGWTMTTAACACWTYS 19800
Indonesia-88065      ASGGGTCAMTRRCWTCRYTGYTMYRRAGWATYRRMYCSRGGTTWCGWTMTTAACACWTYS 19800
                      *. .       .     * .  ** *  *. **     *.                   .

Bhutan-09015         WRWTRGMKMRYGKGAKMKTRYGWGRTYYSGTRKWYAMTKGYYAGARTKAYMRGKYYYSRG 19860
Bhutan-09024         WRWTRGMKMRYGKGAKMKTRYGWGRYYYSGTRKWYAMTKGYYAGARTKAYMRGKYYYSRG 19860
Bhutan-09027         WRWTRGMKMRYGKGAKMKTRYGWGATYYSGTRKWYAMTKGYYAGARTKAYMRGKYYYSRG 19860
Bhutan-09030         WRWTRGMKMRYGKGAKMKTRYGWGATYYSGTRKWYAMTKGYYAGARTKAYMRGKYYYSRG 19860
Bhutan-09005         AGWYGRAKAAYKKTGKATYRCKWRATYYCSWRKWCRAYGSTTRRTRCKRCMARKYTYCGR 19860
Indonesia-88035      AGWYGRAKAAYKKTGKATYRCKWRRYYYCSWRKWCRAYGSTTRRTRCKRCMARKYTYCGR 19860
Indonesia-88045      AGWYGRAKAAYKKTGKATYRCKWRRYYYCSWRKWCRAYGSTTRRTRCKRCMARKYTYCGR 19860
Indonesia-88065      AGWYGRAKAAYKKTGKATYRCKWRRYYYCSWRKWCRAYGSTTRRTRCKRCMARKYTYCGR 19860
                       *    *  * * .* . *  *   **.. ***      .    :* *  *  ** *.

Bhutan-09015         RRRMCYWTSSGKTYYMCGATSRCWRMGSYYSTYMAYCRAGKGWYYTGAGKRARARRSGTW 19920
Bhutan-09024         RRRMCYWTSSGKTYYMCGATSRCWRMGSYYSTYMAYCRAGKGWYYTGAGKRARARRSGTW 19920
Bhutan-09027         RRRMCYWTSSGKTYYMCGATSRCWRMGSYYSTYMAYCRAGKGWYYTGAGKRARARRSGTW 19920
Bhutan-09030         RRRMCYWTSSGKTYYMCGATSRCWRMGSYYSTYMAYCRAGKGWYYTGAGKRARARRSGTW 19920
Bhutan-09005         ARRAMYAYGCGGTYCAYARYGATTRARGCCGYTCRYYAWSGAWCCWSWAGARRRGGSKKT 19920
Indonesia-88035      ARRAMYAYGCKGYYCAYARYGATTRARGCCGYTCRYYAWSGAWCCWSWAGARRRGGSKKT 19920
Indonesia-88045      ARRAMYAYGCKGYYCAYARYGATTRARGCCGYTCRYYAWSGAWCCWSWAGARRRGGSKKT 19920
Indonesia-88065      ARRAMYAYGCGGTYCAYARYGATTRARGCCGYTCRYYAWSGAWCCWSWAGARRRGGSKKT 19920
                      **  *  ..   *    .  .    *  .  .    *   . .*   . .   *   * .

Bhutan-09015         GYMYMAKKARGRKYCTKRRGARYCYCGARYRTAAKCTSRASCRSAWTYTTMRRACAWGCR 19980
Bhutan-09024         GYMYMAKKARGRKYCTKRRGARYCYCGARYRTAAKCTSRASCRSAWTYTTMRRACAWGCR 19980
Bhutan-09027         GYMYMAKKARGRKYCTKRRGARYCYCGARYRTAAKCTSRASCRSAWTYTTMRRACAWGCR 19980
Bhutan-09030         GYMYMAKKARGRKYCTKRRGARYCYCGARYRTAAKCTSRASCRSAWTYTTMRRACAWGCR 19980
Bhutan-09005         RYMYMTTKRASGKTYWTAGACGTYTMAMACRKRRTYWCAMSMASRTYTYYAGAGAWWRSA 19980
Indonesia-88035      RYMYMTTKRASGKTYWTAGACGTYTMAMACRKRRTYWCAASMASRTYTYYAGAGAWWRSA 19980
Indonesia-88045      RYMYMTTKRASGKTYWTAGACGTYTMAMACRKRRTYWCAMSMASRTYTYYAGAGAWWRSA 19980
Indonesia-88065      RYMYMTTKRASGKTYWTAGACGTYTMAMACRKRRTYWCAMSMASRTYTYYAGAGAWWRSA 19980
                      ****:.*  . *   .  ..     .   *.  .  .  *  *         .. * .

Bhutan-09015         AYRYTCYWRYKTCAGRRAWRRTASTGWWSTAWYWYRCYYMSSCTTMARYRYTGMYRYCYY 20040
Bhutan-09024         AYRYTCYWRYKTCAGRRAWRRTASTGWWSTAWYWYRCYYMSSCTTMARYRYTGMYRYCYY 20040
Bhutan-09027         AYRYTCYWRYKTCAGRRAWRRTASTGWWSTAWYWYRCYYMSSCTTMARYRYTGMYRYCYY 20040
Bhutan-09030         AYRYTCYWRYKTCAGRRAWRRTASTGWWSTAWYWYRCYYMSSCTTMARYRYTGMYRYCYY 20040
Bhutan-09005         RCGYWTTAACGYMRSGGMAGGYMCKRWTSYWACACGSTCMCCTKYMGGYRYYAATAYCYY 20040
Indonesia-88035      RCGYWTTAACGYMRSGGMAGGYACKRWTSYWACACGSTCMCCTKYMGGYRYYAATAYYYY 20040
Indonesia-88045      RCGYWTTAACGYMRSGGMAGGYACKRWTSYWACACGSTCMCCTKYMGGYRYYAATAYYYY 20040
Indonesia-88065      RCGYWTTAACGYMRSGGMAGGYMCKRWTSYWACACGSTCMCCTKYMGGYRYYAATAYYYY 20040
                        *          .        .. * *       .  *.. . *. *** .   * **

Bhutan-09015         RTMCGGYTKRWRGKGGARWMMYRRTAYCCKYGCCATYKRYCRCWYWYGYSYRSRRGRGWR 20100
Bhutan-09024         RTMCGGYTKRWRGKGGARWMMYRRTAYCCKYGCCATYKRYCRCWYWYGYSYRSRRGRGWR 20100
```

```
Bhutan-09027        RTMCGGYTKRWRGKGGARWMMYRRTAYCCKYGCCATYKRYCRCWYWYGYSYRSRRGRGWR 20100
Bhutan-09030        RTMCGGYTKRWRGKGGARWMMYRRTAYCCKYGCCATYKRYCRCWYWYGYSYRSRRGRGWR 20100
Bhutan-09005        RYMMSKTYTAARKTSKTATACYARKGTYYGTTTATATGGTYATTTTCSCCYRSRGRRSAA 20100
Indonesia-88035     RYMMCKTYTAARKTSKTATACYARKGTYCGTTTATATGGTCATTTTCSCCYRSRGRASAA 20100
Indonesia-88045     RYMMCKTYTAARKTSKTATACYARKGTYCGTTTATATGGTYATTTTCSCCYRSRGRRSAA 20100
Indonesia-88065     RYMMSKTYTAARKTSKTATACYARKGTYYGTTTATATGGTCATTTTCSCCYRSRGRRSAA 20100
                    * *     .  * .. :    * *..       .::           . .****   .

Bhutan-09015        CTAACAMKRSYMYMWRMTTRGYWTCGRSRGAKCACYTSRGYRGARCCSYTCGRYTCYWMT 20160
Bhutan-09024        CTAACAMKRSYMYMWRMTTRGYWTCGRSRGAKCACYTSRGYRGARCCSYYCGRYTCYWMT 20160
Bhutan-09027        CTAACAMKRSYMYMWRMTTRGYWTCGRSRGAKCACYTSRGYRGARCCSYYCGRYTCYWMT 20160
Bhutan-09030        CTAACAMKRSYMYMWRMTTRGYWTCGRSRGAKCACYTSRGYRGARCCSYYCGRYTCYWMT 20160
Bhutan-09005        MCGRMTAGAGYCTMTGMYARRCAYMAGCAAGTMGYYAGASTGRRGMYCTYCARTKMYWCY 20160
Indonesia-88035     MCGRMTAGAGYCTMTGMYARRCAYMAGCAAGTMGYYAGASTGRAGMYCTYMRRCKMYWCY 20160
Indonesia-88045     MCGRMTAGAGYCTMTGMTARRCAYMAGCAAGTMGYYAGASTGRRGMYCTYMRRCKMYWCY 20160
Indonesia-88065     MCGRMTAGAGYCTMTGMYARRCAYMAGCAAGTMGYYAGASTGRAGMYCTYCARTKMYWCY 20160
                      .  :   .*  *  * :*     . . ... . *:. .       .    * . **

Bhutan-09015        CKRARSTGTRGGCYAMTRGYRGWKTARAYRRGCSTARMRTACCRSTAAACYWWMRSRCYR 20220
Bhutan-09024        CKRARSTGTRGGCYAMTRGYRGWKTARAYRRGCSTARMRTACCRSTAAACYWWMRSRCYR 20220
Bhutan-09027        CKRARSTGTRGGCYAMTRGYRGWKTARAYRRGCSTARMRTACCRSTAAACYWWMRSRCYG 20220
Bhutan-09030        CKRARSTGTRGGCYAMTRGYRGWKTARAYRRGCSTARMRTACCRSTAAACYWWMRSRCYG 20220
Bhutan-09005        MGGCGSCGGGGRATMCKAKCARWTYRGAYRRGTGKMAMAYMYMGSWRMMCTTTAACGATG 20220
Indonesia-88035     MGGCGSYKGGKRATMCTAKCARWTYRGMYRRATGKAAMAYMYMGSWRMMSTTTAACGATR 20220
Indonesia-88045     MGGCGSYKGGKRATMCTAKCARWTYRGMYRRATGKAAMAYMYMGSWRMMSTTTAACGATR 20220
Indonesia-88065     MGGCGSCGGGGRATMCKAKCARWTYRGAYRRGTGKMAMAYMYMGSWRMMCTTTAACGATR 20220
                       . *      .   .     *.    ***. ..  *      *    .     . .

Bhutan-09015        AAYTWAGSKYRYRCWSYATATSRRGYYYAKYRAYMGCAWATRRYATKAKCACKCCACARR 20280
Bhutan-09024        AAYTWAGSKYRYRCWSYATATSRRGYYYAKYRAYMGCAWATRRYATKAKCACKCCACARR 20280
Bhutan-09027        AAYTWAGSKYRYRCWSYATATSRRGYYYAKYRAYMGCAWATRRYATKAKCACKCCACARR 20280
Bhutan-09030        AAYTWAGSKYRYRCWSYATATSRRGYYYAKYRAYMGCAWATRRYATKAKCACKCCACARR 20280
Bhutan-09005        RWTKWRKSTCRYRSAGYRWRYCGRRYTYRKYGTTMTYRTTWARTWCTMGTMMTSYRYRRR 20280
Indonesia-88035     RWTKWRKSTCRYRSAGYRWRYCGRRYTYRKYGTTMTYRTTWARTWCTMGTMMTSYRYRRR 20280
Indonesia-88045     RWTKWRKSTCRYRSAGYRWRYCGRRYTYRKYGTTMTYRTTWARTWCTMGTMMTSYRYRRR 20280
Indonesia-88065     RWTKWRKSTCRYRSAGYRWRYCGRRYTYRKYGTTMTYRTTWARTWCTMGTMMTSYRYRRR 20280
                       .*  *. ***. .*    . * * * ** : *    :  *   .     ..    **

Bhutan-09015        CTMAGGTRCAKWMTGTGTTYWRYYSSCYRAYMCTGRSKKRTYRRARYYTAWGRYMAKRTT 20340
Bhutan-09024        CTMAGGTRCAKWMTGTGTTYWRYYSSCYRAYMCTGRSKKRTYRRARYYTAWGRYMAKRTT 20340
Bhutan-09027        CTMAGGTRCAKWMTGTGTTYWRYYSSCYRAYMCTGRSKKRTYRRARYYTAWGRYMAKRTT 20340
Bhutan-09030        CTMAGGTRCAKWMTGTGTTYWRYYSSCYRAYMCTGRSKKRTYRRARYYTAWGRYMAKRTT 20340
Bhutan-09005        SYMRRWGGYMTWAYRYKYWCWRYYSSSYGRYMSYRGSTKRATAGRRYTYWARGCCWKRWW 20340
Indonesia-88035     SYMRRWGGYMTWAYRYKYWCARCCCSSYGATCCTGGCTTRATAGGATTTAAGGCCAGGTT 20340
Indonesia-88045     SYMRRWGGYMTWAYRYKYWCARCCCSSYGATCCTGGCTTRATAGGATTTAAGGCCAGGTT 20340
Indonesia-88065     SYMRRWGGYMTWAYRYKYWCWRYYSSSYGRYMSYRGSTKRATAGRRYTYWARGCCWKRWW 20340
                    . *       .*         *  .*.*    .   ...*:

Bhutan-09015        AARTTYYTCAMYCKCGRCRMKYKWRSRRKRYAARWRSATTYTRRMGMTYTRYGTRYAYCT 20400
Bhutan-09024        AARTTYYTCAMYCKCGRCRMKYKWRSRRKRYAARWRSATTYTRRMGMTYTRYGTRYAYCT 20400
Bhutan-09027        AARTTYYTCAMYCKCGRCRMKYKWRSRRKRYAARWRSATTYTRRMGMTYTRYGTRYAYCT 20400
Bhutan-09030        AARTTYYTCAMYCKCGRCRMKYKWRSRRKRYAARWRSATTYTRRMGMTYTRYGTRYAYCT 20400
Bhutan-09005        TWRYWYTATRACMGMRAYGCTCGARGAATGTRGGAGSRCCCYARCKCYYYGYRKACRTMY 20400
Indonesia-88035     TWRYWYTATRACMGMRAYGCTCGARGAATGTRGRWRSRCCTYARCKCYYYGYRKACRTMY 20400
```

```
Indonesia-88045       TWRYWYTATRACMGMRAYGCTCGARGAATGTRGRWRSRCCTYARCKCYYYGYRKACRTMY 20400
Indonesia-88065       TWRYWYTATRACMGMRAYGCTCGARGAATGTRGGAGSRCCCYARCKCYYYGYRKACRTMY 20400
                      : *  * :            .   *.  .   .   *      *    *  * .

Bhutan-09015          GRAKRASRGYTMAYRYWAGRRTGTCASRRRYGKCTGYTTAGYTGMRYRWRTCATGRYTAR 20460
Bhutan-09024          GRAKRASRGYTMAYRYWAGRRTGTCASRRRYGKCTGYTTAGYTGMRYRWRTCATGRYTAR 20460
Bhutan-09027          GRAKRAGRGYTMAYRYWAGRRTGTCASRRRYGKCTGYTTAGYTGMRYRWRTCATGRYTAR 20460
Bhutan-09030          GRAKRASRGYTMAYRYWAGRRTGTCASRRRYGKCTGYTTAGYTGMRYRWRTCATGRYTAR 20460
Bhutan-09005          RARTRTCGSCYMWTATWGAGAWRYYRGAGATRTAYKTYGGKTYRCACGAACYRKRGTTRR 20460
Indonesia-88035       RARTRTCGSCYMWTATWGAGAWRYYRGAGATGTCYKTYGGKTYRCACAAACYRKRGTCRR 20460
Indonesia-88045       RARTRTCGSCYMWTATWGAGAWRYYRGAGATGTCYKTYGGKTYRCACAAACYRKRGTCRR 20460
Indonesia-88065       RARTRTCGSCYMWTATWGAGAWRYYRGAGATRTAYKTYGGKTYRCACGAACYRKRGTTRR 20460
                         .*:  .  *    *..       .     ..     .             .     *

Bhutan-09015          YKWGGAARYWYSTKMRKATMWCKWCRRGRWKRARGTSYYAYGYTRYTYAAATTACYCACA 20520
Bhutan-09024          YKWGGAARYWYSTKMRKATMWCKWCRRGRWKRARGTSYYAYGYTRYTYAAATTACYCACA 20520
Bhutan-09027          YKWGGAARYWYSTKMRKATMWCKWCRRGRWKRARGTSYYAYGYTRYTYAAATTACYCACA 20520
Bhutan-09030          YKWGGAARYWYSTKMRKATMWCKWCRRGRWKRARGTSYYAYGYTRYTYAAATTACYCACA 20520
Bhutan-09005          CTWRRWMACACGCGMRTGWCTCGWYRARRTGGCGGYCCTRYKTWAYYCRWMYCRYYYTYC 20520
Indonesia-88035       CTWRRWMACACGCGMRTGWCTTTWYRARRTGGCGRYCCYRYGTWAYYYAWATCACYCACM 20520
Indonesia-88045       CTWRRWMACACGCGMRTGWCTTTWYRARRTGGCGRYCCYRYGTWAYYYAWATCACYCACM 20520
Indonesia-88065       CTWRRWMACACGCGMRTGWCTCGWYRARRTGGCGGYCCTRYKTWAYYCRWMYCRYYYTYC 20520
                       .*        .  **..     * *  *   .   .   *    *         * :

Bhutan-09015          GGMGTTGSAWSASYYKSSRYACCATCWGAAAWSGCMKMTTTAAGMSATYAGCRYKAMYCA 20580
Bhutan-09024          GGMGTTGSAWSASYYKSSRYACCATCWGAAAWSGCMKMTTTAAGMSAKYAGCRYKAMYCA 20580
Bhutan-09027          GGMGTTGSAWSASYYKSSRYACCATCWGAAAWSGCMKMTTTAAGMSATYAGCRYKAMYCA 20580
Bhutan-09030          GGMGTTGSAWSASYYKSSRYACCATCWGAAAWSGCMKMTTTAAGMSATYAGCRYKAMYCA 20580
Bhutan-09005          KCARCYRCRTCRGTTGSCACGYYRYCYRRRRWSRMCGATCCMRRASRGTCRYACGAACMA 20580
Indonesia-88035       GGARCYRCRTCRGTTGSCACGYCRYMYRRARWSRCCGAWCCMRRASRGTCRYACGMACMR 20580
Indonesia-88045       GSARCYRCRTCRGTTGSCACGYCRYMYRRRRWSRCCGAWCCMRRASRGTCRYACGMACMR 20580
Indonesia-88065       KCARCYRCRTCRGTTGSCACGYYRYCYRRRRWSRMCGATCCMRRASRGTCRYACGAACMA 20580
                             .  . .   *.  .     :    **            *   .

Bhutan-09015          CTMKWCAGTCGCRATYCMWRCRATTRMTYRSYYMRKMRWYCKAYGWGRTCWATTYASWAR 20640
Bhutan-09024          CTMKWCAGTCGCRATYCMWRCRATTRMTYRSYYMRKMRWYCKAYGWGRTCWATTYASWAR 20640
Bhutan-09027          CTMKWCAGTCGCRATYCMWRCRATTRMTYRSYYMRKMRWYCKAYGWGRTCWATTYASWAR 20640
Bhutan-09030          CTMKWCAGTCGCRATYCMWRCRATTRMTYRSYYMRKMRWYCKAYGWGRTCWATTYASWAR 20640
Bhutan-09005          CTMKTMMKYTRSARCYYCWRMRMCWRCWTRCYCCRTMAWYYRTYGWSGYGTRKCCMGTAG 20640
Indonesia-88035       MWCTTMMKTCRSARCYYCWRMRMCWRCWTRCYCCRTMAWYYRTYRWSRYGTATCCMGTMG 20640
Indonesia-88045       MWCTTMMKTCRSARCYYCTAMRMCWRCWTRCYCCRTMAWYYRTYRWSRYGTATCCMGTMG 20640
Indonesia-88065       CTMKTMMKYTRSARCYYCWRMRMCWRCWTRCYCCRTMAWYYRTYGWSGYGTRKCCMGTAG 20640
                         .       .   *     *   *   *.*  *.* ** ::* *.     .   .

Bhutan-09015          RMYACWRKAYSYMAYRKRYGTYYGYRYACGCTTKYMGMTYYGCSAYWYARCARKRYWTAT 20700
Bhutan-09024          RMYACWRKAYSYMAYRKRYGTYYGYRYACGCTTKYMGMTYYGCSAYWYARCARKRYWTAT 20700
Bhutan-09027          RMYACWRKAYSYMAYRKRYGTYYGYRYACGCTTKYMGMTYYGCSAYWYARCARKRYWTAT 20700
Bhutan-09030          RMYACWRKAYSYMAYRKRYGTYYGYRYACGCTTKYMGMTYYGCSAYWYARCARKRYWTAT 20700
Bhutan-09005          RMYRYARKRTCTMRTAGAYRYTCRCGTRTRTWCKYCAACCTTTGGYWYWRAWGTRTWYRK 20700
Indonesia-88035       RCYRYARKRTCTMRTAGAYRYTCRCGTRTRTWCKYCAACCTTTGGYWYWRAWGTRTWYRK 20700
Indonesia-88045       RCYRYARKRTCTMRTAGAYRYTCRCGTRTRTWCKYCAACCTTTGGYWYWRAWGTRTWYRK 20700
Indonesia-88065       RMYRYARKRTCTMRTAGAYRYTCRCGTRTRTWCKYCAACCTTTGGYWYWRAWGTRTWYRK 20700
                      * *   **  . *     *              ** .      ..*** *.  .* *  .
```

```
Bhutan-09015         WAKGYWRTCAWAGRRYCYYCKMTYCRSWGMRCKCRYSYGTWRTYWTAARRWRMTRRKGAA 20760
Bhutan-09024         WAKGYWRTCAWAGRRYCYYCKMTYCRSWGMRCKCRYSYGTWRTYWTAARRWRMTRRKGAA 20760
Bhutan-09027         WRKGYWRTCAWAGRRYCYYCKMTYCRSWGMRCKCRYSYGTWRTYWTAARRWRMTRRKGAA 20760
Bhutan-09030         WAKGYWRTCAWAGRRYCYYCKMTYCRSWGMRCKCRYSYGTWRTYWTAARRWRMTRRKGAA 20760
Bhutan-09005         TKGCTTRWYMTRCARCYMCGGCYTYRGAKMRYTTRYSMRAWRCYWYRRGRWRMGRRGSRW 20760
Indonesia-88035      TTGCTTRWYMTRCARCYMCGGCYTYRGAKCRYTTRYSMRATACYWYRRGRWRMGRRGSRW 20760
Indonesia-88045      TTGCTTRWCMTRCARCYMCGGCYTYRGAKMRYTTRYSMRATACYWYRRGRWRMGRRGSRW 20760
Indonesia-88065      TTGCTTRWYMTRCARCYMCGGCYTYRGAKMRYTTRYSMRAWRCYWYRRGRWRMGRRGSRW 20760
                           *       *          *.   * . ***  :   **    **** ** .

Bhutan-09015         ARCTRTRYWTAWKMRTAYRRGYMYGTYAYRGTATCGWKRYYGYWYTCRWWGCKGKSWTYA 20820
Bhutan-09024         ARCTRTRYWTAWKMRTAYRRGYMYGTYAYRGTATCGWKRYYGYWYTCRWWGCKGKSWTYA 20820
Bhutan-09027         ARCTRTRYWTAWKMRTAYRRGYMYGTYAYRGTATCGWKRYYGYWYTCRWWGCKGKSWTYA 20820
Bhutan-09030         ARCTRTRYWTAWKMRTAYRRGYMYGTYAYRGTATCGWKRYYGYWYTCRWWGCKGKSWTYA 20820
Bhutan-09005         RGYYRWGTAGGWKARYMTARSTMYRWTATAGCRAYRARRTYRCWCCYATTKYKKGSAGCW 20820
Indonesia-88035      RGCYAWGTAGGWKARYMTARSYMTRWTRTATCRAYRARRTYRCWCCYATTKYKKGSAGCW 20820
Indonesia-88045      RGCYAWGTAGGWKARYMTARSYMTRWTRTATCRAYRARRTYRCWCCYATTKYKKGSAGCW 20820
Indonesia-88065      RGYYRWGTAGGWKARYMTARSTMYRWTATAGCRAYRARRTYRCWCCYATTKYKKGSAGCW 20820
                               .** *    *. *          :   :* *  *        *  *

Bhutan-09015         GCWTGAAWYCRWTGTYMRYYSCMCMGKYAGCYCMAGCYGCKTWACWYKMTTTYRCCMSYY 20880
Bhutan-09024         GCWTGAAWYCRWTGTYMRYYSCMCMGKYAGCYCMAGCYGCKTWACWYKMTTTYRCCMSYY 20880
Bhutan-09027         GCWTGAAWYCRWTGTYMRYYSCMCMGKYRGCYCMAGCYGCKTWACWYKMTTTYRCCMSYY 20880
Bhutan-09030         GCWTGAAWYCRWTGTYMRYYSCMCMGKYAGCYCMAGCYGCKTWACWYKMTTTYRCCMSYY 20880
Bhutan-09005         AMAARCTWYYGAWRGTMGYTCYCACSKYARTTTMMCSTSYGYWMSATTAKYATATYAGCT 20880
Indonesia-88035      ACAARCTWYYGAWRGTMGYTCYCACSKYRRTTTMMGSTSYGYWMSATKMKYATATYAGCT 20880
Indonesia-88045      ACAARCTWYYGAWRGTMGYTCYCACSKYARTTTMMGSTSYGYWMSATKMKYATATYAGCT 20880
Indonesia-88065      AMAARCTWYYGAWRGTMGYTCYCACSKYARTTTMMCSTSYGYWMSATTAKYATATYAGCT 20880
                     .  : .:**       * * .  . .**     *  . .   * .  . . :     .

Bhutan-09015         TRWYCTRYYRSYACGYCYRRRAYTRGWGCGGGAGGSMYRASSRTYTARCAYCCKRATAYG 20940
Bhutan-09024         TRWYCTRYYRSYACGYCYRRRAYTRGWGCGGGAGGSMYRASSRTYTARCAYCCKRATAYG 20940
Bhutan-09027         TRWYCTRYYRSYACGYCYRRRAYTRGWGCGGGAGGSMTGASSRTYTARCAYCCKRATRYG 20940
Bhutan-09030         TRWYCTRYYRSYACGYCYRRRAYTRGWGCGGGAGGSMYRASSRTYTARCAYCCKRATAYG 20940
Bhutan-09005         KRTCYGATYASTGTACTTARAMCAARTAMKRTGKKRMYAGCCRYYYRASGTSSTGWWGCA 20940
Indonesia-88035      KRTCYGATYASTGTACTTARAMCAAGTAMKRTGKKRMYAACCRYYYRASGTSSTGWWGCA 20940
Indonesia-88045      KRTCYGATYASTGTACTTARAMCAARTAMKRTGKKRMYAACCRYYYRASGTSSTGWWGCA 20940
Indonesia-88065      KRTCYGATYASTGTACTTARAMCAARTAMKRTGKKRMYAGCCRYYYRASGTSSTGWWGCA 20940
                     .*      * * . .    *   :   .    .   *  ...* *   .. ...     .

Bhutan-09015         WTYTTTWGTCASMGCYTRMYTYYTYRGWRYRASGTRKYGGRRCCSMAYWYYYCMCCARAY 21000
Bhutan-09024         WTYTTTWGTCASMGYYTRMYTYYTYRGWRYRASGTRKYGGRRCCSMAYWYYYCMCCARAY 21000
Bhutan-09027         WTYTTTWGTCASMGYYTRMYTYYTYRGWRYRASGTRKYGGRRCCSMAYWYYYCMCCARAY 21000
Bhutan-09030         WTYTTTWGTCASMGYYTRMYTYYTYRGWRYRASGTRKYGGRRCCSMAYWYYYCMCCARAY 21000
Bhutan-09005         TKTWYCTRCYRCAAYYCRMCWCCYCAATACARCRARKYSRRRYSSMGTACTYYMCYGRRY 21000
Indonesia-88035      TKTWYCTRCYRCAAYYCRMCWCCYCAATACARCRARKTSRRRYSSMGTACTYYMCYGRRY 21000
Indonesia-88045      TKTWYCTRCYRCAAYYCAMCWCCYCAATACARCRARKYSRRRYSSMGTACTYYMMYGRRY 21000
Indonesia-88065      TKTWYCTRCYRCAAYYCRMCWCCYCAATACARCRARKYSRRRYSSMGTACTYYMCYGRRY 21000
                      .         . . *  *       .     . :** . ** .**.    * *  .* *

Bhutan-09015         YSRCYYGTGARYGKCRYRKAATRYMKGAYKKTAAGRMARRYAGSKRTMSGACYYRRGYRR 21060
Bhutan-09024         YSRCYYGTGARYGKCRYRKAATRYMKGAYKKTAAGRMARRYAGSKRTMSGACYYRRGYRR 21060
Bhutan-09027         YSRCYYGTGARYGKCRYRKAATRYMKGAYKKTAAGRMARRYAGSKRTMCGACYYRRGYRR 21060
Bhutan-09030         YSRCYYGTGARYGKCRYRKAATRYMKGAYKKTAAGRMARRYAGSKRTMCGACYYRRGYRR 21060
```

```
Bhutan-09005         TCAYCCRKRWACRKMGYRKCRYATMTRRYKTYMGSGARARCRRGGAACGRWYCYRRRCRG 21060
Indonesia-88035      TCAYCCRKRWACRKMGYRKCRYATMTRRYKTYMGSGARARCRRGGAACGRWYCYRRRCRG 21060
Indonesia-88045      TCAYCCRKRWACRKMGYRKCRYATMTRRYKTYMGSGARARCRRGGAACGRWYCYRRRCRG 21060
Indonesia-88065      TCAYCCRKRWACRKMGYRKCRYATMTRRYKTYMGSGARARCRRGGAACGRWYCYRRRCRG 21060
                      .     .     *  ***.    *.  **.  ..    *   .  :      ***  *

Bhutan-09015         WYWRKYTKRAAMGACCCCARMCKGSRYYTRRCATGYRTRRCYTYYTSYCGYCRWKCRKRR 21120
Bhutan-09024         WYWRKYTKRAAMGACCCCARMCKGSRYYTRRCATGYRTRRCYTYYTSYCGYCRWKCRKRR 21120
Bhutan-09027         WYWRKTTKRAAMGACCCCARMCKGSRYYTRRCATGYRTRRCYTYYTSYCGYCRWKCRKRR 21120
Bhutan-09030         WYWRKYTKRAAMGACCCCARMCKGSRYYTRRCATGYRTRRCYTYYTSYCGYCRWKCRKRR 21120
Bhutan-09005         TTTAKTCGRRRARRYMYYGAMYTASRCTWARSRARCAYRRYYCTTWSYYKYMAWKMRKAR 21120
Indonesia-88035      TTTAKTCGRRRARRYMYYGAMYTAGACTWARSRARCAYRRYYCTTWSYYKYMAWKMRKAR 21120
Indonesia-88045      TTTAKTCGRRRARRYMYYGAMYTAGACTWARSRARCAYRRYYCTTWSYYKYMAWKMRKAR 21120
Indonesia-88065      TTTAKTCGRRRARRYMYYGAMYTASRCTWARSRARCAYRRYYCTTWSYYGYMAWKMRKAR 21120
                         *   *         . * ...     *. :    ** *    **  *  ** ** *

Bhutan-09015         YSMAKCSRTCTMRTSKAATRCRWCWRRCYTRSRCCAYWTATYWTARRTKAYYAARKWGRC 21180
Bhutan-09024         YSMAKCSRTCTMRTSKAATRCRWCWRRCYTRSRCCAYWTATYWTARRTKAYYAARKWGRC 21180
Bhutan-09027         YSMAKCSRTCTMRTSKAATRCRWCWRRCYTRSRCCAYWTATYWTARRTKAYYAARKWGRC 21180
Bhutan-09030         YSMAKCSRTCTMRTSKAATRCRWCWRRCYTRSRCCAYWTATYWTARRTKAYYAARKWGRC 21180
Bhutan-09005         YSARTSSRYTYAAYCTRTGGMRTYAAAYCYRCRCYACWCWYTGYRRRYRRTYMRAKTRAY 21180
Indonesia-88035      YSARTSSRYTYAAYCTRTGGMRTYAAAYCYRCRGTRCWCWYTGYRRRYRRTYMRAKTGAC 21180
Indonesia-88045      YCARTSSRYTCAAYCTATGGMRTYAAAYCYRCRGTRCWCWYTGYRRRYRRTYMRAKTGAC 21180
Indonesia-88065      YSARTSSRYTYAAYCTRTGGMRTYAAAYCYRCRCYACWCWYTGYRRRYRRTYMRAKTRAY 21180
                     *.  ..**      .. :   *        *.*    *       ** :  *   *

Bhutan-09015         SRRKGYCYRAYYWGYYTRCRRSWTCYYRYRAMYGTRRYYCTMWWCRMTGKRWCAYAAATR 21240
Bhutan-09024         SRRKGYCYRAYYWGYYTRCRRSWTCYYRYRAMYGTRRYYCTMWWCRMTGKRWCAYAAATR 21240
Bhutan-09027         SRRKGYCYRAYYWGYYTRCRRSWTCYYRYRAMYGTRRYYCTMWWCRMTGKRWCAYAAATR 21240
Bhutan-09030         SRRKGYCYRAYYWGYYTRCRRSWTCYYRYRAMYGTRRYYCTMWWCRMTGKRWCAYAAATR 21240
Bhutan-09005         GRRKRYYYAWTYTSCTKGYGAGAYYTCATARCYRWGGCCYKATWYAMKRKRAYMTWRATR 21240
Indonesia-88035      GRATGCCTAATTTSCTTGCGAGACYTCATAACTGTGGCCCTATACACTGTAACATAAATA 21240
Indonesia-88045      GRATGCCTAATTTSCTTGCGAGACYTCATAACTGTGGCCCTATACACTGTAACATAAATA 21240
Indonesia-88065      GRRKRYYYAWTYTSCTKGYGAGAYYTCATARCYRWGGCCYKATWYAMKRKRAYMTWRRYR 21240
                     .* .         .  .    .                  .      . .

Bhutan-09015         RRRKTTMGTACGTAKRCRCYCCAGAGMACYRTYYMARWYGKRRSRRAAMMRKGATRWTTW 21300
Bhutan-09024         RRRKTTMGTACGTAKRCRCYCCAGAGMACYRTYYMARWYGKRRSRRAAMMRKGATRWTTW 21300
Bhutan-09027         RRRKTTMGTACGTAKRCRCYCCAGAGMACYRTYYMARWYGKRRSRRAAMMRKGATRWTTW 21300
Bhutan-09030         RRRKTTMGTACGTAKRCRCYCCAGAGMRMYRTYYMARWYGKRRSRRAAMMRKGATRWTTW 21300
Bhutan-09005         GRGTKTAKYMSRWWKGYAYCSYMSRRAACCRGYYAGAWYRKRASAARRACAKRMGRWTAA 21300
Indonesia-88035      GGGTTWAGTASGTAYGCACCCCMSRGAACCRGYYAGAWYRKRASAARRACAKRMGRWYAA 21300
Indonesia-88045      GGGTTWAGTASGTAYGCACCCCMGRRAACCRGYYAGAWYRKRASAARRACAKRMGRWYAA 21300
Indonesia-88065      GRGTKTAKYMSRWWKGYAYCSYMSRRARMCRGYYAGAWYRKRASAARRACRKRMGRWTAA 21300
                        ..     .         .  .      * ** . ** ** *       *   ** :

Bhutan-09015         CGYMRGRRCGYMWKASWTYAYWWCARTRSMWRWRRRGTARKTRYMRCYYGCWGATRSYCT 21360
Bhutan-09024         CGYMRGRRCGYMWKASWTYAYWWCARTRSMWRWRRRGTARKTRYMRCYYGCWGATRSYCT 21360
Bhutan-09027         CGYMRGRRCGYMWKASWTYAYWWCARTRSMWRWRRRGTARKTRYMRCYYGCWGATRSYCT 21360
Bhutan-09030         CGYMRGRRCGYMWKASWTYAYWWCARTRSMWRWRRRGTARKTRYMRCYYGCWGATRSYCT 21360
Bhutan-09005         MRYCGKAASKCMTGGCAKYGYTAMTAYAGMARWRRGRYRATKRTAWSYCRSASWWASYYY 21360
Indonesia-88035      MRYCGKAASKCMTGGCAKYGYTAMTAYAGMARWGRGRYRATKRTAWSYCRSASWWASYYY 21360
Indonesia-88045      MRYCGKAASKCMTGGCAKYGYTAMTAYAGMARWGRGRYRATKRTAWSYCRSASWTASYYY 21360
Indonesia-88065      MRYCGKAASKCMTGGCAKYGYTAMTAYAGMARWRRGRYRATKRTAWSYCRSASWWASYYY 21360
```

```
                       *     .  *  .. .*.*   :   .* ** *     ..*   .*  . .   **

Bhutan-09015         YMTYYMCAYMKSYYCYKGTWYSYCMTAYGCAKWKWKGTAYYTTRAWTRGRRRYTARTSRR 21420
Bhutan-09024         YMTYYMCAYMKSYYCYKGTWYSYCMTAYGCAKWKWKGTAYYTTRAWTRGRRRYTARTSRR 21420
Bhutan-09027         YMTYYMCAYMKSYYCYKGTWYSYCMTAYGCAKWKWKGTAYYTTRAWTRGRRRYTARTSRR 21420
Bhutan-09030         YMTYYMCAYMKSYYCYKGTWYSYCMTAYGCAKWKWKGTAYYTTRAWTRGRRRYTARTSRR 21420
Bhutan-09005         CCKYYMYRCCKSCYSCTKYWTCYYMYTYRARTWGATACRTTWWGRAWRRRAGYYRRYSRA 21420
Indonesia-88035      CCKYYMYRCCKSCYSCTKYWTCYYMYTYRARTWGATACRTTWWGGAWRRRAGYYRRYSRA 21420
Indonesia-88045      CMKYTMYRCCKSCYSCTKYWTCYYMYTYRARTWGATACRTTWWGGAWRRRAGYYRRYSRA 21420
Indonesia-88065      CMKYYMYRCCKSCYSCTKYWTCYYMYTYRARTWGATACRTTWWGGAWRRRAGYYRRYSRA 21420
                       .* *    ** *. .  * .* * :* . .*  ..          * *  *  * **

Bhutan-09015         ATCMGRAGYARYMRATTWTATTAAYRRKRRACGGCRAKKTCTYYAKTCGYYWRMWARTYY 21480
Bhutan-09024         ATCMGRAGYARYMRATTWTATTAAYRRKRRACGGCRAKKTCTYYAKTCGYYWRMWARTYY 21480
Bhutan-09027         ATCMGRAGYARYMRATTWTATTAAYAGTRRACGGCRAKKTCTYYAKTCGYYWRMWARTYY 21480
Bhutan-09030         ATCMGRAGYARYMRATTWTATTAAYRRKRRACGGCRAKKTCTYYAKTCGYYWRMWARTYY 21480
Bhutan-09005         WYACAGRRTWATCGGCWAKRCCTAYAGWARRCGGYRRKKGAGCTGTYSRCYWRMWRGYTY 21480
Indonesia-88035      WYACARRRTWATCGGCWAKRCCTWYAGWAGRYSRYRRKKGAGCTGTYSRCYWRMWRGYTY 21480
Indonesia-88045      WYACARRRTWATCGGCWAKRCCTWYAGWAGRYSRYRRKKGAGCTGTYSRCYWRMWRGYTY 21480
Indonesia-88065      WYACARRRTWATCGGCWAKRCCTAYAGWARRCGGYRRKKGAGCTGTYSRCYWRMWRGYTY 21480
                       . .         .   .   : *       .  * ** .   .. .  *****    *

Bhutan-09015         RRTYWCCTCWYRMWKCYAGYYYRTASRTKWCAAYYYYRYWRAWRRKMWAKYGSTRWKCKK 21540
Bhutan-09024         RRTYWCCTCWYRMWKCYAGYYYRTASRTKWCAAYYYYRYWRAWRRKMWAKYGSTGATCKK 21540
Bhutan-09027         RRTYWCCTCWYRMWKCYAGYYYRTASRTKWCAAYYYYRYWRAWRRKMWAKYGSTRWKCKK 21540
Bhutan-09030         RRTYWCCTCWYRMWKCYAGYYYRTASRTKWCAAYYYYRYWRAWRRKMWAKYGSTRWKCKK 21540
Bhutan-09005         ARYTASYYMTTGMTKGYRKYYCRYRGAYGWSWGCCCYRCAAGAGRKMTCTYAGAGWTSGK 21540
Indonesia-88035      ARYTASYYMTTGMTKGYRKYYCRYRGAYGWSWGCCCYRCAAGAGRKMTCTYAGAGWTSGK 21540
Indonesia-88045      ARYTASYYMTTGMTKGYRKYYCRYRGAYGWSWGCCCYRCAAGAGRKMTCTYAGAGATSGK 21540
Indonesia-88065      ARYTASYYMTTGMTKGYRKYYCRYRGAYGWSWGCCCYRCAAGAGRKMTCTYAGAGWTSGK 21540
                      *   .      * * *  ** *  .   *. .   **   .  *** ..*..:  .. *

Bhutan-09015         GKTSGSMATWGGWTRTGATTMSWWGACASWTAAYGTMTRTCWCYRTYAGYRSGTYATRRG 21600
Bhutan-09024         GKTSGSMATWGGWTRTGATTMSWWGACASWTAAYGTMTRTCWCYRTYAGYRSGTYATRRG 21600
Bhutan-09027         GKTSGSMATWGGWTRTGATTMSWWGACASWTAAYGTMTRTCWCYRTYAGYRSGTYATRRG 21600
Bhutan-09030         GKTSGSMATWGGWTRTGATTMSWWGACASWTAAYGTMTRTCWCYRTYAGYRSGTYATRRG 21600
Bhutan-09005         TKWSRGARWTRRWWGKKRCCACTATACTGAGGMTRYMYRYYTATGYYARCASGKCACRGR 21600
Indonesia-88035      TKWSRGARWTRRWWGKTGCCASTWTRYTGAGGMTRYMYRYYTATGYTRRCAGRGYRCRRR 21600
Indonesia-88045      TKWSRGARWTRRWWGKTGCCASTWTRYTGAGGMTRYMYRYYTATGYTRRCAGRGYRCRRR 21600
Indonesia-88065      TKWSRGARWTRRWWGKTGCCACTATRYTGAGGMTRYMYRYYTATGYYARCASGKCACRGR 21600
                      * * .      *  .     .     :.  .    * *   .        .     *

Bhutan-09015         RGRAMRRCAMWCRYRMWRSTTRTKTYMGCCAAACYCSYKTCYGKRTATKGAATRYYYRAG 21660
Bhutan-09024         RGRAMRRCAMWCRYRMWRSTTRTKTYMGCCAAACYCSYKTCYGKRTATKGAATRYYYRAG 21660
Bhutan-09027         RGRAMRRCAMWCRYRMWRSTTRTKTYMGCCAAACYCSYKTCYGKRTATKGAATRYYYRAG 21660
Bhutan-09030         RGRAMRRCAMWCRYRMWRSTTRTKTYMGCCAAACYCSYKTCYGKRTATKGAATRYYYRAG 21660
Bhutan-09005         RRGRCAASMAAARTAMTRGGYGKGKCCATCWWMSTSCTTYMTRGRWWWTRRWYRCCCRGR 21660
Indonesia-88035      RRGRCAASMAAMRTAMTGGGYGKGKCCATYWWMSTCCTTYMTRGRWWWTRRWYRCCCRGR 21660
Indonesia-88045      RRGRCAASMAAMRTAMTGGGYGKGKCCATYWWMSTCCTTYMTRGRWWWTRRWYRCCCRGR 21660
Indonesia-88065      RRGRCAASMAAARTAMTRGGYGKGKCCATCWWMSTSCTTYMTRGRWWWTRRWYRCCCRGR 21660
                     *      .    *  *  .   . .  .     . .. .     *   .    *   *.

Bhutan-09015         CTARTYCGTRKGMRAYGYGTRMGYSRTGYCAGKTCYWWSYTGTAYMAYWCATCRCYCYAR 21720
Bhutan-09024         CTARTYCGTRKGMRAYGYGTRMGYSRTGYCAGKTCYWWSYTGTAYMAYWCATCRCYCYAR 21720
```

```
Bhutan-09027        CTARTYCGTRKGMRAYGYGTRMGYSRTGYCAGKTCYWWSYTGTAYMAYWCATCRCYCYAR 21720
Bhutan-09030        CTARTYCGTRKGMRAYGYGTRMGYSRTGYCAGKTCYWWSYTGTAYMAYWCATCRCYCYAR 21720
Bhutan-09005        YCRRYCYCAATSCARTGCKCRMCYSAASCTGKKKMTAASYCTCWCSRYWMWKYGTCYTRG 21720
Indonesia-88035     YCRRYCYCAATSCARTSCKCRMCYSAASCTGKKKMTAASYCTCWCSRYWMWKYGTCYTRG 21720
Indonesia-88045     YCRRYCYCAATSCARTSCKCRMCYSAASCTGKKKMTAASYCTCWCSRYWMWKYGTCYTRG 21720
Indonesia-88065     YCRRYCYCAATSCARTGCKCRMCYSAASCTGKKKCTAASYCTCWCSRYWMWKYGTCYTRG 21720
                       *     : ..     .    ** ** :.   . *.      **        **   .

Bhutan-09015        GCGWRTYRGRYYYRSGSRYYACCTRYMSRWRYYGTWAWYRSKYWCYCRRSTAYYMAWAWC 21780
Bhutan-09024        GCGWRTYRGRYYYRSGSRYYACCTRYMSRWRYYGTWAWYRSKYWCYCRRSTAYYMAWAWC 21780
Bhutan-09027        GCGWRTYRGRYYYRSGSRYYACCTRYMSRWRYYGTWAWYRSKYWCYCRRSTAYYMAWAWC 21780
Bhutan-09030        GCGWRTYRGRYYYRSGSRYYACCTRYMSRWRYYGTWAWYAGKYWCYCRRSTAYYMAWAWC 21780
Bhutan-09005        CYKAAWTAARTCTGSKSAYTRMMWGCMCGTGYTTATRWYGGCTWYCAGACAACTCTAAAT 21780
Indonesia-88035     CTGAAWTAARTCTGSKSAYTRCCAGYMCGTGYTTATRWTGSYTAYCCGACTMYYCAWMAY 21780
Indonesia-88045     CTGAAWTAARTCTGSKSAYTRCCAGYMCGTGYTTATRWTGSYTWYCCGACTMYYCAWMAY 21780
Indonesia-88065     CYKAAWTAARTCTGSKSAYTRMMWGCMCGTGYTTATRWYGGCTATCAGACWAYYCWWMAT 21780
                              .*     * * *         *.    *   :   *   .       .   .

Bhutan-09015        RRKWRCMACRRCYYTMRTMYYWCKGCRTAGRTSKWCTRCWARAASTYRRRYAWCYCRRKK 21840
Bhutan-09024        RRKWRCMACRRCYYTMRTMYYWCKGCRTAGRTSKWCTRCWARAASTYRRRYAWCYCRRKK 21840
Bhutan-09027        RRKWGCMACRRCYYTMRTMYYWCKGCRTAGRTSKWCTRCWARAASTYRRRYAWCYCRRKK 21840
Bhutan-09030        RRKWRCMACRRCYYTMRTMYYWCKGCRTAGRTSKWCTRCWARAASTYRRRYAWCYCRRKK 21840
Bhutan-09005        GGGAACMRMRAMYYWMRYCTTAYTRSAWARACCKTYKAYWTGRWSYCRRACRAYYMAAGK 21840
Indonesia-88035     GGGAASMRMRAMYYWMRYCTTAYTRSAWWRACCKTYKAYWTGRWSYCRRACRAYYMAAGK 21840
Indonesia-88045     GGGAASMRMRAMYYWMRYCTTAYTRSAWWRACCKTYKAYWTGRWSYCRRACRAYYMAAGK 21840
Indonesia-88065     GGGAASCRMRAMYYWMRYCTTAYTRSAWARACCKTYKAYATGGTSYCRRACRAYYMAAGK 21840
                          .    *   ** **         . .        .*   .     :    *   **       *     *

Bhutan-09015        KCYGGGRARRCRGRWSTYRSYATCAATAWTRYYRYRYRGWARGMRGYYCGMSGAATRRYA 21900
Bhutan-09024        KCYGGGRARRCRGRWSTYRSYATCAATAWTRYYRYRYRGWARGMRGYYCGMSGAATRRYA 21900
Bhutan-09027        KCYGGGRARRCRGRWSTYRSYATCARYAWTRYYRYRYRGWARGMRGYYCGMSGAATRRYA 21900
Bhutan-09030        KCYGGGRARRCRGRWSTYRSYATCARYAWTRYYRYRYRGWARGMRGYYCGMSGAATRRYA 21900
Bhutan-09005        TYCARTRRGGYARGTGYCRCTRCSWRTRAYAMCAYGTGSWGARCAAYTYTMCKWRYAAYR 21900
Indonesia-88035     TYCARTRRGGYARGTGYCRCTRCSWRTRAYAMCAYGTGSWGARCAAYTYTMCTWRYAAYR 21900
Indonesia-88045     TYCAATRRGGYARGTGYCRCTRCSWRTRAYAMCAYGTGSWGARCAAYTYTMCTWRYAAYR 21900
Indonesia-88065     TTCAATRRGGYARGTGYCRCTRCSWRTRAYAMCAYGTGSWGAACAAYTYTMCTWRYAAYR 21900
                    .   .   *          .   *.    .            *    .*.     .*    *.       *

Bhutan-09015        WTYARYCKCCCGWYGYYRYYWCKTAYYGSGYRRYYMTYGWYTTGGRCCAMRGYRRKKKRC 21960
Bhutan-09024        WTYARYCKCCCGWYGYYRYYWCKTAYYGSGYRRYYMTYGWYTTGGRCCAMRGYRRKKKRC 21960
Bhutan-09027        WTYARYCKCCCGWYGYYRYYWCKTAYYGSGYRRYYMTYGWYTTGGRCCAMRGYRRKKKRC 21960
Bhutan-09030        WTYARYCKCCCGWYGYYRYYWCKTAYYGSGYRRYYMTYGWYTTGGRCCAMRGYRRKKKRC 21960
Bhutan-09005        AYYTRTTKYTYKTCRYYAYYAAGGGCCRSKTGRCYCYYSTCYYKKRMYRMGRTGAKTGGM 21960
Indonesia-88035     AYYTRTTKYTYKTCRYYAYYAAGGGCCRSKTGRCYCYYSTCYYKKRMYRMGRTGAKTGGM 21960
Indonesia-88045     AYYTRTTKYTYKTCRCCAYYAAGGGCCRSKTGRCYCYYSTCYYKKRMYRMGRTGAKTGGM 21960
Indonesia-88065     AYYTRTTKYTYKTCRYYACCAAGGGCCRSKTGRCYCYYSTCYYKKRMYRMGRTGAKTGGM 21960
                      *:*   *                 .   .    *    * *   *.       *    *      *.

Bhutan-09015        AYCWGGTMYGRYRGWKYTYRAYRRCARMSMMRTKARMRGYATARATATRGARCAYYRMWR 22020
Bhutan-09024        AYCWGGTMCGRYRGWKYTYRAYRRCARMSMMRTKARMRGYATARATATRGARCAYYRMWR 22020
Bhutan-09027        AYCWGGTMYGRYRGWKYTYRAYRRCARMSMMRTKARMRGYATARATATRGARCAYYRMWR 22020
Bhutan-09030        AYCWGGTMYGRYRGWKYTYRAYRRCARMSMMRTKARMRGYATARATATRGARCAYYRMWR 22020
Bhutan-09005        RYSAKRCCCKACARWTYWCARTRRMRAACMMGWGTRCGRCMYRRGGGWRAMGYMTYRMTG 22020
Indonesia-88035     RYSAKRCCCKACARWTYWCARTRRMRAACMMGTGTRCGRCMYRRGGGWRAAGYMTYRMTG 22020
```

```
Indonesia-88045      RYSAKRCCCKACARWTYWCARTRRMRAACMMGTGTRCGRCMYRRGGGWRAAGYMTYRMTG 22020
Indonesia-88065      RYSAKRCCCTACARWTYWCARTRRMRAACMMGWGTRCGRCMYRRGGGWRAMGYMTYRMTG 22020
                      *.           *.*     **    .**   :*       *. . *.     ***

Bhutan-09015         WYYRAAYRTSAKYYYYATKRYKYWMRAAWYYYGAMTKRAATGCKWWRWYKYGTYGTYSKG 22080
Bhutan-09024         WYYRAAYRTSAKYYYYATKRYKYWMRAAWYYYGAMTKRAATGCKWWRWYKYGTYGTYSKG 22080
Bhutan-09027         WYYRAAYRTSAKYYYYATKRYKYWMRAAWYYYGAMTKRAATGCKWWRWYKYGTYGTYSKG 22080
Bhutan-09030         WYYRAAYRTSAKYYYYATKRYKYWMRAAWYYYGAMTKRAATGCKWWRWYKYGTYGTYSKG 22080
Bhutan-09005         WTCATWTGYSRGCCYYRYGATKYTMRRCATYCSRAYGRWGCKAGTAATCTTKYTRAMCKR 22080
Indonesia-88035      WTCATWTGYSRGCCYYRYGATKYTMRRCATYCSRAYGRWGCKAGTAATCTTKYTRAMCKR 22080
Indonesia-88045      WTCATWTGYSRGCCYYRYGATKYTMRRCATYCSRAYGRWRCKAGTAATCTTKYTRAMCKR 22080
Indonesia-88065      WTCATWTGYSRGCCYYRYGATKYTMRRCATYCSRAYGRWRCKAGTAATCTTKYTRAMCKR 22080
                     *   :    *    **     ** ** .  * .    *    .      .     : .*

Bhutan-09015         TYCYWAYKRKTTAACRRGRTTRRYYRSMCRTMGYTRTYYYCCYTCKCYRGMKTTRTATTY 22140
Bhutan-09024         TYCYWAYKRKTTAACRRGRTTRRYYRSMCRTMGYTRTYYYCCYTCKCYRGMKTTRTATTY 22140
Bhutan-09027         TYCYWAYKRKTTAACRRGRTTRRYYRSMCRTMGYTRTYYYCCYTCKCYRGMKTTRTATTY 22140
Bhutan-09030         TYCYWAYKRKTTAACRRGRTTRRYYRSMCRTMGYTRTYYYCCYTCKCYRGMKTTRTATTY 22140
Bhutan-09005         YCCYMWYTGGWWMRTGRRRYYRATYASMCATMKCTGYTYCGTYWYGYTRRCGAKGKAWWT 22140
Indonesia-88035      YCYYMWYTGGWWMRTGRRRYYRATYGSAYAYCKCCGYTYCGTTWYGYTRRCGAKGKRTWT 22140
Indonesia-88045      YCYYMWYTGGWWMRTGRRRYYRATYGSAYAYCKCCGYTYCGTTWYGYTRRCGAKGKRTWT 22140
Indonesia-88065      YCCYMWYTGGWWMRTGRRRYYRATYASMCATMKCTGYTYCGTYWYGYTRRCGAKGKAWWT 22140
                        *  *.        * *  *  * *           *         *   :. .

Bhutan-09015         MWYTARWSRKCTWKYCAYRYKYYWCAGAYMMAYTYKGRYWRYRRSYWYYKWRMRRKRMYT 22200
Bhutan-09024         MWYTARWSRKCTWKYCAYRYKYYWCAGRYMMAYTYKGRYWRCRRSYWYYKWAMRRKRMYT 22200
Bhutan-09027         MWYTARWSRKCTWKYCAYRYKYYWCAGRYMMAYTYKGRYWRYRRSYWYYKWRMRRKRMYT 22200
Bhutan-09030         MWYTARWSRKCTWKYCAYRYKYYWCAGRYMMAYTYKGRYWRYRRSYWYYKWRMRRKRMYT 22200
Bhutan-09005         AWYWMATSAGYATKYSRYRTKYTTMGTAYMARCCCGRGTTRCGGCCTYTKAAAAATAMYW 22200
Indonesia-88035      AWYWMATSAGYATKYSRYRTKYTTMGTAYMARCCCGRGTTRCGGCCTYTKAAAAATAMYW 22200
Indonesia-88045      AWYWMATSAGYATKYSRYRTKYTTMGTAYMARCCCGRGTTRCGGCCTYTKAAAAATAMYW 22200
Indonesia-88065      AWYWMATSAGYATKYSRYRTKYTTMGTAYMARCCCGRGTTRYGGCCTYTKAAAAATAMYW 22200
                      **    *   : **. ** **   .  **          *   .  * *     . **

Bhutan-09015         RCCYSYYYSGTCMRYGGRYKKMCKRCRMRTWYCYRTYKCYGMRAATGKGACYWCRGWACW 22260
Bhutan-09024         RCCYSYYYSGTCCAYGGRYKKMCKRCRMRTWYCYRTYKCYGMRAATGKGACYWCRGWACW 22260
Bhutan-09027         RCCYSYYYSGTCMRYGGRYKKMCKRCRMRTWYCYRTYKCCGMRAATGKGACYWCRGWACW 22260
Bhutan-09030         RCCYSYYYSGTCMRYGGRYKKMCKRCRMRTWYCYRTYKCCGMRAATGKGACYWCRGWACW 22260
Bhutan-09005         ATTTCCCCCKCGMRTKCRTTGCATGYAARWTCSTATYGYTACARRYKTAGTYWYRRKGTW 22260
Indonesia-88035      AYTTCCCCCKCGCATKCRTTGCATGYAARWTCSTAWYGYTACARRYKTAGTYWYRRKGTW 22260
Indonesia-88045      AYTTCCCCCKCGCATKCRTTGCATGYAARWTCSTAWYGYTACARRYKTAGTYWYRRKGTW 22260
Indonesia-88065      AYTTCCCCCKCGMRTKCRTTGCATGYAARWTCSTATYGYTACARRYKTAGTYWYRRKGTW 22260
                         .   .        * .  ..    *   .   *   .      ... ** *  . *

Bhutan-09015         RRCRRTTYKGYTMCTYYASCKRKYGCSMTGCKRYRYYCTRAWWARGKAGTWGGGGGYCWA 22320
Bhutan-09024         RRCRRTTYKGYTMCTYYASCKRKYGCSMTGCKRYRYYCTRAWWARGKAGTWGGGGGYCWA 22320
Bhutan-09027         RRCRRTTYKGYTMCTYYASCKRKYGCSCTGCKRYRYYCTRAWWARGKAGTWGGGGGYCWA 22320
Bhutan-09030         RRCRRTTYKGYTMCTYYASCKRKYGCSMTGCKRYRYYCTRAWWARGKAGTWGGGGGYCWA 22320
Bhutan-09005         RGTARGGYGKTKCAKTYRGTGGKYCTSACRYKRTGYYYYRGAACARGTRGWCAAAKYCWM 22320
Indonesia-88035      RGTARGGYGKTGMAKTYRGTGGKYCTCACRYKRTGYYYYRGAACARGWRGWCAAATYCWC 22320
Indonesia-88045      RGTARGGYGKTKMAKTYRGTGGKYCTSACATKRTGYYYYRGAACARGWRGWCAAATYCWC 22320
Indonesia-88065      RGTARGGYGKTKCAKTYRGTGGKYCTCACRYKRTGYYYYRGAACARGTRGWCAAAKYMWM 22320
                     *   *  *     .. * .   **  .    **  **  *.  .      * ... * *
```

```
Bhutan-09015         YKSWTAYRRCMAMAYWYMRKMGCKKRGWRRYCCYKWGRKKTRRACRYRKATAYYYARASR 22380
Bhutan-09024         YKSWTAYRRCMAMAYWYMRKMGCKKRGWRRYCCYKWGRKKTRRACRYRKATAYYYARASR 22380
Bhutan-09027         YKSWTAYRRCMAMAYWYMRKMGCKKRGWRRYCCYKWGRKKTRRACRYRKATAYYYARASR 22380
Bhutan-09030         YKSWTAYRRCMAMAYWYMRKMGCKKRGWRRYCCYKWGRKKTRRACRYRKATAYYYARASR 22380
Bhutan-09005         TGCWWAYARYATARCWCMGKMSSKTRCWGATYYYGAKATGYARRMRYGTRARTTTMGGSR 22380
Indonesia-88035      TGCTWRYARYAAARCWCMGKMGSKTRCWGATYYYGAGATGYARRMRYGTRARTTTMGGSR 22380
Indonesia-88045      TGCTWRYARYAAARCWCMGKMGSKTRCWGATYYYGAGATGYARRMRYGTRARTTTMGGSR 22380
Indonesia-88065      TGCWWAYARYATARCWCMGKMSSKTRCWGATYYYGAKATGYARRMRYGTRARTTTMGGSR 22380
                       .   * *  :   * * **..*.* *     *    .   *  ** . :      .**

Bhutan-09015         YYCGGTWAACGTMACTTATAGARYCTRGRMRYAMYYRYYTGWKYTRSWYYGCARAKKKRG 22440
Bhutan-09024         YYCGGTWAACGTMACTTATAGARYCTRGRMRYAMYYRYYTGWKYTRSWYYGCARAKKKRG 22440
Bhutan-09027         YYCGGTWAACGTMACTTATAGARYCTRGRMRYAMYYRYYTGWKYTRSWYYGCARAKKKRG 22440
Bhutan-09030         YYCGGTWAACGTMACTTATAGARYCTRGRMRYAMYYRYYTGWKYTRSWYYGCARAKKKRG 22440
Bhutan-09005         YYYKRRAAWTATCRTKCGGTAMRTSWGRGMACGCCCAYCYRWGCKACWCYRYMRWGGKRR 22440
Indonesia-88035      YYYKRRARWTRKCRTKCGGTAMATSWGRGMACGCCCAYCYRWGCKACWCYRYMRWGGKRR 22440
Indonesia-88045      YYYKRRARWTRKCRTKCGGTAMRTSWGRGMACGCCCAYCYRWGCKACWCYRYMRWGGKRR 22440
Indonesia-88065      YYYKRRAAWTATCRTKCGGTAMRTSWGRGMACGCCCAYCYRWGCKACWCYRYMRWGGKRR 22440
                     **         .   . . :.   .    *  .    *   *  . .* *   *   **

Bhutan-09015         AGYYMSKKYYCGTTGTRKTMMMTGMRARKAGATGATGWRTAYSWMYGSGGRCKTGKTRRA 22500
Bhutan-09024         AGYYMSKKYYCGTTGTRKTMMMTGMRARKAGATGATGWRTAYSWMYGSGGRCKTGKTRRA 22500
Bhutan-09027         AGYYMSKKYYCGTTGTRKTMMMTGMRARKAGATGATGWRTAYSWMYGSGGRCKTGKTRRA 22500
Bhutan-09030         AGYYMSKKYYCGTTGTRKTMMMTGMRARKAGATGATGWRTAYSWMYGSGGRCKTGKTRRA 22500
Bhutan-09005         RKCYMGTTYWAAYWRGAGYCCMWRCGGAGRTCKRWYKWAYRTCACCRSAAGTKYSTYAGR 22500
Indonesia-88035      RKCYMGTTYWAAYWRGAGYCCMWRCGGAGRTCKRWYKWAYRTCACCRSAAGTKYSTYAGR 22500
Indonesia-88045      RKCYMGTTYWAAYWRGAGYCCMWRCGGAGRTCTRWYKWAYRTCACCRSAAGTKYSTYAGR 22500
Indonesia-88065      RKCYMGTTYWAAYWRGAGYCCMWRCGGAGRTCKRWYKWAYRTCACCRSAAGTKYSTYAGR 22500
                        **...*:..         *    .    ..    *    .    *..  * ..

Bhutan-09015         TTCYKRCMTCCRYTGTRAMARRRACAARGMRTMTWCACGMAACRRMYTAYYGAKKMGGGY 22560
Bhutan-09024         TTCYKRCMTCCRYTRYRAMARRRACAARGMRTMTWCACGMAACRRMYTAYYGAKKMGGGY 22560
Bhutan-09027         TTCYKRCMTCCRYTRYRAMARRRACAARGMRTMTWCACGMAACRRMYTAYYGAKKMGGGY 22560
Bhutan-09030         TTCYKRCMTCCRYTRYRAMARRRACAARGMRTMTWCACGMAACRRMYTAYYGAKKMGGGY 22560
Bhutan-09005         YYSTGASMYYYRYKGTRRWCRARWMMWGRCAYMYWYMYRMWRYAAAYKTTCRGTTARSRT 22560
Indonesia-88035      YYSTGASMYYYRYKGTRRWCRARWMMWGRCAYMYWYMYRMWRYAAAYKTTCRGTTARSRT 22560
Indonesia-88045      YYSTGASMYYYRYKGTRRWCRARWMMWGRCAYMYWYMYRMWRYAAAYKTTCRGTTARSRT 22560
Indonesia-88065      YYSTGASMYYYRYKGTRRWCRARWMMWGRCAYMYWYMYRMWRYAAAYKTTCRGTTARSRT 22560
                       .   .*   **.  *  .* *         * *    *      *.:   ...  .

Bhutan-09015         SAAWYARYAYYRTGYRWAWWTAMTWGRCSKGYGAYWAATYKYTYATSACWGGYARYRAGY 22620
Bhutan-09024         SAAWYARYAYYRTGYRWAWWTAMTWGRCSKGYGAYWAATYKYTYATSACWGGYARYRAGY 22620
Bhutan-09027         SAAWYARCATYRTGYRWAWWTAMTWGRCSKGYGAYWAATYKYTYATSACWGGYARYRAGY 22620
Bhutan-09030         SAAWYARCATYRTGYRWAWWTAMTWGRCSKGYGAYWAATYKYTYATSACWGGYARYRAGY 22620
Bhutan-09005         SRRATRAYRYTGCAYATGTAYCCATAGSGGAYAMYWTWWTTCYCRKGGMWRSCWGTAGKY 22620
Indonesia-88035      SRRATRAYRYTGCAYATGTAYCCATAGSGGGYAMYTTWWTTCYCRKGGMWRSCWGTAGKY 22620
Indonesia-88045      SRRATRAYRYTGCAYATGTAYCCATAGSGGGYAMYTTWWTTCYCRKGGMWRGCWGTAGKY 22620
Indonesia-88065      SRRATRAYRYTGCAYATGTACCCATAGSGGAYAMYWTWWTTCYCRKGGMWRGCWGTAGKY 22620
                     *            .*  .   . : . .. .*. * :   .    ... * .     . *

Bhutan-09015         RSTRRRWGTWWSGRRCTCRRRGAWAAKRYCWCYYYCGYACRCYKRAKRAYATCCYASYCA 22680
Bhutan-09024         RSTRRRWGTWWSGRRCTCRRRGAWAAKRTCWSYYYCGYACRCYKRAKRAYATCCYASYCA 22680
Bhutan-09027         RSTRRRWGTWWSGRRCTCRRRGAWAAKRYCWSYYYCGYACRCYKRAKRACATCCYASYCA 22680
Bhutan-09030         RSTRRRWGTWWSGRRCTCRRRGAWAAKRYCWSYYYCGYACRCYKRAKRAYATCCYASYCA 22680
```

```
Bhutan-09005        ACCRGRAAAAWCKGRSYYGAARGARATRTMASYCYMSCRTAMTGGRGRGCRWYSTWGYSR 22680
Indonesia-88035     ACCRGRAAAAWCKRRCCYGAARGARRTRTMASYCYMSCRCAMTGGRGRACRWYSTWGYSR 22680
Indonesia-88045     ACCRGRAAAAWCKRRSCYGAARGARRTRTMASYCYMSCRCAMTGGRGRACRWYSTWGYSR 22680
Indonesia-88065     ACCRGRAAAAWCKGRSCYGAARGARATRTMASYCYMSCRTAMTGGRGRGCRWYSTWGYSR 22680
                     . * * .: *.  *.      .   .*   .* * .          *.    .  .*.

Bhutan-09015        YTRYWAMSSGRTYRWAAYCGYYTGGCRGTGTARTGTGRKCRRYGWTYYRCSAYCRYRAYT 22740
Bhutan-09024        YTRYWAMSSGRTYRWAAYCGYYTGGCRGTGTARTKYGRKCRRYGWTYYRCSAYCRYRAYT 22740
Bhutan-09027        YTRYWAMSSGRTCAWAAYCGYYTGGCRGTGTARTKYGRKCRRYGWTYYRCSAYCRYRAYT 22740
Bhutan-09030        YTRYWAMSSGRTYRWAAYCGYYTGGCRGTGTARTKYGRKCRRYGWTYYRCSAYCRYRAYT 22740
Bhutan-09005        CKGTWRASSKAKCAAMGYYAYCYKRYARYRTARCKYKRKYGATKTKCYGYGRTMRCRRYY 22740
Indonesia-88035     CKGTWRASSKAKCAAMGCYRYCTGRYARYRTARCGTKRKYGATKTKCYGYGRTMRCARYY 22740
Indonesia-88045     CKGTWRASSKAKCAAMGCYRYCTGRYARYRYMRCKYKRKYGATKTKCYGYGRTMRCARYY 22740
Indonesia-88065     CKGTWRASSKAKCAAMGYYAYCYKRYARYRTARCKYKRKYGATKTKCYGYGRTMRCRRYY 22740
                     .  *  **  .    .   *           *    **      . *  .   *   *

Bhutan-09015        YWRATRTAGWTGRAKAYWWRGKAYAWGAYARYYYTRMATKYRYWCGYKACATATAGYAYA 22800
Bhutan-09024        YWRATRTAGWTGRAKAYWWRGKAYAWGAYARYYYTRMATKYRYWCGYKACATATAGYAYA 22800
Bhutan-09027        YWRATRTAGWTGRAKAYWWRGKAYAWGAYARYYYTRMATKYRYWCGYKACWTATAGYAYA 22800
Bhutan-09030        YWRATRTAGWTGRAKAYWWRGKAYAWGAYARYYYTRMATKYRYWCGYKACATATAGYAYA 22800
Bhutan-09005        CAAMTGTTRTWARRKRYTAGRGWYWWKGYWAYTTYARGWKCRYAGKCKWMWYGWRCYMTT 22800
Indonesia-88035     CAAMYGKTRTWARRKRYTAGRGWYWWKGYWAYTTYARGWKCRYAGKCKWCWYGWGCYATT 22800
Indonesia-88045     CAAMYGKTRTWARRKRYTAGRGWYWWKGYWAYTTYARGWKCRYAGKCKWCWYGWRCYATT 22800
Indonesia-88065     CAAMTGTTRTWARRKRYTAGRGWYWWKGYWAYTTYARGWKCRYAGKCKWCWYGWGCYMTT 22800
                          .:   .* * *      * * .*  *     . * **    *    .   *  :

Bhutan-09015        MRKCAWRYYARKSYTYRWYGCWRKMACSMRYYSRARTTYRGGGRWWYTYTWACCSYCCAT 22860
Bhutan-09024        MRKCAWRYYARKSYTYRWYGCWAKMACSMRYYSRARTTYRGGGRWWYTYTWACCSYCCAT 22860
Bhutan-09027        MRKCAWRYYARKSYTYRWYGCWAKMACSMRYYSRARTTYRGGGRWWYTYTWACCSYCCAT 22860
Bhutan-09030        MRKCAWRYYARKSYTYRWYGCWAKMACSMRYYSRARTTYRGGGRWWYTYTWACCSYCCAT 22860
Bhutan-09005        AAGYRWGTTARTGYYCRWYATRRKAMGSMATTSGRAAKYGKRRATWTYTKTRGSCTYTRY 22860
Indonesia-88035     AAGYRWGTTRRTGYYCRWYATRATAMCSMATTSGRAWKYGKRRATWTYTKTRGSCTYYRT 22860
Indonesia-88045     AAGYRWGTTRRTGYYCRWYATRATAMCSMATTSGRAWKYGKRRATWTYTKTRGSCTYYRT 22860
Indonesia-88065     AAGYRWGTTARTGYYCRWYATRRKAMGSMATTSGRAAKYGKGGATWTYTKTRGSCTYTRY 22860
                         *    *..*  ***.   .   **   *    .*      *   .   ..

Bhutan-09015        GKRRKGRRRMWGARYAMGMTWRAAMSYRGYMGTRYWYYWRARTAASKKRYCWSKCGCCAM 22920
Bhutan-09024        GKRRKGRRRMWGARYAMGMTWRAAMSYRGYMGTRYWYYWRARTAASKKRYCWSKCGCCAM 22920
Bhutan-09027        GKRRKGRRRMWGARYAMGMTWRAAMSYRGYMGTRYWYYWRARTAASKKRYCWSKCRYCAM 22920
Bhutan-09030        GKRRKGRRRMWGARYAMGMTWRAAMSYRGYMGTRYWYYWRARTAASKKRYCWSKCGCCAM 22920
Bhutan-09005        RKGAKRRRRAAGMACMCKATAARRCCYGACCCCRTWCTTRMGKWWSTKGTSTSTYGYMWM 22920
Indonesia-88035     RKRAKRRRRAAKMACMCKACAARRCSYGACCCCRTWCTTRMGKWWSTKGTSTSTYGYMWM 22920
Indonesia-88045     RKRAKRRRRAAKMACMCKACAARRCSYGACCCCRTWCTTRMGKWWSTKGTSTSTYGYMWM 22920
Indonesia-88065     RKGAKRRRRAAGMACMCKATAARRCCYGACCCCRTWCTTRMGKWWSTKGTSTSTYGYMWM 22920
                     *  * ***                .* .    * *   *  .  *.*  . *.     *

Bhutan-09015        YKYRACYSAGGKAGAAATYKKSKRKWYTGCYGKGCSYYMWYMYSTAASRRRGTRKYRCRM 22980
Bhutan-09024        YKYRACYSAGGKAGAAATYKKSKRKWYTGCYGKGCSYYMWYMYSTAASRRRGTRKYRCRM 22980
Bhutan-09027        YKYRACYSAGGKAGAAATYKKSKRKWYTGCYGKGCSYYMWYMYSTAASRRRGTRKYRCRM 22980
Bhutan-09030        YKYRACYSAGGKAGAAATYKKSKRKWYTGCYGKGCSYYMWYMYSTAASRRRGTRKYRCRM 22980
Bhutan-09005        TKCRRTYCRARGCAWGRWYTTSGGGWCWKYCRKTGCTTAWYCCCKRWCGGRRTRKCRTAA 22980
Indonesia-88035     TKCRRTYCRARGCRWGAWYTTSGGGWYWKYCRKTGCTTAWYCCCKRWSGGRRCAKCRTAA 22980
Indonesia-88045     TKCRRTYCRARGCRWGAWYTTSGGGWYWKYCRKTGCTTAWYCCCKRWSGGRRCAKCRTAA 22980
Indonesia-88065     TKCRRTYCRARGCAWGRWYTTSGGGWCWKYCRKTGCTTAWYCCCKRWCGGRRTRKCRTAA 22980
```

```
                          * *  *. .   .   .  *..*    *       *  .    **  ..  .   *    * *

Bhutan-09015              RAGGTCGAYRASATMCGSRCMRYTKRKMGCRTYYRGGAKRRRGYYWGARTAAGTGRARAM 23040
Bhutan-09024              RAGGTCGAYRASATMCGSRCMRYTKRKMGCRTYYRGGAKRRRGYYWGARTAAGTGRARAM 23040
Bhutan-09027              RAGGTCRAYRASATMCGSRCMRYTKRKMGCRTYYRGGAKRRRGYYWGARTAAGTGRARAM 23040
Bhutan-09030              RAGGTCGAYRASATMCGSRCMRYTKRKMGCRTYYRGGAKRRRGYYWGARTRAGTGRARAM 23040
Bhutan-09005              RAKSGARRYRRGRYAAAGATMACYKATCRMATCTGKRWKRAAKYCTCRATAAGGARMRRC 23040
Indonesia-88035           RAKSGARRYRRGRYAAAGATAACYGATCRMAYCTGKRWTAAAKYCTCRAWRWRGARMRRC 23040
Indonesia-88045           RWKSGARRCRRGRYAAAGATAACYGATCRMAYCTGKRWTAAAKYCTCRAWAARGARMRRC 23040
Indonesia-88065           RTKSGARRYRRGRYAAAGATMACYKATCRMAYCTGKRWKRAAKYCTCRATRWGGARMRRC 23040
                          *  . .   * .   ...        .           .    *          .* *

Bhutan-09015              KCTCKWTTTGRRTSRYYKYSMRTMWTYRRWYRGYYYGKYWYYYKTAKMYGCRAKWMTAAS 23100
Bhutan-09024              KCTCKWTTTGRRTSRYYKYSMRTMWTYRRWYRGYYYGKYWYYYKTAKMYGCRAKWMTAAS 23100
Bhutan-09027              KCTCKWTTTGRRTSRYYKYSMRTMWTYRRWYRGYYYGKYWYYYKTAKMYGCRAKWMTAAS 23100
Bhutan-09030              KCTCKWTTTGRRTSRYYKYSMRTMWTYRRWYRGYYYGKYWYYYKTAKMYGCRAKWMTAAS 23100
Bhutan-09005              TMYYTTAYTGAATGGCCKTSMGYCAWCAGTTGRCTTKTYWYTYGYRKMTTTATKWMYMWS 23100
Indonesia-88035           TMYYTWAYYRRAYGGCCGTSMGYCAWCAGKTGRCTTGTCWYTYGCRKMTTTATKWMYAAS 23100
Indonesia-88045           TMYYTWAYYRRAYGGCCKTSMGYCAWCAGKTGRCTTGTCWYTYGCRKMTTTATKWMYAAS 23100
Indonesia-88065           TMYYTTAYTGAATGGCCKTSMGYCAWCAGKTGRCTTKTYWYTYGYRKMTTTATKWMYMWS 23100
                          .   . :      .     **                 . ** *    **     :***    *

Bhutan-09015              TYTYYRYATGTGYTARYRGACTYRRWYYRCKCTGCYGTSMWCGKGCCGSTRWGYWWCAAA 23160
Bhutan-09024              TYTYYRYATGTGYTARYRGACTYRRWYYRCKCTGCYGTSMWCGKGCCGSTRWGYWWCAAA 23160
Bhutan-09027              TYTYYRYATGTGYTARYRGACTYRRWYYRCKCTGCYGTSMWCGKGCCGSTRWGYWWCAAA 23160
Bhutan-09030              TYTYYRYATGTGYTARYRGACTYRRWYYRCKCTGCYGTSMWCGKGCCGSTRWGYWWCAAA 23160
Bhutan-09005              YYGTTRYAKSWRCCMRCRTGYYCRGWYYGYGYKRYCGYCCTTSGGCYSGYAWRYWTSCRR 23160
Indonesia-88035           YYGTTRYRKSWRCCMRCRTGYYCRGWYYGYGYKRCCSYCCTTSGRMYSGYAWRYWTSCRR 23160
Indonesia-88045           YYGTTRYRKSWRCCMRCRTGYYCRGWYYGYGYKRCCSYCCTTSGRMYSGYAWRYWTSCRR 23160
Indonesia-88065           YYGTTRYAKSWRCCMRCRTGYYCRGWYYGYGYKRYCGYCCTTSGGCYSGYAWRYWTSCRR 23160
                           *   ** ..     * * .   * ***     .    . .    .     ..  * ** ..

Bhutan-09015              GMRAAAAKCGCSKMRRRKMKTWWRASTCATGMATRMSYYAMWSKTKRCCTYRTGTCRYYC 23220
Bhutan-09024              GMRAAAAKCGCSKMRRRKMKTWWRASTCATGMATRMSYYAMWSKTKRCCTYRTGTCRYYC 23220
Bhutan-09027              GMRAAAAKCGCSKMRRRKMKTWWRASTCATGMATRMSYYAMWSKTKRCCTYRTGTCRYYC 23220
Bhutan-09030              GMRAAAAKCGCSKMRRRKMKTWWRASTCATGMATRMSYYAMWSKTKRCCTYRTGTCRYYC 23220
Bhutan-09005              ACAMRCAKCRYSKMRAGGCTYTWRWCKYRATARYAMSYYACWCGTKRSYWTAYAYYRCTC 23220
Indonesia-88035           AAAAACWKYAYCGMRAGGCTYTWRWSKYGATARYAMSYCWCWCKWKRSYWTAYAYYRCTY 23220
Indonesia-88045           AAAAACWKYAYCGMRAGGCTYTWRWSKYGATARYAMSYCWCWCKWKRSYWTAYAYYRCTY 23220
Indonesia-88065           ACAMRCAKCRYSKMRAGGCTYTWRWCKYRATARYAMSYYACWCGTKRSYWTAYAYYRCTC 23220
                          .     . *    . **     .  ** ..  :      ***    *.  **.      .  *

Bhutan-09015              CYWTCSTRACYCYGRAMCYKRYWMTGRWWAGRWARCACGTCMRYRGTGYTYCACTYRGCW 23280
Bhutan-09024              CYWTCSTRACYCYGRAMCYKRYWMTGRWWAGRWARCACGTCMRYRGTGYTYCACTYRGCW 23280
Bhutan-09027              CYWTCSTRACYCYGRAMCYKRYWMTGRWWAGRWARCACGTCMRYRGTGYTYCACTYRGCW 23280
Bhutan-09030              CYWTCSTRACYCYGRAMCYKRYWMTGRWWAGRWARCACGTCMRYRGTGYTYCACTYRGCW 23280
Bhutan-09005              STWTCGTMGMTGCGRRCTYKRYWCYARTARSAATRYRMGTSCRYGKCTCYTYRMWYAAYW 23280
Indonesia-88035           CTWKYGKMAMTSCRRRCTYKRYWCYAGTARSAATRYRMRYSCRYGKCTCYTYRMWYAAYW 23280
Indonesia-88045           CTWKYGKMAMTSCRRRCTYKRYWCYARTARSAATRYRMRYSCRYGKCTCYTYRMWYAAYW 23280
Indonesia-88065           STWTCGTMGMTGCGRRCTYKRYWCYARTAGSAATRYRMGTSCRYGKCTCYTYRMWYAAYW 23280
                          . *. .. .      *    *****   .     .  :*      . **             * . *

Bhutan-09015              WYWMYRKAGGYTGYGRTKRYYCYRMKWYTRCGWTYRKWRRARKAYYYSCRRARSRCYWMT 23340
Bhutan-09024              WYWMYRKAGGYTGYGRTKRYYCYRMKWYTRCGWTYRKWRRARKAYYYSCRRARSRCYWMT 23340
```

```
Bhutan-09027          WYWMYRKAGGYTGYGRTKRYYCYRMKWYTRCGWTYRKWRRARKAYYYSCRRARSRCYWMT 23340
Bhutan-09030          WYWMYRKAGGYTGYGRTKRYYCYRMKWYTRCGWTYRKWRRARKAYYYSCRRARSRCYWMT 23340
Bhutan-09005          TTWMTGTRSRTWSYGGWKRCYSYRATATAAYSWAYAGAGRMAKWYYTGAAAWRCRTTWCT 23340
Indonesia-88035       WYWMTGTRSRTWSYKGWKRCYSYRATATAAYSWAYAGAGRAAKWYYTGAAAARCRTTWCY 23340
Indonesia-88045       WYWMTGTRSRTWSYKGWKRCYSYRATATAAYSWAYAGAGRAAKWYYTGAAAARCRTTWCY 23340
Indonesia-88065       TTWMTGTASRTWSYGGWKRCYSYRATATAAYSWAYAGAGRMAKWYYTGAAAWRCRTTWCT 23340
                        **  . .   .*   ** *.** .  :  .*:*    *  * ** ..   *.*  *

Bhutan-09015          YASYCWWMKKKAKTGGCRTGMRCYACSMYKYMTCAYRYYTYMKYCWAYARWRAYYRYRKC 23400
Bhutan-09024          YASYCWWMKKKAKTGGCRTGMRCYACSMYKYMTCAYRYYTYMKYCWAYARWRAYYRYRKC 23400
Bhutan-09027          YASYCWWMKKKAKTGGCRTGMRCYACSMYKYMTCAYRYYTYMKYCWAYARWRAYYRYRKC 23400
Bhutan-09030          YASYCWWMKKKAKTGGCRTGMRCYACSMYKYMTCAYRYYTYMKYCWAYARWRAYYRYRKC 23400
Bhutan-09005          YCGYYTWAKKTRGKRRYRCRARYCMTSMYTCCTCWCRTTKYMTCTWRYMRWRGCTACRGS 23400
Indonesia-88035       YCGYYTWAKKTRGKRGCRCRARYCMTSMYTCCYSWCRTTKYMTCTWRYMRWRGCTACRGS 23400
Indonesia-88045       YCGYYTWAKKTRGKRGCRCRARYCMTSMYTCCYSWCRTTKYMTCTWRYMRWRGCTACRGS 23400
Indonesia-88065       YCGYYTWAKKTRGKRRYRCRARYCMTSMYTCCTCWCRTTKYMTCTWRYMRWRGCTACRGS 23400
                      *..*  * **.  .   *   *    ***.   .  *  .**.  * * ***.    * .

Bhutan-09015          TYAYYGRGWYTMRYSRGGGAYRYRCMRAGWKYYSTYMKAKYRYYMTYYYWACCARMYMKA 23460
Bhutan-09024          TYAYYGRGWYTMRYSRGGGAYRYRCMRAGWKYYSTYMKAKYRYYMTYYTWACCARMYMKA 23460
Bhutan-09027          TYAYYGRGWYTMRYSRGGGAYRYRCMRAGWKYYSTYMKAKYRYYMTYYYWACCARMYMKA 23460
Bhutan-09030          TYAYYGRGWYTMRYSRGGGAYRYRCMRAGWKYYSTYMKAKYRYYMTYYTWACCARMYMKA 23460
Bhutan-09005          KYRYYKGKWYYMGYSGRGKRYGYAYMRRKAKTYCKTCKMGTGCTMYYYTTGSYWAMTMWR 23460
Indonesia-88035       KYGYTKGKWYYMGYSGRKKRYGYAYMRRKAKTYCKTMKAGTGCYMTYYYTGSYWAMTMWR 23460
Indonesia-88045       KYGYTKGKWYYMGYSGRKKRYGYAYMRRKAKTYCKTCKAGTGCYMTYYYTGSYWAMTMWR 23460
Indonesia-88065       KYRYYKGKWYYMGYSGRGKRYGYAYMRRKAKTYCKTCKMGTGCTMYYYYTGSYWAMTMWR 23460
                      .* *    ** * **     * *  **   * *..  *      * **  ..   * *

Bhutan-09015          YRGTSYRTCWRRMYMSTYTRGCRYRYRAWGTRRRCGWARRAWRAYCYWGRRYYKCARYKY 23520
Bhutan-09024          YRGTSYRTCWRRMYMSTYTRGCRYRYRAWGTRRRCGWARRAWRAYCYWGRRYYKCARYKY 23520
Bhutan-09027          YRGTSYRTCWRRMYMSTYTRGCRYRYRAWGTRRRCGWARRAWRAYCYWGRRYYKCARYKY 23520
Bhutan-09030          YRGTSYRTCWRRMYMSTYTRGCRYRYRAWGTRRRCGWARRAWRAYCYWGRRYYKCARYKY 23520
Bhutan-09005          TGRACCAYYTARMCAGYTWGRMATRCGWTRKAAACAWWRAGARTTYTTAAGTCGYRGYKT 23520
Indonesia-88035       TGRACCACYTAGMYAGYTWGAMATACGWTRKAAAYAWARRGARTTYTTAAGTCGYRGTKT 23520
Indonesia-88045       TGRACCAYYTAGMYAGYTWGAMATRCGWTRKAAAYAWARRGARTTYTTARGTCGYRGTKT 23520
Indonesia-88065       TGRACCAYYTARMCAGYTWGRMATRCGWTRKAAACAWARAGARTTYTTARGTCGYRGYKT 23520
                         :.       *  .              .    .* * . *:    .         *

Bhutan-09015          SYTTRRYRSYYRMKAYYTMATYYARSRARKTTCRYTMCRCRKKAWGMKGKYSTWRCRRRG 23580
Bhutan-09024          SYTTRRYRSYYRMKAYYTMATYYARSRARKTTCRYTMCRCRKKAWGMKGKYSTWRCRRRG 23580
Bhutan-09027          SYTTRRYRSYYRMKAYYTMATYYARSRARKTTCRYTMCRCRKKAWGMKGKYSTWRCRRRG 23580
Bhutan-09030          SYTTRRYRSYYRMKAYYTMATYYARSRARKTTCRYTMCRCRKKAWGMKGKYSTWRCRRRG 23580
Bhutan-09005          GYYYRRTGGCCRAKRTYYARYYYRRSATGKWKAGTYCTGYGGGMARAGCKYSGWGSRARR 23580
Indonesia-88035       GYYYRRTGGCCRAKATYYARYYYRRSATGKWKAGTYCTGYGGGMWGMGCKYSGWGSRARR 23580
Indonesia-88045       GYYYRRTGGCCRAKATYYARYYYRRSATGKWKAGTYCTGYGGGMWGMGCKYSGWGSRARR 23580
Indonesia-88065       GYYYRRTGGCCRAKRTYYARYYYRRSATGKWKAGTYCTGYGGGMARAGCKYSGWGSRARR 23580
                      .*  **  .  * *  *    ** **  : * ..               *** * .* *

Bhutan-09015          CKKGCTTAGGTMKTRRSSWYGCYSCCRRYGWMWGGCWCWCYMRTTYKKWYRRRAYATYYM 23640
Bhutan-09024          CKKGCTTAGGTMKTRRSSWYGCYSCCRRYGWMWGGCWCWCYMRTTYKKWYRRRAYATYYM 23640
Bhutan-09027          CKKGCTTAGGTMKTRRSSWYGCYSCCRRYGWMWGGCWCWCYMRTTYKKWYRRRAYATYYM 23640
Bhutan-09030          CKKGCTTAGGTMKTRRSSWYGCYSCCRRYGWMWGGCWCWCYMRTTYKKWYRRRAYATYYM 23640
Bhutan-09005          CKKRCTTMRGWATYAASKTTSSCSTTRGCRTAARRSWSWMCMATACKKWCGGGRYRTYYC 23640
Indonesia-88035       YGKRYYWMRRWATYAASKTTSSCSTTRGCRTAARRSWSWMCMAWACKKWCKGGRYRYYYC 23640
```

```
Indonesia-88045      YGKRYYWMRRWATTAASKTTSSCSTTRGCRTAARRSWSWMCMAWACKKWCKGGRYRYYYC 23640
Indonesia-88065      CKKRCTTMRGWATYAASKTTSSCSTTRGCRTAARRSWSWMCMATACKKWCGGGRYRTYYC 23640
                       *         .   *.  .. *  *        .*.*  *  : ***     *  **

Bhutan-09015         TRWARAWRTYCYAYSWRRRMKTATRYACMGARYAGGGTYRCATAKRYTARATRRGYTCYA 23700
Bhutan-09024         TRWARAWRTYCYAYSWRRRMKTATRYACMGARYAGGGTYRCATAKRYTARATRRGYTCYA 23700
Bhutan-09027         TRWARAWRTYCYAYSWRRRMKTATRYACMGRRYAGGGTYRCATAKRYTARATRRGYTCYA 23700
Bhutan-09030         TRWARAWRTYCYAYSWRRRMKTATRYACMGRRYAGGGTYRCATAKRYTARATRRGYTCYA 23700
Bhutan-09005         WAWMGMWRYYYTRTSARAGMGYRYRCRYCGAGTRSRSYYRARCGKAYCCGRKARRYKMYR 23700
Indonesia-88035      WAWMGMWRYYYTRTSARAGMGYRYRCRYCGRGTRSRSYYRAACGKAYCCGRKARRYKMYR 23700
Indonesia-88045      WAWMGMWRYYYTRTCARAGMGYRYRCRYCGRGTRSRSYYRAACGKAYCCGRKARRYKMYR 23700
Indonesia-88065      WAWMGMWRYYYTRTSARAGMGYRYRCRYCRRGTRSRSYYRMRCGKAYCCGRKARRYKMYR 23700
                       *   ** *    . *  *    *         . . **   .* * .  . * *. *

Bhutan-09015         KGWTYSCMCRYYRYSYYYAWTRMCRCSWTYCRYTKWYCYGWGYGGGTCSWRRRAAGSRCW 23760
Bhutan-09024         KGWTYSCMCRYYRYSYYYAWTRMCRCSWTYCRYTKWYCYGWGYGGGTCSWRRRAAGSRCW 23760
Bhutan-09027         KGWTYSCMCRYYRYSYYYAWTRMCRCSWTYCRYTKWYCYGWGYGGGTCSWRRRAAGSRCW 23760
Bhutan-09030         KGWTYSCMCRYYRYSYYYAWTRMCRCSWTYCAYTKWYCYGWGYGGGTCSWRRRAAGSRCW 23760
Bhutan-09005         TRAWYSSAYATTATCTTTRAKAASGTSACTAGYYKTTYYSACYSGGTSSAARRRRSSRSW 23760
Indonesia-88035      TRAWYSSMYATTATCCTTRAKAASGTSACTAGYYKTTYYSACCSRKYSSAARRRRSSRSW 23760
Indonesia-88045      TRAWYSSMYATTATCCTTRAKAASGTSACTAGYYKTTYYSACCSRKYSSAARRAACSRSW 23760
Indonesia-88065      TRAWYSSAYATTATCTTTRAKAASGTSACTAGYYKTTYYSACYSGGTSSAARRRRSSRSW 23760
                     .   **.       .     .  .  *   . * *   *.   .   .*  **   **.*

Bhutan-09015         SAYRWYYYYMAYYGYRSKTWYSRSYASWRGAYRMGRCTTAMCKSYRARCRRTYSRSGYYY 23820
Bhutan-09024         SAYRWYYYYMAYYGYRSKTWYSRSYASWRGAYRMGRCTTAMCKSYRARCRRTYSRSGYYY 23820
Bhutan-09027         SAYRWYYYYMAYYGYRSKTWYSRSYASWRGAYRMGRCTTAMCKSYRARCRRTYSRSGYYY 23820
Bhutan-09030         SAYRWYYYYMAYYGYRSKTWYSRSYASWRGAYRMGRCTTAMCKSYRARCRRTYSRSGYYY 23820
Bhutan-09005         CWMGWYYTYMMTTRYRGKWWTGGSYRGWGRRYRARRSYWRACKGYRRGTGRGYCRGRTCT 23820
Indonesia-88035      CWMGWYYTYMMTTRYRGKWWTGGSYRGWGRRYRARRSYWRAMKGYRRGTGRGYCAGRTCT 23820
Indonesia-88045      CWMGWYYTYMMTTRYRGKWWTGGSYRGWGRRYRARRSYWRAMKGYRRGTGRGYCAGRTCT 23820
Indonesia-88065      CWMGWYYTYMMTTRYRGKWWTGGSYRGWGRRYRARRSYWRACKGYRRGTGRGYCAGRTCT 23820
                     .   *** **    **.* * . ** .*   **  *.     *.**    * *. .

Bhutan-09015         YTWTAYRKRKSARYYYGGGAYYRYCRRRRYCTMGMRMWYGAAYGGGGYASCMRYWTKTTT 23880
Bhutan-09024         YTWTAYRKRKSARYYYGRGAYYRYCRRRRYCTMGMRMWYGAAYGGGGYASCMRYWTKTTT 23880
Bhutan-09027         YTWTAYRKRKSARYYYGGGAYYRYCRRRRYCTMGMRMWYGAAYGGGGYASCMRYWTKKYT 23880
Bhutan-09030         YTWTAYRKRKSARYYYGRGAYYRYCRRRRYCTMGMRMWYGAAYGGGGYASCMRYWTKTTT 23880
Bhutan-09005         CAWKRCAKAGSWAYYYSGGMTTACSARRRTSYMRMGCACRRRYSGSRYRSYMGYAYKTTY 23880
Indonesia-88035      CAWKRCAKAGSWAYYYSGGMTTAYSARRRTSYMRMGCACRRRYSRSRYRSYMGYAYKTTY 23880
Indonesia-88045      CAWKRCAKAGSWAYYYSGAMTTAYSARRRTSYMRMGCACRRRYSRSRYRSYMGYAYKTTY 23880
Indonesia-88065      CAWKRCAKAGSWAYYYSGGMTTACSARRRTSYMRMGCACRRRYSGSRYRSYMGYAYKTTY 23880
                      :*.   *  *  ***. .     . *** . *       *. . * * * *  *.

Bhutan-09015         MRYYYWTTYTTAYRAWWYTCYRTAAAGYYRSCKTRYRTRYSYTTRMRRRYYATGTSRSRK 23940
Bhutan-09024         MRYYYWTTYTTAYRAWWYTCYRTAAAGYYRSCKTRYRTRYSYTYRMRRRYYATGTSRSRK 23940
Bhutan-09027         MRYYYWTTYTTAYRAWWYTCYRTAAAGYYRSCKTRYRTACSYTYRMRRRYYATGTSRSRK 23940
Bhutan-09030         MRYYYWTTYTTAYRAWWYTCYRTAAAGYYRSCKTRYRTRYSYTYRMRRRYYATGTSRSRK 23940
Bhutan-09005         MGTTCAKKTYWRTGRAWYYYYRCWMMTYTRCYKYRYRYRTSYGYGARRATTMWKKSRSGG 23940
Indonesia-88035      MGTTCAKKTYWRTGRAWYYYYRCWMMTYTRCYKYRYRYRTSYGYGARRATTMWKKSRSGG 23940
Indonesia-88045      MGTTCAKKTYWRTGRAWYYYYRCWMMTYTRCYKYRYRYRTSYGYGARRATTMWKKSRSGG 23940
Indonesia-88065      MGTTCAKKTYWRTGRAWYYYYRCWMMTYTRCYKYRYRYRTSYGYGARRATTMWKKSRSGG 23940
                     *     ..        **  **     * *. * ***   **    **      .***
```

```
Bhutan-09015        CYYYATYYGAKSYTMKAMWYCRSARRGSRAWSTGGAAATARSCRCKWKRRRAYRKRYYYT 24000
Bhutan-09024        CYYYATYYGAKSYTMKAMWYCRSARRGSRAWSTGGAAATARSCRCKWKRRRAYRKRYYYT 24000
Bhutan-09027        CYYYATYYGAKSYTMKAMWYCRSARRGSRAWSTGGAAATARCCRCKTGAGAAYRKRYYYT 24000
Bhutan-09030        CYYYATYYGAKSYTMKAMWYCRSARRGSRAWSTGGAAATARSCRCKTGAGAAYRKRYYYT 24000
Bhutan-09005        TCYCMCYYKRTSTKATGCATYRSRARGSRGACCCACTRWCRGTRCTTGAGACYRKATTYY 24000
Indonesia-88035     TCYCMCYCKRTSTKATGCATYRSGARSSRGACCCACTRWCRGTRCTWKRGACYRKATTYY 24000
Indonesia-88045     TCYCMCYCKRTSTTATGCATYRSRARSSRGACCCACTRWCRGTRMTWKRGACYRKATTYY 24000
Indonesia-88065     TCYCMCYCKRTSTTATGCATYRSRARGSRGACCCACTRWCRGTRMTWKRGACYRKATTYY 24000
                      *   *   .* . ..     **  *.**. .  ..:  .*  * .     .***   *

Bhutan-09015        WRRRTYAGYYKYARMWYTYCKRYACSTACGAGMYTACCRYRKARKTTAAASRYRYSWGRY 24060
Bhutan-09024        WRRRTYAGYYKYARMWYTYCKRYACSTACGAGMYTACCRYRKARKTTAAASRYRYSWGRY 24060
Bhutan-09027        WRRRTYAGYYKYARMWYYYCKRYACSTAYKRGMYTACCRYRKARKTTAAASRYRYSWGRY 24060
Bhutan-09030        WRRRTYAGYYKYARMWYYYCKRYACSTAYKRGMYTACCRYRKARKTTAAASRYRYSWGRY 24060
Bhutan-09005        TAGRYYRRYYKTRRATTYCTGGYRTGCTCGARYTCGYARYATRRGCCGCWSRYRTSWKAC 24060
Indonesia-88035     TAGRYYRRCCKTRRATTYCTGGYRTGCTCGARYTCGYARYATRRGCCGCWSRYRTSWKAC 24060
Indonesia-88045     TAGRYYRRCCKTRRATTYCTGGYGTGCTCGARYTCGYAGTATRRGCCGCWSRYRTSAKAC 24060
Indonesia-88065     TAGRYYRRCCKTRRATTYCTGGYRTGCTCGARYTCGYAGTATRRGCCGCWSRYRTSWKAC 24060
                       * *    *  *        *  . :       . .   . *   .. **** *

Bhutan-09015        SRRYCCKGCTYYRRKWGRRTRMKYTACCAYRRYYYCCTYKKASMTTAAAGRARKRAYYRA 24120
Bhutan-09024        SRRYCCKGCTYYRRKWGRRTRMKYTACCAYRRYYYCCTYKKASMTTAAAGRARKRAYYRA 24120
Bhutan-09027        SRRYCCKGCTYYRRTWGRRTRMKYTACCAYRRYYYCCTYKKASMTTAAAGRARKRAYYRR 24120
Bhutan-09030        SRRYCCKGCTYYRRTWGRRTGMKYTACCAYRRYYYCCTYKKASMTTAAAGRARKRAYYRA 24120
Bhutan-09005        GGACMTKSSYCYRRKWARGCAMGTCGMYGTGAYYYYMWYGTRGACAGCCAAMAGRRCCRG 24120
Indonesia-88035     GGACMTKSSYCYRRKWARGCAMGTCGMYGTGAYYYYMWYGTRGACAGCCAAMAGRRCCRG 24120
Indonesia-88045     GGACMTKSSYCYRGKWARGCAMGTCGMYGTGAYYYYMWYGTRGACAGCCAAMAGRRCCRG 24120
Indonesia-88065     GGACMTKSSYCYRRKWARGCAMGTCGMYGTGAYYYYMWYGTRGACAGCCAAMAGRRCCRG 24120
                    .     *..  ** .*.*   *   .  .   ***   * . .  :....    *   *

Bhutan-09015        ACSTMSRYTAATGYYSYRYCGAAYYSRYGSWYCSRATCGCRTAAAARCKACCYGRRWRCS 24180
Bhutan-09024        ACSTMSRYTRWTGYYSYRYCGAAYYSRYGSWYCSRATCGCRTAAMRRCKACCYGRRWRCS 24180
Bhutan-09027        RCSTMSRYTAATGYYSYRYCGAAYYSRYGSWYCSRATCGCRTAAMRRCKACCYGRRWRCG 24180
Bhutan-09030        ACSTMSRYTAATGYYSYRYCGAAYYSRYGSWYCSRATCGCRTAAMRRCKACCYGRRWRCS 24180
Bhutan-09005        AYCWASAYYAACACTGCRTYSRTTCGRYRGTCYSGWYYATGYRRCRGYWCYYTTRGWGTS 24180
Indonesia-88035     ATCWASAYYAACACTGCRTYSRTTCGRYRGTCYSGWYYATGYRRCRGYWCYYTTRGWGTS 24180
Indonesia-88045     ATCWASAYYAACACTGCRTYSRTTCGRYRGTCYSGWYYATGYRRCAGYWCYYTTRGWGTG 24180
Indonesia-88065     ATCWASAYYAACACTGCRTYSRTTCGRYRGTCYSGWYYATGYRRCRGYWCYYTTRGWGTS 24180
                      .  * *    .  . *  . :  .** .   *    .          .    * *  .

Bhutan-09015        YYYCAGTGRTMYKWSRYTRKCAYYYTGCRCRTCSGCCYYRWARCYRRRCGGRAWYYTTKC 24240
Bhutan-09024        YYYCAGTGRTMYKWSRYTRKCAYYYTGCRCRTCSGCCYYRWARCYRRRCGGRAWYYTTKC 24240
Bhutan-09027        TYYCAGTGRTMYKWSRYTRKCAYYYTGCRCRTCSGCCYYRWARCYRRRCGGRAWYYTTKC 24240
Bhutan-09030        YYYCAGTGRTMYKWSRYTRKYAYYYTGCRCRTCSGCCYYRWARCYRRRCGGRAWYYTTKC 24240
Bhutan-09005        YYCCGRYTRWCYTTGRYYRTCAYCCKRYRGGYYSKYYYTGWRRTYRARTRGGRTTYKWTS 24240
Indonesia-88035     YYCYGAYTRWCYTTGRYYRTCAYCCKRYRGGYYSKYYYTGTRRTYRARTRRGRTTYKWTS 24240
Indonesia-88045     TYCCGAYTRWCYTTGRYYRTCWYCCKRYRGGYYSKYYYTGTRRTYRARTRRGRTTYKWTS 24240
Indonesia-88065     YYCCGRYTRWCYTTGRYYRTCWYCCKRYRGGYYSKYYYTGWRRTYRARTRGGRTTYKWTS 24240
                     *  .   *  *. .** *.  *  .  *    *   *    * ** *       *. ..

Bhutan-09015        YAYRKAKRRWTTYYRTGRARAYRRTRRMGYYCGTYKYYSCSRRRYWRRRWWRWCRMYRSG 24300
Bhutan-09024        YAYRKAKRRWTTYYRTGRARAYRRTRRMGYYCGTYKYYSCGRRRYWRRRWWRWCRMYRSG 24300
Bhutan-09027        YAYRKAKRRWTTYYRTGRARAYRRTRRMGYYCGTYKYYSCSRRRYWRRRWWRWCRMYRSG 24300
Bhutan-09030        YAYRKAKRRWTTYYRTGRARAYRRTRRMGYYCGTYKYYSCSRRRYWRRRWWRWCRMYRSG 24300
```

```
Bhutan-09005          TGYGGRTGRWGKYCAKRGRGGCRRYGGCGCCTAGYTYYGTCARAYTGGGWAGWYGMCRCR 24300
Indonesia-88035       YGYGGRKGRWGKYCAKRGRGGCRGYGGCACCTAGYTCYGTCARAYTGGGWAGAYGMCRCR 24300
Indonesia-88045       YGYGGRKGRWGKYCAKRGRGGCRGYGGCACCTAGYTYYGTCARAYTGGGWAGAYGMCRCR 24300
Indonesia-88065       TGYGGRTGRWGKYCAKRGRGGCRRYGGCGCCTAGYTYYGTCARAYTGGGWAGWYGMCRCR 24300
                       .*   . ** .*  .    . *     .   . *. *.   * *    *     * *.

Bhutan-09015          YGGYTRCTCYYCTRRCCGRRTCCAMGATGCRCTGYRMRWTGRTYYMAYTYWRRTRCKCRA 24360
Bhutan-09024          YGGYTRCTCYYCTRRCCGRRTCCAMGATGCRCTGYRMRWTGRTYYMAYTYWRRTRCKCRA 24360
Bhutan-09027          YGGYTGCTCYYCTRRCCGRRTCCMMGATGCRCTGYRMRWTGRTYYMAYTYWRRTRCKCRA 24360
Bhutan-09030          YGGYTRCTCYYCTRRCCGRRTCCMMGATGCRCTGYRMRWTGRTYYMAYTYWRRTRCKCRA 24360
Bhutan-09005          CGATYMYYYTCMKAGYARGGYYYCWSRYRYGGYRYAMRWWAAYTTCRYYCWARARYGTGR 24360
Indonesia-88035       CRATTAYCYTCMKAGYARGGYYYCWSRYRYGGYRYAMRWWAAYTTCRYYCWARWRYGTGR 24360
Indonesia-88045       CRAYTAYCYTCMKAGYARGGYYYCWSRYRYGGYRYAMRWWAAYTTCRYYCWARWRYGTGR 24360
Indonesia-88065       CGAYTAYYYTCMKAGYARGGYYYCWSRYRYGGYRYAMRWWAAYTTCRYYCWARWRYGTGR 24360
                        .         .   .        .        * *** .      *  * * *

Bhutan-09015          YWTTYCYCGRRTMYRTRGGWRGRGCRAGACMYYAKCRTYACKKYYRSYKKYARCRMCYKC 24420
Bhutan-09024          YWTTYCYCGRRTMYRTRGGWRGRGCRAGACMYYAKCRTYACKKYYRSYKKYARCRMCYKC 24420
Bhutan-09027          YWTTYCTCGRRTMYRTRGGWRGRGCRAGACMYYAKCRTYACKKYYRSYKKYARCRMCYKC 24420
Bhutan-09030          YWTTYCYCGRRTMYRTRGGWRGRGCRAGACMYYAKCRTYACKKYYRSYKKYARCRMCYKC 24420
Bhutan-09005          CTKYYSYYRRGYCTMCGRKTRRGRTRRTGTACYWKGAYYTSKKYTRCYKKCRGYACYTTS 24420
Indonesia-88035       CTKYYSYYRRGYCTMCGRKTRRGRTRRTGTACYWKGAYYTSKKYTRCYKKCRGYACYTTS 24420
Indonesia-88045       CTKYYSYYRRGYCTMCGRKTRRGRTRRTGTACTATGAYYTCTKYTRCYKKCRGYACYTTS 24420
Indonesia-88065       CTKYYSYYRRGYCTMCGRKTRRGRTRRTGTACYWKGAYYTCTKYTRCYKKCRGYACYTTS 24420
                        . *.   *          *    *  .     .   *:..** *.***        ..

Bhutan-09015          RTGGYRGAARYTWMWKSAGYARRRKGYTWYTGTYRATYRYCMYKRSYTRKAWGSTSCCAY 24480
Bhutan-09024          RTGGYRGAARYTWMWKSAGYARRRGGYTWYTGTYRATYRYCMYKRSYTRKAWGSTSCCAY 24480
Bhutan-09027          RTGGYRGAARYTWMWKSAGYARRRKGYTWYTGTYRATYRYCMYKRSYTRKAWGSTSCCAY 24480
Bhutan-09030          RTGGYRGAARYTWMWKSAGYARRRKGYTWYTGTYRATYRYCMYKRSYTRKAWGSTSCCAY 24480
Bhutan-09005          GKRRTAARWGTAACWGSRSCRRARGKYCWTCACCARYCATYMCGGGCTRTWTRCASYYRC 24480
Indonesia-88035       GKRRTAARWGTAACWGSRSCRRARGKYCWTCACCARYCATYMCGGGCYRTWTRCASYYRC 24480
Indonesia-88045       GKRRTAARWGTAACWGSRSCRRARGKYCWTCACCARYCATYMCGGGCYRTWTRCASYYRC 24480
Indonesia-88065       GKRRTAARWGTAACWGSRSCRRARGKYCWTCACCARYCATYMCGGGCTRTWTRCASYYRC 24480
                       .    .    :  * * .  * *  * *  .         *   .  *.   .:*

Bhutan-09015          WYMWWMSYMACYCAGYRGMRRTWWYYRWGRGRKCYCYYGKYYTRRRCCKWSYSGKTRGYK 24540
Bhutan-09024          WYMWWMSYMACYCAGYRGMRRTWWYYRWGRGRKCYCYYGKYYTRRRCCKWSYSGKTRGYK 24540
Bhutan-09027          WYMWWMSYMACYCAGYRGMRRTWWYYRWGRGRKCYCYYGKYYTRRRCCKWSYSGKTRGYK 24540
Bhutan-09030          WYMWWMSYMACYCAGYRGMRRTWWYYRWGRGAKCYCYYGKYYTRRRCCKWSYSGKTRGYK 24540
Bhutan-09005          WYMAAASYMCMTTRAYRRCARYTTYTATRGRRGMCTTTRKTYKARAYYTAGYSTKYRRYG 24540
Indonesia-88035       WYMAAASYMCMTTRAYRRCARYTTCTATRGRRGMCTTTRKTYKARAYYTAGYSTKYARYG 24540
Indonesia-88045       WYMAAASYMCMTTRAYRRCARYTTCTATRGRRGMCTTTRKTYKARAYYTAGYSTKCRAYG 24540
Indonesia-88065       WYMAAASYMCMTTRAYRGCAATTTTTATGGGAGMCTTTRKTYKARAYYTAGYSTKYRRYG 24540
                      ***   ***.    .**                      * *. *   . .** *   *

Bhutan-09015          TSGKMGTYMRATYCRYYKKMARRKWRARTKSYKYYYCCTCKTYYWWTRTMSYYGTKGYYA 24600
Bhutan-09024          TSGKMGTYMRATCCRYYKKMARRKWRARTKSYKYYYCCTCKTYYWWTRTMCCYGTKGYYA 24600
Bhutan-09027          TSGKMGTYMRATYCRYYKKMARRKWRARTKSYKYYYCCTCKTYYWWTRTMCCYGTKGYYA 24600
Bhutan-09030          TSGKMGTYMRATYCRYYKKMARRKWRARTKSYKYYYCCTCKTYYWWTRTMCCYGTKGYYA 24600
Bhutan-09005          YGRTARCCAGWWYCRCCKKCMGGTWRWGAGGTKYYTCYKTGYCCWWTAWCGTYKYKRYCR 24600
Indonesia-88035       YGRTARCCAGWWYYGCCKKCMGGTWRWGTGGTKYYCTYKYGYCCWWKAWCGTYKYKRYCR 24600
Indonesia-88045       YGRTARCCAGWWYYGCCKKCMGGTWRWGTGGTKYYCTYKYGYCCWWKAWCGTYKYKRYCR 24600
Indonesia-88065       YGRTARCCAGWWYCRCCKKCMGGTWRWGAGGTKYYTCYKTGYCCWWTAWCGTYKYKRYCR 24600
```

```
                      . .             **    .**  : . ***   .     **.     *  * *

Bhutan-09015         YWRYYSWAGWYTWARTRRKSSRYCACTYACRRTRTCGWGTAYYWYGGATKWYARYYKCKS 24660
Bhutan-09024         YWRYYSWAGWYTWARTRRKSSRYCACTYACRRTRTCGWGTAYYWYRGATKWYARYYKCKS 24660
Bhutan-09027         YWRYYSTAGWYTWARTRRKSSAYCACTYACRRTRTCGWGTAYYWYRGATKWYARYYKCKS 24660
Bhutan-09030         YWRYYSWAGWYTWARTRRKSSAYCACTYACRRTRTCGWGTAYYWYGGATKWYARYYKCKS 24660
Bhutan-09005         CWACYSAAKTTCWWAYAGGGCGYYTYYCGTRRWGYCAARYTYYWYGAGCTATGGYCTCGG 24660
Indonesia-88035      CTACTGAATTTCWWAYAGGGCGYYTYYYGTRRWGYYRARYTYYWYGGGCTTTGGYCTYGG 24660
Indonesia-88045      CTACTGWWTTTCWWAYAGGGCGYYTYYYGTRRWGYYRARYTYYWYGGGCTTTGGYCTYGG 24660
Indonesia-88065      CWACYSWWKTTCWWAYAGGGCGYYTYYCGTRRWGYYAARYTYYWYGAGCTATGGYCTCGG 24660
                          .      *      .. * :   . **        :**** .. .  . * .  .

Bhutan-09015         YRYWGTGKRYGATAYYCKYWGRCWWRYSGAARCSYMYMKYMGTYMTRYYSWYYTRCTCCA 24720
Bhutan-09024         YRYWGTGKRYGATAYYCKYWGRCWWRYSGAARCSYMYMKYMGTYMTRYYSWYYTRCTCCA 24720
Bhutan-09027         YRYWGTGKRYGATAYYCKYWGRCWWRYSGAARCSYMYMKYMGTYMTRYYSWYYTRCTCCA 24720
Bhutan-09030         YRYWGTGKRYGATAYYCKYWGRCWWRYSGAARCSYMYMKYMGTYMTRYYSWYYTRCTCCA 24720
Bhutan-09005         CGTWRARGRYKRAGTYGGCWGRMWTAYCRMRAACCMTMGCARKCATGYTCWYYCAGCGGR 24720
Indonesia-88035      TRTWGAGGRYKRAGTYGGCARGCTTACCRMRAACCMTMGCARKCAAGYTCTTCCAGCGGR 24720
Indonesia-88045      TRTWGAGGRYKRAGTYGGCARGMWTACCRMRAACCMTMGCARKCAAGYTCTTCCAGCGGR 24720
Indonesia-88065      CGTWRARGRYKRAGTYGGCWGRMWTAYCRMRAACCMTMGCARKCATGYTCWYYCAGCGGR 24720
                        * :  **  :. *           .    .. * *    .  : * .

Bhutan-09015         GYRYMSCTWMAYYYYKCGCTRKKYGYYTSKCYSYYWKYSCMCTYYRYMYYWCRRYYYRTG 24780
Bhutan-09024         GYRYMSCTWMAYYYYKCGCTRKKYGYYTSKCYSYYWKYSCMCTYYRYMYYWCRRYYYRTG 24780
Bhutan-09027         GYRYMSCTWMAYYYYKCGCTRKKYGYYTSKCYSYYWKYSCMCTYYGCMYYWCRRYYYRTG 24780
Bhutan-09030         GYRYMSCTWMAYYYYKCGCTRKKYGYYTSKCYSYYWKYSCMCTYYRYMYYWCRRYYYRTG 24780
Bhutan-09005         KYGCMSCYWMRYYTTKYRAKATGTRCYKSTMTGTTWKYCGAYWTCGCMCTATRRYYYRYG 24780
Indonesia-88035      KYGCMGMYTMRYTTTKYRAKATGTRCYKSTCTGTTWKYCGAYTTCGCMCTATRRYYYRYR 24780
Indonesia-88045      KYGCMGMYTMRYYTTKYRAKATGTRCYKSTCTGTTWKYCGAYTTCGCMCTATRRYYYRYR 24780
Indonesia-88065      KYGCMSCYWMRYYTTKYRAKATGTRCYKSTMTGTTWKYCGAYWTCGCMCTATRRYYYRYR 24780
                      *  *.   * *   *  .. .    *.*.  .  ***.        *    ******

Bhutan-09015         AGCKTYYYRSRRKCAWRRRYASATTTMSKGWTRYWYKRTRTKGYAGTRCGYGCRAGMMRA 24840
Bhutan-09024         AGCKTYYYRSRRKCAWRRRYASATTTMSKGWTRYWYKRTRTKGYAGTRCGYGCRAGMMRA 24840
Bhutan-09027         AGCKTYYYRSRRKCAWRRACASATTTMSKGWTRYWYKRTRTKGYAGTRCGYGCRAGMMRA 24840
Bhutan-09030         AGCKTYYYRSRRKCAWRRRYASATTTMSKGWTRYWYKRTRTKGYAGTRCGYGCRAGMMRA 24840
Bhutan-09005         RKTKYCCTRSRRGTTWRRACWCRYYYMGKATAGCTTKRGAWTSTMRYGYCYRCARSMCGR 24840
Indonesia-88035      RKTKYCCTRSRRGTTWRRACACAYYYMGKGTAGCTTKRGAWTCTMRYGYCYAYARSMMRR 24840
Indonesia-88045      RKTKYCCTRSRRGTTWRRACACAYYYMGKGTAGCTTKRGAWTCTMRYGYCYAYARSMMRR 24840
Indonesia-88065      RKTKYCCTRSRRGTTWRRACWCRYYYMGKATAGCTTKRGAWTSTMRYGYCYRCARSMCGR 24840
                        *    ****  :***   .    *.*. :    **   .        *    .*

Bhutan-09015         CGTCCCMYAGSYASRMYCGYTGRCRGMYGGMRRWSTACATSTMYTRCSSMGYRTTAYAKY 24900
Bhutan-09024         CGTCCCMYAGSYASRMYCGYTGRCRGMTGGMRRWSTACATSTMYTRCSSMGYRTTAYAKY 24900
Bhutan-09027         CGTCCCMYAGSYASRMYCGYTGRCRGMTGGMRRWSTACATSTMYTRCSSMGYRTTAYAKY 24900
Bhutan-09030         CGTCCCMYAGSYASRMYCGYTGRCRGMTGGMRRWSTACATSTMYTRCSSMGYRTTAYAKY 24900
Bhutan-09005         YRYMYMMYMKGCRGAMCTKYARGYASYCRRAGRWGWTTMYGKCYYGYCCCRCGKGTCRGT 24900
Indonesia-88035      YRYMYMMTMKGCRGAMCTKYARGCASYCRGMGRWGWTTMYGKCYYGCCCCRCGTGTCRGT 24900
Indonesia-88045      YRYMYMMTMKGCRGAMCTKYARGCASYCRGMGRWGWTTMYGKCYYGCCCCRCGTGTCRGT 24900
Indonesia-88065      YRYMYMMYMKGCRGAMCTKYARGYASYCRRAGRWGWTTMYGKCYYGYCCCRCGKGTCRGT 24900
                           *   .  . *   *:    .      **. :   .. *   ..    . :

Bhutan-09015         GGGCWRRRCTCMYRYGMSAGCRGTMRGWWRAATYAWWGAYCYKYYYRYRTYAYRMGRTWC 24960
Bhutan-09024         GGGCWRRRCTCMYRYGMSAGCRGTMRGWWRAATYAWWGAYCYKYYYRYRTYAYRMGRTWC 24960
```

```
Bhutan-09027         GGGCWRRRCTCMYRYGMSAGCRGTMRGWWRAATYAWWGAYCYKYYYRYRTYATAMGRTWC 24960
Bhutan-09030         GGGCWRRRCTCMYRYGMSAGCRGTMRGWWRAATYAWWGAYCYKYYYRYRTYAYRMGRTWC 24960
Bhutan-09005         GGRTWRRRCATMCGTRACRRYGRKARAAAGRRYYTWAARCYYGCYCAYGYCRCGAKRYAY 24960
Indonesia-88035      RRRTWRARMATMCGYGMCRRYGRKARAAAGRRYYTWAARCYYGCYCAYGYCRCGAKRYAY 24960
Indonesia-88045      RRRTWRARMATMCGYGMCRRYGRKARAAAGRRYYTWAARCYYGCYCAYGYCRCGAKRYAY 24960
Indonesia-88065      GGRTWRRRCATMCGTRACRRYGRKARAAAGRRYYTWAARCYYGCYCAYGYCRCGAKRYAY 24960
                         ** * : *     .     . *.      *:* .   *  *  *        *

Bhutan-09015         WARGATARRRGTMAYGGRSSSYGASSARRMARKRWWSMSTYSTYKMWAARTMTYKKRRTK 25020
Bhutan-09024         WARGATARRRGTMAYGGRSSSYGASSARRMARKRWWSMSTYSTYKMWAARTMTYKKRRTK 25020
Bhutan-09027         WARGATARRRGTMAYGGRSSSYGASSARRMARKRWWSMSTYSTYKMWAARTMTYKKRRTK 25020
Bhutan-09030         WARGATARRRGTMAYGGRSSSYGASSARRMARKRWWSMSTYSTYKMWAARTMTYKKRRTK 25020
Bhutan-09005         WWRAWKRAGGRYACYRTGCGCTATGCRMAMRGKRTTCAGTYGYYGCACRAYCYCGKARGT 25020
Indonesia-88035      WWRAWKRAGGRYACYRTGCGCTATGCRMAMRGKRTTCAGKYGYYGCACRAYCTCGKARGT 25020
Indonesia-88045      WWRAWKRAGGRYACYRTGCGCTATGCRMAMRGKRTTCAGKYGYYGCACRAYCTCGKARGT 25020
Indonesia-88065      WWRAWKRAGGRYACYRTGCGCTATGCRMAMRGKRTTCAGTYGYYGCACRAYCYCGKARGT 25020
                     * *. .       .*   ... .:..   *  **  . ..*. *   .       * * .

Bhutan-09015         YTWYTSWRRSRRTTRWYRAGGCAYSKRCARRCYTAWSGYWKYWGMYTTRRYRCYGTYRMT 25080
Bhutan-09024         YTWYTSWRRSRRTTRWYRAGGCAYSKRCARRCYTAWSGYWKYWGMYTTRRYRCYGTYRMT 25080
Bhutan-09027         YTWYTSWRRSRRTTRWYRAGGCAYSKRCARRCYTAWSGYWKYWGMYTTRRYRCYGTYRMT 25080
Bhutan-09030         YTWYTSWRRSRRTTRWYRAGGCAYSKRCARRCYTAWSGYWKYWGMYTTRRYRCYGTYRMT 25080
Bhutan-09005         TWACYCTAAGRRCTAATGTRKGMCSKGYGRASYWRWCSCWKTWTCTYWARYGYCSWCGAK 25080
Indonesia-88035      TWACYCTAAGRRCTAATGTRKGMCSKGYGRASYWRWCSCWKTWTCTYWARYGYCSWCGAK 25080
Indonesia-88045      TWACYCTAAGRRCWAATGTRKGMCSKGYGRASYWRWCSCWKTWTCTYWARYGCCSWCGAK 25080
Indonesia-88065      TWACYCTAAGRRCTAATGTRKGMCSKGYGRASYWRWCSCWKTWTCTYWARYGYCSWCGAK 25080
                          .   .**      :     **  .* .*  *.. ** *      **   .    .

Bhutan-09015         YRCGTRGGGYRYRACTTAYCGWCMMRAYWTYWAAYCCMYMMGGWWYTTTSAYWYTCRAKY 25140
Bhutan-09024         YRCGTRGGGYRYRACTTAYCGWCMMRAYWTYWAAYCCMYMMGGATTTTTSAYWYTCRAKY 25140
Bhutan-09027         YRCGTRGGGYRYRACTTAYCGWCMMRAYWTYWAAYCCMYMMGGWWYTTTSAYWYTCRAKY 25140
Bhutan-09030         YRCGTRGGGYRYRACTTAYCGWCMMRAYWTYWAAYCCMYMMGGWWYTTTSAYWYTCRAKY 25140
Bhutan-09005         TGMSYRGCAYRCGACTTGYSAWYMCGRTWYYARRTAMAYMMSRTACCGKCRCACKYGRKC 25140
Indonesia-88035      TGMSYRRSAYRCGACTTGYSAWYMCGRTACYARRTAMAYMMSRTACCGTCRCACKYGRKC 25140
Indonesia-88045      TGMSYRRSAYRCGRYYKGYSAWYMCGRTACYARRTAMAYMMSRTACCGTCRCACKYGRKC 25140
Indonesia-88065      TGMSYRGCAYRCGACTTGYSAWYMCGRTWYYARRTAMAYMMSRTACCGKCRCACKYGRKC 25140
                        . *  .**     ..*..* *      *    .  ***.      ..    .   *

Bhutan-09015         AYCYYYCRCRYYKWWYWRARSMCSCTKWYYTSYCYWRRMSSMGGKATGYRGRSKRTGYRY 25200
Bhutan-09024         AYCYYYCRCRYYKWWYWRARSMCSCTKWYYTSYCYWRRMSSMGGKATGYRGRSKRTGYRY 25200
Bhutan-09027         AYCYYYCRCRYYKWWYWRARSMCSCTKWYYTSYCYWRRMSSMGGKATGYRGRSKRTGYRY 25200
Bhutan-09030         AYCYYYCRCRYYKWWYWRARSMCSCTKWYYTSYCYWRRMSSMGGKATGYRGRSKRTGYRY 25200
Bhutan-09005         RWYYYYTGRGRTTKAWTTAWRGCYCYWGTYTGGTTYTRRACSMRRGGGATAGAGTATRYGT 25200
Indonesia-88035      RWYYYYTGRGRTTKAWTTAWRGCYCYWGTYTGGTTYTRRACSMRRGGGATASAGTAYRYGT 25200
Indonesia-88045      RWYYYYTGRGRTTKAWTTAWRGCYCYWGTTTGGTTYTRRACSMRRGGGATASAGTAYRYGT 25200
Indonesia-88065      RWYYYYTGRGRTTKAWTTAWRGCYCYWGTYTGGTTYTRRACSMRRGGGATAGAGTATRYGT 25200
                      : **  * *  * *    *.  .       .  * ** .**   . .  . ..   *

Bhutan-09015         KYRGYCYCWSTGSAATMYYYKYYKRYRYAASMKCRWWKCTWGCYRRRGCRGYWSAWGCKA 25260
Bhutan-09024         KYRGYCCCWSTGSAATMYYYKYYKRYRYAASMKCRWWKCTWGCYRRRGCRGYWSAWGCKA 25260
Bhutan-09027         KYRGYCYCWSTGSAATMYYYKYYKRYRYAACCKCRWWKCTWGCYRRRGCRGYWSAWGCKA 25260
Bhutan-09030         KYRGYCYCWSTGSAATMYYYKYYKRYRYAASMKCRWWKCTWGCYRRRGCRGYWSAWGCKA 25260
Bhutan-09005         TCARCTTSTGYCSCGGMTCCTCTTGCRCRMCCTYRWWGSAWRMYRARRMRAYWCWTKTTM 25260
Indonesia-88035      TCARCTTSTGYCSCGGMTCCTCTTGCRCRMCCTYRWWGSAWRMYRARRMRAYWCWTKTTM 25260
```

```
Indonesia-88045      TCARCTTSTGYCSCGGMTCCTCTTGCRCRMCCTYRWWGSAWRMYRARRMRAYWCWTKTTM 25260
Indonesia-88065      TCARCTTSTGYCSCGGMTCCTCTTGCRCRMCCTYRWWGSAWRMYRARRMRAYWCWTKTTM 25260
                     .      . .  *.. *   .  .  *   . . *** .:*  ** *  *.**.    .

Bhutan-09015         TTMRMGYGCTMYYSYTYMKGASWSYRTYSAYARTMKRYAAMTWTGTKTWWYWCYGCATGW 25320
Bhutan-09024         TTMRMGYGCTMYYSYTYMKGASWSYRTYSAYARTMKRYAAMTWTGTKTWWYWCYGCATGW 25320
Bhutan-09027         TTMRMGYGCTMYYSYTYMKGASWSYRTYSAYARTMKRYAAMTWTGTKTWWYWCYGCATGW 25320
Bhutan-09030         TTMRMGYGCTMYYSYTYMKGASWSYRTYSAYARTMKRYAAMTWTGTKTWWYWCYGCATGW 25320
Bhutan-09005         WWMGCRTKYAMYYCYCCCKRWGTGYRWYCWTTRYMGRYWRAWAWSCKTWWYWTCSSMGKT 25320
Indonesia-88035      WWMGCRTKYAMCCCYCCCKRWGTGYRWYCWTTRYMGRYWRAWAWSCTWAWYWTCSSMGKT 25320
Indonesia-88045      WWMGCRTKYAMCCCYCCCKRWGTGYRWYCWTTRYMGRYWRAWAWSCTWAWYWTCSSAGKT 25320
Indonesia-88065      WWMGCRTKYAMCCCYCCCKRWGTGYRWYCWTTRYMGRYWRAWAWSCKTWWYWTCSSAGKT 25320
                       *      :*  .*   *   . .** *.  :* * **       . .  ***  ..

Bhutan-09015         AAWYRTRCYCSAKTRTATTCWWYYRYYGGRKWRYWGRYAGRCTATRRGGRCSYGGARYTG 25380
Bhutan-09024         AAWYRTRCYCSAKTRTATTCWWYYRYYGGRKWRYWGRYAGRCTATRRGGRCSYGGARYTG 25380
Bhutan-09027         AAWYRTRCYCSAKTRTATTCWWYYRYYGGRKWRYWGRYAGRCTATRRGGRCSYGGARYTG 25380
Bhutan-09030         AAWYRTRCYCSAKTRTATTCWWYYRYYGGRKWRYWGRYAGRCTATRRGGRCSYGGARYTG 25380
Bhutan-09005         GCWMRKRGTMSRKYAWGCGYTACCATTRRAWWATTRRCRRRYKGAGASCRSSCARRGTYR 25380
Indonesia-88035      GCWMRKRGTMSRKYAWGCGYTACCATTRRAWWATTRRCRRRYKGAGASCRSSCARRGTYR 25380
Indonesia-88045      GCWMRKRGTMSRKYAWGCGYTACCATTRRAWWATTRRCRRRYKGAGASCRSSCARRGTYR 25380
Indonesia-88065      GCWMRKRGTMSRKYAWGCGYTACCATTRRAWWATTRRCRRRYKGAGASCRSSCARRGTYR 25380
                     ..* *.*   * *    .              *    *    * ..:  . *.* .

Bhutan-09015         RMGAMWMYGAYRWGRMRCYACRSRYWCYMYCWCRWCAKKTYGTKYYGWGRYGRGSTCKCR 25440
Bhutan-09024         RMGAMWMYGAYRWGRMRCYACRSRYWCYMYCWCRWCAKKTYGTKYYGWGRYGRGSTCKCR 25440
Bhutan-09027         RMGAMWMYGAYRWGRMRCYACRSRYWCYMYCWCRWCAKTTYGTKYYGWGRYGRGSTCKCR 25440
Bhutan-09030         RMGAMWMYGAYRWGRMRCYACRSRYWCYMYCWCRWCAKKTYGTKYYGWGRYGRGSTCKCR 25440
Bhutan-09005         RMKMMAACTRTRATGCGMTMTGCATWTTATTTARAARKTYTTYKCYRARAYARSSYGTYG 25440
Indonesia-88035      RMKMMAACTRTRATGCGMTMTGCATWTTATTTARAARKTYTTYKCYRARAYARSSYGTYG 25440
Indonesia-88045      RMKMMAACTRTRATGCGMTMTGCATWTTATTTARAARKTYTTYKCYRARATARSSYGTYG 25440
Indonesia-88065      RMKMMAACTRTRATGCGMTMTGCATWTTATTTARAARKTYTTYKCYRARAYARSSYGTYG 25440
                     **  *      *          .  *      .* . *.     * *      .*.*  .

Bhutan-09015         AYYCYYRAMRCKKTRAYYRGCRCMSTWYYYYTGRRRRACRCRYWCCYSGYCWRGYRYGTC 25500
Bhutan-09024         AYYCYYRAMRCKKTRAYYRGCRCMSTWYYYYTGRRRRACRCRYWCCYSGYCWRGYRYGTC 25500
Bhutan-09027         AYYCYYRAMRCKKTRAYYRGCRCMSTWYYYYTGRRRRACRCRYWCCYSGYCWRGYRYGTC 25500
Bhutan-09030         AYYCYYRAMRCKKTRAYYRGCRCMSTWYYYYTGRRRRACRCRYWCCYSGYCWRGYRYGTC 25500
Bhutan-09005         CCTYTTAGCGYTGYGRTTRRYAYCGKATYCTWRGARARYRYACKMYYSSYSWRRYGTSYA 25500
Indonesia-88035      CCTYTTAGCGYTGYGRTTRRYAYCGKATYCTWRGARARYRYACKMYYSSYSWRRYGTSYA 25500
Indonesia-88045      CCTYTTAGCGYTGYGRTTRRYAYCGKATYCTWRGARARYRYACKMYYSSTSWRGTGTSYA 25500
Indonesia-88065      CCTYTTAGCGYTGYGRTTRRYAYCGKATYCTWRGARARYRYACKMYYSSYSWRRYGTSYA 25500
                     .      .   .      *      ..  *       *   *       **. .**     . .

Bhutan-09015         YMWGWTCSYAMTYKATARSARRGGCAYTMTYAYCYRRKGRWYRKWTACMYTYAYGGWWGT 25560
Bhutan-09024         YMWGWTCSYAMTYKATARSARRGGCAYTMTYAYCYRRKGRWYRKWTACMYTYAYGGWWGT 25560
Bhutan-09027         YMWGWTCSYAMTYKATARSARRGGCAYTMTYAYCYRRKGRWYRKWTACMYTYAYGGWWGT 25560
Bhutan-09030         YMWGWTCSYAMTYKATARSARRGGCAYTMTYAYCYRRKGRWYRKWTACMYTYAYGGWWGT 25560
Bhutan-09005         TAARWYTSTRACYTWTARSGGGRCTMYKMCYMCYCGGGRGTTAGTYRAACYTMCARWART 25560
Indonesia-88035      TAARWYTSTRACYTWYWRSGGGRCTMYKMCYMCYCGGGRGTTAGTYRAACYTMCARWARY 25560
Indonesia-88045      TAARWYTSTRACYTWYWRSGGGRCTMYKMCYMCYCGGGRGTTAGTYRAACYTMCARWARY 25560
Indonesia-88065      TAARWYTSTRACYTATARSGGGRCTMYKMCYMCYCGGGRGTTAGTYRAACYTMCARWART 25560
                         *  *    *.   **.      *.* *                  .      . *
```

```
Bhutan-09015         RKRKYCTTRRWTYKRTAYRYRGMWTYGTRYRYATGKAKTTKYTAKTSRTSCCGMRRRYRM 25620
Bhutan-09024         RKRKYCTTRRWTYKRTAYRYRGMWTYGTRYRYATGKAKTTKYTAKTSRTSCCGMRRRYRM 25620
Bhutan-09027         RKRKYCTTRRWTYKRTAYRYRGMWTYGTRYRYATGKAKTTKYTAKTSRTSCCGMRRRYRM 25620
Bhutan-09030         RKRKYCTTRRWTYKRTAYRYRGMWTYGTRYRYATGKAKTTKYTAKTSRTSCCGMRRRYRM 25620
Bhutan-09005         RTAGCYCGARWWYGGAGYRYRCAWGCRTRCRYWKAGRKWCKTKWGYSRYCYTGCGGACRC 25620
Indonesia-88035      RTAGCYCGRRWWYGGAGYKYACAWGCRYGCRYWKAGRKWCKTKWGYSRYCYTRCGGACRC 25620
Indonesia-88045      RTAGCYCGRRWWYGGAGYKYACAWGCRYGCRYWKAGRKTCKTKWGYSRYCYTRCGGACRC 25620
Indonesia-88065      RTAGCYCGARWWYGGAGYRYRCAWGCRTRCRYWKAGRKWCKTKWGYSRYCYTGCGGACRC 25620
                     *.       ** *  :.*:*   *      ** ..  *  * .   ** .        *

Bhutan-09015         CGGYTCAAWTGRCAKMRSAYYAKMCACRRAYGYSRYYTTGTYTTGRRWRKYRTTGCGYRM 25680
Bhutan-09024         CGGYTCAAWTGRCAKMRSAYYAKMCACRRAYGYSRYYTTGTYTTGRRWRKYRTTGCGYRM 25680
Bhutan-09027         CGGYTCAAWTGRCAKMRSAYYAKMCACRRAYGYSRYYTTGTYTTGRRWRKYGTTGCGYRM 25680
Bhutan-09030         CGGYTCAAWTGRCAKMRSAYYAKMCACRRAYGYSRYYTTGTYTTGRRWRKYRTTGCGYRM 25680
Bhutan-09005         YSGYCTMRAYGAYWGCRSCYYRGATRYGRGTSTSRTCYYAKTYKCGRWGTTRYYRYRTGM 25680
Indonesia-88035      YSKYCTAAAYKAYWGCRSCYYRGATRYGRGTSTSRTCYYAKTYKCGRWGTTRYYGYATGM 25680
Indonesia-88045      YSKYCTAAAYKAYWGCRSCYYRGATRYGRGTSTSRTCYYAKTYKCGRWGTTRYYGYATGM 25680
Indonesia-88065      YSGYCTMRAYGAYWGCRSCYYRGATRYGRGTSTSRTCYYAKTYKCGRWGTTRYYRYRTGM 25680
                      . *            **.**       *. . **    ..  .  ** .         *

Bhutan-09015         YKGKAYRWWGRYAWYARGGKMRATAGKGGACARMGTRCWMRYYGRGWRCATACTYYRYAA 25740
Bhutan-09024         YKGKAYRWWGRYAWYARGGKMRATAGKGGACARMGTRCWMRYYGRGWRCATACTYYRYAA 25740
Bhutan-09027         YKGKAYRWWGRYAWYARGGKMRATAGKGGACARMGTRCWMRYYGRGWRCATACTYYRYAA 25740
Bhutan-09030         YKGKAYRWWGRYAWYARGGKMRATAGKGGACARMGTRCWMRYYGRGWRCATACTYYRYAA 25740
Bhutan-09005         YKKGAYGATAGTGAYWGRATCAMCRRGGRRTRAARYRGTARYYCARTAYRWRAKYCGCRG 25740
Indonesia-88035      YKKGRCGATGGTGAYWGRATCAMCRRGSRRTRAARYRGTARYYCARTAYRWRAKYCGCRG 25740
Indonesia-88045      YKKGRCGATGGTGAYWGRATCAMCRRGSRRTRAARYRGTARYYCARTAYRWRAKYCGCRG 25740
Indonesia-88065      YKKGAYGATAGTGAYWGRATCAMCRRGGRRTRAARYRGTARYYCARTAYRWRAKYCGCRG 25740
                     **       .  . *   ..       .        *   ***         ..*    .

Bhutan-09015         TAATCKRKGAACTKAKTASARATGMGAGTARAMRAYMRCYYCARWRYTRYCCGRYTYRWM 25800
Bhutan-09024         TAATCKRGGAACTKAKTASARATGMGAGTARAMRAYMRCYYCAGTRYTRYCCGRYTYRWM 25800
Bhutan-09027         TAATCKRKGAACTKAKTASARATGMGAGTARAMRAYMRCYYCAGTRYTRYCCGRYTYRWM 25800
Bhutan-09030         TAATCKRKGAACTKAKTASARATGMGAGTARAMRAYMRCYYCAGTRYTRYCCGRYTYRWM 25800
Bhutan-09005         CGRCYGAKTGGGYTWKYRCRGRWKCRMCWWRRCGGYAAMCCYRAAGCGACAYAGTYYRWC 25800
Indonesia-88035      CGACYGAKTGGGYTWKYRCRGRWGCRMCWWRGCGGYAAMCCYRAAGCGACAYAGTYYRWC 25800
Indonesia-88045      CGACYGAKTGGGYTWKYRCRGRWKCGMCWWRGCGGYAAMCCYRAAGCGACAYAGTYYRWC 25800
Indonesia-88065      CGACYGAKTGGGYTWKYRCRGRWKCRMCWWRGCGGYAAMCCYRAAGCGACAYAGTYYRWC 25800
                      .       ..  . *  .           *   .*              . .   ***

Bhutan-09015         CGGGGAYATGKWCCGRYRRCGYYARAMASTKTMCYAYAGGAYARTMAYGYYYWTWMYYKG 25860
Bhutan-09024         CGGGGAYATGKWCCGRYRRCGYYARAMASTKTMCYAYAGGAYARTMAYGYYYWTWMYYKG 25860
Bhutan-09027         CGGGGAYATGKWCCGRYRRCGYYARAMASTKTMCYAYAGGAYARTMAYGYYYWTWMYYKR 25860
Bhutan-09030         CGGGGAYATGKWCCGRYRRCGYYARAMASTKTMCYAYAGGAYARTMAYGYYYWTWMYYKR 25860
Bhutan-09005         YKRRRWCRWKGTYAKACGGSSYYRRRCTSYKYWYCMTRAKMCRGCACYRYYCWCAACTGG 25860
Indonesia-88035      YKRRRWCRWKGTYAKACGGSSYYRRRCTSYKYWYCMTRAKMCRGCACYRYYCWCAACTGG 25860
Indonesia-88045      YKRRRWCRTKGTYAKACGGSSYYRGACTSYKYTTCMTRAKMCRGCACYRYYCWCAACTGG 25860
Indonesia-88065      YKRRRWCRWKGTYAKACGGSSYYRRRCTSYKYWYCMTRAKMCRGCACYRYYCWCAACTGG 25860
                                  .     ..**    :* *       .       .* ** *

Bhutan-09015         TYTYTRMWYGTCKTACYCMRYKWYYKKCYYRGKRAYARSYRRSARYRYSMKYRWCWCRCW 25920
Bhutan-09024         TYTYTRMWYGTCKTACYCMRYKWYYKKCYYRGKRAYARSYRRSARYRYSMKTRWCWCRCW 25920
Bhutan-09027         TYTYTRMWYGTCKTACYCMRYKWYYKKCYYRGKRAYARSYRRSARYRYSMKYRWCWCRCW 25920
Bhutan-09030         TYTYTRMWYGTCKTACYCMRYKWYYKKCYYRGKRAYARSYRRSARYRYSMKYRWCWCRCW 25920
```

```
Bhutan-09005         WYWYWGCTCACTTCRYTYCRTGWYTTGTCTGKKARCGGGYAGGRGMGYSMKYAWMWYRYW 25920
Indonesia-88035      WCWYWGCTCACTTCACTYCRTGWYTTGTCTGKKARCGGGYAGGRGMGYSMKYAACTYRYW 25920
Indonesia-88045      WCWYWGCTCACTTCRYTYCRTGWYTTGTCTGKKARCGGGYAGGRGMGTSMKYAWMWYRYW 25920
Indonesia-88065      WCWYWGCTCACTTCRYTYCRTGWYTTGTCTGKKARCGGGYAGGRGMGYSMKYAWMWYRYW 25920
                        *     .  .      *  ** .      *   . .*  .     ***      * *

Bhutan-09015         GGGRYKRCRMRTYCYRTACGATARYTKYTKRYRWCCWGWARSYYYYYTCYMWKCMSCSYR 25980
Bhutan-09024         GGGRYKRCRMRTYCYRTACGATARYTKYTKRYRWCCWGWARSYYYYYTCYMWKCMSCSYR 25980
Bhutan-09027         GGGRYKRCRMRTYCYRTACGATARYTKYTKRYRWCCWGWARSYYYYYTCYMWKCMSCSYR 25980
Bhutan-09030         GGGRYKRCRMRTYCYRTACGATARYTKYTKRYRWCCWGWARSYYYYYTCYMWKCMSCSYR 25980
Bhutan-09005         KRRGYTAAAMRKTYYAWRYRTYRRYTGYYTACRWYYWCARGSYYTYYWGCMWKYMCTSCA 25980
Indonesia-88035      KRRGYTAAAMRKTYYAWRYRTYRRYYGYYTACRWYYWCAGGSYYTYYWGCMWKYMCTSCA 25980
Indonesia-88045      KRRGYTAAAMRKTYYAWRYRTYRRYYGYYTACRWYYWCARGSYYTYYWGCMWKYMCTSCA 25980
Indonesia-88065      KRRGYTAAAMRKTYYATRYRTYRRYYGYYTACRWYYWCARGSYYTYYWGCMWKYMCTSCA 25980
                         *. . **.  *     :  **  * .  **  *    *** **   *** *. *

Bhutan-09015         RYYCWYGCMCATWATYTGRRRGCGYCTAKYGYYSTTMCARTSTYWYAYYAAKYCAGRYYR 26040
Bhutan-09024         RYYCWYGCMCATWWTYTGRRRGCGYCTAKYGYYSTTMCARTSTYWCAYYAAKYCAGRYYR 26040
Bhutan-09027         RYYCWYGCMCATWAWYTGRRRGCGYYTAKYGYYSTTMCARTSTYWYAYYAAKYCAGRYYR 26040
Bhutan-09030         RYYCWYGCMCATWATYTGRRRGCGYCTAKYGYYSTTMCARTSTYWYAYYAAKYCAGRYYR 26040
Bhutan-09005         AYTTACATAYGAAATYYRGRRRAAYCCRTTRCTGCYMYMAWCYCTCRYCRRKCCGRRCYG 26040
Indonesia-88035      AYTTACATAYGAAATYYRGRRRAAYCCRTTRCTGCYMYMRTCYCTCRYCRRKCYGRRCYG 26040
Indonesia-88045      AYTTACATAYGAAATYYRGRRRAAYCCRTTRCTGCYMYMRTCYCTCRYCRRKCCGRRCYG 26040
Indonesia-88065      AYTTACATAYGAAATYYRGRRRAAYCCRTTRCTGCYMYMAWCYCTCRCCRRKCCGRRCYG 26040
                      *    .   .:   *   ** ..*   .    .  *    .         *  . * *

Bhutan-09015         AGAGGAGTTGACKSCYGKTWTYSKYGCYCGARKRAAYRCYTAYRRKAWYRSRTKWTSAYT 26100
Bhutan-09024         AGAGGAGTTGACKSCYGKTWTYSKYGCYCGARKRAAYRCYTAYRRKAWYRSRTKWTSAYT 26100
Bhutan-09027         AGAGGAGTTGACKSCYGKTWTYSKYGCYCGARKRAAYRCYTAYRATAWYRSRTKWTSAYT 26100
Bhutan-09030         AGAGGAGTTGACKSCYGKTWTYSKYGCYCGARKRAAYRCYTAYRATAWYRSRTKWTSAYT 26100
Bhutan-09005         ARCACGACYAWAKSYYRGWWKYGGYKYCTGWRKRRGTGMCYRTGGTGWYACRWGWKYWYY 26100
Indonesia-88035      ARCACGACYAWAKSYYRGWWKYGGYKYCTGWRKRRGTGMCYRTGGKGWYACRWGWKYWYY 26100
Indonesia-88045      ARCACGACCAWAKSYYRGWWKYGGYKYCTRWRKRRGTGMCYRTGGKGWYACRWGWKYWYY 26100
Indonesia-88065      MRCACGACCAWAKSYYRGWWKYGGYKYCTGWRKRRGTGMCYRTGGKGWYACRWGWKYWYY 26100
                       .. ..  . .** *   *.*. *      *** .         ..** .*  *.  *

Bhutan-09015         RKGRCKSYYRYKKWSGRAASRRWRRTGGCARRAMWRKAMYRMCSRRGMTWYRSAKCCCTA 26160
Bhutan-09024         RKGRCKSYYRYKKWSGRAASRRWRRTGGCARRAMWRTAMYRMCSRRGMTWYRSAKCCCTA 26160
Bhutan-09027         RKGRCKSYYRYKKWSGRAASRRWRRTGGCARRAMWRKAMYRMCSRRGMTWYRSAKCCCTA 26160
Bhutan-09030         RKGRCKSYYRYKKWSGRAASRRWRRTGGCARRAMWRKAMYRMCSRRGMTWYRSAKCCCTA 26160
Bhutan-09005         ATRGYKCYTRYTKASCRRTGGAWAAYGACRRRRCARKGMCAMMSGGTAYWYACGTTCCYW 26160
Indonesia-88035      ATRGYKCYTRYTKASCRRTGGAWAATGAMRRRRCARKGMCAMMSGGTATWYACGTYSSYW 26160
Indonesia-88045      ATRGYKCYTRYTKASCRRTGGAWAATGACRRRRCARKGACAMMSGGTATWYACGTYSSYW 26160
Indonesia-88065      ATRGYKCYTRYTKASCRRTGGAWAAYKACRRRRCARKGMCAMMSGGTAYWYACGTTCCYW 26160
                      .   *.* **.* * * :.  *    .  **   *..   * *     ** ... ..

Bhutan-09015         KYCRATTCGRRYASGAYYMGYAKYRYMRGACRYCRRYCGRYYYSKYGYKKSRRTGGCGTR 26220
Bhutan-09024         KYCRATTCGRRYASGAYYMGYAKYRYMRGACRYCRRYCGRYYYSKYGYKKSRRTGGCGTR 26220
Bhutan-09027         KYCRATTCGRRYASGAYYMGYAKYRYMRGACRYCRRYCGRYYYSKYGYKKSRRTGGCGTR 26220
Bhutan-09030         KYCRATTCGRRYASGAYYMGYAKYRYMRGACRYCRRYCGRYYYSKYGYKKSRRTGGCGTR 26220
Bhutan-09005         KTARWWWYGGGTRCSMYYMRTTKCAYARKTYRCSARTARGTCCCTCRCGKGARCASYRWG 26220
Indonesia-88035      KTARATTYRGGTRCSMYYMGTTKCAYARKTYRCSARTARGTCCCTCRCGKGARCASYRWG 26220
Indonesia-88045      KTARATTYRGGTRCGMYYMRTTKCAYARKTYRCSARTARGTCCCTCRCGKGARCASYRTG 26220
Indonesia-88065      KTARWWWYGGGTRCSMYYMRTTKCAYARKTYRCSARTARGTCCCTCRCGKGARCASYRWG 26220
```

```
                     * .*         .. ***  :*  * * : * . * .     ..    *. * ..

Bhutan-09015         TTCYGCSMSGMKWRMKWCGWRRTYGGGCGGACWGYRAYKKMYCRWATMWTCWRYRYRWRA 26280
Bhutan-09024         TTCYGCSMSGMKWRMKWCGWRRTYGGGCGGACWGYRAYKKMYCRWATMWTCWRYRYRWRA 26280
Bhutan-09027         TTCYGCSMSGMKWRMKWCGWRRTYGGGCGGACWGTRAYKKMYCRWATMWTCWRYRYRWRA 26280
Bhutan-09030         TTCYGCSMSGMKWRMKWCGWRRTYGGGCGGACWGYRAYKKMYCRWATMWTCWRYRYRWRA 26280
Bhutan-09005         WWTTRTSMSRAGAAAGAARARRCYARATAKRYWRTARYKTMCMRTWWMWYYWRTGTGTGR 26280
Indonesia-88035      WWTTRTSMSRAGAAAGAARARRCYARATAKRYWRTARYKTMCMRTWWMWYYWRTGTGTGR 26280
Indonesia-88045      WWTTRTSMSRAGAAAGAARARRCYARATAKRYWRTARYKTMCMRTWWMWYYWRTGTGTGR 26280
Indonesia-88065      WWTTRTSMSRAGAAAGAARARACYARATAKRYWRTARYKTMCMRTWWMWYYWRTGTGTGR 26280
                           ***        .  *  *. . .   *    **.*  *   **  **

Bhutan-09015         MWYMYTWSYTWYYMYRMCMMGRMWRCYYYKRTRMWRKRAWRWWTCCGGYWYAYWTTCRMY 26340
Bhutan-09024         MWYMYTWSYTWYYMYRMCMMGRMWRCYYYKRTRMWRKRAWRWWTCCGGYWYAYWTTCRMY 26340
Bhutan-09027         MWYMYTWSYTWYYMYRMCMMGRMWRCYYYKRTRMWRKRAWRWWTCCGGYWYAYWTTCRMY 26340
Bhutan-09030         MWYMYTWSYTWYYMYRMCMMGRMWRCYYYKRTRMWRKRAWRWWTCCGGYWYAYWTTCRMY 26340
Bhutan-09005         MWTCCTASTYTTYCTGAYCMRGCAGYTCMTGWGCARTGCARTAYYYCTYWYMTAKCTKMY 26340
Indonesia-88035      MWTCCTASTYTTYCTGAYCMRGCAGYTCMTGWGCARTGCARTAYYYCTYWYMTAKCTKMY 26340
Indonesia-88045      MWTCCYASTYTTYCTGAYCMRGCAGYTCMTGWGCARTGCARTAYYYCTYWYMTAKCTKMY 26340
Indonesia-88065      MWTCCTASTYTTYCTGAYCMRGCAGYTCMTGWGCARTGCARTAYYYCTYWYMTAKCTKMY 26340
                     **     *    *      *         .     *. . *       ***   .  :**

Bhutan-09015         GSGYWRSRYCYYTRAYASAAKYWSSMAMRRWTRSWYATTSACMGGCRRSWRARWWCRRYK 26400
Bhutan-09024         GSGTWRSRYCYYTRAYASAAKYWSSMAMRRWTRSWYATTSACMGGCRRSWRARWWCRRYK 26400
Bhutan-09027         GSGYWRSRYCYYTRAYASAAKYWSSMAMRRWTRSWYATTSACMGGCRRSWRARWWCRRYK 26400
Bhutan-09030         GSGYWRSRYCYYTRAYASAAKYWSSMAMRRWTRSWYATTSACMGGCRRSWRARWWCRRYK 26400
Bhutan-09005         AGRYTRGATYCTYATCGSRWGTACGMRMRGTWGCTTGYCCRYARRYGRSTGRAWTTRATK 26400
Indonesia-88035      AGRYTRGATYCTYATCGSRWGTACGMRMRGTWGCTTGYCCRYARRYGRCTGRAWTTRATK 26400
Indonesia-88045      AGRYTRGATYCTYATCGSRWGTACGMRMRGTWGCTTGYCCRYARRYGRCTGRAATTRATK 26400
Indonesia-88065      AGRYTRGATYCTYATCGSRWGTACGMRMRGTWGCTTGYCCRYARRYGRSTGRAWTTRATK 26400
                     ..   *.       : .*     ..* **    .  .  .       *.       *  *

Bhutan-09015         RWYRRRCCTYYYRARYCTSYGGKWMWRAAWRKRRYGCAGRTMAKMMRWYTCRCYKGGYWY 26460
Bhutan-09024         RWYRRRCCTYYYRARYCTSYGGKWMWRAAWGGRRYGCAGRTMAKMMRWYTCRCYKGGYWY 26460
Bhutan-09027         RWYRRRCCTYYYRARYCTSYGGKWMWRAAWRKRRYGCAGRTMAKMMRWYTCRCYKGGYWY 26460
Bhutan-09030         RWYRRRCCTYYYRARYCTSYGGKWMWRAAWRKRRYGCAGRTMAKMMRWYTCRCYKGGYWY 26460
Bhutan-09005         AWTGGGYSWYTYRWGYYYCYKRGAMTAMMWRGRGYKYRKAWMGKCCGWTYYAYCTTATAT 26460
Indonesia-88035      AWTGGGYSWYTYRWGYYYCYKRGAMTAMMWGGRGYKYRKAWMGKCCGWTYYMYCTTATAT 26460
Indonesia-88045      AWTGGGYSWYTYRWGYYYCYKRGAMTAMMWGGRGYKYRKAWMGKCCGWTYTMYCTTATAT 26460
Indonesia-88065      AWTGGGYSWYTYRWGYYYCYKRGAMTAMMWRGRGYKYRKAWMGKCCGWTYYMYCTTATAT 26460
                      *     . * **  *  .*    *    *  * *      *.*   *      .  .

Bhutan-09015         CATRGMYMKGWYRYYRYYYRTMMKYRRSWWRSRAYGTGRRRWRGCYCRMYRRGKWRATCT 26520
Bhutan-09024         CATRGMYMKGWYRYYRYYYRTMMKYRRSWWRSRAYGTGRRRWRGCYCRMYRRGKWRATCT 26520
Bhutan-09027         CATAGMYMKGWYRYYRYYYRTMMKYRRSWWRSRAYGTGRRRWRGCYCRMYRRGKWRATCT 26520
Bhutan-09030         CATRGMYMKGWYRYYRYYYRTMMKYRRSWWRSRAYGTGRRRWRGCYCRMYRRGKWRATCT 26520
Bhutan-09005         YRCAAMYCGSATAYCATCYRCACGYGRSTTAGGGYCYKGGATGATCYACCARRGAAGYAC 26520
Indonesia-88035      CACAAMYCGSATAYCATCYRCACGYGRSTTAGGGYCYKGGATGATCYACCARRGAAGYAC 26520
Indonesia-88045      CACAAMYCGSATAYCATCYRCACGYGRSTTAGGGYCYKGGATGATCYACCARRGAAGYAC 26520
Indonesia-88065      CACAAMYCGSATAYCATCYRCACGYGRSTTAGGGYCYKGGATGATCYACCARRGAAGYAC 26520
                         .**  .   *    **    * **   . .*        .       *    . .

Bhutan-09015         YSAWMWYRATGWRTTMAYYGTTYCRARAKCYGYRARRWYTWRWYYRYKWASRRYRYYGAG 26580
Bhutan-09024         YSAWMWYRATGWRTTMAYYGTTYCRARAKCYGYRARRWYTWRWYYRYKWASRRYRYYGAG 26580
```

```
Bhutan-09027         YSAWMWYRATGWRTTMAYCGTTYCRARAKCYGYRARRWYTWRWYYRYKWASRRYRYYGAG 26580
Bhutan-09030         YSAWMWYRATGWRTTMAYCGTTYCRARAKCYGYRARRWYTWRWYYRYKWASRRYRYYGAG 26580
Bhutan-09005         CGGTMATGMCKTGCKCRTYRKYYYRRRWGSTTYGGGRWCWWGATCGYKARSRGCRYTCWK 26580
Indonesia-88035      CGGTMATGMCKTGCKCRYYRKYYYRRRWGSTTYGGGRWCWWGATCGYKARSRGCRYTCWK 26580
Indonesia-88045      CGGTMATGMCKTGCKCRYYRKYYYRRRWGSTTYGGGRWCWWGATCGYKARSRGCRYTCWK 26580
Indonesia-88065      CGGTMATGMCKTGCKCRYYRKYYYRRRWGSTTYGGGRWCWWGATCGYKARSRGCRYTCWK 26580
                      .. *         .     . * * *  .  * . **  *     **  **  **

Bhutan-09015         GYGRKRRYRGGYCRSTYWYCGYGYSCKACWRTKCRRRRAYRRGYRKRTRGYWYATTCCAG 26640
Bhutan-09024         GYGRKRRCGGGYCRSTYWYCGYGYSCKACWRTKCRRRRAYRRGYRKRTRGYWYATTCCAG 26640
Bhutan-09027         GYGRKRRYRGGYCRSTYWYCGYGYSCKACWRTKCRRRRAYRRGYRKRTRGYWYATTCCAG 26640
Bhutan-09030         GYGRKRRYRGGYCRSTYWYCGYGYSCKACWRTKCRRRRAYRRGYRKRTRGYWYATTCCAG 26640
Bhutan-09005         KCTGKGACRRAYTACYYWYTGYATSYGMMTACTTGGAAMCRRSYRTRCARCWYTGWYTGG 26640
Indonesia-88035      KCTGKGACRRGYTACYYWCTRYATSYGMMTACTTGGAAMCRRSYRTRCARCWYTGWYTGS 26640
Indonesia-88045      KCTGKGACRRGYTACYYWCTRYATSYGMMTACTTGGAAMCRRSYRTRCARCTCTGWYTGS 26640
Indonesia-88065      KCTGKGACRRAYTACYYWCTGYATSYGMMTACTTGGAAMCRRSYRTRCARCTCTGWYTGG 26640
                         *     .*  . **   *. *       .       **.**.*      :    ..

Bhutan-09015         SSWWGKAYYRCAAWSRRYWRRWRKMKYRYYKTRWRWYKCRYWSYYWMSYAYSTYARAGRG 26700
Bhutan-09024         SSWWGKAYYRCAAWSRRYWRRWRTMKYRYYKTRWRWYKCRYWSYYWMSYAYSTYARAGRG 26700
Bhutan-09027         SSWWGKAYYRCAAWSRRYWRRWGTMKYRYYKTRWRWYKCRYWSYYWMSYAYSTYARAGRG 26700
Bhutan-09030         SSWWGKAYYRCAAASRRYWRRWRKMKYRYYKTRWRWYKCRYWSYYWMSYAYSTYARAGRG 26700
Bhutan-09005         SSTTSKRTYASRGAGAACWAGAGTATTACCTWATGMYGYGTTSCCAMGYGCGCYRAGRGR 26700
Indonesia-88035      SSTTSKRTYACAGAGAACWAGAGTATTACCTWATGMYGYGTTSCCAMGYGCGCYRAGRGR 26700
Indonesia-88045      SSTTSKRTYACAGAGAACWAGAGTATTACCTWATGMYGYGTTSCCAMGYGCGCYRAGRGR 26700
Indonesia-88065      SSTTSKRTYASRGAGAACWAGAGTATTACCTWATGMYGYGTTSCCAMGYGCGCYRAGRGR 26700
                     **  .*  * . . .   *    . .    .     *     *   *.*. . *  .

Bhutan-09015         TRYYCWYYTKYYRGATKGYYWRKAWTYCRYRTWTGWRWRKRCYWTMTTRRRRWATWMYRR 26760
Bhutan-09024         TRYYCWYYTKYYRGATKGYYWRKAWTYCRCRTWTGWRWRKRCYWTMTTRRRRWATWMYRR 26760
Bhutan-09027         TRYYCWYYTKYYRGATKGYYWRKAWTYCGCRTWTGWRWRKRCYWTMTTRRRRWATWMYRR 26760
Bhutan-09030         TRYYCWYYTKYYRGATKGYYWRKAWTYCRYATWTGWRWRKRCYWTMTTRRRRWATWMYRR 26760
Bhutan-09005         YACCYWCYGGYYRRGGKRYTARGWAACYATGYWYRTRAGKAATTAMKKAAAGTRTTCCGG 26760
Indonesia-88035      YACCYWCYGGYYRRGGKRYTARGWAACYATGYWYRTRAGKAATTAMKKAAAGTRYTCCGG 26760
Indonesia-88045      YACCYWCYGGYYRRGGKRYTARGWAACYATGTWYRTRAGKAATTAMKKAAAGTRYTCCGG 26760
Indonesia-88065      YACCYWCYGGYYRRGGKRYTARGWAACYATGYWYRTRAGKAATTAMKKAAAGTRTTCCGG 26760
                          * *  *** . * *  *   :      *   *  * .  :*..

Bhutan-09015         YKTAMASYRRYKCRGAWTARRMMGGYRRWYYYYGTTKKGWKMWYMWRMAASRWYASGATK 26820
Bhutan-09024         YKTAMASYRRYKCRGAWTARRMMGGYRRWYYYYGTTKKGWKMWYMWRMAASRWYASGATK 26820
Bhutan-09027         YKTAMASYRRYKCRGAWTARRMMGGYRRWYYYYGTTKKGWKMWYMWRMAASRWYASGATK 26820
Bhutan-09030         YKTAMASYRRYKCRGAWTARRMMGGYRRWYYYYGTTKKGWKMWYMWRMAASRWYASGATK 26820
Bhutan-09005         YKACMMCTGGTGSGRGAKRAAMCRAYGRWCCCYKKCTGRAGAACCARAWWCGTTWCKGCG 26820
Indonesia-88035      YKACAMCTGGTGSGRGAKRAAMCRAYGRWCCCYKKCTGRAGAACCARAAACGTTWCKGCG 26820
Indonesia-88045      YKACAMCTGGTGSGRGAKRAAMCRAYGRWCCCYKKCTGRAGAACCARAAWCGTTWCKGCG 26820
Indonesia-88065      YKACMMCTGGTGSGRGAKRAAMCRAYGRWCCCYKKCTGRAGAACCAAAWWCGTTWCKGCG 26820
                     **:.  .     .  . .   *  .* **   * . .             .    . .

Bhutan-09015         RYGRYSYRRSRYKYYKWTYRASKRMRTTSWTTCTTWRMCRACRMASCRTTYGTKMRYMRY 26880
Bhutan-09024         RYGRYSYRRSRYKYYKWTYRASKRMRTTSWTTCTTWRMCRACRMASCRTTYGTKMRYMRY 26880
Bhutan-09027         RYGRYSYRRSRYKYYKWTYRASKRMRTTSWTTCTTWRMCRACRMASCRTTYGTKMRYMRY 26880
Bhutan-09030         RYGRYSYRRSRYKYYKWTYRASKRMRTTSWTTCTTWRMCRACRMASCRTTYGTKMRYMRY 26880
Bhutan-09005         GTARTSCGGCRYGYTGTYCAMSTRMGGACTAWMYKWGAYRRSAAAGMGKKTATTMRYMAY 26880
Indonesia-88035      GTARTSCGGCRYGYTGTYCAMSTRMGGACTAWMYKWGAYRRSAARGMGKKTAYTMRYMAY 26880
```

```
Indonesia-88045      GTARTSCGGCRYGYTGTYCAMSTRMGGACTAWMYKWGAYRRSAARGMGKKTAYTMRYMAY 26880
Indonesia-88065      GTARTSCGGCRYGYTGTYCAMSTRMGGACTAWMYKWGAYRRSAARGMGKKTATTMRYMAY 26880
                       .* *   .** *       *.**  :. :   .*   * .   .  .. . .**** *

Bhutan-09015         YACCYGKRYARYRYKYMRKKRRRMWMRSRYAMTSRARWCYKGSRGRRRWWYAATWRTKRK 26940
Bhutan-09024         YACCYGTAYARYRYKYMRKKRRRMWMRSRYAMTSRARWCYKGSRGARRWWYAATWRTKRK 26940
Bhutan-09027         YACYYGKRYARYRYKYMRKKRRRATMGCRYAMTSRARWCYKGSRGARRWWYAATWRTKRK 26940
Bhutan-09030         YACCYGKRYARYRYKYMRKKRRRAWMRSRYAMTSRARWCYKGSRGRRRWWYAATWRTKRK 26940
Bhutan-09005         YRCCCRTAYRATAYKCCGKTRARATCRSGYMAKSRRGAYYGRSGRGGRTACRMATGAKRG 26940
Indonesia-88035      YRTCCRTAYRATAYKCCGKTRARATCRSGYMAKSRRGAYYGRSGRGGRTACRMATGAKRG 26940
Indonesia-88045      YRTCCRTAYRATAYKCCGKTRARATCRSGYMAKSRRGAYYGRSGRGGRTACRMATGAKRG 26940
Indonesia-88065      YRCCCRTAYRATAYKCCGKTRARATCRSGYMAKSRRGAYYGRSGRGGRTACRMATGAKRG 26940
                     *     . *    **   *.* *    . * .**    *  *    *     :  :**

Bhutan-09015         RMTWGARMTRTGMRGWTKYRWMGYTTCWCGWMYYCYYYACRGTTYYRRWYTYAWMKRKYT 27000
Bhutan-09024         RMTWGARMTRTGMRGWTKYRWMGYTTCWCGWMYYCYYYACRGTTYYRRWYTYAWMKRKYT 27000
Bhutan-09027         RMTWGARMTATGMRGWTKYRWMGYTTCWCGWMYYCYYYACRGTTYYRRWYTYAWMKRKYT 27000
Bhutan-09030         RMTWGARMTATGMRGWTKYRWMGYTTCWCGWMYYCYYYACRGTTYYRRWYTYAWMKRKYT 27000
Bhutan-09005         RCYARAAAYRYGCGAAYTTGAMKYYYCTYCWMYTYCCYRTGRKYCTAAATCTGACGATCC 27000
Indonesia-88035      RCYARRAATRYSCGAAYTTGAMKYYYSTYCWMYTYCCYRTGRKYCTAAATCTGACGATCC 27000
Indonesia-88045      RCYARRAATRYSCGAAYTTGAMKYYYSTYCWMYTYCCYRTGRKYCTAAATCTGACGATCC 27000
Indonesia-88065      RCYARAAAYRYGCGAAYTTGAMKYYYCTYCWMYTYCCYRTGRKYCTAAATCTGACGATCC 27000
                     *          .  .  .   * *  .   ***    *    .         .    .

Bhutan-09015         RYATAKYYRRYTMYTCMRYRYCSRGRARYYGCYRGYTKGGRAAARKCMGGYRSMWRCRWA 27060
Bhutan-09024         RYATAKYYRRYTMYTCMRYRYCSRGRARYYGCYRGYTKGGRAAARKCMGGYRSMWRCRWA 27060
Bhutan-09027         RYATAKYYRACTMYTCMRYRYCSRGRARYYGCYRGYTKGGRAAARKCMGGYASAWRCRWA 27060
Bhutan-09030         RYATAKYYRRYTAYTCMRYRYCSRGRARYYGCYRGYTKGGRAAARKCMGGYRSAWRCRWA 27060
Bhutan-09005         RYRWRGCCAACYMTYYAGTAYTSAAGGRYYRMYRTTCKRKGGGCRTYMRKTACATGGGTT 27060
Indonesia-88035      RYRWRGCCARYYMTYYAGTAYYCAAGGRYYRMYRTTCKRKGGGCRTYMRKTACMTGGGTT 27060
Indonesia-88045      RYRWRGCCARYYMTYYAGTACYCAAGGRYYRMYRTTCKRKGGGCRTYMRKTACMTGGGTT 27060
Indonesia-88065      RYRWRGCCARYYMTYYAGTAYTSAAGGRYYRMYRTTCKRKGGGCRTYMRTTACMTGGGTT 27060
                     **                    . . .***  **   *   ...*. *    .      :

Bhutan-09015         YRYGAAWYYRMTRRTYATTYYWKRRAACYSSCRGRYYGCCCWRRRGAYAYRGYGRSACKA 27120
Bhutan-09024         YRYGAAWYYRMTRRTYATTYYWKRRAACYSSCRGRYYKYYYWRRRGAYAYRGYGRSACKA 27120
Bhutan-09027         YRYGAAWYYRMTRRTYATTYYWKRRAACYSSCRGRYYKYYYWRRRGAYAYRGYGRSACKA 27120
Bhutan-09030         YRYGAAWYYRMTRRTYATTYYWKRRAACYSSCRGRYYKYYYWRRRGAYAYRGYGRSACKA 27120
Bhutan-09005         YGCGAGATYAMKGRYYRAYCCTGRGCRGTCGTRRGYYGCCCAGAGRWCRYRKCRRSTTTR 27120
Indonesia-88035      YGCTTGATYAMKGRYYRAYCCTGRGCRGTCGTRRGYYGCCCAGAGRWCRYRKCRRSTTTR 27120
Indonesia-88045      YGCTTGATYAMKGRYYRAYCCTGRGCRGTCGTRRGYYGCCCAGAGRWCRYRKCRRSTTTR 27120
Indonesia-88065      YGCGAGATYAMKGRYYRAYCCTGRGCRGTCGTRRGYYGCCCAGAGRWCRYRKCRRSTTTR 27120
                     *   :.  * *. * * :     * .   .. *  **            **   **: .

Bhutan-09015         GGARKRKASKMCYYAKGYYWGTAARARCGKTSYAYYAYRGWTAAYYMCCAYMYTCARTYY 27180
Bhutan-09024         GGARKRKASKMCYYAKGYYWGTAARARCGKTSYAYYAYRGWTAAYYMCCAYMYTCARTYY 27180
Bhutan-09027         GGARKRKASKMCYYAKGYYWGTAARARCGKTSYAYYAYRGWTAAYYCCCAYMYTCARTYY 27180
Bhutan-09030         GGARKRKASKMCYYAKGYYWGTAARARCGKTSYAYYAYRGWTAAYTMCCAYMYTCARTYY 27180
Bhutan-09005         RRRGTGTCGGCYCYRKTTTWGTRAGRRCKKYGYRTTRYGRAYMRCYAYAMYAYYTTAWYY 27180
Indonesia-88035      AAGGTGTCGGCYCYRKTTTWGKRAGRRYKKYGYRTTRYGRAYMRCYAYCMYAYYTTAWCY 27180
Indonesia-88045      AAGGTGTCGGCYCYRKTTTWRTRWGRRYKKYGYRTTRYGRAYMRCYAYCMYAYYTTAWCY 27180
Indonesia-88065      RRRGTGTCGGCYCYRKTTTWGTRAGRRCKKYGYRTTRYGRAYMRCYAYAMYAYYTTAWYY 27180
                         . ...    * *   * .    *  * .*    *          . * *  :   *
```

```
Bhutan-09015        RWYMSGGYYRTRCTTRMYRTARYRKRRRYYWCRYATSYCSRRRGKAYYRKTRGYWWYAYR 27240
Bhutan-09024        RWYMSGGYYRTRCTTRMYRTARYRKRRRYYWCRYATSYCSRRRGKAYYRKTRGYWWYAYR 27240
Bhutan-09027        RWYMSGGYYRTRCTTRMYRTARYRKRRRYYWCRYATSYCSRRRGKAYYRKTRGYWWYAYR 27240
Bhutan-09030        RWYMSGGYYRTRCTTRMYRTARYRKRRRYYWCRYATSYCSRRRGKAYYRKTRGYWWYAYR 27240
Bhutan-09005        ATTMCKRCYRCRMKYRACRYRAYRGRGGCYWYGCTCGCTGARRRTTYYGKWATYTATGYA 27240
Indonesia-88035     ATTMCKRCCRCRCTYRACRYRAYRGRGGCYWCGCTCGYYSARRGTTYYGKWATYTATGYA 27240
Indonesia-88045     ATTMCKRCCRCRCTYRACRYRAYRGRGGCYWCGCTCGYYSARRGTTYYGKWATYTATGYA 27240
Indonesia-88065     ATTMCKRCYRCRCTYRACRYRAYRGRGGCYWYGCTCGYTGARRRTTYYGKWATYTATGYA 27240
                       *.    * * . *  *   ** *   **   : .  . ** .:** *   *   .*

Bhutan-09015        SRRYYKRSKYRCRTACMRSYAARMRMYKYGTAYMYMSWSYYCGRRKAGYRWTYTGKWKGY 27300
Bhutan-09024        SRRYYKRSKYRCRTACMRSYAARMRMYKYGTAYMYMSWSYYCGRRKAGYRWTYTGKWKGY 27300
Bhutan-09027        SRRYYKRSKYRCRTACMRSYAARMRMYKYGTAYMYMSWSYYCGRRKAGYRWTYTGKWKGY 27300
Bhutan-09030        SRRYYKRSKYRCRTACMRSYAARMRMYKYGTAYMYMSWSYYCGRRKAGYRWTYTGKWKGY 27300
Bhutan-09005        CAGTCGGGKTAYRKRMARSYMGGMGCCKTSYRCMYMGTGTYAKGGGMRCRACYYRTTTCT 27300
Indonesia-88035     CAGTCGGGKYAYRTRMARSYMGGMGCCKTSYRCMYMGTGTYAKGGGMRCRACYYRTTTCT 27300
Indonesia-88045     CAGTCGGGKYAYRTRMARSYMGGMGCCKTSYRCMYMGTGTYAKGGGMRCRACYYRTTTCT 27300
Indonesia-88065     CAGTCGGGKTAYRKRMARSYMGGMGCCKTSYRCMYMGTGTYAKGGGMRCRACYYRTTTCT 27300
                    .      .*   *.   *** . *   * .   ***. . *.       *  *  . .

Bhutan-09015        GTGAMTAGGCTGWRSRYWKYAWYGTSAWWCSYGCCTYGKGWRARKAYYCYYYTGGCCGYG 27360
Bhutan-09024        GTGAMTAGGMYGWRSRYWKYAWYGTSAWWCSYGCCTYGKGWRARGAYYCYYYTSRCCGYG 27360
Bhutan-09027        GTGAMTAGGMYGWRSRYWKYAWYKYSAWWCSYGCCTYGKGWRARKAYYCYYYTGGCCGYG 27360
Bhutan-09030        GTGAMTAGGMYGWRSRYWKYAWYKYSAWWCSYGCCTYGKGWRARKAYYCYYYTGGCCGYG 27360
Bhutan-09005        ACRWAKGTAMYRTRCGYTGYTTTKCGMTATCCRYMKCRTAWRWGGWYYYTTYYCGMTGTT 27360
Indonesia-88035     AYRWAKGTAMYRTRCGYTGYTTTKCGMTATCCRYAGCRTAWRWGGWYYYTTYYGGMTATT 27360
Indonesia-88045     AYRWAKGTAMYRTRCGYTGYTTTKCGMTATCCRYAGCRTAWRWGGWYYYTTYYGGMTATT 27360
Indonesia-88065     AYRWAKGTACTRTRCGYTGYTTTKCGMTATCCRYMKCRTAWRWGGWYYYTTYYCGMTGTT 27360
                    .    .. .    *. *  *:    .    .       ..**    **   *     .

Bhutan-09015        YGTAKTYTAGTSRYTSWTAACSRAKRGGYYMYCACGATRCCGTTCYKAWYYYRYTRKGYY 27420
Bhutan-09024        YGTAKTYTAGTSRYTSWTAACSRAKRGGYYMYCACGATRCCGTTCYKAWYYYRYTRKGYY 27420
Bhutan-09027        YGTAKTYTAGTSRYTSWTAACSRAKRGGYYMYCACGATRCCGTTCYKAWYYYRYTRKGYY 27420
Bhutan-09030        YGTAKTYTAGTSRYTSWTAACSRAKRGGYYMYCACGATACCGTTCYKAWYYYRYTRKGYY 27420
Bhutan-09005        CGTWTKWAGCKSGTCCTWWRSCAWTRRRTYMCMRYRRKGTYKYYYYKCTTCCGTYRKAYY 27420
Indonesia-88035     CGTWTKWWGCKSGTCCTWWRSCAWTRRRTYMCMRYRRKGTYKYYYYKCTTCCGTYRKAYY 27420
Indonesia-88045     CGTWTKWWGCKSGTCCTWWRSCAWTRRRTYMCMRYRRKGTYKYYYYKCTTCCGTYRKAYY 27420
Indonesia-88065     CSWWTKWAGCKSGTCCTWWRSCAWTRRRTYMCMRYRRKGTYKYYYYKCTTCCGTYRKAYY 27420
                     .  ..: . .*   .    ..  .*   **      .       **.       **.**

Bhutan-09015        ARTGKRWKYCTTYTGSMAKWRGYGYGCYKGYGTAYGWAYMRGRTACCGYYTYAGRKTGTR 27480
Bhutan-09024        ARTGKRWKYCTTYTGSMAKWRGYGYGCYKGYGTAYGWAYMRGRTACCGYYTYAGRKTGTR 27480
Bhutan-09027        ARTGKRWKYCTTYTGSMAKWRGYGYGCYKGYGTAYGWAYMRGRTACCGYYTYAGRKTGTR 27480
Bhutan-09030        ARTGKRWGTCTTYTGSMAKWRGYGYGCYKGYGTAYGWAYMRGRTACCGYYTYAGRKTGTR 27480
Bhutan-09005        RAWRTGATYYCKTWGSARTWARCRYRYTGGCRWATRAWTCARGGGSYRTTYYWRRGWRTG 27480
Indonesia-88035     RATGTGATTYCKTWRSARTWARCRYRCTGRCRWRTAAWTCARGGGSYRTTYYWRRGWRCG 27480
Indonesia-88045     RATGTGATTYCKTWRSARTWARCRYRCTGRCRWRTAAWTCARGGGSYRTTYYWRRGWRCG 27480
Indonesia-88065     RAWRTGATYYCKTWGSARTWARCRYRYTGGCRWATRAWTCARGGGSYRTTYYWRRGWRTG 27480
                        .      .   *  .*    *                   ..     *  *

Bhutan-09015        RWYRTWKASGCMTARTAYWTKYYCAGYCRMAYYYMCMASTMCRTRRATCASTCYTRTCAR 27540
Bhutan-09024        RWYRTWKASGCMTARTAYWTKYYCAGYCRMAYYYMCMASTMCRTRRATCASTCYTRTCAR 27540
Bhutan-09027        RWYRTWKASGCMTARTAYWTKYYCAGYCRMAYYYMCMASTMCRTRRATCASTCYTRTCAR 27540
Bhutan-09030        RWYRTWKASGCMTARTAYWTKYYCAGYCRMAYYYMCMASTMCRTRRATCASTCYTRTCAR 27540
```

```
Bhutan-09005           RATAYWKRCATAYMGYRTTYKYCMGGTYAMATCTACMMCYMAAAARATYMSYYTKRYYRA 27540
Indonesia-88035        RATAYWKRSATAYMGYRTTYKYCMGRTYAMATCTAMMMCYMAAAARRKYMSYYTKRYYRA 27540
Indonesia-88045        RATAYWKRSATAYMGYRTTYKYCMGRTYAMATCTAMMMCYMAAAARRKYMSYYTKRYYRA 27540
Indonesia-88065        RATAYWKRCATAYMGYRTTYKYCMGGTYAMTTCTACMMCYMAAAARATYMSYYTKRYYRA 27540
                       *    ** ..          **  .    *:     * . *. : * .  *   .*

Bhutan-09015           GTGWYTTMYRYCCCAYSARKKCATTRWARYTAYRCYYKYRTYCRCATATGSYRYYWKKRK 27600
Bhutan-09024           GTGWYTTMYRYCCCAYSARKKCATTRWARYTAYRCYYKYRTYCRCATATGSYRYYWKKRK 27600
Bhutan-09027           GTGWYTTMYRYCCCAYSARKKCATTRWARYTAYRCYYKYRTYCRCATATGSYRYYWKKRK 27600
Bhutan-09030           GTGWYTTMYRYCCCAYSWRKKCATTRWARYTAYRCYYKYRTYCRCATATGSYRYYWKKRK 27600
Bhutan-09005           AYAWTKWMYGYYSYWTSARTGYRYYGWRRCKRYRMCYTYRWTYGMACMWACYACCKTTAK 27600
Indonesia-88035        ATAWTKWMYGYYSYWTSARTGCRYYGWRRCKRYGCCYTYRWTYGMRCAWACYACCKTTAK 27600
Indonesia-88045        ATAWTKWMYGYYSYWTSARTGYRYYGWRRCKRYGCCYTYRWTYGMRCAWACYACCKTTAK 27600
Indonesia-88065        AYAWTKWMYGYYSYWTSARTGCRYYGWRRCKRYRMCYTYRWTYGMACMWACYACCKTTAK 27600
                       . .* . ** * .   * *.      * * . *   *.**         ..*    .. *

Bhutan-09015           WRRARCYYMTRRTMRYRGRMGYWGTRWRMGGCAWWGGKKCRAYYAAGGAMAAAGYYTRAW 27660
Bhutan-09024           WRRARCYYMTRRTMRYGGRMGYWGTRWRMGGCAWWGGKKCGAYYAAGGAMAAAGYYTRAW 27660
Bhutan-09027           WRRARCYYMTRRTMRYGGRMGYWGTRWRMGGCAWWGGKKCRAYYAAGGAMAAAGYYTRAW 27660
Bhutan-09030           WRRARCYYMTRRTMRYRGRMGYWGTRWRMGGCAWWGGKKCRAYYAAGGAMAAAGYYTRAW 27660
Bhutan-09005           WRRARYTCCTARTCACGSAMRYARAAWRCRSYRTWARKKYGRYCRTKRRMTAGATCYARA 27660
Indonesia-88035        WRAMRYTCCCAAYCACGSAMRYARAAWRMRSYATWARKGYGRYCRTKRRMTWRATCYARA 27660
Indonesia-88045        WRAMRYTCCCAAYCACGSAMRYARAAWRMRSYATTARKKYGRYCRTKRRMTWRATCYARA 27660
Indonesia-88065        WRRARYTCCTARTCACGSAMRYARAAWRCRSYRTWARKKYGRYCRTKRRMTAGATCYARA 27660
                       **  *            . * *  : **  .    . *    *  :   *:  .

Bhutan-09015           GAWWWMRAWMAMKAGTRTTCSAYTGACTYKYGMRYRARYRRGCATYYGYASYTTKGYSKC 27720
Bhutan-09024           GAWWWMRAWMAMKAGTRTTCSAYTGACTYKYGMRYRARYRRGCATYYGYASYTTKGYSKC 27720
Bhutan-09027           GAWWWMRAWMAMKAGTRTTCSAYTGACTYKYGMRYRARYRRGCATYYGYASYTTKGYSKC 27720
Bhutan-09030           GAWWWMRAWMAMKAGTRTTCSAYTGACTYKYGMRYRARYRRGCATYYGYASYTTKGYSKC 27720
Bhutan-09005           RATTACARWACMGTCYRKYYGRYCAMGYYKYRMGTRRAYAGKYGKCYGTMSYYYWKCGTG 27720
Indonesia-88035        GMWWWCARWACMGTCYRKYYGRYCAMGYYKYRMGTRRAYAGKYGKCTRTMSYYYWKCGTG 27720
Indonesia-88045        GMWWWCARWACMGTCYRKYYGRYCAMGYYKYRMGTRRAYAGKYGKCTRTMSYYYWKCGTG 27720
Indonesia-88065        GATTACARWACMGTCYRKYYGRYCAMGYYKYRMGTRRAYAGKYGKCYGTMSYYYWKCGTG 27720
                               * .* :  *.  . * .   *** *  *  *    ..     **     ..

Bhutan-09015           RRRRARRTYKSWRMYYCKCMAKYATGMATMYAATAGRYRGYTGCSSYRRCWSSACKYASA 27780
Bhutan-09024           RRRRARRTYKSWRMYYCKCMAKYATGMATMYAATAGRYRGYTGCSSYRRCWSSACKYAGA 27780
Bhutan-09027           RRRRARRTYKSWRMYYCKCMAKYATGMATMYAATAGRYRGYTGCSSYRRCWSSACTTAGA 27780
Bhutan-09030           RRRRARRTYKSWRMYYCKCMAKYATGMATMYAATAGRYRGYTGCSSYRRCWSSACTTASA 27780
Bhutan-09005           RRGGRGAYCTGARACCYKACRTCRYKAWWCCGCATRRYRCTYRYGSCARGACCACTTGSW 27780
Indonesia-88035        RRGGRGAYCTGARACCYKACRTCRYKAWWCCGCATRRYRCTYRYGSCARGACCRYTTGSW 27780
Indonesia-88045        RRGGRGAYCTGARACCYKACRTCRYKAAWCCGCATRRYRCTYRYGSCARGACCRYTTGSW 27780
Indonesia-88065        RRGGRGAYCTGARACCYKACRTCRYKAAWCCGCATRRYRCTYRYGSCARGACCRYTTGSW 27780
                       **       .. *    *.  .         ..:: ***     .*  *  ..  . ..

Bhutan-09015           YGAAAYYCRRKYMRGRGRATGYRGATMAGRRYYGRTYARKYRYKATCKRYTTCKAYYAAY 27840
Bhutan-09024           TGRAAYYCAGKYMRGRGRATGYRGATMAGRRYYGRTYARKYRYKATCKRYTTCKAYYAAY 27840
Bhutan-09027           TGRAAYYCRRKYMRGRGRATGYRGATMAGRRYYGRTYARKYRYKATCKRYTTCKAYYAAY 27840
Bhutan-09030           YGRAAYYCAGKYMRGRGRATGYRGATMAGRRYYGRTYARKYRYKATCKRYTTCKAYYAAY 27840
Bhutan-09005           CAGMRTTAGRGCCRKAKGRGSTARWKAGARATCRRYYRAGYAYTWWMGKYWKTTMCYRWY 27840
Indonesia-88035        CAGMRTTAGGGCCRKAKGRGSTARWKAGARATCRRYYRAGYAYTWWMGKYWKTTMCYRWY 27840
Indonesia-88045        CAGMRTTAGRGCCRKAKGRGSTARWKAGARATCRRYYRAGYAYTWWMGKYWKTTMCYRWY 27840
Indonesia-88065        CAGMRTTAGRGCCRKAKGRGSTARWKAGARATCRRYYRAGYAYTWWMGKYWKTTMCYRWY 27840
```

```
                      .     .     *      .    . ..*    * *   * *.    :* . .  *  *

Bhutan-09015         YRYMRYRRYRRWMKYRYYRKRGKRAYYTKMYYGAAGYYGTYMYYYGMWRYYYRTWTKSYT 27900
Bhutan-09024         YRYMRYRGYRRWMKYRYYRKRGKRAYYTKMYYGAAGYYGTYMYYYGMWRYYYRTWTKSYT 27900
Bhutan-09027         YRYMRYRGYRRWMKTRYYRKRGKRAYYTKMYYGAAGYYGTYMYYYGMWRYYYRTWTKSYT 27900
Bhutan-09030         YRYMRYRRYRRWMKYRYYRKRGKRAYYTKMYYGAAGYYGTYMYYYGMWRYYYRTWTKSYT 27900
Bhutan-09005         TRYCRTAGYARACGYRYTAKGRGATTYCKAYYARMATCKWTATCCAATKTCCAKWYTATY 27900
Indonesia-88035      TRYCRTAGYARACGYRYTAKGRGRAYYCKAYYARMAYCKWTATCCAATKTCCAKWYTCCY 27900
Indonesia-88045      TRYCGTAGYARACGYRYTAKGRGRAYYCKAYYARMAYCKWTATCCAATKTCCAKWYTCCY 27900
Indonesia-88065      TRYCRTAGYARACGYRYTAKGRGATTYCKAYYARMATCKWTATCCAATKTCCAKWYTATY 27900
                      **     * *    **  *    : * * **.  .         .  :    .* ..

Bhutan-09015         YTGGGYTTYGTWRGGTYAKCYRYKAMKRSYYTKMCYCSMCYWMRCRYYAYTMATAYGMWA 27960
Bhutan-09024         YTGGGYYTYGTWRSGTYAKCYRYKAMKRSYYTKMCYCSMCYWMRCRYYAYTMATAYGMWA 27960
Bhutan-09027         YTGGGYYTYGTWRSGTYAKCYRYKAMKRSYYTKMCYCSMCYWMRCRYYAYTMATAYGMWA 27960
Bhutan-09030         YTGGGYYTYGTWRSGTYAKCYRYKAMKRSYYTKMCYCSMCYWMRCRYYAYTMATAYGMWA 27960
Bhutan-09005         YCSRSTCYTRYWGGCKYRGSTGYKGSKGCTYYTCGYTGCYCAAAYRYTCTCMWYRCRMWM 27960
Indonesia-88035      YCSRCTCYTRYWGGCKYRGSTGYKGSKGCTCYTCGYTGCCCTAAYRYTCCTMWYRCRMWM 27960
Indonesia-88045      YCSRSTCYTRYWGGCKYRGSTGYKGSKGCTCYTCGYTGCCCTAAYRYTCCTMWYRCGMWM 27960
Indonesia-88065      YCSRCTCYTRYWGGCKYRGSTGYKGSKGCTYYTCGYTGCYCAAAYRYTCTCMWYRCRMWM 27960
                     * .        * . .*  .  **. * .   .  * .       ** .  *     **

Bhutan-09015         YGTRWGKGCKYYGWSKTGCACACGRKMTCRRGCCRSYRWSYYSRYGMRYWTYYRKYTAYY 28020
Bhutan-09024         YGTRWGKGCKYYGWSKTGCACACGRKMTCRRGCCRSYRWSYYSRYGMRYWTYYRKYTAYY 28020
Bhutan-09027         YGTRWGKGCKYYGWSKTGCACACGRKMTCRRGCCRSYRWSYYSRYGMRYWTYYRKYTAYY 28020
Bhutan-09030         YGTRWGKGCKYYGWSKTGCACACGRKMTCRRGCCRSYRWSYYSRYGMRYWTYYRKYTAYY 28020
Bhutan-09005         TKYRASKRMKCTRWGKYKTCGCYAGTACYRRKYAACTAWSCCGGCKARYWYYCGGYKMTT 28020
Indonesia-88035      TKYRASKACKCTRWGKYKTCGCYAGTACYRRKYAACTAWSCCGGCKARYWYYCGGYKMTT 28020
Indonesia-88045      TKYRASKACKCTRWGKYKTCGCYAGTACYRRKYAACTAWSCCGGCKARYWYYCGGYKMTT 28020
Indonesia-88065      TKYRASKACKCTRWGKYKTCGCYAGTACYRRKYAACTAWSCCGGCKARYWYYCGGYKMTT 28020
                        * .*  *   *.*   . . . .   **  . .  **  .    *** *   *.

Bhutan-09015         MTYRTRRYTYTKCMYRSRWTCYCRAATGRGACGYMKCGAYYTYRACYRSGCTGYSRARGA 28080
Bhutan-09024         MTYRTRRYTYTKCMYRSRWTCYCRAATGRGACGYMKCGAYYTYRACYRSGCTGYSRARGA 28080
Bhutan-09027         MTYRTRRYTYTKCMYRSRWTCYCRAATGRGACGYMKCGAYYTYRACYRSGCTGYCAARGA 28080
Bhutan-09030         MTYRTRRYTYTKCMYRSRWTCYCRAATGRGACGYMKCGAYYTYRACYRSGCTGYCAARGA 28080
Bhutan-09005         AYYGKAAYGMWGCCCASRWGATYGGGWSRSWTKCCTTRRTTTTGMTCGGRYKSYGGARKW 28080
Indonesia-88035      AYYGKAAYGMWGACCASRWGATYGGGWSRSWTKCCTTRRTTWTGMTCGGRYKSYGGMRKW 28080
Indonesia-88045      AYYGKAAYGMWGACCASRWGATYGGGWSRSWTKCCTTRRTTWTGMTCGGRYKSYGGMRKW 28080
Indonesia-88065      AYYGKAAYGMWGCCCASRWGATYGGGWSRSWTKCCTTRRTTWTGMTCGGRYKSYGGMRKW 28080
                       * .  *    .   *** .   .. .*.     .            .  ..*   *

Bhutan-09015         MRTYSWTKKYRYCMRCATRCRASYRRTCGKACRRRRTRKWYWRCCGRYYRGTWAKYAWWT 28140
Bhutan-09024         MRTYSWTKKYRYCMRCATRCRASYRRTCGKACRRRRTRKWYWRCCGRYYRGTWAKYAWWT 28140
Bhutan-09027         MRTYSWTKKYRYCMRCATRCRASYRRTCGKACRRRRTRKWYWRCCGRYYRGTWAKYAWWT 28140
Bhutan-09030         MRTYSWTKKYRYCMRCATRCRASYRRTCGKACRRRRTRKWYWRCCGAYYRGTWAKYAWWT 28140
Bhutan-09005         CAWTSAYTRYRYYCGTCYRYAWGTAAWMRGRYRRGGYGGAYWRYYRGYYAKKWGKYRTAW 28140
Indonesia-88035      CAWTSAYTRYRYYCGTCYRYAWGTAAWMRGRYRRGGYGGAYWRYYRGYYAKKWGKYRTAW 28140
Indonesia-88045      CAWTSAYTRYRYYCGTCYRYAWGTAAWMRGRYGAGGYGGAYWRYYRGYYAKKWGKYRTAW 28140
Indonesia-88065      CAWTSAYTRYRYYCGTCYRYAWGTAAWMRGRYRRGGYGGAYWRYYRGYYAKKWGKYRTAW 28140
                         *  .:***    . *   .                 ***    **  .*.**

Bhutan-09015         MCSCGRMASSTRRTCGCRWYWRGTMKCYMKWWACGACTATAYGYSCTTRRTCTCMATRYT 28200
Bhutan-09024         MCSCGRMASSTRRTCGCRWYWRGTMKCYMKWWACGACTATAYGYSCTTRRTCTCMATRYT 28200
```

```
Bhutan-09027        MCSCGRMASSTRRTCGCRWYWRGTMKCYMKWWACGACTATAYGYSCTTRRTCTCMATRYT 28200
Bhutan-09030        MCSCGRMASSTRRTCGCRWYWRGTMKCYMKWWACGACTATAYGYSCTTRRTCTCMATRYT 28200
Bhutan-09005        MSCYRGMMSSYGAWMKSAATTRSYMTYYAKWWRYRTGCCWRTRYCMCAAAAMKYAGWAYY 28200
Indonesia-88035     MSCCGRMMSSYGAWCKSAATTRSYMTYYAKWWRYRTGCCWRTRYCMCAAAAMKYAGWAYY 28200
Indonesia-88045     MSCCGRMMSSYGAWCKSAATTRSYMTYYAKWWRYRTGCCWRTRYCMCAAAAMKYAGWAYY 28200
Indonesia-88065     MSCYRGMMSSYGAWMKSAATTRSYMTYYAKWWRYRTGCCWRTRYCMCAAAAMKYAGWAYY 28200
                    *..   * **      .    *. *. * ***   :  .    *.  :  : .  .  *

Bhutan-09015        YYARCATRGAYWKRYYGCRRTGTRTWSYGYYCSKKATGYCKRRWYYSWAYSYRRYAKAAA 28260
Bhutan-09024        YYARCATRGAYWKRYYGCRRTGTRTWSYGYYCSKKATGYCKRRWYYSWAYSYRRYAKAAA 28260
Bhutan-09027        YYARCATRGAYWKRYYGCRRTGTRTWSYGYYCSKKATGYCKRRWYYSWAYSYRRYAKAAA 28260
Bhutan-09030        YYARCATRGAYWKRYYGCRRTGTRTWSYGYYCSKKATGYCKRRWYYSWAYSYRRYAKAAA 28260
Bhutan-09005        TYTAAWWATGTWGRTCTYGAYSGGYAGTAYTYCTKMATTMGRGTTCSAWYCYGGTRTWRT 28260
Indonesia-88035     TYTAAWWATGTWGRTCTYGAYSGGYAGTAYTYCTKMATTMGRGTTCSAWYCYGGTRTWGT 28260
Indonesia-88045     TYTAAWWATGTWGRTCTYGAYSGGYAGTAYTYCTKMATTMGRGTTCSAWYCYGGTRTWGT 28260
Indonesia-88065     TYTAAWWATGTWGRTCTYGAYSGGYAGTAYTYCTKMATTMGRGTTCSAWYCYGGTRTWGT 28260
                     *: .    . * *       .    . .*  ..* :    *    *  *.*    .  :

Bhutan-09015        YWYCTGMCGYRSKCWWTRTRTCWYSYRSGKYRTRMCRKRAGTTSYWYKAGWMRYYYTKGT 28320
Bhutan-09024        YWYCTRMCGYRSKCWWTRTRTCWYSYRSGKYRTRMCRKRAGTTSYWYKAGWMRYYYTKGT 28320
Bhutan-09027        YWYCTGMCGYRSKCWWTRTRTCWYSYRSGKYRTRMCRKRAGTTSYWYKAGWMRYYYTKGT 28320
Bhutan-09030        YWYCWGMCGYRSKCWWTRTRTCWYSYRSGKYRTRMCRKRAGTTSYWYKAGWMRYYYTKGT 28320
Bhutan-09005        TYCYWGAYKTASGSTAYAKAKYTTSYACCKTAYACSGTRGAWYSTWTGTSTCRCTTKTRY 28320
Indonesia-88035     TYCYTGAYKTASGSTAYAKAKYTTSYACCKTAYACSGTRGAWYSTWTGTSTCRCTTKTRY 28320
Indonesia-88045     TYCYTGAYKTASGSTAYAKAKYTTSYACCKTAYACSGTRGAWYSTWTGTSTCRCTTKTRY 28320
Indonesia-88065     TYCYTGAYKTASGSTAYAKAKYTTSYACCKTAYACSGTRGAWYSTWTGTSTCRCTTKTRY 28320
                     :         * .    . .   ** . *     . .*..  * *  :.  *   ..

Bhutan-09015        TRYYKWWRYYKAYYWYTATMRTTTKARRTRSYCWRRAMACGTYYYRGRRTAWGRGCCCKR 28380
Bhutan-09024        TRYYKWWRYYKAYYWYTATMRTTTKARRTRSYCWRRAMACGTYYYRGRRTAWGRGCCCKR 28380
Bhutan-09027        KRYYKWWRYYKAYYWYTATMRTTTKARRTRSYCWRRAMACGTYYYRGRRTAWGRGCCCKR 28380
Bhutan-09030        KRYYKWWRYYKAYYWYTATMRTTTKARRTRSYCWRRAMACGTYYYRGRRTAWGRGCCCKR 28380
Bhutan-09005        TRYCGTWGYSGCCTACYCYCAYKYTRGRWRCTYTAGGCGMTCYYCASRRARARARYTAGG 28380
Indonesia-88035     TRYCGTWGYSGCCTACYCYCAYKYTRGRWRCTYTAGGCGMTCYYCASRRARARARYTAGG 28380
Indonesia-88045     TRYCGTWGYSGCCTACYCYCAYKYTRGRWRCTYTAGGCGMTCYYCASRRARARARYTAGG 28380
Indonesia-88065     TRYCGTWGYSGCCTACYCYCAYKYTRGRWRCTYTAGGCGMTCYYCASRRARARARYTAGG 28380
                    .**   * *  .     .    . .  * *.     . .   **  .**:       .

Bhutan-09015        SCTACRKRGTTYCYCYKSTRGKSYYYWAKRCSWKMKRWRRGYKTACRYRGYYRRRRGCTR 28440
Bhutan-09024        SCTACRKRGTTYCYCYKSTRGKSYYYWAKRCSWKMKRWRRGYKTACRYRGYYRRRRGCTR 28440
Bhutan-09027        SCTACRKRGTTYCYCYKSTRGKSYYYWAKRCSWKMKRWRRGYKTACRYRGYYRRRAGCTR 28440
Bhutan-09030        SCTACRKRGTTYCYCYKSTRGKSYYYWAKRCSWKMKRWRRGYKTACRYRGYYRRAAGCTR 28440
Bhutan-09005        SYKMGRKRTYYYYCYTKSTARGGYYYARGRMSWTCGRWRRRTGWWGGCGSYYRRRGAYYR 28440
Indonesia-88035     SYKMGRKRTYYYYCYTKSTARGGYYYARGRMSWTCGRWRRRTGWWGGCGSYYRRRGAYYR 28440
Indonesia-88045     SYKMGRKRTYYYYCYTKSWRRGGYYYARGRMSWTCGRWRRRTGWWGGCGSYYRRRGAYYR 28440
Indonesia-88065     SYKMGRKRTYYYYCYTKSTARGGYYYARGRMSWTCGRWRRRTGWWGGCGSYYRRRGAYYR 28440
                    * .  ***   *    **    .***   * **.  ****         .****  .  *

Bhutan-09015        AKTAYTGCAGGWRCAAGACRRAYAYTRWYAMWCYRTTGCRAYWCWYAAACTRRTAYGACR 28500
Bhutan-09024        AKTAYTGCAGGWRCAAGACRRAYAYTRWYAMWCYRTTGCRAYWCWYAAACTRRTAYGACA 28500
Bhutan-09027        AKTAYTGCAGGWRCAAGACRRAYAYTRWYAMWCYRTTGCRAYWCWYAAACTRRTAYRACR 28500
Bhutan-09030        AKTAYTGCAGGWRCAAGACRRAYAYTRWYAMWCYRTTGCRAYWCWYAAACTRRTAYRACR 28500
Bhutan-09005        WGKMTYRMRKRWGYRWRMYARMYRCYRWTCAAYCGYYATARCWYTYRGTTGAAKRTGTYA 28500
Indonesia-88035     WGKMTYRMAGRWGYRWRMYARMYRCYRWTCAAYCGYYATARCWYTYRGTTGAAKRTGTYA 28500
```

```
Indonesia-88045      WGKMTYRMAGRWGYRWRMYARMYRCYRWTCAAYCGYYATARCWYTYRGTTGAAKRTGTYA 28500
Indonesia-88065      WGKMTYRMRKRWGYRWRMYARMYRCYRWTCAAYCGYYATARCWYTYRGTTGAAKRTGTYA 28500
                       .        *        * *   ** .       .    *  * .:    .   :

Bhutan-09015         YWYRMCSTRCAATMGTAKRRKTKRRRGCRSMAGATRRCYWYRYRCRASACGAGGCGATCW 28560
Bhutan-09024         YWYRMCSTRCAATMGTAKRRKTKRRRGCRSMAGATRRCYWYRYRCRASACGAGGCGATCW 28560
Bhutan-09027         YWYRMCSTRCAAYMGTAKRRKTKRRRGCRSMAGATRRCYWYRYRCRASACGAGGCGATCW 28560
Bhutan-09030         YWCAMCSTRCAAYMGTAKRRKTKRRRGCRSMAGATRRCYWYRYRCRASACGAGGCGATCW 28560
Bhutan-09005         TATGAMCWRYTATCRCGKRRTKGAARRYRGCRKMYGRTYWTGTAYAGGTYRMSSYKMYYA 28560
Indonesia-88035      TATGAMCWRYTWTCRCGKRRTKGAARRYRGCRKMYGATYWTGTAYAGGTYRMSSYKMYCA 28560
Indonesia-88045      TATGAMCWRYTWTCRCGKRRTKGAARRYAGCRKMYGATYWTGTAYAGGTYRMSSYKMYYA 28560
Indonesia-88065      TATGAMCWRYTATCRCGKRRTKGAARRYRGCRKMYGRTYWTGTAYAGGTYRMSSYKMYYA 28560
                           . * :     .***..   *   .        **      ..:   ..

Bhutan-09015         YWYSKMCYYRMMGMWCTKSGYYAKCRKYKRYTYGCACGAGGMRSARCCYWTWRRARRCMS 28620
Bhutan-09024         YWYSKMCYYRMMGMWCTKSGYYAKCRKYKRYTYGCACGAGGMRSARCCYWTWRRARRCMS 28620
Bhutan-09027         YWYSGCCYYRMMGMWCTKSGYYAKCRKYKRYTYGCACGAGGMRSARCCYWTWRRARRCMS 28620
Bhutan-09030         YWYSKMCYYRMMGMWCTKSGYYAKCRKYKRYTYGCACGAGGMRSARCCYWTWRRARRCMS 28620
Bhutan-09005         YATGGCTCTRMAKAASWGGATCRGYGKCKGTKYRYMYKWARAGSRRTSYWCTAARGGGCC 28620
Indonesia-88035      YATGGCTCTRMAKAASWGGATCRGYGKCKGTKYRYCYKWARAGSRRTSYWCTAARGGGCC 28620
Indonesia-88045      YATGGCTYTRMAKAASWGGATCRGYGKCKGTKYRYMYKWARAGSRRTSYWCTAARGGGCC 28620
Indonesia-88065      YATGGCTYTRMAKAASWGGATCRGYGKCKGTKYRYMYKWARAGSRRTSCWCTAARGGGCC 28620
                     *  .     **    .  ..      * *  .*      .   * * . *         .

Bhutan-09015         GAYGRCSCGGRRRTTYTCKTWYGRMRYYKCRGGYTYRRYWYKRRRRRWTGYSRMGTTYMG 28680
Bhutan-09024         RAYGRCSCGGRRRTTYTCKTWYGRMRYYKCRGGYTYRRYWYKRRRRRWTGYSRMGTTYMG 28680
Bhutan-09027         RAYGRCSCGGRRRTTYTCKTWYGRMRYYKCRGGYTYRRYWYKRRRRRWTGYSRMGTTYMG 28680
Bhutan-09030         GAYGRCSCGGRRRTTYTCKTWYGRMRYYKCRGGYTYRRYWYKRRRRRWTGYSRMGTTYMG 28680
Bhutan-09005         GWTRRYSARSRRATCYYYGYWYARCATYGYAGTCWCAATWYGGARGGAKKCGAAKYYCAS 28680
Indonesia-88035      GWTRRYSARSRRAYCYYYGYWYARCATYGYAAGCWCAATWYGGARGGAKKCGAAKYYCAS 28680
Indonesia-88045      GWTRRYSARSRRAYCYYYGYWYARCATYGYAAGCWCAATWYGGARGGAKKCGAAKYYCAS 28680
Indonesia-88065      GWTRRYSARSRRAYCYYYGYWYARCATYGYAGTCWCAATWYGGARGGAKKCGAAKYYCAS 28680
                         * *. .**   *    **.*   *   .       **   *   .  .       .

Bhutan-09015         CRKAGYGAGYRYTRGSYWYYRMMAYWYAYGGAGKCTCYGYYACCKRYAAWAMWRYRGTAY 28740
Bhutan-09024         CRKAGYGAGYRYTRGSYWYYRMMAYWYAYGGAGKCTCYGYYACCKRYAAWAMWRYRGTAY 28740
Bhutan-09027         CRKAGYGAGYRYTRGSYWYYRMMAYWYAYGGAGKCTCYGYYACCKRYAAWAMWRYRGTAY 28740
Bhutan-09030         CRKAGYGAGYRYTRGSYWYYRMMAYWYAYGGAGKCTCYGYYACCKRYAAWAMWRYRGTAY 28740
Bhutan-09005         YATTRTTGACATWRSCCTCTRMCRCWYRYRRCGKCYCYRYTTMMGGYGGWRMWAYAAYCT 28740
Indonesia-88035      YATTRTTGACATWRSCCTCTRMCRCWYRYRGCKTYTYTRYTTMMGGYGGWRMWAYAAYCT 28740
Indonesia-88045      YATTRTTGACATWRSCCTCTRMCRCWYGYRGCKTYTYTRYTTMMGGYGGWRMWAYAAYCT 28740
Indonesia-88065      YATTRTTGACATWRSCCTCTRMCRCWYRYRRCGKCYCYRYTTMMGGCGGWRMWAYAAYCT 28740
                       .:   ..    *..    **   ** *  . .     * :     ..* ** * . .

Bhutan-09015         RGRCARAYWGYYCCKCGAACYWYWKSYRARYCWYMGKTTYGGRATYCTKGAYRSTYMYWW 28800
Bhutan-09024         RGRCARAYWGYYCCKCGAACYWYWKSYRARYCWYMGKTTYGGRATYCTKGAYRSTYMYWW 28800
Bhutan-09027         RGRCARAYWGYYCCKCGAACYWYWKSYRARYCWYAGKTTYGGRATYCTKGAYRSTYMYWW 28800
Bhutan-09030         RGRCARAYWGYYCCKCGAACYWYWKSYRARYCWYMGKTTYGGRATYCTKGAYRSTYMYWW 28800
Bhutan-09005         RRRTARWTARTCMYTGCRRCYATYTGCARRCSATCRTTTYRRGRWTSTTRWTACKYAYWW 28800
Indonesia-88035      RRRTARWTARTCCYTGCRRMYATYTGCARRYCAYCRTYYYRRGRWYCYTRWTACKYAYWW 28800
Indonesia-88045      RRRTMRWTARTCCYTGCRRMYATYTGCARRYCAYCRTYYYRRGRWYCYTRWTACKYAYWW 28800
Indonesia-88065      RRRTARWTARTCCYTGCRRCYATYTGCARRCSATCRTTTYRRGRWTSTTRWTACKYAYWW 28800
                     * *  *        .     *  :..   * .    .  *      . .    ..* ***
```

```
Bhutan-09015        TRYACYARYCYWGTYRKMYCYMYRARYTAAKAYYMCCAYWSSRGATYRAGRGGCMRATCG 28860
Bhutan-09024        TRYACYARYCYWGTYRKMYCYMYRARYTAAKAYYMCCAYWSSRGATYRAGRGGCMRATCG 28860
Bhutan-09027        TRYACYARYCYWGTYRKMYCYMYRARYTAAKAYYMCCAYWSSRGATYRAGRGGCMRATCG 28860
Bhutan-09030        TRYACYARYCYWGTYRKMYCYMYRARYTAAKAYYMCCAYWSSRGATYRAGRGGCMRATCG 28860
Bhutan-09005        KGYRTYRGYYCARYYRGACSTACAGGCTWRTWYCMTGWTTGCRRRYYRRRRRACMRGKCK 28860
Indonesia-88035     KGYRTYAGYYCAGYYRKACSYACAGGCYARTWYCMTGWTTGCRRRYYRRRGRRYCGGKMG 28860
Indonesia-88045     KGYRTYAGYYCAGYYRKACSYACAGGCYARTWYCMTGWTTGCRRRYYRRRGRRYCGGKMG 28860
Indonesia-88065     KGYRTYRGYYCARYYRGACSTACAGGCTWRTWYCMTGWTTGCRRRYYRRRRRACMRGKCK 28860
                    . *  *  *     **   .    .     . * *     ..*   **        ..

Bhutan-09015        MCRYARYGGYRMKYTTGCTRYKCCAKRSRWCWRYCRKMWYYRMCYARRRWASWYARRCYY 28920
Bhutan-09024        MCRYARYGGYRMKYTTGCTRYKCCAKRSRWCWRYCRKMWYYRMCCARRRWASWYARRCYY 28920
Bhutan-09027        MCRYARYGGYRMKYTTGCTRYKCCAKRSRWCWRYCRKMWYYRMCCARRRWASWYARRCYY 28920
Bhutan-09030        MCRYARYGGYRMKYTTGCTRYKCCAKRSRWCWRYCRKMWTYRMCCARRRWASWYARRCYY 28920
Bhutan-09005        CCGCWACGSTAMKYYWRYGRYGYTWTGCGWSTRYSAAMWTYACYCRGRRTRGTCMAGCTT 28920
Indonesia-88035     CYGCWACRGTAMKYYWRYGRYGYTWTGCGWCTRYSAAMWYYACCCGGRRTRSWCAAGYTT 28920
Indonesia-88045     CYGCWACRGTAMKYYWRYGRYGYTWTGCGWCTRYSAAMWYYACCCGGRRTRSWCAAGYTT 28920
Indonesia-88065     CCGCWACGGTAMKYYWRYGRYGYTWTGCGWSTRYSAAMWYYACCCRGRRTRGTCMAGCTT 28920
                            .  ***     **    . . *. **.  ** *      **  .

Bhutan-09015        MRWRGAKAAGTCGAMYTRCYAGTYRWYRTARAGYRAWYTTKSACYCRRTGTTKKSCSRRM 28980
Bhutan-09024        MRWRGAKAAGTCGAMYTRCYAGTYRWYRTARAGYRAWYTTKSACYCRRTGTTKKSCSRRM 28980
Bhutan-09027        MRWRGAKAAGTCGAMYTRCYAGTYRWYRTARAGYRAWYYTKSACYCRRTGTTKKSCSRRM 28980
Bhutan-09030        MRWRGAKAAGTCGAMYTRCYAGTYRWYRTARAGYRAWYTTKSACYCRRTGTTKKSCSRRM 28980
Bhutan-09005        MGWRRCGRMRCCACMYYRTCGKYTGTTAWWRTRYAAWYYKGSAYYSRAAGTTTKSYCAAC 28980
Indonesia-88035     MGWRRCGRMRYMACMYYATCGKYTGTTAWWRTRYARTYTKGSCYYSRAARYCTKSYCAAC 28980
Indonesia-88045     MGWRRCGRMRYMACMYYRTCGKYTGTTAWWRTRYARTYTKGSCYYSRAARYCTKSYCAAC 28980
Indonesia-88065     MGWRRCGRMRCCACMYYRTCGKYTGTTAWWRTRYAAWYYKGSAYYSRAAGYTTKSYCAAC 28980
                    * ** .      ..**    .         *: *   * . *. *.* :   .** .

Bhutan-09015        WSCCYYCKCACGRKKYKAWSGTYCRAWRWAWYRGAMRRAYGATKWYKSWAYCGTMTSYRY 29040
Bhutan-09024        WSCCYYCKCACGRKKYKAWSGTYCRAWRWAWYRGAMRRAYGATKWYKSWAYCGTMTSYRY 29040
Bhutan-09027        WSCCYYCKCACGRKKYKAWSGTYCRAWRWAWYRGAMRRAYGATKWYKSWAYCGTMTSYRY 29040
Bhutan-09030        WSCCYYCKCACGRKKYKAWSGTYCRAWRWAWYRGAMRRAYGATKWYKSWAYCGTMTSYRY 29040
Bhutan-09005        AGYMCCYKYWAGRTGCKMWSRYYSGRARWRACGKWCRGCCRGCGATGCARYTGYMCGTAT 29040
Indonesia-88035     AGYMCCYKYTARRTGCKMWSRYYCGRARWRACGKWCRGCCGGCGATGCARYTAYMCGTAT 29040
Indonesia-88045     AGCCCCYKYTARRTGCKMWSRYYCGRARWRACGKWCRGCCGGCGATGCARYTAYMCGTAT 29040
Indonesia-88065     AGYMCCYKYWAGRTGCKMWSRYYSGRARWRACGKWCRGCCRGCGATGCARYTGYMCGTAT 29040
                     .     *  . *.  * **  *.   **       * .  .     .  * . * .

Bhutan-09015        AGTWTTMWKCGARKGARGTRGAYKTCWMYKSYYSWCWRYTCTSAMYRWRKTRYKTYSWMM 29100
Bhutan-09024        AGTWTTMWKCGARKGARGTRGAYKTCWMYKSYYSWCWRYTCTSAMYRWRKTRYKTYSWMM 29100
Bhutan-09027        AGTWTTMWKCGARKGARGTRGAYKTCWMYKSYYSWCWRYTCTSAMYRWRKTRYKTYSWMM 29100
Bhutan-09030        AGTWTTMWKCGARKGARGTRGAYKTCWMYKSYYSWCWRYTCTSAMYRWRKTRYKTYSWMM 29100
Bhutan-09005        WSYTYTCWKYRGGGKRRRYATGCTYYAMTKSTCGWYTRYWCYSRMYRAGKYATTKTSWMA 29100
Indonesia-88035     ASYTYKCWKYRGGGTGRRTATGCTYYAMYKSTCGWYTRYWYYCRMYRAGKYATTKTSWMA 29100
Indonesia-88045     ASYTYKCWKYRGGGTGRRTATGCTYYAMYKSTCGWYTRYWYYCRMYRAGKYATTKTSWMA 29100
Indonesia-88065     WSYTYTCWKYRGGGTGRRTATGCTYYAMTKSTCGWYTRYWCYSRMYRAGKYATTKTSWMA 29100
                     .   . **  .    *    . .   * **  .*  **   . ***  *   .. ***

Bhutan-09015        KRSYGKASWYMWYSAWYKGGYTMYYGMTCKYGTRAMYTYMYRRRTYCRCTTYYRYKTRRG 29160
Bhutan-09024        KRSYGKASWYMWYSAWYKGGYTMYYGMTCKYGTRAMYTYMYRRRTYCRCTTYYRYKTRRG 29160
Bhutan-09027        KRSYGKASWYMWYSAWYKGGYTMYYGMTCKYGTRAMYTYMYRRRTYCRCTTYYRYKTRRG 29160
Bhutan-09030        KRSYGKASWYMWYSAWYKGGYTMYYGMTCKYGTRAMYTYMYRRRTYCRCTTYYRYKTRRG 29160
```

```
Bhutan-09005        GRGTKKTCACAWYSMATGSGTKAYYKATYTCCWRGMTACATRGRYYAGYKWCYRTGCRRK 29160
Indonesia-88035     GRGTKKWSWCAWYSMATGSATKAYCKAYYTCCWRGMTACATRGRYYAGYKWCYRTGCRRK 29160
Indonesia-88045     GRGTKKWSWCAWYSMATGSATKAYCKAYYTCCWAGMTACATRGRYYAGYKWCYRTGCRRK 29160
Indonesia-88065     GRGTKKTCACAWYSMATGSGTKAYYKATYTCCWRGMTACATGGRYYAGYKWCYRTGCRRK 29160
                     *.  * .   ***    .. . *     .    .* :     * *.  .  **   **

Bhutan-09015        YSYKRYTCMCKYACCYRRRCYRTCTKCAARATKASYYWCARRTSCYYTWWRRCYACKYTR 29220
Bhutan-09024        YSYKRYTCMCKYACCYRRRCYRTCTKCAARATKASYYWCARRTSCYYTWWRRCYACKYTR 29220
Bhutan-09027        YSYKRYTCMCTYACCYRRRCYRTCTKCAARATKASYYWCARRTSCYYTWWRRCYACKYTR 29220
Bhutan-09030        YSYKRYTCMCKYACCYRRRCYRTCTKCAARATKASYYWCARRTSCYYTWWRRCYACKYTR 29220
Bhutan-09005        TSTTRTYMCMTYRAMTRRATTGYTYKSRTARYTMGCYWYGRATCMTTYAAARYTGCTYCA 29220
Indonesia-88035     TSTTRTYMCCTYRAMYRRRCTGYTYKSRTARTTMGCYWYGRAKCMTTYAAARYTGYTYCA 29220
Indonesia-88045     TSTTRTYMCCTYRAMYRRRCTGYTYKSRTARTTMGCYWYGRAKCMTTYAAARYTGYTYCA 29220
Indonesia-88065     TSTTRTYMCMTYRAMTRRATTGYTYKSRTARYTMGCYWYGRATCMTTYAAARYTGCTYCA 29220
                     * .*     .* .  **       *. :   . . ** .* ..       *  . .*

Bhutan-09015        YYYYWRAKWYWRWWYTYRMTCCCRTKYRKRWTKMKYYACSGGRYMACGMKCMGWTYWGSG 29280
Bhutan-09024        YYYYWRAKWYWRWWYTYRMTCCCRTKYRKRWTKMKYYACSGGRYMACGMKCMGWTYWGSG 29280
Bhutan-09027        YYYYWRAKWYWRWWYTYRMTCCCRTKYRKRWTKMKYYACSGGRYMACGMKCMGWTYWGSR 29280
Bhutan-09030        YYYYWRAKWYWRWWYTYRMTCCCRTKYRKRWTKMKYYACSGGRYMACGMKCMGWTYWGSR 29280
Bhutan-09005        TTTCAGRKWYWGWWYKYRCCGMMRYKYGTRAWKAGCCWYSRGATAWAAMKACRAYCWASG 29280
Indonesia-88035     TTTYAGGKWYWGWWYKYRCCGMMRYKYGTRAWKAGCCWYSGRATAAAAMKACRATCWASG 29280
Indonesia-88045     TTTYARRKWYWGWWYKYRCCGMMRYKYGTRAWKAGCCWYSGGATAWAAMKACRATCWASG 29280
Indonesia-88065     TTTCAGRKWYWGWWYKYRCCGMMRYKYGTRAWKAGCCWYSRGATAWAAMKACRAYCWASG 29280
                           **** ***.**     * ** .*  *      *      ..**.      *.*

Bhutan-09015        TWKYYKKYYAYYYAASYGRAWRRCRSCRRYAASWYTRGTRATCWWWYSYKGSRATKYCYA 29340
Bhutan-09024        TWKYYKKYYAYYYAASYGRAWRRCRSCRRYAASWYTRGTRATCWWWYSYKGSRATKYCYA 29340
Bhutan-09027        KWKYYKKYYAYYYAASYGRAWRRCRSCRRYAASWYTRGTRATCWWWYSYKGSRATKYCYA 29340
Bhutan-09030        TWKYYKKYYAYYYAASYGRAWRRCRSCRRYAASWYTRGTRATCWWWYSYKGSRATKYCYA 29340
Bhutan-09005        TWKMYTKYYATCTACSCRRRTGRYGSSRAYGGGWCCAGCRCYSATAYSCKSSRRYTTYYW 29340
Indonesia-88035     TWKMYTKYCWYCTWCCCRRRTGRYGSSRAYGGGWCCAACACYSATATSCKSSRRYTTYYW 29340
Indonesia-88045     TWKMYTKYCWYCTWCCCRRRTGRYGSSRAYGGGWCCAACACYSATATSCKSSRRYTTYYW 29340
Indonesia-88065     TWKMYTKYYWYCTACSCRRRTGRYGSSRAYGGGWCCAGCRCYSATAYSCKSSRRYTTYYW 29340
                    .** *.**      ..  *   *  *.* *...*   .  . .    * *.**  .  *

Bhutan-09015        YGCYYYCTTYMYAWSTTATAWSWKRYMRAGCWTCKYMATRRRRWTAMTGGYCKTYYCGMG 29400
Bhutan-09024        YGCYYYCTTYMYAWSTTATAWSWKRYMRAGCWTCKYMATRRRRWTAMTGGYCKTYYCGMG 29400
Bhutan-09027        YGCYYYCTTYMYAWSTTATAWSWKRYMRAGCWTCKYMATRRRRWTAMTGGYCKTYYCGMG 29400
Bhutan-09030        YGCYYYCTTYMYAWSTTATAWSWTRYMRAGCWTCKYMATRRRRWTAMTGGYCKTYYCGMG 29400
Bhutan-09005        YRYCTCMAYWCCRTGYTRWWWCATRYARRTYAYYKYARCGARRAYWMTKRYSKTCYSGCR 29400
Indonesia-88035     YRYCTCMATACCRWGYYRWWWCATGYARRGYAYYKYARCGAAAAYWMYKRYSKKCYSRCR 29400
Indonesia-88045     YRYCTCMATACCRWGYYGWWWCATGYARRGYAYYKYARCGAAAAYWMYKRYSKKCYSRCR 29400
Indonesia-88065     YRYCTCMAYWCCRTGYTRWWWCATRYARRTYAYYKYARCGARRAYWMTKRYSKTCYSGCR 29400
                    *      :      .     *. . * *      **          *   *.*. *.

Bhutan-09015        YKYYTCGMAAKKKAYRACTSKRYWMYKRYWCRCRYWRTGKATTCTASWMYRWGARTATTT 29460
Bhutan-09024        YKYYTCGMAAKKKAYRACTSKRYWMYKRYWCRCRYWRTGKATTCTASWMYRWGARTATTT 29460
Bhutan-09027        YKYYTCGMAAKKKAYRACTSKRYWMYKRYWCRCRYWRTGKATTCTASWMYRWGARTATTT 29460
Bhutan-09030        YKYYTCGMAAKKKAYRACTSKRYWMYKRYWCRCRYWRTGKATTCTASWMYRWGARTATTT 29460
Bhutan-09005        TGCTGCSAMMGGTRTAMYWSKRTTSYTGTTYASATWAYSGMTYYKWSWMYGARRRYGYWY 29460
Indonesia-88035     TKCTTYSAAMGGTRTAMYWSKRTTSYTGTTYASATWAYSTMAYYKWSWMYGARRRYRYWY 29460
Indonesia-88045     TKCTTYSAAMGGTRTAMYWSKRTTSYTGTTYASATWAYSKMAYYKWSWMYGARRRYRYWY 29460
Indonesia-88065     TGCTGCSAAMGGTRTAMYWSKRTTSYTGTTYASATWAYSGMTYYKWSWMYGARRRYRYWY 29460
```

```
                           .     .      ***   *.     .  *  .  :  . ****    *

Bhutan-09015         TTKACSAYTRTYRTGMAARRCGYAGYAGGRMGSRRKMSKYKWRRGACCRAYYAARRTRRG 29520
Bhutan-09024         TTKACSACTGTYRTGMAARRCGYAGYAGGRMGSRRKMSKYKWRRGACCRAYYAARRTRRG 29520
Bhutan-09027         TTKACSAYTRTYRTGMAARRCGYAGYAGGRMGSRRKMSKYKWRRGACCRAYYAARRTRRG 29520
Bhutan-09030         TTKACSAYTRTYRTGMAARRCGYRKYAGGRMGSRRKMSKYKWRRGACCRAYYAARRTRRG 29520
Bhutan-09005         YYTRSCRYYRYYRYSAGRRRASYAGCGKRGARGGRKCCGYTWGARACYGWYCMGARKAGA 29520
Indonesia-88035      CYTRSCRYYRYYRYSAGARRASYAGYRKRGARGGRKCSGYTWGARRYYGWYCMGARTAGG 29520
Indonesia-88045      CYTRSCRYYRYYRYSAGARRASYAGYRKRGARGGRKCSGYTWGARRYYGWYCMGARTAGG 29520
Indonesia-88065      YYTRSCRYYRYYRYSAGRRRASYRKCGKRGARGGRKCCGYTWGARACYGWYCMGARKAGA 29520
                       . ..     ** . . **..*         . ** . *.*        *  . *.  .

Bhutan-09015         GRYYGCRTRKATSRTATAGAGYATTRKYRYYGTKKGTTTGYRARTRGAYWSGARRTWMRR 29580
Bhutan-09024         GRYYGCRTRKATSRTATAGAGYATTRKYRYYGTKKGTTTGYRARTRGAYWSGARRTWMRR 29580
Bhutan-09027         GRYYGCRTRKAWSRTATAGAGYATTRKYRYYGTKKGTTTGYRARTRGAYWSGARRTWMRR 29580
Bhutan-09030         GRYYGCRTRKATSRTATAGAGYATTRKYRYYGTKKGTTTGYRARTRGAYWSGARRTWMRR 29580
Bhutan-09005         RGCCCYGWAKMTGGTRTARRRYRYYAGTRCTRTKGSAWTRYAMRTRRMTACGARAYTMAA 29580
Indonesia-88035      GGCCCYGWAKMTGGCAWRRRRYRYYAGTRCTRWKGSAWKRYAARKRRMTACKRGAYTAAA 29580
Indonesia-88045      GGCCCYGWAKMTGGCAAGRRRYRYYAGTRCTRWKGSAWKRYAARKRRMTACKRGAYTAAA 29580
Indonesia-88065      RGCCCYGWAKMTGGTRTARRRYRYYAGTRCTRTKGSAWTRYAMRTRRMTACGARAYTMAA 29580
                              *  .        *      *    * .: . *  *.*    .

Bhutan-09015         ACAYYCWMWTWGSGWRGMAYYYGCTRYRGGRWRYTAKRSRMYKGRRTMGYWYRTYMTYTS 29640
Bhutan-09024         ACAYYCWMWTWGSGWRGMAYYYGCTRYRGGRWRYTAKRSRMYKGRRTMGYWYRTYMTYTS 29640
Bhutan-09027         ACAYYCWMWTWGSGWRGMAYYYGCTRYRGGRWRYTAKRSRMYKGRRTMGYWYRTYMTYTS 29640
Bhutan-09030         ACAYYCWMWTWGSGWRGMAYYYGCTRYRGGRWRYTAKRSRMYKGRRTMGYWYRTYMTYTS 29640
Bhutan-09005         WMRYTGTAAYTSSKWGACCCYYSSYAYATRAARTCRGRSRCYKRAAYAKCATAYYCYYYG 29640
Indonesia-88035      AMRYTGTACYTGSKWGACMCYYSSYAYATRAARTCRGASRMYKGAAYAGCATATYCYYYG 29640
Indonesia-88045      AMRYTGTACYTGSKWGACMCYYSSYAYATRAARTCRGASRMYKGAAYAGCATATYCYYYG 29640
Indonesia-88065      WMRYTGTAAYTSSKWGACCCYYSSYAYATRAARTCRGRSRMYKRAAYAKCATAYYCYYYG 29640
                        *       .* * .   **..  *     *     ** **           *  * .

Bhutan-09015         MCGTCCMRKYRTRGSCGTCRRKGYRTTACMKYSYRRRYKYCYSYSRYYRRCMAYGACKGW 29700
Bhutan-09024         MCGTCCMRKYRTRGSCGTCRRKGYRTTACMKYSYGRATKYCYSYSRYYRRCMAYGACKGW 29700
Bhutan-09027         MCGTCCMRKYRTRGSCGTCRRKGYRTTACMKYSYGRATKYCYSYCRYYRRCMAYGACKGW 29700
Bhutan-09030         MCGTCCMRKYRTRGSCGTCRRKGYRTTACMKYSYGRATKYCYSYSRYYRRCMAYGACKGW 29700
Bhutan-09005         MYSCYSCRGTGTARGMGKMGRKKCAWYRMCKYSYRAATKTYTGYGGCYAAAMMYWRTTRT 29700
Indonesia-88035      MCSCYCCRGTGWARGMRKMGRKGCAWYRMCKYSYRRATKTYTGYGGCYAAAMMYTATTRT 29700
Indonesia-88045      MCSCYCCRGTGTARGMRKMGRKGCAWYRMCKYSYRRRYKTYTGYGGCYAAAMMYTATTRT 29700
Indonesia-88065      MYSCYSCRGTGTARGMGKMGRKKCAWYRMCKYSYRRATKTYTGYGGCYAAAMMYWRTTRT 29700
                     * .  . *      .  .  **        ****    *   .*   *  .* *   .

Bhutan-09015         RRCMAGRYGRYRYMKRGATRGGKKAYAKKTCATARKRWRCTATTKKYKYARTMKSYYWYK 29760
Bhutan-09024         RRCMAGRYGRYRYMKRGATRGGKKAYAKKTCATARKRWRCYWTTKKYKKARTMKSYYWYK 29760
Bhutan-09027         RRCMAGRYGRYRYMKRGATRGGKKAYAKKTCATARKRWRCYWTTKKYKYARTMKSYYWYK 29760
Bhutan-09030         RRCMAGRYGRYRYMKRGATRGGKKAYAKKTCATARKRWRCYWTTKKYKKARTMKSYYWYK 29760
Bhutan-09005         GRSCWKATKAYRYMKRRRYRATKTRCMGTWYGGMRTGAGCTAGGTKYTGWGWAKSCYACG 29760
Indonesia-88035      GRSCWKATKAYRYMKRRRYRATKTRCMGTWYGGMRTGAGYYAGGTKYTGAGWAKSCYACG 29760
Indonesia-88045      GRSCWKATKAYRYMKRRRYRATKTRCMGTWYGGMRTGAGYYAGGTKYTGAGWAKSCYACG 29760
Indonesia-88065      GRSCWKATKAYRYMKRRRYRATKTRCMGTWYGGMRTGAGYYAGGTKYTGAGWAKSCYACG 29760
                      *.       ******   *. *.    .  .  *.        .**.     ** *

Bhutan-09015         MYRATCAMGWTWYSTAGRMAYRKTYSATRRMRTWYTYRYMARATYYRCAACTMRYYRTYY 29820
Bhutan-09024         MYRATCAMGWTWYSTAGRMAYRKTYSATRRMRTWYTYRYMARATYYRCAACTMRYYRTYY 29820
```

```
Bhutan-09027         MYRATCAMGWTWYSTAGRMAYRKTYSATRRMRTWYTYRYMARATYYRCAACTMRYYRTYY 29820
Bhutan-09030         MYRATCAMGWTWYSTAGRMAYRKTYSATRRMRTWYTYRYMARATYYRCAACTMRYYRTYY 29820
Bhutan-09005         MYAWYYMARWYACSYWKAMTCRTTYGGKAAMGWACYCGCCMAMYCTRYRMSWCRCYGACC 29820
Indonesia-88035      MYAWYYMARWYACSYWKAMWCRTKYGGKAAMRWATYCGCCMAMYCTRYRMSWCRCYGACC 29820
Indonesia-88045      MYAWYYMARWYACSYWKAMTCRTKYGGKAAMRWATYCGCCMAMYCTRYRMSWCRCYGACC 29820
Indonesia-88065      MYAWYYMARWYACSYWKAMWCRTTYGGKAAMGWACYCGCCMAMYCTRYRMSWCRCYGACC 29820
                     **       *   *    *  *..*...  *               *   .  * * :

Bhutan-09015         GSYCTGRGACGGGYYYSATCCYAKRKYYRMYTTWACWYTMMCCAAGTYYWRCGCWARTYK 29880
Bhutan-09024         GSYCTGRGACRRGYYYSATCCYAKRKYYRMYTTWACWYTMMCCAAGTYYWRCGCWARTYK 29880
Bhutan-09027         GSYCTGRGACRRGYYYSRTCCYAKRKYYRMYTTWACWYTMMCCAAGTYYWRCGCWARTYK 29880
Bhutan-09030         GSYCTGRGACRRGYYYSATCCYAKRKYYRMYTTWACWYTMMCCAAGTYYWRCGCWARTYK 29880
Bhutan-09005         KGYTWRASRCKGGYTCCRWSYTRTGTTTRCTYYAMCTCCMAYMWARKCTAGYSSWMRYCT 29880
Indonesia-88035      KGYTWRAGRYGGRYTCCRWSCTRTGTTTRCTYYAMATCCMAYAWWRKCTAGYSSWMRYCT 29880
Indonesia-88045      KGYTWRAGRYGGRYTCCATSCTRTGTTTRCTYYAMATCCMAYAWWAGCTAGYSSWMRYCT 29880
Indonesia-88065      KGYTWRASRCKGGYTCCRWSYTRTGTTTRCTYYAMCTCCMAYMWARKCTAGYSSWMRYCT 29880
                      .*    .     *  .  .   . .  *      .   *            ..* *  .

Bhutan-09015         WYRRWKSTWCAKTCGYYYCAWKWWRYWWCTYGRCRYGWMYRMTCAMCAYWATCCCTAACG 29940
Bhutan-09024         WYRRWKSTWCAKTCGYYYCAWKWWRYWWCTYGRCRYGWMYAATCAMCAYWATCCCTAACG 29940
Bhutan-09027         WYRRWKSTWCAKTCGYYYCAWKWWRYWWCTYGRCRYGWMYAATCAMCAYWATCCCTAACG 29940
Bhutan-09030         WYRRWKSTWCAKTCGYYYCAWKWWRYWWCTYGRCRYGWMYRMTCAMCAYWATCCCTAACG 29940
Bhutan-09005         WTAGTGSCWARGYARYYCYRAGWTATKWYYCRRCGTRTMYAMYYMMMMTTMYMMYAMRGR 29940
Indonesia-88035      WTAGTGGTTARKYAGYYCYRAKWTATKWYTCRRYGTRTMYAMYYCCMMTTMYCCYAMRGR 29940
Indonesia-88045      WTAGTGGTTARKYAGYYCYRAKWTATKWYTCRRYGTRTMYAMYYCCACTTMYCCYAMRGR 29940
Indonesia-88065      WTAGTGSCWARGYARYYCYRAGWTATKWYYCRRCGTRTMYAMYYMMMMTTMYMMYAMRGR 29940
                     *     .  .   . **     *    *    *     **               :

Bhutan-09015         YRYGTKAYARTGCMRACCTRTTCASWCWARAGGTKAAMMRRSGSGRKYYYTRARCRATSK 30000
Bhutan-09024         YRYKTKAYARTGCMRACCTRTTCASWCWARAGGTKAAMMRRSGSGRKYYYTRARCRATSK 30000
Bhutan-09027         YRYGTKAYARTGCMRACCTRTTCASWCWRRAGGTKAAMMRRSGSGRKYYYTRARCRATSK 30000
Bhutan-09030         YRYGTKAYARTGCMRACCTRTTCASWCWRRAGGTKAAMMRRSGSGRKYYYTRARCRATSK 30000
Bhutan-09005         CGCGGTWTRAKRGAGGTCGATYATSWYTRGRKKYTRRMCGACKGRGTCTCYRMAMRMWCG 30000
Indonesia-88035      CGCGGTWTRAGAGAGGTYGAYYATSTYTAGRKKCTRRMCGACKGRGTCTCYAMAMRMWCG 30000
Indonesia-88045      CGCGGTWTRAGAGAGGTYGAYYATSTYTRGRKKCTRRMCGACKGRGTCTCYAMAMRMWCG 30000
Indonesia-88065      CGCGGTWTRAKRGAGGTCGATYATSWYTRGRKKYTRRMCGACKGRGTCTCYRMAMRMWCG 30000
                          .         .      .:*         .  *   . .  .        *  .

Bhutan-09015         AWTMWWTKARRARYYCTYYRRWKTARRRAAWKYCCRKYWGSMSTRYRRACWWCASTARCG 30060
Bhutan-09024         AWTMWWTKARRARYYCTYYRRWKTARRRAAWKYCCRKYWGSMSTRYRRACWWCASTARCG 30060
Bhutan-09027         AWTMWWTKARRARYYCTYYRRWKTARRRAAWKYCCRKYWGSMSTRYRRACWWMRSTARCG 30060
Bhutan-09030         AWTMWWTKARRARYYCTYYRRWKTARRRAAWKYCCRKYWGSMSTRYRRACWWMRSTARCG 30060
Bhutan-09005         GACMATYTGAAWRTTYWTTRGAKTRARGRTWKYMYATCAGCAKGGCARWSATCASWRGYK 30060
Indonesia-88035      GACAATYTGAAWRTTYWTTRGAKKRARGRTWKYMYATCAACAKGGCARASATCASWRGYK 30060
Indonesia-88045      GACAATYTGAAWRTTCWTTRGAKKRARGRTWKYMYATCAACAKGGCARASATCASWRGYK 30060
Indonesia-88065      GACAATYTGAAWRTTCWTTRGAKKRARGRTWKYMYATCAGCAKGGCARASATCASWRGYK 30060
                     .      ..   *      *  *.  *  :***   .  .. .    *  .    *

Bhutan-09015         MYTWMSTATMARWTYARKWTTKMASWYYMTSGGRKKAGYYWTCRAGYRWAATTAASKRMT 30120
Bhutan-09024         MYTWMSTATMARWTYARKWTTKMASWYYMTSGGRKKAGYYWTCRAGYRWAATTAASKRMT 30120
Bhutan-09027         MYTWMSTATMARWTYARKWTTKMASWYYMTSGGRKKAGYYWTCRAGYRWAATTAASKRMT 30120
Bhutan-09030         MYTWMSTATMARWTYARKWTTKMASWYYMTSGGRKKAGYYWTCRAGYRWAATTAASKRMT 30120
Bhutan-09005         MYYWMSYRCCRAWKYRRTWKAKCMGTYCACGKCGKKTKYYWYSRGSTAWGMCYGGSGGRC 30120
Indonesia-88035      MYYWMSYRCCRAWKYRRTTKAKCMGTYCACGKCGKKTKYYWYSRGSTAWGMCYGGSGGRC 30120
```

```
Indonesia-88045      MYYWMSYRCCRAWKYRRTWKAKCMGTYCACGKCGKKTKYYWYSRGSTAWGMCYGGSGGRC 30120
Indonesia-88065      MYYWMSYRCCRAWKYRRTWKAKCMGTYCACGKCGKKTKYYWYSRGSTAWGMCYGGSGGRC 30120
                     ** ***      *.* *. .:*  . *   .   **: *** .*..  *.   ..*

Bhutan-09015         WTGMYYGMGYRAWKKAKGAAMYMGCSRGMMRGSRGYTRCKYASYYYARCYKYKAARYYCY 30180
Bhutan-09024         WTGMYYGMGYRAWKKAKGAAMYMGCSRGMMRGSRGYTRCKYASYYYARCYKYKAARYYCY 30180
Bhutan-09027         WTGMYYGMGYRAWKKAKGAAMYMGCSRGMMRGSRGYTRCKYASYYYARCYKYKAARYYCY 30180
Bhutan-09030         WTGMYYGMGYRAWKKAKGAAMYMGCSRGMMRGSRGYTRCKYASYYYARCYKTKAARYYCY 30180
Bhutan-09005         TASCCYKMRCGRATGRGCGCCTASGSRRWAARSGRTTGYKYGGCTYWRMWTTTWWGCCGY 30180
Indonesia-88035      TASCCYKMRCGRATGRGCGCCTASGSRRWAARSGRTYGYKYGGCTYWRCWTTTWWGCCGY 30180
Indonesia-88045      TASCCYKMRCGRATGRGCGCCTASGSRRWAARSGRTYGYKYGGCTYWRCWTTTWWGCCGY 30180
Indonesia-88065      TASCCYKMRCGRATGRGCGCCTASGSRRWAARSGRTTGYKYGGCTYWRMWTTTWWGCCGY 30180
                      :.  * *     .    ..   . **     *      **..  * * :. .      *

Bhutan-09015         RRCKAYARSRRGCSCAACRCSRAYYWARRAACAYRMKRYWYACRRCRTCCWCYMWMTWSS 30240
Bhutan-09024         RRCTAYARSRRGCSCAACRCSRAYYWARRAACAYRMKRYWYACRRCRTCCWCYMWMTWSS 30240
Bhutan-09027         RRCKAYARSRRGCSCAACRCSRAYYWARRAACAYRMKRYWYACRRCRTCCWCYMWMTWSS 30240
Bhutan-09030         RRCKAYARSRRGCSCAACRCSRAYYWARRAACAYRMKRYWYACRRCRTCCWCYMWMTWSS 30240
Bhutan-09005         RAMTTTRGCRRTMCYTGTGTCRCCTWRRRWMYRYRMKRTWCRYRRTGYYYWYCMWCWASG 30240
Indonesia-88035      RAMTTTRGCRRTMCYTGTGTCRCCTWRRRWMYRYRMKRTWCRYRRTGYYYWYCMWCWASG 30240
Indonesia-88045      RAMTTTRGCRRTMCYTGTGTCRCCTWRRRWMYRYRMKRTWCRYRRTGYYYWYCMWCWASG 30240
Indonesia-88065      RAMTTTRGCRRTMCYTGTGTCRCCTWRRRWMYRYRMKRTWCRYRRTGYYYWYCMWCWASG 30240
                     *  .:   .**  . :.   .*.  * **    ***** *   **     *  **   *.

Bhutan-09015         CGTGRRGWYCYCTMRRCWYACYYATTTGYGAYWTRASMYCYRWASACYWYRCRRTTTCYW 30300
Bhutan-09024         CGTGRRGWYCYCTMRRCWYACYYATTTGYGAYWTRASMYCYRWASACYWYRCRRTTTCYW 30300
Bhutan-09027         CGTGRRGWYCYCTMRRCWYACYYATTTGYGAYWTRASMYCYRWASACYWYRCRRTTTCYW 30300
Bhutan-09030         CGTGRRGWYCYCTMRRCWYACYYATTTGYGAYWTRASMYCYRWASACYWYRCRRTTTCYW 30300
Bhutan-09005         AAYRARSATACYKCAGYTYRTTCRCKYRCRMCAYARSAYATRWWSWMCAYAYAGWWKYYA 30300
Indonesia-88035      AACAAGSATACYKCAGYTYRTTCRCKYRCRMCAYARCAYATRWWSWMCAYAYAGWWKYYA 30300
Indonesia-88045      AACAAGSATACYTCAGYTYRTTCRCTTRCRMCAYARSAYATRWWSWMCAYAYAGWWKYYA 30300
Indonesia-88065      AAYRARSATACYKCAGYTYRTTCRCKYRCRMCAYARSAYATRWWSWMCAYAYAGWWKYYA 30300
                     ..    .  .  .     *      .          . *. ** *    *      . *

Bhutan-09015         KCYCTCCCTYMMRGRSRYGWTYGRWGAKYRYWYYCCGGRRSYRYASTCWTRSYAYYRYKG 30360
Bhutan-09024         KSYCTCCCTYMMRGRSRYGWTYGRWGAKYRYWYYCCGGRRSYRYASTCWTRCTAYYRYKG 30360
Bhutan-09027         KCYCTCCCTYMMRGRSRYGWTYGRWGAKYRYWYYCCGGRRSYRYASTCWTASTACYRYKG 30360
Bhutan-09030         KCYCTCCCTYMMRGRSRYGWTYGRWGAKYRYWYYCCGGRRSYRYASTCWTRCTACYRYKG 30360
Bhutan-09005         KCTYYYSTTYCMGRRGRCRWYTKGWKGTYAYWYCYYKKGRGYATRCKSWYRGCATCACKR 30360
Indonesia-88035      KCTYYYSTKCCMGRRGRCRWYTKGWKGTYRYWTCYYKKGRGYATRCKCTTAGCATCACGG 30360
Indonesia-88045      KCTYYYSTKYCMGRRGRCRWYTKGWKGTYRYWTCYYKKGRGYATRCKCTTAGCATCACGG 30360
Indonesia-88065      KCTYYYSTTCCMGRRGRCRWYTKGWKGTYAYWYCYYKKGRGYATRCKSWYRGCRTCACKR 30360
                     *.    . .  *  *.*  *    * ..* **       *.*   ...

Bhutan-09015         CSCCTYRTWWYYCGRRGAARSGTGGGKMRWYTYAYMYTRAACRGARGMRRGYYCTRYKAG 30420
Bhutan-09024         CSCCTYRTWWYYCGRRGAARSGTGGGKMRWYTYAYMYTRAACRGARGMRRGYYCTRYKAG 30420
Bhutan-09027         CSCCTYRTWWYYCGRRGAARSGTGGGKMRWYTYAYMYTRAACRGARGMRRGYYCTRYKAG 30420
Bhutan-09030         CSCCTYRTWWYYCGRRGAARSGTGGGKMRWYTYAYMYTRAACRGARGMRRGYYCTRYKAG 30420
Bhutan-09005         YSMYYTGYTWTTYRGRRRRRCRKRRRKMGWTYCWTMCKGGGYGRRRRCARSCTTWACGRK 30420
Indonesia-88035      CCCCTTGYTATTCGGAARRRCGTGGGTCGTTTCATCCTGGGCGGARRCAGSCTTTACGAG 30420
Indonesia-88045      CCCCTTGYTATTCGGAARRRCGTGGGTCGTTTCATCCTGGGCGGARRCAGSCTTTACGAG 30420
Indonesia-88065      YSMYYTGYTWTTYRGRRRRRCRKRRRKMGWTYCWTMCKGGGYGRRRRCARSCTTWACGRK 30420
                      .                 *. .   .          . ..    *    .
```

```
Bhutan-09015         CYCCTGAYTSAWYRYRTAAGGAAATCAATTWWKRGCGAGMKATGAACYRGGCGYRCKCGW 30480
Bhutan-09024         CYCCTGAYTSAWYRYRTAAGGAAATCAATTWWKRGCGAGMKATGAACYRGGCGYRCKCGW 30480
Bhutan-09027         CYCCTGAYTSAWYRYRTAAGGAAATCAATTWWKRGCGAGMKATGAACYRGGCGYRCKCGW 30480
Bhutan-09030         CYCCTGAYTSAWYRYRTAAGGAAATCAATTWWKRGCGAGMKATGAACYRGGCGYRCKCGW 30480
Bhutan-09005         MYYSCSRTKGWWYRYRYRMSGRCWWYWGYWATGARMAMSCGTWRCWAYAARYRYRYTCGT 30480
Indonesia-88035      CYCCCGRTTGWWYRYRTAAGGAMATYWGYWATGARMAMSCGTWRCTAYAARYRYRYTAAT 30480
Indonesia-88045      CYCCCGRTTGWWYRYRTAAGRAMATYWGYWATGARMAMSCGTWRCTMYAARYRYRYTAAT 30480
Indonesia-88065      MYYSCSRTKGWWYRYRYRMSGACWWYWGYWATGARMAMSCGTWRCWMYAARYRYRYTCGT 30480
                      * . .  .. *****   .       .        . .  :  .  * .   ** ...

Bhutan-09015         GRAYCCWRRRTWRGRGKCTCASKYTYYRYRSTMWRMCGCTWTARARWWGGYYRAMSYGAA 30540
Bhutan-09024         GRAYCCWRRRTWRGRGKCTCASKYTYYRYRSTMWRMCGCTWTARARWWGGYYRAMSYGAA 30540
Bhutan-09027         GRAYCCWRRRTWRGRGKCTCASKYTYYRYRSTMWRMCGCTWTARARWWGGYYRAMSYGAA 30540
Bhutan-09030         GRAYCCWRRRTWRGRGKCTCASKYTYYRYRSTMWRMCGCTWTARARWWGGYYRAMSYGAA 30540
Bhutan-09005         GGGTTATARGCTGSRSKGKYMGKCYTTRCACCCAACSCGCTCGGWGAAATTTGMMGTKGG 30540
Indonesia-88035      AGGTTATARGCTGSRSKGKYAGKCYTTGCACCCAACSCGCTCGGWGAAATTTGAMSYKGG 30540
Indonesia-88045      AGGTTATARGCTGSRSKGKYAGKCYTTRCACCCAACSCGCTCGGWGAAATTTGAMSYKGG 30540
Indonesia-88065      GGGTTATARGCTGSRSKGKYAGKCYTTRCACCCAACSCGCTCGGWGAAATTTGAMGTKGG 30540
                     . .  .  *    .*.* .  .*       .     .     .     .     *.  ..

Bhutan-09015         TYYRYRYCGWYTGAYRWGTTTARYARYTRRRMTTYARWCKRMTAAWRYRYACMTYAYGTR 30600
Bhutan-09024         TYYRYRYCGWYTGAYRWGTTTARYARYTRRRMTTYARWCKRMTAAWRYRYACMTYAYGTR 30600
Bhutan-09027         TYYRYRYCGWYTGAYRWGTTTARYARYTRRRMTTYARWCKRMYAAWRYRYACMTYAYGTR 30600
Bhutan-09030         TYYRYRYCGWYTGAYRWGTTTARYARYTRRRMTTYARWCKRMYAAWRYRYACMTYAYGTR 30600
Bhutan-09005         YTCGTGTARWYARTCATKKYYWRYWRTARRRMCYYRAWYGAMCRRWRCGTCGAATCTTCG 30600
Indonesia-88035      YTCGTGTARWYARTCATKKYYWGYWRTARRRMCYYRAWYGAMCGRWRCRYMGMWYMYKYR 30600
Indonesia-88045      YTCGTGTARWYARTCATKKYYWRYWRTARRRMCYYRAWYGAMCRRWRCRYMGMWYMYKYR 30600
Indonesia-88065      YTCGTGTARWYARTCATKKYYWRYWRTARRRMCYYRAWYGAMCRRWRCRYMGMWYMYKYR 30600
                            . **: :    .    * * :****  *  *   *   **

Bhutan-09015         YRTYTCCRTRGRYCSRCGAGCTSWGGTASYYSRCGKYTATAASTTWKGCARKWTATWWAY 30660
Bhutan-09024         YRTYTCCRTRGRYCSRCGAGCTSWGGTASYYSRYGKYTATAASTTWKGCARKWTATWWAY 30660
Bhutan-09027         YRTYTCCRTRGRYCSRCGAGCTSWGGTASYYSRYGKYTATAASTTWKGCARKWTATWWAY 30660
Bhutan-09030         YRTYTCCRTRGRYCSRCGAGCTSWGGTASYYSRYGKYTATAASTTWKGCARKWTATWWAY 30660
Bhutan-09005         TGCTYYYYRYRRRYAYGYARKYKCARCCRCYYGRYRGYYGCRGCYWTGKYRRGWKCYTWMT 30660
Indonesia-88035      YRYTTCYRYRRRYAYGYARKYKCARCCRCYYGRCRGYYGCRGCYWTGKYRRGWKCYTWMT 30660
Indonesia-88045      YRYTTCYRYRRRYAYGYARKYKCARCCRCYYGRYRGYYGCRGCYWTGKYRRGWKCYTWMT 30660
Indonesia-88065      YRYTYYYRYRRRYAYGYARKYKCARCCRCYYGRYRGYYGCRGCYWTGKYRRGWKCYTWMT 30660
                            * * **.   .   ..     .**.*   * .  ..       * *..  *

Bhutan-09015         WRRRYRRRARRGTRCRAWWSTTYRKCRYAMYASRYATAGGWYGRGGYYGYTKYCCGWRRS 30720
Bhutan-09024         WRRRYRRRARRGTRCRAWWSWTYRKCRYAMYASRYATAGGWYGRGGYYGYTKYCCGWRRS 30720
Bhutan-09027         WRRRYRRRARRGTRCRAWWSWTYRKCRYAMYASRYATAGGWYGRGGYYGYTKYCCGWRRS 30720
Bhutan-09030         WRRRYRRRARRGTRCRAWWSWTYRKCRYAMYASRYATAGGWYGRGGYYGYTKYCCGWRRS 30720
Bhutan-09005         WRRAYAARRAGKWGYRGWACTWYRGAAYRCCTGATRYMKSTTRAAATCTCYGCYSRARGG 30720
Indonesia-88035      WRRAYAARRAGKWGYRGWACTWYRGAAYRCCTGATACAKSTTRAAATCTCYGCYSRAAGG 30720
Indonesia-88045      WRRAYAARRAGKWGYRGWACTWYRGAATRCCTGATACAKSTTRAAATCTCYGCYSRAAGG 30720
Indonesia-88065      WRRAYAARRAGKWGYRGWACTWYRGAAYRCCTGATRYMKSTTRAAATCTCYGCYSRAAGG 30720
                     *** *  *       *.* .  ** .     :.      .    ..        .    .

Bhutan-09015         STGRSAWCCGYWCGTYRYGGTTAYGCRGTRATGCKYYYYACSMSAYTGWYCYSTRMRGKA 30780
Bhutan-09024         STGRSAWCCGYWCGTYRYGGTTAYGCRGKRAWGCKYYYYACSMSAYTGWYCYSTRMRGKA 30780
Bhutan-09027         STGRSAWCCGYWCGTYRYGGTTAYGCRGTRATGCKYYYYACSMSRYTGWYCYSTRMRGKA 30780
Bhutan-09030         STGRSAWCCGYWCGTYRYGGTTAYGCRGTRAWGCKYYYYACSMSAYTGWYCYSTRMRGKA 30780
```

```
Bhutan-09005        GWRAGGWTARYAMRKCGCRRAKWTRYRSTARTATTCCCTWTSCSAYWSWCMYMYGMRRTW 30780
Indonesia-88035     GWRAGGWTARTAMRKCGCRRAKWTRYRSKARAATTCCCTWTSCSAYWSWCMTAYGMRRTW 30780
Indonesia-88045     GWRAGGWTARTAMRKCGCRRAKWTRYRSKARAATTCCCTWTSCSAYWSWCMYMYGMRRTW 30780
Indonesia-88065     GWRAGGWTARYAMRKCGCRRAKWTRYRSKARAATTCCCTWTSCSAYWSWCMYMYGMRRTW 30780
                    .   ..* .     .     :.    *..   . .      * * * .*      ** .

Bhutan-09015        AYRYGTKYYWCYYMTSKYSRSGTYRKYMRRARRARRKRGMMYCCATCYCACYRRCYCTAR 30840
Bhutan-09024        AYRYGTKYYWCYYMTSKYSRSGTYRKYMRRARRARRKRGMMYCCATCYCACYRRCYCTAR 30840
Bhutan-09027        AYRTGTKYYWCYYMTSKYSRSGTYRKYMRRARRARRKRGMMYCCATCYCACYRRCYCTAR 30840
Bhutan-09030        AYRTGTKYYWCYYMTSKYSRSGTYRKYMRRARRARRKRGMMYCCATCYCACYRRCYCTAR 30840
Bhutan-09005        RYGTRYKTTTYYCRYSGCCASRYCGTTARARRAWGGKAKCCCMMRYTTTCGCGGYCSAGA 30840
Indonesia-88035     RYGYRYKTTTYYCRYSGCCASRYCGTTARARRAWGRKAKCCCMMATYTTACYGRYYCAAA 30840
Indonesia-88045     RYGYRYKTTTYYCRYSGCCASRYCGTTARARRAWGRKAKCCCMMATYTTACYGRYYCAAA 30840
Indonesia-88065     RYGYRYKTTTYYCRYSGCCASRYCGTTARARRAWGGKAKCCCMMRYTTTCGCGGYCCAGA 30840
                     *    *    *   *  . *    .  *  *    *            .      .:.

Bhutan-09015        CRWATRKTTAGTAGRGYWSASMCRGRAWSTAMGTWAYSAYACRYCKTCTCSMGGRTAWGK 30900
Bhutan-09024        CRWATRKTTAGTAGRGYWSASMCRGRAWSTRMGTWAYSAYACRYCKTCTCSMGGRTAWGK 30900
Bhutan-09027        CRWATRKTTAGTAGRGYWSASMCRGRAWCTRMGTWAYSAYACRYCKTCTCSMGGRTAWGK 30900
Bhutan-09030        CRWATRKTTAGTAGRGYWSASMCRGRAWGTAMGTWAYSAYACRYCKTCTCSMGGRTAWGK 30900
Bhutan-09005        MGTRKATKAWAGGRGACTGGCMYGRGRTCCAMRYWRYGRTTMAYSTKSKYGARRGCCARK 30900
Indonesia-88035     MGTRKATTTWGTGGRGCTGGCMYGRGRTCCAMRYWRYGRTTMAYSTKSKYGARRGCCARG 30900
Indonesia-88045     MGTRKATTTWGTGGRGCTGGCMYGRGRTCCAMRYTRYGRTTMAYSTKSKYGARRGCCARG 30900
Indonesia-88065     MGTRKATKAWAGGRGACTGGCMYGRGRTCCAMRYWRYGRTTMAYSTKSKYGARRGCCARK 30900
                        . ..: . .  .  ...*         *    *.  :  *..... .     .

Bhutan-09015        YYRWYRGARYYTTKCKMTYRYGTRRKMRGTGATARYCKGWYAATWTARGMTGKWKWCCYM 30960
Bhutan-09024        YYRWYRGARYYTTKCKMTYRYGTRRKMRGTGATARYCKGWYAATWTARGMTGKWKWCCYM 30960
Bhutan-09027        YYRWYRGARYYTTKCKMTYRYGTRRKMRGTGATARYCKGWYRRYWTARGMTGKWKWCCYM 30960
Bhutan-09030        YYRWYRGARYYTTKCKMTYRYGTRRKMRGTGATARYCKGWYRRYWTARGMYGKWKWCCYM 30960
Bhutan-09005        TCGTCGSRGCTCYTTGCCTGTCTRRKCAGKGWTMATYTGTYAATTCRRRCYAGTGWYYTC 30960
Indonesia-88035     TCGTYGSGRCYCYTTGCCTGTCYRRKCAAKRWYMATYTRTYAATTTRGGCYGGTGWYYTC 30960
Indonesia-88045     TCGTYGSGRCYCYTTGCCTGTCYRRKCAAKRWYMATYTRTYAATTTRGGCYGGTGWYYTC 30960
Indonesia-88065     TCGTCGSRGCTCYTTGCCTGTCTRRKCAGKGWTMATYTGTYAATTCRRRCYAGTGWYYTC 30960
                          .      .         ***  ..       .  *          .   *

Bhutan-09015        TARACTRYTRGACTAAAGARCYRCTRGYYCYRAAMATWYTRWYWYCAYMGSGTCCRRTCT 31020
Bhutan-09024        TARACTRYTRGACTAAAGARCYRCTRGYYCYRAAMATWYTRWYWYCAYMGSGTCCRRTCT 31020
Bhutan-09027        TARACTRYTRGACTAAAGARCYRCTRGYYCYRAAMATWYTRWYWYCAYMGSGTCCRRTCT 31020
Bhutan-09030        TARACTRYTRGACTAAAGARCYRCTRGYYCYRAAMATWYTRWYWYCAYMGSGTCCRRTCT 31020
Bhutan-09005        YRGWYYRYCRRRTGCCGCGGTYAYYRRCTTTGWRAWYWTYRWYWYSRTATSRCYYAACYY 31020
Indonesia-88035     TRGWYYRYCRRRTGCCGCGRCYAYYRRCTYTGARAWYWTYRWYWYSRTATSRYYYAACYY 31020
Indonesia-88045     TRGWYYRYCRRRTGCCGCGRCYAYYRRCTTTGARAWYWTYRWYWYSRTATSRYYYAACYY 31020
Indonesia-88065     YRGWYYRYCRRRTGCCGCGGTYAYYRRCTTTGWRAWYWTYRWYWYSRTATSRCYYAACYY 31020
                          ** *    ... .  *   *           *  *****.    *

Bhutan-09015        GAWYYCARAMTYRRMKYGRKSARWYAATGAGYGAATCAYWRWYYYYWKSATYRWMYCWAY 31080
Bhutan-09024        GAWYYCARAMTYRRMKYGRKSARWYAATGAGYGAATCAYWRWYYYYWKSATYRWMYCWAY 31080
Bhutan-09027        GAWYYCARAMTYRRMKYGRKSARWYAATGAGYGAATCAYWRWYYYYWKSATYRWMYCWAY 31080
Bhutan-09030        GAWYYCARAMTYRRMKYGRKSARWYAATGAGYGAATCAYWRWYYYYWKSATYRWMYCWAY 31080
Bhutan-09005        RRTCCMRGGMYCGACGTRAKGRAWTWRYRWRCRRWYYMCAGWTCTTATRRYYGWMCSARC 31080
Indonesia-88035     RRTCCMRGGMTCGACGTRRKGRRWYAAYRWRCRRWYYMCAGWTCTTATRRYYGWMCSARC 31080
Indonesia-88045     RRTCCMRGGMTCGACGTRRKGRRWYAAYRWRCRRWYYMCAGWTCTTATRRYYGWMCSARC 31080
Indonesia-88065     RRTCCMRGGMYCGACGTRAKGRAWTWRYRWRCRRWYYMCAGWTCTTATRRYYGWMCSARC 31080
```

```
                               .*         *.  *                 *     .   * ** .

Bhutan-09015           YYWAGYYKAYTCGRRWYAWWAWRYTRYRGYCMGYMWWWGTRRRCRCRKMYYASMYGTSCT 31140
Bhutan-09024           YYWAGYYKAYTCGRRWYAWWAWRYTRYRGYCMGYMWWWGKRRRCRCRKMYYASMYGTSCT 31140
Bhutan-09027           YYWAGYYKAYTCGRRWYAWWAWRYTRYRGYCMGYMWWWGGRRRCRCRKMYYASMYGTSCT 31140
Bhutan-09030           YYWAGYYKAYTCGRRWYAWWAWRYTRYRGYCMGYMWWWGTRRRCRCRKMYYASMYGTSCT 31140
Bhutan-09005           TTTWKCCGMTWYRRRATATARTGYWGYRRTYMSCAWWTSTARAGGTGTCTYTCMYRYSSW 31140
Indonesia-88035        TTTWKCCGMTWYRRRATRTARWRYTGYRRTYMSCAWWTGTARAGGYGTCTYTCMYRYSSW 31140
Indonesia-88045        TTTWKCCGMTWYRRRATRTARWRYTGYRRTYMSCAWWTGTARAGGYGTCTYTCMYRYSSW 31140
Indonesia-88065        TTTWKCCGMTWYRRRATATARTGYTGYRRTYMSCAWWTSTARAGGYGTCTYTCMYRYSSW 31140
                                    **        *  **   *.  ** .  *     .  *:.**  *.

Bhutan-09015           RAYAMCRYRRRKYMYATYYRYYGARAWWSMRWTAAGGWKSYTYTCTWRSGGSRRKRRWMR 31200
Bhutan-09024           RAYAMCRYRRRKYMYATYYRYYGARAWWSMRWTAAGGWKSYTYTCTWRSGGSRRKRRWMR 31200
Bhutan-09027           RAYAMCRYRRRKYMYATYYRYYGARAWWSMRWTAAGGWKSYTYTCTWRSGGSRRKRRWMR 31200
Bhutan-09030           RAYAMCRYRRRKYMYATYYRYYGARAWWSMRWTAAGGWKSYTYTCTWRSGGSRRKRRWMR 31200
Bhutan-09005           RRTTATACGGRTTACWKTYACTRWARWTKARWCRRRKARSTYCKYYAACRRSRRTGRTCG 31200
Indonesia-88035        RRTTATACGGRTYACWKTCACTGAAAWTKARWCAAGTARSTYCKYYARSGRSRRTGRTCG 31200
Indonesia-88045        RRTTATACGGRTYACWKTCACTGAAAWTKARWCRRRKARSTYCKYYARSGRSRRTGRTCG 31200
Indonesia-88065        RRTTATACGGRTTACWKTYACTRWARWTKARWCAAGTARSTYCKYYAACRRSRRTGRTCG 31200
                       *  :      *.    .         * . **      :*   .    .  ***. *

Bhutan-09015           KYAWRRMGCYSTGAACAKRRWMWKCCGGAATACGKTRGYSTRYKWGCTGRCAGCGRSMRM 31260
Bhutan-09024           KYAWRRMGCYSTGAACAKRRWMWKCCGGAATACGKTRGYSTRYKWGCTGRCAGCGRSMRM 31260
Bhutan-09027           KYAWRRMGCYSTGAACAKRRWMWKCCGGAATACGKTRGYSTRYKWGCTGRCAGCGRSMRM 31260
Bhutan-09030           KYAWRRMGCYSTGAACAKRRWMWKCCGGAATACGKTRGYSTRYKWGCTGRCAGCGRSMRM 31260
Bhutan-09005           GCMAAAMKMCKGRRRATTARWMWTCCTARRYRYAKWAKYGWATKAKCTRAGRGCGGCAAM 31260
Indonesia-88035        GCMAAAMKMCSTRRAATTARWMWTSYTAGGYRYAKWAKYGWATKAKYYRAGRKYTGCAAM 31260
Indonesia-88045        GCMAAAMKMCSTRRRATTARWMWTSYTAGGYRYAKWAKYGWATKAKYYRAGRKYTGCAAM 31260
Indonesia-88065        GCMAAAMKMCKGRRRATTARWMWTCCTARRYRYAKWAKYGWATKAKCTRAGRGCGGCAAM 31260
                             *   .    .:. ****..  .     .*   *.   *            .  *

Bhutan-09015           WWCTKMMRMTAARYCMRCTRRACAAWWGCKAKKGMRKTRSYCWCKGRYYGYSTKWWGACT 31320
Bhutan-09024           WWCTKMMRMTAARYCMRCTRRACAAWWGCKAKKGMRKTRSYCWCKGRYYGYSTKWWGACT 31320
Bhutan-09027           WWCTKMMRMTAARYYMRCTRRACAAWWGCKAKKGMRKTRSYCWCKGRYYGYSTKWWGACT 31320
Bhutan-09030           WWCTKMMRMTAARYYMRCTRRACAAWWGCKAKKGMRKTRSYCWCKGRYYGYSTKWWGACT 31320
Bhutan-09005           ATYYTMCAAKRMGYYAGYKRRRTRWWWASGRKGKMGGYRSYTAMKCRCYGYGYGATKRSY 31320
Indonesia-88035        ATYYTMCAAKRMGCYAGYKRGRTRWWWASGRKGKMGGYRSYTACKCRYYRTGYGATKACY 31320
Indonesia-88045        ATYYTMCAAKRMGYYAGYKRGRTRWWWASGRKGKMGGYRSYTACKCRYYRTGYGATKACY 31320
Indonesia-88065        ATYYTMCAAKRMGYYAGYKRGRTRWWWASGRKGKMGGYRSYTAMKCRCYGYGYGATKRSY 31320
                           .*   .        .*     **..  *  *   ***   * * *  .      .

Bhutan-09015           YGGRRRYRRACRSTKMRTRGGCCCTRGGRRCWWWARRRATCYWRKSARYRGARYRGRAAA 31380
Bhutan-09024           YGGRRRYRRACRSTKMRTRGGCCCTRGGRRCWWWARRRATCYWRKSARYRGARYRGRAAA 31380
Bhutan-09027           YGGRRRYRRACRSTKMRTRSSCCCTRGGRRCWWWARRRATCYWRKSAATRGARYRGRAAA 31380
Bhutan-09030           YGGRRRYRRACRSTKMRTRSSCCCTRGGRRCWWWARRRATCYWRKSAATRGARYRGRAAA 31380
Bhutan-09005           TRSRGRTRGRTGSAGMGKAGGYTYCASTAGYAAWGAAAMKYTARTSRRTRRGGCAGRMWC 31380
Indonesia-88035        TRSRGRTRGRTGSAGMGKAGGYTYCASTAGYAAWGAAAMKYTARTSRRTRRGGCARRMWC 31380
Indonesia-88045        TRSRGRTRGRTGSAGMGKAGGYTYCASTAGYAAWGAAAMKYTARTSRRTRRGGCARRMWC 31380
Indonesia-88065        TRSRGRTRGRTGSAGMGKAGGYTYCASTAGYAAWGAAAMKYTARTSRRTRRGGCAGRMWC 31380
                         .* * *    *: * . ..     .      *.    .   *.*   * .    *  .

Bhutan-09015           YWRASTYWGYKCYARGTGGAAYRATMWSKRMYMRSRCSKGCWAYSAGCRYCMKTCCYMRA 31440
Bhutan-09024           YWRASTYWGYKCYARGTGGAAYRATMWSKRMYMRSRCSKGCWAYSAGCRYCMKTCCYMRA 31440
```

```
Bhutan-09027            YWRASTYWGYKCYARGTGGAAYRATMWSKRMYMRSRCSKGCWAYSAGCRYCMKTCCYMRA 31440
Bhutan-09030            YWRASTYWGYKCYARGTGGAAYRATMWSKRMYMRSRCSKGCWAYSAGCRYCMKTCCYMRA 31440
Bhutan-09005            TTRRCYCWKYKTTARCCRRACYRTCCTSGRCTCGCGMSKKSAWYSRRSGYSCGWSATMRM 31440
Indonesia-88035         TTRRCYCWKYKTTRRCCRRACYRTCCTSGRCTCGCGMSKKSAWYSRRSGYSCGWSATMRA 31440
Indonesia-88045         TTRRCYCWKYKTTRRCCRRACYRTCCTSGRCTCGCGMSKKSAWYSRRSGYSCGWSATMRA 31440
Indonesia-88065         TTRRCYCWKYKTTARCCRRRCYRTCCTSGRCTCGCGMSKKSAWYSRRSGYSCGWSATMRA 31440
                          * .  * **   *     .**:   * *    .  ** .  **  . *.   .. **

Bhutan-09015            KRSWAGTTAAAYRCTSAYMAKCACWKKRTRRGKTYTYRCTRYTAGCYATCATYRACARGW 31500
Bhutan-09024            KRSWAGTTAAAYRCTSAYMAKCACWKKRTRRGKTYTYRCTRYTAGCYATCATYRACARGW 31500
Bhutan-09027            KRSWAGTTAAAYRCTSAYMAKCACWKKRTRRGKTYTYRCTRYTAGCYATCATYRACARGW 31500
Bhutan-09030            KRSWAGTTAAAYRCTSAYMAKCACWKKRTRRGKTYTYRCTRYTAGCYATCATYRACARGW 31500
Bhutan-09005            TAGWRGCWWRCCGYASRCMWGTGTATTAYAATTYTYTAYYRTKRKYCWYSCCCGRSAGKA 31500
Indonesia-88035         TAGWRGCWWRCCGYASRCMWGTGTATTAYAATTYYYTRCTRTKRGCCWTCCCCGRSRGKA 31500
Indonesia-88045         TAGWRRCWWRCCGYASRCMWGTGTATTAYAATTYYYTRCTRTKRGCCWTCCCCGRSRGKA 31500
Indonesia-88065         TAGWRRCWWRCCGYASRCMWGTGTATTAYAATTYTYTAYYRTKRKYCWYSCCCGRSAGKA 31500
                        . .*      .   :*  *   .  ..     .       * .      ..    .

Bhutan-09015            MCMYYTKAMYAYYKGYACWCAYYRGYAWKTAGTRRYYGRSWYYTKRAYAWYSYCARYSTM 31560
Bhutan-09024            MCMYYTKAMYAYYKGYACWCAYYRGYAWKTAGTRRYYGRSWYYTKRAYAWYSYCARYSTM 31560
Bhutan-09027            MCMYYTKAMYAYYKGYACWCAYYRGYAWKTAGTRRYYGRSWYYTKRAYAWYSYCARYSTM 31560
Bhutan-09030            MCMYYTKAMYAYYKGYACWCAYYRGYAWKTAGTRRYYGRSWYYTKRAYAWYSYCARYSTM 31560
Bhutan-09005            AYACCTTWCCWCYGRYTYTTMTCARYRWTKASKRGTTKGSATYCKRMYGTTCYTGRCGWM 31560
Indonesia-88035         AYACCKTWCCWCYGRCTYTTMTCARYRWTKRSKAGTTKGSATYCKRMYGTTCYTGRCGWM 31560
Indonesia-88045         AYACCKTWCCWCYGRYTYTTMTCARYRWTKRSKAGTTKGSATYCKRMYGTTCYTGRCGWM 31560
Indonesia-88065         AYACCTTWCCWCYGRYTYTTMTCARYRWTKASKAGTTKGSATYCKRMYGTTCYTGRCGWM 31560
                             ..     *   :        * *.. ..      *  * ** *.  .* .* . *

Bhutan-09015            YCGTCRTGYWCRCRCRKTYYRCWTACWGYAYTWKTGMYRYRWSKYRRCMATCCWGACTWR 31620
Bhutan-09024            YCGTCRTGYWCRCRCRKTYYRCWTACWGYAYTWKTGMYRYRWSKYRRCMATCCWGACTWR 31620
Bhutan-09027            YCGTCRTGYWCRCRCRKTYYRCWTACWGYAYTWKTGMYRYRWSKYRRCMATCCWGACTWR 31620
Bhutan-09030            YCGTCRTGYWCRCRCRKTYYRCWTACWGYAYTWKTGMYRYRWSKYRRCMATCCWGACTWR 31620
Bhutan-09005            YYGWYATGCWMAGRCGKYTTGSTTRYWRYRCYTGYKCYGCRTCKYRRCMAYGYTRRMKTR 31620
Indonesia-88035         YYRWYAYRCWMAGAMGTYTTGGTCRYWRTRCYAGYKCYGCATCKYRRMMRYGYTRRMKTR 31620
Indonesia-88045         YYRWYAYRCWMAGAMGTYTTGSTCRYWRTRCYAGYKCYGCATCKYRRMMRCGYTRRMKTR 31620
Indonesia-88065         YYGWYATGCWMAGRCGKYTTGSTTRYWRYRCYTGYKCYGCRTCKYRRCMAYGYTRRMKTR 31620
                        *        *      .         *          *    .**** *        . *

Bhutan-09015            TWYWKYMYYRYAARAWWYYRRTTKMRRAYSTTKRSWGYRYRKGCYKRTYSGRTTGTRYRR 31680
Bhutan-09024            TWYWKYMYYRYAARAWWYYRRTTKMRRAYSTTKRSWGYRYRKGCYKRTYSGRTTGTRYRR 31680
Bhutan-09027            TWYWKYMYYRYAARAWWYYRRTTKMRRAYSTTKRSWGYRYGKGCYKRTYSGRTTGTRYRR 31680
Bhutan-09030            TWYWKYMYYRYAARAWWYYRRTTKMRRAYSTTKRSWGYRYRKGCYKRTYSGRTTGTRYRR 31680
Bhutan-09005            WWCAKTCTYRYRRGGATTTGAWYTAGAMTGYWGGGTSYGCAKRSTGAYMSTGATTYRCGA 31680
Indonesia-88035         WWCAKTCTYRYGRGGATTTGAWYTAGAMTGYWGGGTSYGTAKRSTGRYMSGGATTYRYGA 31680
Indonesia-88045         WWCAKTCTYRYRRGGATTTGAWYTAGAMTGYWGGGTSYGTAKRSTGRYMSGGAWTYRYGA 31680
Indonesia-88065         WWCAKTCTYRYRRGGATTTGAWYTAGAMTGYWGGGTSYGCAKRSTGAYMSTGATTYRCGA 31680
                         *  *   ***   .        .     .    .. *   * .     *  :   *

Bhutan-09015            CCWKRRYWSTRKGRMAAAATRMMYKTMKSRASYRGAYGYATYTGATTYAGTTAWKWRWRA 31740
Bhutan-09024            CCWKRRYWSTRKGRMAAAATRMMYKTMKSRASYRGAYGYATYTGATTYAGTTAWKWRWRA 31740
Bhutan-09027            CCWKRRYWSTRKGRMAAAATRMMYKTMKSRASYRGAYGYATYTGATTYAGTTAWKWRWRA 31740
Bhutan-09030            CCWKRRYWSTRKGRMAAAATRMMYKTMKSRASYRGAYGYATYTGATTYAGTTAWKWRWRA 31740
Bhutan-09005            ACTKRRYTGKRGAACWMCAYAMATTYMTGRWCYRSGYAYWGCKKRCCTGCCKRWTTGAGR 31740
Indonesia-88035         MTTKRRYWSKRGGACWMCRYAMMTTYMKGRWCYRSGYAYWGCKKRCCTGCCKGWTTGAGR 31740
```

```
Indonesia-88045     MTTKRRYWSKRGGACWMCAYAMMTTYMKGRWCYRSGYAYWGCKKRCCTGCCKGWTTGAGR 31740
Indonesia-88065     ACTKRRYTGKRGAACWMCAYAMATTYMTGRWCYRSGYAYWGCKKRCCTGCCKRWTTGAGR 31740
                       **** ..* .    .   *  . *..* .**..*.*   .     .  . *.

Bhutan-09015        RMGKCARMWKYYWGWGWCYTTYGRRGTYCATAWWTKMAKRKSARAYCTAWGWYWCTKRCA 31800
Bhutan-09024        RMGKCARMWKYYWGWGWCYKKYGRRGTYCATAWWTKMAKRKSARAYCTAWGWYWCTKRCA 31800
Bhutan-09027        RMGKCARMWKYYWGWGWCYTTYGRRGTYCATAWWTKMAKRKSARAYCTAWGWYWCTKRCA 31800
Bhutan-09030        RMGKCARMWKYYWGWGWCYTTYGRRGTYCATAWWTKMAKRKSARAYCTAWGWYWCTKRCA 31800
Bhutan-09005        GCGKCMAMTGYCWSTGTMCTTCTAGSWCYAKCATYGMWKRKGRAWCGWWAKTYWYKGRSR 31800
Indonesia-88035     GCRKMAAMTGYCWSTRTMCKKCTAGSWCYGKCATYGMWKRKSRAWCCWWAKTYWYKGRCR 31800
Indonesia-88045     GCRKMAAMTGYCWSTRTMCKKCTAGSWCYGKCATYGMWKRKSRAWCCWWAKTYWYKGRCR 31800
Indonesia-88065     GCGKCMAMTGYCWSTGTMCKKCTAGSWCYAKCATYGMWKRKGRAWCGWWAKTYWYKGRSR 31800
                       *   *  * *.     ..    .   ...    * ***.          ** . *.

Bhutan-09015        MAMCTCYARWKKCSCACRWYMRRRTMGGRTYTWYYTKCCYATAYRRYYGCKMKKGMTSSY 31860
Bhutan-09024        MAMCTCYARWKKCSCACRWTMRRRTMGGRTYTWYYTKCCYATAYRRYYGCKMKGGMTSSY 31860
Bhutan-09027        MAMCTCYARWKKCSCACRWTMRRRTMGGRTYTWYYTKCCYATAYRRYYGCKMKKGMTSSY 31860
Bhutan-09030        MAMCTCYARWKKCSCACRWTMRRRTMGGRTYTWYYTKCCYATAYRRYYGCKMKKGMTSSY 31860
Bhutan-09005        MGMYWYYMRWKGTSYMMGTTCGAAYCSRAGYWACYYKYSYWYRTAATYATTATTKACCCY 31860
Indonesia-88035     MGMYWYYMRWKGTSYMMGTTCGAAYCSRAGYWACYYKYSYWYRTAATYATTATTKACCCY 31860
Indonesia-88045     MGMYWYYMRWKGTSYMMGTTCGAAYCSRAGYWACYYKYSYWYRTAATYATTATTKACCCY 31860
Indonesia-88065     MGMYWYYMRWKGTSYMMGTTCGAAYCSRAGYWACYYKYSYWYRTAATYATTATTKACCCY 31860
                    *.*   * ***  *            .   *   * * .*       *. . .    ..*

Bhutan-09015        TRAMYRAWGGYYSRCSAAYYRKYYMGMKKRKRCCWSARRRCYTTATMRARYMMGYWSAYA 31920
Bhutan-09024        TRAMYRAWGGYYSRCSAAYYRKYYMGMKKRKRCCWSARRRCYTTATMRARYMMGCASAYA 31920
Bhutan-09027        TRAMYRAWGGYYSRCSAAYYRKYYMGMKKRKRCCWSARRRCYTTATMRARYMMGYWSAYA 31920
Bhutan-09030        TRAMYRAWGGYYSRCSAAYYRKYYMGMKKRKRCCWSARRRCYTTATMRARYMMGYWSAYA 31920
Bhutan-09005        YRRMYRTWSACCCAYSRRYYRGCYMKMGKGGGTYTSWGARYYAWWYMGRGYCCKTTCRYR 31920
Indonesia-88035     YRRMYRTWSACCCAYSRRYYRGCYMKMGKGGGTYTSWGARYYAWWYMGRGYCCKTTCRYR 31920
Indonesia-88045     YRRMYRTWSACCCAYSRRYYRGCYMKMGKGGGTYTSWGARYYAWWYMGRGYCCKTTCRYR 31920
Indonesia-88065     YRRMYRTWSACCCAYSRRYYRGCYMKMGKGGGTYTSWGARYYAWWYMGRGYCCKTTCRYR 31920
                     * ***:*..  .  *  ***  ** * *      *   * *:   *   *     . *

Bhutan-09015        CRCCMRGYYKTKSTGKTYWARYCRAKTSGKGRTRYRYRAGYYATYYKGCMSTRWGTWWGK 31980
Bhutan-09024        CRCCMRGYYKTKSTGKTYWARYCRAKTSGKGRTRYRYRAGYYATYYKGCMSTRWGTWWGK 31980
Bhutan-09027        CRCCMRGYYKTKSTGKTYWARYCRAKTSGKGRTRYRYRAGYYATYYKGCMSTRWGTWWGK 31980
Bhutan-09030        CRCCMRGYYKTKSTGKTYWARYCRAKTSGKGRTRYRYRAGYYATYYKGCMSTRWGTWWGK 31980
Bhutan-09005        SRSYAGRCTTYKCWRGCYWRKYTARGWCSGARYRYACRCGCCMCTCGRYCCKRARYAWKK 31980
Indonesia-88035     SRSYAGRCTTYKCWRGCYWRKYTARGWCSGAGYRYACRCTYCMCTCGRYCCKRARYAWKK 31980
Indonesia-88045     SRSYAGRCTTYKCWRGCYWRKYTARGWCSGAGYRYACRCTYCMCTCGRYCCKRARYAWKK 31980
Indonesia-88065     SRSYAGRCTTYKCWRGCYWRKYTARGWCSGARYRYACRCGYCMCTCGRYCCKRARYAWKK 31980
                    .*.      . *.    ** :*     .. .  **  *.           ..*    * *

Bhutan-09015        GSRCCYSMWMCAGRGKYTYKWTTCAARAATKYTARTWARRWSKRRWAAGGRYYSRYTWYY 32040
Bhutan-09024        GSRCCYSMWMCAGRGKYTYKWTTCAARAATKYTARTWARRWSKRRWAAGGRYYSRYTWYY 32040
Bhutan-09027        GSRCCYSMWMCAGRGKYTYKWTTCAARAATKYTARTWARRWSKRRWAAGGRYYSRYTWYY 32040
Bhutan-09030        GSRCCYSMWMCAGRGKYTYKWTTCAARAATKYTARTWARRWSKRRWAAGGRYYSRYTWYY 32040
Bhutan-09005        RSATTTSMTMYMSGKKCKTTWYKTGWGRMYTYYRRWWGGRWGTRGTRRKRGYTSGYGATC 32040
Indonesia-88035     RSATTTSMTMYMSGKKCKTTWYKTGWGRMYTYYRRWWGGRWGTRGTRRKRGYTSGYGATC 32040
Indonesia-88045     RSATTTSMTMYMSGKKCKTTWYKTGWGRMYTYYRRWWGGRWGTRGTRRKRGYTSGYGATC 32040
Indonesia-88065     RSATTTSMTMYMSGKKCKTTWYKTGWGRMYTYYRRWWGGRWGTRGTRRKRGYTSGYGATC 32040
                     *    ** *  .  * . .* . .     .*  * *. **..*       * * *
```

```
Bhutan-09015        YRARCRWYAAYYASWRCMGYAMGRTARCGAAKARTTRYGYSGTRGMTYACGTTAGMRCAT 32100
Bhutan-09024        YRARCRWYAAYYASWRCMGYAMGRTARCGAAKARTTRYGYSGTRGMTYACGTTAGMRCAT 32100
Bhutan-09027        YRARCRWYAAYYASWRCMGYAMGRTARCGAAKARTTRYGYSGTRGMTYACGTTAGMRCAT 32100
Bhutan-09030        YRARCRWYAAYYASWRCMGYAMGRTARCGAAKARYYRYGYSGTRGMTYACGTTAGMRCAT 32100
Bhutan-09005        TRAGYGTYRRYTMCAAACRTRMARYGATSCRGTATCGYSYGRYAAAKYRSTAGWRMRYMY 32100
Indonesia-88035     TRTGYGTYRRYTMCAAACRTRMARYGATSCGGTAYCGYSYGRYAAAKYRSTAGWRMRYMY 32100
Indonesia-88045     TRTGYGTYRRYTMCAAACRTRMARYGATSCRGTATCGYSYGRYAAAKYRSTAGWRMRYMY 32100
Indonesia-88065     TRAGYGTYRRYTMCAAACRTRMARYGATSCRGTAYCGYSYGRYAAAKYRSTAGWRMRYMY 32100
                     *:    *  *  .  .    *.* .  ..  :    *.*.   . .* . :   **

Bhutan-09015        CTMCKKRRKYWTCGWKRATYRGWRWARYRATCYSAGTTMCRRRRAKSSRGCTSGRKAAAT 32160
Bhutan-09024        CTMCKKRRKYWTCGWKAATYRGWRWARYRATCYSAGTTMCRRRRAKSSRGCTSGRKAAAT 32160
Bhutan-09027        CTMCKKRRKYWTCGWKAATYRGWRWARYRATCYSAGTTMCRRRRAKSSRGCTSGRKAAAT 32160
Bhutan-09030        CTMCKKRRKYWTCGWKAATYRGWRWARYRATCYSAGTTMCRRRRAKSSRGCTSGRKAAAT 32160
Bhutan-09005        YGCTKGAGTCAYAAWTARKTGKWGWWRYRGKYTSGSYYMYRGGRCKGGARYWSRAYRRRW 32160
Indonesia-88035     YGCTKGAGTCAYAAWTARKTGKWGWWRYRGKYTSGSYYMYRGGRCGGGARYWSRAYRRRW 32160
Indonesia-88045     YGCTKGAGTCAYAAWTARKTGKWGWWRYRGKYTSGSYYMYRGGRCGGGARYWSRAYRRRW 32160
Indonesia-88065     YGCTKGAGTCAYAAWTARKTGKWGWWRYRGKYTSGSYYMYRGGRCKGGARYWSRAYRRRW 32160
                        *   .   ..*.  .   * * ***..  *..  * *  *. ..    *

Bhutan-09015        RRWRMATCARTTCGCMRYYWKYRRCCYWATACKYTGYSTATTWRRCSWMGMTKTTTRTCC 32220
Bhutan-09024        RRWRMATCARTTCGCMRYYWKYRRCCYWATACKYTGYSTATTWRRCSWMGMTKTTTRTCC 32220
Bhutan-09027        RRWRMATCARTTCGCMRYYWKYRRCCYWATACKYTGYSTATTWRRCSWMGMTKTTTRTCC 32220
Bhutan-09030        RRWRMATCARTTCGCMRYYWKYRRCCYWATACKYTGYSTATTWRRCSWMGMTKTTTRTCC 32220
Bhutan-09005        AAAGMWAYGRWYYRYCRTYTGTGGATCWRCWYGTGSCSKCWYTRAYSAATMGKYAGAGYY 32220
Indonesia-88035     AAAGMWAYGRWYYRYCRTYTGTGGATCWRCWYGTGSCSKCWYTRAYSAATMGKYTGAGYY 32220
Indonesia-88045     AAAGMWAYGRWYYRYCRTYTGTGGATCWRCWYGTGSCSKCWYTRAYSAATMGKYTGAGYY 32220
Indonesia-88065     AAAGMWACGRWYYRYCRTYTGTGGMTCWRCWYGTGSCSKCWYTRAYSAATMGKYAGAGYY 32220
                        * : .*      * *        *       . *..   *  *   * * :

Bhutan-09015        KYRCSAAWWYWTKYAMYKRSSTGYGMKRATRKWACRSYCKCRTYCARCRYCTKRCCTARK 32280
Bhutan-09024        KYRCGAAWWYWTKYAMYKRSSTGYGMKRATRKWACRSYCKCRTYCARCRYCTKRCCTARK 32280
Bhutan-09027        KYRCSAAWWYWTKYAMYKRSSTGYGMKRATRKWACRSYCKCRTYCARCRYCTKRCCTARK 32280
Bhutan-09030        KYRCSAAWWYWTKYAMYKRSSTGYGMKRATRKWACRSYCKCRTYCARCRYCTKRCCTARK 32280
Bhutan-09005        KYKMSRRTATAYGTMCYKRGCYRYRCTRRKGGWRMWGTMTSAWCYMASGCTTGRCYTRGG 32280
Indonesia-88035     KYKMSRRTATAYGTMCYKRGCYRYRCTRRKGGWRMWGTCTSRTCYCASGCTWGRYCYRGG 32280
Indonesia-88045     KYKMSRRTATAYGTMCYKRGCYRYRCTRRKGGWRMWGTCTSRTCYCASGCTWGRYCYRGG 32280
Indonesia-88065     KYKMSRRTATAYGTMCYKRGCYGYRCTRRKGGWRMWGTMTSAWCYMASGCTTGRCYTRGG 32280
                    **: .           ***..  *  .* .  *   .  ..      .     *

Bhutan-09015        TKCRSCRRATGARWRMATTGTKCRKKYTYCRTCSGAWTAAMRGCCRMTYRRWCRYCSTGS 32340
Bhutan-09024        TKCRSCRRATGARWRMATTGTKCRKKYTYCRTCSGAWTAAMRGCCRMTYRRWCRYCSTGS 32340
Bhutan-09027        TKCRSCRRATGARWRMATTGTKCRKKYTYCRTCSGAWTAAMRGCCRMTYRRWCRYCSTGS 32340
Bhutan-09030        TKCRSCRRATGARWRMATTGTKCRKKYTYCRTCSGAWTAAMRGCCRMTYRRWCRYCSTGS 32340
Bhutan-09005        WGTRSSRGGGSRAWAARYTKTKYGTGCWTMAKYGRRAACRMARYYRCYYRGWYRTYSYRG 32340
Indonesia-88035     WGTRSSRGGGSRAWAARYTTYKYGTGCWTMAKYGRRAACRMARYYRCYYRGWYRTYSYRG 32340
Indonesia-88045     WGTRSSRGGGSRAWAARYKTYKYGTGCWTMAKYGRRAACRMARYYRCYYRGWYRTYSYRG 32340
Indonesia-88065     WGTRSSRGGGSRAWAARYTKTKYGTGCWTMAKYGRRAACRMARYYRCYYRGWYRTYSTGG 32340
                       **.* . .  *    .  *  .      . .   :. *    *  ** * *  *  .

Bhutan-09015        YMSARYYKWWTYYRARWYMYCYCAMYSRYTCCKRSYATTATCAYGAACTYRRGYKTKAMR 32400
Bhutan-09024        YMSARYYKWWTYYRARWYMYCYCAMYSRYTCCKRSYATTATCAYGAACTYRRGYKTKAMR 32400
Bhutan-09027        YMSARYYKWWTYYRARWYMYCYCAMYSRYTCCKRSYATTATCAYGAACTYRRGYKTKAMR 32400
Bhutan-09030        YMSARYYKWWTYYRARWYMYCYCAMYSRYTCCKRSYATTATCAYGAACTYRRGYKTKAMR 32400
```

```
Bhutan-09005          TCCMGCTGAWKTTGRRTTATSYSRMYGRYGTATAGCCYTWCYAYGWWSYTGRRCGKKRMG 32400
Indonesia-88035       TCCMGCTGAWKTTGRRTTATSYSRMYGRYGTATAGCCYYACCAYAWWSYTGRGYGKKRMG 32400
Indonesia-88045       TCCMGCTGAWKTTGRRTTATSYSRMYGRYGTATAGCCYYACCWYWWWSYTGRGYGKKRMG 32400
Indonesia-88065       TCCMGCTGAWKTTGRRTTATSYSRMYGRYGTATAGCCYTWCYWYGWWSYTGRRCGTGRMG 32400
                        .      *.    *    .*. **.**  .. . .      *   .   *   .  *

Bhutan-09015          AYYAATTKGWMAGCYSYRCYRGTCYKGYRAKYKARYRGARCCTRCYYMCTGSYGATRYTC 32460
Bhutan-09024          AYYAATTKGWMAGCYSYRCYRGTCYKGYRAKYKARYRGARCCTRCYYMCTGSYGRKRYTC 32460
Bhutan-09027          AYYAATTKGWMAGCYSYRCYRGTCYKGYRAKYKARYRGARCCTRCYYMCTGSYGRKRYTC 32460
Bhutan-09030          AYYAATTKGWMAGCYSYRCYRGTCYKGYRAKYKARYRGARCCTRCYYMCTGSYGATRYTC 32460
Bhutan-09005          RTTTRAYKRWAGSYCCTGMCRKYYTTCCGTKCTRRTAARGTSWATYCATACGCKATGCYM 32460
Indonesia-88035       ATTTGWYKRWMGSYCCTGMCRKYCYKCCGTKCTRRTAARGTSWATYCATACGCKRKGCYM 32460
Indonesia-88045       RTTTGWYKRWMGSYCCTGMCRKYCYKCCGTKCTRRTAARGTSWATYCATACGCKRKGCYM 32460
Indonesia-88065       RTTTRAYKRWAGSYCCTGMCRKYCTTCCGTKCTRRTAARGTSWATYCATACGCKRKGCYM 32460
                         :   * * ..  .    *    .   :* . *  .   .   *   : .   .

Bhutan-09015          SYYYARYCCSGRMGTKAAARMRTYACTGWRSWSYCRCAGTYRWACRYYSRYWYTWKCGAY 32520
Bhutan-09024          SYYYARYCCCGRMGTKAAARMRTYACTGWRSWSYCRCAGTYRWACRYYSRYWYTWKCGAY 32520
Bhutan-09027          SYYYARYCCSGRMGTKAAARMRTYACTGWRSWSYCRCAGTYRWACRYYSRYWYTWKCGAY 32520
Bhutan-09030          SYYYARYCCSGRMGTKAAARMRTYACTGWRSWSYCRCAGTYRWACRYYSRYWYTWKCGAY 32520
Bhutan-09005          SYYCGACMCSCRATKKRRWAAGYYRMWATGSASYYGMRRCTAARSGTYGGTACWTGYRRY 32520
Indonesia-88035       SYYCGACMYSCRATGKRRWAAGYYRMWATGSWSYYGMRRCTAAGSGTYGGTACWTGCGRY 32520
Indonesia-88045       SYYCGACMYSCRATGKRRWAAGYYRMWATGSWSYYGMRRCTAAGSGTYGGTACWTGCGRY 32520
Indonesia-88065       SYYCGACMYSCRATKKRRWAAGYYRMWATGSASYYGMRRCTAARSGTYGGTACWTGYRRY 32520
                      *** .    . *   *       *   .  * **          .  *.          *

Bhutan-09015          RRYYCYYAKYCGATATYYGTRTMYATAATGYYYYRYYTAYRAAYRRTKAGGTTCRCTWGW 32580
Bhutan-09024          RRYYCYYAKYCGATATYYGTRTMYATAATGYYYYRYYTAYRAAYRRTKAGGTTCRCTWGW 32580
Bhutan-09027          RRYYCYYAKYCGATATYYGTRTMYATAATGYYYYRYYTAYRAAYRRTKAGGTTCRCTWGW 32580
Bhutan-09030          RRYYCYYAKYCGATATYYGTRTMYATAATGYYYYRYYTAYRAAYRRTKAGGTTCRCTWGW 32580
Bhutan-09005          GGTCYCYRGCYRRWRYCYGYGWAYRYRWKKYTCTGTCKWTARRYGAYKTASKTMASYWRA 32580
Indonesia-88035       GGTCYCYRGCCGAWRYCTRTGTATATAATGTTCTGTCKWTARRYGAYKTASKWMASYWRA 32580
Indonesia-88045       GGTCYCYRGCCGAWRYCTRTGTATATAATGTTCTGTCKWTARRYGAYKTASKWMASYWRA 32580
Indonesia-88065       GGTCYCYRGCYRRWRYCYGYGWAYRYRWKKYTCTGTCKWTARRYGAYKTASKTMASYWRA 32580
                            *                     .        .     *   *:...   . *

Bhutan-09015          TCKKRGTCSYYSAYTGTARAGRWWWRYYTGTCMARTRGYRGMATRGGTCATRWRWGRAYK 32640
Bhutan-09024          TCKKRGTCSYYSAYTGTARAGRWWWRYYTGTCMARTRGYRGMATRGGTCATRWRWGRAYK 32640
Bhutan-09027          TCKGAGTCSYYSAYTGTARAGRWWWRYYTGTCMARTRGYRGMATRGGTCATRWRWGRAYK 32640
Bhutan-09030          TCKKRGTCSYYSAYTGTARAGRWWWRYYTGTCMARTRGYRGMATRGGTCATRWRWGRAYK 32640
Bhutan-09005          TSGGAACYSYYYSWCYRWWAMARTATATYWSYYCWGWRKTGGCRSATAKTTCRAATRRWTT 32640
Indonesia-88035       YSGGAACTSYYYSWCYGWWAMARTATATYWSYYCTGTRKTGACRCATAKTTCRAATRRWTT 32640
Indonesia-88045       YSGGAACTSYYYSWCYGWWAMARTATATYWSYYCTGTRKTGACRSATAKTTCRAATRRWTT 32640
Indonesia-88065       TSGGAACYSYYYSWCYRWWAMARTATATYWSYYCWGWRKTGGCRSATAKTTCRAATRRWTT 32640
                       .   .  ****        .*     * .      *   .     .. : *    *  .

Bhutan-09015          TCRWRYSRYGTTWKRTYWGRCTCGYWYYCATGYMYWTAACMCMRYRCWMTGTWRYKTAAT 32700
Bhutan-09024          TCRWRYSRYGTTWKRTYWGRCTCGYWYYCATGYMYWTAACMCMRYRCWMTGTWRYKTAAT 32700
Bhutan-09027          TCRWRYSRYGTTWKRTYWGRCTCGYWYYCATGYMYWTAACMCMRYRCWMTGTWRYKTAAT 32700
Bhutan-09030          TCRWRYSRYGTTWKRTYWGRCTCGYWYYCATGYMYWTAACMCMRYRCWMTGTWRYKTAAT 32700
Bhutan-09005          YYRTAYCACRWKAGRTTKSRSYTAYTYYTRYATMYWWMRYMMMRCAMAAYRYARTTYRRY 32700
Indonesia-88035       YYRTATCACRWGAGRKTKSRSYTAYTYYTRYATMYWWMRYMMMRCAMAACRTARTTYRRY 32700
Indonesia-88045       YYRTATCACRWGAGRKTKSRSYTAYTYYTRYATMYWWMRYMMMRCAMAACRTARTTYRRY 32700
Indonesia-88065       YYRTAYCACRWKAGRTTKSRSYTAYTYYTRYATMYWWMRYMMMRCAMAAYRYARTTYRRY 32700
```

```
                       *   .       *.  .*.  .* **   . ***    * **         * .

Bhutan-09015         YMRAGRKMWCRASTTTKYAGRSYWYTCTYYRTGGRRTCARCMRACYYTGGYWWKARYTYA 32760
Bhutan-09024         YMRAGRKMWCRASTTTKYAGRSYWYTCTYYRTGGRRTCARCMRACYYTGGYWWKARYTYA 32760
Bhutan-09027         YMRAGRKMWCRASTTTKYAGRSYWYTCTYYRTGGRRTCARCMRACYYTGGYWWKARYTYA 32760
Bhutan-09030         YMRAGRKMWCRASTTTKYAGRSYWYTCTYYRTGGRRTCARCMRACYYTGGYWWKARYTYA 32760
Bhutan-09005         CCGRRAKCATGTSAKAKTTSGCYTYYYYCTAYRAGRCTGAYMRGMCCKKATWWTRAYWCR 32760
Indonesia-88035      CCGRRAKCATRTGATAKTTSRCYTYYYYCTAYRAGRCTGAYMRGMCCKKATWWTRAYWCR 32760
Indonesia-88045      CCGRRAKCATRTSAKAKTTSRCYTYYYYCTAYRAGRCTGAYMRGMCCKKATWTTRAYWCR 32760
Indonesia-88065      CCGRRAKCATGTSATAKTTSGCYTYYYYCTAYRAGRCTGAYMRGMCCKKATWATRAYWCR 32760
                           *    :.:.:* :. .* *        . *  .  **.   . . * .  *

Bhutan-09015         YWRYYAATRGYKMSRAYTAGCYCGSKRYTYYACGYTRAYRTTCAAYASYTATYAGKARAR 32820
Bhutan-09024         YWRYYAATRGYKMSRAYTAGCYCGSKRYTYYACGYTRAYRTTCAAYASYTATYAGKARAR 32820
Bhutan-09027         YWRYYAATRGYKMSRAYTAGCYCGSKRYTYYACGYTRAYRTTCAAYASYTATYAGKARAR 32820
Bhutan-09030         YWRYYAATRGYKMSRAYTAGCYCGSKRYTYYACGYTRAYRTTCAAYASYTATYAGKARAR 32820
Bhutan-09005         YTACCRMWRRYTMSARTKMAATTRGKATWTTWMRYYRGCATTMMATMGTKWYCWRTWARG 32820
Indonesia-88035      YTAYCRMWRRYTMSARTKMAATTRGKATWTTWMRYTRGCAWKMMRTMGTKWTCWRTWARG 32820
Indonesia-88045      YTAYCRMWRRYTMSARTKMAATTRGKATWTTWMRYTRGCAWKMMRTMGTKWTCWRTWARG 32820
Indonesia-88065      YTAYCRMWRRYTMSARTKMAATTRGKATWTTWMRYYRGCATTMMATMGTKWYCWRTWARG 32820
                     *       * *.**   . ..   .*        * *.   .     . .     .

Bhutan-09015         TCAGCYRGYYAGKGCYRGTTGWGKMYMGTTAWRTWCAGCYRYMGMSTRAARGTAKYTTRR 32880
Bhutan-09024         TCAGCYRGYYAGKGCYRGTTGWGKMYMGTTAWRTWCAGCYRYMGMSTRAARGTAKYTTRR 32880
Bhutan-09027         TCAGCYRGYYAGKGCYRGTTGWGKMYMGTTAWRTWCAGCYRYMGMSTRAARGTAKYTTRR 32880
Bhutan-09030         TCAGCYRGYYAGKGCYRGTTGWGKMYMGTTAWRTWCAGCYRYMGMSTRAARGTAKYTTRR 32880
Bhutan-09005         WMRSMTRGYTRSGCTCARCCRTRTMTATYYRAKYAYMKMCGYMTMCYRRMGRKWKYTKRR 32880
Indonesia-88035      WMRSMTRRYTRSGSTCARCCRTRTMTAGYYRAKYAYMKMCGYMTMCYRRMGRKWKYYKRR 32880
Indonesia-88045      WMRSMTRRYTRSGSTCARCCRTRTMTAGYYRAKYAYMKMCGYMTMCYRRMGRKWKYYKRR 32880
Indonesia-88065      WMRSMTRGYTRSGCTCARCCRTRTMTATYYRAKYAYMKMCGYMTMCYRRMGRKWKYTKRR 32880
                        .  * *  .           .*       :        ** *. *    . **  .**

Bhutan-09015         GARGRYCRTGMYRATKRYKRYTYCTWGCGRRWYRRTTYYYRKGRWWTGAYRCCYSTYYCK 32940
Bhutan-09024         GARGRYCRTGMYRATKRYKRYTYCTWGCGRRWYRRTTYYYRKGRWWTGAYRCCYSTYYCK 32940
Bhutan-09027         GARGRYCRTGMYRATKRYKRYTYCTWGCGRRWYRRTTYYYRKGRWWTGAYRCCYSTYYCK 32940
Bhutan-09030         GARGRYCRTGMYRATKRYKRYTYCTWGCGRRWYRRTTYYYRKGRWWTGAYRCCYSTYYCK 32940
Bhutan-09005         TAGSGYSAWRCCRCYGGYKGTKYTWARGAGAAYGAYWTCTRKRRATWRWCAYYYCCCTMG 32940
Indonesia-88035      TMGSGYSAWRCCRCYGGYKGTKYTWARGAGAAYGAYWTCTRKRRATWGWCAYYYCCCTMS 32940
Indonesia-88045      TMGSGYSAWRCCRCYGGYKGTKYTWARGAGAAYGAYWTCTRKRRATWGWCAYYYCCCTMS 32940
Indonesia-88065      TAGSGYSAWRCCRCYGGYKGTKYTWARGAGAAYGAYWTCTRKRRATWGWCAYYYCCCTMG 32940
                        . *.     *.   **  .*     .   *       ** *         *.

Bhutan-09015         TWTARRRYACRYAATRCRTTYRRTYRRYRTCYAWYRYGCRGYCTTYYGRCKRWCCARAGC 33000
Bhutan-09024         TWTARRGTACRYAATACRTTYRRTYRGYRTCYAWYRYGCRGYCTTYYGRCKRWCCARAGC 33000
Bhutan-09027         TWTARRGTACRYAATRCRTTYRRTYRRYRTCYAWYRYGCRGYCTTYYGRCKRWCCARAGC 33000
Bhutan-09030         TWTARRRYACRYAATRCRTTYRRTYRRYRTCYAWYRYGCRGYCTTYYGRCKRWCCARAGC 33000
Bhutan-09005         CAWMRRGTGYACRRYAYAYYTARGTRRCGYMTTWYATRYACCYYYYTTAYTRAYTRAWRY 33000
Indonesia-88035      TATARRGTGYACRRYAYAYYTARGTRRCGYMTTWYATATACCYYYYTTAYTRAYTRAWRY 33000
Indonesia-88045      TATARRGTGYACRRYAYAYYTARGTRRCGYMTTWYATRYACCYYYYTTAYTRAYTRAWRY 33000
Indonesia-88065      CAWMRRGTGYACRRYAYAYYTARGTRRCGYMTTWYATATACCYYYYTTAYTRAYTRAWRY 33000
                         **  .             *  *      :**          *    .*

Bhutan-09015         KMKGRTRGARYAWSTTYYTMYAARWCYRWARTCGRGGCYYAYYRWRYGRYKYAGSARRYC 33060
Bhutan-09024         KMKGRTRGARYAWSTTYYTMYAARWCYRWARTCGRGGCYYAYYRWRYGRYKYAGSRRRYC 33060
```

```
Bhutan-09027         KMKGRTRGARYAWSTTYYTMYAARWCYRWARTCGRGGCYYAYYRWRYGRYKYAGSARRYC 33060
Bhutan-09030         KMKGRTRGARYAWSTTYYTMYAARWCYRWARTCGRGGCYYAYYRWRYGRYKYAGSARRYC 33060
Bhutan-09005         KAGRGTGRWGTTTSYYCCKMYRWATYYAWMGYYAGCAGCCTYTAAACGGTGTWRSRGRYY 33060
Indonesia-88035      KAGRGTGRWGTTTSYYCCKMYRWATYYAWMGYYAGCGGCCTYTAAACAGTGTWASAGRYY 33060
Indonesia-88045      KAGRGWGRWGTTTSYYCCKMYRWATYYAWMGYYAGCGGCCTYTAAACAGTGTWRSAGRYY 33060
Indonesia-88065      KAGRGTGRWGTTTSYYCCKMYRWATYYAWMGYYAGCAGCCTYTAAACGGTGTWRSAGRYY 33060
                     *          : *    .**     * *    .  .   :*     .      *  **

Bhutan-09015         AYCCGYKRRRCCWMARYTGCRRRTCTSCGRYYRGARTRKRRWRYYCATYKKRSYGYGRRR 33120
Bhutan-09024         AYCCGYTARRCCWMARYTGCRRRYCTSCGRYYRGARTRKRRWRYYCATYKKRSYGYGRRR 33120
Bhutan-09027         AYCCGYKRRRCCWMARYTGCRRRYCTSCGRYYRGARTRKRRWRYYCATYKKRCYGYGRRR 33120
Bhutan-09030         AYCCGYTARRCCWMARYTGCRRRYCTSCGRYYRGARTRKRRWRYYCATYKKRCYGYGRRR 33120
Bhutan-09005         RTYSKYKRARYYTCRACCAMAGATYYCYRRYYRRAGYATRGAGCTSRYCGGACTSCSRGR 33120
Indonesia-88035      RTYSKYTAARYYTCRACCAMAGATYYCYRRYYRRRGYATRGAGCTSRYCGGASTSCSRGR 33120
Indonesia-88045      RTYSKYTAARYYTCRACCAMAGATYYCYRRYYRRRGYATRGAGCTSRYCGGASTSCSRGR 33120
Indonesia-88065      RTYSKYTAARYYTCRACCAMAGATYYCYRRYYRRAGYATRGAGCTSRYCGGASTSCSRGR 33120
                        . *.  *        .       .  ****     .*     .      . . .* *

Bhutan-09015         SCCGGGWRRCRWSRSTGGYYAMYARYCYKYKYWRMKKRMCTWYMYRTYYRKRMCGRCASG 33180
Bhutan-09024         SCCRGGWRRCRWSRSTGGYYAMYARYCYKYKYWRMKKRMCTWYMYRTYYRKRMCGRCASG 33180
Bhutan-09027         SYCRRGWRRCRWSRSTGGYYAMYARYCYKYKYWRMKKRMCTWYMYRTYYRKRMCGRCASG 33180
Bhutan-09030         SCCGRGWRRCRWSRSTGGYYAMYARYCYKYKYWRMKKRMCTWYMYRTYYRKRMCGRCASG 33180
Bhutan-09005         CCYGGRARGTGWGAGYKACCMACRRCYCGYKYWRMGKGMYYAYMYRCTTRGGMSKGATCR 33180
Indonesia-88035      CCCAGRARGTGWGAGYKACCMACRRCYCGYKYWRMGKGMYYAYMYRCTTRGGMSKGATCR 33180
Indonesia-88045      CCCAGRARGTGWGAGYKACCMACRRCYCGYKYWRMGKGMYYAYMYRCTTRGGMSKGATCR 33180
Indonesia-88065      CCCAGRARGYGWGAGYKACCMACRRCYCGYKYWRMGKGMYYAYMYRCTTRGGMSKGATCR 33180
                     .      *   *. .  .      *    ****** * *   ****   *  *.  .:.

Bhutan-09015         KYTCTGYCYMCRRTCYCATAGCRTTRCGYYGATRRGYGARYYARKAWMCTAYYAKYYCMR 33240
Bhutan-09024         KYTCTGYCYMCRRTCYCATAGCRTTRCGYYGATRRGYGARYYARKAWMCTAYYAKYYCMR 33240
Bhutan-09027         KYTCTGYCYMCRRTCYCATAGCRTTRCGYYGATRRGYGARYYARKAWMCTAYYAKYYCMR 33240
Bhutan-09030         KYTCTGYCYMCRRTCYCATAGCRTTRCGYYGATRRGYGARYYARKAWMCTAYYAKYYCMR 33240
Bhutan-09005         GTYGKACYYCAGAYYYYRYRRYRYKRTKTTKGYAGRTRMGYYRGTRAAMKRCCWKTTYCG 33240
Indonesia-88035      GTYGKACYYCAGAYYYYRYRRYRYKATKTTKGYAGRTRMGYYRGTRAAMKRCCWKTTYCG 33240
Indonesia-88045      GTYGKACYYCAGAYYYYRYRRYRYKATKTTKGYAGRTRMGYYRGTRAAMKRCCWKTTYCG 33240
Indonesia-88065      GTYGKACYYCAGAYYYYRYRRYRYKRTKTTKGYAGRTRMGYYRGTRAAMKRCCWKTTYCG 33240
                         ..  * .    *      * .      .        **  .    .    *

Bhutan-09015         YMACGGTMTTCRCTCGGGYMRACYWRRWRGCYKYGCCYYRARGAKYCRTKRKYWYWYRKT 33300
Bhutan-09024         YMACGGTMTTCRCTCGGGCMRACYWRRWRGCYKYGCCYYRARGAKYCRTKRKYWYWYRKT 33300
Bhutan-09027         YMACGGTMTTCRCYYRGGYMRACYWRRWRGCYKYGCCYYRARGAKYCRTKRKYWYWYRKT 33300
Bhutan-09030         YMACGGTMTTCRCYYRGGYMRACYWRRWRGCYKYGCCYYRARGAKYCRTKRKYWYWYRKT 33300
Bhutan-09005         YAWYRRWCAGYAYTCGRRCMRRYYTGRWRASTTYRMSTTGRRACKYYRYGAKCACTTAKC 33300
Indonesia-88035      YAWYRRWCAGYAYTCGRRCMRRYYTGRWRASTTYRMSTTGRRACKYYRTGAKCACTTAKC 33300
Indonesia-88045      YAWYRRWCAGYAYTCGRRCMRRYYTGRWRASTTYRMSTTGRRACKYYRTGAKCACTTAKC 33300
Indonesia-88065      YAWYRRWCAGYAYTCGRRCMRRYYTGRWRASTTYRMSTTGRRACKYYRYGAKCACTTAKC 33300
                     *       :          **  *  ***.. .*  .    *..** *   *      *

Bhutan-09015         RCAMWRYYYMARCWWRWRGYMGRMRYYTGRGCCARYCCTYYGTKMYYRSRRAAYYATKYA 33360
Bhutan-09024         RCAMWRYYYMARCWWRWRGYMGRMRYYTGRGCCARYCCTYYGTKMYYRSRRAAYYATKYA 33360
Bhutan-09027         RCAMWRYYYMARCWWRWRGYMGRMRYYTGRGCCARYCCTYYGTKMYYRSRRAAYYATKYA 33360
Bhutan-09030         RCAMWRYYYMARCWWRWRGYMGRMRYYTGRGCCARYCCTYYGTKMYYRSRRAAYYATKYA 33360
Bhutan-09005         GTRAWAYTCCRRYWWRWRSTCRRCRYYYARGYSRGYYSYTTSYKMYCACGGWMCYMYKCW 33360
Indonesia-88035      GTRAWAYTCCRRYWWRTGGTCGACRTTTARGYSAGYCCTTTGTGATCACGGWMCYMTGCA 33360
```

```
Indonesia-88045      GTRAWAYTCCRRYWWRTGGTCGACRTTTARGYSAGYCCTTTGTGATCACGGWMCYMTGCA 33360
Indonesia-88065      GTRAWAYTCCRRYWWRWRSTCRRCRYYYARRYSRGYYSYTTSYKMYCACGGWMCYMYKCW 33360
                         * *    * ***  .     *   .*  .  * .   .      .     *

Bhutan-09015         CYATRATYTCRYGRGYAYTMSYTTKRCTTRSGKRGGTYYTSWCRKGRCWCSKYRRSKTSC 33420
Bhutan-09024         CYATRATYTCRYGRGYAYTMSYTTKRCTTRSGKRGGTYYTSWCRKGRCWCSKYRRSKTSC 33420
Bhutan-09027         CYATRATYTCRYGRGYWYTMSYTTKRCTTRSGKRGGTYYTSWCRKGRCWCSKYRRSKTSC 33420
Bhutan-09030         CYATRATYTCRYGRGYWYTMSYTTKRCTTRSGKRGGTYYTSWCRKGRCWCSKYRRSKTSC 33420
Bhutan-09005         YYTKGMWYTYSCAGRYWYYASYWGKGGCKASSYGGTWCTWGWYRKRRSTYSKCRRCTYGY 33420
Indonesia-88035      CTTTGATCWCGCAGRCTYTASYWGKGGCKASSYGATWTTWGATATGGCTCGTCAGCTTGC 33420
Indonesia-88045      CTTTGATCWCGCAGRCTYTASYWGTGGCKASSYGATWTTWGATATGGCTCGTCAGCTTGC 33420
Indonesia-88065      YYTKGMWYTYSCAGRCWYYASYWGKGGCKASSYGGTWYTWGWYRKRRSTYSKCRRCTYGY 33420
                       :.        .    *  **  .   . *.  .     .   .  .  ..   .. .

Bhutan-09015         YYTRWYTYWRYGCYKKRYKCSTMYWRWTARTRWGGSRSMKAARYSYGGYWCMSGSCGKTC 33480
Bhutan-09024         YYTRWYTYWRYGCYKKRYKCGTMYWRWTARTRWGGSRSMKAARYSYGGYWCMCGSCGKTC 33480
Bhutan-09027         YYTRWYTYWRYGCYKKRYKCGTMYWRWTAGTRWGGSRSMKAARYSYGGYWCMSGSCGKTC 33480
Bhutan-09030         YYTRWYTYWRYGCYKKRYKCGTMYWRWTARTRWGGSRSMKAARYSYGGYWCMSGSCGKTC 33480
Bhutan-09005         YYKRWTWTWRCRMYTKRYKTSGMYARTKWGYAWRRSASMTRRATCTRRTAYMCGSCRTAA 33480
Indonesia-88035      TCTGATTTARYGCYTTGYKTCGMCAATKAGTAAGGCAGCTAAATCTGGTACACKCMRTAA 33480
Indonesia-88045      TCTGATTTARYGCYTTGYKTCGMCAATKAGTAAGGCAGCTAAATCTGGTACACKCMRTAA 33480
Indonesia-88065      YYKRWTWTWRCRMYTKRYKTSGMYARTKWGYAWRRSASMTAAATCTRRTAYMCGSCRTAA 33480
                       .      *   *.. **   *    .       . . .    .       . .  .:.

Bhutan-09015         MYRTRYYKRYACTGGGTYSGMGWWYAAMGTCCATSCRWYGMKRCCARCWMTCCWMTGWSY 33540
Bhutan-09024         MYRTRYYKRYACTGGGTYSGMGWWYAAMGTCCATSCGWYGMKRCCARCWMTCCWMTGWSY 33540
Bhutan-09027         MYRTRYYKRYACTGGGTYSGMGWWYAAMGTCCATSCRWYGMKRCCARCWMTCCWMTGWSY 33540
Bhutan-09030         MYRTRYYKRYACTGGGTYSGMGWWYAAMGTCCATSCRWYGMKRCCARCWMTCCWMTGWSY 33540
Bhutan-09005         CCAYRCYTRYRYKRSRYCCGARAWYWMARYYMWWCMRWTSCGRSYRRTWAKYSWMKKWSC 33540
Indonesia-88035      CCATACTTGTACTGCGTCCGAGAWTAAAGTTCATCCATTGCGASYRRTTATCCTCTGACC 33540
Indonesia-88045      CCATACTTGTACTGCGTCCGAGAWTAAAGTTCATCCATTGCGASYRRTTATCCTCTGACC 33540
Indonesia-88065      CCAYRCYTRYRYKRSRYCCRARAWYWMAACCMWWCMGWTSCGRSYRRTWAKYSWMKKWSC 33540
                            .    .     .    *          .    .   .  *   . .  .  .

Bhutan-09015         RCATYRRKYRKTATYAGMWRRRKYGRRYTAACWTWRWYRCRMRARRAATRYWGYRYYAYG 33600
Bhutan-09024         RCATYRRKYRKTATYAGMWRRRKYGARYTAACWTWRWYRCRMRARRAATRYWGYRYYAYG 33600
Bhutan-09027         RCATYRRKYRKTATYAGMWRRRTYGRRYTAACWTWRWYRCRMRARRAATRYWGYRYYAYG 33600
Bhutan-09030         RCATYRRKYRKTATYAGMWRRRTTGRRYTAACWTWRWYRCRMRARRAATRYWGYRYYAYG 33600
Bhutan-09005         AYRWYRSTCAKYGYTWTAARGATYRARYWRMYTYAGWCAAGCRRGRRMKRTWAYAYCRCK 33600
Indonesia-88035      ACATCGGTCATCGCTWTAAGGATYRAATTGCTTTAGTCAAGCGAGAAATGTWAYAYCRCK 33600
Indonesia-88045      ACATCGGTCATCGCTWTAAGGATYRAATTGCTTTAGTCAAGCGAGAAATGTWAYAYCRCK 33600
Indonesia-88065      AYRWYRSTCAKYGYTWTAARGATYRARYWRMYTYAGWCAAGCRRGRRMKRTWAYAYCRCK 33600
                            .  . .         .                .        .  *.* *

Bhutan-09015         CGCAGWTCTCYYASYCGAGTTARYKWRMGAWTMRTARRSRYRAWARRYRKYAWCYYRAAY 33660
Bhutan-09024         CGCAGWTCTCYYRSYCGAGTTARYKWRMGAWTMRTARRSRYRAWARRYRKYAWCYYRAAY 33660
Bhutan-09027         CGCAGWTCTCYYASYCGAGTTARYKWRMGAWTMRTARRSRYAAWARRYRKYAWCYYRAAY 33660
Bhutan-09030         CGCAGWTCTCCYASYCGAGTTARYKWRMGAWTMRTAAGCGYRAWARRYRKYAWCYYRAAY 33660
Bhutan-09005         TRMGKAYYYYYTRSYAARRKWWACKTRMCWWYCRWRAGCGCGRTRRGYGKYWAGTTRRWC 33660
Indonesia-88035      TRMGKAYYYYYTRSYAARRKWTACKTRMCWWYCRWRAGCGCGRTRRGYGKYWAGTTRRWC 33660
Indonesia-88045      TRMGKAYYYYYTRSYAARRKWWACKTRMCWWYCRWRAGCGCGGTRRGYGKYWAGTTRRWC 33660
Indonesia-88065      TRMGKAYYYYYTRSYAARRKWWACKTRMCWWYCRWRAGCGCGRTRRGYGKYWAGTTRRWC 33660
                        .         **..  .    * **  *  *    .      * * **     *
```

```
Bhutan-09015        TMYKSSYWTAGYSRWWTSAGRKRARRGTWWRYATCYYWYACGAGGCTRAGGCAATATATG 33720
Bhutan-09024        TMYKSSYWTAGYSRWWTSAGRKRARRGTWWRYATCYYWYACGAGGCTRAGGCAATATATG 33720
Bhutan-09027        TMYKSSYWTAGYSRWWTSAGRKRARRGTWWRYATCYYWYACGAGGCTRAGGCAATATATG 33720
Bhutan-09030        TMYKSSYWTAGYSRWWTSAGRKRARRGTWWRYATCYYWYACGAGGCTRAGGCAATATATG 33720
Bhutan-09005        WACGSCYAYWRYSRAAYCGTAGGRGGKWWTRYRWSTCACRCRMSRYYGGRRSRMYWKRCA 33720
Indonesia-88035     WACGGCCATAGCCGAACCGTAGGAGGKTATACGTCTCACAMGAGGCYGGARSAATATATG 33720
Indonesia-88045     WACGGCCATAGCCGAACCGTAGGAGGKTATACGTCTCACAMGAGGCYGGRRSAATATATG 33720
Indonesia-88065     WACGSCYAYWRYSRAAYCGTAGGRGGKWWTRYRWSTCACRCRMSRYYGGRRSRMYWKRYR 33720
                        ..      .    ..               .        .    .  .    .

Bhutan-09015        YGTTYMYRTSYRAMCTYCKCWRYTAAYGRAAYYWTRSATGYYKGSRARYCKCRSRRARAR 33780
Bhutan-09024        YGTTYMYRTSYRAMCTYCTCWRYTAAYGRAAYYWTRSATGYYKGSRARYCKCRSRRARAR 33780
Bhutan-09027        YGTTYMTRTSYRAMCTYCTCWRYTAAYGRAAYYWTRSATGYYKGSRARYCKCRSRRARAR 33780
Bhutan-09030        YGTTYMYRTSYRAMCTYCTCWRYTAAYGRAAYYWTRSATGYYKGSRARYCKCRSRRARAR 33780
Bhutan-09005        YRKYYATGWSYRRAYKCCTCTAYYMMTKGRRCTAWACRWKTYGRCKGRCSGYGGGRWARR 33780
Indonesia-88035     YGTTYATGTGTGRAYTCMTMTAYYAAYGGAACTAWACGTGTTGACGGACCGTGGGRWARR 33780
Indonesia-88045     YGTTYATGTGTGRAYTCMTMTAYYAAYGGAACTAWACGTGTTGACGGACCGTGGGRWARR 33780
Indonesia-88065     YRKYYATGWSYRRAYKCCKCTAYYMMTKGRRCTAWACRWKTYGRCKGRCSGYGGGRWARR 33780
                    * . *    .     .  .   *             .       . .  .   . *   *

Bhutan-09015        TAYRGGRWRGRKYYMCYSRRWMTTRTATAAYYCCCTWWWRYWCYWGTSYAGTTRGAGGYR 33840
Bhutan-09024        TAYRGGRWRGRKYYMCYSRAAMTTRTATAAYYCCCTWWWRYWCYWGTSYAGTTRGAGGYR 33840
Bhutan-09027        TAYRGGRWRGRKYYMCYSRRWMTTRTATAAYYYCCTWWWRYWCYWGTSYAGTTRGAGGYR 33840
Bhutan-09030        TAYRGGRWRGRKYYMCYSRAAMTTRTATAAYYCCCTWWWRYWCYWGTSYAGTTRGAGGYR 33840
Bhutan-09005        KRCRKKATRSRTCTMYYSRAAAYWAWMYWRYTTYMYAAWAKWSTWKYGYWSWWRRRSSCR 33840
Indonesia-88035     TACRGGATGGGTCTCYCGAAAATTATATAAYTTCCTAAAATTCTTGTGTAGTTGGAGGCA 33840
Indonesia-88045     TACRGGATGGGTCTCYCGAAAATTATATAAYTTCCTAAAATTCTTGTGTAGTTGGAGGCA 33840
Indonesia-88065     KRCRKKATRSRTCTMYYSRAAAYWAWMYWRYTTYMYAAWAKWSTWKYGYWSWWRRRSSCR 33840
                    .  *     . .     .            *           .    .  .     ..

Bhutan-09015        GSGAGCCWYKCCAYMRGCCWSAASCYCGCRYYKTYRYMYMSTYKARMCCYTAMRARRKAM 33900
Bhutan-09024        GSGAGCCATGCCAYMRGCCWSAASCYCGCRYYKTYRYMYMSTYKARMCCYTAMRARRKAM 33900
Bhutan-09027        GSGAGCCWYKCCAYMRGCCWSAASCYCGCRYYKTYRYMYMSTYKARMCCYTAMRARRKAM 33900
Bhutan-09030        GSGAGCCWYKCCAYMRGCCWSAASCYCGCRYYKTYRYMYMSTYKARMCCYTAMRARRKAM 33900
Bhutan-09005        KGACKTMWMGYSRCCRTYMTSGGSGTMAMAYTKKYRYAYMGCCGMGMSYYGRCAWGGGGC 33900
Indonesia-88035     TGACTTCTAGCCRCCRTYMTSGGSGTMAMAYTKKYRYAYMGCCGMGMSYYGRCAWGGGGC 33900
Indonesia-88045     TGACTTCTAGCCRCCRTYMTSGGSGTMAMAYTKKYRYAYMGCCGMGMSYYGRCAWGGGGC 33900
Indonesia-88065     KGACKTMWMGYSRCCRTYMTSGGSGTMAMAYTKKYRYAYMGCCGMGMSYYGRCAWGGGGC 33900
                     ...       .   *    *..*   .  * *.*** **.     *. *        .

Bhutan-09015        RRYRRWTTRRRGYKAATKGYYCMYRGWGTAAGRRKYTGRGCGMCWSRRYYMGMTWTARRR 33960
Bhutan-09024        RRYRRWTTRRRGYKAATKGYYCMYRGWGTAAGRRKYTGRGCGMCWSRRYYMGMTWTCRRR 33960
Bhutan-09027        RRYRRWTTRRRGYKAATKGYYCMYRGWGTAAGRRKYTGRGCGMCWSRRYYMGMTWTMRRR 33960
Bhutan-09030        RRYRRWTTRRRGYKAATKGYYCMYRGWGTAAGRRKYTGRGCGMCWSRRYYMGMTWTCRRR 33960
Bhutan-09005        AGTAAWYWAAARYKGRYKRTYYCTGRWSWWGTARYTYRRAMRMMWSRRCTCAMYWYCGRR 33960
Indonesia-88035     AGTAAWYWAAARYKGRYKRTYYCTGRWSWWGTARYTYRRAMRMMWSRACTCAMYWYTGRR 33960
Indonesia-88045     AGTAAWYWAAARYKGRYKRTYYCTGRWSWWGTARYTYRRAMRMMWSRRCTCAMYWYAGRR 33960
Indonesia-88065     AGTAAWYWAAARYKGRYKRTYYCTGRWSWWGTARYTYRRAMRMMWSRRCTCAMYWYTGRR 33960
                         *      **.  *  *     *.  .  *    *.  * ***    .* *   **

Bhutan-09015        TAARCTRRYYRRAWYWSCYSYYTGYWYRAACYASTSKRGRSYWRAWWMYWTTSMCASYAC 34020
Bhutan-09024        TAARCTRRYYRRAWYWSCYGYYTGYWYRAACYASTSKRGRSYWRAWWMYWTTSMCASYAC 34020
Bhutan-09027        TAARCTRRYYRRAWYWSCYGYYTGYWYRAACYASTSKRGRSYWRAWWMYWTTSMCASYAC 34020
Bhutan-09030        TAARCTRRYYRRAWYWSCYGYYTGYWYRAACYASTSKRGRSYWRAWWMYWTTSMCASYAC 34020
```

```
Bhutan-09005        YRRGYYRGTTRRRTYTCYYGYTWRTTYRGCTCGSWCKARASCWRMATMTWWCGMYRSCRY 34020
Indonesia-88035     YRRGYYRGTTRRRTYTCYYGYTWRTTYRGCTCGSWCKARASCWRMATMTWWCGMYRSCRY 34020
Indonesia-88045     YRRGYTRGTTRRRTYTCYYGYTWRTTYRGCTCGSWCKARASCWRMATMTWWCGMYRSCRY 34020
Indonesia-88065     YRRGYTRGTTRRRTYTCYYGYTWRTTYRGCTCGSWCKARASCWRMATMTWWCGMYRSCRY 34020
                          *   **  * . *.*     **..  .* .*   * **   * *  .*  *

Bhutan-09015        WRTYAYRYCGMGAGYKCCCRTTYYMYASTCATRWWYTTAWRRAAASKARRSWRTYRTYCG 34080
Bhutan-09024        WRTYAYRYCGMGAGYKCCCRTTYYMYASTCATRWWYTTAWRRWAASKARRSWRTYRTYCG 34080
Bhutan-09027        WRTYAYRYCGMGAGYKCCCRTTYYMYASTCATRWWYTTAWRRWAASKARRSWRTYRTYCG 34080
Bhutan-09030        WRTYAYRYCGMGAGYKCCCRTTYYMYASTCATRWWYTTAWRRAAASKARRSWRTYRTYCG 34080
Bhutan-09005        TRKYRYRTYSCARRYTTMYRYCYTMYGCCYTWRWWTCYWAAAARMSSMGRSAGATGWTTR 34080
Indonesia-88035     TRKYRYRTYSCARRYTTMYRYCYTMYGCCCTWRWWTCYWAAAARMSSMGRSAGATGWTTR 34080
Indonesia-88045     TRKYRYRTYSCARRYTTMYRYCYTMYGCCCTWRWWTCYWAAAAGCSSMGRSAGATGWTTR 34080
Indonesia-88065     TRKYRYRTYSCARRYTTMYRYCYTMYGCCCTWRWWTCYWAAAAGCSSMGRSAGATGWTTR 34080
                     *.* **  . .  *.   *  * **..  : ***          *.  **  :

Bhutan-09015        WMMYCRTTMGCYYGWACYRKYYSCMRGTGAAYMAKSMWRTCWTCTRGYYGKSAGMCRGRM 34140
Bhutan-09024        WMMYCRKTMGCYYGWACYRKCYSCMRGTGAAYMAKSMWRTCWTCTRGYYGKSAGMCRGRM 34140
Bhutan-09027        WMMYCRTTMGCYYGWACYRKYYSCMRGTGAAYMAKSMWRTCWTCTRGYYGKSAGMCRGRM 34140
Bhutan-09030        WMMYCRTTMGCYYGWACYRKYYSCMRGTGAAYMAKSMWRKYWTCTRGYYGKSAGMCRGRM 34140
Bhutan-09005        TCMTTGTWMASTTRATTCAGTCSMAGKYRRRYWRTSCWATCACTWASCYKKSMKAYRRAC 34140
Indonesia-88035     TCMTTGTWMASTTRATTCAGTCSMAGKYRRRYWRTSCWATCACYWASCYKKSMKAYRRAC 34140
Indonesia-88045     TCMTTGKWMASTTRATTCAGTCSMAGKYRRRYWRTSCWATCACYWASCYKKSMKAYRRAC 34140
Indonesia-88065     TCMTTGTWMASTTRATTCAGTCSMAGKYRRRYWRTSCWATCACYWASCYKKSMKAYRRAC 34140
                      *   . *..    :      *        *  .* * .      . * **    *

Bhutan-09015        YKGCWCASRRYRMSRWYAYRGMGMYWYYRCGCYSSTGMYYCAAYSKRGCCCRTRRKAWWS 34200
Bhutan-09024        YGGCWCASRRYRMSRWYAYRGMGMYWYYRCGCYSSTGMYYCAAYSKRGCCCRTRRKAWWS 34200
Bhutan-09027        YGGCWCASRRYRMSRWYAYRGMGMYWYYRCGCYSSTGMYYCWAYSKRGCCCRTRRKAWWS 34200
Bhutan-09030        YKGCWCASRRYRMSRWYAYRGMGCYWYYRCGCYSSTGMYYCAAYSKRGCCCRTRRKAWWS 34200
Bhutan-09005        TTRYTAMGAATRCSRAYRCGRARAYACYGMKYCCCYTMYTTARYSTRAATTRYAAGWWWC 34200
Indonesia-88035     TTGYTAMGAATRCSRAYRCGRARAYACYGMKYCCCYTMYTTARYSTRAATTRYAAGWWWC 34200
Indonesia-88045     TTRCTAMGAATRCSRAYRCGRARAYACYGMKYCCCYTMYTTARYSTRAATTRYAAGWWWC 34200
Indonesia-88065     TTRYTAMGAATRCSRAYRCGRARAYACYGMKYCCCYTMYTTARYSTRAATTRYAAGWWWC 34200
                         . .   * ** *       *  *     ..  **    **.*..  *     **.

Bhutan-09015        YYACCSAGCYRTGCTRYWWRRCAGGGRMYRCGWWYYYTTRWRWMRATYTTTCRRAAYMWY 34260
Bhutan-09024        YYACCSAGCYRTGCTRYWWRRCAGGGRMYRCGWWYYYTTRWRWMRATYTTTCRRAAYMWY 34260
Bhutan-09027        YYACCSAGCYRTRMTRYWWRRCAGGGRMYRCGWWYYYTTRWRWMRATYTTTCRRAAYMWY 34260
Bhutan-09030        YYACCSAGCYRTRMTRYWWRRCAGGGRMYRCGWWYYYTTRWRWMRATYTTTCRRAAYMWY 34260
Bhutan-09005        TTGYSCMAMYACGCARCWTAGYRRKTACCAGTTTCTYWYAAGTMARYTCKWSAAWRTAAY 34260
Indonesia-88035     TTGYSCMAMYACGCARCWTAGYRRKTACCAGTTTCTYWYAAGTMARYTCKWSAAWRTAAY 34260
Indonesia-88045     TTGYSCMAMYACGCARCWTAGYRRKTACCAGTTTCTYWYAAGTMARYTCKWSAAWRTAAY 34260
Indonesia-88065     TTGYSCMAMYACGCARCWTAGYRRKTACCAGTTTCTYWYAAGTMARYTCKWSAAWRTAAT 34260
                      . .. . *    :* *                  *      *     . .

Bhutan-09015        YWCCACACYTMYWGTTSRYACTMTGKTYCRYRTYGTRYMSTTKWGRTKYYKSKWRRCGYA 34320
Bhutan-09024        YWCCACACYTMYWGTTSRYACTMTGKTYCRYRTYGTRYMSTTKWGRTKYYKSKWRRCGYA 34320
Bhutan-09027        YACCACACYTMYWGYYSRYACTMTGKTYCRYRTYGTRYMSTTKWGRTKYYKSKWRRCGYA 34320
Bhutan-09030        YWCCACACYTMYWGTTSRYACTMTGKTYCRYATTGTRYMSTTKWGRTKYYKSKWRRCGYA 34320
Bhutan-09005        YTYMTMRMTWMCTATTSATMYYMYRKCYAACRYTKYATAGYYTTRRYKYCKGKWRGYRYW 34320
Indonesia-88035     YTYMTMRMTWMCTATTGATMYYMYRKCYAACRYTKYATAGYYTTRRYKYCKGKAAGYRYW 34320
Indonesia-88045     YTYMTMRMTWMCTATTSATMYYMYRKCYAACRYTKYATAGYYTTRRYKYCKGKWRGYRYW 34320
Indonesia-88065     YTYMTMRMTWMCTATTSATMYYMYRKCYAACRYTKYATAGYYTTRRYKYCKGKWRGYRYW 34320
```

```
                     *   :     *  .  .     *  * *.          .  .  * ** *.*     *

Bhutan-09015         YARWRMTYYAYRRYTGRKYMKGTGWKGTAMAWSMRGRCGAYGYYTTYYKGKCARYYMTRK 34380
Bhutan-09024         YARWRMWYYAYRRYTGRKYMKGTGWKGTAMAWSMRGRCGAYGYYTTYYKGKCARYYMTRK 34380
Bhutan-09027         YARWRMWYYACRRYTGRKYMKGTGWKGTAMAWSMRGRCGAYGYYTTYYKGKCARYYMTRK 34380
Bhutan-09030         YARWRMTYYACRRYTGRKYMKGTGWKGTAMAWSMRGRCGAYGYYTTYYKGKCARYYMTRK 34380
Bhutan-09005         YRRWRMTYTMTGGCTRRKTATAYKAGACWRWTGCATRYRGYKCTCCYTKAKYGGTTAWRG 34380
Indonesia-88035      YRRARCWTTMTGAYYGGKTATAYKAGACWRWTGCATRYRGYKCTCCYTKAKYGGTTAWRG 34380
Indonesia-88045      YRRARMWYTMTGATYGGKTATAYKAGACWRWTGCATRYRGYKCTCCYTKAKYGGTTAWRG 34380
Indonesia-88065      YRRARMWYTMTGGCTRRKTATAYKAGACWRWTGCATRYRGYKCTCCYTKAKYGGTTAWRG 34380
                     * * *            *  ..    .     .   *  .*     * *.* .     *

Bhutan-09015         AAAGAYRATGSRTAYCKSMYYTMRRYCASAYSYYAYKWAYYRKATMSYGRKRTACRKTGT 34440
Bhutan-09024         AAAGAYRATGSRTAYCKSMYYTMRRYCASAYSYYAYKWAYYRKATMSYGRKRTACRKTGT 34440
Bhutan-09027         AAAGAYRATGSRTAYCKSMYYTMRRYCASAYSYYAYKWAYYRKATMSYGRKRTACRKTGT 34440
Bhutan-09030         AAAGAYRATGSRTAYCKSMYYTMRRYCASAYSYYAYKWAYYRKATMSYGRKRTACRKTGT 34440
Bhutan-09005         RRGRRTGATACRYGYYGSAYYWCRRTSMCTYCTCRCGAWTYRGRCCSTSAGGWRTATWRW 34440
Indonesia-88035      RRGRRTGRYACRYGYTGSAYYWCRRTSMCTYCTCRCGAWTYRGRCMSTSAGGWRCATWRW 34440
Indonesia-88045      RRGRRTGRYACRYGYYGSAYYWCRRTSMCTYCTCRCGAWTYRGRCMSTSAGGWRCATWRW 34440
Indonesia-88065      RRGRRTGATACRYGYYGSAYYWCRRTSMCTYCTCRCGAWTYRGRCCSTSAGGWRTATWRW 34440
                       .      ..* .*  * **  ** . .:*.        **    * .       .

Bhutan-09015         AMWRRKTWAYRKRYRTWYAKRAATSKAGTRCTCCRWACGYAKYRYRTAYRGGACCYSAGR 34500
Bhutan-09024         AMWRRKTWAYRKRYRTWYAKRAATSKAGTRCTCCRWACGYAKYRYRTAYRGGACCYSAGR 34500
Bhutan-09027         AMWRRKTWAYRKRYRTWYAKRAATSKAGTRCTCCRWACGYAKYRYRTAYRGGACCYSAGR 34500
Bhutan-09030         AMWRRKTWAYRKRYRTWYAKRAATSKAGTRCTCCRWACGYAKYRYRTAYRGGACCYSAGR 34500
Bhutan-09005         TMTAGGGWRYRKRTGCWTRTRWRYGTCAWAYATYAARYKCGKCGTAWRYRRRRTACSRSG 34500
Indonesia-88035      TMTAGGGWRYRKRTGCAYRTRWRYGTCAWAYATYAARYKCGKCGTAWRYGRRRTCCSRGG 34500
Indonesia-88045      TMTAGGGWRYRKRTGCWYRTRWRYGTCAWAYATYAARYKCGKCGTAWRYGRRRTCCSRGG 34500
Indonesia-88065      TMTAGGGWRYRKRTGCATRTRWRYGTCAWAYATYAARYKCGKCGTAWRYGRRRTACSRSG 34500
                     :*     * ****      .*   ....   :        .*      *     . * .

Bhutan-09015         YTRYARRTYMRRCYWYKYGTSAAYMTSWKSKTRYRGTTKRCTRTGTRYGCAWSWTYYAYC 34560
Bhutan-09024         YTRYARRTYMRRCYWYKYGTSAAYMTSWKSKTRYRGTTKRCTRTGTRYGCAWSWTYYAYC 34560
Bhutan-09027         YTRYARRTYMRRCYWYKYGTSAAYMTSWKSKTRYRGTTKRCTRTGTRYGCAWSWTYYAYC 34560
Bhutan-09030         YTRYARRTYMRRCYWYKYGTSAAYMTSWKSKTRYRGTTKRCTRTGTRYGCAWSWTYYAYC 34560
Bhutan-09005         CKRTMRACTCAGMYTTTYKYCWRCMYCATSTYGCATGKTRYTRYRTATRYRWGTAYYRCM 34560
Indonesia-88035      CKRYARACTCAGMYTTTYKYSAACMYCATSTYGCATGKTRYWGYRWATRTRTGTAYYGCM 34560
Indonesia-88045      CKRYARACTCAGMYTTTYKYSAACMYCATSTYGCATGKTRYWGYRWATRTRTGTAYYGCM 34560
Indonesia-88065      CKRTMRACTCAGMYTTTYKYCWRCMYCATSTYGCATGKTRYTRYRTATRYRWGTAYYRCM 34560
                      .*  *       *  .*  .   * . .*.      ..*            . :**

Bhutan-09015         RTCATYGRCYMMYYMRTTKRRAKYRYKKSTYMYYARMWATTTRAAAWRGTGCYRKKASRG 34620
Bhutan-09024         RTCATYGRCYMMYYMRTTKRRAKYRYKKSTYMYYARMWATTTRAAAWRGTGCYRKKASRG 34620
Bhutan-09027         RTCATYGRCYMMYYMRTTKRRAKYRYKKSTYMYYARMWATTTRAAAWRGYGCYRKKASRG 34620
Bhutan-09030         RTCATYGRCYMMYYMRTTKRRAKYRYKKSTYMYYARMWATTTRAAAWRGYGCYRKKASRG 34620
Bhutan-09005         AYMATCRGYTMATTCAAYGGRCKTGTSGCYYMYTAGMARCGCGRMTTRRTAGYGKTRCAR 34620
Indonesia-88035      AYCATCRGYTMATTCAAYGGRCKTGTSGCYYMYTRGMARCGCGRMTTRRTAGYGKTRCAR 34620
Indonesia-88045      AYCWWCRGYTMATTCAAYGGRCKTGTSGCYYMYTRGMARCGCGRMTTRRTAGYGKTRCAR 34620
Indonesia-88065      AYMATCRGYTMATTCAAYGGRCKTGTSGCYYMYTRGMAGCGCGRMTTRRTAGYGKTRCAR 34620
                               *     :   *.*   . . ***   *        : *  . * *. .

Bhutan-09015         ARCRRARRGAARYGYYTAKAYYRRTRAKWYSATAYSSYKTYTRAGTYYRSGWYATGCTAG 34680
Bhutan-09024         ARCRRARRGAARYGYYTAKAYYRRTRAKWYSATAYSSYKTYTRAGTYYRSGWYRTGCTAG 34680
```

```
Bhutan-09027         ARCRRARRGAARYGYYTAKAYYRRTRAKWYSATAYSSYKTYTRAGTYYRSGWYATGCTAG 34680
Bhutan-09030         ARCRRARRGAARYGYYTAKAYYRRTRAKWYSATAYSSYKTYTRAGTYYRSGWYRTGCTAG 34680
Bhutan-09005         GAMAGTAAAWRACRCYKRKRYYAGKRMGATCGYRTSCTTYYYRRTYCTASSACAWCSYWS 34680
Indonesia-88035      GAMAGTAAAWRACRCYKRKRYYAGKRMGATCGYRTSCTTYYYRRTYCTASSACAWCSYWS 34680
Indonesia-88045      GAMAGTAAAWRACRCYKRKRYYAGKRMGATCGYRTSCTTYYYRRTYCTASSACAWCSYWS 34680
Indonesia-88065      GAMAGTAAAWRACRCYKRKRYYAGKRMGATCGYRTSCTTYYYRRTYCTASSACAWCSYWS 34680
                     .    :  .      *. * **  .*    ..   *. . * *      *.     .  .

Bhutan-09015         RTWKRYRCRRWRKAAGRARTGCYWYRRACMRYTWWCRAYGSRYAMRSRRWATCGAWCCAT 34740
Bhutan-09024         RTWKRYRCRRWRKARRRARTGCYWYRRACMRYTWWCRAYGSRYAMRSRRWATCGAWCCAT 34740
Bhutan-09027         RTWKRYRCRRWRKARRRARTGCYWYRRACMRYTWWCRAYGSRYAMRSRRWATCGAWCCAT 34740
Bhutan-09030         RTWKRYRCRRWRKARRRARTGCYWYRRACMRYTWWCRAYGSRYAMRSRRWATCGAWCCAT 34740
Bhutan-09005         AYATAYRYGRTRKRRARRRYRMCTCRARMAATYWAYARCRSGCRMARAAAWYYKRTMSRC 34740
Indonesia-88035      AYATAYRYGRTRKRRARRRYRMCTCRARMAATYWAYARCRSGCRMARAAAWYYKRTMSRC 34740
Indonesia-88045      AYATAYRYGRTRKRRARRRYRMCTCRARMAATYWAYARCRSGCRMARAAAWYYKATMSRC 34740
Indonesia-88065      AYATAYRYGRTRKRRARRRYRMCTCRARMAATYWAYARCRSGCRMARAAAWYYKRTMSRC 34740
                        . **  * **   * *      *       *      *   *            .

Bhutan-09015         GYYRWYRKYYCMSRATYYAYRTTYMYRKAYACATAYRGYGWYWAGYAYTGRYYGYRAYSM 34800
Bhutan-09024         GYYRWYRKYYCMSRATYYAYRYTYMYRKAYACATAYRGYGWYWAGYAYTGRYYGYRAYSM 34800
Bhutan-09027         GYYRWYRKYYCMSRATYYAYRYTYMYRKAYACATAYRGYGWYWAGYAYTGRYYGYRAYSM 34800
Bhutan-09030         GYYRWYRKYYCMSRATYYAYRYTYMYRKAYACATAYRGYGWYWAGYAYTGRYYGYRAYSM 34800
Bhutan-09005         ACTRWYRTCYSCSAMWYYRYGYYYACRKRCCTWYRCGRCRWCWMSTTYYKACYTCRRCCC 34800
Indonesia-88035      ACTRWYRTCYSCSAMWYYRYGYYYACRKRCCTWYRCGRCRWCWMSTTYYKACYTCRACCC 34800
Indonesia-88045      ACTRWYRTCYSCSAMWYYRYGYYYACRKRCCTWYRCGRCRWCWMSTTYYKACYTCRACCC 34800
Indonesia-88065      ACTRWYRTCYSCSAMWYYRYGYYYACRKRCCTWYRCGRCRWCWMSTTYYKACYTCRRCCC 34800
                     .  ****. *.  *  ** *   *  **  .         * * . :*    *  *  .

Bhutan-09015         RWCRTAGMGKTYWAWACRRRYYCMRASMYGRTTTCMSCTKACAGWRYRCSYWYARAYKGK 34860
Bhutan-09024         RWCRTAGMGKTYWAWACRRRYYCMRASMYGRTTTCMSCTKACAGWRYRCSYWYARAYKGK 34860
Bhutan-09027         RWCRTAGMGKTYWAWACRRRYYCMRASMYGRTTTCMSCTKACAGWRYRCSYWYARAYKGK 34860
Bhutan-09030         RWCRTAGMGKTYWAWACRRRYYCMRASMYGRTTTCMSCTKACAGWRYRCSYWYARAYKGK 34860
Bhutan-09005         GAYAKGRCSGWTWCTWYGAMYCYCGCCCTRAYACGMGMWTGMGRWGTGYSCWYWRRCKKT 34860
Indonesia-88035      GAYAKGRCGGWTWCTWYGAMYCYCGCCCTRAYACGMGMWTGMGRWGTGYSCWYWRRCKKT 34860
Indonesia-88045      GAYAKGRCGGWTWCTWYGAMYCYCGCCCTRAYACGMGMWTGMGRWGTGYSCWYWRRCKKT 34860
Indonesia-88065      GAYAKGRCSGWTWCTWYGAMYCYCGCCCTRAYACGMGMWTGMGRWGTGYSCWYWRRCKKT 34860
                         ..  .   *.      *    ..     :  *.  .. . *    * ** *  * .

Bhutan-09015         RGGTARRTAWRRTYYGASYMRAYMYYYYMCTGRGYRKSRTCTMTWWWYTAYYAMARKYAW 34920
Bhutan-09024         RGGTARRTAWRRTYYGASYMRAYMYYYYMCTGRGYRKSRTCTMTWWWYTAYYAMARKYAW 34920
Bhutan-09027         RGGTARRTAWRRTYYGASYMRAYMYYYYMCTGRGYRKSRTCTMTWWWYTAYYAMARKYAW 34920
Bhutan-09030         RGGTARRTAWRRTYYGASYMRAYMYYYYMCTGRGYRKSRTCTMTWWWYTAYYAMARKYAW 34920
Bhutan-09005         AKAWRAGWWWRRWCTSWGCMRRYACTCTMYCAASYATSRYSYMYTATCYGYYRMWAGCRA 34920
Indonesia-88035      AKAWRAGWWWRRWCTSWGCMRRYACTCTMYCAASYATSRYSYMYTATCYGYYRMWAGCRA 34920
Indonesia-88045      AKAWRAGWWWRRWCTSWGCMRRYACTCTMYCAASYATSGYSYMYTATCYGYYRMWAGCRR 34920
Indonesia-88065      AKAWRAGWWWRRWCTSWGCMRRYACTCTMYCAASYATSGYSYMYTATCYGYYRMWAGCRA 34920
                       .      ***   . . ** *     *  . .* .*  . *      .** *

Bhutan-09015         TTYYMMMMYGRSTTASTCRSYWAGKTRTTTRWCTTRWSMSMKYMGKMRAWACYYRGAARW 34980
Bhutan-09024         TTYYMMMMYGRSTTASTCRSYWAGKTRTTTRWCTTRWSMSMKYMGKMRAWACYYRGAARW 34980
Bhutan-09027         TTYYMMMMYGRSTTASTCRSYWAGKTRTTTATCTTRWSMSMKYMGKMRAWACYYRGAARW 34980
Bhutan-09030         TTYYMMMMYGRSTTASTCRSYWAGKTRTTTRWCTTRWSMSMKYMGTMRAWACYYRGAARW 34980
Bhutan-09005         YCTTMMACYARSKYGCYMRGTWRSTKRKCYATCYYGWSMSMKTMCTCARWMTTCAAWMRW 34980
Indonesia-88035      YCTTMMACYARSTYGCYMRGTWRSTKRKCYRTYYTGWSMSMKTMCTCARWMTTCAAWMRW 34980
```

```
Indonesia-88045       YCTTMMACYARSTYGCYMRGTWRSTKRKCYRTYYYGWSMSMKTMCTCARWMTTCAAWMRW 34980
Indonesia-88065       YCTTMMACYARSKYGCYMRGTWRSTKRKCYRTYYYGWSMSMKTMCTCARWMTTCAAWMRW 34980
                          **  *.**. ..  *. * ...*.        ****** * .   *     .  **

Bhutan-09015          TRKRRWAGAMYTCTRATYKYTTAMRGSTCRWAWAWSSYSKMYCKCYYGYWTGRGTSAAYC 35040
Bhutan-09024          TRKRRWAGAMYTCTRATYKYTTAMRGSTCRWAWAWSSYSKMYCKCYYGYWTGRGTSAAYC 35040
Bhutan-09027          TRKRRWAGAMYTCTRATYKYTTACRGSTCRWAWAWSSYSKMYCKCYYGYWTGRGTSAAYC 35040
Bhutan-09030          TRKRRWAGAMYTCTRATYKYTTAMRGSTCRWAWAWSSYSKMYCKCYYGYWTGRGTSAAYC 35040
Bhutan-09005          YAGRRAWKWCCYGWRATTGYYTRMRKSAYGTWTWWSRTCTACYGTYTKCAYRRGYCMRTY 35040
Indonesia-88035       YAGRRAWKWCCYGWRRYTGYYYRMRKSAYGTTTATCGTCTACCGTYTKCATGRRTCAATT 35040
Indonesia-88045       YAGRRAWKWCCYGWRRYTGYYYRMRKSAYGTTTATCGTCTACCGTYTKCATGRRTCAATT 35040
Indonesia-88065       YAGRRAWKWCCYGWRATTGYYTRMRKSAYGTWTWWSRTCTACYGTYTKCAYRRGYCMRTY 35040
                         **         *    *    * *:       .  ..     *      *  .

Bhutan-09015          WAACYRWYTKSTYTRAWYTTGAYGYWYMATRRGSMTASWYRAGMGRYYRGYKYYCTWYKA 35100
Bhutan-09024          WAACYRWYTKSTTTRAWYTTGAYGYWYMATRRGSMTASWYRAGMGRYYRGYKYYCTWYKA 35100
Bhutan-09027          WAACYRWYTKSTYTRAWYTTGAYGYWYMATRRGSMTASWYRAGMGRYYRGYKYYCTWYKA 35100
Bhutan-09030          WAACYRWYTKSTYTRAWYTTGAYGYWYMATRRGSMTASWYRAGMGRYYRGYKYYCTWYKA 35100
Bhutan-09005          AMMMTATCYKSYCYGRWYYKKRYRTTTCRYARSGAYRGACAWAAKACCRRCGTYYYTYKM 35100
Indonesia-88035       ACACTATCTGCCCTGAATTGTACGTTTCGCARGGATAGACAWGAGACCGGCGTTCTTCTA 35100
Indonesia-88045       ACACTATCTGCCCTGAATTGTACGTTTCGCARGGATAGACAWGAGACCGGCGTTCTTCTA 35100
Indonesia-88065       AMMMTATCYKSYCYGRWYYKKRYRTTTCAYARSGAYRGACAWAAKACCRRCGTYYYTYKM 35100
                                .                    *..   .    .               .

Bhutan-09015          RYRMGWKTRAWRMRSWSYRTAWTCGGAGRRYWRWMYRKTWYGAMTWRKWYTWMMGTRWCY 35160
Bhutan-09024          RYRMGWKTRAWRMRSWSYRTAWTCGGAGRRYWRWMYRKTWYGAMTWRKWYTWMMGTRWCY 35160
Bhutan-09027          RYRMGWKTRAWRMRSWSYRTAWTCGGAGRRYWRWMYRTTWYGAMTWRKWYTWMMGTRWCY 35160
Bhutan-09030          RYRMGWKTRAWRMRSWSYRTAWTCGGAGRRYWRWMYRTTWYGAMTWRKWYTWMMGTRWCY 35160
Bhutan-09005          ATAAKTKKRMTAAAGTCCRKATWMRGWRGGTWGWCTGAYRYRMMYTGKWYWAAMRYRWCC 35160
Indonesia-88035       ATAAKTGTGATAAAGTCTATWTTCGKAGGGTWGTCTGACRYGMCTTGTATTAAAGYRATC 35160
Indonesia-88045       ATAAKTGTGATAAAGTCTATWTTCGKAGGGTWGTCTGACRYGMCTTGTATTAAAGYRATC 35160
Indonesia-88065       ATAAKTKKRMTAAAGTCCRKATWMRGWRGGTWGWCTGAYRYRMMYTGKWYWAAMRYRWCC 35160
                             .      . .  .           *        *      .        *

Bhutan-09015          RRGRGAATWRWAAMTCAYKYASYCRCGAGYCWRWMGRMSRWRWAKYSCRRRSYAAMYGGC 35220
Bhutan-09024          RRGRGAATWAWAAMTCAYKYASYCRCGAGYCWRWMGRMSRWRWAKYSCRRRSYAAMYGGC 35220
Bhutan-09027          RRGRGAATWRWAAMTCAYKYASYCRCGAGYCWRWMGRMSRWRWAKYSCRRRSYAAMYGGC 35220
Bhutan-09030          RRGRGAATWAWAAMTCAYKYASYCRCGAGYCWRWMGRMSRWRWAKYSCRRRSYAAMYGGC 35220
Bhutan-09005          AGKAKMRWTRAWMMGCGTKCRCTYAMKAGTCAATCGGASGAGTTTTSMRGAGYMMWYKAM 35220
Indonesia-88035       AGGAGMRTTAAAAMGMGTKCRCTCACGAGTSAATCAGASGAGTTTTSCRGAGTAAATGAM 35220
Indonesia-88045       AGGAGMRTTAAAAMGCGTKCRCTCACGAGTSAATCAGASGAGTTTTSCRGAGTAAATGAM 35220
Indonesia-88065       AGKAKMRWTRAWMMGCGTKCRCTYAMRRRTCAATCGGASGAGTTTTSMRGAGYMMWYKAM 35220
                                   *  . *  .        .    .  *    :. * *  .      .

Bhutan-09015          TARRSRRAGYYRKKWCAYYYAKCYRTRYYTAKAKCRTCCGGYYTYGCYYRRRKMSRAGGC 35280
Bhutan-09024          TARRSRRAGYYRKKWCAYYYAKCYRTRYYTAKAKCRTCCGGYYTYGCYYRRRKMSRAGGC 35280
Bhutan-09027          TARRSRRAGYYRKKWCAYYYAKCYRTRYYTAKAKCRTCCGGYYTYGCYYRRRKMSRAGGC 35280
Bhutan-09030          TARRSRRAGYYRKKWCAYYYAKCYRTRYYTAKAKCRTCCGGYYTYGCYYRRRKMSRAGGC 35280
Bhutan-09005          WCRASRAASCTRTGTSGCYYRTYYGYRTCWRKRTYAYSYRSYYYTSYCTGRRTCSRWGGC 35280
Indonesia-88035       WMAACGAWGCTATGTGGCCCATCCGTGTCTATATCAYSCGGTTYTGCCTGGATCCAAKGC 35280
Indonesia-88045       WMAACGAWGCTATGTGGCCCATCCGTGTCTATATCAYSCGGTTYTGCCTGGATCCAAGGA 35280
Indonesia-88065       WCRASRAASCTRTGTSGCYYRTYYGYRTCWRKRTYAYSYRSYYYTSYCTGRRTCSRAGAC 35280
                          .   .   .   .    .         . .   .  .    .      . .   ..
```

```
Bhutan-09015         ARRRKGGATMYSTMTCTCRYWRMATRRYYGKSYYTTRWGRSKGTAKYAAACRRYKTMGAR 35340
Bhutan-09024         ARRRKGGATMYSTMTCTCRYWRMATRRYYGKSYYTTRWGRSKGTAKYAAACRRYKTMGAR 35340
Bhutan-09027         ARRRKGGATMYSTMTCTCRYWRMATRRYYGKSYYTTRWGRSKGTAKYAAACRRYKTMGAR 35340
Bhutan-09030         ARRRKGGATMYSTMTCTCRYWRMATRRYYGKSYYTTRWGRSKGTAKYAAACRRYKTMGAR 35340
Bhutan-09005         WRRGTKKMYCYCYCYSYYGTWAAWYAATTGGGYYKWAARGGKKYRGYRGMYRRYKYARRA 35340
Indonesia-88035      AAAGTGGATCYCTCTCTCGTTAAATAATTSGGTCKTAAGGGGKYRGTAGACAGTGTAGAA 35340
Indonesia-88045      AAAGTGGATCYCTCTCTCGTTAAATAATTSGGTCKTAAGGGGKYRGTAGACAGTGTAGAA 35340
Indonesia-88065      WRRGTKKMYCYCYCYSYYGTWAAWYAATTGGGYYKWAARGGKKYRGYRGMYRRYKCARRA 35340
                         .     *.   .             . .  .     .       .

Bhutan-09015         RYRGAKTWTWKKRGTCYRAGACGGTRATCCTGYYTGCYRKWYYCGYGCTGYYCGYCGTAS 35400
Bhutan-09024         RYRRMKTWTWKKRGTCYRAGACGGTRATCCTGYYTGCYRKWYYCGYGCTGYYCKYCGTAS 35400
Bhutan-09027         RYRRMKTWTWKKRGTCYRAGACGGTRATCCTGYYTGCYRKWYYCGYGCTGYYCKYCGTAS 35400
Bhutan-09030         RYRRMKTWTWKKRGTCYRAGACGGTRATCCTGYYTGCYRKWYYCGYGCTGYYCGYCGTAS 35400
Bhutan-09005         RYRGAGWAWTKKARWMYRRRRYRRYARYCCYKTTYRYYATWYYYRTASYRTCCKYAATRC 35400
Indonesia-88035      ACAGAGTATTTGAGTCCRAGACGRTAATYYTGTTTGCCATTYYYGTACTGTCCKYAAWAC 35400
Indonesia-88045      ACAGAGTATTTGAGTCCRAGACGRTAATYYTGTTTGCCATTYYYGTACTGTCCKYAAWAC 35400
Indonesia-88065      RYRGAGWAWTKKARWMYRRRRYRRYARYCCYKTTYRYYATWYYYRTASYRTCTKYAATRC 35400
                               .      *                     . **   ..      *..  .

Bhutan-09015         TGCYKRRASWWKWYCYKTCCGWCWAGCCGAGARGSARCACGRWRACYCMGYAATYTCRGR 35460
Bhutan-09024         TGCYKRRASWWKWYCYKTCCGWCWAGCCGAGARGSARCACGRWRACYCMGYAATYTCRGR 35460
Bhutan-09027         TGCYKRRASWWKWYCYKTCCGWCWAGCCGAGARGSARCACGRWRACYCMGYAATYTCRGR 35460
Bhutan-09030         TGCYKRRASWWKWYCYKTCCGWCWAGCCGAGARGSARCACGRWRACYCMGYAATYTCRGR 35460
Bhutan-09005         YRTTKAAWGTAGATCTGYYYRWYWWRMMGWKRARGRAYAMGRTAWSTTMKTWRKTTYGRA 35460
Indonesia-88035      TRTTKAAAGTAGATYTGTCCGWCAAGCCKAGAAGGAACMCRATAACTTMGTAATTWCGGA 35460
Indonesia-88045      TGTTKAAAGTAGATYTGTCCGWCAAGCCKAGAAGGAACMCRATAACTTMGTAATTWCGGA 35460
Indonesia-88065      YRTTKAAWGTAGATCTGYYYRWYWWRMMGWKRARGRAYAMGRTAWSTTMKTWRKTTYGRA 35460
                         *   .            *            .          .  *    .

Bhutan-09015         RWMYMGCWMMTGWRMAGATRKKKWYMTMCCATAYTASYYGWTTTKMTRTKYACGTACTAA 35520
Bhutan-09024         RWMYMGCWMMTGWRMAGATRKKKWYMTMCCATAYTASYYGWTTTKMTRTKYACGTACTAA 35520
Bhutan-09027         RWMYMGCWMMTGWRMAGATRKKKWYMTMCCATAYTASYYGWTTTKMTRTKYACGTACTAA 35520
Bhutan-09030         RWMYMGCWMMTGWRMAGATRKKKWYMTMCCATAYTASYYGWTTTKMTRTKYACRTACTAA 35520
Bhutan-09005         AACTMRYTMCWSAACGRAYAGTGTYACAYMRCGTKWCTTKTTCGTATAYKYRYGYAYKRR 35520
Indonesia-88035      AACTCGCTMCWGWACGGWTAGTGTCAAACCACGTKWSTTGTTCGTATATTYACGCWYKAA 35520
Indonesia-88045      AACTCGCTMCWGWACGGWTAGTGTCAAACCACGTKWSTTGTTCGTAKATTYACGCWYKAA 35520
Indonesia-88065      AACTMRYTMCWSAACGRAYAGTGTYACAYMRCGTKWCTTKTYCGTATAYKYRYGYAYKRR 35520
                             *  .   .     .          . . .       . .  .*      .

Bhutan-09015         RRMWCWCCRSCRWTARKRRACTYRRAACGYYGGTTYWRRWYTYTYASRCSMCMYYRRGGG 35580
Bhutan-09024         RRMWCWCCRSCRWTARKRRACTYRRAACGYYGGTTYWRRWYTYTYASRCSMCMYYRRGGG 35580
Bhutan-09027         RRMWCWCCRSCRWTARKRRACTYRRAACGYYGGTTYWRRWYTYTYASRCSMCMYYRRGGG 35580
Bhutan-09030         RRMWCWCCRSCRWTARKRRACTYRRAACGYCGGTTYWRRWYTYTYASRCSMCMYYRRGGG 35580
Bhutan-09005         RRAWYTYTASYATARRTGRRMYTGRMRYRCCGTYWYTARWCYTKYRSRYCSTACCARRRK 35580
Indonesia-88035      GAATYTYTASYATAAATGRRMYTGAAACGCCKTTTTTAAACTTTTACGYCCTACCAAGGG 35580
Indonesia-88045      GAATYTYTASYATAAATGRRMYTGAAACGCCKTTTTTAAACTTTTACGYCCTACCAAGGG 35580
Indonesia-88065      RRAWYTYTASYATARRTGRRMYTGRMRYRCCGTYWYTARWCYTKYRSRYCSTACCARRRK 35580
                              *   :  . *                        .  .  .

Bhutan-09015         YMWKARWAGATWWKKAAGMYRTTGGCYSGYRCRYRAKGYYMTATAKYGCRRCWWRWKYWA 35640
Bhutan-09024         YMWKARWAGATWWKKAAGMYRTTGGCYSGYRCRYRAKGYYMTATAKYGCRRCWWRWKYWA 35640
Bhutan-09027         YMWKARWAGATWWKKAAGMYRTTGGCYSGYRCRYRAKGYYMTATAKYGCRRCWWRWKYWA 35640
Bhutan-09030         YMWKARWAGATWWKKAAGMYRTTGGCYSGYRCRYRAKGYYMTATAKYGCRRCWWRWKYWA 35640
```

```
Bhutan-09005          YATKWGWRKWYWAKKRWKMYRYWKSTYSRTRMACARTCTYAYWTRGYKYGGTATATTCTR 35640
Indonesia-88035       TATTAGAAGATAATGAAGCTATTGGYTGGTGMACAATCTCATWKRGYGCGGYATATTCTR 35640
Indonesia-88045       TATTAGAAGATAATGAAGCTATTGGYTGGTGMACAATCTCATWKGGYGCGGYATATTCTR 35640
Indonesia-88065       YATKWGWRKWYWAKKRWKMYRYWKSTYSRTRMACARTCTYAYWTRGYKYGGTATATTCTR 35640
                         .         .          .  .        .      .  *         .

Bhutan-09015          RRRRRYYCGWRRRYTAYRRRYRCTTGKMACYRTYYRRTTCKMAGRMSKRYCTARMATAMY 35700
Bhutan-09024          RRRRRYYCGWRRRYTAYRRRYRCTTGKMACYRTYYRRTTCKMAGRMSKRYCTARMATAMY 35700
Bhutan-09027          RRRRRYYCGWRRRYTAYRRRYRCTTGKMACYRTYYRRTTCKMAGRMSKRYCTARMATAMY 35700
Bhutan-09030          RRRRRYYCGWRRRYTAYRRRYRCTTGKMACYRTYYRRTTCKMAGRMSKRYCTARMATAMY 35700
Bhutan-09005          RRRRRTTYRTGGRYWWYGAAYGYYWRTARYYRKCCRRWTYKCWKAACGGCYYMRAWKWAY 35700
Indonesia-88035       RAAAATTCGTGGGCTACGAAYGCTTGTAACTAKCCAATYCTCAGAACGGCCTAAAATAAT 35700
Indonesia-88045       RAAAATTCGTGGGCTACGAAYGCTTGTAACTATCCAATYCTCAGAACGGCCTAAAATAAT 35700
Indonesia-88065       RRRRRTTYRTGGRYWWYGAAYGYYWRTARYYRTCCRRWTYKCWKAACGGCYYMRAWKWAY 35700
                      *                   *     .     .       .     .         .

Bhutan-09015          RWRRRCYMAKYKTCRYTYMRASYYTCACKGTTTRTCMAWYRYYYYMTRGRRCRRRGRYGS 35760
Bhutan-09024          RWRRRCYMAKYKTCRYTYMRASYYTCACKGTTTRTCMAWYRYYYYMTRGRRCRRRGRYGS 35760
Bhutan-09027          RWRRRCYMAKYKTCRYTYMRASYYTCACKGTTTRTCAAWYRYYYYMTRGRRCRRRGRYGS 35760
Bhutan-09030          RWRRRCYMAKYKTCRYTYMRASYYTCACKGTTTRTCMAWYRYYYYMTRGRRCRRRGRYGS 35760
Bhutan-09005          RWGGASTMMKCKYMRCYYMGMSYCYSRMKGKTKRYYCRWYGCYTYMGAKGGYGRRCRTRG 35760
Indonesia-88035       GTGGASTMMKCKTCACTCAGASTCTCACTRTYKRYYCRWYGCYTYMGAKGGYGRRCRTRG 35760
Indonesia-88045       GTGGASTMMKCKTCACTCAGASTCTCACTRTYKRYYCRWYGCYTYMGAKGGYGRRCRTRG 35760
Indonesia-88065       RWGGASTMMKCKYMRCYYMGMSYCYSRMKGKTKRYYCRWYGCYTYMGAKGGYGRRCRTRG 35760
                           . * * *         *   .  . . .*    **  * **       ** *  .

Bhutan-09015          AATYYGKATRYAGRAGYRRWTCGCMYGCCYTGGKCGWYYWGGKGRAGRWRKYKATGARMC 35820
Bhutan-09024          AATYYGKATRYAGRAGYRRWTCRCMYGCCYTGGKCGWYYWGGKGRAGRWRKYKATGARMC 35820
Bhutan-09027          AATYYGKATACAGRAGYRRWTCRCMYGCCYTGGKCGWYYWGGKGRAGRWRKYKATGARMC 35820
Bhutan-09030          AATYYGKATACAGRAGYRRWTCRCMYGCCYTGGKCGWYYWGGKGRAGRWRKYKATGARMC 35820
Bhutan-09005          RCWYYKTRWGTCARCTCAAAYTGMATKYTYYRRGYSAYYAAAKARMRRAAGCKRGTGGMY 35820
Indonesia-88035       RCTYYKTRWGTCARCTCAAAYYGMATKYTYTGAGYSAYYAAAKAGCRRAAGCKGGTGGMY 35820
Indonesia-88045       RCTYYKTRWGTCARCTCAAAYYGMATKYTYTGAGYSAYYAAAKARMRRAAGCKGGTGGMY 35820
Indonesia-88065       RCWYYKTRWGTCARCTCAAAYYGMATKYTYTGAGYSAYYAAAKARMRRAAGCKRGTGGMY 35820
                       . ** .    ..*.              *     . ** ..*.   *    *   . *

Bhutan-09015          CCGSWWKMGATTTCRGSWWYTMCAYYAYSYYSYKYYGRRCGCWWMAGSYMYARTTCGMMR 35880
Bhutan-09024          CCGSWWKMGATTTCRGSWWYTMCAYYAYSYYSYKYYGRRCGCWWMAGSYCYARTTCGMMR 35880
Bhutan-09027          CCGSWWKMGATTTCRGSWWYTMCAYYAYSYYSYKYYGRRCGCWWMAGSYMYARTTCGMMR 35880
Bhutan-09030          CCGSWWKMGATTTCRGSWWYTMCAYYAYSYYSYKYYGRRCGCWWMAGSYCYARTTCGMMR 35880
Bhutan-09005          TTAGTTKMCCCCCTGRCWWYCCYWYYTYSYTCYKYCRRGTAAAACCAGCCYMAGASRCMR 35880
Indonesia-88035       TTAGTTKMCCCCCTGRCWWYCCYWYYTYSYTCYKYCRRGTAAATCCAGCCYMRGASGCMR 35880
Indonesia-88045       TTAGTTKMCCCCCTGRCWWYCCYWYYTYSYTCYKYCRRGTAAATCCAGCCYMAGASGCMR 35880
Indonesia-88065       TTAGTTKMCCCCCTGRCWWYCCYWYYTYSYTCYKYCRRGTAAAACCAGCCYMAGASRCMR 35880
                        ..  ** .      .***    **:*** .***  *  ..   ...  *   :.  **

Bhutan-09015          RYYMRGWYYTAWTATTAGTYATTMWTYYYRRYTCYTACCMRCTTTACCYACCATCRACYA 35940
Bhutan-09024          RYYMRGWYYTAWTATTAGTYATTMWTYYYRRYTCYTACCMRCTTTACCYACCATCRACYA 35940
Bhutan-09027          RTYMRGWYYTAWTATTAGTYATTMWTTYYRRYTCYTACCMRCTTTACCYACCATCRACYA 35940
Bhutan-09030          RYYMRGWYYTAWTATTAGTYATTMWTTYYRRYTCYTACCMRCTTTACCYACCATCRACYA 35940
Bhutan-09005          GTYMGATCTWMTACGATRCCMYWWWWCTCAGCYYTYRYYARYYYYRYYYRYYRYYRCTYR 35940
Indonesia-88035       GTYMGATCTWMTACGATRCCAYWWWWCTCAGCYYTYRYYARYYYYRYYYRYYRYYRCTYR 35940
Indonesia-88045       GTYMGATCTWMTACGATRCCAYWWWWCTCAGCYYTYRYYARYYYYRYYYRYYRYYRCTYR 35940
Indonesia-88065       GTYMGATCTWMTACGATRCCMYWWWWCTCAGCYYTYRYYARYYYYRYYYRYYRYYRCTYR 35940
```

```
                                   ** .      :. ::       *               *       *      *. *

Bhutan-09015         GYACWRKRTRYYGATRRYGACTYAGARTCAKAARGRKATCTYRKMYCRRKKMMYYAYYWR 36000
Bhutan-09024         GYACWRKRTRYYGATRRYGACTYAGARTCAKAARGRKATCTYRKMYCRRKKMMYYAYYWR 36000
Bhutan-09027         GYACWRKRTRYYGATRRYGACTYAGARTCAKAARGRKATYKYRKMYCRRKKMMYYAYYWR 36000
Bhutan-09030         GYACWRKRTRYYGATRRYGACTYAGARTCAKAARGRKATYKYRKMYCRRKKMMYYAYYWR 36000
Bhutan-09005         RYTTTAKRAGCCSGGARYRMYACTARGAYCGRMRAATRCCTTRKCTYGGKKAACYRTCTG 36000
Indonesia-88035      RYTTTAKRAGCCSGGARYRMYACTARGAYCGRARAATRCCTTRKCTCGGKKAACYRTCTG 36000
Indonesia-88045      RYTTTAKRAGCCSGGARYRMYACTARGAYCGRARAATRCCTTRKCTCGGKKAACYRTCTG 36000
Indonesia-88065      RYTTTAKRAGCCSGGARYRCYACTARGAYCGRARAATRCCTTRKCTYGGKKAACYRTCTG 36000
                      *:   **:    ..  **   : :.  : .   *. .   . **     **   *

Bhutan-09015         YSKWAYYTGYTYYAWTSWMSWTYRRRRRCTARRCMARCAACYKGCKSYGGSYAGMRAWWG 36060
Bhutan-09024         YSKWAYYTGYTYYAWTSWMSWTYRRRRRCTARRCMARCWWCYKGCKSYGGSYAGMRAWWG 36060
Bhutan-09027         YSKWAYYTGYTYYAWTSWMSWTYRRRRRCTARRCMARCWWCYKGCKSYGGSYAGMRAWWG 36060
Bhutan-09030         YSKWAYYTGYTYYAWTSWMSWTYRRRRRCTARRCMARCWWCYKGCKSYGGSYAGMRAWWG 36060
Bhutan-09005         YCKAGYCYKTCCYGWCSWMGWKYGGGGGYYCAGYARRYAATYGGMGCYSRGCWRMGMTTR 36060
Indonesia-88035      YCKAGYCYKTCCYGWCSWMGWKYGGGGGYYCAGYARRYAATYGSMGCYSRGCWRMGMTTR 36060
Indonesia-88045      YCKAGYCYKTCCYGWCSWMGWKYGGGGGYYCAGYAGRYAWTYGGMGCYSRGCWRMGMTTR 36060
Indonesia-88065      YCKAGYCYKTCCYGWCSWMGWKYGGGGGYYCAGYARRYAWTYGGMGCYSRGCWRMGMTTR 36060
                     *.* .*      *.* ***.*.*        .     *    * .  .*. .   *

Bhutan-09015         YTATRGRYYYYGGKYYYRRAYWAARMATRYGRMWRRRRGAGYKTCYYGCAGMRRTMTACW 36120
Bhutan-09024         YTATRGRYYYYGGKYYYRRAYWAARMATRYGRMWRRRRGAGYKTCYYGCAGMRRTMTACW 36120
Bhutan-09027         YTATRGRYYYYGGKYYYRRAYWAARMATRYGRMWRRRRGAGYKTCCCGCAGMRRTMTACW 36120
Bhutan-09030         YTATRGRYYYYGGKYYYRRAYWAARMATRYGRMWRRRRGAGYKTCYYGCAGMRRTMTACW 36120
Bhutan-09005         CYRKAKGCTTTRRGTYYRGWCAGGGMRCGYKAAAAGAGRWRYTYGCCTTCAMRGTAGCTA 36120
Indonesia-88035      CYAKAKGCTTTRRGTYYRGWCARRGMRCGYKAAAAGAGRWRYTYGCCTTCAMRGKAGCTA 36120
Indonesia-88045      CYAKAKGCTTTRRGTYYRGWCARRGMRCGYKAAAAGAGRWRYTYGCCTTCAMRGTAGCTA 36120
Indonesia-88065      CYRGAKGCTTTRRGTYYRGWCAGGGMRCGYKAAAAGAGRWRYTYGCCTTCAMRGTAGCTA 36120
                                ***       *   *           *.      ..** .  .

Bhutan-09015         YKAYRKMSRCAKAMKGARYCYAYYCGCYCMWCCRYKRYKSYCRSKRRYSGYRYWTYCRAG 36180
Bhutan-09024         YKAYRKMSRCAKAMKGARYCYAYYCGCYCMWCCACKRYKSYCRSKRRYSGYRYWTYCRAG 36180
Bhutan-09027         YKAYRKMSRCAKAMKGARYCYAYYCGCYCMWCCACKRYKSYCRSTRRYSGYRYWTYCRAG 36180
Bhutan-09030         YKAYRKMSRCAKAMKGARYCYAYYCGCYCMWCCACKRYKSYCRCTRRYSGYRYWTYCRAG 36180
Bhutan-09005         TGRYRKAGGYRGGCKGARYAYGYYYRACYMATTACKGCTYYYSSKAATCATATTWYTRRA 36180
Indonesia-88035      TGRYRKAGGYRGGCKGAGYAYGYYYRACYMATTACKGCTYYYSSKAATCATAYTWYTRRA 36180
Indonesia-88045      TGRYRKAGGYRGGCKSWRYAYGYYYRACYMATTACKGCTYYYSSKAATCATAYTWYTGRA 36180
Indonesia-88065      TGRYRKAGGYRGGCKGARYAYGYYYRACYMATTACKGCTYYYSSKAATCATAYTWYTRRA 36180
                       *** .    . *.  *.*.**  .  *      *  . *  ..    ..     *   .

Bhutan-09015         RGYYRGRYSRWGWWKKKGTAGYYSCSAAMYKRYYRCARCSYRTRRGYWTRWWKARYYCAA 36240
Bhutan-09024         RGYYRGRYSRWGWWKKKGTAGYYSCSAAMYKRYYRCARCSYRTRRGYWTRWWKARYYCAA 36240
Bhutan-09027         RGYYRGRYSRWGWWKKKRTAGYYSCSAAMYKRYYRCARCSYRTRRGYWTRWWKARYYCAA 36240
Bhutan-09030         RGYYRGRYSRWGWWKKKRTAGYYSCSAAMYKRYYRCARCSYRTRRGYWTRWWKARYYCAA 36240
Bhutan-09005         RRYTRRACCGTSWWKKTRYRSCCCMGGWMTTATTAYGGTCCGCAACYWCRWWKGRYYMGG 36240
Indonesia-88035      RRYTRRACCGTSWWKKTRYRSCCCMGGWMTTATTAYGGTCCGCAACYWCRWWKGRYYMGG 36240
Indonesia-88045      RRYTRRACCGTSWWKKTRYRSCCCMGGWMTTATTAYGGTCCGCAACYWCRWWKGRYYMGG 36240
Indonesia-88065      RRYTRRACCGTSWWKKTRYRSCCCMGGWMTTATTAYGGTCCGCAACYWCRWWKGRYYMGG 36240
                     * * *    .  .****.   .  . .. * .     .  .      ** ****.*** ..

Bhutan-09015         TAWCMTGCCCTWWGTAGWRAAMYRRMAGAYACWYWTCAGRAMMRGCMYKRAAGCKYWCRS 36300
Bhutan-09024         TAWCMWGCCCTWWGTAGWRAAMYRRMAGAYACWYWTCAGRAMMRGCMYKRAAGCKYWCRS 36300
```

```
Bhutan-09027          TAWCMWGCCCTWWGTAGWRAAMYRRMAGAYACWYWTCAGRAMMRGCMYKRAAGCKYWCRS 36300
Bhutan-09030          TAWCMWGCCCTWWGTAGWRAAMYRRMAGAYACWYWTCAGRAMAGGCMYKRAAGCKYWCRS 36300
Bhutan-09005          YMWTMTAYYMYATAARSAARRACARCWKGTGTATACYMSRRAAGATMYKAGGTTTTWYRC 36300
Indonesia-88035       YMWTMTAYYMYATAARSAARRACARCWKGTGTATACYMSRRAAGATMYKAGGTTTTWYRC 36300
Indonesia-88045       YMWTMTAYYMYATAARSAARRACARCWKGTGTATACYMSRRAAGATMYKAGGTTTTWYRC 36300
Indonesia-88065       YMWTMTAYYMYATAARSAARRACARCWKGTGTATACYMSRRAAGATMYKAGGTTTTWYRC 36300
                        * * .      .: .       *   . .       .*    . *** ..  . * *.

Bhutan-09015          WYTGTKKGGRCTYRYYWRYYYCYYAMYYWYMWYYGRRYTGYTTGTKKCYATAYWMGRMGC 36360
Bhutan-09024          WYTGTKKGGRCTYRYYWRYYYCYYAMYYWYMWYYGRRYTGYTTGTKKCYATAYWMGRMGC 36360
Bhutan-09027          WYTGTKKGGRCTYRYYWRYYYCYYAMYYWYMWYYGRRYTGYTTGTKKCYATAYWMGRMGC 36360
Bhutan-09030          WYTGTKKGGRCTYRYYWRYYYCYYAMYYWYMWYYGRRYTGYTTGTKKCYATAYWMGRMGC 36360
Bhutan-09005          WYWAGKKRRRYYYRCTWRYCCSYCGCTTWTCTTTKRRYWATKYRYGKYYRYMCACARMAY 36360
Indonesia-88035       WYWAGKKRRRYYYRCTWRYCCSYCGCTTWTCTTTKRRYWATKYRYGKYYRYMCACARMAY 36360
Indonesia-88045       WYWAGKKRRRYYYRCTWRYCCSYCGCTTWTCTTTKRRYWATKYRYGKYYRYMCACARMAY 36360
Indonesia-88065       WYWAGKKRRRYYYRCTWRYCCSYCGCTTWTCTTTKRRYWATKYRYGKYYRYMCACARMAY 36360
                      ** . **  *  **  ***  .* .   *      *** . .    * *      .**.

Bhutan-09015          YCRGRCAKGCWRRGCTRRSTCRRATSKKRRGAAGCWAARCMCYRRCCTGTSMWRRYYYYR 36420
Bhutan-09024          YCRGRCAKRCARRGCTRRSTCRRATSKKRRGAAGCWAARCMCYRRCCTGTSMWRRYYYYR 36420
Bhutan-09027          YCRGRCAKRCWRRGCTRRSTCRRATSKKRRGAAGCWAARCMCYRRCCTGTSMWRRYYYYR 36420
Bhutan-09030          YCRGRCAKRCTRRGCTRRSTCRRATSKKRRGAAGCWAARCMCYRRCCTGTSMWRRYYYYR 36420
Bhutan-09005          TCASAGRGASTRAKYYRRCYYGGRKGGTRRTCWATATGGYATYGRTYWKYSCAARYTYYR 36420
Indonesia-88035       TYASAGRGASTRAKYYRRCYYGGRKGGTRRTCWATATGGCATYGRTYWKYSCAARYTYYR 36420
Indonesia-88045       TYASAGRGASTRAKYYRRCYYGGRKGGTRRTCWATATGGCATYGRTYWKYSCAARYTYYR 36420
Indonesia-88065       TCASAGRGASTRAKYYRRCYYGGRKGGTRRTCWATATGGCATYGRTYWKYSCAARYTYYR 36420
                         .     . *    **.     .. .** . .  :.    * *     *   ** ***

Bhutan-09015          RGYGGRYMSYAMRAKAAGARSGCGAYYMYRKRRGWMCGMGRGGRGCCYCKCMCGRGTYRG 36480
Bhutan-09024          RGYGGRYMSYAMRAKAAGARSGCGAYYMYRKRRGWMCGMGRGGRGCCYCKCMCGRGTYRG 36480
Bhutan-09027          RGYGGRYMSYAMRAKAAGARSGCRAYYMYRKRRGWMCGMGRGGRGCCYCKCMCGRGTYRG 36480
Bhutan-09030          RGYGGRYMSYAMRAKAAGARSGCRAYYMYRKRRGWMCGMGRGGRGCCYCKCMCGRGTYRG 36480
Bhutan-09005          GSCRRGCMCCTMGRGGRRRRSAYGWCTCTGTAGRAAYRMSGRRRKYYYYTMMMAGRWCAS 36480
Indonesia-88035       GSCRRGCMCCTMGRGGRRRRSAYRWCTCTGTAGRAAYRMSGRRRKYYYYTMMMAGRWCAS 36480
Indonesia-88045       GSCRRGCMCCTMGRGGRRRRSAYRWCTCTGTAGRAACRMSGRRRKYYYYTMMMAGRWCAS 36480
Indonesia-88065       GSCRRGCMCCTMGRGGRRRRSAYRWCTCTGTAGRAAYRMSGRRRKYYYYTMMMAGRWCAS 36480
                       .     *. :*   .   **.        .       *.   *   * . * .     .

Bhutan-09015          CGGMYYKRTTGRRGTYATGTCWRRRRKTGRSRRYGAYYRRMRKAAGRKYKWTMRCCGYYT 36540
Bhutan-09024          CGGMYYKRTTGRRGTYATGTCWRRRRKTGRSRRYGAYYRRMRKAAGRKYKWTMRCCGYYT 36540
Bhutan-09027          CGGMYYKRTTGRRGTYATGTCWRRRRKTGRSRRYGAYYRRMRKARGRKYKWTMRCCGYYT 36540
Bhutan-09030          CGGMYYKRTTGRRGTYATGTCWRRRRKTGRSRRYGAYYRRMRKARGRKYKWTMRCCGYYT 36540
Bhutan-09005          SGGATCKGYTTGRTYCRYRYYWGRRRKKRAGARYRWYYARMGTGRARGYKAYCASTKTYY 36540
Indonesia-88035       SRRATCKGYWKRGTYCRYRYYWGRRRKKRAGARYRWYYARMGTGAARGYKAYCASTKTYY 36540
Indonesia-88045       SRRATCKGYWKRGTYCRYRYYWGRRRKKRAGARYRWYYAAMGTGRARGYKAYCASTKTYY 36540
Indonesia-88065       SRRATCKGYWKGRTYCRYRYYWGRRRKKRAGARYRWYYARMGTGRARGYKAYCASTKTYY 36540
                      .     *              * ****.  . **  **  * .. .* **    .   *

Bhutan-09015          ARRGTCMRYYACWCCTRYRYYRYGGAARYRRSGYYCKMSRWTYSTYCMYSYRRCRRASYY 36600
Bhutan-09024          ARRGTCMRYYACWCCTRYRYYRYGGAARYRRSGYYCKMSRWTYSTYCMYSYRRCRRASYY 36600
Bhutan-09027          ARRGTCMRYYACWCCTRYRYYRYGGAARYRRSGYYCKMSRWTYSTYCMYSYRRCRRASYY 36600
Bhutan-09030          ARRGTCMRYYACWCCTRYRYYRYGGAARYRRSGYYCKMSRWTYSTYCMYSYRRCRRASYY 36600
Bhutan-09005          TRRCCTMRCYRTATAGATGYYRYGGAAAYRRCCCCYKCCGWWCCYCYCYSYGRTGRRGYY 36600
Indonesia-88035       TRRCCTMRCYRTATAGATRYYRYKRRWAYRRCSYCYKCCGWWCCYCYCYSYGRTGRRGYY 36600
```

```
Indonesia-88045      TRRCCTMRCYRTATAGATRYYRYKRRWAYRRCSYCYKCCGWWCCYCYCYSYGRTGRRGYY 36600
Indonesia-88065      TRRCCTMRCYRTATAGATGYYRYKRRWAYRRCCCCYKCCGWWCCYCYCYSYGRTGRRGYY 36600
                     :**   ** *    .    ****     ***.    * . *  .    *** *  * .**

Bhutan-09015         YYTAGTTTSKRRARGYRRGYTTRMYGMKRTAARYRRYRMRYGCRTWAAWSCRYTKWKTGR 36660
Bhutan-09024         YYTAGTTTSKRRARGYRRGYTTRMYGMKRTAARYRRYRMRYGCRTWAAWSCRYTKWKTGR 36660
Bhutan-09027         YYTAGTTTSKRRARGYRRGYTTRMYGMKRTAARYRGTGMRYGCRTWAAWSCRYTKWKTGR 36660
Bhutan-09030         YYTAGTTTSKRRARGYRRGYTTRMYGMKRTAARYRRYRMRYGCATWAAWSCRYTKWKTGR 36660
Bhutan-09005         CCKTKGKYGGAGARRCARKYKYGMTKMGRTAGGYGACACATKGGWARRTGMRCCTAKYRR 36660
Indonesia-88035      CCKTKGKYGGAGRRRCARKYKYGMTKAGRYAGGYGACACATKGGWARRTGMRCCTAKYRR 36660
Indonesia-88045      CCKTKGKYGGAGRRRCARKYKYGMTKMGRYRGGYGACACATKGGWARRTGMRCCTAKYRR 36660
Indonesia-88065      CCKTKGKYGGAGRRRCARKYKYGMTKMGRYRGGYGACACATKGGWARRTGMRCCTAKYRR 36660
                       .:  . .    *   * *.  *    *  . *               . *  . *  *

Bhutan-09015         YYRRYCYCTGTMYGTYYYCRAWKRAKCRYYGSYCRACMKYSYARTYRAAYTYYMYGTTKW 36720
Bhutan-09024         YYRRYCYCTGTMYGTYYYCRAWKRAKCRYYGSYCRACMKYSYARTYRAAYTYYMYGTTKW 36720
Bhutan-09027         YYRRYCYCTGTMYGTYYYCRAWKRRKCRYYGSYCRACMKYSYARTYRAAYTYYMYGTTKW 36720
Bhutan-09030         YYRRYCYCTGTMYGTYYYCAAWKRAKCRYYGSYCRACMKYSYARTYRAAYTYYMYGTTKW 36720
Bhutan-09005         YTRATYCCYRCMCAYYYCYRAATRAGMRYYCGYTARAAKYSTWRWYRGRYGYYCYKYYGA 36720
Indonesia-88035      YTRATYCCYRCMCAYYYCYRRATRAGMRYYCGYTARAAKYSTWRWYRGRYGYYCYKYYGA 36720
Indonesia-88045      YTRATYCCYRCMCAYYYCYRRATRAGMRYYCGYTARAAKYSTWRWYRGRYGYYCYKYYGA 36720
Indonesia-88065      YTRATYCYYRCMCAYYYCYRRATRAGMRYYCGYTARAAKYSTWRWYRGRYGYYCYKYYGA 36720
                     * *        * . **     .*   *** .*   . ***  * **. * ** *

Bhutan-09015         GKYATATRMYYYYSRRGCRGARYMRAYWMRCYYWMRKGMWWCCAAATRYCRCWGAGRWYY 36780
Bhutan-09024         GKYAYATRMYYYYSRRGCRGARYMRAYWMRCYYWMRKGMWWCCAAATRYCRCWGAGRWYY 36780
Bhutan-09027         GKYATATRMYYYYSRRGCRGARYMRAYWMRCYYWMRKGMWWCCAAATRYCRCWGAGRWYY 36780
Bhutan-09030         GKYATATRMYYYYSRRGCRGARYMRAYWMRCYYWMRKGMWWCCAAATRYCRCWGAGRWYY 36780
Bhutan-09005         ATYMCGCGCCCTTSARRYRAGACARWCWMAMTCTCRKCCATAAWRRYATYRYWCRRGWTY 36780
Indonesia-88035      RTYMCGCGCCCTTSARRYRAGACARWCWMAMTCTCRKCCATAAWRRYATYRYWCRRGWTY 36780
Indonesia-88045      RTYMCGCGCCCTTSARRYRAGACARWCWMAMTCTCRKCCATAAWRRYATYRYWCRRGWTY 36780
Indonesia-88065      RTYMCGCGCCCTTSARRYRAGACARWCWMAMTCTCRKCCATAAWRRYATYRYWCRRGWTY 36780
                      .*  .       * *  *..   *  **      **    ..       * *    * *

Bhutan-09015         SYAMMWGKSCWYAARWMRKRRMYMYRYAARYWRRSSKTSGMCCGSTYYRSGRTYYAYMGT 36840
Bhutan-09024         SYAMMWGKSCWYAARWMRKRRMYMYRYAARYWRRSSKTSGMCCGCTYYRSGRTYYAYMGT 36840
Bhutan-09027         SYAMMWGKGCATAARWMRKRRMYMYRYAARYWRRSSKTSGMCCGCTYYRSGRTYYAYMGT 36840
Bhutan-09030         SYAMMWGKSCWYAARWMRKRRMYMYRYAARYWRRSSKTSGMCCGSTYYRSGRTYYAYMGT 36840
Bhutan-09005         CCRAAAKKSYKCGCAWMRGRRMCMYRCRMGCWRAGCGCGRCTYACGCYASKACCTRCCKY 36840
Indonesia-88035      CCRAAAKKSYKCGCAWMRGRRMCMYRCRMGCWRAGCGCGRCTYACGCYASKACCTRCCKY 36840
Indonesia-88045      CCRAAAKKSYKCGCAWMRGRRMCMYRCRMGCWRAGCGCGRCTYACGCYASKACCTRCCKY 36840
Indonesia-88065      CCRAAAKKSYKCGCAWMRGRRMCMYRCRMGCWRAGCGCGRCTYACGCYASKACCTRCCKY 36840
                     .      *.   .. *** *** ***     ** ..  .    ..  * *

Bhutan-09015         YRRRCTYKRYSCRGMGRRMTGACCGCCTGRSRGAYRTRYTGRRAGWRWRGTYRSYRMYAA 36900
Bhutan-09024         YRRRCTYKRYSCRGMGRRMTGACCGCCTGRSRKWYRTRYTGRRAGWRWRGTYRSYRMCAA 36900
Bhutan-09027         YRRRCTYKRYSCRGMGRRMTGACCGCCTGRSRGAYRTRYTGRRAGWRWRGTYRSYRMCAA 36900
Bhutan-09030         YRRRCTYKRYSCRGMGRRMTGACCGCCTGRSRGAYRTRYTGRRAGWRWRGTYRSYRMCAA 36900
Bhutan-09005         CAARYYTGRYSYATCKGGAYRWYYGMMAAGGAGAYGARYYRAGRRARWASWYASYACCAW 36900
Indonesia-88035      CAARYYTGRYSYATCKGGAYRWYYKMMAAGGAGAYGARYYRAGRRARWASWYASYACCAA 36900
Indonesia-88045      CAARYYTGRYSYATCKGGAYRWYYKMMAAGGAGAYGARYYRAGRRARWASWYASYACCRA 36900
Indonesia-88065      CAARYYTGRYSYATCKGGAYRWYYKMMAAGGAGAYGARYYRAGRRARWASWYASYACCAW 36900
                        *    ***                :. .   * :**       ** . * **
```

```
Bhutan-09015         AWCYRTKYGAKMRTAYYYTYYRRCRGTCCRKCACGARYCCYYMSKYTACATYRYMGGARC 36960
Bhutan-09024         AWCYRKKYGAKMRTAYYYTYYRRCRGTCCRKCACGARYCCYYMSKYTACATYRYMGGARC 36960
Bhutan-09027         AWCYRTKYGAKMRTAYYYTYYRRCRGTCCRKCACGARYCCYYMSKYTACATYRYMGGARC 36960
Bhutan-09030         AWCYRTKYGAKMRTAYYYTYYRRCRGTCCRKCACGARYCCYYMSKYTACATYRYMGGARC 36960
Bhutan-09005         GWMTATKYSRWYRKWYTCYYYAASGACTTRGYRYARATSYYTCSKCYMYTWCAYCSKCAM 36960
Indonesia-88035      RWMTAKKYSRWYRKWYTCYYYAASGACTTRGYRYARATSYYTCSKCYMYAACAYCCTCRM 36960
Indonesia-88045      RWMTAKKYSAWYRKWYTCYYYAASGACTTRGYRYARATSYYTCSKCYMYTACAYCCTCRM 36960
Indonesia-88065      RWMTAKKYSRWYRKWYTCYYYAASGACTTRGYRYARATSYYTCSKCYMYAWCAYCCTCRM 36960
                      *   .**.   *. *   **  . .   *    .   . *  **    :   *   .

Bhutan-09015         GACMGYTGYWKSMGRWAAKKYRGWYGRYCYSYSSSGYYTGCTTCGAGSWYSRMAGMATTY 37020
Bhutan-09024         GACMGYTGYWKSMGRWAATGYRGWYGRYCYSYSSSGYYTGCTTCGAGSWTSRMAGMATTY 37020
Bhutan-09027         GACMGYTGYWKSMGRWAAKKYRGWYGRYCYSYSSSGYYTGCKTCGAGSWYSRMAGMATTY 37020
Bhutan-09030         GACMGYTGYWKSMGRWAAKKYRGWYGRYCYSYSSSGYYTGCKTCGAGSWYSRMAGMATTY 37020
Bhutan-09005         KRSMSTYSTTKSARGWRGGTTRCWCAAYMCCYSSSRYMYKYTYYSRAGWTSRATRAMYCC 37020
Indonesia-88035      KRSMSTYSTTKSARGWRGGTTRCWCAAYMCCYSCGRYMYKYTYYSRAGWTSRATRAMYCC 37020
Indonesia-88045      KRSMGTYSTTKSAAGWRGGTTRCWCAAYMCCYSCGRYMYKYTYYSRAGWTSRATRAMYCC 37020
Indonesia-88065      KRSMSTYSTTKSARGWRGGTTRCWCAAYMCCYSSSRYMYKYTYYSRAGWTSRATRAMYCC 37020
                       .*.  .  **   * .   * * . *  .**.. *    .  . ..* ** :

Bhutan-09015         YYARATRMKMMWWTYRGSYYTSCWATKGYRTMRWRYRRTRARTTWTRRRSRMYYYYKYRY 37080
Bhutan-09024         YYARATRMKMMWWTYRGSYYTSCWATKGYRTMRWRYRRTRARTTWTRRRSRMYYYYKYRY 37080
Bhutan-09027         YYARATRMKMMWWTYRGSYYTSCWATKGYRTMRWRYRRTRARTTWTRRRSRMYYYYKYRY 37080
Bhutan-09030         YYARATRMKMMWWTYRGSYYTSCWATKGYRTMRWRYRRTRARTTWTRRRSRMYYYYKYRY 37080
Bhutan-09005         CTGGCGGCTAMATKCGTGTCYGGTGCGKTRKMGWGCAGCAMAYATYAARGGCCCTTGCGC 37080
Indonesia-88035      CTGGCGGCTAMATKCGTGTCYGGTGCGKTRKMGWGCAGCAMAYATYAARGGCCCTTGCGC 37080
Indonesia-88045      CTGGCGGCTAMATKCGTGTCYGGTGCGKTRKMGWGCAGCAMAYATYAARGGCCCTTGCGC 37080
Indonesia-88065      CTGGCGGCTAMATKCGTGTCYGGTGCGKTRKMGWGCAGCAMAYATYAARGGCCCTTGCGC 37080
                       . .   . *  .   .   .  .    *.* *         :    *.

Bhutan-09015         SACRGRYYTCTSRYACYTCYACTCTTWCAYYKMRYYKKGCTRACYRRKYRYWCKGSYTKK 37140
Bhutan-09024         SACRGRYYTCTSRYACYTCYACTCTTWCAYYKMRYYKKGCTRACYRAGYRYWCKGSYTKK 37140
Bhutan-09027         SACRGRYYTCTSRYACYTCYACTCTTWCAYYKMRYYKKGCTRACYRAGYRYWCKGSYTKK 37140
Bhutan-09030         SACRGRYYTCTSRYACYTCYACTCTTWCAYYKMRYYKKGCTRACCRAGYRYWCKGSYTKK 37140
Bhutan-09005         GRYRTGCTYMWSGTMSCYYYMYYYYWTYWYTKMRTYKKGCYGRYYGGKTACTYKTGTKGG 37140
Indonesia-88035      GACRTGCTTCTGGTACCTCTACTCYTTCATTTMRTYKKRYYGACYGGTTACTCKTGTTGG 37140
Indonesia-88045      GACRTGCTTCTGGTACCTCTACTCYTTCATTTMRTYKKRYYGACYGGTTACTCKTGTTGG 37140
Indonesia-88065      GRYRTGCTYMWSGTMSCYYYMYYYYWTYWYTKMRTYKKGCYGRYYGGKTACTYKTGTKGG 37140
                     .  *       .   .               .** ***               * . .

Bhutan-09015         CKKKKWCRARGYRYGWYKKAAAYAACGYAMYGWYRYYYAYRKSCRYMYKTCGCYTGMRAC 37200
Bhutan-09024         CKKKKWCRARRYRYGWYKKAAAYAAYGYAMYGWYRYYYAYRKSCRYMYKTCGCYTGMRAC 37200
Bhutan-09027         CKKKKWCRARGYRYGWYKKAAAYAAYGYAMYGWYRYYYAYRKSCRYMYKTCGCYTGMRAC 37200
Bhutan-09030         CKKKKWCRARGYRYGWYKKAAAYAAYGYAMYGWYRYYYAYRKSCRYMYKTCGCYTGCRAC 37200
Bhutan-09005         TGGTGTSRRGRYRYRWCKTWRGYMRYKYRAYRWYATTCACRGSSGYAYGCMRYYYRCRRM 37200
Indonesia-88035      TGGTGTSRRGGYRYRWCKTWRGYMRYKYRAYRWYATTCRCRGSSGYAYGCMRYYYRCRRM 37200
Indonesia-88045      TGGTGTSRRGRYRYRWCKTWRGYMRYKYRAYRWYATTCACRGSSGYAYGCMRYYYRCRRM 37200
Indonesia-88065      TGGTGTSRRGRYRYRWCKTWRGYMRYKYRAYRWYATTCRCRGSSGYAYGCMRYYYRCRRM 37200
                        .  .*   *** * *.  .*    *  * **      * *. * *     *   *

Bhutan-09015         YGRGRRTYYTKRYAYWYGCMTMTWCAMTWGWRMYWMTYYCGTKTRRYTMSMTCCYGSRGG 37260
Bhutan-09024         YGRGRRTYYTKRYAYWYGCMTMTWCAMTWGWRMYWMTYYCGTKTRRYTMSMTCCYGSRGG 37260
Bhutan-09027         YGRGRRTYYTKRYAYWYGCMTMTWCAMTWGWRMYWMTYYCGTKTRRYTMSMTCCYGSRGG 37260
Bhutan-09030         YGRGRRTYYTKRYAYWYGCMTMTWCAMTWGWRMYWMTYYCGTKTRRYTMSMTCCYGSRGG 37260
```

```
Bhutan-09005         YRGRRGYCTATGCWYWCRTMYCYWYMCCWRARMCWAATCMRCKYRGCCMKCYYYTRGGKS 37260
Indonesia-88035      YRGRRGYCTATGCWYWCRTMYCYWYMCCWRARMCWAATCMRCKYRGCCMKCYYYTRGGKS 37260
Indonesia-88045      YRGRRGYCTATGCWYWCRTMYCYWYMCCWRARMCWAATCMRCTCGGCCMKCYYYTRGGKS 37260
Indonesia-88065      YRGRRGYCTATGCWYWCRTMYCYWYMCCWRARMCWAATCMRCTCGGCCMKCYYYTRGGKS 37260
                     *   *    :.   **   *   *    *  ** * :     .     *.      .  .

Bhutan-09015         CMATARRRATRMWWMSRGAGMCRWSRGGCTRAYATAYCRTYYYWGYWYWRATWTRCKTWK 37320
Bhutan-09024         CMATARRRATRMWWMSRGMGMCRWSRGGCTRAYATAYCRTYYYWGYAYWRATWTRCKTWK 37320
Bhutan-09027         CMATARRRATRMWWMSRGMGMCRWSRGGCTRAYATAYCRTYYYWGYWYWRATWTRCKTWK 37320
Bhutan-09030         CMATARRRATRMWWMSRGARMCRWSRGGCTRAYATAYCRTYYYWGYWYWRATWTRCKTWK 37320
Bhutan-09005         YCRCRRGRWCACWWMGGGARRTAAGGARMARRCRCRTTGYCYTWSTWCTARYWYGYKWWT 37320
Indonesia-88035      YCRCAAGRWCACWWMGGRAGRTAAGGARMARRCRCRTTGYCYTWSYACTAGTWYRYKWWT 37320
Indonesia-88045      YCRCRRGRWCACWWMGGRARRTAAGGARMARRCRCRTTGYCYTWSYACTAGTWYRYKWWT 37320
Indonesia-88065      YCRCRRGRWCACWWMGGRARRTAAGGARMARRCRCRTTGYCYTWSTACTARYWYGYKWWT 37320
                            *    ***.        . .  :*          * *.       *   * *.

Bhutan-09015         YRKMYCYSRSRTTKGRTRYSRYCTRKYWRRWCRWTSATGGMMAACYTCKWWRSYAARRCA 37380
Bhutan-09024         YRKMYCYSRSRTTKGRTGCSRYCTRKYWRRWCRWTSATGGMMAACYTCKWWRSYAARRCA 37380
Bhutan-09027         YRKMYCYSRSRTTKGRTRYSRYCTRKYWRRWCRWTSATGGMMAACYTCKWWRSYAARRCA 37380
Bhutan-09030         YRKMYCYSRSRTTKGRTGCSRYCTRKYWRRWCRWTSATGGMMAACYTCKWWRSYAARRCA 37380
Bhutan-09005         CRKMTSCCRSAAATYGWACCACTCGGYTGRWYGTASRCGCAMMRYTWMGTWRGYWRRRYM 37380
Indonesia-88035      CRKMTSCCRSAAWKSGWAYSACTCGGYTGRWYGTWCRCRCAMMRYTWMGTWRSYWARRYM 37380
Indonesia-88045      CRKMTSCCRSAAWKSGWAYSACTCGGYTGRWYGTWCRCRCAMMRYTWMGTWRSYWARRYM 37380
Indonesia-88065      CRTATSCCRSAAATYGWACCACTCGGYTGRWYGTASRCGCAMMRYTWMGTWRGYWRRRYM 37380
                      *.  . .** : .     .      *  **    .     *        **.*  **

Bhutan-09015         CTGRSGCYGWSYYAKTTGGTSYSYYKRRYKMYAKRWTYTWWRKTWGRGSRYRRYMRACGK 37440
Bhutan-09024         CTGRSGCYGWSYYAKTTGGTSYSYYKRRYKMYAKRWTYTWWRKTWGRGSRYRRYMRACGK 37440
Bhutan-09027         CTGRSGCYGWSYYAKTTGGTSYSYYKRRYKMYAKRWTYTWWRKTWGRGSRYRRYMRACGK 37440
Bhutan-09030         CTGRSGCYGWSYYAKTTGGTSYSYYKRRYKMYAKRWTYTWWRKTWGRGSRYRRYMRACGK 37440
Bhutan-09005         MYSRSRMYKTSTTATGCRSYGCCTCAAGCGMTRSGTKTKAARTCWSRSGRCGRTAAWYKG 37440
Indonesia-88035      MYSRSGMYKTSTTRTGCRSYGCCCTTAGCGMTRSGTKTKAARTCWSRSGRCGRTAAWYKG 37440
Indonesia-88045      MYSRSGMYKTSTTRTGCRSYGCCCTTAGCGMTRSGTKTKAARTCWSRSGRCGRTAAWYKG 37440
Indonesia-88065      MYSRSRMYKTSTTATGCRSYGCCTCAAGCGMTRSGTKTKAARTCWSRSGRCGRTAAWYKG 37440
                       .**  *  *   .   . . .       *  .  . .  *. *.*..*  *

Bhutan-09015         GGGAAYSSRRMCRSYAYRTCGRKWRRRMAACWYYCARRRAMWYWRRYGRAAYRRWRGTYY 37500
Bhutan-09024         GGGAAYSSRRMCRSYAYRTCGRKWRRRMAACWYYCARRRAMWYWRRYGRAAYRRWRGTYY 37500
Bhutan-09027         GGGAAYSSRRMCRSYAYRTCGRKWRRRAAACWYYCARRRAMWYWRRYGRAAYRRWRGTYY 37500
Bhutan-09030         GGGAAYSSRRACRSYAYRTCGRKWRRRMAACWYYCARRRAMWYWRRYGRAAYRRWRGTYY 37500
Bhutan-09005         RATRGCSCAACAASTRCGAGSGTWRRACRWYWCCYRGGRMCAYTRATRGGGCAGAGRTTC 37500
Indonesia-88035      RATRGCSCAACAASTGCGAGSGTWRRACRWYWCCYRGGRMCAYTRATRGGGCAGAGRKTC 37500
Indonesia-88045      RATRGCSCAACAASTGCGAGSGTWRGACRWYWCCYRGGRMCAYTRATRGGGCAGAGRTTC 37500
Indonesia-88065      RATRGCSCAACAASTGCGAGSGTWRRACRWYWCCYRGGRMCAYTRATRGGGCAGAGRKTC 37500
                      .  . *.   . *    : . .**      *      *   * *    ..      .

Bhutan-09015         TYWRGWSYRYAAKMYARTARYYWSYSCYSAKSRCSYTAWTCSSSMYAMRYATKRAYRGWT 37560
Bhutan-09024         TYWRGWSYRYAAKMYARTARYYWSYSCYSAKSRCSYTAWTCSSSMYAMRYATKRAYRGTT 37560
Bhutan-09027         TYWRGWSYRYAAKMYARTARYYWSYSCYSAKSRCSYTAWTCSSSMYAMRYATKRAYRGWT 37560
Bhutan-09030         TYWRGWSYRYAAKMYARTARYYWSYSCYSAKSRCSYTAWTCSSSMYAMRYATKRAYRGWT 37560
Bhutan-09005         KCTGSASTATRMGMTMAYMGYYWCYCMTSWTCGYCTYRAYYCSSMTRCRTWKTGRCGATY 37560
Indonesia-88035      KCTGGWSTATRMGMTMAYMGYYWCYCMTSWTCGYGTYRAYYCSSMTRCRTWKTGRCGGTY 37560
Indonesia-88045      KCTGGWSTATRMGMTMAYMGYYWCYCMTSWTCGYGTYRAYYCSSMTRCRTWKTGRCGGTY 37560
Indonesia-88065      KCTGSASTATRMGMTMAYMGYYWCYCMTSWTCGYCTYRAYYCSSMTRCRTWKTGRCGGTY 37560
```

```
                     .   . *      *      ***.*.  * ..         .***   *  ..    .

Bhutan-09015         TWCTWRMYSYAKWSKWTCRYAYYYTRKTKRMKRYGYRMYWYYMTYYRYYAYGYRWAGTGR 37620
Bhutan-09024         TWCTWRMYSYAKWSKWTCRYAYYYTRKTKRMKRYGYRMYWYYMTYYRYYAYGYRTAGTGR 37620
Bhutan-09027         TWCTWRMYSYAKWSKWTCRYAYYYTRKTKRMKRYGYRMYWYYMTYYRYYAYGYRTAGTGR 37620
Bhutan-09030         TWCTWRMYSYAKWSKWTCRYAYYYTRKTKRMKRYGYRMYWYYMTYYRYYAYGYRTAGTGR 37620
Bhutan-09005         YATYWAATGTGGTCTAYTATWYCCCRTAGRAGRCKYRCYTTTMWTYATCRTATGTAGTRR 37620
Indonesia-88035      YATYWAATGTGGTCTAYTATWYCCCRTAGRAGRCKYRCYTTTMWTYATCRTATGWTTTRR 37620
Indonesia-88045      YATYWAATGTGGTCTAYTATWYCCCRTAGRAGRCKYRCYTTTMWTYATCRTATGWTMTRR 37620
Indonesia-88065      YATYWAATGTGGTCTAYTATWYCCCRTAGRAGRCKYRCYTTTMWTYATCRTATGWATWRR 37620
                         *   . .  ..      *   *.: *  *  ** *   *  *     .   :   *

Bhutan-09015         TGRKMAWRRTGRCSCCRRARGTGCCATCTYAWYRTGAGACAYKKYRRCGAYWCCYGRRYM 37680
Bhutan-09024         TGRKMAWRRTGRCSCCRRARGTGCCATCTYAWYRTGAGACAYKKYRRCGAYWCCYGRRYM 37680
Bhutan-09027         TGRKMAWRRTGRCSCCRRARGTGCCATCTYAWYRTGAGACAYKKYRRCGAYWCCYGRRYM 37680
Bhutan-09030         TGRKMAWRRTGRCSCCRRARGTGCCATCTYAWYRTGAGACAYKKYRRCGAYWCCYGRRYM 37680
Bhutan-09005         WKRKMMARRYCAYCYYGAWGSWKYMWYYKCWWYAWKGTWSGYTKYGGMRMCTTTYRGAYC 37680
Indonesia-88035      WKRKMMARRYCAYCYYGAWGSWKYCAYYKCWWYAWKGTWSGYTKYGGMRMCTTTYRGAYC 37680
Indonesia-88045      WKRKMMARRYCAYCYYGAWGSWKYMWYYKCWWYAWGGTWSGYTKYGGMRMCTTTYRGAYC 37680
Indonesia-88065      WKRKMMARRYCAYCYYGAWGSWKYMWYYKCWWYAWGGTWSGYTKYGGMRMCTTTYRGAYC 37680
                       ***  **    .      .       .  **   .  ..*.**         *   *

Bhutan-09015         RSTAGGWACYYTWRGYYGMRCGYAGTYRKGTMYSGGGGCGYWKRMARMMCWWTRRTTMYW 37740
Bhutan-09024         RSTAGGWACYYTWRGYYGMRCGYAGTYRKGTMYSGGGGCGYWKRMARMMCWWTRRTTMYW 37740
Bhutan-09027         RRTAGGWACYYTWRGYYGMRCGYAGTYRKGTMYSGGGGCGYWKRMAACMCWWTRRTTMYW 37740
Bhutan-09030         RSTAGGWACYYTWRGYYGMRCGYAGTYRKGTMYSGGGGCGYWKRMARMMCWWTRRTTMYW 37740
Bhutan-09005         ACKRSRWRYTCGARTTTRARYKTRKWYATKYACCRRRKYRYWKRAWGMASWTWGGKKCTA 37740
Indonesia-88035      ACTAGGARCTCGAGTTTGAACGTAGTTATGTACCGGGGCGCATAAAGMACTTTGGTTCTA 37740
Indonesia-88045      ACTAGGARCTCGAGTTTGAACGTAGTTATGTACCGGGGCGCATAAAGMACTTTGGTTCTA 37740
Indonesia-88065      ACKRSRWRYTCGARTTTRARYKTRKWYATKYACCRRRKYRYWKRAWGMASWTWGGKKCTA 37740
                       . .                       .    .        .      .     ..

Bhutan-09015         ARYGTTCSYYYRRTACTWMSMRWTRYMWCGAGGCGRWAGGYRRCMTAAAKYRARYTGTTY 37800
Bhutan-09024         ARYGTTCSYYYRRTACTWMSMRWTRYMWCGAGGCGRWAGGYRRCMTAAAKYRARYTGTTY 37800
Bhutan-09027         ARYGTTCSYYYRRTACTWMSMRWTRYMWCGAGGCGRWAGGYRRCMTAAAKYRARYTGTTY 37800
Bhutan-09030         ARYGTTCSYYYRRTACTWMSMRWTRYMWCGAGGCGRWAGGYRRCMTAAAKYRARYTGTTY 37800
Bhutan-09005         RGTRYYMSYCCRRKRYKAMSMGWYGYMWMGARRYRGAWRAYRGYCYRWAGTACACGTAAT 37800
Indonesia-88035      AGTGTTCCCCCGGTRCTAMSAGTTGTATCGAGGCGGAWRAYGGYCYRWRKTACACGTAAT 37800
Indonesia-88045      AGTGTTCCCCCGGTRCTAMSAGTTGTATCRRGGCGGAWRAYGGYCYRWRKTACACGTAAT 37800
Indonesia-88065      RGTRYYMSYCCRRKRYKAMCMGWYGYMWMGARRYRGAWRAYRGYCYRWAKTACACGTAAT 37800
                            .     .  . *.                   .*           .    ::

Bhutan-09015         RYCYSYRYGRYYSAAAGGCCATRRYMYWMCGYRTWYYTRRAGYYCMYWCKRACKGKWYYS 37860
Bhutan-09024         RYCYSYRYRGTYSAAAGGCCATRRYMYWMCGYRTWYYTRRAGYYCMYWCKRACKRKWYYS 37860
Bhutan-09027         RYCYSYRYRGTYSAAAGGCCATRRYMYWMCGYRTWYYTRRAGYYCMYWCKRACKRKWYYS 37860
Bhutan-09030         RYCYSYRYRRYYSAAAGGCCATRRYMYWMCGYRTWYYTRRAGYYCMYWCKRACKRKWYYS 37860
Bhutan-09005         GYYTSTRYGGTTCARRRKCYRKAGTCTACTTCGWTCTKGGWRTTTATAMKARYGAKTCTC 37860
Indonesia-88035      GYYTSTRYGGTTCRRRRKMYRKAGTCTACTTCGWTCTKGGWRTTTATAMKARYGAKTCTC 37860
Indonesia-88045      GYYTSTRYGGTTCRRRRKMYRKAGTCTACTTCGWTCTKGGWRTTTATAMKARYGAKTCTC 37860
Indonesia-88065      GYYTSTRYGGTTCRRRRKMYRKAGTCTACTTCGWTCTKGGWRTTTATAMKARYGAKTCTC 37860
                      *  * **    .        .               .           *     *   .

Bhutan-09015         GTRAGAKKGGGYAYMGAYKGWYGGATRWYRRRGTAYYACWGTKAMYTWWKYYRYYYTGAA 37920
Bhutan-09024         GTRAGAKKGGGYAYMGAYKGWYGGATRWYRRRGTAYYACWGTKAMYTWWKYYRYYYTGAA 37920
```

```
Bhutan-09027          GTRAGAKKGGGYAYMGAYKGWYGGATRWYRRRGTAYYACWGTKAMYTWWKYYRYYYTGAA 37920
Bhutan-09030          GTRAGAKKGGGYAYMGAYKGWYGGATRWYRRRGTAYYACWGTKAMYTWWKYYRYYYTGAA 37920
Bhutan-09005          RWARKRTTKRRCGTCKMTKKWCKAGTAATGRGRAMCYCGARWGWCCYWTGTYRCCTCCGM 37920
Indonesia-88035       RWARKRTTKRRCGYMGMTKKWCKAGWRATGRGRAMCYCGARWGWCCYWTGTYRCCTCCGM 37920
Indonesia-88045       RWARKRTTKRRCGYMGMTKKWCKAGWRATGRGRAMCYCGAGWGWCCYWTGTYRCCTCCGM 37920
Indonesia-88065       RWARKRTTKRRCGTCKMTKKWCKAGTAATGRGRAMCYCGARWGWCCYWTGTYRCCTCCGM 37920
                            ..    .     * *  ..     *  :  *.         *   **     .

Bhutan-09015          ATTYMRYSCTCRCKCYAGAATYKYAGTYYYAACARWYMWKMRKTCMKCYKWWRYYAATCC 37980
Bhutan-09024          ATTYMRYSCTCRCKCYAGAATYKYAGTYYYAACARWYMWKMRKTCMKCYKWWRYYAATCC 37980
Bhutan-09027          ATTYMRYSCTCRCKCYAGAATYKYAGTYYYAACARWYMWKMRKTCMKCYKWWRYYAATCC 37980
Bhutan-09030          ATTYMRCCCTCRCKCYAGAATYKYAGTYYYAACARWYMWKMRTTCMKCYKWWRYYAATCC 37980
Bhutan-09005          WWKTARCCTYMRATYYRRRWKCGCGTKCYCRWYMAWYATKCRTYYMGMYKTTGCCRRWYY 37980
Indonesia-88035       WWKTARCCTYMRATYYRRRWKCGCGTKCYCRWYMAWYATKCRTYYMGMYKTTGCCRRWYY 37980
Indonesia-88045       WWKTARCCTYMRATYYRRRWKCGCGTKCYCRWYMAWYATKCRTYCMGMYKTTGCCRRWYY 37980
Indonesia-88065       WWKTARCCTYMRATYYRRRWKCGCGTKCYCRWYMAWYATKCRTYYMGMYKTTGCCRRWYY 37980
                        .  * .   *.. *    .   . . *      **  * *.  *  **

Bhutan-09015          CRRCYSRGTGYTAARGGRTGKRCYWAYAYRRRMTWARWMCAKYCKYYGMMRYRRGYATGT 38040
Bhutan-09024          CRRSYSRGTGYTAARGGRTGKRCYWAYAYRRRMTWARWMCAKYCKYYGMMRYRRGYATGT 38040
Bhutan-09027          CRRSYSRGTGYTAARGGRTGKRCYWAYAYRRRMTWAAWMCAKYCKYYGMMRYRRGYATGT 38040
Bhutan-09030          CRRSYSRGTGYTAARGGRTGKRCYWAYAYRRRMTWARWMCAKYCKYYGMMRYRRGYATGT 38040
Bhutan-09005          YRACYCARYKYYMRRTGRYRKRSCAWTRTGRGCKAAAWMSCTTSGCTKCMGTGARCGWAK 38040
Indonesia-88035       YRACYCARYKYYMRRTSRYRKRSCAWTRTGRGCKARAWMSCTTSGCTKCMGTGARCGWAK 38040
Indonesia-88045       YRACYCARYKYYMRRTGRYRKRSCAWTRTGRGCGARAWMSCTTSGCTKCMGTGARCGWAK 38040
Indonesia-88065       YRACYCARYKYYMRRTGRYRKRSCAWTRTGRGCKARAWMSCTTSGCTKCMGTGARCGWAK 38040
                       * .*.    *   * .*  **.       *      **... .     *      . ..

Bhutan-09015          MMGGRYYGGMTTTGYGWGGTYYAYSSGRWCRYWKTCRWWYYRRGAAYRTWRYGCAWYTRR 38100
Bhutan-09024          MMGGRYYGGMTTTGYGWGGTYYAYSSGRWCRYWKTCRWWYYRRGAAYRTWRYGCAWYTRR 38100
Bhutan-09027          MMGGRYYGGMTTTGYGWGGTYYAYSSGRWCRYWKTCRWWYYRRGAAYRTWRYGCAWYTRR 38100
Bhutan-09030          MMGGRYYGGMTTTGYGWGGTYYAYSSGRWCRYWKTCRWWYYRRGAAYRTWRYGCAWYTRR 38100
Bhutan-09005          AMARACTKKCCCWATRWAKYYCRTSSRATYGCTGWMRTTTTGRKTCCAYAGTRMRTCYGR 38100
Indonesia-88035       AMARACTKKCCCWATRWAKYYCRTSSRATYGCTGWMRTTTTGRKTCCAYAGTGCRTCYGR 38100
Indonesia-88045       AMARACTKKCCCWATRWAKYYCRTSSRATYGCTGWMRTTTTGRKTCCAYAGTRMRTCYGR 38100
Indonesia-88065       AMARACTKKCCCWATRWAKYYCRTSSRATYGCTGWMRTTTTGRKTCCAYAGTRMRTCYGR 38100
                       *.          .  *.  *   **          *     * :.             *

Bhutan-09015          AGAARTCGRRSKYYYRACYCRACRYCRWRRWACACRTMYYTRKRRYCCTAKSSRCSYRYM 38160
Bhutan-09024          AGAARTCGRRSKYYYRACYCRACRYCRWRRWACACRTMYYTRKRRYCCTAKSSRCSYRYM 38160
Bhutan-09027          AGAARTCGRRSKCCYRACYCRACRYCRWRRWACACRTMYYTRKRRYCCTAKSSRCSYRYM 38160
Bhutan-09030          AGAARTCGRRSKCCYRACYCRACRYCRWRRWACACRTMYYTRKRRYCCTAKSSRCSYRYM 38160
Bhutan-09005          RRRRAYMRGGGGTTCGGYCYRGYATYRWRRTMCACRYAYYWRKGGTMMYMGGSGYRYGTM 38160
Indonesia-88035       RRRRAYMRGGGGTTCGGYCYRGYATYRWRRTMSMYRYAYCWRKGGYMMYMGGSGYRYGTM 38160
Indonesia-88045       RRRRAYMRGGGGTTCGGYCYRGYATYRWRRTMSMYRYAYYWRKGGCMMYMGGSGYRYGTM 38160
Indonesia-88065       RRRRAYMRGGGGTTCGGYCYRGYATYRWRRTCGCTRYAYYWRKGGTMMYMGGSGYRYGTM 38160
                                .     .   *.    ****     *  *  **        .*   *  *

Bhutan-09015          RWYYWWGRRAKRWGYTRRYSRYRGYRCRCGGTAGSGGTRWTRRYTYRYSYARGRTRYRSS 38220
Bhutan-09024          RWYYWWGRRAKRWGYTRRYSRYRGYRCRCGGTAGSGGTRWTRRYTYRYSYARGRTRYRSS 38220
Bhutan-09027          RWYYWWGRRAKRWGYTRRYSRYRRYRCRCGGTAGSGGTRWTRRYTYRYSYARGRTRYRSS 38220
Bhutan-09030          RWYYWWGRRAKRWGYTRRYSRYRRYRCRCGGTAGSGGTRWTRRYTYRYSYARGRTRYRSS 38220
Bhutan-09005          ATYTTTRRAMKGWRYYGGYSGYARCRTGYRRYWSSKTAAWWARYKTGYSTGARRAACGGG 38220
Indonesia-88035       ATYTTTRRAMKGWRYYGGYSGYAGCRTGYRRYWSSKTAAWWAACKTGYSTGARRAACGGG 38220
```

```
Indonesia-88045      ATYTTTRRAMKGWRYYGGYSGYAGCRTGYRRYWSSKTAAWWARYKTGYSTGARRAACGGG 38220
Indonesia-88065      ATYTTTRRAMKGWRYYGGYSGYAGCRTGYRRYWSSKTAAWWARYKTGYSTGARRAACGGG 38220
                       *    *  * * *   ** *   *       .*  : *    .  ** .  *:   ..

Bhutan-09015         KYGACRRGTWARCTYTYCYYKWWSYGYKKYTYYRMCGATTSMRCKYCYAYMMWWMRYKMT 38280
Bhutan-09024         KYGACRRGTWARCTYTYCYYKWWSYGYKKYTYYRMCGATTSMRCKYCYAYMMWWMRYKMT 38280
Bhutan-09027         KYGACRRGTWARCTYTYCYYKWWSYGYKKYTYYRMCGATTSMRCKYCYAYMMWWMRYKMT 38280
Bhutan-09030         KYGACRRGTWARCTYTYCYYKWWSYGYKKYTYYRMCGATTSMRCKYCYAYMMWWMRYKMT 38280
Bhutan-09005         KTARYRRACAWGAYTKCYCTGATSCRCTGYWYTGMGKRWYCCGSTYSYWYCMWAMGCKCC 38280
Indonesia-88035      KTARYRRACAWGAYTKCYCTGATSCRCTGYWYTGMGKRWYCCGSTYSYWYCMWAMGCKCC 38280
Indonesia-88045      KTARYRRACAWGAYTKCYCTGATSCGCTGYWYTGMGKRWYCCGSTYSYWYCMWAMGCKCC 38280
Indonesia-88065      KTARYRRACAWGAYTKCYCTGATSCRCTGYWYTGMGGGWYCCGSTYSYWYCMWAMGCKCC 38280
                     * .  **.    .  .       *   . * *  *     .  ..*.* * ** *  *

Bhutan-09015         GTAMYKAACYAGGMGRGYTYAWGYYRMYTAACCWSYCCSYRRYTYSGAYGWARWWARACM 38340
Bhutan-09024         GTAMYKAACYAGGMGRGYTYAWGYYRMYTAACCWSYCCSYRRYTYSGAYGWARWWARACM 38340
Bhutan-09027         GTAMYKAAYYAGGMGRGYTYAWGYYRMYTAACCWSYCCSYRRYTYGGAYGWARWWARACM 38340
Bhutan-09030         GTAMYKAACYAGGMGRGYTYAWGYYRMYTAACCWSYCCSYRRYTYGGAYGWARWWARACM 38340
Bhutan-09005         CARAYKWRYTRRKAGASTYYGAKCCGCYYWRAYTGYYYSCRATYTCCTCRTMRAARRRTM 38340
Indonesia-88035      CARAYKWRYTGRGAGASTYYGAKCCGCYYWRAYTGYYYSCRATYTCCTCRTMRAARRRTM 38340
Indonesia-88045      CARAYKWRYTRRGAGASTYYGAKCCGCYYWRAYTGYYYSCRATYTSCTCRTMRAARRRTM 38340
Indonesia-88065      CARAYKWRYTRRGATASTYYGAKCCGCYYWRAYTGYYYSCRATYTSCTCRTMRAARRRTM 38340
                      :  **          .  *.      *   .  .*  * *      :    *   *  *

Bhutan-09015         RGYMRYACGYRTMMGTCGMRSTCTRYATARTGRWRWTCGWAWYAGGCTGTGYYCWGAMKW 38400
Bhutan-09024         RGYMRYACGYRTMMGTCGMRSYCTRYATARTGRWRWTCGWAWYAGGCTGTGYYCWGAMKW 38400
Bhutan-09027         RGYMRYACGYRTMMGTCGMRSYCTRYATARTGRWRWTCGWAWYAGGCTGTGYYCWGAMKW 38400
Bhutan-09030         RGYMRYACGYRTMMGTCGMRSYCTRYATARTGRWRWTCGWAWYAGGCTGTGYYCWGAMKW 38400
Bhutan-09005         AKTMGCRYSYGYMMSYMAAASTMYACRWMRWAAWAWYYSWWTCMRGYYACGCCMAGMMKW 38400
Indonesia-88035      AKTMGCRYSYGYMMSTMAAASTCYACRWMRWRAWAWYYCWWTCMRRYYAYRCCMARMMKW 38400
Indonesia-88045      AKTMGCRYSYGYMMSTMAAASTMYACRWMRWRAWAWYYCWWTCMRRYYAYRCCMARMMKW 38400
Indonesia-88065      AKTMGCRYSYGYMMSYMAAASTMYACRWMRWRRWAWYYSWWTCMRGYYACGCCMAGMMKW 38400
                        *    .*  **.  .  *        *   * *   *        .        ***

Bhutan-09015         YWRSRAAYGGMWGRWRRCAYMWTRCYGGSKYRTYMYWWYMTRWGARTMYWTMAGWTTYWR 38460
Bhutan-09024         YWRSRAATGGMWGRWRRCAYMWTRCYGGSKYRTYMYWWYMTRWGARTMYWTMAGWTTYWR 38460
Bhutan-09027         YWRSRAATGGMWGRWRRCAYMWTRCYGGSKYRTYMYWWYMTRWGARTMYWTMAGWTTYWR 38460
Bhutan-09030         YWRSRAAYGGMWGRWRRCAYMWTRCYGGSKYRTYMYWWYMTRWGARTMYWTMAGWTTYWR 38460
Bhutan-09005         CWASAMRYRRMTRRTGASMYMWYRMYCRCKYRWYCCWATAWRTKRGYAYAWARRAYKYAR 38460
Indonesia-88035      CAACAAATGGATGATGACCCAATGCCCGCGCGATCCAATATATGAGTACATAAGATTTAA 38460
Indonesia-88045      CAACAAATGGATGATGACCCAATGCCCGCGCGATCCAATATATGAGTACATAAGATTTAA 38460
Indonesia-88065      CWASAMRYGGMTRRTGASMYMWYRMYCRCKYRWYCCWATAWRWKRGTAYAWARRAYKYAR 38460
                        .             .          .                           .

Bhutan-09015         TTGAGGWKYMTYKWSRWKKRYTYRAWAACWYTGTYCTYKYTYYCTCCTYKTMWMYGYARG 38520
Bhutan-09024         TTGAGGWKYMTYKWSRWKKRYTYRAWAACWYTGTYCTYKYTYYCTCCTYKTMWMYGYARG 38520
Bhutan-09027         TTGAGGWKYMTYKWSRWKKRYTYRAWAACWYTGTYCTYKYTYYCTCCTYKTMWMYGYARG 38520
Bhutan-09030         TTGAGGWKYMTYKWSRWKKRYTYRAWAACAYTGTYCTYKYTYYCTCCTYKTMWMYGYARG 38520
Bhutan-09005         WTAGAATGCCYCGWSRTGKRYWCGWAWRYACKRYCYYTGCKYCMCYYWCTYCAMYRCRAR 38520
Indonesia-88035      TYRGGGTGCCTCGTSRTGTGTTCGAAAACACTGTCCTTGCTTCMTCCTCTTCACCGCAAG 38520
Indonesia-88045      TYRGGGTGCCTCGWSRTGTGTTCGAAAACACTGTCCTTGCTTCMTCCTCTTCACCGCAAG 38520
Indonesia-88065      WTAGRRTGCCYCGWSRTGKRYWCGWAWRYACKRYCYYTGCKYCMYYYWCTYCAMYRCRAR 38520
                        .          **  .            .        .        .
```

```
Bhutan-09015          RCRYWSAKYYCYRRAKTCCMRCKWYRYYTCMRTCASCYYRKATATTARRRWYKYCYGAGY 38580
Bhutan-09024          RCRYWSAKYYYYRRAKTCCMRCKWYRYYTCMRTCASCYYRKATATTARRRWYKYCYGAGY 38580
Bhutan-09027          RCRYWSAKYYYYRRAKTCCMRCKWYRYYTCMRTCASCYYRKATATTARRRWYKYCYGAGY 38580
Bhutan-09030          RCRYWSAKYYYYRRAKTCCMRCKWYRYYTCMRTCASCYYRKATATTARRRWYKYCYGAGY 38580
Bhutan-09005          GSRYWCRTYYTCGGRGAGYARYGWYGYCYYARYCGGYCTATWWWYYRARATCGTCYSWRY 38580
Indonesia-88035       GCACTCATTTTCGGAGAGCAACGATGTCTCAAYCGGCCTATATAYTAAGATCGTCYGAGY 38580
Indonesia-88045       GCACTCATTTTCGGAGAGCAACGATGTCTCAAYCGGCCTATATAYTAAGATCGTCYGAGY 38580
Indonesia-88065       GSRYWCRTYYTCGGRGAGYARYGWYGYCYYARCYGGYCTATWWWYYRARATCGTYYSARY 38580
                       .   . .        :                 ..    .              *.  *

Bhutan-09015          YGYMYSYGGCTMWCGYYYAAYTRWRYRAYCRYCSKKATGYYYRKYGGRAYYRRCKGGRYY 38640
Bhutan-09024          YGYMYSYGGCTMWCGYYYAAYTRWRYRAYCRYCSKKATGYYYRKYGGGAYYRRCKGGRYY 38640
Bhutan-09027          YGYMYSYGGCTMWCGYYYAAYTRWRYRAYCRYCSKKATGYYYRKYGRGAYYRRCKGGRYY 38640
Bhutan-09030          YGYMYSYGGCTMWCGYYYAAYTRWRYRAYCRYCSKKATGYYYRKYGGGAYYRRCKGGRYY 38640
Bhutan-09005          YRCMTGCRRYYATYACYCRRYYRWACAGTGGYYGKKRWRCYYAGTRGGWYYRGMTSRRCY 38640
Indonesia-88035       YRCMTGCAAYYATYRCYCRRYYRWACAGTGGYYGKKRWRCYYAGTRGGWYYRGMTSRRCY 38640
Indonesia-88045       YRCMTGCAAYYATYRCYCRRYYRWACAGTGGYYGKKRWRCYYAGTRGGWYYRGMTSRRCY 38640
Indonesia-88065       YRCMTGCRRYYATYRCYCRRYYRWACAGTGGYYGKKRWRCYYAGTRGGWYYRGMTSRRCY 38640
                      *  * .          *   * **   .   * .**    **       ***  .. * *

Bhutan-09015          RCCGAACYGRYTRSYWTRARARCTCCRTTGRRRGAWRRKKYRMGRYRTGGYKRYYRCGKM 38700
Bhutan-09024          RCCGAACYGRYTRSYWTRARARCTCCRTTGRRRGAWRRKKYRMGRYRTGGYKRYYRCGKM 38700
Bhutan-09027          RCCGAACYGRYTRSYWTRARARCTCCRTTGRRRGAWRRKKYRMGRYRTGGYKRYYRCGKM 38700
Bhutan-09030          RCCGAACYGRYTRSYWTRARARCTCCRTTGRRRGAWRRKKYRMGRYRTGGYKRYYRCGKM 38700
Bhutan-09005          RYYSRGSCRGYCASYTCGRGWRTCTARCYSGAASGWGAGTYRCRGYAGKACKRYTGYRKA 38700
Indonesia-88035       RYYSRGSCRGYCASYTCGRGWRTCTARCYSGAASGWGAGTYRCRGYAGKACKRYTGCGKA 38700
Indonesia-88045       RYYSRGSCRGYCASYTCGRGWRTCTARCYSGAACGWGAGTYRCRGYAGKRCKRYTGCGKA 38700
Indonesia-88065       RYYSRGSCRGYCASYTCGRGWRTCTARCYSGAASGWGAGTYRCRGYAGKRCKRYTGYRKA 38700
                      *  . ..   *  **      *   .* .    .*   .**   *     ***    *

Bhutan-09015          WKMRRCSCSCTRSRMRYACRKACMRSTYAAAGACAMWYSCKWYMRCAYTWMSTYRSMRGA 38760
Bhutan-09024          WKMRRCSCSCTRSRMRYACRKACMRSTYAAAGACAMWYSCKWYMRCAYTWMSTYRSMRGA 38760
Bhutan-09027          WKMRRCSCSCTRSRMRYACRKACMRSTYAAAGACAMWYSCKWYMRCAYTWMSTYRSMRGA 38760
Bhutan-09030          WKMRRCSCSCTRSRMRYACRKACMRSTYAAAGACAMWYSCKWYMRCAYTWMSTYRSMRGA 38760
Bhutan-09005          TGCAGYSMGYGRGRMGCRYRTWSCGYKTWMWRWSRCATCYKWCMAARYYAAGYYAGMGKA 38760
Indonesia-88035       TGCAGCCCGYGAGRCGCACGTTCCGCTTAAAGACACATCCGACMAARYYAAGYYAGMGGR 38760
Indonesia-88045       TGCAGCCCGYGAGRCGCACGTTCCGCTTAAAGACACATCCGACMAARYYAAGYYAGMGGA 38760
Indonesia-88065       TGCAGYSMGYGRGRMGCRYRTWSCGYKTWMWRWSRCATCYKWCMAARYYAAGYYAGMGKA 38760
                            . .   .*      . .   .      .    .    * . *   . * .*

Bhutan-09015          YWYYSTARATTASMCACMTRYKKYARYMTYWRGRRWYYGATTTTTTGYRCRGAYCWRYRR 38820
Bhutan-09024          YWYYSTARATTAGMCACMTRYKKYARYMTYWRGRRWYYGATTTTTTGYRCRGAYCWRYRR 38820
Bhutan-09027          YWYYSTARATTACMCACMTRYKKYARYMTYWRGRRWYYGATTTTTTGYRCRGAYCWRYRR 38820
Bhutan-09030          YWYYSTARATTAGMCACMTRYKKYARYMTYWRGRRWYYGATTTTTTGYRCRGAYCTRYRR 38820
Bhutan-09005          TAMCGYWGRYYGCMAGTMYRCGTCRGCAYCAGKRRATTRRYYWWWWRYRYGAGTSTGTGA 38820
Indonesia-88035       TAMCGYWGRYYGCMAGTRYRCGTCRGCAYCAGKRRATTRRYYWWWWRYRYGARTSTGTGA 38820
Indonesia-88045       TAMCGYWGRYYGCMAGTRYRCGTCRGCAYCAGKRRATTRRYYWWWWRYRYGARTSTGTGA 38820
Indonesia-88065       TAMCGYWGRYYGCMAGTRYRCGTCRGCAYCAGKRRATTRRYYWWWWRYRYGRRTSTGTGA 38820
                          .      . *..   *  .          **            **     .

Bhutan-09015          YYYTYKARYCRCGAAYWSWMAAAGGYYRTCRCTRCRYRCCRKMMKSRYTYWGYCKYWYTA 38880
Bhutan-09024          YYYTYKARYCRCGAAYWSWMAAAGGYYRTCRCTRCRYRCCRKMMKSRYTYWGYCKYWYTA 38880
Bhutan-09027          YYYTYKARYCRCGAAYWSWMAAAGGYYRTCRCTRCRYRCCRKMMKSRYTYWGYCKYWYTA 38880
Bhutan-09030          YYYTYKARYCRCGAAYWSWMAAAGGYYRTCRCTRCRYRCCRKMMKSRYTYWGYCKYWYTA 38880
```

```
Bhutan-09005        TTTWTKWRYCGSRRGCTSAMRRRRRTCRYYAMWASATGYAAKCMGCAYWTARCTTTATYG 38880
Indonesia-88035     TTTWTKWRYMGSRRGCTGAMRRRRRTCRYYAMWASATGYAAKCMGCAYWTARCTTTATYG 38880
Indonesia-88045     TTTWTKWRYMGSRRGCTGAMRRRRRTCRYYAMWASATGYAAKCMGCAYWTAGCTTTATYG 38880
Indonesia-88065     TTTTTKWRYMGSRRGCTSAMRRRRRTCRYYAMWASATGYAAKCMGCAYWTARCTTTATYG 38880
                         * **  .  .  . *       *      .    . * * . *      .    .

Bhutan-09015        WGYSGCAAAACCCAAGGSWWRGAGTAACYCYYMYKRWTACARWAKRYGRYCCYKAGARGY 38940
Bhutan-09024        WGYSGCAAAACCCAAGGSWWRGAGTAACYCYYMYKRWTACARWAKRYGRYCCYKAGARGY 38940
Bhutan-09027        WGYSGCAAARCCCAAGGSWWRGAGTAACYCYYMYKRWTACARWAKRYGRYCCYKAGARGY 38940
Bhutan-09030        WGYSGCAAARCCCAAGGSWWRGAGTAACYCYYMYKRWTACARWAKRYGRYCCYKAGARGY 38940
Bhutan-09005        WRCSSYRCCAGAYRWRSSWWRKRRYMMMYYCCMCKRAYGYCGARGAYKAYYMCGRRRRRY 38940
Indonesia-88035     ARCSSYRCCAGAYRWRSSWWRKRRYMMMYYCCMCKRAYGYCGARGAYKAYYMCGRARRRY 38940
Indonesia-88045     WRCSSYRCCAGAYRWRSSWWRKRRYMMMYYCCMCKRAYGYCGARGAYKAYYMCGRARRRY 38940
Indonesia-88065     WRCSSYRCCAGAYRWRSSWWAKRRYMMMYYCCMCKRAYGYCGARGAYKAYYMCGRRRRRY 38940
                       *.  .. .    .***        *   * **  . .     *  *       * *

Bhutan-09015        RGASWKGYYRWWGAYAAYSSCGCRGYRCCYTTMRRMSTACYASSWRARWCSWTWTYMCKG 39000
Bhutan-09024        RGASWRGYYRWWGAYAAYSSYRCRGYRCCYTTMRRMSTACYASSWRARWCKYTWTYMCKG 39000
Bhutan-09027        RGASWGGYYRWWGAYAAYSSYRCRGYRCCYTTMRRMSTACYASSWRARWCKYTWTYMCKG 39000
Bhutan-09030        RGASWKGYYRWWGAYAAYSSYRCRGYRCCYTTMRRMSTACYASSWRARWCKYTWTYMCKG 39000
Bhutan-09005        ARMCWGKYYRWWRRTARYSGCGYGSCGYYYWYMAAMSYRSTRSSARRRASGTYTYYAYTK 39000
Indonesia-88035     ARMCWMKYYRWWRRTARYSGCGYGSCGYYYWYMAAMSYRSTRSSARRRASGTYTYYAYTK 39000
Indonesia-88045     ARMCWTKYYRWWRRTWRYSGCGYGSCGYYYWYMAAMSYRCTRSSARRRASGTYTYYAYTK 39000
Indonesia-88065     ARMCWAKYYRWWRRTYRYSGCGYGSCGYYTTTAAAMSYRSTRSSARRRASGTYTYYAYTK 39000
                       .*  *****     **.    .          **  .  ** * * .     *  .

Bhutan-09015        WRGYAMRGRMGYRTWKRYGCWYAYAAYRGYYWRWYTGCYASCGATYWACAYTRYTTTGCR 39060
Bhutan-09024        WRGYAMRGRMGYRTWKRYGCWYAYAAYRGYYWRWYTGCYASCGATYWACAYTRYTTTGCR 39060
Bhutan-09027        WRGYAMRGRMGYRTTKRYGCWYAYAAYRGYYWRWYTGCYASCGATYWACAYTRYTTTGCR 39060
Bhutan-09030        WRGYAMRGRMGYRTWKRYGCWYAYAAYRGYYWRWYTGCYASCGATYWACAYTRYTTTGCR 39060
Bhutan-09005        TRATWAGKGWRTRWWTACRTWCWTWTCRRCCTRWYYRSTMSGACGCWRYMCYGCYYYRAA 39060
Indonesia-88035     TRATWAGKGWRTRWTTACRTWCWTWTCRRCCTRWYYRSTMSGACGCWRYMCYGCYYYRAA 39060
Indonesia-88045     TRATWAGKGWRTRWTTACRTWCWTWTCRRCCTRWYYRSTMSGACGCWRYMCYGCYYYRAA 39060
Indonesia-88065     TRATWAGKGWRTRWWTACRTWCWTWTCRRCCTRWYYRSTMSGACGCWRYMCYGCYYYRAA 39060
                     *.         *  .    *    : *    ***  .  * ..  *           .

Bhutan-09015        KYTAGWGRMYGYWYMCWYYKYCRYKKYCCRRYRSRATCRGSTGTAYTCWKWWCTYGYYYM 39120
Bhutan-09024        KYTAGWGRMYGYWYMCWYYKYCRYKKYCCRRYRSRATCRGSTGTAYTCWKWWCTYGYYYM 39120
Bhutan-09027        KYTAGWGAATGYWYMCWYYKYCRYKKYCCRRYRSRATCRGSTGTAYTCWKWWCTYGYYYM 39120
Bhutan-09030        KYTAGWGRATGYWYMCWYYKYCRYKKYCCRRYRSRATCRGSTGTAYTCWKWWCTYGYYYM 39120
Bhutan-09005        TTWRSAAGCCRTTTAYWTCGCYACTTTATRGYRSRMKYRSCGKYWTGTWKTTMYYRYTYM 39120
Indonesia-88035     TTWRSARGCCRTTTAYWTCGCYACTTTATRGYRSRMKYRSCGKYWTGTWKTTMYYRYTYM 39120
Indonesia-88045     TTWRSARGCCRTTTAYWTCGCYACTTTATRGYRSRMKYRSCGKYWTGTWKTTMYYRYTYM 39120
Indonesia-88065     TTWRSARGCCRTTTAYWTCGCYACTTTATRGYGGRATYRSCGKYWTGTWKTTMYYRYTYM 39120
                    .   .           *       .. . * * .* . *..       **    * * **

Bhutan-09015        KGCGGTAWGATCACTTKGTAYMSTWKGGWYRCARMYWWRRRAAARRWYWGATYYTYCCTY 39180
Bhutan-09024        KGCGGTAWGATCACTTKGTAYMSTWKGGWYACARMYWWRRRAAARRWYWGATYYTYCCTY 39180
Bhutan-09027        KGCGGTAWGATCACTTKGWRYMSTWKGGWYRCARMYWWRRRAAARRWYWGATYYTYCCTY 39180
Bhutan-09030        KGCGGTAWGATCACTTKGWRYMSTWKGGWYRCARMYWWRRRAAARRWYWGATYYTYCCTY 39180
Bhutan-09005        GTYARCGWCRYYRMYWKAWAYMSWTKKATCAYWACYWAAGARGRGGTTWARYCTCYTYYY 39180
Indonesia-88035     GTYARCGWCRYYRMYWKATAYMSWTKKATCAYWACYWAAGARGRGGTTWARYCTCYTYYY 39180
Indonesia-88045     GTYARCGWCRYYRMYWKATAYMSWTKKATCAYWACYWAAGARGRGGTTWARYCTCYTYYY 39180
Indonesia-88065     GTYARCGWCRYYRMYWKATAYMSWTKKATCAYWACYWAAGARGRGGTTWARYCTCYTYYY 39180
```

```
                        .  .*        *.  ***  * .       **     .     *.     *   *

Bhutan-09015         KATCGGRYGCRRKSWTWRYYGYSRGWRAATRRTCYWSRRMGCAAWAYTCAKGTGYWWCRR 39240
Bhutan-09024         KATCGGRYGCRRKSWTWRYYGYSRGWRAATRRTCYWSRRMGCAAWAYTCAKGTGYWWCRR 39240
Bhutan-09027         KATCGGRYGCRRKSWTWRYYGYSRGWRAATRRTCYWSRRMGCAAWAYTCAKGTGYWWCRR 39240
Bhutan-09030         KATCGGRYGCRRKSWTWRYYKYSRGWRAATRRTCYWSRRMGCAAWAYTCAKGTGYWWCRR 39240
Bhutan-09005         TGGAARRYATAGKGTYAGTCKCGAAWRMRWGGASTTGAGATGGRARTKYRTRYRCWTYGA 39240
Indonesia-88035      TGGAGRRYATAGKGTYAGTCKCGAAWRMRWGGASTTGAGATGGRARTKYRTRYRCWTYGA 39240
Indonesia-88045      TGGAGRRYATAGKGTYAGTCKCGAAWRMRWGGASTTGAGATGGRARTKYRTRYRCWTYGA 39240
Indonesia-88065      TGGAARRYATAGKGTYAGTCKCGAAWRMRWGGASTTGAGATGGRARTKYRTRYRCWTYGA 39240
                     .. .. **.   *.        . .**     :.  .     .    .  .    *

Bhutan-09015         YAYKAATCCYCYCYGTGCWMYYACTWKRRRCTWYYYRKYATRAAGGYYRGGGGKARYAWY 39300
Bhutan-09024         YAYKAATCCYCYCYGTGCWMYYACTWKRRRCTWYYYRKYATRAAGGYYRGGGGKARYAWY 39300
Bhutan-09027         YAYKAATCCYCYCYGTGCWMYYACTWKRRRCTWYYYRKYATRAAGGYYRGGGGKARYAWY 39300
Bhutan-09030         YAYKAATCCYCYCYGTGCWMYYACTWKRRRCTWYYYRKYATRAAGGYYRGGGGKARYAWY 39300
Bhutan-09005         YMTTWRYYYCMTYTRYRYTAYCRYCTTARRYGWYTCRTYRYRGRRRTYRSKCKWMGTGWT 39300
Indonesia-88035      YMTTWRYYYCMTYTRYRYTAYCRYCTTARRYGWYTCRTYRYRGRRRTYRSKCKWMGTGWT 39300
Indonesia-88045      YMTTWRYYYCMTYTRYRYTAYCRYCTTARRYGWYTCGTCRYRGRRRTYRSKCKWMGTGWT 39300
Indonesia-88065      YMTTWRYYYCMTYTRYRYTAYCRYCTTARRYGWYTCRTYRYRGRRRTYRSKCKWMGTGWT 39300
                     *  .                *     . **  **   .   *.    **.       .*

Bhutan-09015         TRYYMYRGRCKTACARGTYRRGRGRRGWMCCRWWRGRMASGWYCYYGGCRGYGGRWWRRG 39360
Bhutan-09024         TRYYMYRGRCKTACARGTYRRGRGRRGWMCCRWWRGRMASGWYYYYGGCRGYGGRTTGRG 39360
Bhutan-09027         TRYYMYRGRCKTACARGTYRRGAGRRGWMCCRWWRGRMASGWYYYYGGCRGYGGRWWRRG 39360
Bhutan-09030         TRYYMYRGRCKTACARGTYRRGRGRRGWMCCRWWRGRMASGWYYYYGGCRGYGGRWWRRG 39360
Bhutan-09005         KRCCMTRRRYGYGYRGAYYRAAGRGRRTAYYRWWRRRCMGSWYCTCSRYRCTAARTTGAG 39360
Indonesia-88035      KRCCMTRRRYGYGTRGAYYRAAGRGRRTAYYRWWRRRCMGSWYCTCSRYRCTAARTTGAR 39360
Indonesia-88045      KRCCMTRRRYGYGYGGAYYRAAGRGRRTAYYRWWRRRCMGSATCTCSRYRCTAARTTGAR 39360
Indonesia-88065      KRCCMTRRRYGYGYRGAYYRAAGRGRRTAYYRWWRRRCMGSWYCTCSRYRCTAARTTGAG 39360
                     .*  * * *   .   . ** .   *     **** *  ..     .  *  ..*

Bhutan-09015         TRTKYCRTGCRTTCGGTMSGWRCTWGSSGYRRYARCTRRCYGMACRAKGSRSAYRGTKRR 39420
Bhutan-09024         TRTKYCRTGCRTTCGGTMSGWRCTWGSSGYRRYARCTRRCYGMACRAKGSRSAYRGTKRR 39420
Bhutan-09027         TRTKYCRTGCRTTCGGTMSGWRCTWGSSGYRAYARCTRACCGMACRAKGSRSAYRGTKRR 39420
Bhutan-09030         TRTKYCRTGCRTTCGGTMSGWRCTWGSSGYRRYARCTRRCCGMACRAKGSRSAYRGTKRR 39420
Bhutan-09005         CAYTYYRYRSRYCYRKWACRWGMYTTSSGYAACWGYCRAYCRMWSGMKRSRSGTARYTGG 39420
Indonesia-88035      CAYTYYRYRSRYCYRKWACRWGMYTTSSRYAACWGYCRAYCAMWSGMKRSRSGTARYTGG 39420
Indonesia-88045      CAYTYYRCASGCCYRKWACRWGMYTTSSRYAACWGYCRAYCAMWSGMKRSRSGTARYTGG 39420
Indonesia-88065      CACTYYRCASGCCYRKWACRWGMYTTSSGYAACWGYCRAYCAMWSGMKRSRSGTARYTGG 39420
                        .* *  .        . *     ** *       *    * .  * ***.    .

Bhutan-09015         CARAAATYGATSSRCCYWTCTKMRYYKTWGKTCCAGAACRMMYCWYRTYRGKRKGYATYR 39480
Bhutan-09024         CARAAATYGATSSRCCYWTCTKMRYYKTWGKTCCAGAACRMMYCWYRTYRGKRKGYATYR 39480
Bhutan-09027         CARAAATYGATSSRCCYWTCTKMRYYKTWGKTCCAGAACRMMYCWYRTYRGKRKGYATYR 39480
Bhutan-09030         CARAAATYGATSSRCCYWTCTKMRYYKTWGKTCCAGAACRMMYCWYRTYRGKRKGYATYR 39480
Bhutan-09005         MRGRCRKCARKCCKYMTWYTGKMRYYTKWKKCYMMRWMYRMMYATYAYYGSKAGRCWKCA 39480
Indonesia-88035      MRGRCRKCARKCCKYMTWYTGKMRYYTTWKKCYMMRWMCRMMYATYAYYGSKAGRCWKCA 39480
Indonesia-88045      MRGRCRKCARKCCKYMTWYTGKMRYYTTWKKCYMMRWMCRMMYATYAYYGSKAGRCWKCA 39480
Indonesia-88065      MRGRCRKCARKCCKYMTWYTGKMRYYTKWKKCYMMRWMYRMMYATYAYYGSKAGRCWKCA 39480
                         . . . ...:   *   *****..* *        ****. *  * .*     .

Bhutan-09015         YRCCAAGYACTRYKGCGATRWWAKACYKYTCRGRGRTGRRAACGCCTCGYCRRGYWRKRR 39540
Bhutan-09024         YRCCAAGYACTRYKGCGATRTWAKACYKYTCRGRGRTGRRAACGCCTCGYCRRGYWRKRR 39540
```

```
Bhutan-09027            YRCCAAGYACTRYKGCGATRWWAKACYKYTCRGRGRTGRRAACGCCTCGYCRRGYWRTRR 39540
Bhutan-09030            YRCCAAGYACTRYKGCGATRWWAKACYKYTCRGRGRTGRRAACGCCTCGYCRRGYWRTRR 39540
Bhutan-09005            CAMYRMCTRYYGTKRYKWWGAWMKMMTGCTYARGRGYAARWRTTYYKSATSARRYTRGAG 39540
Indonesia-88035         CAMYRMCTRYYGTKAYKTAGAWMKMMTGCTYARGRGYAARWRTTYYKSATSARRYTRGAG 39540
Indonesia-88045         CAMYRMCTRYYGTKRYKTAGAWMKMMTGCYTARGRGYAARWRTTYYKSATSARRYTRGAG 39540
Indonesia-88065         CAMYRMCTRYYGTKRYKTAGAWMKMMTGCTYARGRGYAARWRTTYYKSATSARRYTRGAG 39540
                                     *       * *             . *      ... . * * *

Bhutan-09015            YYACYRWAWKRWCYWTYARTYSKGGYTYKGRCGCCCTCTTGCKCYARYSYYYTCACKCYA 39600
Bhutan-09024            YYACYRWAWKRWCYWTYARTYSKGGYTYKGRCGCCCTCTTGCKCYARYSYYYTCACKCYA 39600
Bhutan-09027            YYACYRWAWKRWCYWTYARTYSKGGYTYKGRCGCCCTCTTGCKCYARYSYYYTCACKCYA 39600
Bhutan-09030            YYACYRWAWKRWCYWTYARTYSKGGYTYKGRCGCCCTCTTGCKCYARYSYYYTCACKCYA 39600
Bhutan-09005            CYGYYRWRWTGAMYWKYWRWTSTKKYYTGRRGKYYGYYYYAAGTTRATCYYCGSWSKYTA 39600
Indonesia-88035         CYGYYRWRWTGAMYWKYWRWTSTKKYYTGRRGGCYGYYYYAAGTTRATCYYCGSWSKYTR 39600
Indonesia-88045         CYGYYRWRWTGAMYWKYWRWTSTKKYYTGRRGGCYGYYYYAAGTTRATCYYCGSWSKCTR 39600
Indonesia-88065         CYGYYRWRWTGAMYWKYWRWTSTKKYYTGRRGKYYGYYYYAAGTTRATCYYCGSWSKYTR 39600
                         *. *** *.   **.* *  *.  *    *         ..      .**  . .*

Bhutan-09015            YYRRRRCAGSSRMSSACTCWYCYRRRMRKTGCRTYATYRYYMRTCGCGCTTCYYYWGCTT 39660
Bhutan-09024            YYRRRRCAGSCRMSSACTCWYCYRRRMRKTGCRTYATYRYYMRTCGCGCTTCYYYWGCTT 39660
Bhutan-09027            YYRRRRSAGSSRMSSACTCWYCYRRRMRKTGCRTYATYRYYMRTCGCGCTTCYYYWGCTT 39660
Bhutan-09030            YYRRRRSAGSSRMSSACTCWYCYRRRMRKTGCRTYATYRYYMRTCGCGCTTCYYYWGCTT 39660
Bhutan-09005            TYGGAACGRGSGCGSRMYMWYTYGGAARGYRYAYTMCYATTMRGYKYSYYYYTCYWRYYT 39660
Indonesia-88035         TYGGAACGGGSGCGSRMYMWYTCGGAAAGYRCATTMCCATTAAGCGCGCTTCTCYTRYYW 39660
Indonesia-88045         TYGGAACGGGSGCGSRMYMWYTCGGAAAGYRCATTMCCATTAAGCGCGCTTCTCYTGYYW 39660
Indonesia-88065         TYGGAACGRGSGCGSRMYMWYTYGGAARGYRYAYTMCYATTMRGYKYSYYYYTCYWRYYT 39660
                         *    .. ..  .*    **                          .      *

Bhutan-09015            ASSRYYKYGWCAACTCMRTARKGGKGTTYYMGYMRGTYSYGCAGRAAAAARSWGSRRYAG 39720
Bhutan-09024            ASSRYYKYGWCAACTCMRTARKGGKGTTYYMGYMRGTYSYGCAGRAAAAARSWGSRRYAG 39720
Bhutan-09027            ASSRYYKTGWCAACTCMRTARKGGKGTTYYMGYMRGTYSYGCAGRAAAAARSWGSRRYAG 39720
Bhutan-09030            ASSRYYKYGWCAACTCMRTARKGGKGTTYYMGYMRGTYSYGCAGRAAAAARSWGSRRYAG 39720
Bhutan-09005            WGSRTTTCRWSRRTYYARKRAGRRKAGCTTCSYMRRYCSYKYRTAMWRRRRSTRGAGCRK 39720
Indonesia-88035         AGCGTTTCRWCAATTYARKRAGRRKAGCTTCSYMRRYCSYKYRTAMWRRRRSTRGAGCRK 39720
Indonesia-88045         AGCGTTTCRWCAATTYARKRAGRRKAGCTTCSYMRRYCSYKYRTAMWRRRRSTRGAGCRK 39720
Indonesia-88065         WGSRTTTCRWSRRTYYARKRAGRRKAGCTTCSYMRRYCSYKYRTAMWRRRRSTRGAGCRK 39720
                         ..   .  *.      *.     *.     .***   **          **  .

Bhutan-09015            RRCRTYYTTAARSYKGAGRYRATCTRYARSRGCYRCYYYCCYKRWRMYYYRRRTAATRR 39780
Bhutan-09024            RRCRTYYTTAARSYKGAGRYRATCTAYARSRGCYRCYYYCCYKRWRMYYYRRRTAATRR 39780
Bhutan-09027            RRCRTYYTTAARSYKGAGRYRATCTRYARSRGCYRCYYYCCYKRWRMYYYRRRTAATRR 39780
Bhutan-09030            RRCRTYYTTAARSYKGAGRYRATCTRYARSRGCYRCYYYCCYKRWRMYYYRRRTAATRR 39780
Bhutan-09005            RASACTCYYRRRSCTRCAGTAGWSYRTRAGRRYYGYYTTYYTKRWRMYCCYAAAKRWCGG 39780
Indonesia-88035         RASACTCYYRRRSCTRCAGTAGWSYRTRAGRRYYGYYTTYYTKRWRMYCCYAAAKRWCGG 39780
Indonesia-88045         RASACTCYYRRRSCTRCAGTAGWSYATRAGRRYYGYYTTYYTKRWRMYCCYAAAKRWCGG 39780
Indonesia-88065         RASACTCYYRRRSCTRCAGTAGWSYRTRAGRRYYGYYTTYYTKRWRMYCCYAAAKRWCGG 39780
                        * .        ** . ..   . .     .*  *  *     ******  *   .

Bhutan-09015            AYRKSYAYYTCCYRMWYWYYCKKWCCRKWRWGCSWGCRCTTACYTYAYGCMYTTTKYAGC 39840
Bhutan-09024            AYRKSYAYYTCCYRMWYWYCCKKWCCRKWRWGCSWGCGCTTACYTYAYGCMYKTTKYAGC 39840
Bhutan-09027            AYRKSYAYYTCCYRMWYWYYCKKWCCRKWRWGCSAGCGCTTACYTYAYGCMYTTTKYASC 39840
Bhutan-09030            AYRKSYAYYTCCYRMWYWYCCKKWCCRKWRWGCSAGCGCTTACYTYAYGCMYTTTKYASC 39840
Bhutan-09005            CTATCCWYYWYYYRMWYTYCYGTAMTRKWATGCSAGCGTCCMMYCYWCKTCCTWYKYMCT 39840
Indonesia-88035         CTATCCWYYWYYYRMWYTYCYGTAMTRKWATGTSAGMGTCCMMYCYWCKTCCTWYKYMCT 39840
```

```
Indonesia-88045      CTATCCWYYYWYYYRMWYTYCYGTAMTRKWATGCSATCGTCCMMYCYWCKTCCKWYKYMCT 39840
Indonesia-88065      CTATCCWYYYWYYYRMWYTYCYGTAMTRKWATKCSARCGTCCMMYCYWCKTCCTWYKYMCT 39840
                     .  ..  **   ***** *   .   ***    *         * *      .  **

Bhutan-09015         RCTSWAYRYSSAYYYMYWRYCTRRCASTMYMATRRWSCRYSYRSYRMKCCTRTATARRGT 39900
Bhutan-09024         RCTSWAYRYSSAYYYMYWRYCTRRCASTMYMATRRWSCRYSYRSYRMKCCTRTATARRGT 39900
Bhutan-09027         RCTSWAYRYSSAYYYMYWRYCTRRCASTMYMATRRWSCRYSYRSYRMKCCTRTATARRGT 39900
Bhutan-09030         RCTSWAYRYSSAYYYMYWRYCTRRCASTMYMATRRWSCRYSYRSYRMKCCTRTATARRGT 39900
Bhutan-09005         AYYSWMYGTGGRYTTCTAGYYYAAYRSYCYAWWAGACTAYGYACCRMKYSGGYWKMRGRC 39900
Indonesia-88035      ATYCWMYGTGGRYTTCTAGYYYAAYRSYCYAWWAGACTAYGYACCRMKYSGGYWKMRGRY 39900
Indonesia-88045      ATYSWMYGTGGRYTTCTAGYYYAAYRSYCYAWWAGACTAYGYACCRMKYSGGYWKMRGRT 39900
Indonesia-88065      AYYSWMYGTGGRYTTCTAGYYYAAYRSYCYAWWAGACTAYGYACCRMKYSGGYWKMRGRC 39900
                        .* *  .. *      *      *  *      .  *.* . *** .    . *

Bhutan-09015         TTGWTTYRTGWYTTACYYGCTCCCGATYRCARYYWRWYWYKRWKAMRKRTGRARMCMMMW 39960
Bhutan-09024         TTGTTTYRTGWYTTACYYGCTCCCGATYRCARYYWRWYWYKRWKAMRKRTGRARMCMMMW 39960
Bhutan-09027         TTGTTTYRTGWYTTACYYGCTCCCGATYRCARYYWRWYWYKRWKAMRKRTGRARMCMMMW 39960
Bhutan-09030         TTGTTTYRTGWYTTACYYGCTCCCGATYRCARYYWRWYWYKRWKAMRKRTGRARMCMMMW 39960
Bhutan-09005         CCAWCCYAARTCYCWYCCRYWYYMRRCCASWATCTAATATGRWGWCATRYSRGRCMMMMT 39960
Indonesia-88035      YYRTCCYAARTCYCWYCCRYWYYMRRCCASWATCTAATATGRWGWCATRYSRGRCMMMMT 39960
Indonesia-88045      TTGTCCYAARTCYCWYCCRYWYYMRRCCASWATCTAATATGRWGWCATRYSRGRCMMMMT 39960
Indonesia-88065      CCATCCYAARTCYCWYCCRYWYYMRRCCASWATCTAATATGRWGWCATRYSRGRCMMMMT 39960
                           * :                    .           **    .* .*.*  ***

Bhutan-09015         KMYRSRCGMGSRRGKSWGRRCCRCYWKGRRTRWGTARARCYKTAATMRTTYKSKAWWYKY 40020
Bhutan-09024         KMYRSRCGMGSRRGKSWGRRCCRCYWKGRRTRWGTARARCYKTAATMRTTYKSKAWWYKY 40020
Bhutan-09027         KMYRSRYGMGSRRGKSWGRRCCRCYWKGRRTRWGTARARCYKTAATMRTTYKSKAWWYKY 40020
Bhutan-09030         KMYRSRYGMGSRRGKSWGRRCCRCYWKGRRTRWGTARARCYKTAATMRTTYKSKAWWYKY 40020
Bhutan-09005         KMYACGYRCKGGRAKCWKAGYYAYTATRRRYRTRACRRGCCKTGGKCRWYYKGTWWWTKC 40020
Indonesia-88035      KMYACGYRCKGGRAKCWKAGYYAYTATRRRYRTRACRRGTCKTGGKCATYCTGTATATTC 40020
Indonesia-88045      KMYACGYRCKGGRAKCWKAGYYAYTATRRRYRTRACRRGTCKKGGKCATYCTGTATATTC 40020
Indonesia-88065      KMYACGYRCKGGRAKCWKAGYYAYTATRRRYRTRACRRGCCKTGGKCRWYYKGTWWWTKC 40020
                     *** .     . *.*.*         . ** *  :.*    *....     ...    .

Bhutan-09015         RYYYGKMKMSYAYSRRYYGTKTTRRRMCRYTWKAGCCTTYSRWTYCCCSGYWCCTTRAYY 40080
Bhutan-09024         RYYYGKMKMSYAYSRRYYGTKTTRRRMCRYTWKAGCCTTYSRWTYCCCSGYWCCTTRAYY 40080
Bhutan-09027         RYYYGKMKMSYAYSRRYYGTKTTRRRMCRYTWKAGCCTTYSRWTYCCCSGYWCCTTRAYY 40080
Bhutan-09030         RYYYGKMKMSYAYSRRYYGTKTTRRRMCRYTWKAGCCTTYSRWTYCCCSGYWCCTTRAYY 40080
Bhutan-09005         RYCTRTCKACCMCCGRCYKYKWYRRRMMRTKTKRKYYKKTGAWYYSSMCATTYAYAGMTT 40080
Indonesia-88035      ACCTGTCGACCMYCGACTGTGTTAGACCATTTGAGCCTTTGAWYYSSMCATTYAYAGMTT 40080
Indonesia-88045      ACCTGTCGACCMYCGACTGTGTTAGACCATTTGAGCCTTTGAWYYSSMCATTYAYAGMTT 40080
Indonesia-88065      RYCTRTCKACCMCCGRCYKYKWYRRRMMRTKTKRKYYKKTGAWYYSSMCATTYAYAGMTT 40080
                          .   .   .                .      .. . * *.. ..   . :

Bhutan-09015         WSCWAMKRWAYYRRTWGWRWSCYCKGYRSAMYCRATMGMTTGYRMYRMTAYTGCTCKRWT 40140
Bhutan-09024         WSCWAMKRWAYYRRTWGWRWSCYCKGYRSAMYCRATMGMTTGYRMYRMTAYTGCTCKRWT 40140
Bhutan-09027         WSCWAMKRWAYYRRTWGWRWSCYCKGYRSAMYCRATMGMTTGYRMYRMTAYTGCTCKRWT 40140
Bhutan-09030         WSCWAMKRWAYYRRTWGWRWSCYCKGYRSAMYCRATMGMTTGYRMYRMTAYTGCTCKRWT 40140
Bhutan-09005         WSMARCKGARYYAGYTAAAAGSYMTRCRGMATYGCCCRMYCKCAAYRCYWTKRYWYGAAY 40140
Indonesia-88035      WSMARCKGARYYAGYTAAAAGSYMTACRGMATYGCCCRMYCKCAAYRCYWTKRTWYGAAY 40140
Indonesia-88045      WSMARCKGARYYAGYTAAAAGSYMTACRGMATYGCCCRMYCKCAAYRCYWTKRYWYGAAY 40140
Indonesia-88065      WSMARCKGARYYAGYTAAAAGSYMTACRGMATYGCCCRMYCKCAAYRCYWTKRYWYGAAY 40140
                     **    *   **    .   ..* .  *.     .   *      **    .
```

```
Bhutan-09015         CTTRKTYRYMSCMARKSCWYYRRCCRTGYAKRMYCCTYTATATYYRCTRYKYTGRRGGYA 40200
Bhutan-09024         CTTRKTYRYMSCMARKSCWYYRRCCRTGYAKRMYCCTYTATATYYRCTRYKYTGRRGGYA 40200
Bhutan-09027         CTTRKTYRYMSCMAATSCWYYAACCRTGYAKRMYCCTYTATATYYRCTRYKYTGRRGGYA 40200
Bhutan-09030         CTTRKTYRYMSCMARKSCWYYAACCRTGYAKRMYCCTYTATATYYRCTRYKYTGRRGGYA 40200
Bhutan-09005         SAARGYYATMGMAGGGGYWYCGGYMGTGYRGAATMCYTWRWMTYTRYYGCTTYRAGTCCR 40200
Indonesia-88035      SAARGYYATMGMAGGGGYWYCGGYMGYRCAGAATCYTTTRTAKCTGCTGCTTTGAGTCCA 40200
Indonesia-88045      SAARGYYATMGMAGGGGYWYCGGYMGYRCAGAATCYTTTRTAKCTGCTGCTTTGAGTCCR 40200
Indonesia-88065      SAARGYYATMGMAGGGGYWYCGGYMGTGYRGAATMCYTWRWMTYTRYYGCTTYRAGTCCA 40200
                     .::*  *  *.  .  . **                      .       .

Bhutan-09015         TCTATGRCCRATARGCRYRWCRYYYYGTTTTRTKAYGGMTSYCYARRCCYGRYYATWAAG 40260
Bhutan-09024         TMTATGRCCRATARGCRYRWCRYYYYGTTTTRTKAYGGMTSYCYARRCCYGRYYATWAAG 40260
Bhutan-09027         TCTATGRCCRATARGCRYRWCRYYYYGTTTTRTKAYGGMTSYCYARRCCYGRYYATWAAG 40260
Bhutan-09030         TCTATGRCCRATARGCRYRWCRYYYYGTTTTRTKAYGGMTSYCYARRCCYGRYYATWAAG 40260
Bhutan-09005         CCCGYGGYYGWYRARYACGAYRTCYTRYYYWRKKAYGGCKSYYCGAGTSCRRCWWYTMRR 40260
Indonesia-88035      CACGTAGYYGWYRARYACGAYRTCYTRYYYWRKKAYGGCKSYYCGAGCSCRRCWWYTMAG 40260
Indonesia-88045      CCCGTTGYYGWYRARYACGAYRTCYTRYYYWRKKAYGGCKSYYCGAGCSCRRCWWYTCAG 40260
Indonesia-88065      CTCGTAGYYGWYRARYACGAYRTCYTRYYYWRKKRCRRCKSYYCGAGYSCRRCWWYTMRR 40260
                        .                 *  *      *.*     .**  .   .  * :

Bhutan-09015         CYWGRMYYYRRGCCGMGCTYWYYRAGGTRCRRCCATCTRRWMCTYAAYWTGKWMAMSKSK 40320
Bhutan-09024         CYWGRMYYYRRGCCGMGCTYWYYRAGGTRCRRCCATCTRRWMCTYAAYWTGKTCAMSKSK 40320
Bhutan-09027         CYWGRMYYTARGCCGMGCTYWYYRAGGTRCRRCCATCTRRWMCTYAAYWTGKWMAMSKSK 40320
Bhutan-09030         CYWGRMYYYRRGCCGMGCTYWYYRAGGTRCRRCCATCTRRWMCTYAAYWTGKWMAMSKSK 40320
Bhutan-09005         YTWAGATYCGGRYTRCRYWCACYRCKKYAARGTCTCTCGGTMTWTRATAKSTAMGACGCK 40320
Indonesia-88035      CTWAGATYCGGGYTRCRYWCACYRCGGYAARGTYTCTCGGTMTWTRATAKSTAMGACGCK 40320
Indonesia-88045      CTWAGATYCGGGYTRCRYWCACYRCGGYAARGTCTCTCGGTMTWTRATAKSTAMGACGCK 40320
Indonesia-88065      YTWAGATYCGGRYTRCRYWCACYRCKKYAARGTCTCTCGGTMTWTRMTAKSTAMGACGCK 40320
                       *.   *              **.    .*   :      *       ...  . . .*

Bhutan-09015         AWGASGCYTAAGSYCKYAKYWMMRTKTKCYAKACAGRTWYYRCYYRCSKAAWTGAATGTC 40380
Bhutan-09024         AWGASGCYTAAGSYCKYAKYWMMRTKTKCYAKACAGRTWYYRCYYRCSKAAWTGAATGTC 40380
Bhutan-09027         AWGASGCYTAAGSYCKYAKYWMMRTKTKCYAKACAGRTWYYRCYYRCSKAAWTGAATGTC 40380
Bhutan-09030         AWGASGCYTAAGSYCKYAKYWMMRTKTKCYAKACAGRTWYYRCYYRCSKAAWTGAATGTC 40380
Bhutan-09005         CTRGCACCARAASCCKTMKYAAMAYGGKYTRGMYRKAWWCCGACTRCGTAAWWRRRYAAT 40380
Indonesia-88035      CTRGCAYCARWASCMKTAKYAAMAYGGKYTRGMYRKAWWCCGAYYGSGKRRWWRRRYAAT 40380
Indonesia-88045      CTRGCAYCARWASCMKTMKYAAAAYGGKYTRGMYRKAWWCCGAYYGSGKRRWWRRRYAAT 40380
Indonesia-88065      CTRGCACCARAASCMKTAKYAAMAYGGKYTRGMYRKAWWCCGACTRCGKAAWWRRRYAAT 40380
                     .  ...  :  .*  *  **       *          *   .   ...  *     .:

Bhutan-09015         TTGWRMRRTKWWRRAYCWRCYYKCAYGKYYTAYCGWGGTRKCACACRAWYKYRACTRWKY 40440
Bhutan-09024         TTGWRMRRTKWWRRAYCWRCYYKCAYGKYYTAYCGWGGTRKCACACRAWYKYRACTRWKY 40440
Bhutan-09027         TTGWRMRRTKWWRRAYCWRCYYKCAYGKYYTAYCGWGGTRKCACACRAWYKYRACTRWKY 40440
Bhutan-09030         TTGWRMRRTKWWRRAYCWRCYYKCAYGKYYTAYCGWGGTRKCACACRAWYKYRACTRWKY 40440
Bhutan-09005         YWGTRAGGWKTWGGRCGTAYTCTSMTSKYYTMYTGYRRGGTCWCMMAWATTCGATYRWKC 40440
Indonesia-88035      YWRTAAGGYTTAGGRCGTAYTCTCATGTCCWACYRTGRTGTYWYAMAWATTCGTCYRWKC 40440
Indonesia-88045      YWRTAAGGYTTAGGRCGTAYTCTCATGTCCWACCATGRTGTYWYAMAWATTCGTCYRWKC 40440
Indonesia-88065      YWGTRAGGWKTWGGRCGTAYTCTSMTSKYYTMYTGYRRGGTCWCMMAWATTCGATYRWKC 40440
                              .            ..  ..            .         .  :  ***

Bhutan-09015         RAYAWCTYSACTRTTCRRCYSAKMTCWWYCKMSYCWTSSWYCMCGAYAKTKWYRWSYYTR 40500
Bhutan-09024         RAYAWCTYSACTRTTCRRCYSAKMTCWWYCKMSYCWTSSWYCMCGAYAKTKWYRWSYYTR 40500
Bhutan-09027         RAYAWCTYSACTRTTCRRSYSAKMTCWWYCKMSYCWTSSWYCMCGAYAKTKWYRWSYYTR 40500
Bhutan-09030         RAYAWCTYSACTRTTCRRSYSAKMTCWWYCKMSYCWTSSWYCMCGAYAKTKWYRWSYYTR 40500
```

```
Bhutan-09005         ACTRTMWCSWYKRKYSGRSYSRGAYYTTYYKACYSTYGSWYCAYRMYAKYKACGTSTCCR 40500
Indonesia-88035      ACTRTMWCCWYKRKYSGRSYSRGAYYTTYYKACYSTYGSWYYMTRMYWKYGACGTGTCCA 40500
Indonesia-88045      ACTRTMWCSWYKRKYSGRSYSRGAYYTTYYKACYSTYGSWYYAYRMYWKYGACGTGTCCA 40500
Indonesia-88065      ACTRTMWCSWYKRKYSGRSYSRGAYYTTYYKACYSTYGSWYYAYRAYAKYKACGTSTCCR 40500
                      .      .  .*. . *.**       * * .*.  .***     * *      .

Bhutan-09015         RTRWRTCWRGKGYYTYYMAYMGWYKMAYCTAACTARAWCKRCRYYWRRYSRSAAGRTGCK 40560
Bhutan-09024         RTRWRTCWRGKGYYTTYMAYMGWYKMAYCTAACTARAWCKRCRYYWRRYSRSAAGRTGCK 40560
Bhutan-09027         RTRWRTCWRGKGYYTYYMAYMGWYKMAYCTAACTARAWCKRCRYYWRRYSRSAAGRTGCK 40560
Bhutan-09030         RTRWRTCWRGKGYYTTYMAYMGWYKMAYCTAACTARAWCKRCRYYWRRYSRSAAGRTGCK 40560
Bhutan-09005         RYRTAKCWRRTKCCWYCARYCRAYTARTYYARSYWAMMYKGYACYTRATSRSWRSRYKYK 40560
Indonesia-88035      GTATATYTAATTCCWTCAAYCRACTAATCTMACTAAAMCGGCACTTRATGGSARSRYKYK 40560
Indonesia-88045      GTATATYTAATTCCWTCAAYCRACTAATCTMACTAAAMCGGCACTTRATGGSARSRYKYK 40560
Indonesia-88065      RYRTAKCWRRTKCCWYCARYCRAYTARTTYAASYWAMMYKGYACYTRATSRSWRSRYKYK 40560
                          .    .        *    .       .             *  . *  .*   *

Bhutan-09015         CGAMKWYSRRGWAMRGYGTYWWSAMGSTGCYGKAKYCATTAKGRYSCRRCGAKYTTSGRM 40620
Bhutan-09024         CGAMKWYSRRGWAMRGYGTYWWSAMGSTGCYGKAKYCATTAKGRYSCRRCGAKYTTSGRM 40620
Bhutan-09027         CGAMKWYSRRGWAMRGYGTYWWSAMGSTGCYGKAKYCATTAKGRYSCRRCGAKYTTSGRM 40620
Bhutan-09030         CGAMKWYSRRGWAMRGYGTYWWSAMGSTGCYGKAKYCATTAKGRYSCRRCGAKYTTSGRM 40620
Bhutan-09005         CGGCTWCGGRGTTCRSTSATATGGMGCTRSYRKRTYTWKWGTRAYGSARYRMTYYYGRGM 40620
Indonesia-88035      YRGCTWCGGRKTTCRSTSATATGGMGCTRSYRKRTYTWKWGTRAYGSARYRMTYYYGRGM 40620
Indonesia-88045      YRGCTWCGGRKTTCRSTSATATGGMRCKRSYRKRTYTWKWGTRAYGSARYRMTYYYGRGM 40620
Indonesia-88065      CGGCTWCGGRGTTCRSTSATATGGMGCTRSYRKRTYTWKWGTRAYGSARYRMTYYYGRGM 40620
                       . .* . *  : *. .:   ..* .. .* * .*  . ..  *.. *   .*  .  *

Bhutan-09015         TCYCAWRTWWYAGYRYAYCRRRKYYGMYCYRRWRRYGGTGMCRGAGCTAWCCTRWAYYGS 40680
Bhutan-09024         TCYCAWRTWWYAGYRYAYCRRRKYYGMYCYRRWRRYGGTGMCRGAGCTAWCCTRWAYYGS 40680
Bhutan-09027         TCYCAWRTWWYAGYRYAYCRRRKYYGMYCYRRWRRYGGTGMCRGAGCTAWCCTRWAYYGS 40680
Bhutan-09030         TCYCAWRTWWYAGYRYAYCRRRKYYGMYCYRRWRRYGGTGMCRGAGCTAWCCTRWAYYGS 40680
Bhutan-09005         YYYGCWRYWWYWRCRCMCYRRGGCCSMYMCGAARRYRSKKAYGCTATAGAYSWAARYYAC 40680
Indonesia-88035      YYYGCWRYWWYWRCRCMCYRRGGCCSMYMCGAARRYRSKKAYGCTATAGAYSTAARYYAC 40680
Indonesia-88045      YYYGCWRYWWYWRCRCMCYRRGGCCSMYMCGAARRYRSKKAYGCTATAGAYSTAARYYAC 40680
Indonesia-88065      YYYGCWRYWWYWRCRCMCYRRGGCCSMYMCGAARRYRSKKAYGCTATAGAYSWAARYYAC 40680
                       * .** ***   *     **    .**      *** ..      :. :.  .    **..

Bhutan-09015         ACTMRRRRTTSRMRRWACRGKYTTYRATGRSRAYKWRMRCKTSRSTRYTRGKTWTGWTAA 40740
Bhutan-09024         ACTMRRRRTTSRMRRWACRGKYTTYRATGRSRAYKWRMRCKTSRSTRYTRGKTWTGWTAA 40740
Bhutan-09027         ACTMRRRRTTSRMRRWACRGKYTTYRATGRSRAYKWRMRCKTSRSTRYTRGKTWTGWTAA 40740
Bhutan-09030         ACTMRAAGTTSRMRRWACRGKYTTYRATGRSRAYKWRMRCKTSRSTRYTRGKTWTGWTAA 40740
Bhutan-09005         RYYMGGGAYTSGCRAWWYARKTKYCARYRGSRRTGWGAAYKYSASCAYYGRTTWTSTKGR 40740
Indonesia-88035      RYYCGGGATTCGCAATACAGTTTTCARYGGSRATGAGAACGTSASCAYYGRTYWCSTKGR 40740
Indonesia-88045      RYYCGGGATWCGCAATACAGTTTTCARYGGSRATGAGAACGTSASCAYYGRTYWCSTKGG 40740
Indonesia-88065      RYYCGGGAYTSGCRAWWYARKTKYCARYRGSRRTGTGAAYKYSASCAYYGRTTWTSTKGR 40740
                               .         . .       **          * *  *   . * . ..

Bhutan-09015         RRWYRYYWTSRYMAMYRGYCYKSAGYRKRCRGWRRKCTTRWYWCGSAWRYASYYRAYTRK 40800
Bhutan-09024         RRWYRYYWTSRYMAMYRGYCYKSAGYRKRCRGWRRKCTTRWYWCGSAWRYASYYRAYTRK 40800
Bhutan-09027         RRWYRYYWTSRYMAMYRGYCYKSAGYRKRCRGWRRKCTTRWYWCGSAWGCASYYRAYTRK 40800
Bhutan-09030         RRWYATYWTSRYMAMYRGYCYKSAGYRKRCRGWRRKCTTRWYWCGSAWRYASYYRAYTRK 40800
Bhutan-09005         AGWYATCTWSACCWAYARCTYKSRGYARRTARWGAGCKWRWCWYRCRARCCGCYRGCYRG 40800
Indonesia-88035      AGWYATTTWGACCWAYARCTYTGRKYARRTAGWGAGCKWATCWYRCRARCCGCYRGCYRG 40800
Indonesia-88045      AGWYATTTWGACCWAYARCTTKSRKYARRTAGWGAGYKWATCWYRCRARCCGCYRGCYRG 40800
Indonesia-88065      AGWYATCTWSACCWAYARCTTKSRGYARRTARWGAGYKWRWCWYRCRARCCGCYRGCYRG 40800
```

```
                              **     .     *     ..  * :*    *     .     *  .     .. **.  *

Bhutan-09015          TCSCAAGWAARCRRACYRSYAMKMYGYTTRGGGGAAGAKRCRGYAKCKRYYRRAKYYRYY 40860
Bhutan-09024          TCSCRAGWAARCRRACYRSYAMKMYGYTTRGGGGAAGAKRCRGYAKCKRYYRRAKYYRYY 40860
Bhutan-09027          TCSYAAGWAARCRRACYRSYAMKMYGYTTRGGGGAAGAKRCRGYAKCKRYYRRAKYYRYY 40860
Bhutan-09030          TCSYAAGWAARCRRACYRSYAMKMYGYTTRGGGGAAGAKRCRGYAKCKRYYRRAKYYRYY 40860
Bhutan-09005          GWCCAMKTRMRMRGMMTGCTWCGMYRYYWARRRRWRRGKRCRRCRTYTACTAGTGTCGCC 40860
Indonesia-88035       GTCCAMKTAAACAGACTGCTACGCTGTTTAGGGRWARGKRSRRCRTYTACTAGTGTCGCC 40860
Indonesia-88045       GTCCAMKTAAACAGACTGCTACGCTGTTTAGGGRWARGKRSRRCRTYTACTAGTGTCGCC 40860
Indonesia-88065       GWCCAMKTRMRMRGMMTGCTWCGMYRYYWARRRRWRRGKRCRRCRTYTACTAGTGTCGCC 40860
                        .               .                  .**.*    . .      :

Bhutan-09015          KWRKGTYRASGRRWGAAYYYMRWKKWAGRRYRYCARCATGTRCRCAWCCCTKAGKCCTKR 40920
Bhutan-09024          KWRKGTYRASGRRWGAAYYYMRWKKWAGRRYRYCARCATGTRCRCAWCCCTKAGKCCTKR 40920
Bhutan-09027          KWRKGTYRASGAGWGAAYYYMRWKKWAGRRYRYCARCATGTRSRCAWCCCTKAGKCCTKR 40920
Bhutan-09030          KWRKGTYRASGRRWGAAYYYMRWKKWAGRRYRYCARCATGTRCRCAWCCCTKAGKCCTKR 40920
Bhutan-09005          KTGGRWCGAGRRRYRRMTYYMRWKRWRRAACATTTAYWYGYRGGTRWSYYCGRRTMSWKR 40920
Indonesia-88035       TAAGRWCGGGRRRYRRMTYYAAWKATRRAACATTTAYWYKYRGGTRWSYYCGRRTMSWKR 40920
Indonesia-88045       TAAGRWCGGGRRRYRRMTYYAAWKATRRAACATTTAYWYKYRGGTRWSYYCGRRTMSWKR 40920
Indonesia-88065       TTGGRWCGAGRRRYRRMTYYMRWKRWRRAACATTTAYWYGYRGGTRWSYYCGRRTMSWKR 40920
                      .       ..   :    **  **          :      *    *.      . . **

Bhutan-09015          YMGGAGRMGRYSGRRRYYGTMRGTSATYRTMARCKAYCGYWRKCSAGKKTGTYWTMRKSG 40980
Bhutan-09024          YMGGAGRMGRYSGRRRYYGTMRGTSATYRTMARCKAYCGYWRKCSAGKKTGTYWTMRKSG 40980
Bhutan-09027          YMGGAGRMGRYSGRRRYYGTMRGTSATYRTMARCKAYCGYWRKCSAGKKTGTYWTMRKSG 40980
Bhutan-09030          YMGGAGRMGRYSGRRRYYGTMRGTSATYRTMARCKAYCGYWRKCSAGKKTGTYWTMRKSG 40980
Bhutan-09005          YMRKRRAAKAYSARAGTCRWMRAYSWCYAYARRSTMCYSTWGKYSMRGGKTAYWYAGTGR 40980
Indonesia-88035       YMRKRRAAKAYSARAGTCRWMRAYSWCYAYARRSTMCYSTWGKYSMRGGKTAYWYAGTGA 40980
Indonesia-88045       YMRKRRAAKAYSARAGTCRWMRAYSWCYAYARRSTMCYSTWGKYSMRGGKTAYWYAGTGA 40980
Indonesia-88065       YMRKAGAAKAYSARAGTCRWMRAYSWCYAYARRSTMCYSTWGKYSMRGGKTAYWYAGTGA 40980
                      **         **.*      **. *  *    *..   . * * *    . :**   ..

Bhutan-09015          YCTYYWYYMRARYRGYYAAACKTMSTRKMACSSGCTTYRRYYRWWYATMARYCTYRYYYS 41040
Bhutan-09024          YCTYYWYYMRARYRGYYAAACKTMSTRKMACSSGCTTYRRYYRWWYATMARYCTYRYYYS 41040
Bhutan-09027          YCTYYWYYMRARYRGYYAAACKTMSTRKMACSSGCTTYRRYYRWWYATMARYCTYRYYYS 41040
Bhutan-09030          YCTYYWYYMRARYRGYYAAACKTMSTRKMACSSGCTTYRRYYRWWYATMARYCTYRYYYS 41040
Bhutan-09005          CTACCTTTAAWGYGRCCCRGYKYAGCATACTSSAGWYYGGYCAATYRYARGTMYTAYYTS 41040
Indonesia-88035       CTACCTTTAAWGYGRCCCRGYKYAGCATACTSSAGWYYGGYCAATYRYAGGTMYTAYYTS 41040
Indonesia-88045       CTACCTTTAAWGYGRCCCRGYKYAGYATACTSSAGTYYGGYCAATYRYAGGTMYTAYYTS 41040
Indonesia-88065       CTACCTTTAAWGYGRCCCRGYKYAGYATACTSSAGTYYGGYCAATYRYARGTMYTAYYTS 41040
                        :          *     . . *  .  . . **.   *  *     *          ** *

Bhutan-09015          SRGMYMTTTKKYTGYTMYATCAWRGTRCRASMGWAGCMYRRKRYMRKYMSRKYGWGYAKY 41100
Bhutan-09024          CGGMYMTTTKKYTGYTMYATYAWRGTRCRASMGWAGCMYRRKRYMRKYMSRKYRWGYAKY 41100
Bhutan-09027          SRGMYMTTTKKYTGYTMYATYAWRGTRCRASMGWAGCMYRRKRYMRKYMSRKYRWGYAKY 41100
Bhutan-09030          SRGMYMTTTKKYTGYTMYATYAWRGTRCRASMGWAGCMYRRKRYMRKYMSRKYGWGYAKY 41100
Bhutan-09005          SGRMYMGCATTTARYYMCWWTRAGKWGGARCCSARATCYRRKAYMRKTMCGKTGWRTWGM 41100
Indonesia-88035       SGRMYMGCATTTARYTMCWWTRAGKWGGARCCSARATCYRRKAYMRKTMCGKTGWRTWGM 41100
Indonesia-88045       SGRMYMGCATTTARYTMCWWTRAGKWGGARCCSARATCYRRTAYMRKTMCGKTGWRTWGM 41100
Indonesia-88065       SGRMYMGCATTTARYYMCWWTRAGKWGGARCCSARATCYRRKAYMRKTMCGKTGWRTWGM 41100
                      .  ***  :.. : * *              . .  .  ***. **** *. *  *

Bhutan-09015          RKYYACGYWSYSACTRRKTCWATAGGWYKTWYGCMGYWTWYRMRCGRSRGMKYRACAGTM 41160
Bhutan-09024          RGCYACGYWSYSACTRRKTCWATAGGWYKTWYGCMGYWTWYRMRCGRSRGMKYRACAGTM 41160
```

```
Bhutan-09027            RGCYACGYWSYSACTRRKTCWATAGGWYKTWYGCMGYWTWYRMRCGRSRGMKYRACAGTM 41160
Bhutan-09030            RKYYACGYWSYSACTRRKTCWATAGGWYKTWYGCMGYWTWYRMRCGRSRGMKYRACAGTM 41160
Bhutan-09005            RGCYGSRCTCTGGACRGTWYRWYMRKTYTKATASARCWYATGCRYRGCRKCKYAWTTRKM 41160
Indonesia-88035         RGCYGGACTCTGGACRGTWYRAYMRKTYTKATRSAACWYATGCRYRGCRKCKYAWTTRKM 41160
Indonesia-88045         RGCYGSRCTCTGGACRGTWYRAYMRKTYTKATRSAACWYATGCRYRGCRKCKYAWTTRKM 41160
Indonesia-88065         RGCYGGACTCTGGACRGTWYRAYMRKTYTKATASAACWYATGCRYRGCRKCKYAWTTRKM 41160
                        *  *.    . ... * .         *..   .   *     *   .*  **   : .*

Bhutan-09015            ATCMGYCRYWRWRYRMCYKSGKTGAAYYYAAYRTGRGYAWTRKAYWYYCTAGMYGMRTYY 41220
Bhutan-09024            ATCMGYCRYWRWRYRMCYKSGKTGAAYYYAAYRTGRGYAWTRKAYWYYCTAGMYGMRTYY 41220
Bhutan-09027            ATCMGYCRYWRWRYRMCYKSGKTGAAYYYAAYRTGRGYAWTRKAYWYYCTAGMYGMRTYY 41220
Bhutan-09030            ATCMGYCRYWRWRYRMCYKSGKTGAAYYYAAYRTGRGYAWTRKAYWYYCTAGMYGMRTYY 41220
Bhutan-09005            RYACKCSRTARTRCGCYTGCRTYARWYSCRRYAGRRKCWWWRKRCWCYYYRRACAAGYYC 41220
Indonesia-88035         RYACKCSRTARTRCGCYTGCRTYARWYSCRRYAGRRKCWWWRKRCWCYYYRRACAAGYYC 41220
Indonesia-88045         RYACKCSRTARTACGCYTGCRTYARWYSCRACAGRRKCWWWRKRCWCYYYRRACAAGYYC 41220
Indonesia-88065         RYACKCSRTARTRCGCYTGCRTYARWYSCRRYAGRRKCWWWRKRCWCYYYRRACAAGYYC 41220
                          .   .*  *        . . .  *        *   * **  * *      .   *

Bhutan-09015            YTKMRTTYRYYYCYGCWSGGCRATKGGGGGAGAATCGGCMMKYATCMAGCTRKYAAYCGK 41280
Bhutan-09024            YTKMRTTYRYYYCYGCWSGGCGATKGGGGGAGAATCGGCMMKYATCMAGCTRKYAAYCGK 41280
Bhutan-09027            YTKMRTTYRYYYCYGCWSGGCGRTGGGGGGRGAATCGGCMMKYATCMAGCTRKYAAYCGK 41280
Bhutan-09030            YTKMRTTYRYYYCYGCWSGGCGRTKGRGGGRGAATCGGCMMKYATCMAGCTRKYAAYCGK 41280
Bhutan-09005            CCGCACYYACYYYCRYASKRYGAYGSRRRSARMRTYRRYMCTCRYYMWRMWGKYAAYYKT 41280
Indonesia-88035         CCGCACYYACYYYCGYWSGGCGAYGGRGGGAGAAYCGGCCCTCATYAAGCTGTCAAYCGT 41280
Indonesia-88045         CCGCACYYACYYYCGYASGGCGAYGGRGGGAGAAYCGGCCCTCATYAAGCTGTCMMYCGT 41280
Indonesia-88065         CCGCACYYACYYYCGYASKRYGAYGSRRRSARMRTYRRYMCTCRYYMWRMWGKYAAYYKT 41280
                               *  **     *       .   .           .          .   *  .

Bhutan-09015            YYGRRATKYRRRWGRACATCAWYRCATGGWKCCTTCYYRYRCCAACWYGAGGRYRGYMRW 41340
Bhutan-09024            YYGRRATKYRRRWGRACATCAWYRCATGGWKCCTTCYYRYRCCAACWYGAGGRYRGYMRW 41340
Bhutan-09027            YYGRRATKYRRRWGRACATCAWYRCATGGWKCCTTCYYRYRCCAACWYGAGGRYRGYMRW 41340
Bhutan-09030            YYGRRATKYRRRWGRACATCAWYRCATGGWKCCTTCYYRYRCCAACWYGAGGRYRGYMRW 41340
Bhutan-09005            YCRRAMKGCARRWAARMRKSTTCGMTWSMWGYTWCYCCRYAYYGRYTCRWRRATAKCAGA 41340
Indonesia-88035         CCGGAATGCAGATGAACATSTTCGMTWSMWGYTWCYCCRYAYYGRCTCRWRRATAKCARA 41340
Indonesia-88045         CCGGAATGCAGATGAACATSTTCGMTWSMWGYTWCYCCRYAYYGRCTCRWRRATAKCARA 41340
Indonesia-88065         YCRRACGGCARRWAARMRKSTTCGMTWSMWGYTWCYCCRYAYYGRYTCRWRRATAKCAGA 41340
                                     .    ..:    : . *        **   .

Bhutan-09015            KRRYAYRAKWGRTYAYTRRRYRCWTMYWRSRAYMRWGCRATTCRCRTYMARRSCTTGRGC 41400
Bhutan-09024            KRRYAYRAKWGRTYAYTRRRYRCWTMYWRSRACARWGCRATTCRCRTYMARRSCTTGRGC 41400
Bhutan-09027            KRRYAYRAKWGRTYAYTRRRYRCWTMYWRSRAYMRWGCAATTCRCRTYMARRSCTTGRGC 41400
Bhutan-09030            KRRYAYRAKWGRTYAYTRRRYRCWTMYWRSRACARWGCAATTCRCRTYMARRSCTTGRGC 41400
Bhutan-09005            GGRYRTGGGWRGCTRYGGGGCGYACMYARGRCCAGTGYGCCCYAMGWYMCAACTYYKARY 41400
Indonesia-88035         GGRYRTGGGWRGCTRYGGGGCGYACMYARGRCCAGTAYGCCCYAMGWYMCAACTYYKARY 41400
Indonesia-88045         GGRYRTGGGWRGCTRYGGGGCGYACMYARGRCCAGTAYGCCCTAMGWYMCAACTYYKARY 41400
Indonesia-88065         GGRYRTGGGWRGCTRYGGGGCGYACMYARGRCCAGTGYGCCCTAMGWYMCAACTYYKARY 41400
                          **   . *     *         ** *.*.    .  .       **.  .

Bhutan-09015            CARSKMTAYSRYCTWTGRRCYRTRRRCGRTWCYRGRCCCARSYSGRCTCTCRSAYCSWYK 41460
Bhutan-09024            CARSKMTAYSRYCTWTGRRCYRTRRRCGRTWCYRGRCCCARSYSGRCTCTCRSAYCSWYK 41460
Bhutan-09027            CARSKMTAYSRYCTWTGRRCYRTRRRCGRTWCYRGRCCCARSYSGRCTCTCRSAYCSWYK 41460
Bhutan-09030            CARSKMTAYSRYCTWTGRRCYRTRRRCGRTWCYRGRCCCARSYSGRCTCTCRSAYCSWYK 41460
Bhutan-09005            SRGGTATACGGCCTAWAGGCTACRRRGAGGTYCACRMSCTRCCCGGYKSYYRSGCSGWTK 41460
Indonesia-88035         SRGGTAYRCGGCMYAWAGGTTACRRRGAGGTYCACRMSYTRCCCRGYKSYYRSGCSGWTK 41460
```

```
Indonesia-88045      SRGGTAYRCGGCMYAWAGGTTACRRRGAGGTYCACRMSYTRCCCRGYKSYYRSGCSGWTK 41460
Indonesia-88065      SRGGTAYRCGGCMTAWAGGCTACRRRGAGGTYCACRMSCTRCCCGGYKSYYRSGCSGWTK 41460
                     .  ..    .      .      *** .       * . :*. .   ..  **. ..* *

Bhutan-09015         TYYRYKYRAKAACAGWRRTCGKKAMGWMYGRCTMRCACRSMRRYAWTASGTAARYCATGR 41520
Bhutan-09024         TYYRYKYRAKAACAGWRRTCGKKAMGWMYGRCTMRCACRSMRRYAWTASGTAARYCATGR 41520
Bhutan-09027         TYYRYKYRAKAACAGWRRTCGKKAMGWMYGRCTMRCACRSMRRYAWTASGTAARYCATGR 41520
Bhutan-09030         TYYRYKYRAKAACAGWRRTCGKKAMGWMYGRCTMRCACRSMRRYAWTASGTAARYCATGR 41520
Bhutan-09005         KCCRCKSARGGGTMSWRGCMRKKMAAAAYARGYMAYRTASCRRTWAWRGRKGRGYTGYRR 41520
Indonesia-88035      KCCRCKSARGGGTMSWRGCMRKKMAAAAYARGYMAYRTASCRRTWAWRGRKGRGYTGYRR 41520
Indonesia-88045      KCCRCKSARGGGTMSWRGCMRKKMAAAAYARGYMAYRTASCRRTWAWRGRKGRGYTGYRR 41520
Indonesia-88065      KCCRCKSARGGGTMSWRGCMRKKMAAAAYARGYMAYRTASCGRTWAWRGRKGRGYTGYRR 41520
                     .  * *    ..  .**    **  .  *.*  *      *  *     . ..  * .  *

Bhutan-09015         MGCATSTCYYTRARWARCYATYYMCYAKCARYMWRGRKGMMGMAYYTYGYAACGGCCAYR 41580
Bhutan-09024         MGCATSTCYYTRARWARCYATYYMCYAKCARYMWRGRKGMMGMAYYTYGYAACGGCCAYR 41580
Bhutan-09027         MGCATSTCYYTRARWARCYATYYMCYAKCARYMWRGRKGMMGMAYYTYGYAACGGCCAYR 41580
Bhutan-09030         MGCATSTCYYTRARWARCYATYYMCYAKCARYMWRGRKGMMGMAYYTYGYAACGGCCAYR 41580
Bhutan-09005         AAYGYSTSCYYGRRARAYTGYTCATYRTMMRYAAAARKRACRMTCTGCRYRGYRRAARCA 41580
Indonesia-88035      AAYGYSYCCCTGAGAAACTGTTCATYGTMMRYAAAARKRACRMTCTGCRYRGYGGAAACA 41580
Indonesia-88045      AACGYSTCCCTGAGAAACTGTTCATYRTMMRYAAAARKRACRMTCTGCRYRGYGGAAACA 41580
Indonesia-88065      AACGYSTSCYTGRRARAYTGYTCATYRTMMRYAAAARKRACGCTCTGCRYRGYRRRARCA 41580
                      . . * .          .     * .  **   .**      :      * .    .

Bhutan-09015         RMCCRCWKYRTSWGGYMRCKKCARYYRWKCRRRMYGMYMAAAYCMCRCTTYRWAWTYWAY 41640
Bhutan-09024         RMCCRCWKYRTSWGGYMRCKKCARYYRWKCRRRMYGMYMAAAYCMCRCTTYRWAWTYWAY 41640
Bhutan-09027         RMCCRCWKYRTSWGGYMRCKKCARYYAWKCRRRMYGMYMAAAYCMCRCTTYRWAWTYWAY 41640
Bhutan-09030         RMCCRCWKYRTSWGGYMRCKKCARYYRWKCRRRMYGMYMAAAYCMCRCTTYRWAWTYWAY 41640
Bhutan-09005         GMMAASAKMRWGTRRCCGYGKYCATCAAKMRGRCYRCCMMRMCMCYRTYCCAATAWTWMY 41640
Indonesia-88035      GMCAASAKMATGTGGCCGYGKYAATCAAKMRGRCYGCCMMGCCMCYRTYCCAATAWTWMY 41640
Indonesia-88045      GMCAASAKMATGTGGCCGYGKYAATCAAKMRGRCYGCCMMGMCMCYRTYCCAATAWTWMY 41640
Indonesia-88065      GMMAASAKMRWGTRRCCGYGKYAATCAAKMRGRCYRCCMMRMCMCYRTYCCAATAWTWMY 41640
                      * . . *    .        * .      * * * *   *       *       :   * *

Bhutan-09015         YRYTCRRCTGKRGYAWTMYYRCMRRSCYRYRWAYKAYMYTGMMYWTTYYTTRYYWAGRYG 41700
Bhutan-09024         YRYTCRRCTGKRGYAWTMYYRCMRRSCYRYRWWCGAYMYTGMMYTTTYYTTRYYWAGRYG 41700
Bhutan-09027         YRYTCRRCTGKRGYAWTMYYRCMRRSCYRYRWWCGAYMYTGMMYWTTYYTTRYYWAGRYG 41700
Bhutan-09030         YRYTCRRCTGKRGYAWTMYYRCMRRSCYRYRWWYKAYMYTGMMYWTTYYTTRYYWAGRYG 41700
Bhutan-09005         YRYWYAGMKRTRATRTWATTAYAAGCYCGTAWWYGWTMYYSAATTWWTYYARTCWWRAYA 41700
Indonesia-88035      YRYWYAGMKRTRATRTWATTAYAAGCYCGTAWWYGWTMYYSAATTWWTYYARTCWWRAYA 41700
Indonesia-88045      YRYWYAGMKRTRATRTWATTAYAAGCYCGTAWWYGWTMYYSAATTWWTYYARTCWWRAYA 41700
Indonesia-88065      YRYWYAGMKRTRATRTWATTAYAAGCYCGTAWWYGWTMYYSAATTWWTYYARTCWAGATA 41700
                     ***      . .*.              .     *     ** .       * :*  *    .

Bhutan-09015         YRGYCTCGGYMWGKMSWWCCYKGATYGTWCCKYTGTKYTYSCTGRCWKCYRTAGMCTTGS 41760
Bhutan-09024         YRGYCTCGGYMWGKMSWWCCYKGATYGTWCCKYTGTKYTYSCTGRCWKCYRTAGMCTTGS 41760
Bhutan-09027         YRGYCTCGGYMWGKMSWWCCYKGATYGTWCCKYWGTKYTYSCTGRCWKCYRTAGMCTTGS 41760
Bhutan-09030         YRGYCTCGGYMWGKMSWWCCYKGATYGTWCCKYWGTKYTYSCTGRCWKCYRTAGMCTTGS 41760
Bhutan-09005         CARTYKYRRYCAAGMKAWTTCKRRYCRGASYKYTKYKCYYCMCRASTTYTGYWRASKWSG 41760
Indonesia-88035      CARTYKYRRYCAAGMKAWTTCGARYCRGASYKYTKYKCYYCMCAASTTYTGYWRASKWSG 41760
Indonesia-88045      CARTYKYRRYCAAGMKAWTTCGARYCRGASYKYTKYKCYYCMCAASTTYTGYWRASKWSG 41760
Indonesia-88065      CAGTYKYRRYCAAGMKAWTTCKRRYCRGASYKYTKYKCYYCMCAASTTYTGYWRASKWSG 41760
                          .   *  . *. *            . **   *  *.     . .        .. ..
```

```
Bhutan-09015       RARGCTACKTYYRCRYRYAWGMSTWCRCYCCWRMRRYKCRGSACGCAGTGASRYKYRTCT 41820
Bhutan-09024       RARGCTACKTYYRCRYRYAWGMSTWCRCYCCWRMRRYKCRGSACGCAGTGASRYKYRTCT 41820
Bhutan-09027       RARGCTACKTYYRCRYRYAWGMSTWCRCYCCWRMRRYKCRGSACGCAGTGASRYKYRTCT 41820
Bhutan-09030       RARGCTACKTYYRCRYRYAWGMSTWCRCYCCWRMRRYKCRGSACGCAGTGASRYKYRTCT 41820
Bhutan-09005       WCRKSYWMTKTTRYGTATRWKMGWWYGYYYYAAMGRYKMAKGRMRYWRWKCSACTCGCTK 41820
Indonesia-88035    TCRKCYWMTKTTRYGTATRWKMGWWYGYYYYAAMGRYTMAKGRMGCAGTGCSACTCGCTK 41820
Indonesia-88045    TCRKCYWMTKTTRYGTATGWKMGWWYGYYYYAAMGRYKMAKGRMGCAGTGCSACTCGCTK 41820
Indonesia-88065    WCRKSYWMTKTTRYGTATGWKMGWWYGYYYYAAMGRYKMAKGRMRYWRWKCSACTCGCTK 41820
                    .* .   ..  *      * *. *   *    * **.   .        .*  .    .

Bhutan-09015       TACKMRARRCRRWCWYMRGARTKCYMTRMYARTKRMKWYTTCCTRKGAKYTCTATGRSCA 41880
Bhutan-09024       TACKMRWRRCRRWCWYMRGARTKCYMTRMYARTKRMKWYTTCCTRKGAKYTCTATGRSCA 41880
Bhutan-09027       TACKMRWRRCRRWCWYMRGARTKCYMTRMYARTKRMKWYTTCCTRKGAKYTCTATGRSCA 41880
Bhutan-09030       TACKMRWRRCRRWCWYMRGARTKCYMTRMYARTKRMKWYTTCCTRKGAKYTCTATGRSCA 41880
Bhutan-09005       YGTTARAAGYARWYWYCGRRGYTYTCYGACRRCKGAKWYCCTTWGTSRKCCTYRCRGGYR 41880
Indonesia-88035    YGTTARAAGYARWYWYCGRRGYTYTCYGACRRCKGAKWYCCTTWGTSRKCCTYRCRGGYR 41880
Indonesia-88045    YGTTARAAGYARWYWYCGRRGYTYTCYGACRRCKGAKWYCCTTWGTSRKCCTYRCRGGYR 41880
Indonesia-88065    YGTTARAAGYARWYWYCGRRGYTYTCYGACRRCKGAKWYCCTTWGTSRKCCTYRCRGGYR 41880
                    . . *     ** **      .        * *  ***      .. *        .

Bhutan-09015       RACYCAGGGTKGYAACGTYMRRYYAGRKTRYWCACWYRYYYRKYCTYRYAYAWYWTSAMT 41940
Bhutan-09024       RACYCAGGGTKGYAACGTYMRRYYAGRKTRYWCACWYRYYYRKYCTYRYAYAWYWTSAMT 41940
Bhutan-09027       RACYCAGGGTKGYAACGTYMRRYYAGRKTRYWCACWYRYYYRKYCTYRYAYAWYWTSAMT 41940
Bhutan-09030       RACYCAGGGTKGYAMCGTYMRRYYAGRKTRYWCACWYRYYYRKYCTYRYAYAWYWTSAMT 41940
Bhutan-09005       AWYYARKGRYKATRAGAYYAARCTGRRKCAYWCRCACAYYYGGCAYYGTWTCWTACGRMY 41940
Indonesia-88035    AWYYAGTGRYKATRAGAYYAARCTGRRKCAYTCACACACCYRGCAYYGTWTCWTACGRMY 41940
Indonesia-88045    AWYYARKRRYKATRAGAYYAARCTGRRKCAYWYAYACACCYRGCAYYGTWTCWTACGRMY 41940
Indonesia-88065    AWYYARKGRYKATRAGAYYAARCTGRRKCAYWYACACAYCYRGCAYYGTWTCWTACGRMY 41940
                      *.     *.    . *  *  . **  *         *   . *    .*   . *

Bhutan-09015       GACMYAWCTWCTYYMTCRWGYSTWMKWRKRAKWRYYYTAWTKYMTCSYCMTRWTWCCYKG 42000
Bhutan-09024       GACMYAWCTWCTYYMTCRWGYSTWMKWRKRAKWRYYYTAWTKYMTCSYCMTRWTWCCYKG 42000
Bhutan-09027       GACMYAWCTWCTYYMTCRWGYSTWMKWRKRAKWRYYYTAWTKYMTCSYCMTRWTWCCYKG 42000
Bhutan-09030       GACMYAWCTWCTYYMTCRWGYSTWMKWRKAAKWRYYYTAWTKYMTCSYCMTRWTWCCYKG 42000
Bhutan-09005       KCYACRAYYWAYYTCGYRTRTSYACKWRKGGKTGTTTCGTYKYMCCCTCAGRWKAYMTGA 42000
Indonesia-88035    KCYACRAYYWAYYTCGCRTRTSYACKWRKGRKTGTTTCGTYKYACYCTAAGRWKAYMTGA 42000
Indonesia-88045    KCYACRAYYWAYYTCGCRTRTSYACKWRKGRKTGTTTCGTYKYACYCTAAGRWKAYMTGA 42000
Indonesia-88065    KCYACRAYYWAYYTCGCRTRTSYACKWRKGRKTGTTTCGTYKYMCCCTCAGRWKAYMTGA 42000
                    .       *. *    *   *   ****  *      .  **   . .  **.     .

Bhutan-09015       TGGCRATTWCRRRKTCCYAGGSCWMTTRYWTGGGYMCRCCYMCCWSTMYRTSRTYSYSTR 42060
Bhutan-09024       TGGCRATTWCRRRKTCCYAGGSCWMTTRYWTGGGCACRCCYMCCWSTMYRTSRTYSYSTR 42060
Bhutan-09027       TGGCRATKWCRRRKTCCYAGGSCWMTTRYWTGGGCACRCCYMCCWSTMYRTSRTYSYSTR 42060
Bhutan-09030       TGGCRATKWCRRRKTCCYAGGSCWMTTRYWTGGGYMCRCCYMCCWSTMYRTSRTYSYSTR 42060
Bhutan-09005       CSRYGWAKYYRAGKCYSCRAASSTCCCGYAYTTACAMGYGTCYYAGYCCRWGAYYCTSWA 42060
Indonesia-88035    CSRYGWATYYRAGKCYSCRAASSTCCCGTAYTTACACGCGTCYYAGYCCGWGAYYCTSWA 42060
Indonesia-88045    CSRYGWAKYYRAGKCYSCRAASSTCCCGTATTTACACGCGTCYYAGYCCGWGAYTCTSWA 42060
Indonesia-88065    CSRYGWAKYYRAGKCYSCRAASSTCCCGYAYTTACAMGYGTCYYAGYCCRWGAYYCTSWA 42060
                    .    :.: *  *  ..*.          .           .     .   . *

Bhutan-09015       YWRYRYRARTAKAWRTTRCYYYYRYAWRYRRCRRRGCYCYRRYGRMTTRYRKRWTRKYYT 42120
Bhutan-09024       YWRYRYRARTAKAWRTTRCYYYYRYAWRYRRCRRRGCYCYRRYGRCTTRYRKRWTRKYYT 42120
Bhutan-09027       YWRYRYRARTAKAWRTTRCYYYYRYAWRYRRCRRRGCYCYRRYGRCTTRTGKRWTRKYYT 42120
Bhutan-09030       YWRYRYRARTAKAWRTTRCYYYYRYAWRYRRCRRRGCYCYRRYGRMTTRYRKRWTRKYYT 42120
```

```
Bhutan-09005           CTACGTATGYRKRTGKYRSCTYTGYRWGCGRYGRARYSCTRRCKGCWYRCATRWYRKTTK 42120
Indonesia-88035        CTACGTATGYRTATGGTACCTYTGTATGCGRYGRAGYSTTRRCKGCTYRCATRWYRKTTG 42120
Indonesia-88045        CTACGTATGYRTATGGTACCTYTGTATGCGRYGRAGYSTTRRCGGCWYRCATRWYRKTTG 42120
Indonesia-88065        CTACGTATGYRKRTGKYRSCTYTGYRWGCGRYGRARYSCTRRCGGCTYRCATRWYRKTTK 42120
                              :   .      .  *        *  *      **      *  .** **

Bhutan-09015           TGYCGRMCCAMKWSRCAGGCAYSMAYMYSTRAAYRGTRCYRAGTARKAGACWTCRGARRG 42180
Bhutan-09024           TGYCGRMCCAMKWSRCAGGCAYSMAYMYSTRAAYRGTRCYRAGTARKAGACWTCRGARRG 42180
Bhutan-09027           TGYCGRMCCAMKWSRCAGGCAYSMAYMYSTRAAYRGTRCYRAGTARKAGACWTCRGARRG 42180
Bhutan-09030           TGYCGRMCCAMKWSRCAGGCAYSMAYMYSTRAAYRGTRCYRAGTARKAGACWTCRGARRG 42180
Bhutan-09005           WRTYRRCGGMMKACAYRSCGWYSAWTAYGGGGMYGTCRMYKRCYAGGGAMYAKMASWRAS 42180
Indonesia-88035        WRTYRRCGSAMKACRYRSCGWYSAWTAYGGGAMYGTCRMYKRCYRGGGAACAKMASWRAS 42180
Indonesia-88045        WRTYRRCGSAMKACRYRSCGWYSAWTAYGGGAMTGTCRMYKRCYRGGGAACAKMASWRAS 42180
Indonesia-88065        WRTYRRCGGMMKACRYRSCGWYSAWTAYGGGGMYGTCRMYKRCYRGGGAMYAKMASWRAS 42180
                            *    ** .   .   **    *.  .     * *:      ..   .  . * .

Bhutan-09015           KRYGKCGGATTYCRGRSGYGGTARWWTKTAAGRCACASKTCYGTCKGAATRYYGAAARAA 42240
Bhutan-09024           KRYGGCGGATTMCRGRSGYGGTARWWTKTAAGRCACASKTCYGTCKGAATRYYGAAARAA 42240
Bhutan-09027           KRYGKCGGATTYCRGRSGYGGTARWWTKTAAGRCACASKTCYGTCKGAATRYYGAAARAA 42240
Bhutan-09030           KRYGKCGGATTCCRGRSGYGGTARWWTKTAAGRCACASKTCYGTCKGAATRYYGAAARAA 42240
Bhutan-09005           TGCSTYKSRYKYCRRRCAYRRKMATTYGARRRRTMSGSTKMCKYSKARTWACTKRRRRWW 42240
Indonesia-88035        TGCSTYKSRYKCCRRRCAYRRKMATTYGTAAGRTMSGSTKCCGTSKAATWACTKRRRRWW 42240
Indonesia-88045        TGCSTYKSRYKCARRRCAYRRKMATTYGTAAGRTMSGSTKCCGTSKARTWACTKRRRRWW 42240
Indonesia-88065        TGCSTYKSRYKCCRRRCAYRRKMATTYGARRRRTMSGSTKCCGTSKAATWACTKRRRRWW 42240
                       .  .   .  . .* *..*  .      :   *  ..*..    .*. :        *

Bhutan-09015           RRRMYRWTASAYSRYYTRCRACSWYMGKKCTYGGRYYRSAKWATYRTGRMMACAAARRRY 42300
Bhutan-09024           RRRMYRWTASAYSRYYTRCRACSWYMGKKCTYGGRYYRSAGWATYRTGRMMACAAARRRY 42300
Bhutan-09027           RRRMYRWTASAYSRYYTRCRACSWYMGKKCTYGGRYYRSAGWATYRTGRMMACAAARRRY 42300
Bhutan-09030           RRRMYRWTASAYSRYYTRCRACSWYMGKKCTYGGRYYRSAKWATYRTGRMMACAAARRRY 42300
Bhutan-09005           AAAMYRWYRCRYGGYCAAYARYSWYCKTTYKTRRGCTASWGARYCAGRACAWTRWGGRRY 42300
Indonesia-88035        AAACCGTYACACGGYCAAYAGCGWYCGTTYKTRRGCTAGAGARTCAGRACAWTAAGGRRY 42300
Indonesia-88045        AAACCGTYACACGGYCAAYARCGWYCGTTYKTRRGCTAGAGARTCAGRACAWTAAGGRRY 42300
Indonesia-88065        AAAMYRWYRCRYGGYCAAYARYSWYCKTTYKTRRGCTASWGARYCAGRACAWTGWGGRRY 42300
                                .  . * :     .**  .. .       .                . ***

Bhutan-09015           WARRMYWYRMGTYWMYRMGCGTTACAWGYMRYRYCGRWYYTCYCSCKYCYSYWRYCGRRC 42360
Bhutan-09024           WARRMYWYRMGTYWMYRMGCGTTACAWGYMRYRYCGRWYYTCYCSCKYCYSYWRYCGRRC 42360
Bhutan-09027           WARRMYWYRMGTYWMYRMGCGTTACAWGYMRYRYCGRWYYTCYCSCKYCYSYWRYCGRRC 42360
Bhutan-09030           WARRMYWYRMGTYWMYRMGCGTTACAWGYMRYRYCGRWYYTCYCSCKYCYSYWRYCGRRC 42360
Bhutan-09005           WRGAWTAYRAAKCAAYAAKSCCGMSRARYMRCRCTKGATTYYCYGTTCYTGTWRTYKAGY 42360
Indonesia-88035        WRGAWTAYRAAKCAAYAAKSCCGACAAGCCGCRCTKGATTYYCYGYTCYTGTWRTYKAGY 42360
Indonesia-88045        WRGAWTAYRAAKCAAYAAKSCCGACAAGCCGCRCTKGATTYYCYGYTCYTGTWRTYKAGY 42360
Indonesia-88065        WRGAWTAYRAAKCAAYAAKSCCGMSRARYMRCRCTKGATTYYCYGTTCYTGTWRTYKAGY 42360
                       *      ** ..   *   .    .       *           . .   . **

Bhutan-09015           CACAMKWGRSYCRYYCGYKRGTAKTGAACYSYMAGAGACTGMCATGGCCGAARYMRCTWR 42420
Bhutan-09024           CACAMKWGRSYCRYYCGYKRGTAKTGAACYSYMAGAGACTGMCATGGCCGAARYMRCYWR 42420
Bhutan-09027           CACAMKWGRSYCRYYCGYKRGTATTGAACYSYMAGAGACTGMCATGGCCGAARYMRCYWR 42420
Bhutan-09030           CACAMKWGRSYCRYYCGYKRGTATTGAACYSYMAGAGACTGMCATGGCCGAARYMRCTWR 42420
Bhutan-09005           YRYWMTAKGSCYATCSRYGGRKRTKRMRMCSYMCRRRRSCKCTTTRRYYARRACAATYAA 42420
Indonesia-88035        CACAMTAKGSCCATCCGTGGRTAKTGAACCSYMCGAGRSCKCTTTRGCCAARACAATYAR 42420
Indonesia-88045        CACAMTAKGSCCATCCGTGGRTAKTGAACCSYMCGAGRSCKCTTWRGCCAARACAATYAR 42420
Indonesia-88065        YRYWMTAKGSCYATCSRYGGRKRTKRMRMCSYMCRRRRSCKCTTTRRYYARRACAATYAA 42420
```

```
                        *.   *     .    . ..     ***.    .    :     .

Bhutan-09015        RCTYWARWMMRTCAGGGSYSKTATTGAYYGYKRSARYARRYTWRSMRAAYTRCYTTTRYW 42480
Bhutan-09024        RCTYWARWMMRTCAGGGSYSKYATTGAYYGYKRSARYARRYTWRSMRAAYTRCYTTTRYW 42480
Bhutan-09027        RCTYWARWMMRTCAGGGSYSKTATTGAYYGYKRSAATARRYTWRSMRAAYTRCYTTTRYW 42480
Bhutan-09030        RCTYWARWMMRTCAGGGSYSKTATTGAYYGYKRSAATARRYTWRSMRAAYTRCYTTTRYW 42480
Bhutan-09005        GGYCTRRWCCAWCMRARCTGGYWWWKMTYRCKRGRATWAAYKAGGAARRTKAYTWKGGTA 42480
Indonesia-88035     RGTCTRRWCCAWYCRAGCTGGYWTTGATTRCGGGRATWAAYKAGGAAARTTACTWKGGTA 42480
Indonesia-88045     RGTCTRRWCCAWYCRAGCTGGYWTTGATTRCGGGRATWAAYKAGGAAARTTACTWKGGTA 42480
Indonesia-88065     GGYCTRRWCCAWCCRARCTGGYWWWKMTYRCKRGRATWAAYKAGGAARRTKAYTWKGGTA 42480
                          **       . . .            .     *.  .     .    .

Bhutan-09015        TTTRGCCAGYYYYTCCYKGTAATKYAYRTGGGMGRYARRAKGAYKGKACGGGCSKCGGCT 42540
Bhutan-09024        TTTRGCCAGYYYYTCCYKGTAATKYAYRTGGGMGRYARRAKGAYKGKACGGGCSKCGGCT 42540
Bhutan-09027        TTTRGCCAGYYYYTCCYKGTAATKYAYRTGGGMGRYARRAKGAYKGKACGGGCSKCGGCT 42540
Bhutan-09030        TTTRGCCAGYYYYTCCYKGTAATKYAYRTGGGMGRYARRAKGAYKGKACGGGCSKCGGCT 42540
Bhutan-09005        CYKGRMMRRTCYCWYYYGRWRRYGTRCGCGRRATACRAAWTRRYKKGRSKRRYCGYRKYY 42540
Indonesia-88035     CTKGGMCARTCTCTCCTGGTAGCGTACGCGGGMKACAAAATGATGGGACGGGCCGCGGCT 42540
Indonesia-88045     CTKGGMCARTCTCTCCTGGTAGCGTACGCKGGMKACAAAATGATGGGACGGGCCGCGGCT 42540
Indonesia-88065     CYKGRMMRRTCYCWYYYGRWRRYGTRCGCGRRMKACRAAWTRRYKKGRSKRRYCGYRKYY 42540
                      .                                   .       .    .

Bhutan-09015        YGTTGTAGCCYGCYAMYYCSGAACCCRGWWKYYGYYGWRWRCMMYTRRWYGSRYWWCGTY 42600
Bhutan-09024        YGTTGTAGCCYGCYAMYYCSGAACCCRGWWKYYGYYGWRWRCMMYTRRWYGSRYWWCGTY 42600
Bhutan-09027        YGTTGTAGCCYGCYAMYYCSGAACCCRGWWKYYGYYGWRWRCMMYTRRWYGSRYWWCGTY 42600
Bhutan-09030        YGTTGTAGCCYGCYAMYYCSGAACCCGGWWKYYGYYGWRWRCMMYTRRWYGSRYWWCGTY 42600
Bhutan-09005        YTGYRWRRMYCRTCMATYYGRRRYYMGKWWKYCRYCRARARTACTYRGWTKGATATYRYT 42600
Indonesia-88035     CTGTGTAGCCCGTCMATTCGGAACCCGGWAGTCGTCGAAAATACTTAGWTKGATATYRYT 42600
Indonesia-88045     CTGTGTAGCCCGTCMATTCGGAACCCGGWAGTCGTCGAAAATACTTAGWTKGATATYRYT 42600
Indonesia-88065     YTGYRWRRMYCRTCMATYYGRRRYYMGKWWKYCRYCRARARTACTYRGWTKGATATYRYT 42600
                                      .        *                  *  .

Bhutan-09015        MWRYWMACTYMYR 42613
Bhutan-09024        MWRYWMACTYMYR 42613
Bhutan-09027        MWRYWMACTYMYR 42613
Bhutan-09030        MWRYWMACTYMYR 42613
Bhutan-09005        MTRCAMMMKYACR 42613
Indonesia-88035     MTRCAMMMKYACR 42613
Indonesia-88045     MTRCAMMMKYACR 42613
Indonesia-88065     MTRCAMMMKYACR 42613
                    * *  *  .*  *
```

Supplementary Figure S3.

```
CLUSTAL 2.1 multiple sequence alignment


Bhutan-09015        MMMMMMMMMMMMMMMMMMMMMMMMMMMMMMMMMMMMMMMMMMMMMMMMMMMMMMMMMMMM 60
Bhutan-09024        MMMMMMMMMMMMMMMMMMMMMMMMMMMMMMMMMMMMMMMMMMMMMMMMMMMMMMMMMMMM 60
Bhutan-09027        MMMMMMMMMMMMMMMMMMMMMMMMMMMMMMMMMMMMMMMMMMMMMMMMMMMMMMMMMMMM 60
Bhutan-09030        MMMMMMMMMMMMMMMMMMMMMMMMMMMMMMMMMMMMMMMMMMMMMMMMMMMMMMMMMMMM 60
Bhutan-09005        ACMMMMMMMMMMMMMCMMMMAMMMMMAAAMAAAMAMMMCCCMCMCCMMMMAACCCMMMMA 60
Indonesia-88035     MCCAACACCAACMCMMAMMCMCMAAMMMMMMMMMCMACCMMAAMAMCMCMAMMMMMCCCCM 60
Indonesia-88045     MCCAACACCAACACCMAMMCMCCAAMMMMMMMMMCMACCMMAAMAMCCCCMMMMMMCCCMM 60
Indonesia-88065     AMMMMMMMMMMMMMMCMCCMAMMAMAAAAAMAAMAMMMCCCMCMCMCMMMMACCCMMMMA 60


Bhutan-09015        MMMMMMMMMMMMMMMMMMMMMMMMMMMMMMMMMMMMMMMMMMMMMMMMMMMMMMMMMMMM 120
Bhutan-09024        MMMMMMMMMMMMMMMMMMMMMMMMMMMMMMMMMMMMMMMMMMMMMMMMMMMMMMMMMMMM 120
Bhutan-09027        MMMMMMMMMMMMMMMMMMMMMMMMMMMMMMMMMMMMMMMMMMMMMMMMMMMMMMMMMMMM 120
Bhutan-09030        MMMMMMMMMMMMMMMMMMMMMMMMMMMMMMMMMMMMMMMMMMMMMMMMMMMMMMMMMMMM 120
Bhutan-09005        MCMMMCMCCAACAAMMCMMCMMMMAAAMMMMMMCMMMMMMMMMCMMMMMMMMAMMMMMMM 120
Indonesia-88035     CCMACMAAMMMMMMMAMCAMCACCMMMACACCCMCACCAAMAAMAACCMACMMCCACAMA 120
Indonesia-88045     CMMACMAACMMMMMAAMCAMCACCMMMACACCMMCACCAMMAAMAACCMACAACCACAMA 120
Indonesia-88065     MMCMMCMCCAACAAMMCMMMMAMMMAAMMMMMMCMMMMMMAMMCMMMMAMMMACMMMMCM 120


Bhutan-09015        MMMRRRRRRRRRRRRRRRRRRRRRRRRRRRRRRRRRRRRRRRRRRRRRRRRRRRRRRRRR 180
Bhutan-09024        MMMRRRRRRRRRRRRRRRRRRRRRRRRRRRRRRRRRRRRRRRRRRRRRRRRRRRRRRRRR 180
Bhutan-09027        MMMRRRRRRRRRRRRRRRRRRRRRRRRRRRRRRRRRRRRRRRRRRRRRRRRRRRRRRRRR 180
Bhutan-09030        MMMRRRRRRRRRRRRRRRRRRRRRRRRRRRRRRRRRRRRRRRRRRRRRRRRRRRRRRRRR 180
Bhutan-09005        MMAARRRARRAGRRRRAARRRRRRRRRRRRRRRRRRRRRRRRRGRGRRRGRRARRAAGRA 180
Indonesia-88035     CCMRRGARARRRAARARRRGRGGAGAGAGGGAGAAAAGGAGRARARGAGRAGGAGGRRAR 180
Indonesia-88045     CCMRARRRAGRRARGRRRAGGGGAGAGAGGGAGAAAAGGAGARRARGRGRAGGRRGRRAR 180
Indonesia-88065     MMMARRRRRRAGRRRRAARRRRRRRRRRRRRRRRRRRRRRRRRARGRGRRRGARARRAAGRA 180


Bhutan-09015        RRRRRRRRRRRRRRRRRRRRRRRRRRRRRRRRRRRRRRRRRRRRRRRRRRRRRRRRRRRR 240
Bhutan-09024        RRRRRRRRRRRRRRRRRRRRRRRRRRRRRRRRRRRRRRRRRRRRRRRRRRRRRRRRRRRR 240
Bhutan-09027        RRRRRRRRRRRRRRRRRRRRRRRRRRRRRRRRRRRRRRRRRRRRRRRRRRRRRRRRRRRR 240
Bhutan-09030        RRRRRRRRRRRRRRRRRRRRRRRRRRRRRRRRRRRRRRRRRRRRRRRRRRRRRRRRRRRR 240
Bhutan-09005        AARRRRRRRRRAARRRRRGRRGRRRRARGGRRARRGRAAAARRAARRGAGAARRRRARRR 240
Indonesia-88035     GRAAGGGGGRRGGGAGGRARRRGAGRRARARRRGGRARRRRAARRAGGGRRRAGAARGRG 240
Indonesia-88045     GRRAGGGGGRRGGGRRGGARARGAGGRARAAGRRGRARRRRARRRAGRGRRRRGAARGRG 240
Indonesia-88065     AARRRRRRRRGGAAGRRRRGGRGRRRRARGGRRARRGRAAAARRAARRGAGARRRRRRRGR 240


Bhutan-09015        RRRRRRRRRRRRRRRRRRRRRRRRRRRRRRRRRRRRRRRRRRRRRRRRRRRRRRRRRRRR 300
Bhutan-09024        RRRRRRRRRRRRRRRRRRRRRRRRRRRRRRRRRRRRRRRRRRRRRRRRRRRRRRRRRRRR 300
Bhutan-09027        RRRRRRRRRRRRRRRRRRRRRRRRRRRRRRRRRRRRRRRRRRRRRRRRRRRRRRRRRRRR 300
Bhutan-09030        RRRRRRRRRRRRRRRRRRRRRRRRRRRRRRRRRRRRRRRRRRRRRRRRRRRRRRRRRRRR 300
Bhutan-09005        RGRRGRRRRRGARRRRGRRRRRRRRGGRRRRGARRAAAARRRRGGGRGRRRRRRRRARGR 300
Indonesia-88035     GRGGGGAGGGAGGGRRAGAGGARGARRRAAGRRAARRRRAAAGRRARRAARAAAAGAGRG 300
Indonesia-88045     RRGGRGAGGGAGGGGGAGAGGAAGARRARRGRRARRRRRRRAGRRAARAAAAAAAGAGRG 300
Indonesia-88065     RRRRRRRRRRGARRRRGRRGRRARRGRRRRRRARRAAAARRRRGGGRGRRRRRRRRRRRR 300
```

```
Bhutan-09015        RRRRRRRRRRRRRRRRRRRRRRRRRRRRRRRRRRRRRRRRRRRRRRRRRRRRRRRRRRRR 360
Bhutan-09024        RRRRRRRRRRRRRRRRRRRRRRRRRRRRRRRRRRRRRRRRRRRRRRRRRRRRRRRRRRRR 360
Bhutan-09027        RRRRRRRRRRRRRRRRRRRRRRRRRRRRRRRRRRRRRRRRRRRRRRRRRRRRRRRRRRRR 360
Bhutan-09030        RRRRRRRRRRRRRRRRRRRRRRRRRRRRRRRRRRRRRRRRRRRRRRRRRRRRRRRRRRRR 360
Bhutan-09005        GRARGRGRRARRRAARRRRRRRGRGRARRRARRRRRRARRGARRRRRRRAGRRRRRRAGR 360
Indonesia-88035     RGGARARAARGGARAARRGRAGRGRARAGRRRRGAARRRRRARAGGARRRGAGAAGARRA 360
Indonesia-88045     RGGRRARAARRGRRRAGGGRRGRGRARAGGRRRGAAGRGARRAAGGRARRRAGAAGARRA 360
Indonesia-88065     GRARGRGRRARRRARAGRRARRGRGRARRRAAARRRRARRGARRRRRRGAGRRRRRRRGR 360


Bhutan-09015        RRRRRRRRRRRRRRRRRRRRRRRRRRRRRRRRRRRRRRRRRRRRRRRRRRRRRRRRRRRR 420
Bhutan-09024        RRRRRRRRRRRRRRRRRRRRRRRRRRRRRRRRRRRRRRRRRRRRRRRRRRRRRRRRRRRR 420
Bhutan-09027        RRRRRRRRRRRRRRRRRRRRRRRRRRRRRRRRRRRRRRRRRRRRRRRRRRRRRRRRRRRR 420
Bhutan-09030        RRRRRRRRRRRRRRRRRRRRRRRRRRRRRRRRRRRRRRRRRRRRRRRRRRRRRRRRRRRR 420
Bhutan-09005        RARRRRGGGRGGGGRGAAGARARRRARAGGRRRRRRRRRRRRRRRRRRRRRRRRRRRRRR 420
Indonesia-88035     GRAGGGRRRARRRRGRRRRRGRAAARGRRRAGAAGAGGGAAGAGGAGAGGAGAGGAGAAA 420
Indonesia-88045     GRAGRRRRRARRRRGRRRRRGRAAARGRRRAGAAGAGGGAAGAGGAGAGGAGAGGAGARR 420
Indonesia-88065     RARRRRRRRAGGGGRGAAGAGAARRARAGGRRRRRRRRRRRRRRRRRRRRRRRRRRRRRR 420


Bhutan-09015        RRRRRRRRRRRRRRRRRRRRRRRRRRRRRRRRRRRRRRRRRRRRRRRRRRRRRRRRRRRR 480
Bhutan-09024        RRRRRRRRRRRRRRRRRRRRRRRRRRRRRRRRRRRRRRRRRRRRRRRRRRRRRRRRRRRR 480
Bhutan-09027        RRRRRRRRRRRRRRRRRRRRRRRRRRRRRRRRRRRRRRRRRRRRRRRRRRRRRRRRRRRR 480
Bhutan-09030        RRRRRRRRRRRRRRRRRRRRRRRRRRRRRRRRRRRRRRRRRRRRRRRRRRRRRRRRRRRR 480
Bhutan-09005        GRRRRRRRRRRRRRRRRRRRRRRRRRRRRRRRRRRRRRRRRRRARRGRRGARRGRRRRRR 480
Indonesia-88035     AGGGRGGAAGAGGAAAAAGAAAGAAAAGAAGAAAAGAAAAGAGRGRRGRRRARRGAAGG 480
Indonesia-88045     AGGGGGGAAGAGGAAAAAGAAAGAAAAGAAGAAAAGAAAAGARARGRGARRGRRGGAAGG 480
Indonesia-88065     GRGRGRRRRRRRRRRRRRRRRRRRRRRRRRRRRRRRRRRRRRRRARRGRRGRGRGRRRRRR 480


Bhutan-09015        RRRRRRRRRRRRRRRRRRRRRRRRRRRRRRRRRRRRRRRRRRRRRRRRRRRRRRRRRRRR 540
Bhutan-09024        RRRRRRRRRRRRRRRRRRRRRRRRRRRRRRRRRRRRRRRRRRRRRRRRRRRRRRRRRRRR 540
Bhutan-09027        RRRRRRRRRRRRRRRRRRRRRRRRRRRRRRRRRRRRRRRRRRRRRRRRRRRRRRRRRRRR 540
Bhutan-09030        RRRRRRRRRRRRRRRRRRRRRRRRRRRRRRRRRRRRRRRRRRRRRRRRRRRRRRRRRRRR 540
Bhutan-09005        RARARRRRRRRRRRRRRRRRRRRRRRRRRRRRRRRRRRRRRRRGRRRRRGRRRGRRARRAG 540
Indonesia-88035     GRAAAGGAAGAAAGAGRRRRAAGAAAAGAAGGAAGAAGAAAAAARGGARRGARGARGGRR 540
Indonesia-88045     GRRAAGGAAGAAAGAGRRGGAAGAAAAGAAGGAAGAAGAAAAAAAGGARRGARGARGGRR 540
Indonesia-88065     RARRRRRRRRRRRRRRAGRGRRRRRRRRRRRRRRRRRRRRRRRRGRRRRRGGRRRRRRRAG 540


Bhutan-09015        RRRRWWWWWWWWWWWWWWWWWWWWWWWWWWWWWWWWWWWWWWWWWWWWWWWWWWWWWWWW 600
Bhutan-09024        RRRRWWWWWWWWWWWWWWWWWWWWWWWWWWWWWWWWWWWWWWWWWWWWWWWWWWWWWWWW 600
Bhutan-09027        RRRRWWWWWWWWWWWWWWWWWWWWWWWWWWWWWWWWWWWWWWWWWWWWWWWWWWWWWWWW 600
Bhutan-09030        RRRRWWWWWWWWWWWWWWWWWWWWWWWWWWWWWWWWWWWWWWWWWWWWWWWWWWWWWWWW 600
Bhutan-09005        RRRRWWWWWWWWWWWWWWTTTTAWATAWWWWWWTTTAAWWATWWAWWWWWTWTWWWTWTWW 600
Indonesia-88035     GAAAWTTTTTATTTTWTWAWTWWWWWAAAATAWAWWTATWWATWAAWAWWTWTAAWTWAW 600
Indonesia-88045     GAAAATTTTTATTTTATWAWTWTAWWAAWAWAWAWWTATWWATWAAAAWWTWTAAWTWAT 600
Indonesia-88065     RRRRWWWWWWWWWWWWWWTTTWATATAAWWWWWTTTAAWWATWWAWWWWTTWTWWWTWWWW 600


Bhutan-09015        WWWWWWWWWWWWWWWWWWWWWWWWWWWWWWWWWWWWWWWWWWWWWWWWWWWWWWWWWWWW 660
Bhutan-09024        WWWWWWWWWWWWWWWWWWWWWWWWWWWWWWWWWWWWWWWWWWWWWWWWWWWWWWWWWWWW 660
Bhutan-09027        WWWWWWWWWWWWWWWWWWWWWWWWWWWWWWWWWWWWWWWWWWWWWWWWWWWWWWWWWWWW 660
```

```
Bhutan-09030        WWWWWWWWWWWWWWWWWWWWWWWWWWWWWWWWWWWWWWWWWWWWWWWWWWWWWWWWWWWW 660
Bhutan-09005        TAWTWWWWAWWAWWWAWAWWWTTAWWWWAWWWWWWWWWWWTTAATWATWWWWWWWTTTAW 660
Indonesia-88035     WWAWTAWTWTAWWTAWAWWTTWWWWTATTATTTAAATWWWWWWTWTWWTTTTATWWAWWW 660
Indonesia-88045     WWAWTATTWTAWTTAWWWWTTWWWATATTAWTTAAWWATTWWWTWTWWTTWTATTWAWWT 660
Indonesia-88065     WAWTWWWWAWWAWWWAAWAWWTTAWWWWAWWWWWWWWWWTWTTAATWATWWWWWWWTTTAA 660

Bhutan-09015        WWWWWWWWWWWWWWWWWWWWWWWWWWWWWWWWWWWWWWWWWWWWWWWWWWWWWWWWWWWW 720
Bhutan-09024        WWWWWWWWWWWWWWWWWWWWWWWWWWWWWWWWWWWWWWWWWWWWWWWWWWWWWWWWWWWW 720
Bhutan-09027        WWWWWWWWWWWWWWWWWWWWWWWWWWWWWWWWWWWWWWWWWWWWWWWWWWWWWWWWWWWW 720
Bhutan-09030        WWWWWWWWWWWWWWWWWWWWWWWWWWWWWWWWWWWWWWWWWWWWWWWWWWWWWWWWWWWW 720
Bhutan-09005        WWWWWWWWWWWWWWWWWWWWWWWWWWWWWAWWWWWAAWWWWWWWWWTWWWWWWWWWWWWWWWTWA 720
Indonesia-88035     TAAAATTTATAATTAAATTATAATTAWTAAATTWAATTTAAATTTAAAWTTAATTATATW 720
Indonesia-88045     TAAAATTTATAATTWAWTTATAATTAWTAAATTWAATTTAAATWTAAWATTAATTATATA 720
Indonesia-88065     WWWWWWWWWWWWWWWAAWWWWWWWWWWAWWWWWAAWWWWWWWWWWWWWWWWWWWWWWTWTWA 720

Bhutan-09015        WWWWWSSSSSSSSSSSSSSSSSSSSSSSSSSSSSSSSSSSSSSSSSSSSSSSSSSSSSS 780
Bhutan-09024        WWWWWSSSSSSSSSSSSSSSSSSSSSSSSSSSSSSSSSSSSSSSSSSSSSSSSSSSSSS 780
Bhutan-09027        WWWWWSSSSSSSSSSSSSSSSSSSSSSSSSSSSSSSSSSSSSSSSSSSSSSSSSSSSSS 780
Bhutan-09030        WWWWWSSSSSSSSSSSSSSSSSSSSSSSSSSSSSSSSSSSSSSSSSSSSSSSSSSSSSS 780
Bhutan-09005        WWWWWCCSSSSSSSCSCCGSCSGSSCGGSCGSSSGSSSCSSGSSSSSSCCSSSSSSCSSC 780
Indonesia-88035     TTTTAGSGGCGCCSGSSSSGSCCGSSSSGSSCCSSCCCSCCCCGCGCCSSGGCCGSSGCS 780
Indonesia-88045     TWTTAGSGGCGCCGGGSSSGSCCGCSSSGSSCCSSCSSSCCCCGCGCCSSGGCCGCSGSS 780
Indonesia-88065     WWWWWCCSSSSSSGCSCCGSCSGSSCGGSCGSSCGSSSCSSGSSSSSSCCSSSSSSCSCC 780
                          ........ ....... .................. ..................

Bhutan-09015        SSSSSSSSSSSSSSSSSSSSSSSSSSSSSSSSSSSSSSSSSSSSSSSSSSSSSSSSSSSS 840
Bhutan-09024        SSSSSSSSSSSSSSSSSSSSSSSSSSSSSSSSSSSSSSSSSSSSSSSSSSSSSSSSSSSS 840
Bhutan-09027        SSSSSSSSSSSSSSSSSSSSSSSSSSSSSSSSSSSSSSSSSSSSSSSSSSSSSSSSSSSS 840
Bhutan-09030        SSSSSSSSSSSSSSSSSSSSSSSSSSSSSSSSSSSSSSSSSSSSSSSSSSSSSSSSSSSS 840
Bhutan-09005        CSCSSSSSGCGSCSCSSSGCGGSSSSSSSSSSSSCSSSSCSSSSSCSGCSSSSSSSSSSSS 840
Indonesia-88035     SCSSGGCCSSSCSCSGCCSSSSGGCGCCGCGGGSCCCCSCGCGGSCSGCSCCGSCCCGGC 840
Indonesia-88045     SCSCGGCCSSSCSCSGSCSSSSSGCGCCGCGGSSCCCCSCGCGGSCSGCSCCGSCSSGGC 840
Indonesia-88065     CSCSSSSSGCGSCSCSSSGCGGSSSSSSSSSSSSSCSSSSCSSSSSCSGCSCSSSGSSSSSS 840
                    .............................................. .............

Bhutan-09015        SSSSYYYYYYYYYYYYYYYYYYYYYYYYYYYYYYYYYYYYYYYYYYYYYYYYYYYYYYYY 900
Bhutan-09024        SSSSYYYYYYYYYYYYYYYYYYYYYYYYYYYYYYYYYYYYYYYYYYYYYYYYYYYYYYYY 900
Bhutan-09027        SSSSYYYYYYYYYYYYYYYYYYYYYYYYYYYYYYYYYYYYYYYYYYYYYYYYYYYYYYYY 900
Bhutan-09030        SSSSYYYYYYYYYYYYYYYYYYYYYYYYYYYYYYYYYYYYYYYYYYYYYYYYYYYYYYYY 900
Bhutan-09005        SSSSYCYYYYTCYYTTYYCYYYYYYYTYTYYYYYYYYYYYYCYTTCYYCTTTYYYTYTCY 900
Indonesia-88035     GGGGYCYCTTCYTCCYCTCTCTCCCTYCCCCTTTCCTCCTCYCYYTYTYYYTCYYYTYYT 900
Indonesia-88045     GSGGTYYCYTCYTCCYCTCTCTCCCTYCCCCTTTCCTCCTYYYYYTTTYYYTCTTYTYYT 900
Indonesia-88065     SSSSYCTYYYTCYYTTYYYYYYYYYYTYTYYYYYYYYYYTYYYYYCYYCTTYYTTTYTCY 900
                    ....

Bhutan-09015        YYYYYYYYYYYYYYYYYYYYYYYYYYYYYYYYYYYYYYYYYYYYYYYYYYYYYYYYYYYY 960
Bhutan-09024        YYYYYYYYYYYYYYYYYYYYYYYYYYYYYYYYYYYYYYYYYYYYYYYYYYYYYYYYYYYY 960
Bhutan-09027        YYYYYYYYYYYYYYYYYYYYYYYYYYYYYYYYYYYYYYYYYYYYYYYYYYYYYYYYYYYY 960
Bhutan-09030        YYYYYYYYYYYYYYYYYYYYYYYYYYYYYYYYYYYYYYYYYYYYYYYYYYYYYYYYYYYY 960
Bhutan-09005        YTYTTYYTCYTTYCTYTYYYTCYTTTYYTYYYYYTCYYYCTTCTYYYYCYYYCCYYCCYY 960
Indonesia-88035     CYTYCCCYCCCYTYYCYYCYYYYYYCYCYCCYYTYTTTCTCYYYCTTYYCYYYYCTYYCC 960
Indonesia-88045     CYTYCCYYYCCYYYYCYTYCYYCYYCYYYYCTCTYTTYCTCYYYCTTCYCCYYYCTYYCC 960
```

```
Indonesia-88065      YTYTTYCTCYTTYCTYTYYYTCYTTTCYTYYYCYTCYYYCTTCYYTYYCYYTCYYYYYYY 960

Bhutan-09015         YYYYYYYYYYYYYYYYYYYYYYYYYYYYYYYYYYYYYYYYYYYYYYYYYYYYYYYYYYYY 1020
Bhutan-09024         YYYYYYYYYYYYYYYYYYYYYYYYYYYYYYYYYYYYYYYYYYYYYYYYYYYYYYYYYYYY 1020
Bhutan-09027         YYYYYYYYYYYYYYYYYYYYYYYYYYYYYYYYYYYYYYYYYYYYYYYYYYYYYYYYYYYY 1020
Bhutan-09030         YYYYYYYYYYYYYYYYYYYYYYYYYYYYYYYYYYYYYYYYYYYYYYYYYYYYYYYYYYYY 1020
Bhutan-09005         YYYCCTYYYYTYYYYYCYCYTTYYCYYYYTCTYYCTCYCYCTYCTYYYYCTCTYYYYYYY 1020
Indonesia-88035      YCTTYCTCTTYCCCTYYCYCTYCTYTTYCYYYTCTCYTYCYYYYCCCTTTYYYTTCTTTY 1020
Indonesia-88045      TYTTYCTCTTYCCCTCYCYCYYCTYYYTCYYYTCTCYTYCYYCYCCCTTTYYYTYCTTTT 1020
Indonesia-88065      YYYCCTYYYYYYYYYYCYYYTYYTCYYYYYYTYYCTYTCYCTYCTYYYYCTCTYYYYYYY 1020

Bhutan-09015         YYYYYYYYYYYYYYYYYYYYYYYYYYYYYYYYYYYYYYYYYYYYYYYYYYYYYYYYYYYY 1080
Bhutan-09024         YYYYYYYYYYYYYYYYYYYYYYYYYYYYYYYYYYYYYYYYYYYYYYYYYYYYYYYYYYYY 1080
Bhutan-09027         YYYYYYYYYYYYYYYYYYYYYYYYYYYYYYYYYYYYYYYYYYYYYYYYYYYYYYYYYYYY 1080
Bhutan-09030         YYYYYYYYYYYYYYYYYYYYYYYYYYYYYYYYYYYYYYYYYYYYYYYYYYYYYYYYYYYY 1080
Bhutan-09005         YCCYCTYYYYYYCYYYTYTCYTYCYYYYTYTYYTYCCYYYYYTYYYYYYYYYYYTYYYYYC 1080
Indonesia-88035      TYYTYYYYYYTCYYCCYTYYTCCYCCCYYCTYCCTYTCTCTTYYCCYYYCCYYYCYYCCY 1080
Indonesia-88045      TYYTYYCCYYTCYCCCYTYYTCCYCCCTYYYTCCTYTCTCYTYTCCTTTCCTYYCCCCCY 1080
Indonesia-88065      YCCYYYYYCCYYCYYYTYTCYTYCCCCTTYYYTTYCCYYYYYTYCCYYYYCYCYCCYYYY 1080

Bhutan-09015         YYYYYYYYYYYYYYYYYYYYYYYYYYYYYYYYYYYYYYYYYYYYYYYYYYYYYYYYYYYY 1140
Bhutan-09024         YYYYYYYYYYYYYYYYYYYYYYYYYYYYYYYYYYYYYYYYYYYYYYYYYYYYYYYYYYYY 1140
Bhutan-09027         YYYYYYYYYYYYYYYYYYYYYYYYYYYYYYYYYYYYYYYYYYYYYYYYYYYYYYYYYYYY 1140
Bhutan-09030         YYYYYYYYYYYYYYYYYYYYYYYYYYYYYYYYYYYYYYYYYYYYYYYYYYYYYYYYYYYY 1140
Bhutan-09005         TYTTTYTCYYYCTTTTYTCYTYCCYYTTTTTYYYCCCTTTYYCYTYYCCCCTYYYYCYYY 1140
Indonesia-88035      YTYYCCCCYTYYYYYYCYYCYTYTCTYYYYYYTTYYYYYYCCYTYCTTYYYYTTTTYTTT 1140
Indonesia-88045      YTYYCCCYYTYYYYYYCYYCYTYTYTYYYYYYTTYYYYYYYCYYTYYTTYYYYTTTTYTTT 1140
Indonesia-88065      TYTTTYTYCYCCTTTTYTCYYYCCCYTYYYYYYYCCCTTTYYCYTYYCCYCTYYYYYYYY 1140

Bhutan-09015         YYYYYYYYYYYYYYYYYYYYYYYYYYYYYYYYYYYYYYYYYYYYYYYYYYYYYYYYYYYY 1200
Bhutan-09024         YYYYYYYYYYYYYYYYYYYYYYYYYYYYYYYYYYYYYYYYYYYYYYYYYYYYYYYYYYYY 1200
Bhutan-09027         YYYYYYYYYYYYYYYYYYYYYYYYYYYYYYYYYYYYYYYYYYYYYYYYYYYYYYYYYYYY 1200
Bhutan-09030         YYYYYYYYYYYYYYYYYYYYYYYYYYYYYYYYYYYYYYYYYYYYYYYYYYYYYYYYYYYY 1200
Bhutan-09005         YYYCYYCYYYYYYYYYYTYYYYYCTTYYYYCYYYYYYYYYYYYYYYYYYYYYYYYYYYYT 1200
Indonesia-88035      TCCTTCYCTTTCTCCCTYTCTYTYYYTCTCTTTTCCCTTTCTTCCCCTTTTTCCCTTCTY 1200
Indonesia-88045      TCCTTCYCTTTCTCCCTYTCTYYTYYTCTCTTTTCCCTTTCTTCCCCTTTTTCCCTTCTY 1200
Indonesia-88065      YYCYYYCYYYYYYYYYYTYYYTYCTTYYYYCYYYYYYYYYYYYYYYYYYYYYYYYYYYYY 1200

Bhutan-09015         YYYYYYYYYYYYYYYYYYYYYYYYYYYYYYYYYYYYYYYYYYYYYYYYYYYYYYYYYYYY 1260
Bhutan-09024         YYYYYYYYYYYYYYYYYYYYYYYYYYYYYYYYYYYYYYYYYYYYYYYYYYYYYYYYYYYY 1260
Bhutan-09027         YYYYYYYYYYYYYYYYYYYYYYYYYYYYYYYYYYYYYYYYYYYYYYYYYYYYYYYYYYYY 1260
Bhutan-09030         YYYYYYYYYYYYYYYYYYYYYYYYYYYYYYYYYYYYYYYYYYYYYYYYYYYYYYYYYYYY 1260
Bhutan-09005         CYYTTCYYYYTYTYYYYYYYYYYYYYYYYYYYYYYYCYYYYCTYYYYYCYYYYYYYYYYY 1260
Indonesia-88035      YTTYCTTCCTYCYCCCCTCTTTCCTTTTYYYCCCCYTCCYYYCCCCTTYTTYCCCYCCTY 1260
Indonesia-88045      YTTYCTTCCTYYCYCCCTCTTTCCTTTTYCTCCCCYTCCYYYCCCCTTTTTCCCCYCCTT 1260
Indonesia-88065      CYYTTCYYYYTYTYYYYYYYYYYYYYYYTYYYYYCYYYCCTYYYYYCTYYYYYYTYCYY 1260

Bhutan-09015         YYYYYYYYYYYYYYKKKKKKKKKKKKKKKKKKKKKKKKKKKKKKKKKKKKKKKKKKKKKKKK 1320
```

```
Bhutan-09024         YYYYYYYYYYYYYYYKKKKKKKKKKKKKKKKKKKKKKKKKKKKKKKKKKKKKKKKKKKKK 1320
Bhutan-09027         YYYYYYYYYYYYYYYKKKKKKKKKKKKKKKKKKKKKKKKKKKKKKKKKKKKKKKKKKKKK 1320
Bhutan-09030         YYYYYYYYYYYYYYYKKKKKKKKKKKKKKKKKKKKKKKKKKKKKKKKKKKKKKKKKKKKK 1320
Bhutan-09005         YYYYYYYYYYYYYYYGKGGKKKKKKKKKKKGGTKKKGGGKGKKTTKTGTKKTKKKTGGTG 1320
Indonesia-88035      TYCCCTTTTTCTTTGTKKGGKTGTTTGTGKKKKGGTKKKKKTTKKGKKKGTKGTGKKKKK 1320
Indonesia-88045      TTCCCTTTTTCTTTKKKKGGTTGTTTGTGTKKKGKKKKKTKTTKKGTKKGKGGKGKGKKK 1320
Indonesia-88065      YYYYYYYYYYYYYYYGKGGKKKKKKKKKKKKGTKGKGGGKKKKTTKTGTKKTKKKTKGTG 1320
                                    .    .. ... . .  .  .   . .... . . .  . .  .

Bhutan-09015         KKKKKKKKKKKKKKKKKKKKKKKKKKKKKKKKKKKKKKKKKKKKKKKKKKKKKKKKKKKK 1380
Bhutan-09024         KKKKKKKKKKKKKKKKKKKKKKKKKKKKKKKKKKKKKKKKKKKKKKKKKKKKKKKKKKKK 1380
Bhutan-09027         KKKKKKKKKKKKKKKKKKKKKKKKKKKKKKKKKKKKKKKKKKKKKKKKKKKKKKKKKKKK 1380
Bhutan-09030         KKKKKKKKKKKKKKKKKKKKKKKKKKKKKKKKKKKKKKKKKKKKKKKKKKKKKKKKKKKK 1380
Bhutan-09005         KTKKKGGKKGKKKKKKKKKKGKGGGKKKGKTKKKKKKKGKTKKKKKGGGGGKKKKTKKTK 1380
Indonesia-88035      TKGGGKKKGKTTGTTTTGGGGTKKKKTGTTKTTTGTGGKGKKKTGTKKTKKGTGTKGKKG 1380
Indonesia-88045      TKGGKKKGGKTTGTTTTGGGKTKKKKTGTTKTTTGTKGKGKTTTKTKKKKKGTGTKGKKG 1380
Indonesia-88065      KTKKKKKKKGKKKKKKKKKKGKGKGTKKGKTKKKKKKKGKTKTKKKGGGGGKKKKTKGTK 1380
                     ..        .. ....    .   ..  ..... .    .... .      . ..  .

Bhutan-09015         KKKKKKKKKKKKKKKKKKKKKKKKKKKKKKKKKKKKKKKKKKKKKKKKKKKK 1432
Bhutan-09024         KKKKKKKKKKKKKKKKKKKKKKKKKKKKKKKKKKKKKKKKKKKKKKKKKKKK 1432
Bhutan-09027         KKKKKKKKKKKKKKKKKKKKKKKKKKKKKKKKKKKKKKKKKKKKKKKKKKKK 1432
Bhutan-09030         KKKKKKKKKKKKKKKKKKKKKKKKKKKKKKKKKKKKKKKKKKKKKKKKKKKK 1432
Bhutan-09005         KKKKKKKKKKKKKKKKKKKKKKKKTKGKKKKKKKTKKKKKKKKKKKKKKKKK 1432
Indonesia-88035      GKTTTTGTGTTGGTGTTTGTTTKKKTKGTGTTGGGKTTGGTGTTKTGTTGGG 1432
Indonesia-88045      GTTTTTGTGTTGGTGTTTGTTTTKKTKGTGTTGGGKTTGGTGKTTTGKTGGG 1432
Indonesia-88065      KKKKKKKKKKKKKKKKKKKKKKTTTKKKKKKKKKKKKKKKKKKKKKKTKKKK 1432
                      ..... . ..  . ... .......  . ..   ...  . .... ..
```

Supplementary Figure S4.

```
CLUSTAL 2.1 multiple sequence alignment


Bhutan-09015        MMCMMMMMGGGRRGRGRRRRRAGRRRGGAARRAGRRRRRRRRRRARAGRRRRGWWWWTWW 60
Bhutan-09024        AMCMAMMCGRGARRRRGGRRRARRRRRGRARRAGRRGRRRGGRGRRRGRRRRGTWTWWTW 60
Bhutan-09027        MAMMACCMGRRRARRRRGARGRRRRARGRRARRRAGRGARRGARARRRRGAGGWWWATWT 60
Bhutan-09030        MMMAMMMMRRRARRARRGRGRARAARRRRRRGRGRRRRAARGARAARRARRRRWTWWWWT 60
Bhutan-09005        MMMMMMMMRRRRRRRRRRRRRRRRRRRRRRRRRRRRRRRRRRRRRRRRRRRRRWWWWWWW 60
Indonesia-88035     MMMMMMMMRRRRRRRRRRRRRRRRRRRRRRRRRRRRRRRRRRRRRRRRRRRRRWWWWWWW 60
Indonesia-88045     MMMMMMMMRRRRRRRRRRRRRRRRRRRRRRRRRRRRRRRRRRRRRRRRRRRRRWWWWWWW 60
Indonesia-88065     MMMMMMMMRRRRRRRRRRRRRRRRRRRRRRRRRRRRRRRRRRRRRRRRRRRRRWWWWWWW 60


Bhutan-09015        AACCSCCSSSSSSSCSYYYYYYYTYYYYTYYYTYTYCYYCTTYYYYYYYYTCYYTYCCCY 120
Bhutan-09024        AWSSSSSSGGCSSCCCYTYYYTYTYYYTYYYTYTYYYYYCYTYCTTYYYCTCYYYYYCCC 120
Bhutan-09027        WWSSSSSCGSSSGSSSTTCCTTCYYCCYTTCYYYYTYCTYYYTYYYCYTYTYYYYYYYYC 120
Bhutan-09030        AWSSCCSSSSSCSSSSYTCCYTYYTYYYTTYYTYYYYCTCYYYYYYCTYYYYTCYCYCYY 120
Bhutan-09005        WWSSSSSSSSSSSSSSYYYYYYYYYYYYYYYYYYYYYYYYYYYYYYYYYYYYYYYYYYYY 120
Indonesia-88035     WWSSSSSSSSSSSSSSYYYYYYYYYYYYYYYYYYYYYYYYYYYYYYYYYYYYYYYYYYYY 120
Indonesia-88045     WWSSSSSSSSSSSSSSYYYYYYYYYYYYYYYYYYYYYYYYYYYYYYYYYYYYYYYYYYYY 120
Indonesia-88065     WWSSSSSSSSSSSSSSYYYYYYYYYYYYYYYYYYYYYYYYYYYYYYYYYYYYYYYYYYYY 120
                      ..............

Bhutan-09015        TTKKKTKKKKGGKG 134
Bhutan-09024        YYKGKTTKGTGKKG 134
Bhutan-09027        YTKKTKKTKKKKTG 134
Bhutan-09030        TTTGKKKTKKKGTK 134
Bhutan-09005        YYKKKKKKKKKKKK 134
Indonesia-88035     YYKKKKKKKKKKKK 134
Indonesia-88045     YYKKKKKKKKKKKK 134
Indonesia-88065     YYKKKKKKKKKKKK 134
                      . .... .  .
```

Supplementary Figure S5.

```
CLUSTAL 2.1 multiple sequence alignment



Bhutan-09015          AAAAAAAAAAAAAAAAAAAAAAAAAAAAAAAAAAAAAAAAAAAAAAAAAAAAAAAAAAAA 60
Bhutan-09024          AAAAAAAAAAAAAAAAAAAAAAAAAAAAAAAAAAAAAAAAAAAAAAAAAAAAAAAAAAAA 60
Bhutan-09027          AAAAAAAAAAAAAAAAAAAAAAAAAAAAAAAAAAAAAAAAAAAAAAAAAAAAAAAAAAAA 60
Bhutan-09030          AAAAAAAAAAAAAAAAAAAAAAAAAAAAAAAAAAAAAAAAAAAAAAAAAAAAAAAAAAAA 60
Bhutan-09005          AMAMAMMMMMAMMAMAMMMAMMMMMMMMAAMMMMMMMAMMMMMMCAMAMMMMMAMMCAA 60
Indonesia-88035       MCMAMAAAAAAMACMAMMACMMACCMAAMMMAAMAAAAMAAMAMAMMCMCAAAAMAAMMM 60
Indonesia-88045       MMMAMAAAAAAMAMMAMAACMMAMMMAAAMMAAMAAAAMAAMAAAMMCMCAAMAMAAMMM 60
Indonesia-88065       AMAMAMMMMMAMMAMAMMMMAAMMAAMMAAMAAMMMMAMMAMMMCAMAMMMMMAMMCAA 60


Bhutan-09015          AAAAAAAAAAAAAAAAAAAAAAAAAAAAAAAAAAAAAAAAAAAAAAAAAAAAAAAAAAAA 120
Bhutan-09024          AAAAAAAAAAAAAAAAAAAAAAAAAAAAAAAAAAAAAAAAAAAAAAAAAAAAAAAAAAAA 120
Bhutan-09027          AAAAAAAAAAAAAAAAAAAAAAAAAAAAAAAAAAAAAAAAAAAAAAAAAAAAAAAAAAAA 120
Bhutan-09030          AAAAAAAAAAAAAAAAAAAAAAAAAAAAAAAAAAAAAAAAAAAAAAAAAAAAAAAAAAAA 120
Bhutan-09005          MMAAMMAMMMMMMMAMAAAMAMMMCMMMCMMCCMMMMAMMMMMMMMMMMMMMMMMMMMCM 120
Indonesia-88035       AAMMACAAAAAAMAAAMMMCAAAAMCMAMAAMMAAACMAAACAAAAAAMAACAAAAAAAMA 120
Indonesia-88045       AAMMACMAAAAAAAAAAMMMMMAAAMCCAMAAMMAAACMAAACAAAAAACAACAAAAAAAMA 120
Indonesia-88065       MMAMMMAMMMMMAMMMAAMMAMAMCMMMCAAMMMAMMAMMMMMMMMMMMCMMMMMMMMMCM 120


Bhutan-09015          AAAAAAAAAAAAAAAAAAAAAAAAAAAAAAAAAAAAAAAACCCCCCCCCCCCCCCCCCCC 180
Bhutan-09024          AAAAAAAAAAAAAAAAAAAAAAAAAAAAAAAAAAAAAAAACCCCCCCCCCCCCCCCCCCC 180
Bhutan-09027          AAAAAAAAAAAAAAAAAAAAAAAAAAAAAAAAAAAAAAAACCCCCCCCCCCCCCCCCCCC 180
Bhutan-09030          AAAAAAAAAAAAAAAAAAAAAAAAAAAAAAAAAAAAAAAACCCCCCCCCCCCCCCCCCCC 180
Bhutan-09005          MAMMMMMMMMMMAMMMMMMAMMMMMAMMMMAAMMMMMMMMCMMMMMMMMMMMMMMMCMMM 180
Indonesia-88035       AMAAACAMAAAMMACAMAMAAAAAMMAAAAAAAACAAAACACAACMCACCCCCCACCMCAC 180
Indonesia-88045       AMAAAMAMAAAMMACAMACAMAAAMMAAAAMMAMAAAACAMAACMCACCCCCCACCMCAC 180
Indonesia-88065       MAMMMMMCAMMCCMMMAMMMAMMMAAMMMMAACMMMMMCMCAAMCMMMMMMMMMMMMCMM 180


Bhutan-09015          CCCCCCCCCCCCCCCCCCCCCCCCCCCCCCCCCCCCCCCCCCCCCCCCCCCCCCCCCCCC 240
Bhutan-09024          CCCCCCCCCCCCCCCCCCCCCCCCCCCCCCCCCCCCCCCCCCCCCCCCCCCCCCCCCCCC 240
Bhutan-09027          CCCCCCCCCCCCCCCCCCCCCCCCCCCCCCCCCCCCCCCCCCCCCCCCCCCCCCCCCCCC 240
Bhutan-09030          CCCCCCCCCCCCCCCCCCCCCCCCCCCCCCCCCCCCCCCCCCCCCCCCCCCCCCCCCCCC 240
Bhutan-09005          CCMMMMMMCMCMCMAMMMMMMMCMMCMACMCAMMMMCCMCMCAMMMCMCACCMCMCMCMM 240
Indonesia-88035       MMCCACCAMMCAMCMCMAAACCMMCCMMMCMMACMMMMCMCCMMCCCCMAMCCMCMCMCA 240
Indonesia-88045       MMCCAMCAMMMAMCMCMAAACCMACCAMMCMMMCMMMMCMCMMMCCCCMAMMMMCMMCCA 240
Indonesia-88065       CMMMMMMMCCCMCMAMCMMMMMCMMMMACMCAMMAACCMCMCACMMMCCMCMMCMCMCCM 240


Bhutan-09015          CCCCCCCCCCCCCCCCCCCCCCCCCCCCCCCCCCCCCCCCCCCCCCCCCCCCCCCCCCCC 300
Bhutan-09024          CCCCCCCCCCCCCCCCCCCCCCCCCCCCCCCCCCCCCCCCCCCCCCCCCCCCCCCCCCCC 300
Bhutan-09027          CCCCCCCCCCCCCCCCCCCCCCCCCCCCCCCCCCCCCCCCCCCCCCCCCCCCCCCCCCCC 300
Bhutan-09030          CCCCCCCCCCCCCCCCCCCCCCCCCCCCCCCCCCCCCCCCCCCCCCCCCCCCCCCCCCCC 300
Bhutan-09005          CMMMMCCCMMMMMMMMMMAMCCACAMMCMMCCCMMMMMCMMMMMMMMMMMMMMCMMMMCCMM 300
Indonesia-88035       MCCCCMMMMCAMCCCCCACMMMMACCMCCMMMCCCCMCCCCCCCCCCCCCMCCCCMMCC 300
Indonesia-88045       MCCCCMMMCCAACCCCCMCMMMMACCMCCMMMCCCCCCCCCCCCCCCCMCCMMMCCMCCC 300
Indonesia-88065       CMCMCCCCMMMMMMMMMMMMCCACMMMCMMCCCMMMMMCMMMMMMMMMMMMMMMMMMMMCMM 300
```

```
Bhutan-09015         CCCCCCCCCCCCCAAAAAAAAAAAAAAAAAAAAAAAAAAAAAAAAAAAAAAAAAAAAAA 360
Bhutan-09024         CCCCCCCCCCCCCAAAAAAAAAAAAAAAAAAAAAAAAAAAAAAAAAAAAAAAAAAAAAA 360
Bhutan-09027         CCCCCCCCCCCCCAAAAAAAAAAAAAAAAAAAAAAAAAAAAAAAAAAAAAAAAAAAAAA 360
Bhutan-09030         CCCCCCCCCCCCCAAAAAAAAAAAAAAAAAAAAAAAAAAAAAAAAAAAAAAAAAAAAAA 360
Bhutan-09005         CMMMMCMMMMMMMRAAAAAARRRRRRRARRRRARAARRRRRRRARRRRRRGRARRGRRAR 360
Indonesia-88035      MCCCCMCCCCCCCAAARRRRRAGGARRRRRAARARRAAGAAAGRAAAAAAARRRRRRGRAA 360
Indonesia-88045      MCCCCMCCCCCCCRAARRRRAARRARGRGRAARARRAAGAAAGRAAAAAARAARRRRGRA 360
Indonesia-88065      MMMMMMMMCMMMMRRRARAARARRRGRARARRARAARRARRRRARRRRRRGAAAARRRRR 360


Bhutan-09015         AAAAAAAAAAAAAAAAAAAAAAAAAAAAAAAAAAAAAAAAAAAAAAAAAAAAAAAAAAAA 420
Bhutan-09024         AAAAAAAAAAAAAAAAAAAAAAAAAAAAAAAAAAAAAAAAAAAAAAAAAAAAAAAAAAAA 420
Bhutan-09027         AAAAAAAAAAAAAAAAAAAAAAAAAAAAAAAAAAAAAAAAAAAAAAAAAAAAAAAAAAAA 420
Bhutan-09030         AAAAAAAAAAAAAAAAAAAAAAAAAAAAAAAAAAAAAAAAAAAAAAAAAAAAAAAAAAAA 420
Bhutan-09005         RAGGARAAAAARRRRAAARRRRARRRAARAARRRRRRARGARRRAAARRRARGRAARRRR 420
Indonesia-88035      ARRRRRRRRRAGRAAARRRRAGRRGGRRGRRAAGRARRARRRAGRRRARARARGRRGAGG 420
Indonesia-88045      ARRRRGRRRRRGGRARRAAGARRAGGRRGRAAAGAARRRRRGAGRRRRAARARGRRGAGG 420
Indonesia-88065      RRGGARAAARAGGRRRRAAGRRARRRAARAARRRRRGARGRRRRAAARRRRRGRAARRRG 420


Bhutan-09015         AAAAAAAAAAAAAAAAAAAAAAAAAAAAAAAAAAAAAAAAAAAAAAAAAAAAAAAAAAAA 480
Bhutan-09024         AAAAAAAAAAAAAAAAAAAAAAAAAAAAAAAAAAAAAAAAAAAAAAAAAAAAAAAAAAAA 480
Bhutan-09027         AAAAAAAAAAAAAAAAAAAAAAAAAAAAAAAAAAAAAAAAAAAAAAAAAAAAAAAAAAAA 480
Bhutan-09030         AAAAAAAAAAAAAAAAAAAAAAAAAAAAAAAAAAAAAAAAAAAAAAAAAAAAAAAAAAAA 480
Bhutan-09005         AGRAARRARAGRRRRRRRRARRRRRRARRRRRRARRRARRRRAAGAAARRRRRRARARRR 480
Indonesia-88035      RRARRGGRAARGARAAAAARAAAAAGRRAGAAARAARARARGRARARRAARGRGRARAAA 480
Indonesia-88045      RRARRGGRARRRAGAAAAARAAAAAGRAAGAAARAAAAAAAARRRRRRRAAGRARRARAAA 480
Indonesia-88065      AGRAARRARAGRRRRRRRRRRRRRRRARRRRRRRRRRRRRGAAGARRRAGRRRARARRA 480


Bhutan-09015         AAAAAAAAAAAAAAAAAAAAAAAAAAAAAAAAAAAAAAAAAAAAAAAAAAAAAAAAAAAA 540
Bhutan-09024         AAAAAAAAAAAAAAAAAAAAAAAAAAAAAAAAAAAAAAAAAAAAAAAAAAAAAAAAAAAA 540
Bhutan-09027         AAAAAAAAAAAAAAAAAAAAAAAAAAAAAAAAAAAAAAAAAAAAAAAAAAAAAAAAAAAA 540
Bhutan-09030         AAAAAAAAAAAAAAAAAAAAAAAAAAAAAAAAAAAAAAAAAAAAAAAAAAAAAAAAAAAA 540
Bhutan-09005         RGRGRRARRRRRRRRRRRARARARRARRAAARGAAAARARARRRRRRRRRRRRARARRR 540
Indonesia-88035      ARGRARAGAGAGAAAGAAARARRRAAAGGRRRRGRRRRGRARAGRRGAARRGRAAARAGR 540
Indonesia-88045      ARGRARRGARAGARAGAARRARARAAAGGRRRRRRRRRRRARAGRRGAAAARGARARAGA 540
Indonesia-88065      RGRARARGRRRRRARRRRRARARARRRGGRAAGRAAAAGARARRGARRRRRRRRARAAGR 540


Bhutan-09015         AAAAAAAAAAAAAAAAAAAAAAAAAAAAAAAAAAAAAAAAAAAAAAAAAAAAAAAAAAAA 600
Bhutan-09024         AAAAAAAAAAAAAAAAAAAAAAAAAAAAAAAAAAAAAAAAAAAAAAAAAAAAAAAAAAAA 600
Bhutan-09027         AAAAAAAAAAAAAAAAAAAAAAAAAAAAAAAAAAAAAAAAAAAAAAAAAAAAAAAAAAAA 600
Bhutan-09030         AAAAAAAAAAAAAAAAAAAAAAAAAAAAAAAAAAAAAAAAAAAAAAAAAAAAAAAAAAAA 600
Bhutan-09005         RRRRAARAAARGARRRRRARRRGRGARAARARRRRRRRRARRRRRRRAAAAARARRRRR 600
Indonesia-88035      AGAARRGRRRARRGARAGRGGRRARRARRAAAAAAGAAGARAAAAGGARARRRGRGGAGG 600
Indonesia-88045      RRAARRGRRRARRGAGAGRGRGRARRAGRAAAAAARAAGARARRRGGARARRRRAGRRGG 600
Indonesia-88065      RRARRARAAARGRGRRRRAGRRRRGARAARRRRRARRRRRARAARRRRARAAARARRRRR 600


Bhutan-09015         AAAAAAAAAAAAAAAAAAAAAAAAAAAAAAAAAAAAAAAAAAAAAAAAAAAAAAAAAAAA 660
Bhutan-09024         AAAAAAAAAAAAAAAAAAAAAAAAAAAAAAAAAAAAAAAAAAAAAAAAAAAAAAAAAAAA 660
```

```
Bhutan-09027        AAAAAAAAAAAAAAAAAAAAAAAAAAAAAAAAAAAAAAAAAAAAAAAAAAAAAAAAAAAA 660
Bhutan-09030        AAAAAAAAAAAAAAAAAAAAAAAAAAAAAAAAAAAAAAAAAAAAAAAAAAAAAAAAAAAA 660
Bhutan-09005        RRRAARRRRRRRRRRRRRRRRRRRRRARRARRRARRRRRARRRRRRRRRRRRRRRRRRRR 660
Indonesia-88035     AAARRAAAAAGAARAGAAAAAGAAARRAGRRRARAAAGAAAAAAAAAAAAAAAAAAAAAA 660
Indonesia-88045     AAARRAAAAAGAAGAGAAAAAGAAAGRAGRRAARAAAGAAAAAAAAAAAAAAAAAAAAAA 660
Indonesia-88065     RRRAARAARRRRRRRRRRRRRRRRRGARRRGRRARRRARRRRRRRRRRRRRRRRRRRRRR 660


Bhutan-09015        AAAAAAAAAAAAAAAAAAAAAAAAAAAAAAAAAAAAAAAAAAAAAAAAAAAAAAAAAAAA 720
Bhutan-09024        AAAAAAAAAAAAAAAAAAAAAAAAAAAAAAAAAAAAAAAAAAAAAAAAAAAAAAAAAAAA 720
Bhutan-09027        AAAAAAAAAAAAAAAAAAAAAAAAAAAAAAAAAAAAAAAAAAAAAAAAAAAAAAAAAAAA 720
Bhutan-09030        AAAAAAAAAAAAAAAAAAAAAAAAAAAAAAAAAAAAAAAAAAAAAAAAAAAAAAAAAAAA 720
Bhutan-09005        RRRRRRRRGGAAAAAGRRRARRRARRRRAAAARRRRRRRRRRRRRAGRARRRRRARAARRR 720
Indonesia-88035     AARAAGRARRRRARARRAARAGARGAAAARRRRGAAAAAAAAAAARRRRAAAAAAAARRAAA 720
Indonesia-88045     AAGAAGGARRRRRRRRAAAARGARGAAARRRRRAAAAAAAAAAAARGRAAAARAARRAAA 720
Indonesia-88065     RRRRRRRRGGRRRRARRRRRRRRAGRRRAARRGRRRRRRRRRRRRARRRRRRRARRAARRA 720


Bhutan-09015        AAAAAAAAAAAAAAAAAAAAAAAAAAAAAAAAAAAAAAAAAAAAAAAAGGGGGGGGGGGG 780
Bhutan-09024        AAAAAAAAAAAAAAAAAAAAAAAAAAAAAAAAAAAAAAAAAAAAAAAAGGGGGGGGGGGG 780
Bhutan-09027        AAAAAAAAAAAAAAAAAAAAAAAAAAAAAAAAAAAAAAAAAAAAAAAAGGGGGGGGGGGG 780
Bhutan-09030        AAAAAAAAAAAAAAAAAAAAAAAAAAAAAAAAAAAAAAAAAAAAAAAAGGGGGGGGGGGG 780
Bhutan-09005        RRRRRRRRRRRARRRRRRRRGRRARRRRRRRRRRRRRRRRRRRRRRRRRGRGGARRRRRRG 780
Indonesia-88035     ARAARGRAAAARAAGAGRGARAARAAAAAGAAAAAAAAAAAGAAAAAAAARAGRRGGRRAAR 780
Indonesia-88045     AGAARGAAAAARAARAGGRARAARAARAARAAAAAAAAAAAGAAAAAAAGAGRAGGAAAAR 780
Indonesia-88065     RRRRARRRRRRRRRRRRGRARRRRRRARRRGRRRRRRRRRRRRRRRRRRGRRRRRRRRARG 780


Bhutan-09015        GGGGGGGGGGGGGGGGGGGGGGGGGGGGGGGGGGGGGGGGGGGGGGGGGGGGGGGGGGGG 840
Bhutan-09024        GGGGGGGGGGGGGGGGGGGGGGGGGGGGGGGGGGGGGGGGGGGGGGGGGGGGGGGGGGGG 840
Bhutan-09027        GGGGGGGGGGGGGGGGGGGGGGGGGGGGGGGGGGGGGGGGGGGGGGGGGGGGGGGGGGGG 840
Bhutan-09030        GGGGGGGGGGGGGGGGGGGGGGGGGGGGGGGGGGGGGGGGGGGGGGGGGGGGGGGGGGGG 840
Bhutan-09005        RGRRRRRRRRRRRRRRRRRRRRRRRGRGGGRRGGGAARGRRARRRRRRRRGAARRGRRGRG 840
Indonesia-88035     RRRGGGGGGGAAGGGGGGGGGGGGGGGRGRRRRRRRRGRGRRGRAGRGRGRRRARRRARGR 840
Indonesia-88045     ARGGGGGGGGAAGGGGGGGGGGGGGRRRRRRARRRRRGRGGAGAARGGGGRRRAGRGRRGR 840
Indonesia-88065     AGRRRRRRRRRRRRRRRRRRRRRRRRRRGGGRRGGRAGGRRARARRRGRGRAARRGRRGRG 840


Bhutan-09015        GGGGGGGGGGGGGGGGGGGGGGGGGGGGGGGGGGGGGGGGGGGGGGGGGGGGGGGGGGGG 900
Bhutan-09024        GGGGGGGGGGGGGGGGGGGGGGGGGGGGGGGGGGGGGGGGGGGGGGGGGGGGGGGGGGGG 900
Bhutan-09027        GGGGGGGGGGGGGGGGGGGGGGGGGGGGGGGGGGGGGGGGGGGGGGGGGGGGGGGGGGGG 900
Bhutan-09030        GGGGGGGGGGGGGGGGGGGGGGGGGGGGGGGGGGGGGGGGGGGGGGGGGGGGGGGGGGGG 900
Bhutan-09005        RGRGGRARGRGRRRAAGGRGGGRRGRARRGRGGRRGRGGGRGGRRRRRGGRRGRARRRR 900
Indonesia-88035     ARGRRARRRGRARGRRRRGRGRRRGRGRGGGGRRGGRARRRGRRGGGGGRRRRGGRGRGA 900
Indonesia-88045     ARGRRRRARGRARGRRRRGRGRRGGRGRGGGGRRGGRARRRGRRGGGGGGRGGRRRGGGR 900
Indonesia-88065     RGRGRRARGRGRARARRGRGRGGRRGRRRRRRGGRRGRGGGRGGRRRRRGGRGGRARRRR 900


Bhutan-09015        GGGGGGGGGGGGGGGGGGGGGGGGGGGGGGGGGGGGGGGGGGGGGGGGGGGGGGGGGGGG 960
Bhutan-09024        GGGGGGGGGGGGGGGGGGGGGGGGGGGGGGGGGGGGGGGGGGGGGGGGGGGGGGGGGGGG 960
Bhutan-09027        GGGGGGGGGGGGGGGGGGGGGGGGGGGGGGGGGGGGGGGGGGGGGGGGGGGGGGGGGGGG 960
Bhutan-09030        GGGGGGGGGGGGGGGGGGGGGGGGGGGGGGGGGGGGGGGGGGGGGGGGGGGGGGGGGGGG 960
Bhutan-09005        RRRRRRRRRRRGRGRRRRRGRGRGRRRARGRGGRRRRRRRGRGRRGRGGARAGAGRRRRR 960
Indonesia-88035     GGRGGRGAGGGGGGARGAGRGRRRGGRRARGGRGGGGGAARRGAGRRGRAGRRRRARAAG 960
```

```
Indonesia-88045      GGRGGAGAGGGRRRRGGAGRGRARGGARARGRRGGGRRAARGRAGRARRRGRRRRRRARG 960
Indonesia-88065      RRGRRRRRRRRRRGRRRRRGRGRGRGRRRGRGGRRRRRRRRRRGRRGRGGAGRGAGRGRRR 960

Bhutan-09015         GGGGGGGGGGGGGGGGGGGGGGGGGGGGGGGGGGGGGGGGGGGGGGGGGGGGGGGGGGGG 1020
Bhutan-09024         GGGGGGGGGGGGGGGGGGGGGGGGGGGGGGGGGGGGGGGGGGGGGGGGGGGGGGGGGGGG 1020
Bhutan-09027         GGGGGGGGGGGGGGGGGGGGGGGGGGGGGGGGGGGGGGGGGGGGGGGGGGGGGGGGGGGG 1020
Bhutan-09030         GGGGGGGGGGGGGGGGGGGGGGGGGGGGGGGGGGGGGGGGGGGGGGGGGGGGGGGGGGGG 1020
Bhutan-09005         GGRARRRGGRRRRRRRGGRRRAARRAGGGRGGRRRGGGGRGGRRRRARRGGRRGGRGRGR 1020
Indonesia-88035      RARRGGGRRGGGGGGGRRRRRRRRRRRRARRGAGRGRRARRRRRRRGGRRAGRRGRRRG 1020
Indonesia-88045      RRRRGGGRRRGGGGGRRRARARRRRRRRRARRGAGRGRRARRRRRARGGRRAGRRGRGRG 1020
Indonesia-88065      GGAARRRGGRRRRRRRGGAARAAGGAGGGRGGRRRGRGGRGGGGRARRGRRRGGRGRGR 1020

Bhutan-09015         GGGGGGGGGGGGGGGGGGGGGGGGGGGGGGGGGGGGGGGGGGGGGGGGGGGGGGGGGGGG 1080
Bhutan-09024         GGGGGGGGGGGGGGGGGGGGGGGGGGGGGGGGGGGGGGGGGGGGGGGGGGGGGGGGGGGG 1080
Bhutan-09027         GGGGGGGGGGGGGGGGGGGGGGGGGGGGGGGGGGGGGGGGGGGGGGGGGGGGGGGGGGGG 1080
Bhutan-09030         GGGGGGGGGGGGGGGGGGGGGGGGGGGGGGGGGGGGGGGGGGGGGGGGGGGGGGGGGGGG 1080
Bhutan-09005         RRGGRGRRGRRGGRGRGRRRRRAGGGRRGGRRGGRRRRRRRRRRRRRGRGGRRRRGGGGGG 1080
Indonesia-88035      ARGRGRAAGGGRRARGRRAGGGRRRRGGRRGGRRRGAAGGAGGGGGGGRRGGGGRRGRRR 1080
Indonesia-88045      AGRRRRAARGGRRARGRGAGGGRRRRGGGRGGRRAGAAGGAGGGGGRGRRGGRGRRRRRR 1080
Indonesia-88065      RRGGRGRRGRRGGRGGGRARRRAGGGRRGGRRGGRRRRRRRRRRRRRGRGGRRGRGGRGGG 1080

Bhutan-09015         GGGGGGGGGGGGGGGGGGGGGGGGGGGGGGGGGGGGGGGGGGGGGGGGGGGGGGGGGGGG 1140
Bhutan-09024         GGGGGGGGGGGGGGGGGGGGGGGGGGGGGGGGGGGGGGGGGGGGGGGGGGGGGGGGGGGG 1140
Bhutan-09027         GGGGGGGGGGGGGGGGGGGGGGGGGGGGGGGGGGGGGGGGGGGGGGGGGGGGGGGGGGGG 1140
Bhutan-09030         GGGGGGGGGGGGGGGGGGGGGGGGGGGGGGGGGGGGGGGGGGGGGGGGGGGGGGGGGGGG 1140
Bhutan-09005         GRRRRRGRGRRRRGRRRRRRRRGRRRRRRARRRRRRGRRRRRRGRRRRRRRRRRRRRGRR 1140
Indonesia-88035      RRRGGGRGRGAAGGGGGGGGGGGGGGGGGAGGAGGGGRGGGGGGGGGGGGGGGRGGGRGG 1140
Indonesia-88045      RRRGGGRGRGRRGGGGGGGGGGGGGGGGGRGGAGRGGRGGGGGGGGGGGGGGGGGGGRGG 1140
Indonesia-88065      GGGRRRGRGGARRRRRRRRRRRRRARRRRRRRRRRRGRRRRRRRRRRRRRRRRRRRRGRR 1140

Bhutan-09015         GGGGGGGGGGGGGGGGGGGGGGGGGGGGGGGGGGGGGGGGGGGGGGGGGGGGGGGGGGGG 1200
Bhutan-09024         GGGGGGGGGGGGGGGGGGGGGGGGGGGGGGGGGGGGGGGGGGGGGGGGGGGGGGGGGGGG 1200
Bhutan-09027         GGGGGGGGGGGGGGGGGGGGGGGGGGGGGGGGGGGGGGGGGGGGGGGGGGGGGGGGGGGG 1200
Bhutan-09030         GGGGGGGGGGGGGGGGGGGGGGGGGGGGGGGGGGGGGGGGGGGGGGGGGGGGGGGGGGGG 1200
Bhutan-09005         RRRRRRRGRRRGGGARGGGRRRRRRRRRGRRRRRRAGGGRRRRRAAARRRRRRAARRARRA 1200
Indonesia-88035      GGGGGGGRGAGRRRRRRRRGGGGGGGGGGGGRGRRRRRGGGGGRGGGGGGAARAGGARAR 1200
Indonesia-88045      GGGGGGGRGAGRRRRARRRGGGGGGGGRGGGGRGRRRRGGGGGRGGGGGGAARRGGAGAR 1200
Indonesia-88065      RRRRRRRGGARRRRRRRGRGRRRRRRRRRGRRRRRRRGGGGGRRRARRRRRRRRRRRRRRR 1200

Bhutan-09015         GGGGGGGGGGGGGGGGGGGGGGGGGGGGGGGGGGGGGGGGGGGGGGGGGGGGGGGGGGGG 1260
Bhutan-09024         GGGGGGGGGGGGGGGGGGGGGGGGGGGGGGGGGGGGGGGGGGGGGGGGGGGGGGGGGGGG 1260
Bhutan-09027         GGGGGGGGGGGGGGGGGGGGGGGGGGGGGGGGGGGGGGGGGGGGGGGGGGGGGGGGGGGG 1260
Bhutan-09030         GGGGGGGGGGGGGGGGGGGGGGGGGGGGGGGGGGGGGGGGGGGGGGGGGGGGGGGGGGGG 1260
Bhutan-09005         GRGRRRRARRGRGGRRGGRRGGRRRRRRRRRRARRRRRRRRRRGRRRRRRRRRRRRGRRR 1260
Indonesia-88035      RRRAAGRRGARGGGGGRRGARGGGGGGGGRAARAGGGGGGGGGRRGGGGGRRAAGGGGGG 1260
Indonesia-88045      RARARGGGGARGGGGGRAGARRGGGGGGGRARRAGGGGGGGGGRRGGGGGRRAAGGRGGG 1260
Indonesia-88065      GAGARRRARAGRRRRRGGRRGGRRRRRRRGAAAAGRRRRRRRRGGRRRRRGGRARRGRRR 1260
```

```
Bhutan-09015        GGGGGGGGGGGGGGGGGGGGAAAAAAAAAAAAAAAAAAAAAAAAAAAAAAAAAAAAAAAA 1320
Bhutan-09024        GGGGGGGGGGGGGGGGGGGGAAAAAAAAAAAAAAAAAAAAAAAAAAAAAAAAAAAAAAAA 1320
Bhutan-09027        GGGGGGGGGGGGGGGGGGGGAAAAAAAAAAAAAAAAAAAAAAAAAAAAAAAAAAAAAAAA 1320
Bhutan-09030        GGGGGGGGGGGGGGGGGGGGAAAAAAAAAAAAAAAAAAAAAAAAAAAAAAAAAAAAAAAA 1320
Bhutan-09005        RRRRRRRRRRRRRRRRRRRRWAWWAWWWWWAAAWWWWWWAWAAWWWAWWAWWWTWWTWWA 1320
Indonesia-88035     GGGGGGGGGGGGGGGGGGGGTAWTWAAAAAWWWAWATTWAAWAAWWAAAAAAAWATWWAT 1320
Indonesia-88045     GGGGGGGGGGGGGGGGGGGGTAWTWAAAAAWWWAWATTWWAWAAWTWAWAAAAWATWWWT 1320
Indonesia-88065     RRRRRRRRRRRRRRRRRRRRTWTWAWWWWWAAAWAWTTAAWAWATTWWWWWWWTWWTTWW 1320


Bhutan-09015        AAAAAAAAAAAAAAAAAAAAAAAAAAAAAAAAAAAAAAAAAAAAAAAAAAAAAAAAAAAA 1380
Bhutan-09024        AAAAAAAAAAAAAAAAAAAAAAAAAAAAAAAAAAAAAAAAAAAAAAAAAAAAAAAAAAAA 1380
Bhutan-09027        AAAAAAAAAAAAAAAAAAAAAAAAAAAAAAAAAAAAAAAAAAAAAAAAAAAAAAAAAAAA 1380
Bhutan-09030        AAAAAAAAAAAAAAAAAAAAAAAAAAAAAAAAAAAAAAAAAAAAAAAAAAAAAAAAAAAA 1380
Bhutan-09005        AWWWWWWWWWWWWTAATWWWWWWWAWWAAWAAAWAATAATWWAWAWWWWWWWWWATAWTA 1380
Indonesia-88035     WWTTATTAAATATTWWWATAAWAAWATWTTWAWWAAWWWWAAWTWTWAAAAAAAWAWWWA 1380
Indonesia-88045     WATTAWTAAATATWWWWATWATAAWATWWTWAAAAWWAWWAAWTWTAAAAAAAAWAWWWW 1380
Indonesia-88065     WWWWWWWWWWWWWTAATWWWWWWWAWWAAWWWAAWWTAATWWAWAWAAWWWWWWAWATTT 1380


Bhutan-09015        AAAAAAAAAAAAAAAAAAAAAAAAAAAAAAAAAAAAAAAAAAAAAAAAAAAAAAAAAAAA 1440
Bhutan-09024        AAAAAAAAAAAAAAAAAAAAAAAAAAAAAAAAAAAAAAAAAAAAAAAAAAAAAAAAAAAA 1440
Bhutan-09027        AAAAAAAAAAAAAAAAAAAAAAAAAAAAAAAAAAAAAAAAAAAAAAAAAAAAAAAAAAAA 1440
Bhutan-09030        AAAAAAAAAAAAAAAAAAAAAAAAAAAAAAAAAAAAAAAAAAAAAAAAAAAAAAAAAAAA 1440
Bhutan-09005        AWAAWWAWAWWAWWWAAWAWWWTWAAWWTAWWWWWWWWAWWWWWWWWWWWWAWWAWWAWW 1440
Indonesia-88035     WAWWAAAWAAWWAAAAWWWATAWAWWAAWWAAATAAAAAATAAAATAAAAAATAWAAWAA 1440
Indonesia-88045     AAWWAAWAWAWWAAWWWAWATAWWWWAATWAAATAAAAWATAAAAWAAAAAWTAWAAWAA 1440
Indonesia-88065     WWAAWAWWWWAAWWWAAAAWWWTWWAWAWAAWWWWWWWWWWWWWWWWWWWAWWAWWAAW 1440


Bhutan-09015        AAAAAAAAAAAAAAAAAAAAAAAAAAAAAAAAAAAAAAAAAAAAAAAAATTTTTTTTTTT 1500
Bhutan-09024        AAAAAAAAAAAAAAAAAAAAAAAAAAAAAAAAAAAAAAAAAAAAAAAAATTTTTTTTTTT 1500
Bhutan-09027        AAAAAAAAAAAAAAAAAAAAAAAAAAAAAAAAAAAAAAAAAAAAAAAAATTTTTTTTTTT 1500
Bhutan-09030        AAAAAAAAAAAAAAAAAAAAAAAAAAAAAAAAAAAAAAAAAAAAAAAAATTTTTTTTTTT 1500
Bhutan-09005        WWWWWWAAWWWWWWWWAAWWWWWWWWWWWWWWWWWAAWWWWWWWWWWWWWWTWTWWWWWWWWT 1500
Indonesia-88035     AAAAAAWWAAAAAAAAAWAAAAAAAAAATAAATAAWWAAAAAAAWAAAAAAWTWWTAATTTTTW 1500
Indonesia-88045     AAAAAAWWAAAAAAAAWWAAWAAAAAAATAAATAAWWAAAAAAAWAAAAAAAATWTAATTTTTW 1500
Indonesia-88065     WWWWWWAAWWWWWWWWWAWWWWWWWWWWAWWWWTWWAAWWWWAWAWWWWWWWWWTWWWWWWWWT 1500


Bhutan-09015        TTTTTTTTTTTTTTTTTTTTTTTTTTTTTTTTTTTTTTTTTTTTTTTTTTTTTTTTTTTT 1560
Bhutan-09024        TTTTTTTTTTTTTTTTTTTTTTTTTTTTTTTTTTTTTTTTTTTTTTTTTTTTTTTTTTTT 1560
Bhutan-09027        TTTTTTTTTTTTTTTTTTTTTTTTTTTTTTTTTTTTTTTTTTTTTTTTTTTTTTTTTTTT 1560
Bhutan-09030        TTTTTTTTTTTTTTTTTTTTTTTTTTTTTTTTTTTTTTTTTTTTTTTTTTTTTTTTTTTT 1560
Bhutan-09005        WWTWTTWWWTTWWWTWTTWWTTATWTWAWWWWWWWWWWWAWAWWWWTWWWTWWWWTWAWT 1560
Indonesia-88035     TAWWWWTAWTWTWAWAWWTTWWWWTWTWATAATTWTTTTWTWTTTWTWTATTTAAWAWWW 1560
Indonesia-88045     TWWTWWTATTWTTWWAWWTTWWWWTWTWATAATWATTTTWTWTTTAWTTAWTTWAWWWWW 1560
Indonesia-88065     TWTWTTTWWWTWTWWWTTWWTTATWTWAWWWWWWWWWWWWWWWWWAWWWTWWWWTWWTT 1560


Bhutan-09015        TTTTTTTTTTTTTTTTTTTTTTTTTTTTTTTTTTTTTTTTTTTTTTTTTTTTTTTTTTTT 1620
Bhutan-09024        TTTTTTTTTTTTTTTTTTTTTTTTTTTTTTTTTTTTTTTTTTTTTTTTTTTTTTTTTTTT 1620
Bhutan-09027        TTTTTTTTTTTTTTTTTTTTTTTTTTTTTTTTTTTTTTTTTTTTTTTTTTTTTTTTTTTT 1620
Bhutan-09030        TTTTTTTTTTTTTTTTTTTTTTTTTTTTTTTTTTTTTTTTTTTTTTTTTTTTTTTTTTTT 1620
```

```
Bhutan-09005        TWWAWTTWWWWTTWTWWWAWWTATTTTAWTTWWWWWWWTAWTTTTTWWWAWTWTAWTWTW 1620
Indonesia-88035     WTWATWTTTTTWWTWTWATTTWWWWWWWTTWWWWTTTWTWTWTWWWWTTWTTTWWTWTWT 1620
Indonesia-88045     WTWATWTTTTTWWTWTTATTTWWWWWWWTWWTTWTTTTTWTWWAWTTTTWTWTWWTWTWT 1620
Indonesia-88065     TWTWWTWWWWWTTWTWWWWWWTATTTTWWTTWWTWWWWWAWWTTTTWWWWTTWTAWTWTW 1620


Bhutan-09015        TTTTTTTTTTTTTTTTTTTTTTTTTTTTTTTTTTTTTTTTTTTTTTTTTTTTTTTTTTTT 1680
Bhutan-09024        TTTTTTTTTTTTTTTTTTTTTTTTTTTTTTTTTTTTTTTTTTTTTTTTTTTTTTTTTTTT 1680
Bhutan-09027        TTTTTTTTTTTTTTTTTTTTTTTTTTTTTTTTTTTTTTTTTTTTTTTTTTTTTTTTTTTT 1680
Bhutan-09030        TTTTTTTTTTTTTTTTTTTTTTTTTTTTTTTTTTTTTTTTTTTTTTTTTTTTTTTTTTTT 1680
Bhutan-09005        TWTWWWWWWWWWWWWTTTWWWWWWWWTTWWWWWWTWWWAATWWTWWWWWWWWWWTWWWWT 1680
Indonesia-88035     TTWTTTTTTTTTTTTWWTTTTTTTTTWWTTTTTTWATTWWTTTWATTTTTTWWAWTTTTW 1680
Indonesia-88045     WTWTTTTTTTTTTTTWWWTTTTTTTTWWTTTTTTWATTWWTTTWATTTTTTWWAWTTTTW 1680
Indonesia-88065     TWTWWWWWWWWWWWWTTTWWWWWWWWTTWWWWWWWWWWWAAWWWTWWWWWWWTTATWWWWT 1680


Bhutan-09015        TTTTTTTTTTTTTTCCCCCCCCCCCCCCCCCCCCCCCCCCCCCCCCCCCCCCCCCCCCCC 1740
Bhutan-09024        TTTTTTTTTTTTTTCCCCCCCCCCCCCCCCCCCCCCCCCCCCCCCCCCCCCCCCCCCCCC 1740
Bhutan-09027        TTTTTTTTTTTTTTCCCCCCCCCCCCCCCCCCCCCCCCCCCCCCCCCCCCCCCCCCCCCC 1740
Bhutan-09030        TTTTTTTTTTTTTTCCCCCCCCCCCCCCCCCCCCCCCCCCCCCCCCCCCCCCCCCCCCCC 1740
Bhutan-09005        WTWWWWWWTWWWWWSSCSSSSCSCSSSCCGCCGSSSCCSCSSSSCSSSCCSSSCSSGCSC 1740
Indonesia-88035     TTTWTTTTTTTTTTSCSCCSCSCSSCCSSSSSSGGGSSCCGCCCSGCCSSCCCSCCSSSS 1740
Indonesia-88045     TWTTTTTWWTTTTTCSSCCGSSCSSCCSCSSSSSGGSSCCGCCSSGCCSSCCCSSCSSCS 1740
Indonesia-88065     WTWTWWWTTWWWWWSSCSSSSCSCCCCCCGCSGSGSCCSSSSSSSSSSCCSSSSSSGCCC 1740


Bhutan-09015        CCCCCCCCCCCCCCCCCCCCCCCCCCCCCCCCCCCCCCCCCCCCCCCCCCGGGGGGGGGG 1800
Bhutan-09024        CCCCCCCCCCCCCCCCCCCCCCCCCCCCCCCCCCCCCCCCCCCCCCCCCCGGGGGGGGGG 1800
Bhutan-09027        CCCCCCCCCCCCCCCCCCCCCCCCCCCCCCCCCCCCCCCCCCCCCCCCCCGGGGGGGGGG 1800
Bhutan-09030        CCCCCCCCCCCCCCCCCCCCCCCCCCCCCCCCCCCCCCCCCCCCCCCCCCGGGGGGGGGG 1800
Bhutan-09005        CSCSSSSSSSSCSSSSSSSSSSSSCSSSSSSSCSSSSSSCSSCSSSSGSSSSSSSSGSGGCG 1800
Indonesia-88035     SCSCCCCCCCCSCCGCCCCCCCCSGCCCCCCSCCCCSCSCCSGCCCSCCCGGGGSGSSSS 1800
Indonesia-88045     SCSCCCCCCCCSCCSCCCCCCCCSGCCCCCCSCCCCCCSCCSSCCCSCCCGGGGGGSSSS 1800
Indonesia-88065     CSCSSSSSSSCCSSSSSSSSSSSSCSSSSSSSGSSSSSSCSSCGSSSGSSSSSSSSGSGSCG 1800


Bhutan-09015        GGGGGGGGGGGGGGGGGGGGGGGGGGGGGGGGGGGGGGGGGGGGGGGGGGGGGGGGGGGG 1860
Bhutan-09024        GGGGGGGGGGGGGGGGGGGGGGGGGGGGGGGGGGGGGGGGGGGGGGGGGGGGGGGGGGGG 1860
Bhutan-09027        GGGGGGGGGGGGGGGGGGGGGGGGGGGGGGGGGGGGGGGGGGGGGGGGGGGGGGGGGGGG 1860
Bhutan-09030        GGGGGGGGGGGGGGGGGGGGGGGGGGGGGGGGGGGGGGGGGGGGGGGGGGGGGGGGGGGG 1860
Bhutan-09005        GSSGSCCCGGSSGSSSSSGSGGSGSSCSGGSSGSGSCGGSGGGSSSSSSSCSSSSSSSSS 1860
Indonesia-88035     SCGSSSSSSSCGSGGGGSSCSSSGCCGSSSGSSSSCSSSSSSGCGGGGGGSGGCGGGGGG 1860
Indonesia-88045     SCGSGSSSSSCGSGGGSGSCSSSSSCSGSSGGSCSCSSSGSSGSGGGGGGSGGCGGGGGG 1860
Indonesia-88065     GSSGSCCCGGSSGSSSSSGSGGGGSSCSGGSGGSGSCGGSGGSCGSSSSSCSSSSSSSSS 1860


Bhutan-09015        GGGGGGGGGGGGGGGGGGGGGGGGCCCCCCCCCCCCCCCCCCCCCCCCCCCCCCCCCCCC 1920
Bhutan-09024        GGGGGGGGGGGGGGGGGGGGGGGGCCCCCCCCCCCCCCCCCCCCCCCCCCCCCCCCCCCC 1920
Bhutan-09027        GGGGGGGGGGGGGGGGGGGGGGGGCCCCCCCCCCCCCCCCCCCCCCCCCCCCCCCCCCCC 1920
Bhutan-09030        GGGGGGGGGGGGGGGGGGGGGGGGCCCCCCCCCCCCCCCCCCCCCCCCCCCCCCCCCCCC 1920
Bhutan-09005        SSSSSSGSSGGCSSSSGSSSSSSSTTCYYCYCCCTYYYCYCYYYYYYYYYCYYCYYYYTY 1920
Indonesia-88035     GGGGGGSGGSGSCSGGSCGSGGGGYYYTCCTCYYYTTYYTYCCCTTCTCCYCCYTTCYYT 1920
Indonesia-88045     GGGGGGSGGGSSCGGGGCGCGGGGYYYTCYYYYYYTTTYTYYCCTTCTCCYCCYTTCYYT 1920
Indonesia-88065     SSSSSSGSSGGCCSSSGSSSSSSSSYYCYCCYYYYTYTYCTCCYYYYYYYYCYYCYYYCYT 1920
```

```
Bhutan-09015         CCCCCCCCCCCCCCCCCCCCCCCCCCCCCCCCCCCCCCCCCCCCCCCCCCCCCCCCCCCC 1980
Bhutan-09024         CCCCCCCCCCCCCCCCCCCCCCCCCCCCCCCCCCCCCCCCCCCCCCCCCCCCCCCCCCCC 1980
Bhutan-09027         CCCCCCCCCCCCCCCCCCCCCCCCCCCCCCCCCCCCCCCCCCCCCCCCCCCCCCCCCCCC 1980
Bhutan-09030         CCCCCCCCCCCCCCCCCCCCCCCCCCCCCCCCCCCCCCCCCCCCCCCCCCCCCCCCCCCC 1980
Bhutan-09005         YYCYYYYCYYYYYCYYYCCCYYYCYYYYCYYCCCYTYYCYYTYYYYCYYCTCYYYYCYCY 1980
Indonesia-88035      TCCCCTTYCYCTTYTCCYYYTYYYCCYCYYCYYCCYCCYCYYCTCTYYYYYYTYYTYCTC 1980
Indonesia-88045      YYCCCTYYCTCTTYTCCYYYTCCYCCTCCTCYYYCYCCYCCYCTCYYTTYYYYCCTYCTC 1980
Indonesia-88065      YYYYYYTYYYYTTYCYYCCCTYYCYYYYCYYCCYCTYYCYYTYYYYCYYCTYYYYYCYYY 1980

Bhutan-09015         CCCCCCCCCCCCCCCCCCCCCCCCCCCCCCCCCCCCCCCCCCCCCCCCCCCCCCCCCCCC 2040
Bhutan-09024         CCCCCCCCCCCCCCCCCCCCCCCCCCCCCCCCCCCCCCCCCCCCCCCCCCCCCCCCCCCC 2040
Bhutan-09027         CCCCCCCCCCCCCCCCCCCCCCCCCCCCCCCCCCCCCCCCCCCCCCCCCCCCCCCCCCCC 2040
Bhutan-09030         CCCCCCCCCCCCCCCCCCCCCCCCCCCCCCCCCCCCCCCCCCCCCCCCCCCCCCCCCCCC 2040
Bhutan-09005         YYTYCYYYTTYYTYYYYYYYCCYYYYYTYYYCYYCYYYYTYYYYYYYCCYYCTYYYYYTC 2040
Indonesia-88035      TCYCYTCTYYYCYCCCCYYCYYTCCCCTCCYYTCYCCTCYTYTCCCTCCCCCYTTTTCTY 2040
Indonesia-88045      TCYCYTCTYYTCYCCYCCYCYYYCCCCYCCTYTCYCCTCYTCTCCCYYCCYYYYTTYCYY 2040
Indonesia-88065      YYTYCYYYTTYYTYYYYYCYYYYYYYYTYYTCYYCYYYYTYYYYYYYCYYYCYYYYYYTY 2040

Bhutan-09015         CCCCCCCCCCCCCCCCCCCCCCCCCCCCCCCCCCCCCCCCCCCCCCCCCCCCCCCCCCCC 2100
Bhutan-09024         CCCCCCCCCCCCCCCCCCCCCCCCCCCCCCCCCCCCCCCCCCCCCCCCCCCCCCCCCCCC 2100
Bhutan-09027         CCCCCCCCCCCCCCCCCCCCCCCCCCCCCCCCCCCCCCCCCCCCCCCCCCCCCCCCCCCC 2100
Bhutan-09030         CCCCCCCCCCCCCCCCCCCCCCCCCCCCCCCCCCCCCCCCCCCCCCCCCCCCCCCCCCCC 2100
Bhutan-09005         CCYYYYYCYYCCYYYCYYYYYYYYYYYYYYYYYYYCCCYYTYCCTYTYCCCYCCCYCCTCC 2100
Indonesia-88035      CCCCYCCYCCYCCTYYCCCCCCYCTCCCCCCCCCYYYTYYYYYYYYCYYYCYYYTYYYYY 2100
Indonesia-88045      YYCCYCCYCCYYCTTYCYCCCCCCTCCCCCCCCCYYYTYYYYYYYTYCYYYCYYYTCYYYY 2100
Indonesia-88065      CCYYCYYCYYCCYYYYYCYYYYYYYYYYYYYYYYYCYCYTTTCCYYTYCCCYCCCTCCTYC 2100

Bhutan-09015         CCCCCCCCCCCCCCCCCCCCCCCCCCCCCCCCCCCCCCCCCCCCCCCCCCCCCCCCCCCC 2160
Bhutan-09024         CCCCCCCCCCCCCCCCCCCCCCCCCCCCCCCCCCCCCCCCCCCCCCCCCCCCCCCCCCCC 2160
Bhutan-09027         CCCCCCCCCCCCCCCCCCCCCCCCCCCCCCCCCCCCCCCCCCCCCCCCCCCCCCCCCCCC 2160
Bhutan-09030         CCCCCCCCCCCCCCCCCCCCCCCCCCCCCCCCCCCCCCCCCCCCCCCCCCCCCCCCCCCC 2160
Bhutan-09005         CYYYCYYCTYYTCYTYYCYYYCCCCYCYCCCCYCCYCCYYYYYYYTTTCCCYYYCYYYCY 2160
Indonesia-88035      YCCYCYCYYYCYYCYCCYCCCYYYYCYYYYYYCYYCYYYCCCCCCYYYYYYCCYYCCCYC 2160
Indonesia-88045      YCCCYTYCYTCYYCYCYYCCYYYYYCYCYYYYCYYCYYCCCCCCCYTYYYYCCYYCCCYC 2160
Indonesia-88065      CYYYCYYCTYCTCYTYCYYYYCCCCCCYCCCCYYCYCCCYYYYYYTTYCCCYYCCYYCYY 2160

Bhutan-09015         CCCCCCCCCCCCCCCCCCCCCCCCCCCCCCCCCCCCCCCCCCCCCCCCCCCCCCCCCCCC 2220
Bhutan-09024         CCCCCCCCCCCCCCCCCCCCCCCCCCCCCCCCCCCCCCCCCCCCCCCCCCCCCCCCCCCC 2220
Bhutan-09027         CCCCCCCCCCCCCCCCCCCCCCCCCCCCCCCCCCCCCCCCCCCCCCCCCCCCCCCCCCCC 2220
Bhutan-09030         CCCCCCCCCCCCCCCCCCCCCCCCCCCCCCCCCCCCCCCCCCCCCCCCCCCCCCCCCCCC 2220
Bhutan-09005         YYYYTYYYYYYYYYYYYYYYYYYTYYYCYYYYYYYYYYYYCCYCYYYYYYYYYYTYTYYYYYT 2220
Indonesia-88035      CTTCTCCCTCCCCTCCTCTCCCYYTTYCTCCCCCCCCCYYCYCCCCCCCCCYCYCCCCCY 2220
Indonesia-88045      CTYCTCCCTCCCCTCCTCTCCCYCYTYCTCCCCCCCCCYYCYCCCCCCCCCYCYCCCCCY 2220
Indonesia-88065      YYTCYYYYYYYYYCYYYYYYYCYYYYYYYYYYYYYYYYCCYCYYYYYYYYYYTYTYYYYYY 2220

Bhutan-09015         CCCCCCCCCCCCCCCCCCCCCCCCCCCCCCCCCCCCCCCCCCCCCCCCCCCCCCCCCCCC 2280
Bhutan-09024         CCCCCCCCCCCCCCCCCCCCCCCCCCCCCCCCCCCCCCCCCCCCCCCCCCCCCCCCCCCC 2280
```

```
Bhutan-09027        CCCCCCCCCCCCCCCCCCCCCCCCCCCCCCCCCCCCCCCCCCCCCCCCCCCCCCCCCCCC 2280
Bhutan-09030        CCCCCCCCCCCCCCCCCCCCCCCCCCCCCCCCCCCCCCCCCCCCCCCCCCCCCCCCCCCC 2280
Bhutan-09005        YCYYCYYYYYCYYYYYYYYCYYYYYYYCYCYYYYYYYYYYYYYYYYYYCYTYCCTCCCYC 2280
Indonesia-88035     CYCYCCCCCCYCCCCCCCYYCCCCCCCCCCCCCCTCYCYCCCCCTCCTYCCCYYYYYYTY 2280
Indonesia-88045     CYCCCCCCCCYCCCCCCCCYCCCCCCCCCCCCCCYCTCCCCCCCTCCYYCCCCYCYYYYY 2280
Indonesia-88065     YCCYYYYYYYCYYYYYYYYTYYYYYYYYYYYYYYYYYYYYYYYYYYYYCYYYCCTCCYYC 2280


Bhutan-09015        CCCCCCCCCCCCCCCCCCCCCCCCCCCCCCCCCCCCCCCCCTTTTTTTTTTTTTTTTTTT 2340
Bhutan-09024        CCCCCCCCCCCCCCCCCCCCCCCCCCCCCCCCCCCCCCCCCTTTTTTTTTTTTTTTTTTT 2340
Bhutan-09027        CCCCCCCCCCCCCCCCCCCCCCCCCCCCCCCCCCCCCCCCCTTTTTTTTTTTTTTTTTTT 2340
Bhutan-09030        CCCCCCCCCCCCCCCCCCCCCCCCCCCCCCCCCCCCCCCCCTTTTTTTTTTTTTTTTTTT 2340
Bhutan-09005        YYYCYYCYYYYYYCYYYCCYCYYYTYYYYYCYYYYYYYYYYTYYTYYYYYTTYTYYCYYC 2340
Indonesia-88035     CCCYCCCCCCCCYYYCCCCCYCCCYCCCCCYCCCCCCCCCCYTYYYYYTTYYTYTCYTTY 2340
Indonesia-88045     CCCYCCYCCCCCTYCCCYYCYCCCYCCCCCYCCCCCCCCCCTYCYYTTTTYCTYTCYTTY 2340
Indonesia-88065     TYYCYYYYYYYYTCCYYYCCCYYYTYYYYYCYYYYYYYYYYYTYYCYYTYTYYTYYCYYC 2340


Bhutan-09015        TTTTTTTTTTTTTTTTTTTTTTTTTTTTTTTTTTTTTTTTTTTTTTTTTTTTTTTTTTTT 2400
Bhutan-09024        TTTTTTTTTTTTTTTTTTTTTTTTTTTTTTTTTTTTTTTTTTTTTTTTTTTTTTTTTTTT 2400
Bhutan-09027        TTTTTTTTTTTTTTTTTTTTTTTTTTTTTTTTTTTTTTTTTTTTTTTTTTTTTTTTTTTT 2400
Bhutan-09030        TTTTTTTTTTTTTTTTTTTTTTTTTTTTTTTTTTTTTTTTTTTTTTTTTTTTTTTTTTTT 2400
Bhutan-09005        YYTTTYYYYCYYCYYYYYYYYYCCYYYYYTYTYYTYYYYYYYYTTYYYYYTTCTYYTTYYYC 2400
Indonesia-88035     CTYYTTTTTYTTTTTTTYTYCTYYYYYTTYCYCYYYTYTYYYYYTYTTYYYYCTYYTCYY 2400
Indonesia-88045     CTYYTTTTTYTTTTTTTYTCYTYYCCTTYYCYYTYYTTTTYTYYTCTTYYYYCYYYTCCY 2400
Indonesia-88065     YYTTYYYYYCYYYYYYYYTTCYTYCCCYYYTYYYYYCYYYYTYTTYYTYTTCTYYTTYYYC 2400


Bhutan-09015        TTTTTTTTTTTTTTTTTTTTTTTTTTTTTTTTTTTTTTTTTTTTTTTTTTTTTTTTTTTT 2460
Bhutan-09024        TTTTTTTTTTTTTTTTTTTTTTTTTTTTTTTTTTTTTTTTTTTTTTTTTTTTTTTTTTTT 2460
Bhutan-09027        TTTTTTTTTTTTTTTTTTTTTTTTTTTTTTTTTTTTTTTTTTTTTTTTTTTTTTTTTTTT 2460
Bhutan-09030        TTTTTTTTTTTTTTTTTTTTTTTTTTTTTTTTTTTTTTTTTTTTTTTTTTTTTTTTTTTT 2460
Bhutan-09005        YYCCCYYYTYTTTTTYTYYTYTCYYYYYTYTYYYYYTTYYTYYYYYYYYTYYYYCYYYYTY 2460
Indonesia-88035     TTYYYTYYTYYYYYYTYCTYTYYTYTTYYCYCTCTTYYTTYTTCCTTYCYTTYTYTTTTYT 2460
Indonesia-88045     TTYYYTTYTYYYYYYTYCTYTYYTTYTTYCYCTCTTYYTTYTTCCTTCCYTTYTYTTTTYT 2460
Indonesia-88065     TYCCCYTTYTTTTYYTYYTYTYYYYYTTYYYYYYYTTYYYYYYYYYCYTYYCYCYYYYTY 2460


Bhutan-09015        TTTTTTTTTTTTTTTTTTTTTTTTTTTTTTTTTTTTTTTTTTTTTTTTTTTTTTTTTTTT 2520
Bhutan-09024        TTTTTTTTTTTTTTTTTTTTTTTTTTTTTTTTTTTTTTTTTTTTTTTTTTTTTTTTTTTT 2520
Bhutan-09027        TTTTTTTTTTTTTTTTTTTTTTTTTTTTTTTTTTTTTTTTTTTTTTTTTTTTTTTTTTTT 2520
Bhutan-09030        TTTTTTTTTTTTTTTTTTTTTTTTTTTTTTTTTTTTTTTTTTTTTTTTTTTTTTTTTTTT 2520
Bhutan-09005        YTYYYYYCYYYYYCYYYYYTCYTCTYCYYYYCYTYCYYYYYYTYYTYCYYYYYYYTYTTYY 2520
Indonesia-88035     YYTTTCCYYTTYTCYCCYCYCCYYYTYTTTTYTYTYCTTCTTYTTYYYTTTTYCTTYYYC 2520
Indonesia-88045     CYTTTCCYTTTYYYTCCTCYYYYYYTYTTTTYTYTYCTTCTTYTTYTYTTTTCCTTYYYC 2520
Indonesia-88065     YYYYYYYCYYYTYCTYCYYTYYTCTYCYYYYCTTYCYYYYYYTYYTYCYYYYYYYYYTTCC 2520


Bhutan-09015        TTTTTTTTTTTTTTTTTTTTTTTTTTTTTTTTTTTTTTTTTTTTTTTTTTTTTTTTTTTT 2580
Bhutan-09024        TTTTTTTTTTTTTTTTTTTTTTTTTTTTTTTTTTTTTTTTTTTTTTTTTTTTTTTTTTTT 2580
Bhutan-09027        TTTTTTTTTTTTTTTTTTTTTTTTTTTTTTTTTTTTTTTTTTTTTTTTTTTTTTTTTTTT 2580
Bhutan-09030        TTTTTTTTTTTTTTTTTTTTTTTTTTTTTTTTTTTTTTTTTTTTTTTTTTTTTTTTTTTT 2580
Bhutan-09005        YTTYTTTYTTTYYTYTTYYTYYYTYTTTTTYYYTYTTYCYTTYTTTTCTYTYYYTTYYYT 2580
Indonesia-88035     TTYTYYYCYYYTCYYYYTCYYYTTCYYYYYYTTTYYYTYTYYTYYYYYYTYTTTYYCTTY 2580
```

```
Indonesia-88045      TYYTYYYCYYYTYYTYYTCYYCTYCYYYYYCTTYTYYTYTYYTYYYYYYTYTTTYYCTTY 2580
Indonesia-88065      YTTYYTTYTTTYYTYTTTYTTYYTYTTTTYCYYTYTTYYYTYYTTTTCYTTYYYTTYYYT 2580


Bhutan-09015         TTTTTTTTTTTTTTTTTTTTTTTTTTTTTTTTTTTTTTTTTTTTTTTTTTTTTTTTTTTT 2640
Bhutan-09024         TTTTTTTTTTTTTTTTTTTTTTTTTTTTTTTTTTTTTTTTTTTTTTTTTTTTTTTTTTTT 2640
Bhutan-09027         TTTTTTTTTTTTTTTTTTTTTTTTTTTTTTTTTTTTTTTTTTTTTTTTTTTTTTTTTTTT 2640
Bhutan-09030         TTTTTTTTTTTTTTTTTTTTTTTTTTTTTTTTTTTTTTTTTTTTTTTTTTTTTTTTTTTT 2640
Bhutan-09005         YTYYYYYYCCYYYTTYCYTYYTYTTYTYYTYYYYTYYYYYYYYYYYYYYYYCYYYYYYT 2640
Indonesia-88035      CYCYTTTTYYTCTYYTYTYTTYYYYYYTTYCTTTYTTTTTTTTTTTCCTTCTTTTTTTYY 2640
Indonesia-88045      CYCTTTTTYYTCTYYTYTYTTYCYYYYTTYCTTTYTTTTTTTTTTTCCTTCTTTTTTTTY 2640
Indonesia-88065      YTYYYYYYYYYYYTTYCYTYYTYTTTTYYTYYYYTYYYYYYYYYYCYYYYYYYYYYYYTT 2640


Bhutan-09015         TTTTTTTTTTTTTTTTTTTTTTTTTTTTTTTTTTTTTTTTTTTTTTTTTTTTTTTTTTTT 2700
Bhutan-09024         TTTTTTTTTTTTTTTTTTTTTTTTTTTTTTTTTTTTTTTTTTTTTTTTTTTTTTTTTTTT 2700
Bhutan-09027         TTTTTTTTTTTTTTTTTTTTTTTTTTTTTTTTTTTTTTTTTTTTTTTTTTTTTTTTTTTT 2700
Bhutan-09030         TTTTTTTTTTTTTTTTTTTTTTTTTTTTTTTTTTTTTTTTTTTTTTTTTTTTTTTTTTTT 2700
Bhutan-09005         TYTTYYYYYYYYYYYYYYYYYYYYYYYYYYYYTYYYYYYYYTYYYYTYTYYYYYYYYYCYY 2700
Indonesia-88035      YTYYTTTCTTCTTCTTTTTTTTTTTTTTTTTTCTTTTTTYTTTTYTYTTTYTTTTTTYTT 2700
Indonesia-88045      YYYYTTTCTTCTTCTTTTTTTTTTTTTTTTTTCTTTTTTYTTTTYTYTTTCTTTTTTYTT 2700
Indonesia-88065      TYTTYYYYYYYYYYYYYYYYYCYYYYYYYYYYYYYYYYYYTYYYYTTYYYYCYYYYYYCYT 2700


Bhutan-09015         TTTTTTTTTTTTTTTTTTTTTTTTTTTTTTTTTTTTTTTTTTTTTTTTTTTTTTTTGGGG 2760
Bhutan-09024         TTTTTTTTTTTTTTTTTTTTTTTTTTTTTTTTTTTTTTTTTTTTTTTTTTTTTTTTGGGG 2760
Bhutan-09027         TTTTTTTTTTTTTTTTTTTTTTTTTTTTTTTTTTTTTTTTTTTTTTTTTTTTTTTTGGGG 2760
Bhutan-09030         TTTTTTTTTTTTTTTTTTTTTTTTTTTTTTTTTTTTTTTTTTTTTTTTTTTTTTTTGGGG 2760
Bhutan-09005         YTYYYCYYYYYYYYTYYYYCCCYYTYYYYYYYYYYTYCYTYTTTYYYYYYYYYYYYGKKK 2760
Indonesia-88035      TYTTTTTTYTYYYYTTTTTYYYTTYTTTTTTTTTTYTCTYTYYYTTYTTTTTCTTTGGKT 2760
Indonesia-88045      TYTTTTTTYTYYCCYTTTTTTTTTYTTTTTTTTTTYTYTYTYYTTTTTTTTTCTTTKKKT 2760
Indonesia-88065      YTYYYYYYCYTCCCTYYYYCCCYYTYYYTYYYYYYTYYYTYYTTTYYYTYYYYYYYGGTT 2760


Bhutan-09015         GGGGGGGGGGGGGGGGGGGGGGGGGGGGGGGGGGGGGGGGGGGGGGGGGGGGGGGGGGGG 2820
Bhutan-09024         GGGGGGGGGGGGGGGGGGGGGGGGGGGGGGGGGGGGGGGGGGGGGGGGGGGGGGGGGGGG 2820
Bhutan-09027         GGGGGGGGGGGGGGGGGGGGGGGGGGGGGGGGGGGGGGGGGGGGGGGGGGGGGGGGGGGG 2820
Bhutan-09030         GGGGGGGGGGGGGGGGGGGGGGGGGGGGGGGGGGGGGGGGGGGGGGGGGGGGGGGGGGGG 2820
Bhutan-09005         KGKGKKKKGGGKKKKTKGGGKKKKKKGGGGKTKGKGKKKKKGKKGGKGGKGGGKKKKKK 2820
Indonesia-88035      KKGKGGGGKKGKGKGKTKKGGGTTGKGKKKKGGTGGKGKKTKKGTKKGKKGKKKGGKGTG 2820
Indonesia-88045      GKGKGGGGGKKGGTGTTKKKGGTTGGGKKKGKGTGGKGKTKTKGTKKGKKGKKKGGKGTG 2820
Indonesia-88065      KGKGKKKKGGKKKKKKGGGKKKKKKGGGGKKKKKGKGKKKGKKGGKGGKGGGKKGKTK 2820


Bhutan-09015         GGGGGGGGGGGGGGGGGGGGGGGGGGGGGGGGGGGGGGGGGGGGGGGGGGGGGGGGGGGG 2880
Bhutan-09024         GGGGGGGGGGGGGGGGGGGGGGGGGGGGGGGGGGGGGGGGGGGGGGGGGGGGGGGGGGGG 2880
Bhutan-09027         GGGGGGGGGGGGGGGGGGGGGGGGGGGGGGGGGGGGGGGGGGGGGGGGGGGGGGGGGGGG 2880
Bhutan-09030         GGGGGGGGGGGGGGGGGGGGGGGGGGGGGGGGGGGGGGGGGGGGGGGGGGGGGGGGGGGG 2880
Bhutan-09005         KKKKKGKGGGKGGKGKKGKKGKKKTKGKKKGKKKKKKKKKKGKKKGKKKGKKKGKKKKKK 2880
Indonesia-88035      TKTGGKGKKKTKKGGKGKGTKGGGKTKGTGKGGGGGGTTTGKGGGKGGGKGGGKGGGGGG 2880
Indonesia-88045      TKTGGKGKKKTKKKGKGKGTKGGGKKKGTGKGGGGGGTTTGKGGGGGGGKGGGKGGGGGG 2880
Indonesia-88065      TTKKKGKGGGKGGKKTKGKTGKKKKTGKKKGKKKKKKKKKKGKKKGKKKGKKKGKKKKKK 2880
```

```
Bhutan-09015        GGGGGGGGGGGGGGGGGGGGGGGGGGGGGGGGGGGGGGGTTTTTTTTTTTTTTTTTTTTT 2940
Bhutan-09024        GGGGGGGGGGGGGGGGGGGGGGGGGGGGGGGGGGGGGGGTTTTTTTTTTTTTTTTTTTTT 2940
Bhutan-09027        GGGGGGGGGGGGGGGGGGGGGGGGGGGGGGGGGGGGGGGTTTTTTTTTTTTTTTTTTTTT 2940
Bhutan-09030        GGGGGGGGGGGGGGGGGGGGGGGGGGGGGGGGGGGGGGGTTTTTTTTTTTTTTTTTTTTT 2940
Bhutan-09005        TGGKKKKKKKKKKKKKGKKKKKGGGKKKKKKKKGTKKKKTTTTGTKKKTKKTTTKKKTGK 2940
Indonesia-88035     KKKTKGGGGGKGGGGGGGGGGTKKKGGGTKGGGGKGGGGTKKKKTTTTTKTKTKGTGKKG 2940
Indonesia-88045     KKKTGGGGGGKGGGGGGGGGGTKKKGGGKGGGGKKGGGGTKKKKKTTTKTTKTKGTGKKK 2940
Indonesia-88065     KKKTGKKKKKGGKKKKKKKKKKGGGKKKKGGKKGKKKKKKTTTGTKKKTKTTKKKKKTGK 2940


Bhutan-09015        TTTTTTTTTTTTTTTTTTTTTTTTTTTTTTTTTTTTTTTTTTTTTTTTTTTTTTTTTTTT 3000
Bhutan-09024        TTTTTTTTTTTTTTTTTTTTTTTTTTTTTTTTTTTTTTTTTTTTTTTTTTTTTTTTTTTT 3000
Bhutan-09027        TTTTTTTTTTTTTTTTTTTTTTTTTTTTTTTTTTTTTTTTTTTTTTTTTTTTTTTTTTTT 3000
Bhutan-09030        TTTTTTTTTTTTTTTTTTTTTTTTTTTTTTTTTTTTTTTTTTTTTTTTTTTTTTTTTTTT 3000
Bhutan-09005        TKKKTKKGKKTKTGKTKKKKKKTKTKKKKTKTTKTTKTTTTTTGKKKKKKKKKTKTTTKT 3000
Indonesia-88035     KGKKKTTKTKTTKKKTTTTKTTTTKTTTTKGKTTTKKKKKKTKKGTTTTTTGGKKKKKKK 3000
Indonesia-88045     KGTGKTTKTGKTKKTKKKTTTTKTKTTTTKGKKKKKGKKKKKKKGTTTTTTGKKTKKKTK 3000
Indonesia-88065     TKKKKKKGKKKKTGTKKKKKTKTKTKKTKTGTTKTKKTTTTKTKKKKKKKKKKTKTTTTT 3000


Bhutan-09015        TTTTTTTTTTTTTTTTTTTTTTTTTTTTTTTTTTTTTTTTTTTTTTTTTTTTTTTTTTTT 3060
Bhutan-09024        TTTTTTTTTTTTTTTTTTTTTTTTTTTTTTTTTTTTTTTTTTTTTTTTTTTTTTTTTTTT 3060
Bhutan-09027        TTTTTTTTTTTTTTTTTTTTTTTTTTTTTTTTTTTTTTTTTTTTTTTTTTTTTTTTTTTT 3060
Bhutan-09030        TTTTTTTTTTTTTTTTTTTTTTTTTTTTTTTTTTTTTTTTTTTTTTTTTTTTTTTTTTTT 3060
Bhutan-09005        KTTKTKKKTTTTKTTTKKTKKTKKKKTTKKKKTKTKKKKKKKKKKKKKKKTKTKKKKTKT 3060
Indonesia-88035     TKTTKTTGKKKKTKKKKGKKKKTTTTKTKGTGKTKTTTTTTTTTTTGTTTTTKKTTKKTK 3060
Indonesia-88045     TKKTTTTGKKKKTKKKGGKTTKTTTTKKKGTGKKKTTTTTTTTTTTGTTTKTKTTTKTTT 3060
Indonesia-88065     KTTKTTKKTTTTKTTTKKKKKTKKKKTTTKKKTTTKKKKKKKKKKKKKKKTKTTKKGTKK 3060


Bhutan-09015        TTTTTTTTTTTTTTTTTTTTTTTTTTT 3087
Bhutan-09024        TTTTTTTTTTTTTTTTTTTTTTTTTTT 3087
Bhutan-09027        TTTTTTTTTTTTTTTTTTTTTTTTTTT 3087
Bhutan-09030        TTTTTTTTTTTTTTTTTTTTTTTTTTT 3087
Bhutan-09005        KKKKKKKKKKKKTKKKTKTKKKKKKKK 3087
Indonesia-88035     TTTTTKTTTTKTTTTTKTTTTTGGTTT 3087
Indonesia-88045     TTTTTGTTTTKTKTTTKTKTTTGGTTT 3087
Indonesia-88065     KKKKKKKKKKTKTKKKTKTKGKKKKKK 3087
```

Supplementary Figure S6.

```
CLUSTAL 2.1 multiple sequence alignment


Bhutan-09015        MMACMCAAMMMMMMAMAMAMMMCCMMMMMMCAMMMMACAAMMMCCMMMMMMMMRRRRRRG 60
Bhutan-09024        MMMMACAMMAMMMMMMMAAACMCCMMMCMMCAMCMMMMMAMMMCCMCMMMMCRAARARAR 60
Bhutan-09027        AMMMAMAMCMCACMMMMMAAMCMMMAAMAAMMCCAAMMMMCCAMMAMMMAACARRARGRR 60
Bhutan-09030        MAMMMMMMMMMAMCMAAAMMMMCMCAMMMMMMCCMAAMMMMMMMMMCCAMAMRAAARGRR 60
Bhutan-09005        AAAAAAAAAAAAAAAAAAAACCCCCCCCCCCCCCCCCCCCCCCCCCCCCCCAAAAAAAAA 60
Indonesia-88035     AAAAAAAAAAAAAAAAAAAACCCCCCCCCCCCCCCCCCCCCCCCCCCCCCCAAAAAAAAA 60
Indonesia-88045     AAAAAAAAAAAAAAAAAAAACCCCCCCCCCCCCCCCCCCCCCCCCCCCCCCAAAAAAAAA 60
Indonesia-88065     AAAAAAAAAAAAAAAAAAAACCCCCCCCCCCCCCCCCCCCCCCCCCCCCCCAAAAAAAAA 60


Bhutan-09015        RRRRRAGAARRRGGGAGRRRARRRARRRARRRRRRARRRARAARRRRRRRRAAAAARRR 120
Bhutan-09024        RRGRRAGRRAAARGGRRAARRAARRRGRARAAARRRRARRARARRGRRARAAAARAARAR 120
Bhutan-09027        RRRRARRRRAAARGGRRRARARAAAAGGAARRRGAGRRAARRRAAGAGRARARRRRRGAA 120
Bhutan-09030        GGGGAAGRRAAARRRRRRRAARRRRARRRRRRRRRRRRARGAARGRRRRRRRAARRRAR 120
Bhutan-09005        AAAAAAAAAAAAAAAAAAAAAAAAAAAAAAAAAAAAAAAAAAAAAAAAAAAAAAAAAAAA 120
Indonesia-88035     AAAAAAAAAAAAAAAAAAAAAAAAAAAAAAAAAAAAAAAAAAAAAAAAAAAAAAAAAAAA 120
Indonesia-88045     AAAAAAAAAAAAAAAAAAAAAAAAAAAAAAAAAAAAAAAAAAAAAAAAAAAAAAAAAAAA 120
Indonesia-88065     AAAAAAAAAAAAAAAAAAAAAAAAAAAAAAAAAAAAAAAAAAAAAAAAAAAAAAAAAAAA 120


Bhutan-09015        RRRRAGRGRRARGRAARRRRRAAARRRARRGGGRRGRRGRRRGRRRAGRRRRRRRRGRRR 180
Bhutan-09024        AARARRARRRAGRRAAARRRRRAARRRRGRGGGGARGRRRRGGRRRAGRRRGARRAGGRR 180
Bhutan-09027        RRRRARARGGRRRARRRAARRARRAGARRGGRRRAGGAGGGRGRGARGAAGRGARRRGAR 180
Bhutan-09030        RRAARRARRRAGRRRRRRRGAARRRRARGRRRRRAGGRGRGGRARRRRRARRRRAAGRRG 180
Bhutan-09005        AAAAAAAAAAAAAAAAAAAAAAAAAAAGGGGGGGGGGGGGGGGGGGGGGGGGGGGGGGGG 180
Indonesia-88035     AAAAAAAAAAAAAAAAAAAAAAAAAAAGGGGGGGGGGGGGGGGGGGGGGGGGGGGGGGGG 180
Indonesia-88045     AAAAAAAAAAAAAAAAAAAAAAAAAAAGGGGGGGGGGGGGGGGGGGGGGGGGGGGGGGGG 180
Indonesia-88065     AAAAAAAAAAAAAAAAAAAAAAAAAAAGGGGGGGGGGGGGGGGGGGGGGGGGGGGGGGGG 180


Bhutan-09015        GRRRRRRRRRRRRRRRRGARRRGGRGRARGRRGRRRRGRRRARRRRGRGRGGGRGGRRRRG 240
Bhutan-09024        GRARRRRRGRRGAAGARARGRRGRRRARRRRGRRRARRGGRAGRRRRGRRGRGGGRRRRG 240
Bhutan-09027        RGAARGRRGGAGRRGRRRGRRRRRGGRARGARAGRAGRGRRRGARGARRRRRGRRGRRAR 240
Bhutan-09030        GRARGRAAGGRRGGARRARRARGARGAAGRRRARARGARRRARAAGARAGRRRRRRGGRR 240
Bhutan-09005        GGGGGGGGGGGGGGGGGGGGGGGGGGGGGGGGGGGGGGGGGGGGGGGGGGGGGGGGGGGG 240
Indonesia-88035     GGGGGGGGGGGGGGGGGGGGGGGGGGGGGGGGGGGGGGGGGGGGGGGGGGGGGGGGGGGG 240
Indonesia-88045     GGGGGGGGGGGGGGGGGGGGGGGGGGGGGGGGGGGGGGGGGGGGGGGGGGGGGGGGGGGG 240
Indonesia-88065     GGGGGGGGGGGGGGGGGGGGGGGGGGGGGGGGGGGGGGGGGGGGGGGGGGGGGGGGGGGG 240


Bhutan-09015        GGRGRRRRGRGRGRRRRRRRRRRRRGRRRRAWAWWWAWWWWAWWWWTAWWAWAAWAAAAWW 300
Bhutan-09024        RGRRRRARRGGGRRRGGRRRRRRGRGRRRWWAWWWWWTWAWWWWAWWTTWWAWAWAWWWT 300
Bhutan-09027        RGARRRAARGRGRAARGAAAARRRRGARRWTWTTWWAWAWWWATWWAWTAWAWWWWWAWW 300
Bhutan-09030        RRARGAARRRGGRRRRGRAARAARGGAAGWTWWWTAATAWWTWWWWAWTAAWWAAAWAAW 300
Bhutan-09005        GGGGGGGGGGGGGGGGGGGGGGGGGGGGGAAAAAAAAAAAAAAAAAAAAAAAAAAAAAAA 300
Indonesia-88035     GGGGGGGGGGGGGGGGGGGGGGGGGGGGGAAAAAAAAAAAAAAAAAAAAAAAAAAAAAAA 300
Indonesia-88045     GGGGGGGGGGGGGGGGGGGGGGGGGGGGGAAAAAAAAAAAAAAAAAAAAAAAAAAAAAAA 300
Indonesia-88065     GGGGGGGGGGGGGGGGGGGGGGGGGGGGGAAAAAAAAAAAAAAAAAAAAAAAAAAAAAAA 300
```

```
Bhutan-09015      WWATTWWTTTTWWWWTWWTWTTWWWTWWWWWWTSSCCCSSSSSCSSSSSCSCGSCGGGCC 360
Bhutan-09024      WTWTWWWWWWWATTWWAATWTWAWWWATWTTTTCSCCCSSSGSSSCSCSSGCGSSGGSCC 360
Bhutan-09027      AWWTWAAWWWWWWWATAWWTWWWATWWWWWWWWSCCSCSCGSCCCSSCSSCSSCSGGGSS 360
Bhutan-09030      AWWWWWWWWTWWTWWWWWTWTWWWWWTWTWWWWCCSCSCSSSSCGSCSCSGSSSCSSSCS 360
Bhutan-09005      AAATTTTTTTTTTTTTTTTTTTTTTTTTTTTTTCCCCCCCCCCCCCCCCCCCCGGGGGGG 360
Indonesia-88035   AAATTTTTTTTTTTTTTTTTTTTTTTTTTTTTTCCCCCCCCCCCCCCCCCCCCGGGGGGG 360
Indonesia-88045   AAATTTTTTTTTTTTTTTTTTTTTTTTTTTTTTCCCCCCCCCCCCCCCCCCCCGGGGGGG 360
Indonesia-88065   AAATTTTTTTTTTTTTTTTTTTTTTTTTTTTTTCCCCCCCCCCCCCCCCCCCCGGGGGGG 360
                                                   .......  .. ..... .   ...

Bhutan-09015      SSSSGSSSGGSCYCCYYYYYCYYTYYYYCYCCCYCCTCCCCYCYYYCTYYYCCYYYYYCY 420
Bhutan-09024      GSSCSSSCGGGCYCYYYTYYCYCTYTCYYYYCYCCCTCCCCTCCYTCTCYYCCYYTYTCC 420
Bhutan-09027      SCCCSCCSSSGSCYCTCYCCYYYYYYYCYYYCCYYCYYYCYTCYCTYYYCCYCCCTTYYY 420
Bhutan-09030      SCSCSCSCSSGCCCYYYYCYYTYYCYCYCCCYCYCYYCYYYYYYYTYYYYYYYYYTYYCY 420
Bhutan-09005      GGGGGGGGGGGGCCCCCCCCCCCCCCCCCCCCCCCCCCCCCCCCCCCCCCCCCCCCCCCC 420
Indonesia-88035   GGGGGGGGGGGGCCCCCCCCCCCCCCCCCCCCCCCCCCCCCCCCCCCCCCCCCCCCCCCC 420
Indonesia-88045   GGGGGGGGGGGGCCCCCCCCCCCCCCCCCCCCCCCCCCCCCCCCCCCCCCCCCCCCCCCC 420
Indonesia-88065   GGGGGGGGGGGGCCCCCCCCCCCCCCCCCCCCCCCCCCCCCCCCCCCCCCCCCCCCCCCC 420
                  .   .   ...

Bhutan-09015      YCCCCTYTYYTTCCYCYYYCYYYYTYYYCYCYYYYYCYYYYYCYYYCYYYTTTTYYYYYY 480
Bhutan-09024      CCYYYTTYCTTTCCCCTYYCYYCYYYCYYYYYYCYYCCCCYTYCTYCTTYTTTYYTCYYC 480
Bhutan-09027      YYYYYYTYCTYTYYYCYYTYCCCTTTCYYTYCTYTCYCYCTYYYYTYYYTTTYYYYYYTY 480
Bhutan-09030      YCYYYYYYCTYYCYYYYCTYYYCYTYCCYTYCYCYYYYCYYYCCYYYYTYYYYYCYYTYY 480
Bhutan-09005      CCCCCCCCCCCCCCCCCCCCCCCCCCCCCCCCCCCCCCCCCTTTTTTTTTTTTTTTTTTT 480
Indonesia-88035   CCCCCCCCCCCCCCCCCCCCCCCCCCCCCCCCCCCCCCCCCTTTTTTTTTTTTTTTTTTT 480
Indonesia-88045   CCCCCCCCCCCCCCCCCCCCCCCCCCCCCCCCCCCCCCCCCTTTTTTTTTTTTTTTTTTT 480
Indonesia-88065   CCCCCCCCCCCCCCCCCCCCCCCCCCCCCCCCCCCCCCCCCTTTTTTTTTTTTTTTTTTT 480


Bhutan-09015      TYCYYYYTYTYTYYYYYYYTYYTYYYYTTYCYTTYYTYYYYYYTCYCYKKTKGGKKGKKG 540
Bhutan-09024      TCYYCTYTYYTTYCCYCYYTYYTYTCTYTYCCTTYYTYYTTYYYYYYYKKKKKGKKKKKG 540
Bhutan-09027      YYYYCYTYCYYYCCYTCTYYYCYTTYTYYTYYYYYCYCTYTCCYYYYTGTTGKKKTKGGG 540
Bhutan-09030      YYYTCYYYCYYTYCYYYTCYTCYTTYYYYYCYTTTCYCYYYCCYYTYTGKKKKKTTKKKK 540
Bhutan-09005      TTTTTTTTTTTTTTTTTTTTTTTTTTTTTTTTTTTTTTTTTTTTTTTTGGGGGGGGGGGG 540
Indonesia-88035   TTTTTTTTTTTTTTTTTTTTTTTTTTTTTTTTTTTTTTTTTTTTTTTTGGGGGGGGGGGG 540
Indonesia-88045   TTTTTTTTTTTTTTTTTTTTTTTTTTTTTTTTTTTTTTTTTTTTTTTTGGGGGGGGGGGG 540
Indonesia-88065   TTTTTTTTTTTTTTTTTTTTTTTTTTTTTTTTTTTTTTTTTTTTTTTTGGGGGGGGGGGG 540


Bhutan-09015      KKKTGKKGKKGKKGKKKKKKKKTTKTKGKKKKKGKTKKKKKTKTKKTKKTKKTKTKK 597
Bhutan-09024      KTKKGGGKGKKKGKTKKGKGGKTTTTKKTGKTGGTTKTTKKTKTKTKGKTGKTGTKG 597
Bhutan-09027      GKKTKKKKKGGGKGKTTGGGGGTKKTGKKKTTGKKKTTKKTKTKKKGKTTGKKKKKK 597
Bhutan-09030      GKTTKKKKKKGKKGKTKKKKKGKTKKGKKGTTKGKTKKKGTKKTTKTKTKKTKKKTK 597
Bhutan-09005      GGGGGGGGGGGGGGGGGGGGGTTTTTTTTTTTTTTTTTTTTTTTTTTTTTTTTTTTT 597
Indonesia-88035   GGGGGGGGGGGGGGGGGGGGGTTTTTTTTTTTTTTTTTTTTTTTTTTTTTTTTTTTT 597
Indonesia-88045   GGGGGGGGGGGGGGGGGGGGGTTTTTTTTTTTTTTTTTTTTTTTTTTTTTTTTTTTT 597
Indonesia-88065   GGGGGGGGGGGGGGGGGGGGGTTTTTTTTTTTTTTTTTTTTTTTTTTTTTTTTTTTT 597
                                        ....  . ..  ..... ......  .. .. ..
```

Supplementary Figure S7.

```
CLUSTAL 2.1 multiple sequence alignment


Bhutan-09015         AYGACTKRYYKTTKSWKRMYATKWAGGKTKWRRWRCTYRMYWYAATYRKWAKRGAYGMRR 60
Bhutan-09024         RTRACKKAYCTTTKSWKGMYATTWAGGKTGAARWACTYMMYTTRAKYRKWMKGGAYRMRR 60
Bhutan-09027         RYGRCKKAYYKYTKSWKRMYMTKWWSSKKKWAAWRCTTAMYWYRRKYAGWMTRRMYRMAR 60
Bhutan-09030         RYGAYTKAYCTYYKSWKRMTATKWAGGTKKWARWRCWTRMCWYRRKTAGWMTRRMYGCAW 60
Bhutan-09005         RTGRYKSAKYKYTRMMSRWYAWKTAGGKKKWARARMWCAYYTTGRKYAGMASRRMAGMAG 60
Indonesia-88035      ATTACTCRGCTTTRCAGGACMRTKWSSKTGARGCAMTYRYYWYAATCRKCCSGGAWGCRG 60
Indonesia-88045      ATTACTSRTCTYTGCAGGACMRKKWSSTTGARGCRMWYRCTTTAAKCRKMCSGGAWRMRG 60
Indonesia-88065      AYAACTSRKCTYYRAMSRWTAWKTAGGKKGARRARWTCACYTTGAKYAGMMSGGAAGCRG 60
                          .    .           .  ....                .     .

Bhutan-09015         RKGCWRACRGGGATRYCCGYKWGGTTSSRTCMTTTGSYAKAYRRTRAAACKWSKYMRGTR 120
Bhutan-09024         RKSYWGACGGGGAKRMCMRCGWRRTWGGGTCAWKKRSCAKATARYRAAACKASKCCAKYA 120
Bhutan-09027         AKSCWRRYRRRRRTACCMRYKWGGTWSGGKCATTKGSCAKAYRATGRRACTWSKYMRKYA 120
Bhutan-09030         AKGCARRYRRRRRTACCMRYKWGGTWSSRKCAWTTGSCAGWYRATGAARMKWGKYMRKYA 120
Bhutan-09005         ARSYWRACRRRARTACWMRYKRRRTWSGGTYMWKTARMYGAYTATGKAACWWGCCMRKYR 120
Indonesia-88035      RGGCWGRYAGGRATRAACGCGARRKTSGGKKATTTRGMAKTTGRYRTRACWWSYCCAGTA 120
Indonesia-88045      RGSCWGACAGGRRKRAACGCGAGGKTGSRKKATTKRGMAKTTGRYRTAACWASYCCAKYA 120
Indonesia-88065      RRSCARACRGGAATRAWCGCGARRYWSSRKYMTTTRGMYGWYTRYRTARMWWGCYMRKYR 120
                       .          .            .. .   ..                 .

Bhutan-09015         AWYRAWKRASYRRWMKRRRYMCTGYRSTYTYAGWRYGTYGAARCTTSYTTTGATTKAKRS 180
Bhutan-09024         AWTRAWKRRSYRRWMKGRRYMMYSYGSTYTYAGWATGYKRAARYKWSYKKTGRKTKRTAS 180
Bhutan-09027         AWYRRTGARGTGRTMTRRACAMYGYRSTYYYAGWATGYYRRRAYTTSTTTGRKTKAKRC 180
Bhutan-09030         RWTRRTGARSYRAWMTRRRYAMYGTGSWTTYRKWATGYKRARRYTWSTTTYGATTKATAC 180
Bhutan-09005         ATTGATGARSYMRAWTRAACAMYCYRAWTYWAGAATWTGKRRRYTTMTTTTGATSGRKRC 180
Indonesia-88035      RKYKRWKRRSYARAWKGMRYMMYGTGCTYTAAGCATTYGGRAACKAAYKKYARKCSATAS 180
Indonesia-88045      AKYKRWKRAGTARWTKGMRYMMYGTRCTYTAAGCRYTYGGARAYKAMYKKTWRKSSATAS 180
Indonesia-88065      RKYGRWKRRSYAAWWKRMRYMCTCYRATYYWRKAATWYGKRRRYKAMYKKYGRKSGATAS 180
                              .     .          .                 .   ..   .   . .

Bhutan-09015         GRASRRKRYWYATTWRSKRGMMAGAWTKGGWGYKWGSGSYSKASWWTRGTWCTWYCKCRT 240
Bhutan-09024         RRAGGRTRYATCKWWRSKAGMMWGAWKGRGAGCKTGSGSTSRAKYWTAGKTMTWTCKCRT 240
Bhutan-09027         RRWGRRTRYWYMTWWASTRGMMWRRATGGGWGYKTGRRGTSGAKYTWRGTTCTWYCKCRK 240
Bhutan-09030         GRWGRRTRYWYCTTWRSTAGMMWRRWTGGRWGCKTGSRGYSKAKYWWRGTTCTWTCKCRK 240
Bhutan-09005         GSWSRSTKKWMCTTAARARKWSAGRKTKRRWYCATGCRCYYGAGTWWRGTWCWYYWRAWK 240
Indonesia-88035      AGTCAGTGTTATTWARGAAGACARAKKTGGASYTWTCGCTCMAGTTTRGTTAYTTTAATT 240
Indonesia-88045      AGTCAGTGTTAAKWRRGAAGACWRRKKTRRASYTWMCGSTCTWGTTTATKTCYTTTAATK 240
Indonesia-88065      ASWSGSKKKWMTTWARRARRWSWRRKKKRRAYCAWTCGSYYAYGTWTRRTTTWYYWRRWK 240
                           .     .             .                      .         .

Bhutan-09015         YK 242
Bhutan-09024         MK 242
Bhutan-09027         YT 242
Bhutan-09030         CT 242
Bhutan-09005         YT 242
Indonesia-88035      CK 242
Indonesia-88045      CK 242
Indonesia-88065      CT 242
                      .
```

Supplementary Figure S8.

```
CLUSTAL 2.1 multiple sequence alignment


Bhutan-09015        CGTATTGATCTAAACGCCTGGAGTATATATAATTGATCTCACAGTGATTCTCAGAGCAGT 60
Bhutan-09024        CGTATTGATCTAAACGCCTGGAGTATATATAATTGATCTCACAGTGATTCTCAGAGCAGT 60
Bhutan-09027        CGTATTGATCTAAACGCCTGGAGTATATATAATTGATCTCACAGTGATTCTCAGAGCAGT 60
Bhutan-09030        CGTATTGATCTAAACGCCTGGAGTATATATAATTGATCTCACAGTGATTCTCAGAGCAGT 60
Bhutan-09005        CGAGTCGAGCGAAGAGGCCGTAGTATGCATGCGTAATTTCATAGACCTTACAGGACATGC 60
Indonesia-88035     TATAGTCGTTTAAACACATCGGGTACATTCAATCGGACATGCTATGAACCTCAGGGCAGT 60
Indonesia-88045     TATAGTAGTTTATACACATCGGTTTCATTCAATCGGACATGCTATGAACCTCAAGGCAGT 60
Indonesia-88065     TGAGTCAAGCGTAGAGGCCGTACCATGCATGCGTAATTTCATAGACCTTACAGGACATCC 60


Bhutan-09015        ACGCACAGACAGTTTTACGTTACAGGTTTTAGACACGAAGCCGAGAAGTCCTCGGGTTTT 120
Bhutan-09024        ACGCACAGACAGTTTTACGTTACAGGTTTTAGACACGAAGCCGAGAAGTCCTCGGGTTTT 120
Bhutan-09027        ACGCACAGACAGTTTTACGTTACAGGTTTTAGACACGAAGCCGAGAAGTCCTCGGGTTTT 120
Bhutan-09030        ACGCACAGACAGTTTTACGTTACAGGTTTTAGACACGAAGCCGAGAAGTCCTCGGGTTTT 120
Bhutan-09005        ATACAAGGACAGCTAAGAGTCGCAAGTCCCAGTCAAAAAACCTAAAGCGGTATAAGTTTG 120
Indonesia-88035     ACGACCAAGATATCTTACTATAAGGTATTTCTAAGCGCTGTTGTGGAGTCCTCGGATCGT 120
Indonesia-88045     ACGACCAAGATATCTTACTATAAGGTATTTCTAAGCGCTGTAGTGGAGTCCTCGGACCGT 120
Indonesia-88065     TTACAAGGACAGCTAAGAGTCGCAAGTCCCAGTCAAGAAACATAAAGCGGTATAAGTTTG 120


Bhutan-09015        TCAAGCTCACGGACCTAGCACTTAGGTCGTGGGCGACGTATATCGGATGGCGGTTGTCTG 180
Bhutan-09024        TCAAGCTCACGGACCTAGCACTTAGGTCGTGGGCGACGTATATCGGATGGCGGTTGTCTG 180
Bhutan-09027        TCAAGCTCACGGACCTAGCACTTAGGTCGTGGGCGACGTATATCGGATGGCGGTTGTCTG 180
Bhutan-09030        TCAAGCTCACGGACCTAGCACTTAGGTCGTGGGCGACGTATATCGGATGGCGGTTGTCTG 180
Bhutan-09005        AATAGATCTTGCGCATTATGGTTGGGACATAAACGAAGTATTCCGTATGGTGTGTATCCG 180
Indonesia-88035     TCAGACCTACTGAGCCAGCACCCAGATTGAGGGTTTCACACATAAGCCAACAGTAGCATA 180
Indonesia-88045     TCAGACCTACTGAGCCAGCACCCAAATTGAGGGTTTCACACATAAGCCAACAGTAGCATA 180
Indonesia-88065     AATAGATCTTGCGCATTATGGTTGGGACATAAACGAAGTTTTCCGTATGGTGTGTATCCG 180


Bhutan-09015        CGGACATGTGGTGCTGTGCGACGATGGTGGGCCGCGGCCTAGAGGCCGTACTAGGCACCA 240
Bhutan-09024        CGGACATGTGGTGCTGTGCGACGATGGTGGGCCGCGGCCTAGAGGCCGTACTAGGCACCA 240
Bhutan-09027        CGGACATGTGGTGCTGTGCGACGATGGTGGGCCGCGGCCTAGAGGCCGTACTAGGCACCA 240
Bhutan-09030        CGGACATGTGGTGCTGTGCGACGATGGTGGGCCGCGGCCTAGAGGCCGTACTAGGCACCA 240
Bhutan-09005        CGGCGGAAGAGCATGGTTCAAGGAAGTCAGGTAACGGCCCTAAGACCGGATTAAGCCCCG 240
Indonesia-88035     AAAACATGTGATGCTTCGTGGCTTTAGTGAACCGTAGCCAAGTGGTTATTCCGGATAATA 240
Indonesia-88045     AAAACATGTGATGCTTCGTGGCTTTAGTGAACCGTAGACATGTGGCTTTTCCGGATAATA 240
Indonesia-88065     CGGCGGAAGAGCATGGTTCAAGGAAGTCAGGTAACGACTCAGATACCAGATTAAGCACCG 240


Bhutan-09015        CT 242
Bhutan-09024        CT 242
Bhutan-09027        CT 242
Bhutan-09030        CT 242
Bhutan-09005        CA 242
Indonesia-88035     CT 242
Indonesia-88045     AT 242
Indonesia-88065     CA 242
```

Supplementary Figure S9.

```
CLUSTAL 2.1 multiple sequence alignment


Bhutan-09015         TG 2
Bhutan-09024         AA 2
Bhutan-09027         AA 2
Bhutan-09030         TG 2
Bhutan-09005         AG 2
Indonesia-88035      AG 2
Indonesia-88045      AG 2
Indonesia-88065      AG 2
```

Supplementary Figure S10.

```
CLUSTAL 2.1 multiple sequence alignment


Bhutan-09015         SSRRRSMYRYKRWMKMWYKSRSRYRKWYRMYSWYMWKRKYSMYKWKMRKWRSWSMWWKYR 60
Bhutan-09024         SSRRRSMYRYKRWMKMWYKSRSRYRKWYRMYSWYMWKRKYSMYKWKMRKWRSWSMWWKYR 60
Bhutan-09027         SSRRRSMYRYKRWMKMWYKSRSRYRKWYRMYSWYMWKRKYSMYKWKMRKWRSWSMWWKYR 60
Bhutan-09030         SSRRRSMYRYKRWMKMWYKSRKRYRKWYRMYSWYMMKRKYSMYKWKMRKWRSWSMWWKYR 60
Bhutan-09005         YMMMMMWSRKWSMSWWRKWYSSSSWYRMKYSMRMYYSMSMMRWRRSYKWYWKYYWMRYMM 60
Indonesia-88035      YMMMMMWSSKWSMSWWRKWYSSSSWYRMKYSMRMYYYMSMMRWRRSYKKYWKYYWMRYMM 60
Indonesia-88045      YMMMMMWSSKWSMSWWRKWYSKSSWYRMKYSMRMYYYMSMMRWRRSYKKYWKYYWMRYMM 60
Indonesia-88065      YMMMMMWSRKWSMSWWRKWYSSSSWYRMKYSMRMYYSMSMMRWRRSYKWYWKYYWMRYMM 60
                                          .      :         .   :: . : : .:

Bhutan-09015         MYKWWWSWKMRRMRRWRRRYWYKYYSSSYMWKYYKSKKRMYYWWYMMWRKSYMKMRMRYY 120
Bhutan-09024         MYKWWWSWKMRRMRRWRRRYWYKYYSSSYMWKYYKSKKRMYYWWYMMWRKSYMKMRMRYY 120
Bhutan-09027         MYKWWWSWKMRRMRRWRRRYWYKYYSSSYMWKYYKSKKRMYYWWYMMWRKSYMKMRMRYY 120
Bhutan-09030         MYKWWWSWKMRRMRRWRRRYWYKYYSSSYMWKYYKSKKRMYYWWYMMWRKSYMKMRMRYY 120
Bhutan-09005         WSWKYYRMYWWWWSSYSKKKRSYWWMKRKSYRMMRRRKWSMMMKWWRRMWKMYWWMYMWM 120
Indonesia-88035      WSWKYYRMYWWWWSSYSKKKRSYWWMKRKSYRMMRRRYWSMMMKWWRRMWKMYWWMYMWM 120
Indonesia-88045      WSWKYYRMYWWWWSSYSKKKRSYWWMKRKSYRMMRRRYWSMMMKWWRRMWKMYWWMYMWM 120
Indonesia-88065      WSWKYYRMYWWWWSSYSKKKRSYWWMKRKSYRMMRRRKWSMMMKWWRRMWKMYWWMYMWM 120
                         ::         : ::    :: .   ::  : :       :     .       :

Bhutan-09015         KWRYSYRWYWKRRMYKWYWYMWSMMYSMSSKSYRKRRWRKKKMMKSRWMSRKMYSMKSYM 180
Bhutan-09024         KWRYSYRWYWKRRMYKWYWYMWSMMYSMSSKSYRKRRWRKKKMMKSRWMSRKMYSMKSYM 180
Bhutan-09027         KWRYSYRWYWKRRMYKWYWYMWSMMYSMSSKSYRKRRWRKKKMMKSRWMSRKMYSMKSYM 180
Bhutan-09030         KWRYSYRWYWKRRMYKWYWYMWSMMYSMSSKSYRKRRWRKKKMMKSRWMSRKMYSMKSYM 180
Bhutan-09005         WKRMYMKMWKWKKSMRYSYMSKKRWWYRRKRKMKYKWKKYYSWRSRMRWYSWYMKRSRMM 180
Indonesia-88035      WKKMYMKMWKWKKSMRYSYMSKKRWWYRRKRSMKYKWKKYYSWRSRMRWYSWYMKRSRMR 180
Indonesia-88045      WKKMYMKMWKWKKSMRYSYMSKKRWWYRRKRSMKYKWKKYYSWRSRMRWYSWYMKRSRMR 180
Indonesia-88065      WKRMYMKMWKWKKSMRYSYMSKKRWWYRRKRKMKYKWKKYYSWRSRMRWYSWYMKRSRMR 180
                       :   : :  ::  :: :   .  :   .:. : :  :  .  .         . .

Bhutan-09015         MKRYWKWYWYYYSWYRM 197
Bhutan-09024         MKRYWKWYWYYYSWYRM 197
Bhutan-09027         MKRYWKWYWYYYSWYRM 197
Bhutan-09030         MKRYWKWYWYYYSWYRM 197
Bhutan-09005         WWKWMRYMRSSMKYSKW 197
Indonesia-88035      WWKWMRYMRSSMKYSKW 197
Indonesia-88045      WWKWMRYMRSSMKYSKW 197
Indonesia-88065      WWKWMRYMRSSMKYSKW 197
                       :: ::     .: :
```
